# Green-Synthesized Gold Nanoparticles Using *Pfaffia glomerata* Extract Improve Maternal Hypertension and Angiogenic Markers in Pregnant Hypertensive Rats

**DOI:** 10.3390/pharmaceutics18091074

**Published:** 2026-08-27

**Authors:** Maria Medina de Azevedo, Maria Luiza Fidelis da Silva, Gabriela Pereira da Silva, Joyner David Anaya Miranda, Annye Vitória Moraes, Luana Ale Bertoncello Pael, Telma Lélia Gonçalves Schultz de Carvalho, Giselle Nathaly Calaça, Thainá Aparecida Rafael Silva, Bianca Viana Silva, Thaylla Bianca de Almeida Vilela, João Pedro Vilella Neto, Ariany Carvalho dos Santos, Ana Paula de Carlos Sela, Monique Assis de Vasconcelos Barros, Francislaine Aparecida dos Reis Lívero, Otávio Akira Sakai, Érica Marusa Pergo Coelho, Arquimedes Gasparotto Junior

**Affiliations:** 1 Laboratory of Cardiovascular Pharmacology (LaFaC), Faculty of Health Sciences, Federal University of Grande Dourados (UFGD), Dourados 79804-970, MS, Brazil; mariamedinaazevedo@outlook.com (M.M.d.A.); m.alufidelis@hotmail.com (M.L.F.d.S.); gabriela.pereirasilva01@gmail.com (G.P.d.S.); joynerdavid-10@hotmail.com (J.D.A.M.); annye_mrs@icloud.com (A.V.M.); luanaalee@hotmail.com (L.A.B.P.); telmaschultz@yahoo.com.br (T.L.G.S.d.C.); bianca.viana.vs@gmail.com (B.V.S.); thayllaba@gmail.com (T.B.d.A.V.); joaovilella.site@gmail.com (J.P.V.N.); arianysantos@ufgd.edu.br (A.C.d.S.); moniquebarros@ufgd.edu.br (M.A.d.V.B.); 2 Laboratory of Chromatographic and Spectroscopic Analysis (LACE), Federal Institute of Paraná (IFPR), Umuarama 87507-014, PR, Brazil; giselle.trindade@ifpr.edu.br (G.N.C.); thaina.rafael97@gmail.com (T.A.R.S.); otavio.sakai@ifpr.edu.br (O.A.S.); 3 Graduate Program in Biotechnology Applied to Agriculture, Paranaense University, Umuarama 87502-210, PR, Brazil; ana.sela@edu.unipar.br; 4 Laboratory of Cardiometabolic Pharmacology, Federal University of Paraná (UFPR), Curitiba 80060-000, PR, Brazil; francislaine@ufpr.br; 5 Department of Agricultural Sciences, Maringa State University, Umuarama 87508-210, PR, Brazil; empcoelho@uem.br

**Keywords:** Amaranthaceae, Brazilian ginseng, hypertension, nanotechnology

## Abstract

**Background:** Hypertensive disorders of pregnancy present a major global health challenge. Green nanotechnology offers a strategy to enhance the stability and efficacy of bioactive natural products. This study evaluated gold nanoparticles green-synthesized with *Pfaffia glomerata* extract (AuNPs-PG) against gestational hypertension and fetal complications in spontaneously hypertensive rats (SHRs). **Methods:** *P. glomerata* root extract was characterized by UHPLC-MS/MS. AuNPs-PG were green-synthesized with 2 mmol/L HAuCl_4_ (1:5 *v*/*v*) at 40 °C and characterized by UV-Vis and DLS. Pregnant Wistar-Kyoto and SHRs were divided into naive, negative control (NC), amlodipine (5 mg/kg), and AuNPs-PG groups (0.03, 0.1, and 0.3 mg/kg) treated daily on gestational days 1–18 to evaluate maternal cardiovascular, renal, biochemical, and reproductive outcomes. **Results:** UHPLC-MS/MS identified 38 compounds. AuNPs-PG showed a plasmon band at 520–550 nm and 88.1 nm hydrodynamic diameter. In SHRs, 0.3 mg/kg AuNPs-PG reduced systolic blood pressure by 18.5% and mean arterial pressure by 15.4%, while mitigating vascular dysfunction by lowering phenylephrine vasoconstriction by 52.7% and boosting acetylcholine vasodilation by 239.3%. Treatment shortened QTc interval by 26.4%, normalized T-wave inversion, and restored renal excretion, increasing urinary volume by 75.0% and sodium by 94.2%. Furthermore, AuNPs-PG elevated placental growth factor by 180.9%, lowered malondialdehyde by 63.5%, and preserved placental histology. Consequently, mean fetal weight increased by 21.6%, implantation index by 50.5%, and offspring-to-mother ratio by 129.6%. **Conclusions:** AuNPs-PG (0.3 mg/kg) effectively mitigate maternal and fetal complications in gestational hypertension. Gold nanoparticles serve as nanocarriers, whereas surface-adsorbed phytochemicals drive the biological effects.

## 1. Introduction

Gestational Hypertensive Syndromes (GHSs) constitute a group of hypertensive disorders that occur during pregnancy, including chronic hypertension superimposed on pregnancy, gestational hypertension, preeclampsia, and eclampsia [1]. Currently, they represent a major public health challenge, affecting approximately 10% of all pregnancies worldwide [2]. The incidence of GHS has shown an upward trend, rising from 16.3 million to 18.8 million cases between 1990 and 2021, and it remains one of the leading causes of maternal hospitalization and mortality [3,4].

Despite the increasing incidence of GHS cases, case fatality rates decreased by 30.5% in 2019 compared to 1990 [5]. This trend is attributed to the strengthening of health systems, facilitated by the training of healthcare professionals and improvements in outpatient care. These factors enable prompt diagnosis and specialized monitoring throughout gestation [6]. However, this progress is not uniform in low-income countries. In these settings, limited socioeconomic development hinders the provision of healthcare services, particularly specialized obstetric care. This subsequently impacts gestational follow-up and emergency care, contributing to clinical worsening in cases of GHS [7].

In this context, medicinal plants are particularly relevant, as they embody ancestral knowledge recognized by the World Health Organization as a cornerstone of primary healthcare [8]. Lack of access to both specialized care and standard pharmacological treatment drives populations to utilize medicinal plants and/or natural products as therapeutic tools [9]. However, evidence regarding the safety of medicinal plant use during gestation is often lacking, thereby exposing pregnant women to potential adverse effects [10].

*Pfaffia glomerata* (Spreng.) Pedersen (Amaranthaceae) is an endemic Brazilian plant traditionally used as a tonic, aphrodisiac, anti-inflammatory, and analgesic [11]. Chemically, *P. glomerata* is characterized by the presence of ecdysteroids—most notably 20-hydroxyecdysone (beta-ecdysone), the primary compound identified in its roots—as well as triterpenes, saponins, flavonoids, anthraquinones, tannins, coumarins, alkaloids, and polysaccharides [11,12,13].

Previous pharmacological studies have demonstrated that various preparations obtained from *Pfaffia glomerata* exhibit significant antioxidant [14,15] and anti-inflammatory properties, which are intricately linked to the activation of the endothelial nitric oxide/soluble guanylate cyclase (NO/sGC) pathway [16,17]. By enhancing NO-mediated signaling and mitigating oxidative stress, these bioactive compounds exert potential vasodilatory and endothelial-protective actions, providing a biological rationale for their application in cardiovascular dysfunction. Beyond its vascular effects, *P. glomerata* possesses a broad spectrum of other pharmacological activities, including well-documented anxiolytic and antidepressant effects [18], gastroprotective action [19], and complex modulatory roles in male reproductive function [20,21,22]. However, elevated blood pressure during pregnancy triggers severe systemic vascular complications, and despite *P. glomerata*’s promising endothelial protective profile, its safety and therapeutic index during pregnancy complicated by hypertension remain completely unstudied.

As a promising strategy to enhance the systemic absorption of plant extracts, nanotechnology enables advanced drug formulations by modulating release kinetics and protecting unstable phytocompounds from chemical degradation [23]. While traditional nanocarriers—such as liposomes, polymeric nanoparticles, solid lipid nanoparticles (SLNs), and nanoemulsions—are widely employed, they often present limitations, including low encapsulation efficiency for complex botanical extracts, unwanted burst release, and poor physical stability. In this context, gold nanoparticles (AuNPs) have emerged as a viable alternative. Owing to their unique surface chemistry, high surface-to-volume ratio, ease of functionalization, and remarkable stability, AuNPs enhance bioavailability, facilitate targeted delivery, and sustain therapeutic efficacy for natural products [24,25,26,27].

However, size plays a critical role in their biocompatibility: AuNPs smaller than 2 nm exhibit significantly higher toxicity than larger particles (such as 10–100 nm or 15–50 nm). Their reduced dimensions enable them to freely penetrate ~9 nm nuclear membrane pores and directly damage DNA. Furthermore, their increased surface area and altered electronic structure trigger high reactive oxygen species (ROS) production, while specific sizes between 1.4 and 1.5 nm irreversibly bind to and denature essential proteins, rendering ultra-small clusters (<2 nm) the highest cytotoxic risk [24,25].

Given the importance of developing research on the cardioprotective effects of plant species and ensuring safe natural products for use during gestation, the present study aimed to evaluate the safety and cardioprotective potential of AuNCs synthesized from the aqueous extract of *P. glomerata* (AuNCs-PP) in spontaneously hypertensive rats (SHR) subjected to continuous treatment during the gestational period.

## 2. Materials and Methods

### 2.1. Plant Material and Extract Preparation

The plant material was provided by the Association of Small Ginseng Producers of Querência do Norte (ASPAG). Samples were collected on Jujuí Island, Paraná, Brazil (23°05′04.54″ S, 53°37′30.74″ W). The botanical identity of *P. glomerata* was confirmed at the Anchieta Herbarium (PACA), located in the city of São Leopoldo, RS, Brazil, and affiliated with the Universidade do Vale do Rio dos Sinos (UNISINOS) and the Instituto Anchietano de Pesquisas. A voucher specimen was deposited in the PACA Herbarium under accession number PACA 118716. All procedures involving access to genetic heritage were conducted in accordance with Brazilian legislation (Law No. 13,123/2015) and registered in the National System for the Management of Genetic Heritage and Associated Traditional Knowledge (SisGen) under No. A7C536D. To prepare the *P. glomerata* aqueous root extract, 5 g of roots were combined with 100 mL of distilled water at 40 °C for 20 min. Subsequently, the extract was filtered through a 14 μm pore size filter paper via gravity filtration.

### 2.2. Phytochemical Characterization of the P. glomerata Aqueous Root Extract

After the extraction protocol, the extract was filtered through gauze, and a 1 mL aliquot of the sample was collected in a microcentrifuge tube and centrifuged at 3000 rpm. After centrifugation, the supernatant was collected and diluted to a concentration of 25 mg/mL using 80% analytical-grade methanol. The solution was then filtered through 0.45 µm nylon and PTFE syringe filters and transferred to vials for analysis by UHPLC-MS/MS (Nexera^®^ X2 HPLC coupled to a Shimadzu^®^ model 8050 MS). The injection volume was 1 µL. The mobile phases consisted of Milli-Q water acidified with 0.1% formic acid (A) and LC-MS grade methanol (Merck^®^, Darmstadt, Germany) (B). The system was operated at a flow rate of 0.5 mL/min under the following gradient conditions: 1–9 min (20% B), 10–15 min (40% B), and 16–30 min (10% B). Chromatographic separation was performed on a C18 column (5 µm, 150 × 4.6 mm, Shimadzu^®^, Kyoto, Japan) without a precolumn. Both the autosampler and column temperatures were maintained at 40 °C. The MS/MS detector was equipped with an electrospray ionization (ESI) source operating in both negative and positive modes. Data were acquired in multiple reaction monitoring (MRM) mode with a scanning rate of up to 30,000 u/s (up to 555 MRMs/s). Collision energy varied between 10 and 30 V depending on the transition. The flow rates for the nebulizing gas and drying gas were set to 3 L/min and 10 L/min, respectively, while the heating gas flow was 10 L/min. The interface voltage, current, and temperature were 3 kV, 7 µA, and 300 °C, respectively, and the desolvation temperature was 526 °C. Argon was used as the collision gas at a maximum pressure of 20 kPa. The dwell time ranged from 2 to 12 ms [28]. The limits of quantification ranged from 10 to 250 μg L^−1^, while the limit of detection was 5 μg L^−1^. Authentic analytical standards for all 38 phenolic compounds were individually analyzed under identical UHPLC-MS/MS conditions and used for method optimization, compound identification, and external calibration. No tentative identifications based solely on spectral libraries were made in this study.

### 2.3. Green Synthesis of Gold Nanoparticles Using P. glomerata Root Aqueous Extract

AuNCs-PP were synthesized using the plant extract diluted in distilled water at a 1:5 (*v*/*v*) ratio. The mixture was heated in a water bath at 40 °C, followed by the addition of a 2 mmol·L^−1^ HAuCl_4_ solution as the metallic precursor. The formation of AuNCs-PP was evidenced by a visual color transition of the reaction medium from red to purple. No post-synthesis purification step was performed; the as-synthesized AuNC-PP suspension was used directly for characterization and biological assays, as the phytochemicals from *P. glomerata* remained adsorbed onto the nanoparticle surface, acting as natural capping and stabilizing agents, which imparted suitable colloidal stability throughout the experimental period.

### 2.4. Characterization of AuNCs-PP

#### 2.4.1. Ultraviolet–Visible (UV-Vis) Spectroscopy

Spectroscopic analyses were performed using a UV–Vis spectrophotometer (NanoDrop™ One/OneC Microvolume, Thermo Fisher Scientific^TM^, Waltham, MA, USA) operating in the 400–800 nm wavelength range. The aqueous plant extract was diluted in distilled water (1:2 ratio, *v*/*v*) prior to analysis. Additionally, UV–Vis measurements were conducted to monitor the stability of gold nanoparticles synthesized using *P. glomerata* root aqueous extracts.

#### 2.4.2. Dynamic Light Scattering (DLS)

The hydrodynamic diameter of the synthesized AuNPs was determined by DLS using a Litesizer^TM^ 100 particle analyzer (Anton Paar, Graz, Austria). Samples were prepared by diluting 1 mL of the nanoparticle suspension in 5 mL of distilled water to achieve a transmittance between 50% and 90% for optimal measurement.

### 2.5. Pharmacological Studies

#### 2.5.1. Animals

Three-month-old SHRs (60 females and 20 males), weighing between 180 and 250 g, were obtained from the Central Animal Facility of the Federal University of Grande Dourados (UFGD). Additionally, Wistar–Kyoto rats (10 females and 4 males) were obtained from the Central Animal Facility of the Federal University of Mato Grosso do Sul (UFMS). The animals were housed at the FCS/UFGD research animal facility under controlled conditions, with a temperature of 22–25 °C and a 12 h light/dark cycle. For maintenance, the animals were kept in groups of 3 to 4 per rectangular rat cage (49 × 34 × 16 cm—length × width × height), using wood shavings as bedding, with food and water provided ad libitum. For the different experimental protocols, each group consisted of 10 individuals. All protocols were approved by the UFGD Ethics Committee on Animal Use under protocol no. 23022/2023.

#### 2.5.2. Experimental Design

##### Mating Procedures

At three months of age, the animals were mated in a harem system at a 3:1 female-to-male ratio for both SHR and Wistar–Kyoto strains. The selected animals were housed together at the onset of the dark cycle and separated at the end of the light cycle. To confirm pregnancy, vaginal secretions were collected to prepare smears for microscopic examination. Females were considered pregnant upon the detection of spermatozoa in the vaginal smear.

##### Experimental Groups and Treatments

Upon confirmation of pregnancy, the animals were assigned to the experimental groups described below and treated daily via oral gavage. Treatments were administered once daily at 08:00 a.m. for 18 consecutive days.

1. Naïve group (n = 10): Wistar–Kyoto rats receiving vehicle (filtered water, 1 mL/kg);
2. Negative control group (n = 10): SHRs receiving vehicle (filtered water, 1 mL/kg);
3. AuNCs-PP groups (n = 30; n = 10 per subgroup): SHRs receiving three different doses of the *P. glomerata* nanoformulation (0.03, 0.1, and 0.3 mg/kg);
4. Positive control group (n = 10): SHRs receiving amlodipine (5 mg/kg).

The sample size (n = 10) was defined based on previous experimental protocols using SHR to evaluate cardiorenal effects, adhering to the principle of reduction (3Rs) while ensuring sufficient statistical power (80% power at alpha = 0.05) to detect significant differences in blood pressure, urine volume, and electrolyte excretion [29].

The doses of AuNPs-PG (0.03, 0.1, and 0.3 mg/kg) were selected based on a semi-logarithmic geometric progression, a standard pharmacological methodology used to construct dose–response curves and determine the minimal effective dose. Furthermore, this sub-milligram dosage range (30–300 µg/kg) aligns with prior preclinical studies employing green-synthesized rutin-capped gold nanoparticles (e.g., 0.3 mg/kg, p.o.) [30], ensuring therapeutic efficacy while avoiding tissue accumulation and systemic toxicity during gestation.

During the entire treatment period, gestational and organogenic body weight gain (g) were monitored and recorded. Moreover, the animals were monitored daily for clinical signs of toxicity. Observations included changes in skin, fur, eyes, and mucous membranes, as well as autonomic and central nervous system alterations (e.g., excessive salivation, tremors, and abnormal reflexes). Additionally, locomotor activity, gait, posture, respiratory rate, food and water intake, and signs of gastrointestinal distress were continuously evaluated.

#### 2.5.3. Experimental Protocols

##### Renal Function

Renal function was evaluated according to the methodology described by Gasparotto Junior et al. (2009) [31]. Initially, on the morning of the experiments, all animals received 0.9% NaCl orally (5 mL/100 g body weight) to ensure uniform hydration. Following a 6 h fast on the 1st and 15th days of gestation, animals were placed in individual metabolic cages for 24 h urine collection. In the resulting samples, urine volume (mL/100 g), pH, and density were determined, along with the concentrations of sodium, potassium, and chloride.

##### Electrocardiography

On the 18th day of gestation, the animals were anesthetized via inhalation with isoflurane (2–3%) and placed in dorsal recumbency. Four electrodes were positioned on the wrists and ankles. A small amount of conductive gel was applied to each electrode interface to optimize electrical conductance. The PR, QRS, QT, and QTc intervals (ms) and P, Q, R, and S waves (mV) were recorded for five minutes using an electrocardiograph (WinCardio, Micromed, Brasília, Brazil), following an equivalent acclimation period.

##### Blood Pressure

Following the electrocardiogram, the animals received a subcutaneous injection of heparin (20 IU). Subsequently, while still under anesthesia (2–3% isoflurano), the left carotid artery was isolated and cannulated using a 24G catheter, which was coupled to a pressure transducer connected to a PowerLab system with LabChart 8.1.31 software (ADInstruments, Dunedin, New Zealand). After a 15 min period for hemodynamic stabilization, SBP, DBP, MAP, and HR were recorded for 5 min [32].

##### Blood Collection and Biochemical Analysis

Following blood pressure measurement, blood samples (4–5 mL) were collected from the previously cannulated carotid artery. The samples were transferred into serum separator tubes and centrifuged at 1000 rpm for 10 min. Serum levels of alanine aminotransferase (ALT), aspartate aminotransferase (AST), urea, creatinine, albumin, creatine kinase-MB (CK-MB), sodium, and potassium were measured using an automated biochemical analyzer (Cobas Integra 400 Plus; Roche, Basel, Switzerland). Nitrotyrosine (NT), placental growth factor (PlGF), and soluble fms-like tyrosine kinase-1 (sFlt-1) levels were determined by enzyme-linked immunosorbent assay (ELISA) using commercial kits (MyBioSource, San Diego, CA, USA). Malondialdehyde levels were determined using a thiobarbituric acid reactive substances (TBARS) assay (Cayman Chemical, Ann Arbor, MI, USA).

##### Mesenteric Vascular Bed Reactivity

Following hemodynamic measurements and prior to euthanasia, the MVBs were rapidly isolated and prepared for perfusion [33]. The entire intestine and its associated vascular bed were removed, and the mesenteric bed was carefully separated. Only the four main arterial branches of the superior mesenteric trunk located near the terminal ileum were utilized. The superior mesenteric artery was cannulated and gently flushed with physiological salt solution (PSS) to eliminate residual blood. The isolated MVBs were placed in glass organ baths, maintained at 37 °C, and aerated with carbogen (95% O_2_ and 5% CO_2_). The preparations were perfused at a constant flow rate of 4 mL/min with PSS using a peristaltic pump. The PSS composition was as follows: 119 mM NaCl, 4.7 mM KCl, 2.4 mM CaCl_2_, 1.2 mM MgSO_4_, 25.0 mM NaHCO_3_, 1.2 mM KH_2_PO_4_, 11.1 mM dextrose, and 0.03 mM EDTA. Perfusion pressure was monitored via a pressure transducer coupled to the perfusion system and connected to a computerized data acquisition system (LabChart 8.1.31; ADInstruments, Dunedin, New Zealand). After a 30 min stabilization period, the integrity of the preparation was verified by a bolus injection of KCl (120 mmol). Subsequently, a dose–response curve for phenylephrine (Phe; 1, 3, 10, and 30 nmol; 10–30 μL) was performed. Following another 30 min stabilization period, the preparations were continuously perfused with PSS containing 3 μM Phe to induce a sustained increase in perfusion pressure. Under these conditions, vascular reactivity changes induced by acetylcholine (ACh; 1, 3, 10, and 30 pmol; 10–30 μL) and sodium nitroprusside (SNP; 1, 3, 10, and 30 nmol; 10–30 μL) were investigated.

##### Reproductive Parameters

Following the removal of the MVB, the rats were laparotomized, and the uterine horns were removed. Subsequently, the following reproductive parameters were determined: offspring-to-mother ratio, ovary weight (mg/100 g), mean fetal weight (g), placental weight (g), number of corpora lutea, implantation index (%), resorption index (%), pre-implantation loss (%), and post-implantation loss (%). Additionally, external macroscopic features were evaluated to identify potential fetal malformations.

##### Relative Organ Weights, Morphometry, and Histopathological Analysis

Following euthanasia by anesthetic overdose with isoflurane (>40%), the liver, right kidney, uterus, and heart were removed, longitudinally sectioned, and cleaned. Subsequently, relative organ weights were determined using the following formula: [relative organ weight = (absolute organ weight (g)/total body weight (g)) × 100]. Portions of the cardiac tissue, aortic arch, placenta, liver, right kidney, uterus, and ovary were fixed in 10% buffered formalin. Samples were then dehydrated in graded alcohol, cleared with xylene, and embedded in paraffin. Histological sections (5 μm) were stained with hematoxylin and eosin (H&E) and examined by light microscopy. Data acquisition and analysis were performed using Motic Images Plus 2.0 software.

#### 2.5.4. Statistical Analysis

Data were evaluated for normality using the Shapiro–Wilk test and for homogeneity of variances using Levene’s test prior to statistical analysis. Differences among means were determined by one-way analysis of variance (ANOVA) followed by Bonferroni’s post hoc test. Results are expressed as the mean ± standard error of the mean (SEM). Statistical significance was defined as *p* < 0.05, with specific significance levels indicated as *p* < 0.05, *p* < 0.01, and *p* < 0.001. Graphs and statistical analyses were performed using GraphPad Prism version 10 for macOS (GraphPad Software, San Diego, CA, USA).

## 3. Results

### 3.1. Extraction Yield and Phytochemical Analysis of the P. glomerata Root Aqueous Extract

The aqueous extraction of *P. glomerata* roots at 5% (*w*/*v*), performed at 40 °C for 20 min, afforded a yield of 17%. Subsequently, the extract was analyzed by ultra-high-performance liquid chromatography coupled with tandem mass spectrometry (UHPLC-MS/MS) to identify the present secondary metabolites. LabSolutions Insight Explorer CSD v. 1.00 software (Shimadzu^®^) was used to quantify the detected phenolic compounds based on analytical curves (10–250 µg/L). Phytochemical analysis allowed the identification of 38 compounds, including catechol, morin, isovanillin, gallic acid, quercetin, hydroxybenzaldehyde, naringenin, syringaldehyde, chlorogenic acid, syringic acid, protocatechuic acid, vanillic acid, salicylic acid, vanillin, ferulic acid, p-hydroxybenzoic acid, naringin, p-coumaric acid, caffeic acid, coniferyl aldehyde, sinapic acid, syringaldazine, catechin, sinapaldehyde, luteolin, rutin, theobromine, epicatechin, baicalin, chrysin, quinic acid, malic acid, kaempferol, coumarin, caffeine, resorcylic acid, nicotinic acid, and fumaric acid. The detailed chromatographic data for each identified compound are presented in Table 1 and the Supplementary Materials.

### 3.2. Green Synthesis of Gold Nanoparticles Using P. glomerata Root Aqueous Extract

**Figure 1:**
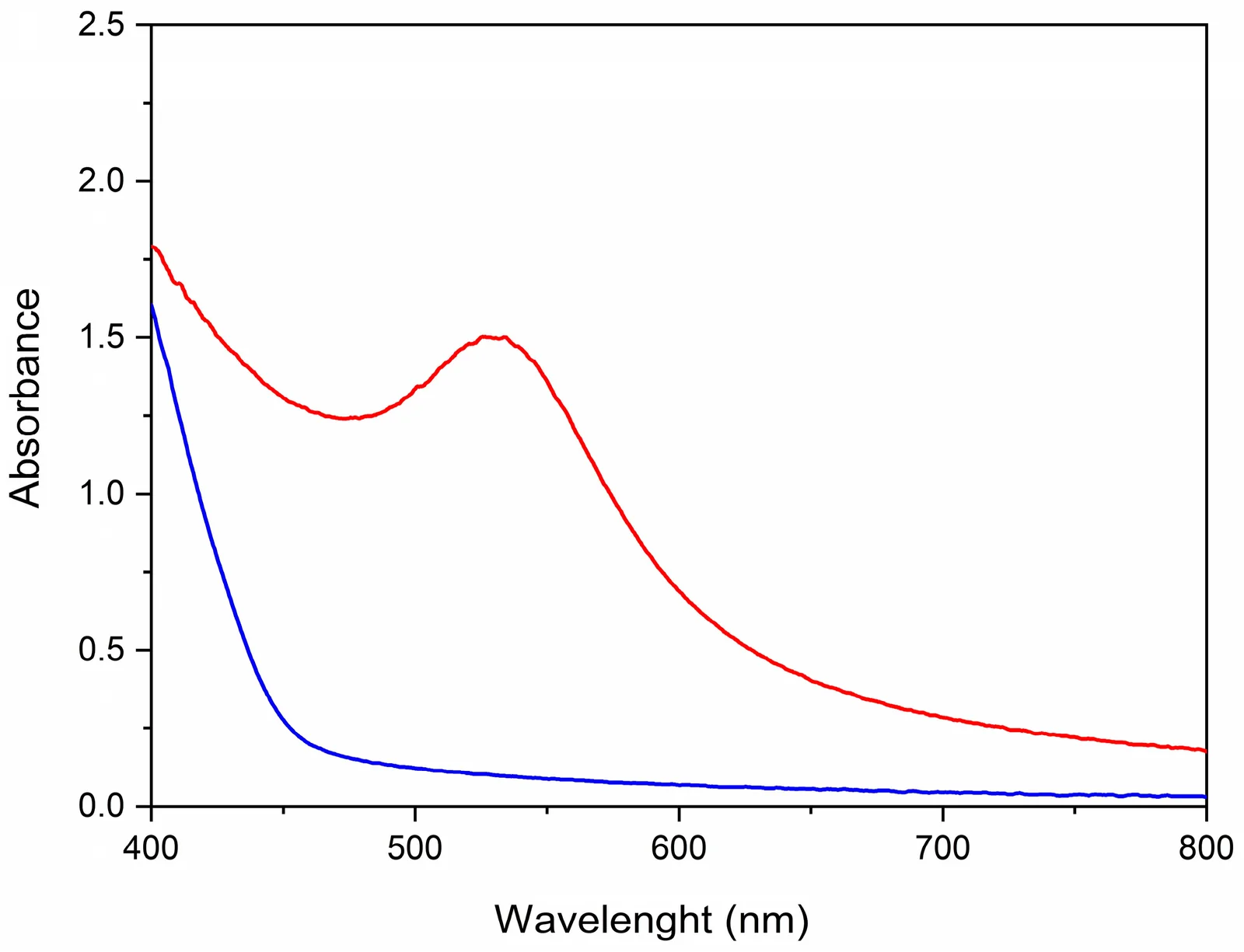
UV-Vis absorption spectra of *P. glomerata* aqueous root extract (blue) and synthesized gold nanoparticles (red) recorded from 400 to 800 nm.

The aqueous extract of *P. glomerata* (blue line) exhibited strong absorption in the near-ultraviolet (UV) region (~400 nm), followed by a sharp decline across the visible spectrum (Figure 1). This profile is characteristic of plant-derived organic compounds—such as phenolics, flavonoids, and saponins—that act as key reducing and stabilizing agents during green synthesis [34]. In contrast, the spectrum of the synthesized gold nanoparticles (red line) displayed a characteristic localized surface plasmon resonance (LSPR) band centered between 520 and 550 nm (Figure 1), confirming the successful formation of AuNPs [35].

**Figure 2:**
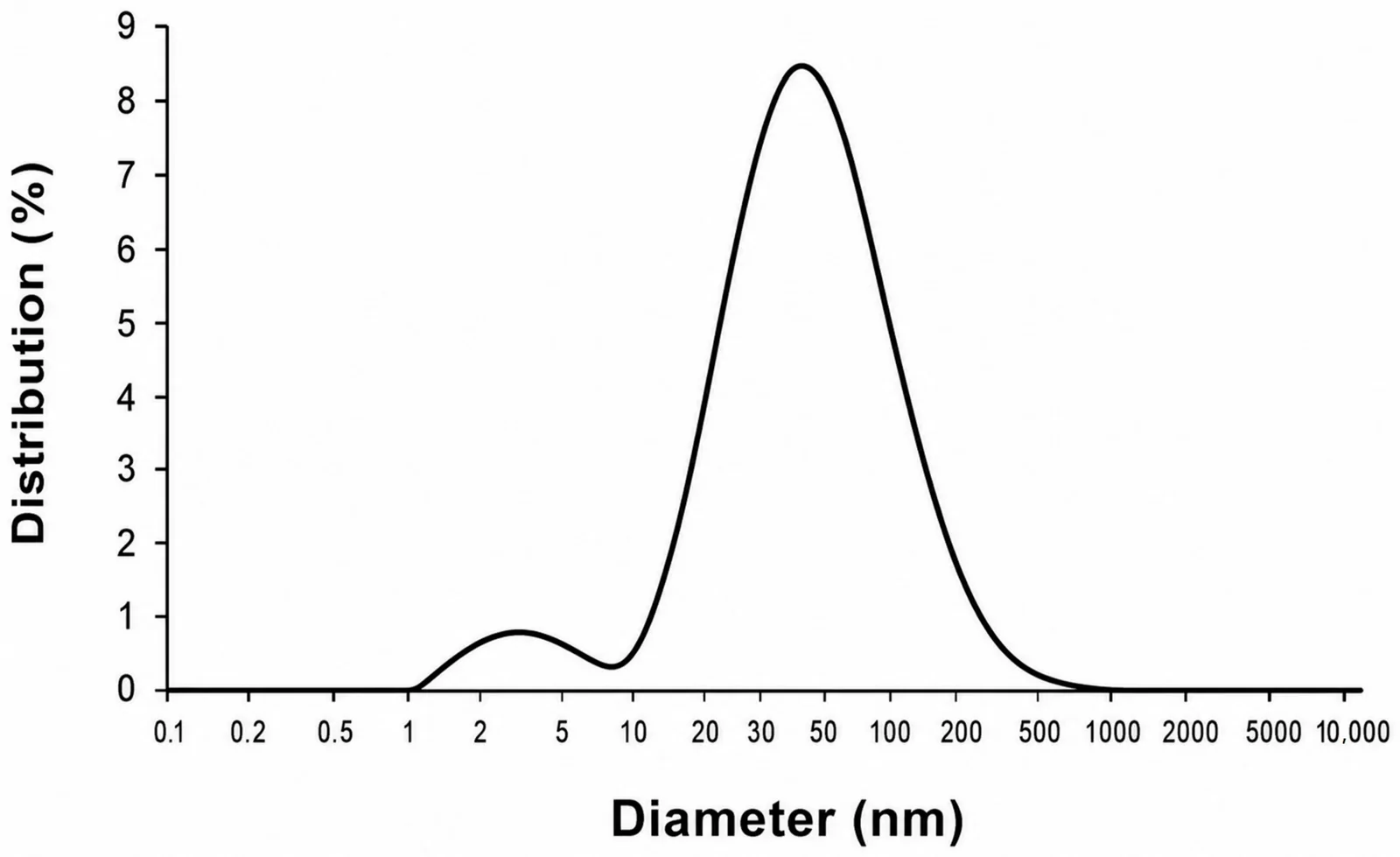
Size distribution of gold nanoparticles synthesized using *P. glomerata* root extract.

Furthermore, dynamic light scattering (DLS) analysis revealed a mean hydrodynamic diameter of 88.1 nm with a polydispersity index (PDI) of 0.23 (Figure 2). The hydrodynamic diameter measured by DLS encompasses the metallic core, the surrounding hydration layer, and the phytochemical capping shell adsorbed onto the nanoparticle surface. Consequently, DLS typically yields larger dimensions than the dry core size determined by transmission electron microscopy (TEM). These hydrodynamic measurements are consistent with the observed broadening of the LSPR band, indicating a well-dispersed colloidal system stabilized by biomolecules from the extract.

### 3.3. Renal Function

Table 2 presents data regarding urinary volume and renal electrolyte excretion collected over 24 h following treatments with either AuNCs-PP or amlodipine. On the first day of treatment and gestation, all SHR groups, regardless of treatment, showed a significant reduction in the renal excretion of sodium, potassium, and chloride compared to the naïve group. Urinary volume remained unchanged across all evaluated experimental groups.

In contrast, by the 15th day of treatment, pregnant SHRs treated with amlodipine or the highest dose of AuNCs-PP (0.3 mg/kg) exhibited a significant increase in 24 h urinary volume, as well as a significant elevation in renal sodium, potassium, and chloride excretion compared to the NC group. Animals treated with AuNCs-PP at doses of 0.03 and 0.1 mg/kg showed renal function similar to that observed in the NC group. No significant changes were observed in pH or urinary density when comparing all experimental groups.

### 3.4. Electrocardiography

Table 3 presents the electrocardiographic parameters of normotensive (naïve) and pregnant SHRs treated with AuNCs-PP (0.03, 0.1, and 0.3 mg/kg) or amlodipine on the 18th day of gestation. Negative control animals and those treated with the lowest doses of AuNCs-PP (0.03 and 0.1 mg/kg) exhibited QT and QTc interval prolongation (ms), as well as T-wave inversion (mV). Conversely, treatment with amlodipine or the highest dose of AuNCs-PP (0.3 mg/kg) prevented these alterations, maintaining QT, QTc, and T-wave values similar to those observed in the naïve group. No significant differences were observed in any other electrocardiographic parameters across the experimental groups.

### 3.5. Blood Pressure and Heart Rate

Table 4 presents the values for systolic blood pressure (SBP), diastolic blood pressure (DBP), mean arterial pressure (MAP), and heart rate (HR) obtained from normotensive (Wistar-Kyoto) and SHRs on the 18th day of gestation. All animals in the NC group showed a significant elevation in SBP, DBP, and MAP compared to the naïve group. Treatment with either amlodipine or AuNCs-PP at a dose of 0.3 mg/kg significantly reduced SBP, DBP, and MAP levels compared to the NC group. On the other hand, AuNCs-PP doses of 0.03 and 0.1 mg/kg were unable to induce any significant response in blood pressure levels when compared to SHRs treated only with the vehicle. HR levels were not significantly altered in any of the experimental groups evaluated.

### 3.6. Serum Biochemical Parameters

The serum biochemical parameters of pregnant Wistar–Kyoto (naïve) and SHRs treated with different doses of AuNCs-PP or amlodipine on the 18th day of gestation are presented in Table 5. All hypertensive rats in the NC group showed a significant increase in malondialdehyde (MDA) and soluble fms-like tyrosine kinase-1 (sFlt-1) levels, as well as a significant reduction in serum placental growth factor (PlGF) levels. Prolonged treatment with amlodipine and AuNCs-PP at a dose of 0.3 mg/kg was able to reverse the alterations in sFlt-1 and PlGF levels, maintaining values similar to those observed in the naïve group. Furthermore, only the treatment with AuNCs-PP (0.3 mg/kg) significantly reduced MDA levels, keeping them close to those obtained for the naïve rats. No other evaluated serum parameters showed significant changes when compared across all experimental groups.

### 3.7. Reactivity in the Mesenteric Vascular Bed (MVB)

Table 6 presents the data regarding the evaluation of mesenteric vascular reactivity, in which responsiveness was assessed following the administration of phenylephrine (Phe), acetylcholine (ACh), and sodium nitroprusside (SNP). Rats in the negative control group showed a significant increase in reactivity to 30 nmol of Phe, as well as a reduction in ACh-induced vasodilator activity at doses of 10 and 30 pmol when compared to animals in the naïve group. Treatment with AuNCs at a dose of 0.3 mg/kg and with amlodipine was able to prevent these changes, maintaining vascular reactivity similar to that obtained in the naïve group.

### 3.8. Effects on Gestational Development

Table 7 summarizes the reproductive parameters of pregnant Wistar–Kyoto (naïve) and SHRs on the 18th day of gestation following treatment with different doses of AuNCs-PP or amlodipine. Compared to the naïve group, the negative control group exhibited a significant reduction in the offspring-to-mother ratio, mean fetal weight, placental weight, and implantation index, alongside a higher resorption index. Treatment with amlodipine or AuNCs at 0.3 mg/kg significantly reduced these changes, maintaining fetal and placental weights, as well as implantation and resorption indices, at levels similar to those of the naïve group. Furthermore, only the highest dose of AuNCs (0.3 mg/kg) was capable of reversing the reduction in the offspring-to-mother ratio, restoring values to those similar to the naïve rats.

### 3.9. Changes in Relative Organ Weights

The relative organ weights of pregnant Wistar–Kyoto (naïve) and SHRs treated with different doses of AuNCs-PP or amlodipine on the 18th day of gestation are presented in Table 8. All hypertensive animals, regardless of treatment, showed a significant increase in relative liver and heart weights compared to the naïve group. Conversely, the relative uterine weight of animals in the negative control (NC) group and those treated with AuNCs-PP at doses of 0.03 and 0.1 mg/kg showed a significant reduction compared to the naïve rats. Treatment with amlodipine or AuNCs-PP at a dose of 0.3 mg/kg prevented this alteration, maintaining relative uterine weights similar to those observed in the naïve group. The relative weight of the right kidney remained unchanged across all experimental groups.

### 3.10. Histopathological Changes

**Figure 3:**
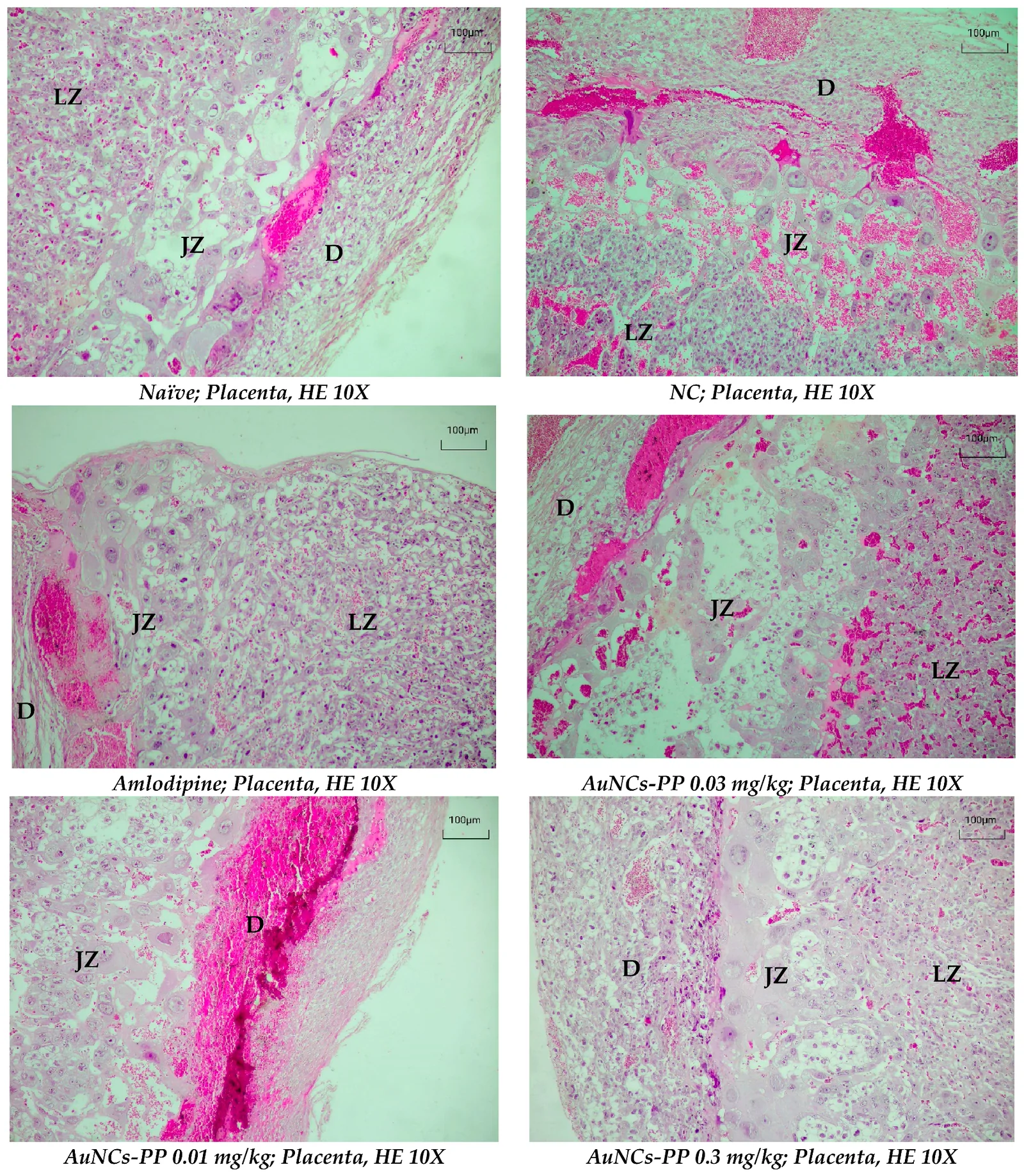
Representative photomicrographs of histological sections of the placenta from pregnant Wistar–Kyoto (naïve) and SHRs treated with different doses of AuNCs-PP or amlodipine on the 18th day of gestation. H&E: hematoxylin and eosin staining. D: decidual layer; JZ: junctional zone; LZ: labyrinth zone.

Figure 3 presents representative photomicrographs of histological placental sections from pregnant Wistar–Kyoto (naïve) and SHRs treated with various doses of AuNCs-PP or amlodipine on the 18th day of gestation. In placental samples from the NC groups and those treated with AuNCs-PP at doses of 0.01 and 0.03 mg/kg, areas of hemorrhage and dilation were observed in the decidua. Within the basal zone of these samples, an increased number of glycogen cells in pyknosis was also noted. Furthermore, dilation of the maternal sinusoids was present in the junctional and labyrinth zones. Treatment with amlodipine or the highest dose of AuNCs-PP (0.3 mg/kg) significantly reduced these alterations, maintaining a placental morphology similar to that of the naïve group. Analyses of cardiac tissue, aortic arch, liver, right kidney, uterus, and ovary samples revealed no significant histopathological changes across any experimental groups.

## 4. Discussion

The present study investigated the cardioprotective effects and reproductive safety of AuNCs-PP in a pregnancy SHR model. Continuous treatment with a 0.3 mg/kg dose effectively reduced SBP, DBP, and MAP. Furthermore, it prevented electrocardiographic alterations, such as QT/QTc interval prolongation and T-wave inversion. Chronic hypertension during pregnancy in SHRs induces both electrical and structural myocardial remodeling, driven by persistent pressure overload that promotes cardiomyocyte hypertrophy and interstitial fibrosis [36], ultimately altering the kinetics of potassium channels responsible for ventricular repolarization [37]. This delay in electrical recovery manifests as QT and QTc prolongation, indicating extended action potential duration and heightened vulnerability to arrhythmias [38]. Simultaneously, relative ischemia—stemming from increased cardiac muscle mass combined with coronary microvascular dysfunction—shifts the repolarization vector, yielding T-wave inversion as a classic hallmark of left ventricular overload and myocardial distress [39]. Treatment with 0.3 mg/kg AuNCs-PP effectively prevented these pathological changes by significantly lowering blood pressure, thereby decreasing cardiac afterload and normalizing the electrocardiographic profile.

During gestation, untreated SHRs exhibited a significant reduction in the urinary excretion of sodium, potassium, and chloride. Gestational hypertension compromises renal function via nephrosclerosis, wherein high-pressure blood flow damages renal arterioles and glomeruli, inducing vascular wall thickening and subsequent renal ischemia [40]. This elevated intraglomerular pressure impairs the filtration barrier, leading to proteinuria and the progressive replacement of functional nephrons with scar tissue (glomerulosclerosis). In response to compromised renal perfusion, the kidneys activate the renin–angiotensin–aldosterone system (RAAS), exacerbating systemic vasoconstriction and persistent inflammation. Over time, this cascade of structural and hemodynamic damage precipitates a progressive decline in the glomerular filtration rate (GFR) [41]. Notably, by the 15th day of gestation, treatment with 0.3 mg/kg AuNCs-PP exerted significant diuretic and natriuretic effects, increasing urinary volume and electrolyte excretion to levels comparable to amlodipine. From this perspective, AuNCs-PP significantly reduced blood pressure, likely through the modulation of renal ion transporters or by enhancing GFR via the controlled release of phytosteroids and saponins from *P. glomerata*, which are well recognized for their intrinsic diuretic properties [12,42].

The relationship between endothelial dysfunction and hypertension is defined by a self-perpetuating vicious cycle. The diminished capacity of the endothelium to produce key vasodilators, such as nitric oxide (NO), drives an increase in vascular resistance. Simultaneously, the mechanical stress exerted by high-pressure blood flow triggers localized inflammatory cascades and oxidative stress, which further impair the bioavailability of relaxing factors while stimulating the release of vasoconstrictors, such as endothelin-1 [43]. Hypertensive rats in this study demonstrated significant hypersensitivity to phenylephrine and impaired acetylcholine-induced vasodilation, confirming endothelial dysfunction. Remarkably, AuNCs-PP (0.3 mg/kg) counteracted this impairment, preserving vascular reactivity at levels comparable to the normotensive (naïve) control group.

Oxidative stress serves as a central mediator in this pathology by disrupting the critical balance between ROS production and endogenous antioxidant defenses. Excess ROS readily react with NO to form peroxynitrite, thereby elevating vascular tone and driving hypertrophic remodeling. Within this framework, hypertension exacerbates oxidative stress, which recursively elevates peripheral resistance and further aggravates systemic blood pressure [44]. Treatment with 0.3 mg/kg AuNCs-PP uniquely reduced MDA levels—a primary biomarker of lipid peroxidation—reflecting a significative reduction in oxidative damage to cell membrane lipids [45]. These findings indicate that AuNCs enhance the targeted delivery of intrinsic antioxidant compounds contained within the *P. glomerata* aqueous extract, enabling a more effective mitigation of lipid peroxidation and endothelial cell protection.

In the pregnant hypertensive model, oxidative stress acts as the primary trigger connecting placental ischemia to systemic angiogenic imbalance [46]. Excessive ROS production induces a significant release of anti-angiogenic factors, such as sFlt-1, alongside the downregulation of pro-angiogenic factors, including vascular endothelial growth factor and PlGF [47]. This anti-angiogenic state triggers widespread endothelial dysfunction, compromising NO bioavailability and ultimately raising total peripheral vascular resistance [48]. Accordingly, correlation analyses revealed a positive association between maternal blood pressure and the serum sFlt-1/PlGF ratio. This linear relationship indicates that elevated circulating levels of sFlt-1 relative to PlGF directly govern systemic hemodynamic resistance, establishing the sFlt-1/PlGF ratio not merely as a consequence of the disease, but as a sensitive molecular biomarker of hypertensive severity [49].

The interplay among hypertension, oxidative stress, and angiogenic dysregulation establishes a self-reinforcing feed-forward loop that accelerates placental histopathological damage through persistent hypoxia and chronic inflammation. Pathologically, this hostile microenvironment induces vascular wall thickening, parenchymal hemorrhage, sinusoidal dilation, and impaired trophoblast invasion of spiral arteries, severely compromising maternal-fetal perfusion [50]. In the untreated hypertensive group, this unmitigated vascular insult manifested as reduced fetal and placental weights, elevated resorption rates, and focal placental necrosis. Conversely, treatment with 0.3 mg/kg AuNCs-PP significantly lowered the sFlt-1/PlGF ratio—an anti-angiogenic rescue that positively correlated with blood pressure reduction, demonstrating that restoring angiogenic balance directly translates into systemic blood pressure normalization. By mitigating oxidative stress and restoring angiogenic homeostasis, AuNCs-PP optimized utero-placental hemodynamics, thereby ensuring sustained nutrient and oxygen delivery to the fetus and effectively preventing intrauterine growth restriction [51].

The pharmacological profile of *P. glomerata* regarding male reproductive health exhibits a complex dichotomy between its aphrodisiac potential and its impact on testicular structural integrity. While earlier research indicated that *P. glomerata* crude extract may impair male fertility in mice [19], accumulating evidence also highlights its capacity to mitigate bisphenol A-induced reproductive injury [20]. Recent findings demonstrate that the *P. glomerata* extract significantly enhances sexual behavior in male rodent models, as evidenced by a reduction in mount latency alongside a significant increase in mount frequency [52]. Furthermore, *P. glomerata* has demonstrated an ameliorative effect on paroxetine-induced sexual dysfunction, restoring serum testosterone and luteinizing hormone levels while improving erectile parameters and penile tissue pathology [20,52]. These aphrodisiac effects may be directly linked to an upregulation of testicular nitric oxide production and vasodilation [53].

Evidence suggests that the protective role of *P. glomerata* appears to be mediated by its significant antioxidant capacity. Treatment has been shown to enhance the enzymatic activities of key antioxidants, including superoxide dismutase, catalase, and glutathione peroxidase, while simultaneously reducing MDA content within testicular tissue [52]. Despite these therapeutic benefits, the safety profile of long-term or high-dose administration remains a point of concern. While several studies indicate that *P. glomerata* does not act as an endocrine disruptor when administered during early postnatal life [54,55], other data highlight potential deleterious effects. Long-term intake has been associated with impaired spermatogenesis through the induction of apoptosis and alterations in seminiferous tubule epithelial dynamics [56]. Additionally, chronic administration has been linked to decreased Leydig cell viability, subsequently reducing testosterone production [57]. In summary, while *P. glomerata* extract holds substantial promise as a sexual stimulant and a protective agent against specific reproductive toxins [19,20,57], its potential to induce testicular apoptosis and compromise cell viability [53,56] warrants careful dose–response evaluation in future clinical applications.

When focusing specifically on the nanoformulation evaluated in this study, our findings align closely with previous reports investigating AuNP-based therapies in pregnant rat models [58,59]. Metallic platforms—particularly AuNPs—have demonstrated efficacy in attenuating placental oxidative stress and endothelial dysfunction, largely attributable to their intrinsic anti-inflammatory and antioxidant properties [60]. However, plant-derived nanoparticles and green-synthesized nanoformulations offer distinct biocompatibility advantages, often acting synergistically through bioactive phytoconstituents that modulate intracellular signaling cascades—such as the MAPK/NF-κB pathways [61,62]—and restore vascular reactivity in hypertensive pregnancy models [63]. In comparison, the bio-nanoparticles evaluated here not only effectively reduced vascular resistance and systemic blood pressure, but also promoted structural and functional recovery at the maternal-fetal interface. This dual mechanism—combining targeted nano-delivery with the intrinsic vasorelaxant and cardioprotective actions of natural bioactives—highlights the compelling therapeutic advantages of green nanoplatforms over conventional metallic systems in counteracting hypertensive disorders of pregnancy.

UHPLC–MS/MS analysis (Table 1) revealed a diverse profile of phenolic acids, flavonoids, and redox-active metabolites in the *P. glomerata* extract [11,64,65]. These phytochemicals are essential for green synthesis, as plant secondary metabolites reduce Au^3+^ to metallic Au^0^ and act as capping agents to stabilize the nanoparticles [66]. Thus, characterizing the extract provides mechanistic insight into the formation, stabilization, and surface chemistry of the synthesized AuNPs.

Although malic and fumaric acids were the most abundant compounds, both are ubiquitous intermediates of the tricarboxylic acid (TCA) cycle involved in primary energy metabolism, carbon allocation, and redox homeostasis [67,68]. Their high concentration reflects baseline plant housekeeping rather than primary drivers of the observed pharmacological responses. In contrast, several identified phenolics possess well-established vascular and cardiovascular bioactivities. Ferulic, p-coumaric, protocatechuic, and caffeic acids are recognized for their antioxidant, anti-inflammatory, and endothelial-protective properties, largely through enhancing nitric oxide bioavailability and promoting vasodilation [69,70,71,72]. Additionally, lower-abundance compounds such as rutin and vanillin contribute established cardioprotective, vasorelaxant, and anti-inflammatory effects [73,74,75,76].

Rather than attributing biological activity to an individual constituent, this phytochemical profile highlights the multifunctional nature of the biosynthesized AuNPs. During synthesis, these bioactive metabolites facilitate gold reduction and remain functionalized onto the AuNP corona, likely acting synergistically to drive the therapeutic efficacy observed in vivo.

Despite the promising therapeutic potential and favorable safety profile of AuNCs-PP, several limitations of this study should be acknowledged. First, complementary physicochemical characterization techniques—such as transmission electron microscopy, scanning electron microscopy, X-ray diffraction, Fourier-transform infrared spectroscopy, and zeta potential measurements—were not performed. Although UV-Vis spectroscopy confirmed gold nanoparticle formation via the characteristic LSPR band, and DLS determined the hydrodynamic size and polydispersity, these methods alone cannot resolve nanoparticle morphology, crystalline structure, capping chemistry, or surface charge. Consequently, these comprehensive analyses will be integrated into future investigations to fully characterize the biosynthesized gold nanoparticles. Furthermore, further investigation is required to fully elucidate the specific molecular signaling pathways responsible for the restoration of angiogenic balance. Finally, future studies should focus on mapping the complete pharmacokinetics of the nanoformulation, evaluating long-term postnatal outcomes in offspring, and establishing clinical efficacy in humans to consolidate green nanotechnology and natural bioactives as a viable therapeutic strategy for hypertensive disorders of pregnancy.

## 5. Conclusions

In conclusion, continuous treatment with AuNPs-PG, particularly at the dose of 0.3 mg/kg, proved safe during pregnancy and effective in mitigating maternal-fetal complications in SHRs, with the nanoparticles acting as nanocarriers and the surface phytochemicals driving the biological effects. This intervention promoted a reduction in maternal blood pressure, prevention of electrocardiographic alterations, restoration of renal function, attenuation of oxidative stress, and normalization of the angiogenic balance, thereby preserving placental microarchitecture and reestablishing fetal weight and implantation indices without inducing toxicity. However, the study presents limitations, including the need for full elucidation of the underlying molecular signaling pathways, detailed characterization of the pharmacokinetic and biodistribution profiles of the nanoformulation during pregnancy, evaluation of long-term postnatal impacts on offspring development, and the execution of translational studies and human clinical trials before this biotechnology can be translated into clinical practice.

## Figures and Tables

**Table 1:** Identification and quantification of phenolic compounds in the *P. glomerata* root aqueous extract.

	Compound λ = 100–700 nm	Mass g/mol	Ionization Mode	Transitions (*m*/*z*)	Retention Time (min)	[0.5 g/100 mL] (ppb) *	[0.5 g/100 mL] (ppb) **	[10 g/100 mL] (ppb) *	[10 g/100 mL] (ppb) **
1	Caffeic acid	180.1	negative	179.2 > 135.0	8.3	-	-	30.36	204.46
2	Chlorogenic acid	354.3	negative	353.1 > 191.1	7.9	50.56	60.29	54.79	2025.57
			positive	354.8 > 162.9	7.9	20.67	22.05	26.87	741.32
3	Ferulic acid	194.1	negative	193.2 > 133.9	8.9	28.40	20.37	153.70	99.43
4	Fumaric acid	116.0	negative	115.2 > 71.0	5.5	74.35	68.24	499.57	116.0
5	Gallic acid	170.1	negative	169.2 > 124.9	6.6	-	-	-	-
6	Malic acid	134.0	negative	133.2 > 114.9	3.9	1932.95	2101.80	1942.99	8891.44
7	Nicotinic acid	123.1	negative	122.4 > 77.9	4.6	17.10	18.75	57.27	55.14
			positive	123.9 > 79.8	4.6	26.88	23.99	136.82	122.65
8	p-coumaric acid	164.1	negative	163.2 > 119.0	8.8	-	-	13.45	17.20
9	p-hydroxybenzoic acid	138.1	negative	137.2 > 92.9	8.3	32.90	23.95	74.31	55.14
10	Protocatechuic acid	154.1	negative	153.2 > 108.9	7.6	-	-	138.73	76.22
11	Quinic acid	192.1	negative	191.2 > 84.8	3.6	-	-	67.52	172.15
12	Resorcylic acid	154.1	negative	153.2 > 108.9	7.5	-	-	-	-
13	Salicylic acid	138.1	negative	137.4 > 92.9	9.8	<LOQ	<LOQ	<LOQ	<LOQ
14	Syringic acid	198.2	negative	197.2 > 182.0	8.4	-	-	-	25.26
15	Sinapic acid	224.2	negative	223.2 > 208.0	8.9	-	-	-	-
			positive	225.0 > 206.9	8.9	-	-	-	-
16	Vanillic acid	168.1	negative	167.4 > 152.0	8.4	37.51	40.83	23.58	65.19
17	Baicalin	446.3	negative	445.2 > 269.1	9.6	-	-	-	-
			positive	446.9 > 270.9	9.6	-	-	-	-
18	Catechol	110.1	negative	109.4 > 90.9	8.0	-	-	-	-
19	Catechin	290.2	positive	290.8 > 138.9	7.6	-	-	-	-
			negative	289.1 > 245.1	7.6	-	-	-	-
20	Caffeine	194.1	positive	194.9 > 137.9	8.5	35.76	57.31	40.60	45.93
21	Chrysin	254.2	negative	253.0 > 143.0	10.9	-	-	-	-
			positive	254.9 > 152.8	10.9	-	-	-	-
22	Coniferyl aldehyde	178.1	negative	177.2 > 162.0	9.1	-	-	-	-
			positive	178.9 > 90.9	9.1	-	-	-	-
23	Coumarin	146.1	positive	146.8 > 90.9	9.4	<LOQ	<LOQ	<LOQ	<LOQ
24	Epicatechin	290.2	negative	289.1 > 245.1	7.63	-	-	-	-
25	Hydroxybenzaldehyde	122.1	negative	121.4 > 91.9	8.5	-	-	-	<LOQ
			positive	123.1 > 76.9	8.5	13.18	10.67	17.20	25.19
26	Isovanillin	152.1	positive	153.1 > 65.0	8.6	<LOQ	<LOQ	<LOQ	<LOQ
27	Luteolin	286.2	negative	285.0 > 133.0	9.9	-	-	<LOQ	<LOQ
			positive	286.9 > 152.8	9.9	-	-	-	-
28	Kaempferol	286.2	negative	285.1 > 229.1	10.1	-	-	-	-
			positive	286.8 > 152.9	10.1	-	-	-	-
29	Morin	302.2	positive	302.8 > 152.9	9.7	-	-	-	-
30	Naringin	580.5	negative	579.0 > 271.1	8.8	-	-	-	<LOQ
31	Naringenin	272.2	negative	271.1 > 151.0	9.8	-	-	-	<LOQ
			positive	273.0 > 152.9	9.8	-	-	-	-
32	Quercetin	302.2	negative	301.1 > 151.0	9.7	-	-	-	-
33	Rutin	610.5	negative	609.0 > 300.0	8.9	<LOQ	<LOQ	<LOQ	52.08
34	Syringaldazine	360.3	negative	359.1 > 344.1	9.8	-	-	-	-
			positive	361.0 > 182.0	9.8	-	-	-	-
35	Syringaldehyde	182.1	positive	183.0 > 94.9	8.7	19.15	18.47	17.40	23.72
36	Sinapaldehyde	208.2	negative	207.2 > 177.0	9.0	-	-	<LOQ	<LOQ
			positive	209.0 > 54.9	9.0	-	-	-	-
37	Theobromine	180.1	positive	181.0 > 66.9	7.5	-	-	-	-
38	Vanillin	152.1	negative	151.4 > 136.0	8.8	35.18	35.44	57.05	119.82

* Diluted in water; ** Diluted in methanol; ppb: parts per billion. Compounds with concentrations below the limit of quantification (LOQ) were reported as “<LOQ” (or “<10 ppb”) and were considered qualitatively detected but not quantitatively reliable. No statistical comparisons were performed for the concentrations of the identified compounds, as the UHPLC-MS/MS analysis was intended solely for phytochemical characterization of the *P. glomerata* extract. Statistical analyses were performed only for the biological outcome variables.

**Table 2:** A 24 h urinary volume (mL/100 g body weight) and renal excretion of sodium, potassium, and chloride of pregnant Wistar-Kyoto (naïve) and SHRs treated with different doses of AuNCs-PP or amlodipine on the 15th day of gestation.

Parameter	Naïve	NC	Amlodipine	AuNCs-PP 0.03 mg/kg	AuNCs-PP 0.1 mg/kg	AuNCs-PP 0.3 mg/kg
**Day 1**						
Urinary volume (mL/100 g/24 h)	3.26 ± 0.43	4.21 ± 0.77	3.36 ± 0.62	3.47 ± 0.83	4.44 ± 1.66	3.61 ± 0.68
Sodium (µmol/100 g/24 h)	1223.7 ± 182.3	**822.7 ± 89.7 ^a^**	**852.4 ± 81.7 ^a^**	**829.1 ± 84.2 ^a^**	**844.5 ± 90.1 ^a^**	**816.7 ± 88.8 ^a^**
Potassium (µmol/100 g/24 h)	831.8 ± 89.5	**516.5 ± 52.6 ^a^**	**509.2 ± 52.2 ^a^**	**499.5 ± 59.7 ^a^**	**494.1 ± 52.1 ^a^**	**512.3 ± 59.7 ^a^**
Chloride (µmol/100 g/24 h)	1021.8 ± 133.5	**621.9 ± 66.1 ^a^**	**600.3 ± 66.8 ^a^**	**633.5 ± 60.7 ^a^**	**619.2 ± 59.9 ^a^**	**631.9 ± 66.6 ^a^**
**Day 15**						
Urinary volume (mL/100 g/24 h)	3.54 ± 0.54	4.12 ± 0.22	**9.41 ± 2.82 ^ab^**	5.29 ± 0.26	4.76 ± 0.39	**7.21 ± 1.12 ^ab^**
Sodium (µmol/100 g/24 h)	1551.1 ± 175.9	**800.1 ± 80.1 ^a^**	**1612.2 ± 180.2 ^b^**	**772.5 ± 78.5 ^a^**	**812.1 ± 88.6 ^a^**	**1553.8 ± 166.8 ^b^**
Potassium (µmol/100 g/24 h)	899.2 ± 93.3	**601.4 ± 66.1 ^a^**	**912.1 ± 90.1 ^b^**	**553.6 ± 60.7 ^a^**	**612.4 ± 68.8 ^a^**	**884.5 ± 88.6 ^b^**
Chloride (µmol/100 g/24 h)	1121.1 ± 125.4	**712.3 ± 72.3 ^a^**	**1200.4 ± 133.3 ^b^**	**766.6 ± 84.5 ^a^**	**732.1 ± 77.4 ^a^**	**1155.8 ± 129.5 ^b^**

Statistical analyses were performed using one–way ANOVA followed by Bonferroni’s post hoc test. Values are expressed as mean ± SEM (standard error of the mean). n = 10 rats per group. ^a^ *p* ≤ 0.05 when compared to naïve group. ^b^ *p* ≤ 0.05 when compared to negative control (NC) group.

**Table 3:** Electrocardiographic parameters of pregnant Wistar-Kyoto (naïve) and SHRs treated with different doses of AuNCs-PP or amlodipine on the 18th day of gestation.

Parameter	Naïve	NC	Amlodipine	AuNCs-PP 0.03 mg/kg	AuNCs-PP 0.1 mg/kg	AuNCs-PP 0.3 mg/kg
PR segment (ms)	39.25 ± 3.47	36.90 ± 2.20	40.11 ± 2.24	40.33 ± 3.18	37.43 ± 3.11	40.12 ± 3.11
QRS complex (ms)	39.55 ± 3.43	38.70 ± 2.78	38.33 ± 2.62	38.00 ± 2.94	39.13 ± 2.06	39.50 ± 2.70
QT interval (ms)	89.00 ± 4.95	**122.01 ± 4.55 ^a^**	**85.22 ± 4.43 ^b^**	**130.33 ± 6.55 ^a^**	**132.87 ± 5.68 ^a^**	**80.88 ± 6.32 ^b^**
QTc interval (ms)	207.8 ± 11.45	**274.1 ± 11.78 ^a^**	**197.6 ± 10.11 ^b^**	**283.67 ± 16.13 ^a^**	**279.12 ± 18.12 ^a^**	**201.77 ± 12.21 ^b^**
P wave (mV)	0.07 ± 0.02	0.08 ± 0.02	0.06 ± 0.02	0.08 ± 0.02	0.07 ± 0.02	0.07 ± 0.02
Q wave (mV)	−0.03 ± 0.01	−0.02 ± 0.01	−0.02 ± 0.01	−0.02 ± 0.01	−0.02 ± 0.01	−0.02 ± 0.01
R wave (mV)	0.33 ± 0.17	0.35 ± 0.12	0.32 ± 0.12	0.35 ± 0.14	0.34 ± 0.12	0.36 ± 0.12
S wave (mV)	−0.02 ± 0.01	−0.02 ± 0.01	−0.03 ± 0.01	−0.03 ± 0.01	−0.02 ± 0.01	−0.01 ± 0.01
T wave (mV)	0.03 ± 0.01	**−0.02 ± 0.01 ^a^**	**0.02 ± 0.01 ^b^**	**−0.03 ± 0.01 ^a^**	**−0.02 ± 0.01 ^a^**	**0.02 ± 0.01 ^b^**

Statistical analyses were performed using one–way ANOVA followed by Bonferroni’s post hoc test. Values are expressed as mean ± SEM (standard error of the mean). n = 10 rats per group. ^a^ *p* ≤ 0.05 when compared to naïve group. ^b^ *p* ≤ 0.05 when compared to negative control (NC) group.

**Table 4:** Blood pressure and heart rate of pregnant Wistar-Kyoto (naïve) and SHRs treated with different doses of AuNCs-PP or amlodipine on the 18th day of gestation.

Parameter	Naïve	NC	Amlodipine	AuNCs-PP 0.03 mg/kg	AuNCs-PP 0.1 mg/kg	AuNCs-PP 0.3 mg/kg
SBP (mm Hg)	107.03 ± 7.84	**166.24 ± 7.12 ^a^**	**139.33 ± 9.33 ^ab^**	**172.05 ± 8.33 ^a^**	**158.36 ± 6.12 ^a^**	**135.42 ± 8.66 ^ab^**
DBP (mm Hg)	63.21 ± 5.12	**105.44 ± 8.16 ^a^**	**88.13 ± 8.24 ^ab^**	**107.21 ± 5.84 ^a^**	**99.53 ± 8.77 ^a^**	**84.33 ± 7.21 ^ab^**
MAP (mm Hg)	83.78 ± 7.81	**128.42 ± 9.35 ^a^**	**110.11 ± 8.23 ^ab^**	**134.62 ± 8.21 ^a^**	**128.41 ± 7.53 ^a^**	**108.62 ± 7.99 ^ab^**
HR (bpm)	322.64 ± 23.21	330.43 ± 23.59	331.54 ± 36.28	325.24 ± 28.33	325.52 ± 31.77	323.76 ± 27.13

Statistical analyses were performed using one–way ANOVA followed by Bonferroni’s post hoc test. Values are expressed as mean ± SEM (standard error of the mean). n = 10 rats per group. ^a^ *p* ≤ 0.05 when compared to naïve group. ^b^ *p* ≤ 0.05 when compared to negative control (NC) group. DBP: diastolic blood pressure; HR: heart rate; MAP: mean arterial pressure; SBP: systolic blood pressure.

**Table 5:** Serum biochemical parameters of pregnant Wistar–Kyoto (naïve) and SHRs treated with different doses of AuNCs-PP or amlodipine on the 18th day of gestation.

Parameter	Naïve	NC	Amlodipine	AuNCs-PP 0.03 mg/kg	AuNCs-PP 0.1 mg/kg	AuNCs-PP 0.3 mg/kg
ALT (U/L)	38.22 ± 3.45	40.15 ± 3.72	36.28 ± 3.27	38.82 ± 3.71	41.33 ± 3.52	37.44 ± 3.64
AST (U/L)	84.66 ± 5.66	89.27 ± 5.18	86.67 ± 5.51	90.32 ± 5.42	88.72 ± 5.13	87.37 ± 5.75
Urea (mg/dL)	37.18 ± 3.55	40.66 ± 3.57	39.33 ± 3.78	41.81 ± 3.44	36.22 ± 3.97	41.22 ± 3.17
Creatinine (mg/dL)	0.61 ± 0.12	0.67 ± 0.10	0.69 ± 0.11	0.69 ± 0.13	0.65 ± 0.11	0.64 ± 0.12
Albumin (g/dL)	3.55 ± 0.84	3.34 ± 0.92	3.25 ± 0.88	3.42 ± 0.75	3.49 ± 0.80	3.63 ± 0.97
Sodium (mg/dL)	125.45 ± 3.78	124.57 ± 4.12	127.11 ± 3.54	129.13 ± 4.78	131.11 ± 4.01	128.33 ± 4.55
Potassium (mg/dL)	5.12 ± 0.71	5.07 ± 0.62	5.19 ± 0.7197	5.12 ± 0.55	5.57 ± 0.87	5.60 ± 0.68
CK-MB (ng/mL)	5.75 ± 0.98	5.45 ± 0.77	5.62 ± 0.81	5.37 ± 0.71	5.57 ± 0.66	5.71 ± 0.91
NT (μmol/L)	0.011 ± 0.003	0.013 ± 0.003	0.012 ± 0.002	0.013 ± 0.002	0.011 ± 0.002	0.012 ± 0.003
MDA (mmol/L)	7.92 ± 1.12	**25.23 ± 2.84 ^a^**	**21.54 ± 3.12 ^a^**	**26.75 ± 3.44 ^a^**	**20.72 ± 3.27 ^a^**	**9.21 ± 2.11 ^b^**
PIGF (pg/mL)	450.2 ± 21.33	**155.6 ± 14.77 ^a^**	**427.1 ± 22.14 ^b^**	**166.8 ± 16.12 ^a^**	**171.3 ± 24.47 ^a^**	**437.1 ± 42.11 ^b^**
sFlt-1 (pg/mL)	932.7 ± 142.21	**3254.6 ± 135.22 ^a^**	**1052.3 ± 444.54 ^b^**	**1024.5 ± 155.42 ^a^**	**989.7 ± 141.13 ^a^**	**3524.4 ± 412.25 ^b^**

Statistical analyses were performed using one–way ANOVA followed by Bonferroni’s post hoc test. Values are expressed as mean ± SEM (standard error of the mean). n = 10 rats per group. ^a^ *p* ≤ 0.05 when compared to naïve group. ^b^ *p* ≤ 0.05 when compared to negative control (NC) group. ALT: alanine aminotransferase; AST: aspartate aminotransferase; CK-MB: creatine kinase-MB; NT: nitrotyrosine; MDA: malondialdehyde; PIGF: placental growth factor; sFlt-1: soluble fms-like tyrosine kinase-1.

**Table 6:** Mesenteric vascular bed reactivity of pregnant Wistar–Kyoto (naïve) and SHRs treated with different doses of AuNCs-PP or amlodipine on the 18th day of gestation.

Parameter	Naïve	NC	Amlodipine	AuNCs-PP 0.03 mg/kg	AuNCs-PP 0.1 mg/kg	AuNCs-PP 0.3 mg/kg
Phe (nmol)						
1	0.61 ± 0.19	0.69 ± 0.17	0.63 ± 0.18	0.67 ± 0.17	0.69 ± 0.15	0.77 ± 0.18
3	2.56 ± 0.76	2.61 ± 0.65	2.76 ± 0.77	2.58 ± 0.85	2.64 ± 0.84	2.78 ± 0.75
10	7.77 ± 1.28	7.99 ± 1.22	7.29 ± 1.17	7.28 ± 1.48	7.33 ± 1.55	7.12 ± 1.44
30	10.76 ± 2.77	**20.62 ± 3.13 ^a^**	**13.80 ± 2.09 ^b^**	**21.17 ± 2.50 ^a^**	**19.49 ± 2.09 ^a^**	**9.76 ± 1.99 ^b^**
ACh (pmol)						
1	−0.87 ± 0.21	−0.78 ± 0.27	−0.77 ± 0.23	−0.66 ± 0.21	−0.70 ± 0.21	−0.69 ± 0.26
3	−3.20 ± 0.51	−3.43 ± 0.73	−3.14 ± 0.55	−3.36 ± 0.81	−3.25 ± 0.76	−3.53 ± 0.41
10	−10.69 ± 2.47	**−3.76 ± 0.83 ^a^**	**−11.50 ± 2.33 ^b^**	**−3.52 ± 0.73 ^a^**	**−3.69 ± 0.80 ^a^**	**−11.21 ± 1.87 ^b^**
30	−20.50 ± 3.87	**−6.74 ± 1.14 ^a^**	**−21.38 ± 3.90 ^b^**	**−6.13 ± 1.66 ^a^**	**−7.05 ± 1.56 ^a^**	**−22.87 ± 3.15 ^b^**
SNP (nmol)						
1	−2.30 ± 0.68	−3.02 ± 0.85	−2.96 ± 0.95	−2.42 ± 0.86	−2.59 ± 0.79	−2.84 ± 0.77
3	−7.32 ± 1.24	−7.12 ± 1.29	−7.56 ± 1.57	−8.54 ± 1.47	−7.74 ± 1.74	−7.03 ± 1.54
10	−14.73 ± 2.28	−13.45 ± 2.35	−13.88 ± 2.97	−14.26 ± 2.56	−15.09 ± 2.06	−13.06 ± 2.27
30	−25.10 ± 3.61	−21.98 ± 3.74	−21.98 ± 3.60	−22.62 ± 3.89	−23.07 ± 3.40	−23.02 ± 3.99

Statistical analyses were performed using one–way ANOVA followed by Bonferroni’s post hoc test. Values are expressed as mean ± SEM (standard error of the mean). n = 10 rats per group. ^a^ *p* ≤ 0.05 when compared to naïve group. ^b^ *p* ≤ 0.05 when compared to negative control (NC) group. ACh: acetylcholine; SNP: sodium nitroprusside; Phe: phenylephrine.

**Table 7:** Reproductive parameters of pregnant Wistar–Kyoto (naïve) and SHRs treated with different doses of AuNCs-PP or amlodipine on the 18th day of gestation.

Parameter	Naïve	NC	Amlodipine	AuNCs-PP 0.03 mg/kg	AuNCs-PP 0.1 mg/kg	AuNCs-PP 0.3 mg/kg
Gestational BW gain (g)	35.00 ± 2.61	38.00 ± 4.20	31.83 ± 3.92	35.57 ± 3.52	33.50 ± 3.83	36.29 ± 4.84
Organogenic period BW gain (g)	13.00 ± 2.43	15.43 ± 3.25	15.27 ± 3.33	14.18 ± 3.51	12.54 ± 3.59	13.14 ± 2.98
Offspring-to-mother ratio	3.55 ± 0.37	**1.25 ± 0.36 ^a^**	**1.50 ± 0.32 ^a^**	**1.37 ± 0.43 ^a^**	**1.25 ± 0.39 ^a^**	**2.87 ± 0.48 ^b^**
Ovary weight (mg/100 g)	71.16 ± 4.48	77.01 ± 5.12	76.25 ± 4.85	73.92 ± 5.69	72.54 ± 4.94	70.55 ± 4.57
Mean fetal weight (g)	0.67 ± 0.17	**0.37 ± 0.03 ^a^**	**0.47 ± 0.18 ^b^**	**0.36 ± 0.07 ^a^**	**0.38 ± 0.06 ^a^**	**0.45 ± 0.15 ^b^**
Placental weight (g)	0.46 ± 0.08	**0.23 ± 0.02 ^a^**	**0.22 ± 0.05 ^a^**	**0.26 ± 0.03 ^a^**	**0.25 ± 0.04 ^a^**	**0.26 ± 0.02 ^a^**
Number of corpora lutea	17.25 ± 1.25	16.10 ± 1.03	17.13 ± 1.87	16.13 ± 1.95	16.14 ± 1.45	16.25 ± 1.22
Implantation index (%)	53.74 ± 6.14	**36.54 ± 4.90 ^a^**	**45.31 ± 6.95**	**34.75 ± 3.90 ^a^**	**33.24 ± 3.04 ^a^**	**54.98 ± 6.88 ^b^**
Resorption index (%)	25.50 ± 3.52	**14.83 ± 3.35 ^a^**	**32.81 ± 3.13 ^b^**	**15.78 ± 3.04 ^a^**	**16.28 ± 3.13 ^a^**	**30.96 ± 3.50 ^b^**
Pre-implantation loss (%)	56.26 ± 8.14	63.46 ± 6.90	54.69 ± 10.95	65.25 ± 6.90	66.76 ± 7.04	55.02 ± 6.88
Post-implantation loss (%)	62.77 ± 12.73	78.00 ± 10.25	79.17 ± 8.35	79.15 ± 8.38	81.46 ± 10.27	69.02 ± 10.39

Statistical analyses were performed using one–way ANOVA followed by Bonferroni’s post hoc test. Values are expressed as mean ± SEM (standard error of the mean). n = 10 rats per group. ^a^ *p* ≤ 0.05 when compared to naïve group. ^b^ *p* ≤ 0.05 when compared to negative control (NC) group. BW: body weight. Offspring-to-mother ratio = total litter weight (g)/maternal body weight (g) × 100. Mean fetal weight (g) = total weight of all live fetuses/number of live fetuses per litter. Total corpora lutea (CL) = right ovary CL + left ovary CL. Implantation index (%) = number of implantation sites/number of corpora lutea × 100. Resorption index (%) = number of resorptions/numbers of implantation sites × 100. Pre-implantation loss (%) = (number of corpora lutea − number of implantation sites)/number of corpora lutea × 100. Post-implantation loss (%) = (number of implantation sites − number of live fetuses)/number of implantation sites × 100.

**Table 8:** Relative organ weights of pregnant Wistar–Kyoto (naïve) and SHRs treated with different doses of AuNCs-PP or amlodipine on the 18th day of gestation.

Parameter	Naïve	NC	Amlodipine	AuNCs-PP 0.03 mg/kg	AuNCs-PP 0.1 mg/kg	AuNCs-PP 0.3 mg/kg
Liver (%)	4.27 ± 0.14	**5.25 ± 0.35 ^a^**	**5.24 ± 0.31 ^a^**	**5.28 ± 0.13 ^a^**	**5.28 ± 0.29 ^a^**	**5.20 ± 0.24 ^a^**
Right kidney (%)	0.36 ± 0.01	0.37 ± 0.01	0.37 ± 0.01	0.36 ± 0.01	0.37 ± 0.01	0.36 ± 0.02
Uterus (%)	0.97 ± 0.07	**0.62 ± 0.06 ^a^**	0.84 ± 0.08	**0.60 ± 0.05 ^a^**	**0.65 ± 0.05 ^a^**	0.82 ± 0.08
Heart (%)	0.29 ± 0.03	**0.45 ± 0.02 ^a^**	**0.47 ± 0.02 ^a^**	**0.46 ± 0.02 ^a^**	**0.45 ± 0.02 ^a^**	**0.47 ± 0.02 ^a^**

Statistical analyses were performed using one–way ANOVA followed by Bonferroni’s post hoc test. Values are expressed as mean ± SEM (standard error of the mean). n = 10 rats per group. ^a^ *p* ≤ 0.05 when compared to naïve group. Relative organ weight = absolute organ weight (g)/total body weight (g) × 100.

## Data Availability

The original contributions presented in this study are included in the article/Supplementary Materials. Further inquiries can be directed to the corresponding author.

## Supplementary Materials

The following supporting information can be downloaded at https://www.mdpi.com/article/10.3390/pharmaceutics18091074/s1.
