# Supplementary material for: Green-Synthesized Gold Nanoparticles Using Pfaffia glomerata Extract Improve Maternal Hypertension and Angiogenic Markers in Pregnant Hypertensive Rats
Source: Pharmaceutics. 2026 Aug 27;18(9):1074. doi: 10.3390/pharmaceutics18091074 (PMC13610012; doi:10.3390/pharmaceutics18091074)
Supplement: Supplementary file 1 [file pharmaceutics-18-01074-s001.zip › pharmaceutics-4406431-supplementary.pdf]

Compound: Morina

Curve Fit: Linear | Weighting: None | Zero: Not Forced
Quantitative Method: External Standard
Q 302,80>152,90
R^2 = 0.9701964 R = 0.9849855
y = 686,5927x + 16275,07

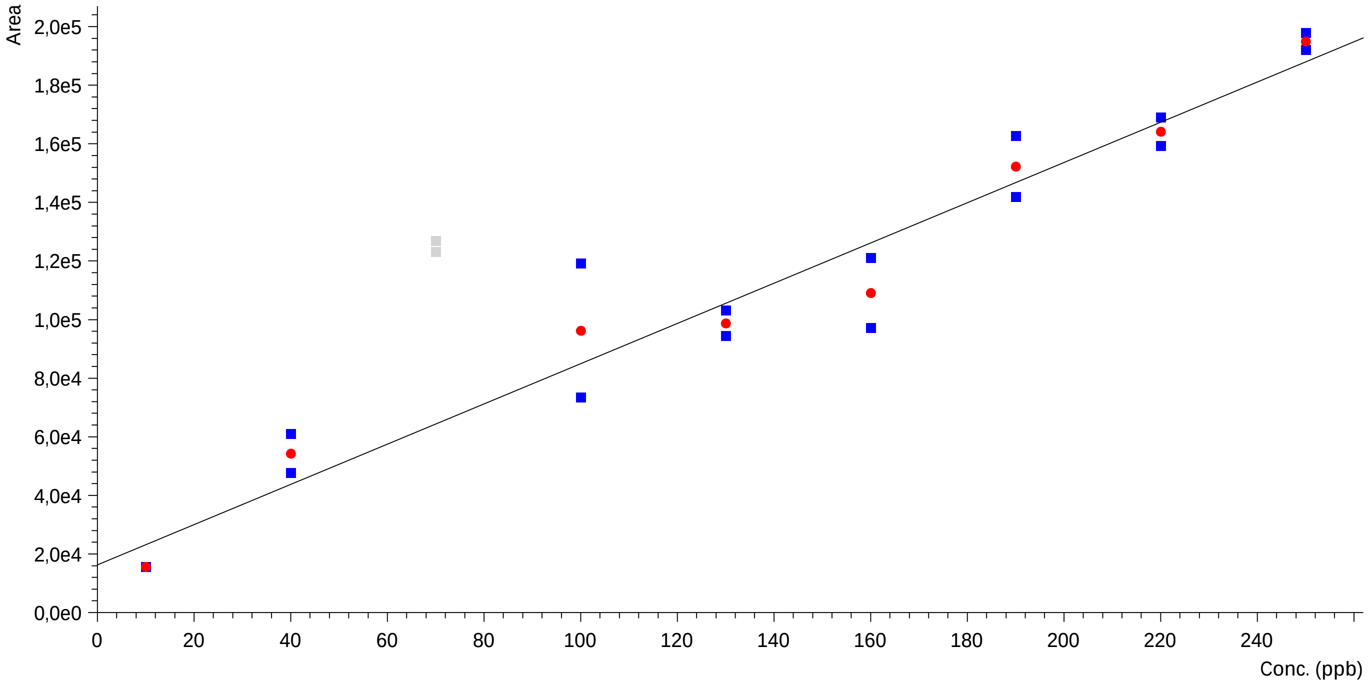

Sample ID	Area	Conc.	Sample Type	Level	Cal Point	Found RT	Ref 1 Actual Ratio
0	----	----	Ctrl.	0	----	----	----
10ppb001	----	----	Std.	1	No	----	----
10 ppb002	15617	-0,9577	Std.	1	Yes	9,743	29,32
40 ppb001	47578	45,5916	Std.	2	Yes	9,708	60,95
40ppb002	60980	65,1116	Std.	2	Yes	9,701	58,95
70 ppb001	123150	155,6599	Std.	3	No	9,735	49,86
70ppb002	126925	161,1578	Std.	3	No	9,732	64,90
100 ppb001	119151	149,8354	Std.	4	Yes	9,719	57,52
100ppb002	73373	83,1620	Std.	4	Yes	9,713	91,60
130 ppb001	103076	126,4226	Std.	5	Yes	9,717	53,67
130ppb002	94509	113,9448	Std.	5	Yes	9,715	94,57
160 ppb001	121045	152,5947	Std.	6	Yes	9,718	59,12
160ppb002	97110	117,7341	Std.	6	Yes	9,718	55,03
190 ppb001	162655	213,1972	Std.	7	Yes	9,730	53,27
190ppb002	141866	182,9188	Std.	7	Yes	9,708	60,81
220 ppb001	169026	222,4763	Std.	8	Yes	9,715	63,55
220ppb002	159259	208,2508	Std.	8	Yes	9,715	55,82
250 ppb001	197889	264,5149	Std.	9	Yes	9,722	70,18
250 ppb002	192153	256,1608	Std.	9	Yes	9,709	67,11
10H	----	----	Unk.	----	----	----	----
10M	----	----	Unk.	----	----	----	----
9H	----	----	Unk.	----	----	----	----
9M	----	----	Unk.	----	----	----	----
Average	117962	148,1044				9,719	61,54
%RSD	43	49,6367				0,111	24,12
Maximum	197889	264,5149				9,743	94,57
Minimum	15617	-0,9577				9,701	29,32
Std. Dev.	50474	73,5141				0,011	14,84

Compound: Morina (continued)

0
Conc ----
Area ----
R#1 302,80>228,95 ---- (67,11)
R#2 302,80>256,85 ---- (42,86)
Q 302,80>152,90 (+) 8,97e2

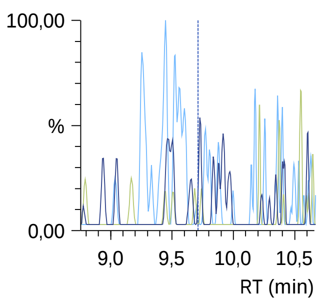

10ppb001
Conc ----
Area ----
R#1 302,80>228,95 ---- (67,11)
R#2 302,80>256,85 ---- (42,86)
Q 302,80>152,90 (+) 2,44e3

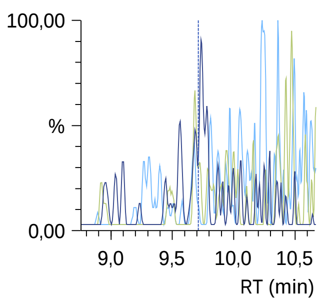

10 ppb002
Conc -0,9577
Area 15617
R#1 302,80>228,95 29,32 (67,11)
R#2 302,80>256,85 22,83 (42,86)
Q 302,80>152,90 (+) 5,00e3

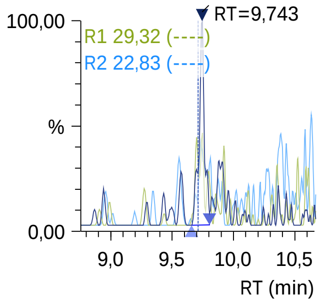

40 ppb001
Conc 45,5916
Area 47578
R#1 302,80>228,95 60,95 (67,11)
R#2 302,80>256,85 40,80 (42,86)
Q 302,80>152,90 (+) 1,00e4

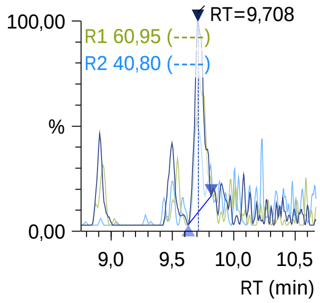

40ppb002
Conc 65,1116
Area 60980
R#1 302,80>228,95 58,95 (67,11)
R#2 302,80>256,85 21,36 (42,86)
Q 302,80>152,90 (+) 1,30e4

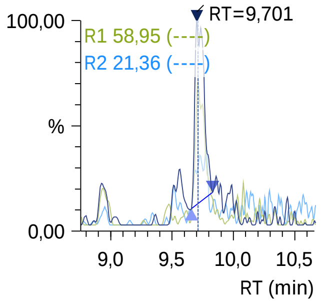

70 ppb001
Conc 155,6599
Area 123150
R#1 302,80>228,95 49,86 (67,11)
R#2 302,80>256,85 31,90 (42,86)
Q 302,80>152,90 (+) 2,84e4

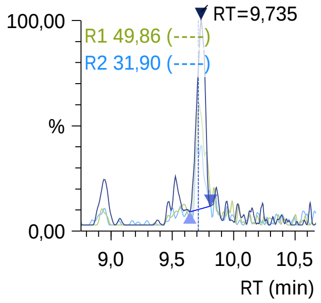

70ppb002
Conc 161,1578
Area 126925
R#1 302,80>228,95 64,90 (67,11)
R#2 302,80>256,85 22,34 (42,86)
Q 302,80>152,90 (+) 2,40e4

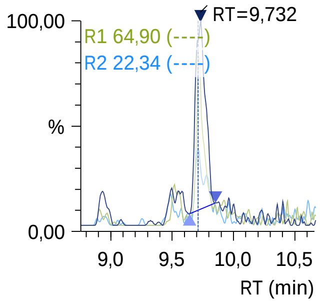

100 ppb001
Conc 149,8354
Area 119151
R#1 302,80>228,95 57,52 (67,11)
R#2 302,80>256,85 28,74 (42,86)
Q 302,80>152,90 (+) 2,89e4

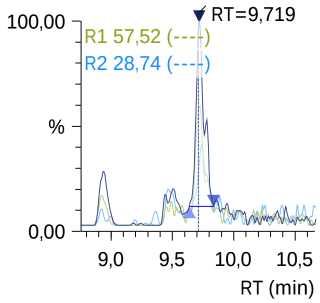

100ppb002
Conc 83,1620
Area 73373
R#1 302,80>228,95 91,60 (67,11)
R#2 302,80>256,85 20,58 (42,86)
Q 302,80>152,90 (+) 1,47e4

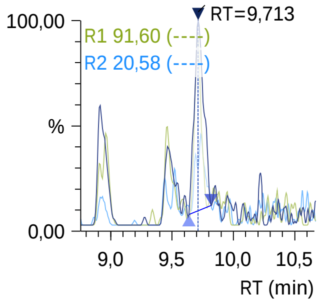

130 ppb001
Conc 126,4226
Area 103076
R#1 302,80>228,95 53,67 (67,11)
R#2 302,80>256,85 35,75 (42,86)
Q 302,80>152,90 (+) 2,24e4

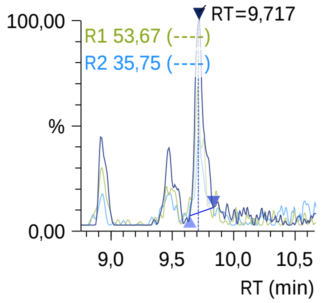

130ppb002
Conc 113,9448
Area 94509
R#1 302,80>228,95 94,57 (67,11)
R#2 302,80>256,85 37,24 (42,86)
Q 302,80>152,90 (+) 2,29e4

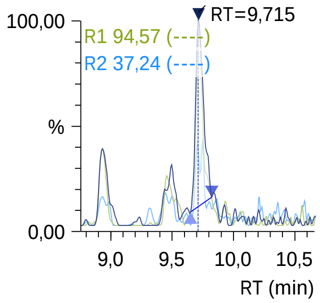

160 ppb001
Conc 152,5947
Area 121045
R#1 302,80>228,95 59,12 (67,11)
R#2 302,80>256,85 24,59 (42,86)
Q 302,80>152,90 (+) 3,01e4

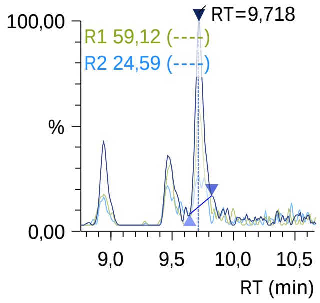

Compound: Morina (continued)

160ppb002
Conc 117,7341
Area 97110
R#1 302,80>228,95 55,03 (67,11)
R#2 302,80>256,85 26,70 (42,86)
Q 302,80>152,90 (+) 3,30e4

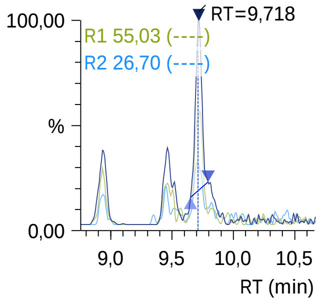

190 ppb001
Conc 213,1972
Area 162655
R#1 302,80>228,95 53,27 (67,11)
R#2 302,80>256,85 34,10 (42,86)
Q 302,80>152,90 (+) 3,37e4

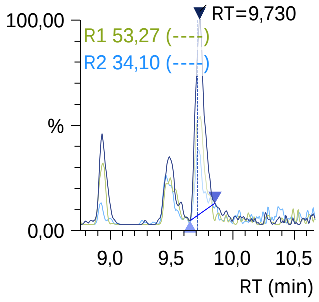

190ppb002
Conc 182,9188
Area 141866
R#1 302,80>228,95 60,81 (67,11)
R#2 302,80>256,85 31,79 (42,86)
Q 302,80>152,90 (+) 3,78e4

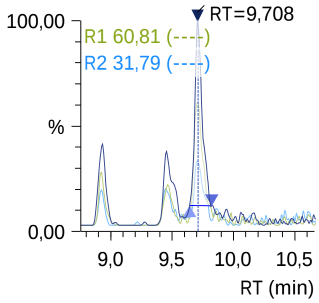

220 ppb001
Conc 222,4763
Area 169026
R#1 302,80>228,95 63,55 (67,11)
R#2 302,80>256,85 23,83 (42,86)
Q 302,80>152,90 (+) 3,98e4

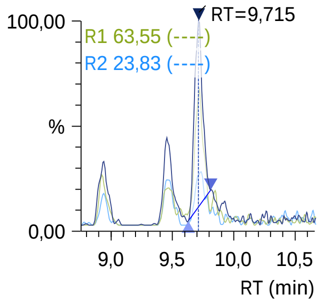

220ppb002
Conc 208,2508
Area 159259
R#1 302,80>228,95 55,82 (67,11)
R#2 302,80>256,85 23,33 (42,86)
Q 302,80>152,90 (+) 3,91e4

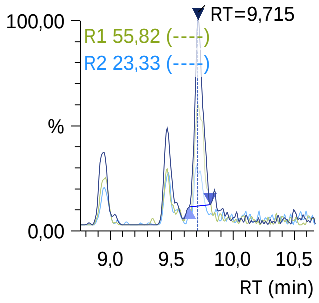

250 ppb001
Conc 264,5149
Area 197889
R#1 302,80>228,95 70,18 (67,11)
R#2 302,80>256,85 27,62 (42,86)
Q 302,80>152,90 (+) 4,58e4

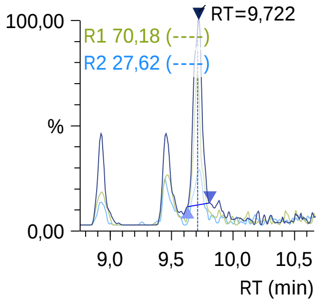

250 ppb002
Conc 256,1608
Area 192153
R#1 302,80>228,95 67,11 (67,11)
R#2 302,80>256,85 42,86 (42,86)
Q 302,80>152,90 (+) 4,33e4

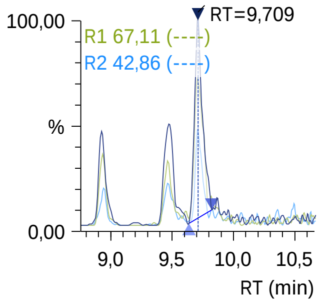

10H
Conc ----
Area ----
R#1 302,80>228,95 ---- (67,11)
R#2 302,80>256,85 ---- (42,86)
Q 302,80>152,90 (+) 3,39e3

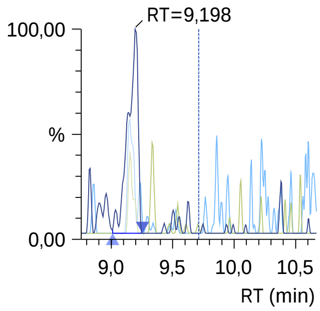

10M
Conc ----
Area ----
R#1 302,80>228,95 ---- (67,11)
R#2 302,80>256,85 ---- (42,86)
Q 302,80>152,90 (+) 2,78e3

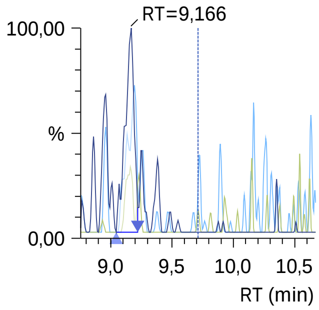

9H
Conc ----
Area ----
R#1 302,80>228,95 ---- (67,11)
R#2 302,80>256,85 ---- (42,86)
Q 302,80>152,90 (+) 8,11e3

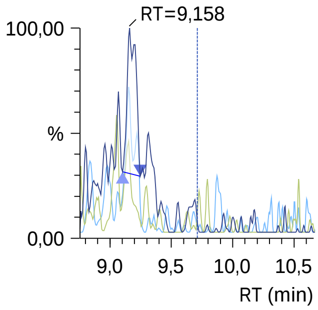

9M
Conc ----
Area ----
R#1 302,80>228,95 ---- (67,11)
R#2 302,80>256,85 ---- (42,86)
Q 302,80>152,90 (+) 1,20e4

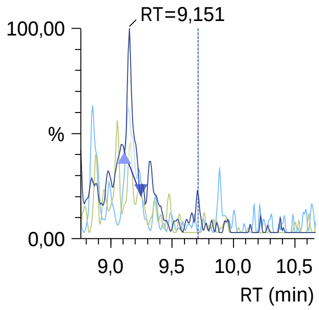

Compound: Isovanilina

Curve Fit: Linear | Weighting: None | Zero: Not Forced
Quantitative Method: External Standard
Q 153,10>65,00
R^2 = 0.9961571 R = 0.9980767
y = 9407,413x + 957820,8

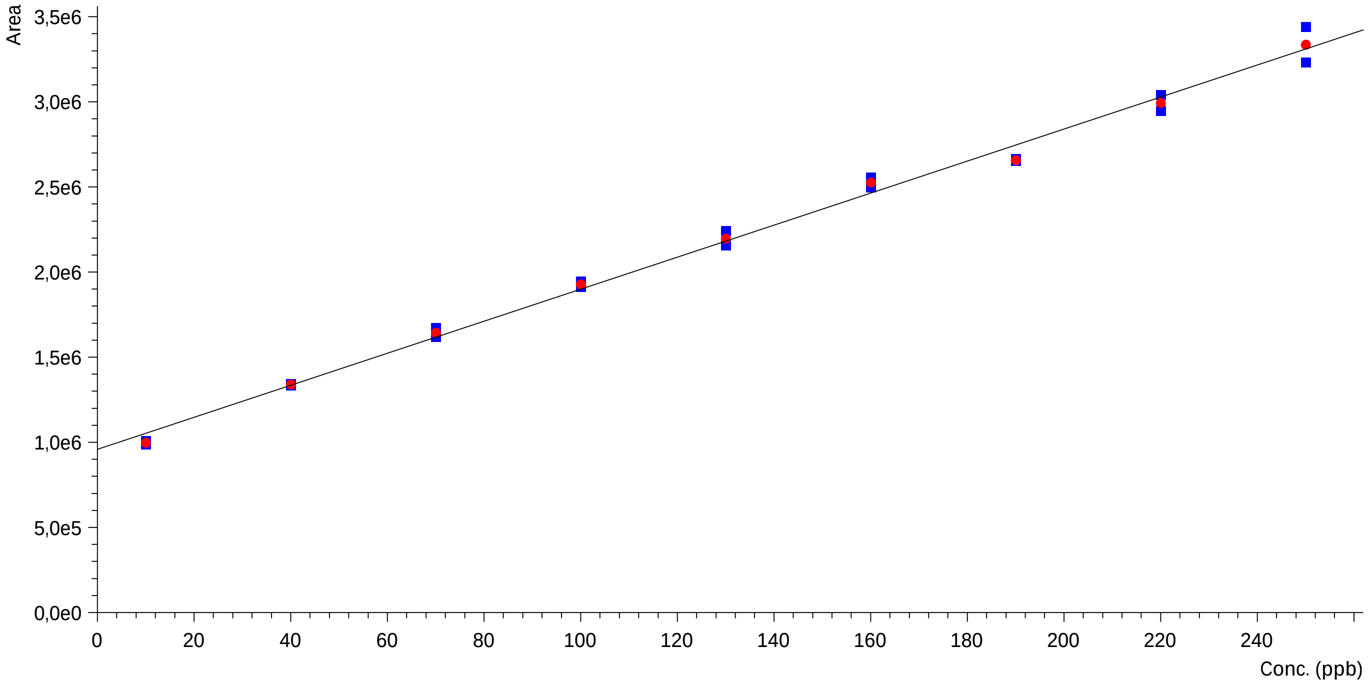

Sample ID	Area	Conc.	Sample Type	Level	Cal Point	Found RT	Ref 1 Actual Ratio
0	555294	-42,7883	Ctrl.	0	----	8,787	44,05
10ppb001	1010320	5,5807	Std.	1	Yes	8,713	42,75
10 ppb002	987926	3,2001	Std.	1	Yes	8,732	49,68
40 ppb001	1335539	40,1511	Std.	2	Yes	8,709	47,36
40ppb002	1344184	41,0701	Std.	2	Yes	8,629	43,42
70 ppb001	1619687	70,3558	Std.	3	Yes	8,633	47,88
70ppb002	1671586	75,8727	Std.	3	Yes	8,632	42,67
100 ppb001	1946075	105,0506	Std.	4	Yes	8,615	53,07
100ppb002	1911528	101,3782	Std.	4	Yes	8,624	50,77
130 ppb001	2240311	136,3276	Std.	5	Yes	8,616	51,37
130ppb002	2156414	127,4094	Std.	5	Yes	8,625	52,48
160 ppb001	2556008	169,8860	Std.	6	Yes	8,624	52,57
160ppb002	2498346	163,7565	Std.	6	Yes	8,621	51,00
190 ppb001	2652976	180,1936	Std.	7	Yes	8,625	54,31
190ppb002	2663209	181,2813	Std.	7	Yes	8,617	51,61
220 ppb001	3041354	221,4778	Std.	8	Yes	8,619	49,81
220ppb002	2946425	211,3869	Std.	8	Yes	8,624	52,55
250 ppb001	3439793	263,8315	Std.	9	Yes	8,615	54,94
250 ppb002	3232440	241,7901	Std.	9	Yes	8,617	54,58
10H	642679	-33,4993	Unk.	----	----	8,761	49,40
10M	577189	-40,4609	Unk.	----	----	8,751	49,65
9H	473912	-51,4391	Unk.	----	----	8,738	53,55
9M	489874	-49,7424	Unk.	----	----	8,745	52,33
Average	1825786	92,2639				8,664	50,08
%RSD	52	110,0749				0,695	7,54
Maximum	3439793	263,8315				8,787	54,94
Minimum	473912	-51,4391				8,615	42,67
Std. Dev.	955411	101,5594				0,060	3,78

Compound: Isovanilina (continued)

0
Conc -42,7883
Area 555294
R#1 153,10>92,95 44,05 (50,10)
R#2 153,10>125,00 12,71 (21,72)
Q 153,10>65,00 (+) 7,17e4

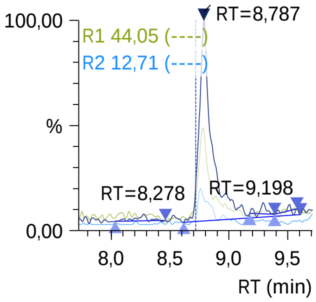

10ppb001
Conc 5,5807
Area 1010320
R#1 153,10>92,95 42,75 (50,10)
R#2 153,10>125,00 16,50 (21,72)
Q 153,10>65,00 (+) 9,16e4

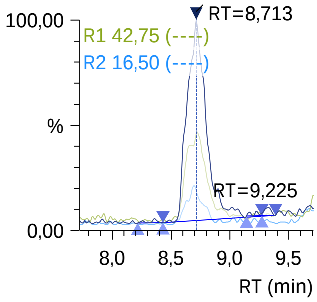

10 ppb002
Conc 3,2001
Area 987926
R#1 153,10>92,95 49,68 (50,10)
R#2 153,10>125,00 18,24 (21,72)
Q 153,10>65,00 (+) 8,19e4

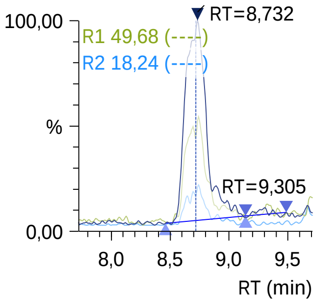

40 ppb001
Conc 40,1511
Area 1335539
R#1 153,10>92,95 47,36 (50,10)
R#2 153,10>125,00 14,90 (21,72)
Q 153,10>65,00 (+) 1,12e5

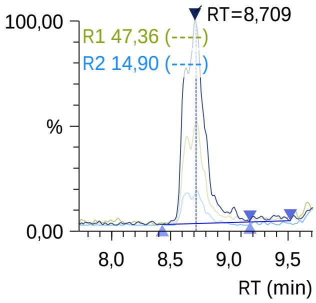

40ppb002
Conc 41,0701
Area 1344184
R#1 153,10>92,95 43,42 (50,10)
R#2 153,10>125,00 15,45 (21,72)
Q 153,10>65,00 (+) 1,21e5

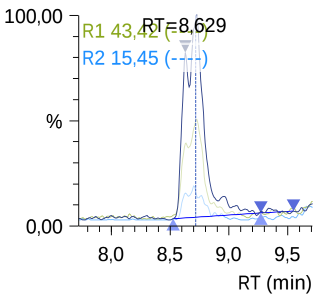

70 ppb001
Conc 70,3558
Area 1619687
R#1 153,10>92,95 47,88 (50,10)
R#2 153,10>125,00 18,80 (21,72)
Q 153,10>65,00 (+) 1,51e5

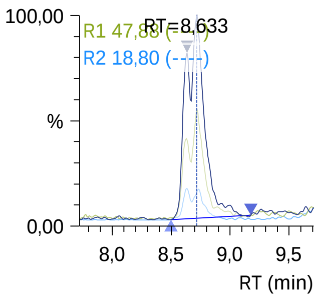

70ppb002
Conc 75,8727
Area 1671586
R#1 153,10>92,95 42,67 (50,10)
R#2 153,10>125,00 15,37 (21,72)
Q 153,10>65,00 (+) 1,47e5

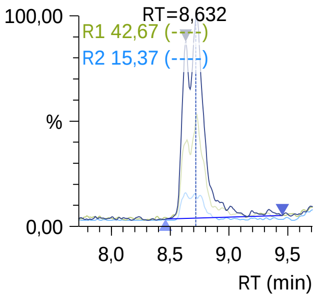

100 ppb001
Conc 105,0506
Area 1946075
R#1 153,10>92,95 53,07 (50,10)
R#2 153,10>125,00 16,79 (21,72)
Q 153,10>65,00 (+) 1,76e5

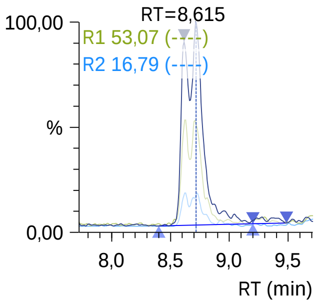

100ppb002
Conc 101,3782
Area 1911528
R#1 153,10>92,95 50,77 (50,10)
R#2 153,10>125,00 21,42 (21,72)
Q 153,10>65,00 (+) 1,87e5

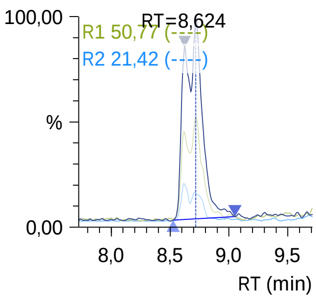

130 ppb001
Conc 136,3276
Area 2240311
R#1 153,10>92,95 51,37 (50,10)
R#2 153,10>125,00 16,75 (21,72)
Q 153,10>65,00 (+) 2,13e5

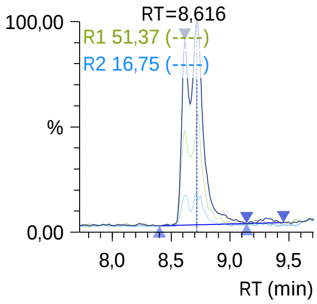

130ppb002
Conc 127,4094
Area 2156414
R#1 153,10>92,95 52,48 (50,10)
R#2 153,10>125,00 23,28 (21,72)
Q 153,10>65,00 (+) 2,18e5

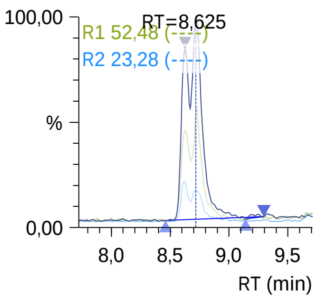

160 ppb001
Conc 169,8860
Area 2556008
R#1 153,10>92,95 52,57 (50,10)
R#2 153,10>125,00 21,44 (21,72)
Q 153,10>65,00 (+) 2,55e5

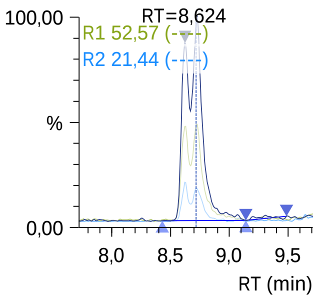

Compound: Isovanilina (continued)

160ppb002
Conc 163,7565
Area 2498346
R#1 153,10>92,95 51,00 (50,10)
R#2 153,10>125,00 16,95 (21,72)
Q 153,10>65,00 (+) 2,38e5

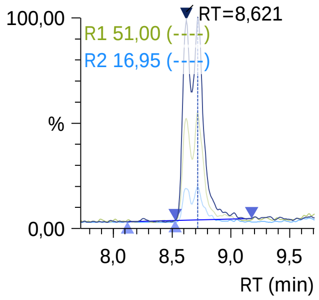

190 ppb001
Conc 180,1936
Area 2652976
R#1 153,10>92,95 54,31 (50,10)
R#2 153,10>125,00 20,05 (21,72)
Q 153,10>65,00 (+) 2,71e5

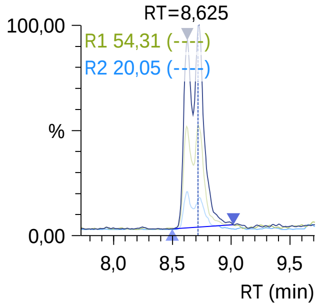

190ppb002
Conc 181,2813
Area 2663209
R#1 153,10>92,95 51,61 (50,10)
R#2 153,10>125,00 19,58 (21,72)
Q 153,10>65,00 (+) 2,74e5

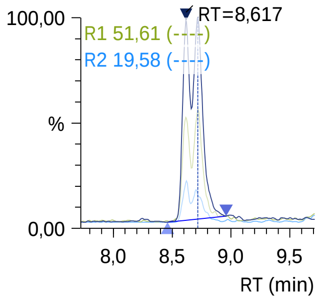

220 ppb001
Conc 221,4778
Area 3041354
R#1 153,10>92,95 49,81 (50,10)
R#2 153,10>125,00 17,34 (21,72)
Q 153,10>65,00 (+) 3,16e5

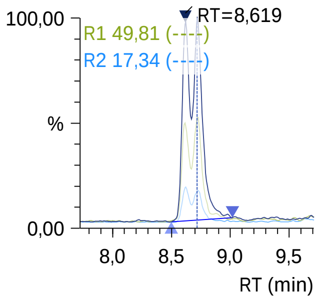

220ppb002
Conc 211,3869
Area 2946425
R#1 153,10>92,95 52,55 (50,10)
R#2 153,10>125,00 15,34 (21,72)
Q 153,10>65,00 (+) 3,11e5

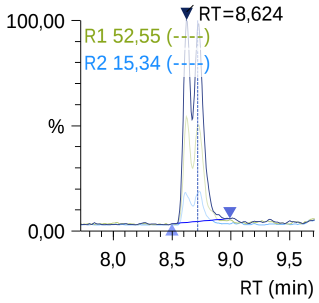

250 ppb001
Conc 263,8315
Area 3439793
R#1 153,10>92,95 54,94 (50,10)
R#2 153,10>125,00 19,76 (21,72)
Q 153,10>65,00 (+) 3,59e5

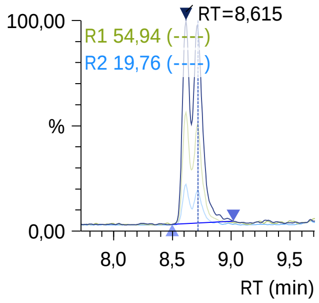

250 ppb002
Conc 241,7901
Area 3232440
R#1 153,10>92,95 54,58 (50,10)
R#2 153,10>125,00 17,90 (21,72)
Q 153,10>65,00 (+) 3,51e5

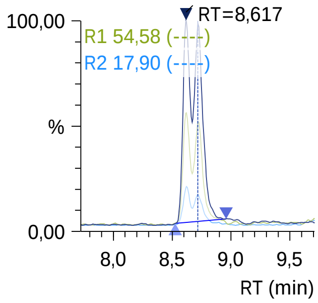

10H
Conc -33,4993
Area 642679
R#1 153,10>92,95 49,40 (50,10)
R#2 153,10>125,00 15,08 (21,72)
Q 153,10>65,00 (+) 6,22e4

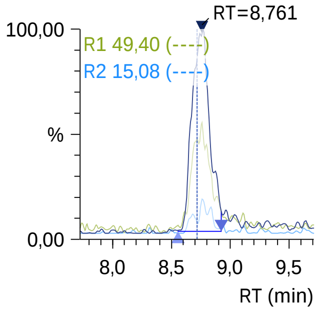

10M
Conc -40,4609
Area 577189
R#1 153,10>92,95 49,65 (50,10)
R#2 153,10>125,00 16,72 (21,72)
Q 153,10>65,00 (+) 6,77e4

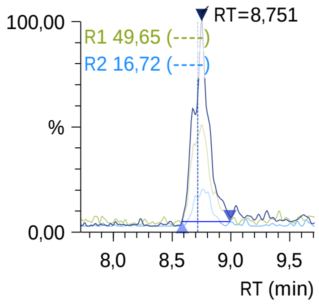

9H
Conc -51,4391
Area 473912
R#1 153,10>92,95 53,55 (50,10)
R#2 153,10>125,00 16,37 (21,72)
Q 153,10>65,00 (+) 4,85e4

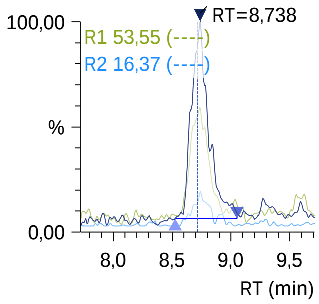

9M
Conc -49,7424
Area 489874
R#1 153,10>92,95 52,33 (50,10)
R#2 153,10>125,00 16,40 (21,72)
Q 153,10>65,00 (+) 4,16e4

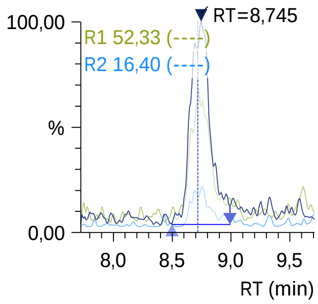

Compound: Hidroxibenzaldeido

Curve Fit: Linear | Weighting: None | Zero: Not Forced
Quantitative Method: External Standard
Q 123,10>76,95
R^2 = 0.9895631 R = 0.9947679
y = 2467,528x - 12200,66

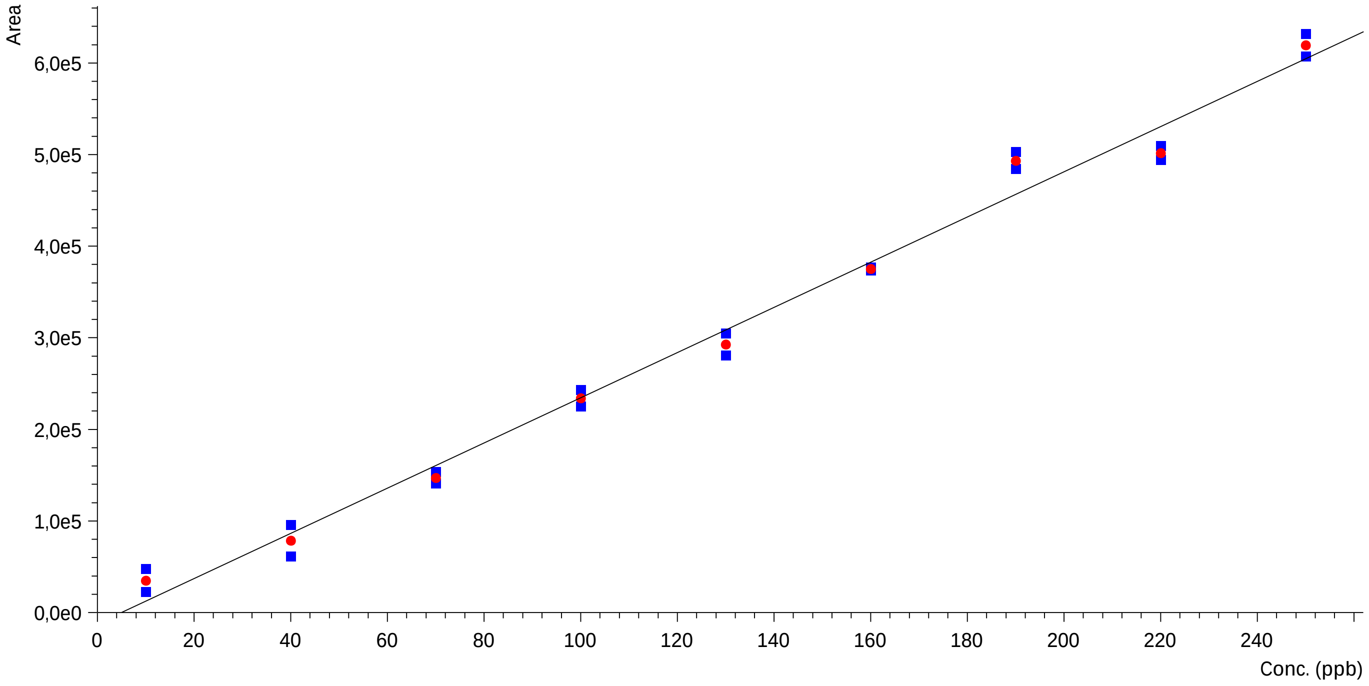

Sample ID	Area	Conc.	Sample Type	Level	Cal Point	Found RT	Ref 1 Actual Ratio
0	----	----	Ctrl.	0	----	----	----
10ppb001	47856	24,3387	Std.	1	Yes	8,548	48,21
10 ppb002	22213	13,9467	Std.	1	Yes	8,567	35,13
40 ppb001	61026	29,6761	Std.	2	Yes	8,547	65,89
40ppb002	95886	43,8035	Std.	2	Yes	8,551	63,24
70 ppb001	140708	61,9683	Std.	3	Yes	8,562	57,81
70ppb002	153240	67,0469	Std.	3	Yes	8,568	59,86
100 ppb001	224851	96,0683	Std.	4	Yes	8,553	61,44
100ppb002	243101	103,4645	Std.	4	Yes	8,557	61,75
130 ppb001	280644	118,6794	Std.	5	Yes	8,552	67,54
130ppb002	305194	128,6285	Std.	5	Yes	8,558	64,89
160 ppb001	373527	156,3213	Std.	6	Yes	8,564	54,27
160ppb002	376985	157,7227	Std.	6	Yes	8,558	69,25
190 ppb001	502737	208,6855	Std.	7	Yes	8,565	67,16
190ppb002	484176	201,1634	Std.	7	Yes	8,555	63,73
220 ppb001	493898	205,1034	Std.	8	Yes	8,558	65,49
220ppb002	509533	211,4396	Std.	8	Yes	8,561	67,62
250 ppb001	631634	260,9231	Std.	9	Yes	8,553	60,72
250 ppb002	607198	251,0200	Std.	9	Yes	8,558	62,70
10H	----	----	Unk.	----	----	----	----
10M	----	----	Unk.	----	----	----	----
9H	----	----	Unk.	----	----	----	----
9M	----	----	Unk.	----	----	----	----
Average	308578	130,0000				8,557	60,93
%RSD	64	61,7307				0,073	13,54
Maximum	631634	260,9231				8,568	69,25
Minimum	22213	13,9467				8,547	35,13
Std. Dev.	198019	80,2500				0,006	8,25

Compound: Hidroxibenzaldeido (continued)

0
Conc ----
Area ----
R#1 123,10>94,95 ---- (60,72)
R#2 123,10>50,95 ---- (43,60)
Q 123,10>76,95 (+) 6,04e3

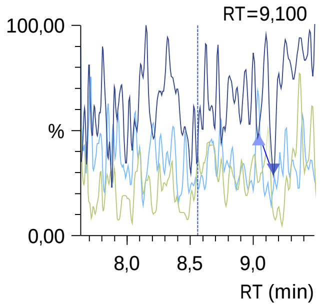

10ppb001
Conc 24,3387
Area 47856
R#1 123,10>94,95 48,21 (60,72)
R#2 123,10>50,95 59,44 (43,60)
Q 123,10>76,95 (+) 1,21e4

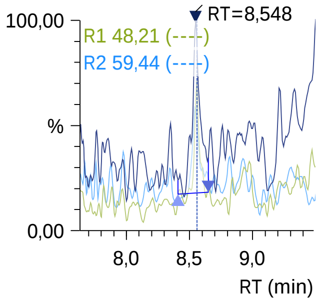

10 ppb002
Conc 13,9467
Area 22213
R#1 123,10>94,95 35,13 (60,72)
R#2 123,10>50,95 27,43 (43,60)
Q 123,10>76,95 (+) 1,22e4

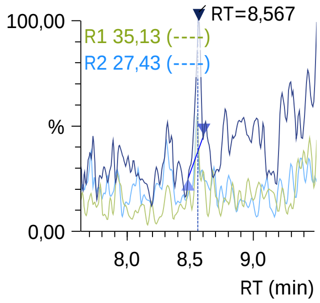

40 ppb001
Conc 29,6761
Area 61026
R#1 123,10>94,95 65,89 (60,72)
R#2 123,10>50,95 58,17 (43,60)
Q 123,10>76,95 (+) 2,08e4

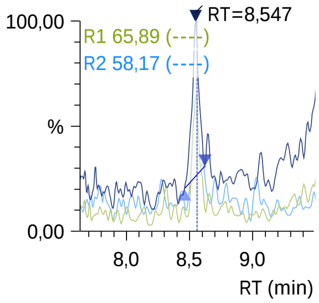

40ppb002
Conc 43,8035
Area 95886
R#1 123,10>94,95 63,24 (60,72)
R#2 123,10>50,95 52,24 (43,60)
Q 123,10>76,95 (+) 1,88e4

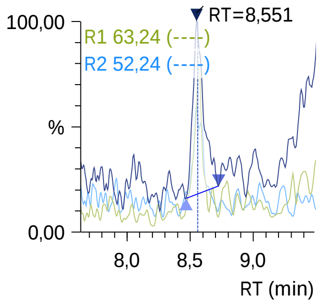

70 ppb001
Conc 61,9683
Area 140708
R#1 123,10>94,95 57,81 (60,72)
R#2 123,10>50,95 38,12 (43,60)
Q 123,10>76,95 (+) 4,28e4

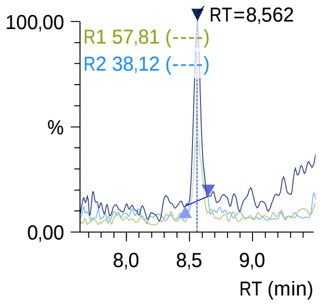

70ppb002
Conc 67,0469
Area 153240
R#1 123,10>94,95 59,86 (60,72)
R#2 123,10>50,95 54,03 (43,60)
Q 123,10>76,95 (+) 4,03e4

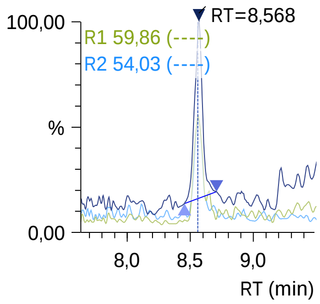

100 ppb001
Conc 96,0683
Area 224851
R#1 123,10>94,95 61,44 (60,72)
R#2 123,10>50,95 53,11 (43,60)
Q 123,10>76,95 (+) 5,71e4

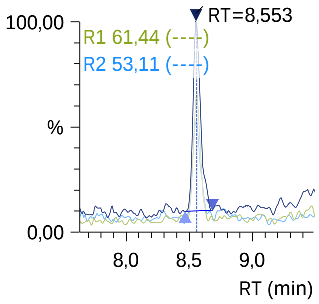

100ppb002
Conc 103,4645
Area 243101
R#1 123,10>94,95 61,75 (60,72)
R#2 123,10>50,95 49,20 (43,60)
Q 123,10>76,95 (+) 6,09e4

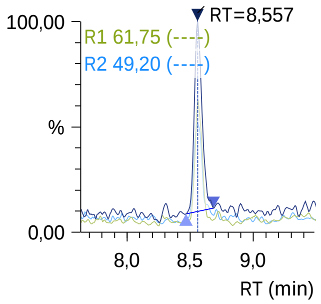

130 ppb001
Conc 118,6794
Area 280644
R#1 123,10>94,95 67,54 (60,72)
R#2 123,10>50,95 61,95 (43,60)
Q 123,10>76,95 (+) 6,62e4

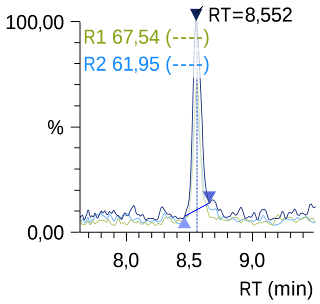

130ppb002
Conc 128,6285
Area 305194
R#1 123,10>94,95 64,89 (60,72)
R#2 123,10>50,95 55,97 (43,60)
Q 123,10>76,95 (+) 7,98e4

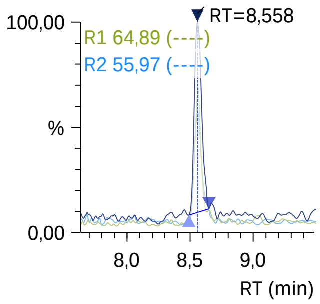

160 ppb001
Conc 156,3213
Area 373527
R#1 123,10>94,95 54,27 (60,72)
R#2 123,10>50,95 48,20 (43,60)
Q 123,10>76,95 (+) 1,01e5

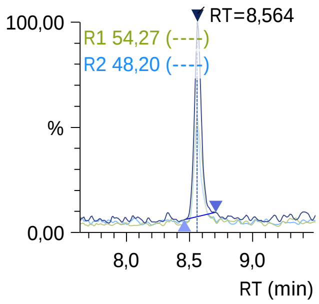

Compound: Hidroxibenzaldeido (continued)

160ppb002
Conc 157,7227
Area 376985
R#1 123,10>94,95 69,25 (60,72)
R#2 123,10>50,95 59,78 (43,60)
Q 123,10>76,95 (+) 8,64e4

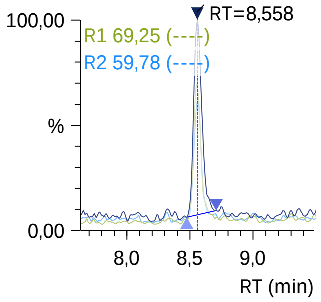

190 ppb001
Conc 208,6855
Area 502737
R#1 123,10>94,95 67,16 (60,72)
R#2 123,10>50,95 44,26 (43,60)
Q 123,10>76,95 (+) 1,24e5

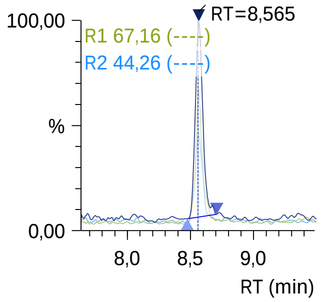

190ppb002
Conc 201,1634
Area 484176
R#1 123,10>94,95 63,73 (60,72)
R#2 123,10>50,95 49,14 (43,60)
Q 123,10>76,95 (+) 1,15e5

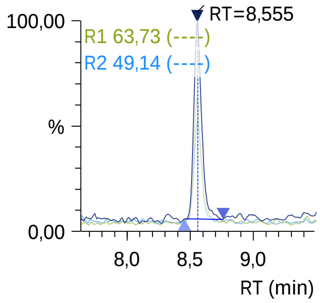

220 ppb001
Conc 205,1034
Area 493898
R#1 123,10>94,95 65,49 (60,72)
R#2 123,10>50,95 60,40 (43,60)
Q 123,10>76,95 (+) 1,17e5

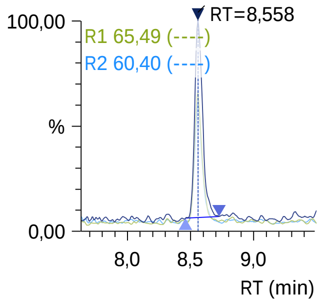

220ppb002
Conc 211,4396
Area 509533
R#1 123,10>94,95 67,62 (60,72)
R#2 123,10>50,95 54,22 (43,60)
Q 123,10>76,95 (+) 1,27e5

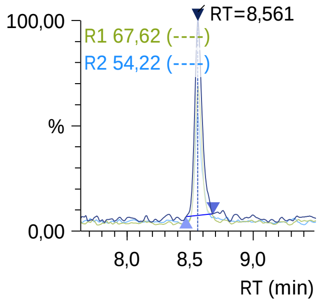

250 ppb001
Conc 260,9231
Area 631634
R#1 123,10>94,95 60,72 (60,72)
R#2 123,10>50,95 43,60 (43,60)
Q 123,10>76,95 (+) 1,55e5

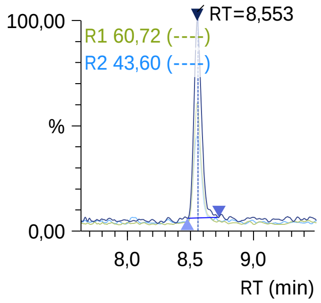

250 ppb002
Conc 251,0200
Area 607198
R#1 123,10>94,95 62,70 (60,72)
R#2 123,10>50,95 55,28 (43,60)
Q 123,10>76,95 (+) 1,52e5

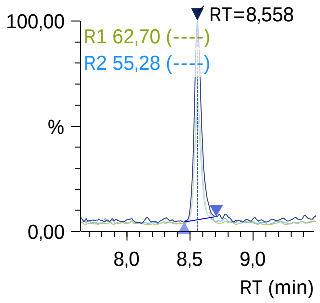

10H
Conc ----
Area ----
R#1 123,10>94,95 ---- (60,72)
R#2 123,10>50,95 ---- (43,60)
Q 123,10>76,95 (+) 3,86e3

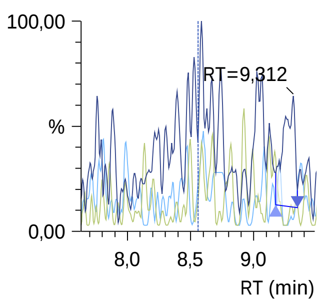

10M
Conc ----
Area ----
R#1 123,10>94,95 ---- (60,72)
R#2 123,10>50,95 ---- (43,60)
Q 123,10>76,95 (+) 4,34e3

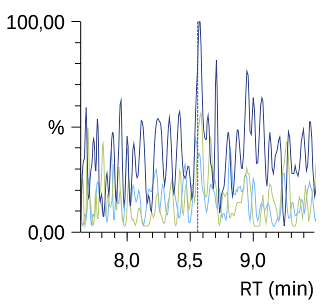

9H
Conc ----
Area ----
R#1 123,10>94,95 ---- (60,72)
R#2 123,10>50,95 ---- (43,60)
Q 123,10>76,95 (+) 4,67e3

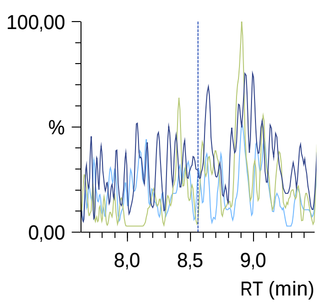

9M
Conc ----
Area ----
R#1 123,10>94,95 ---- (60,72)
R#2 123,10>50,95 ---- (43,60)
Q 123,10>76,95 (+) 6,93e3

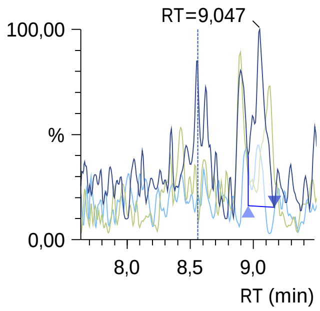

Compound: Naringenina

Curve Fit: Linear | Weighting: None | Zero: Not Forced
Quantitative Method: External Standard
Q 273,00>152,90
R^2 = 0.9919701 R = 0.9959770
y = 5547,662x + 43016,82

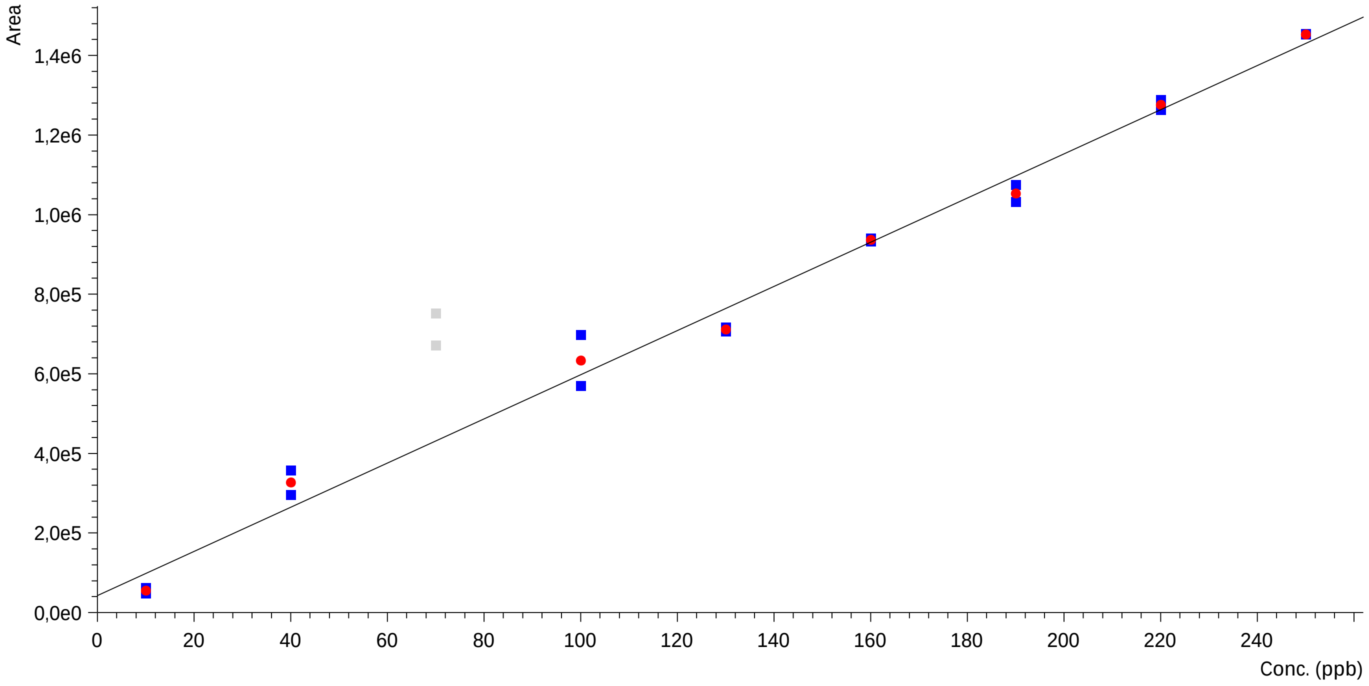

Sample ID	Area	Conc.	Sample Type	Level	Cal Point	Found RT	Ref 1 Actual Ratio
0	----	----	Ctrl.	0	----	----	----
10ppb001	48212	0,9365	Std.	1	Yes	9,761	59,96
10 ppb002	61986	3,4193	Std.	1	Yes	9,766	47,21
40 ppb001	296163	45,6312	Std.	2	Yes	9,752	44,08
40ppb002	358222	56,8177	Std.	2	Yes	9,758	46,87
70 ppb001	752013	127,8010	Std.	3	No	9,758	48,84
70ppb002	671395	113,2691	Std.	3	No	9,762	48,81
100 ppb001	698010	118,0666	Std.	4	Yes	9,759	49,07
100ppb002	569128	94,8347	Std.	4	Yes	9,754	48,30
130 ppb001	706380	119,5752	Std.	5	Yes	9,758	45,40
130ppb002	716442	121,3890	Std.	5	Yes	9,753	50,10
160 ppb001	933195	160,4601	Std.	6	Yes	9,757	49,14
160ppb002	940096	161,7041	Std.	6	Yes	9,752	47,99
190 ppb001	1032413	178,3447	Std.	7	Yes	9,761	52,30
190ppb002	1074824	185,9895	Std.	7	Yes	9,751	49,23
220 ppb001	1263571	220,0123	Std.	8	Yes	9,749	44,74
220ppb002	1288327	224,4748	Std.	8	Yes	9,758	43,66
250 ppb001	1452492	254,0665	Std.	9	Yes	9,750	49,57
250 ppb002	1453664	254,2778	Std.	9	Yes	9,755	49,93
10H	----	----	Unk.	----	----	----	----
10M	----	----	Unk.	----	----	----	----
9H	----	----	Unk.	----	----	----	----
9M	----	----	Unk.	----	----	----	----
Average	795363	135,6150				9,756	48,62
%RSD	54	57,0341				0,047	7,47
Maximum	1453664	254,2778				9,766	59,96
Minimum	48212	0,9365				9,749	43,66
Std. Dev.	429094	77,3468				0,005	3,63

Compound: Naringenina (continued)

0

Conc ----
Area ----
R#1 273,00>146,90 ---- (49,23)
R#2 273,00>118,95 ---- (26,26)
Q 273,00>152,90 (+) 2,25e3

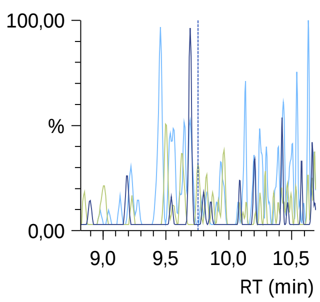

10ppb001

Conc 0,9365
Area 48212
R#1 273,00>146,90 59,96 (49,23)
R#2 273,00>118,95 42,48 (26,26)
Q 273,00>152,90 (+) 1,08e4

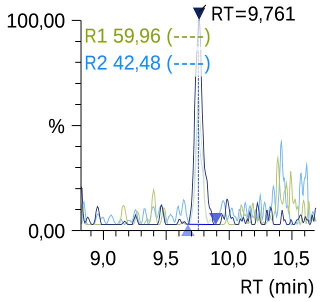

10 ppb002

Conc 3,4193
Area 61986
R#1 273,00>146,90 47,21 (49,23)
R#2 273,00>118,95 6,97 (26,26)
Q 273,00>152,90 (+) 1,39e4

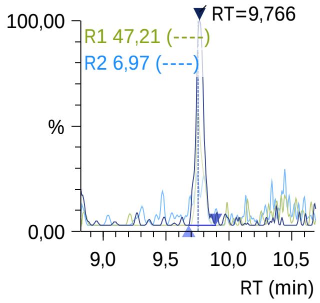

40 ppb001

Conc 45,6312
Area 296163
R#1 273,00>146,90 44,08 (49,23)
R#2 273,00>118,95 22,80 (26,26)
Q 273,00>152,90 (+) 7,42e4

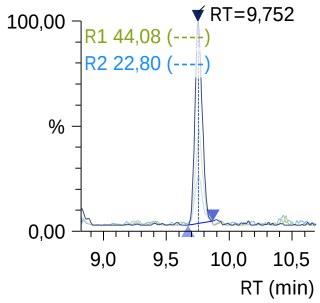

40ppb002

Conc 56,8177
Area 358222
R#1 273,00>146,90 46,87 (49,23)
R#2 273,00>118,95 26,23 (26,26)
Q 273,00>152,90 (+) 8,04e4

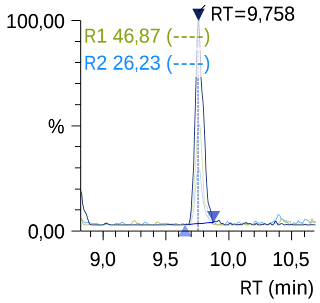

70 ppb001

Conc 127,8010
Area 752013
R#1 273,00>146,90 48,84 (49,23)
R#2 273,00>118,95 22,85 (26,26)
Q 273,00>152,90 (+) 1,71e5

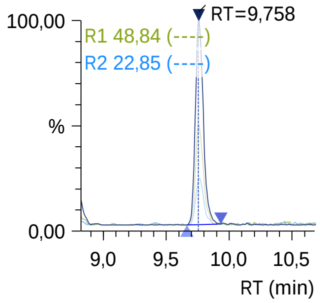

70ppb002

Conc 113,2691
Area 671395
R#1 273,00>146,90 48,81 (49,23)
R#2 273,00>118,95 25,82 (26,26)
Q 273,00>152,90 (+) 1,56e5

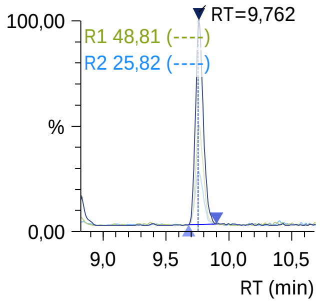

100 ppb001

Conc 118,0666
Area 698010
R#1 273,00>146,90 49,07 (49,23)
R#2 273,00>118,95 24,39 (26,26)
Q 273,00>152,90 (+) 1,50e5

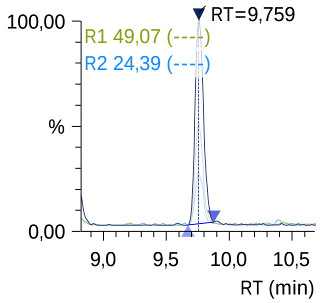

100ppb002

Conc 94,8347
Area 569128
R#1 273,00>146,90 48,30 (49,23)
R#2 273,00>118,95 23,66 (26,26)
Q 273,00>152,90 (+) 1,28e5

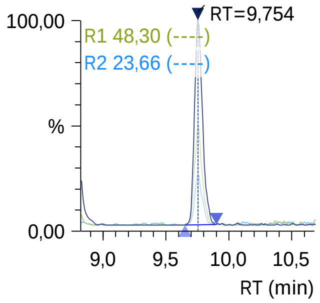

130 ppb001

Conc 119,5752
Area 706380
R#1 273,00>146,90 45,40 (49,23)
R#2 273,00>118,95 26,22 (26,26)
Q 273,00>152,90 (+) 1,63e5

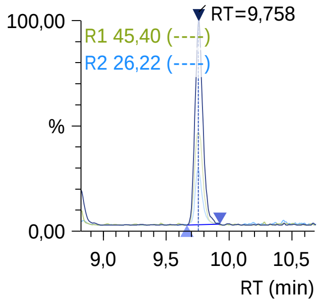

130ppb002

Conc 121,3890
Area 716442
R#1 273,00>146,90 50,10 (49,23)
R#2 273,00>118,95 24,35 (26,26)
Q 273,00>152,90 (+) 1,63e5

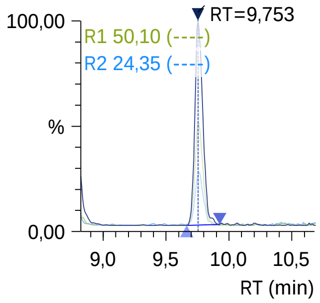

160 ppb001

Conc 160,4601
Area 933195
R#1 273,00>146,90 49,14 (49,23)
R#2 273,00>118,95 22,43 (26,26)
Q 273,00>152,90 (+) 2,10e5

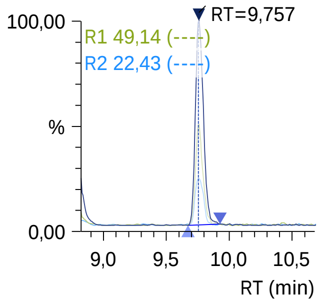

Compound: Naringenina (continued)

160ppb002
Conc 161,7041
Area 940096
R#1 273,00>146,90 47,99 (49,23)
R#2 273,00>118,95 19,66 (26,26)
Q 273,00>152,90 (+) 2,20e5

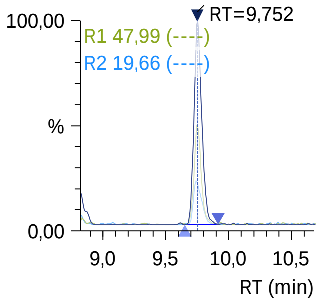

190 ppb001
Conc 178,3447
Area 1032413
R#1 273,00>146,90 52,30 (49,23)
R#2 273,00>118,95 26,11 (26,26)
Q 273,00>152,90 (+) 2,41e5

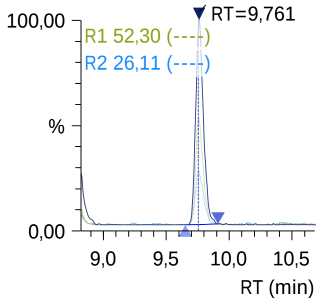

190ppb002
Conc 185,9895
Area 1074824
R#1 273,00>146,90 49,23 (49,23)
R#2 273,00>118,95 26,26 (26,26)
Q 273,00>152,90 (+) 2,56e5

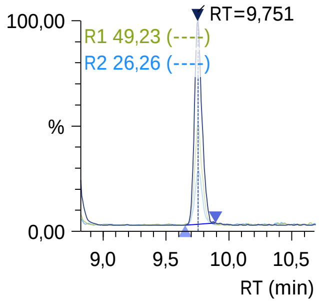

220 ppb001
Conc 220,0123
Area 1263571
R#1 273,00>146,90 44,74 (49,23)
R#2 273,00>118,95 21,62 (26,26)
Q 273,00>152,90 (+) 3,09e5

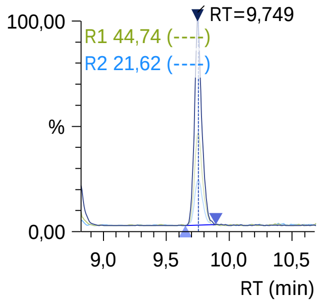

220ppb002
Conc 224,4748
Area 1288327
R#1 273,00>146,90 43,66 (49,23)
R#2 273,00>118,95 21,21 (26,26)
Q 273,00>152,90 (+) 3,07e5

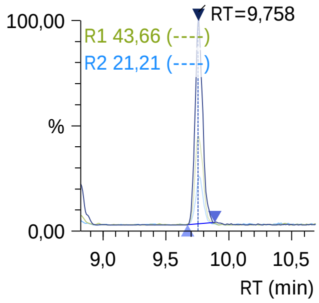

250 ppb001
Conc 254,0665
Area 1452492
R#1 273,00>146,90 49,57 (49,23)
R#2 273,00>118,95 24,97 (26,26)
Q 273,00>152,90 (+) 3,28e5

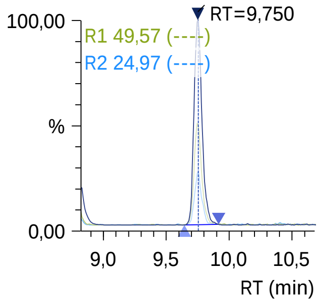

250 ppb002
Conc 254,2778
Area 1453664
R#1 273,00>146,90 49,93 (49,23)
R#2 273,00>118,95 24,14 (26,26)
Q 273,00>152,90 (+) 3,33e5

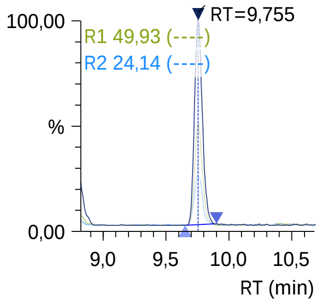

10H
Conc ----
Area ----
R#1 273,00>146,90 ---- (49,23)
R#2 273,00>118,95 ---- (26,26)
Q 273,00>152,90 (+) 7,22e2

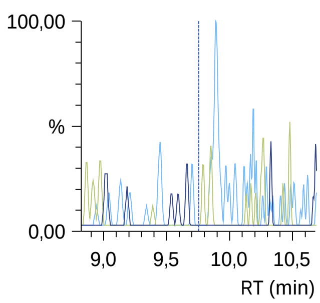

10M
Conc ----
Area ----
R#1 273,00>146,90 ---- (49,23)
R#2 273,00>118,95 ---- (26,26)
Q 273,00>152,90 (+) 6,60e2

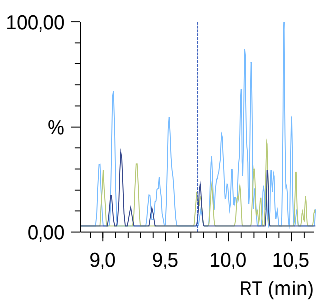

9H
Conc ----
Area ----
R#1 273,00>146,90 ---- (49,23)
R#2 273,00>118,95 ---- (26,26)
Q 273,00>152,90 (+) 2,28e3

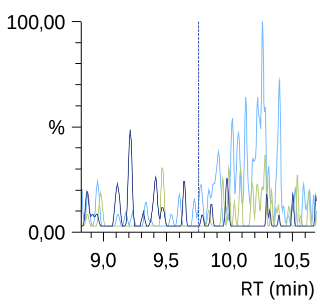

9M
Conc ----
Area ----
R#1 273,00>146,90 ---- (49,23)
R#2 273,00>118,95 ---- (26,26)
Q 273,00>152,90 (+) 1,56e3

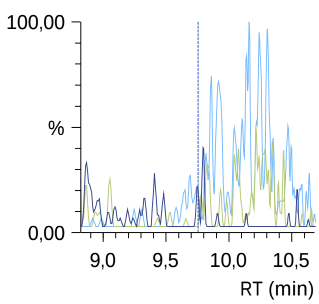

Compound: Seringaldeido

Curve Fit: Linear | Weighting: None | Zero: Not Forced
Quantitative Method: External Standard
Q 183,00>94,95
R^2 = 0.9922690 R = 0.9961270
y = 2340,794x - 775,4528

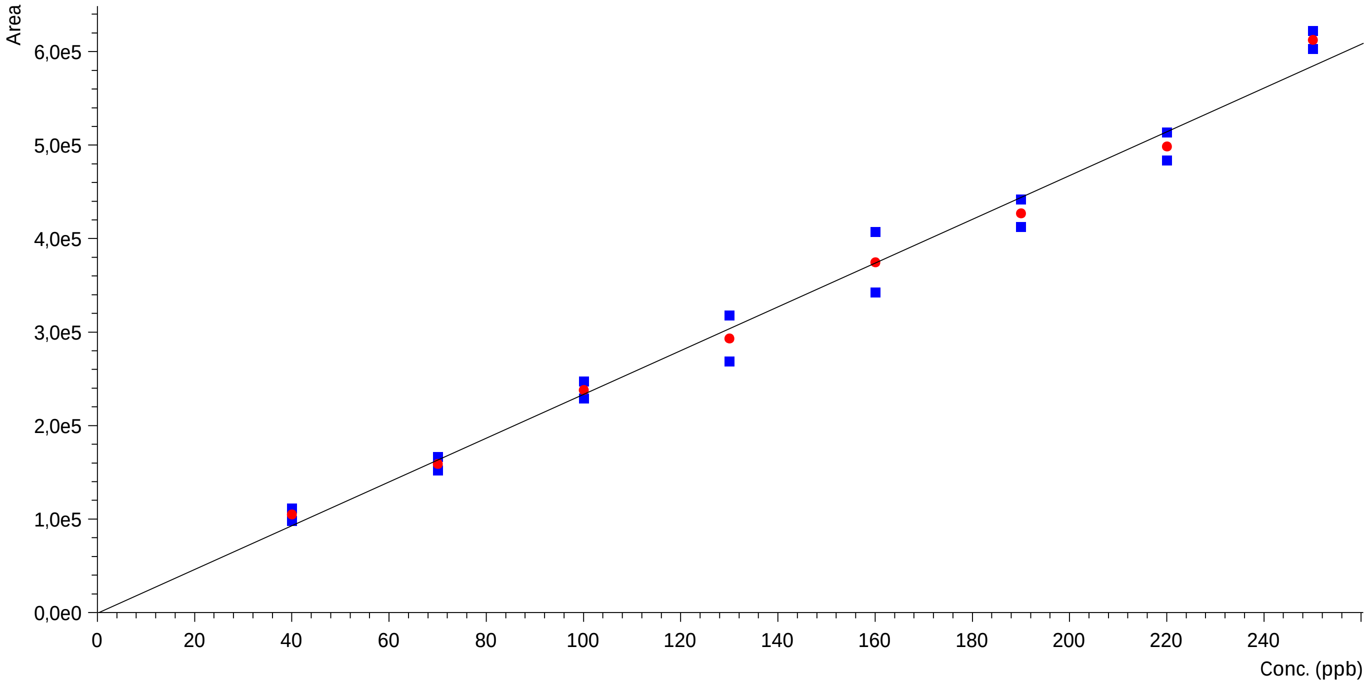

Sample ID	Area	Conc.	Sample Type	Level	Cal Point	Found RT	Ref 1 Actual Ratio
0	34471	15,0577	Ctrl.	0	----	8,290	80,61
10ppb001	----	----	Std.	1	No	----	----
10 ppb002	----	----	Std.	1	No	----	----
40 ppb001	111674	48,0392	Std.	2	Yes	8,727	118,30
40ppb002	98307	42,3287	Std.	2	Yes	8,730	99,31
70 ppb001	166289	71,3707	Std.	3	Yes	8,739	116,26
70ppb002	152180	65,3435	Std.	3	Yes	8,734	114,16
100 ppb001	229291	98,2857	Std.	4	Yes	8,731	121,09
100ppb002	247115	105,9000	Std.	4	Yes	8,739	111,57
130 ppb001	268880	115,1985	Std.	5	Yes	8,730	114,11
130ppb002	318022	136,1922	Std.	5	Yes	8,738	140,43
160 ppb001	342807	146,7805	Std.	6	Yes	8,739	118,16
160ppb002	406858	174,1431	Std.	6	Yes	8,733	116,67
190 ppb001	441885	189,1069	Std.	7	Yes	8,746	123,76
190ppb002	412346	176,4877	Std.	7	Yes	8,730	144,42
220 ppb001	483684	206,9639	Std.	8	Yes	8,735	124,24
220ppb002	513553	219,7240	Std.	8	Yes	8,743	112,89
250 ppb001	602860	257,8765	Std.	9	Yes	8,734	117,09
250 ppb002	622482	266,2590	Std.	9	Yes	8,733	112,47
10H	----	----	Unk.	----	----	----	----
10M	----	----	Unk.	----	----	----	----
9H	78739	33,9689	Unk.	----	----	8,766	69,31
9M	92081	39,6687	Unk.	----	----	8,751	94,80
Average	295975	126,7734				8,714	113,14
%RSD	62	61,9546				1,183	15,62
Maximum	622482	266,2590				8,766	144,42
Minimum	34471	15,0577				8,290	69,31
Std. Dev.	183850	78,5419				0,103	17,68

Compound: Seringaldeido (continued)

0

Conc 15,0577
Area 34471
R#1 183,00>76,95 80,61 (99,31)
R#2 183,00>122,90 45,22 (57,55)
Q 183,00>94,95 (+) 1,10e4

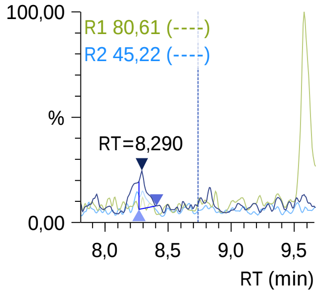

10ppb001

Conc ----
Area ----
R#1 183,00>76,95 ---- (99,31)
R#2 183,00>122,90 ---- (57,55)
Q 183,00>94,95 (+) 1,33e4

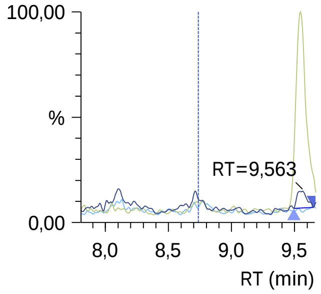

10 ppb002

Conc ----
Area ----
R#1 183,00>76,95 ---- (99,31)
R#2 183,00>122,90 ---- (57,55)
Q 183,00>94,95 (+) 1,33e4

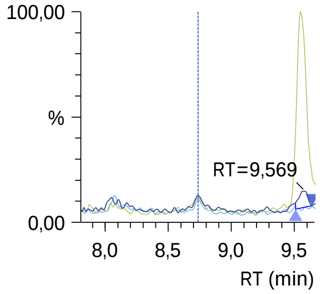

40 ppb001

Conc 48,0392
Area 111674
R#1 183,00>76,95 118,30 (99,31)
R#2 183,00>122,90 48,33 (57,55)
Q 183,00>94,95 (+) 2,54e4

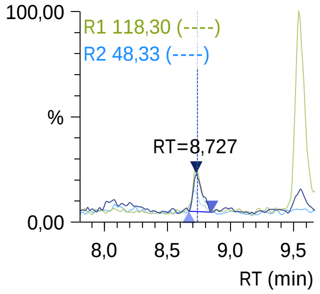

40ppb002

Conc 42,3287
Area 98307
R#1 183,00>76,95 99,31 (99,31)
R#2 183,00>122,90 57,55 (57,55)
Q 183,00>94,95 (+) 2,71e4

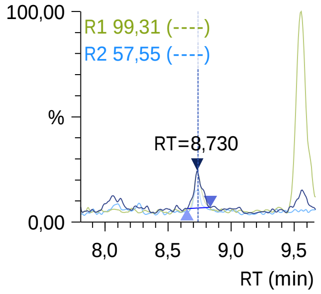

70 ppb001

Conc 71,3707
Area 166289
R#1 183,00>76,95 116,26 (99,31)
R#2 183,00>122,90 54,46 (57,55)
Q 183,00>94,95 (+) 4,85e4

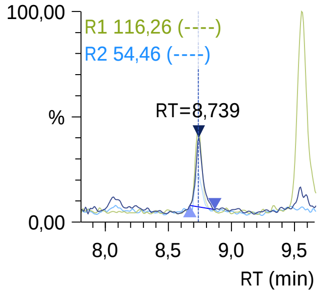

70ppb002

Conc 65,3435
Area 152180
R#1 183,00>76,95 114,16 (99,31)
R#2 183,00>122,90 55,09 (57,55)
Q 183,00>94,95 (+) 3,96e4

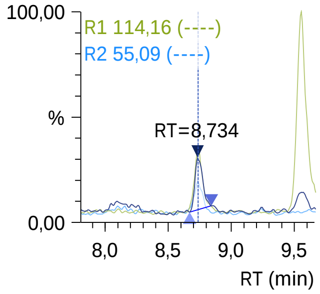

100 ppb001

Conc 98,2857
Area 229291
R#1 183,00>76,95 121,09 (99,31)
R#2 183,00>122,90 48,68 (57,55)
Q 183,00>94,95 (+) 5,58e4

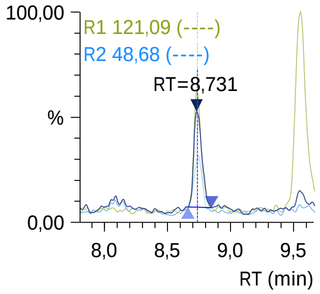

100ppb002

Conc 105,9000
Area 247115
R#1 183,00>76,95 111,57 (99,31)
R#2 183,00>122,90 64,62 (57,55)
Q 183,00>94,95 (+) 6,11e4

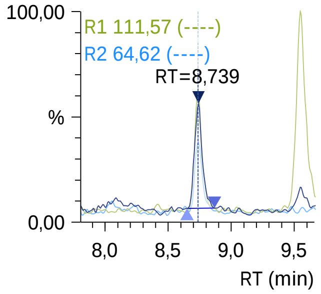

130 ppb001

Conc 115,1985
Area 268880
R#1 183,00>76,95 114,11 (99,31)
R#2 183,00>122,90 66,36 (57,55)
Q 183,00>94,95 (+) 7,37e4

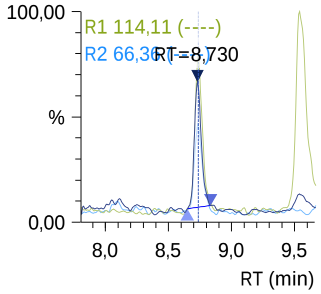

130ppb002

Conc 136,1922
Area 318022
R#1 183,00>76,95 140,43 (99,31)
R#2 183,00>122,90 60,92 (57,55)
Q 183,00>94,95 (+) 7,08e4

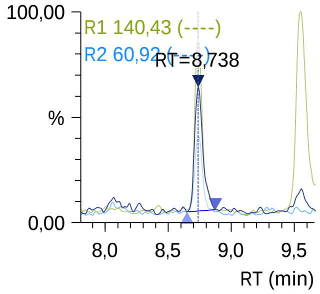

160 ppb001

Conc 146,7805
Area 342807
R#1 183,00>76,95 118,16 (99,31)
R#2 183,00>122,90 60,69 (57,55)
Q 183,00>94,95 (+) 8,74e4

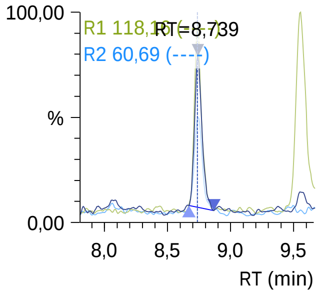

Compound: Seringaldeido (continued)

160ppb002
Conc 174,1431
Area 406858
R#1 183,00>76,95 116,67 (99,31)
R#2 183,00>122,90 54,81 (57,55)
Q 183,00>94,95 (+)   9,02e4

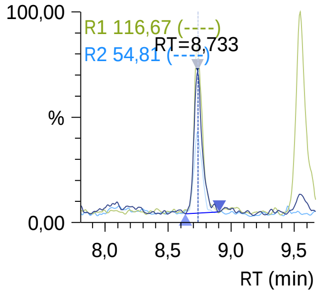

190 ppb001
Conc 189,1069
Area 441885
R#1 183,00>76,95 123,76 (99,31)
R#2 183,00>122,90 76,06 (57,55)
Q 183,00>94,95 (+)   1,00e5

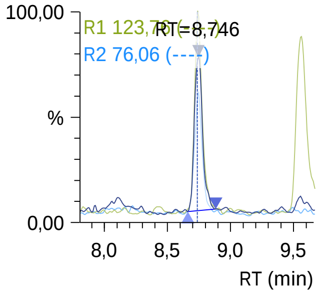

190ppb002
Conc 176,4877
Area 412346
R#1 183,00>76,95 144,42 (99,31)
R#2 183,00>122,90 76,32 (57,55)
Q 183,00>94,95 (+)   9,59e4

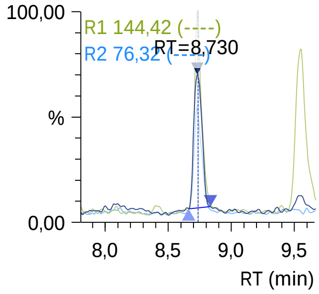

220 ppb001
Conc 206,9639
Area 483684
R#1 183,00>76,95 124,24 (99,31)
R#2 183,00>122,90 68,84 (57,55)
Q 183,00>94,95 (+)   1,20e5

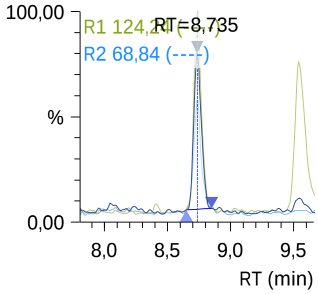

220ppb002
Conc 219,7240
Area 513553
R#1 183,00>76,95 112,89 (99,31)
R#2 183,00>122,90 72,37 (57,55)
Q 183,00>94,95 (+)   1,26e5

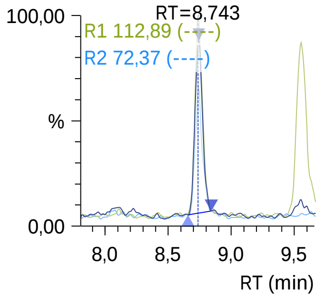

250 ppb001
Conc 257,8765
Area 602860
R#1 183,00>76,95 117,09 (99,31)
R#2 183,00>122,90 66,69 (57,55)
Q 183,00>94,95 (+)   1,49e5

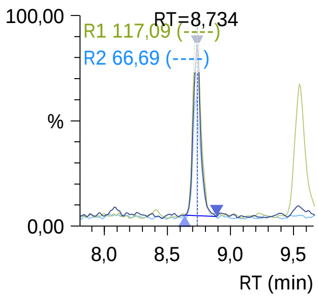

250 ppb002
Conc 266,2590
Area 622482
R#1 183,00>76,95 112,47 (99,31)
R#2 183,00>122,90 60,74 (57,55)
Q 183,00>94,95 (+)   1,56e5

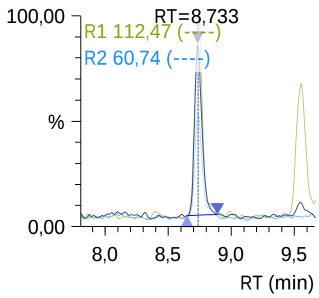

10H
Conc ----
Area ----
R#1 183,00>76,95 ---- (99,31)
R#2 183,00>122,90 ---- (57,55)
Q 183,00>94,95 (+)   6,09e3

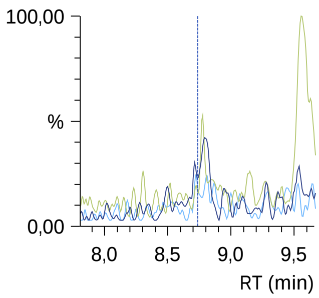

10M
Conc ----
Area ----
R#1 183,00>76,95 ---- (99,31)
R#2 183,00>122,90 ---- (57,55)
Q 183,00>94,95 (+)   5,28e3

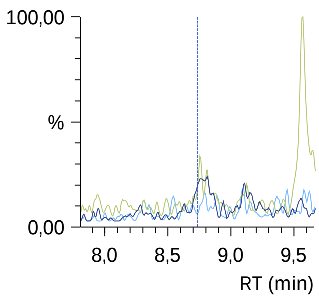

9H
Conc 33,9689
Area 78739
R#1 183,00>76,95 69,31 (99,31)
R#2 183,00>122,90 40,56 (57,55)
Q 183,00>94,95 (+)   1,96e4

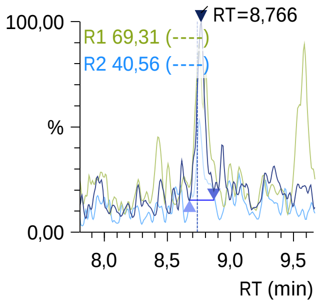

9M
Conc 39,6687
Area 92081
R#1 183,00>76,95 94,80 (99,31)
R#2 183,00>122,90 42,98 (57,55)
Q 183,00>94,95 (+)   1,86e4

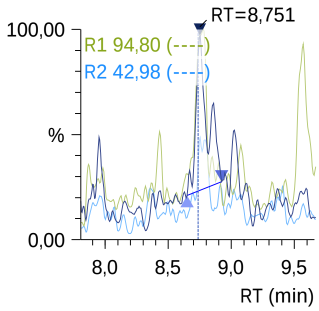

Compound: Ac. Clorogenico

Curve Fit: Linear | Weighting: None | Zero: Not Forced
Quantitative Method: External Standard
Q 354,80>162,95
R^2 = 0.9962179 R = 0.9981072
y = 2265,823x - 21644,46

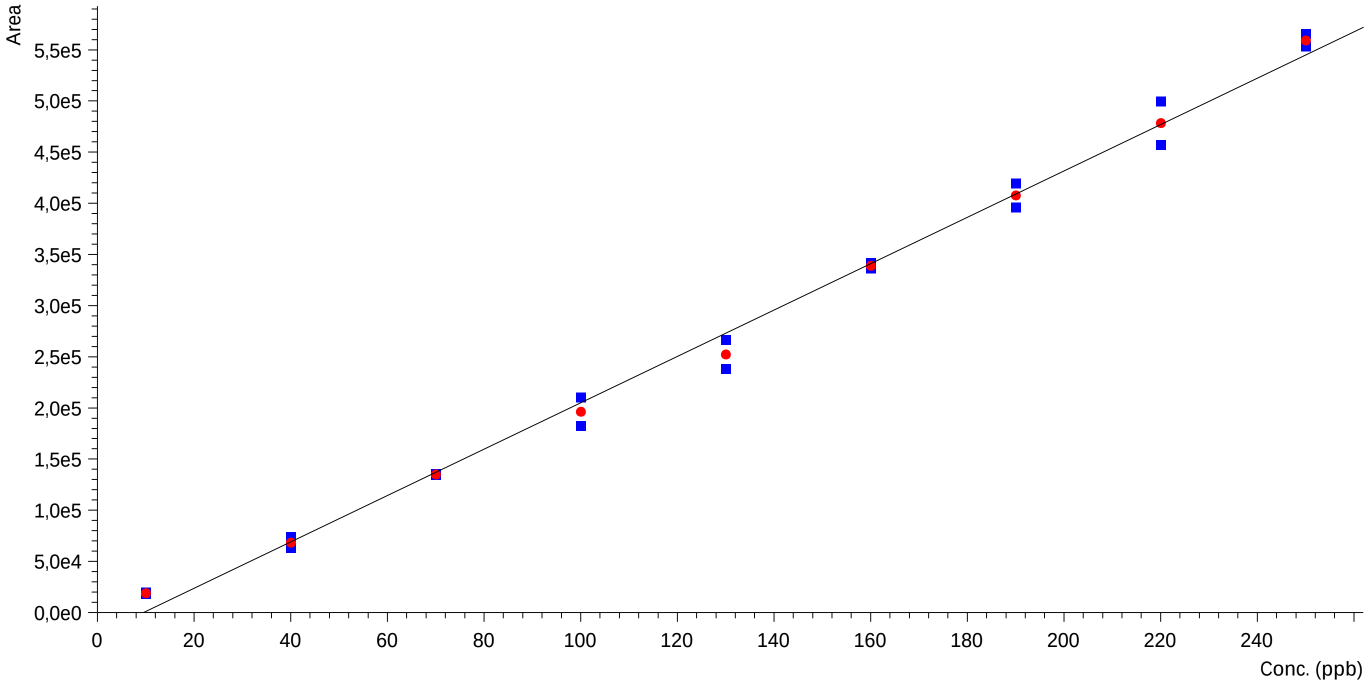

Sample ID	Area	Conc.	Sample Type	Level	Cal Point	Found RT	Ref 1 Actual Ratio
0	----	----	Ctrl.	0	----	----	----
10ppb001	19830	18,3042	Std.	1	Yes	7,920	42,28
10 ppb002	18529	17,7301	Std.	1	Yes	7,938	78,03
40 ppb001	73835	42,1392	Std.	2	Yes	7,915	54,00
40ppb002	63255	37,4694	Std.	2	Yes	7,929	60,73
70 ppb001	135496	69,3524	Std.	3	Yes	7,922	73,68
70ppb002	134827	69,0571	Std.	3	Yes	7,922	70,37
100 ppb001	182577	90,1314	Std.	4	Yes	7,927	73,83
100ppb002	210206	102,3252	Std.	4	Yes	7,925	71,98
130 ppb001	266484	127,1630	Std.	5	Yes	7,922	63,16
130ppb002	238072	114,6236	Std.	5	Yes	7,924	70,80
160 ppb001	336209	157,9351	Std.	6	Yes	7,927	72,08
160ppb002	341881	160,4388	Std.	6	Yes	7,923	73,04
190 ppb001	396115	184,3744	Std.	7	Yes	7,928	64,57
190ppb002	419360	194,6333	Std.	7	Yes	7,921	63,02
220 ppb001	457301	211,3782	Std.	8	Yes	7,930	71,27
220ppb002	499640	230,0643	Std.	8	Yes	7,931	59,96
250 ppb001	565388	259,0812	Std.	9	Yes	7,922	63,99
250 ppb002	553420	253,7992	Std.	9	Yes	7,924	64,50
10H	12278	14,9715	Unk.	----	----	7,933	64,85
10M	32095	23,7175	Unk.	----	----	7,933	27,36
9H	11128	14,4637	Unk.	----	----	7,953	57,01
9M	22663	19,5547	Unk.	----	----	7,922	115,69
Average	226845	109,6685				7,927	66,19
%RSD	84	77,0127				0,101	23,99
Maximum	565388	259,0812				7,953	115,69
Minimum	11128	14,4637				7,915	27,36
Std. Dev.	191368	84,4587				0,008	15,88

Compound: Ac. Clorogenico (continued)

0
Conc ----
Area ----
R#1 354,80>88,95 ---- (73,04)
R#2 354,80>135,00 ---- (29,33)
Q 354,80>162,95 (+) 1,14e3

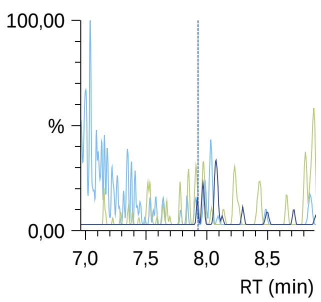

10ppb001
Conc 18,3042
Area 19830
R#1 354,80>88,95 42,28 (73,04)
R#2 354,80>135,00 16,46 (29,33)
Q 354,80>162,95 (+) 5,39e3

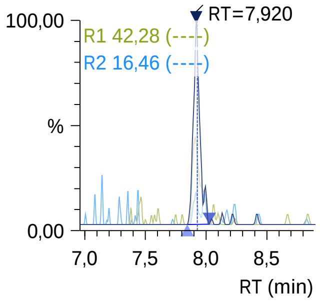

10 ppb002
Conc 17,7301
Area 18529
R#1 354,80>88,95 78,03 (73,04)
R#2 354,80>135,00 36,64 (29,33)
Q 354,80>162,95 (+) 4,97e3

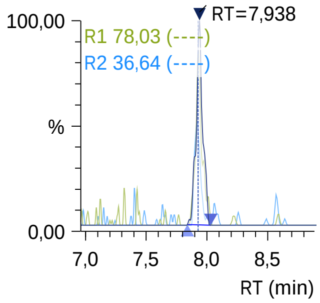

40 ppb001
Conc 42,1392
Area 73835
R#1 354,80>88,95 54,00 (73,04)
R#2 354,80>135,00 35,45 (29,33)
Q 354,80>162,95 (+) 1,74e4

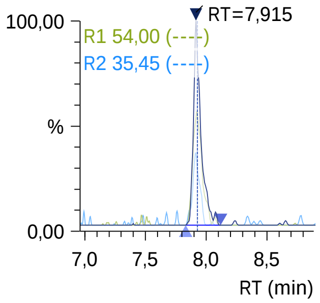

40ppb002
Conc 37,4694
Area 63255
R#1 354,80>88,95 60,73 (73,04)
R#2 354,80>135,00 38,21 (29,33)
Q 354,80>162,95 (+) 1,41e4

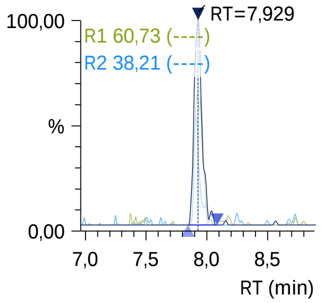

70 ppb001
Conc 69,3524
Area 135496
R#1 354,80>88,95 73,68 (73,04)
R#2 354,80>135,00 40,08 (29,33)
Q 354,80>162,95 (+) 3,05e4

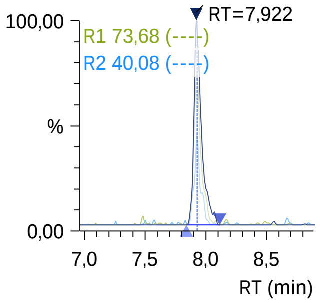

70ppb002
Conc 69,0571
Area 134827
R#1 354,80>88,95 70,37 (73,04)
R#2 354,80>135,00 39,01 (29,33)
Q 354,80>162,95 (+) 2,70e4

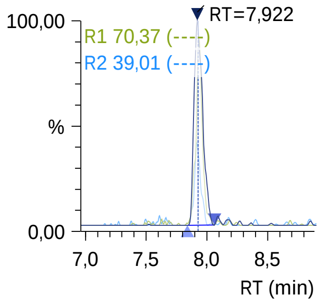

100 ppb001
Conc 90,1314
Area 182577
R#1 354,80>88,95 73,83 (73,04)
R#2 354,80>135,00 31,33 (29,33)
Q 354,80>162,95 (+) 3,80e4

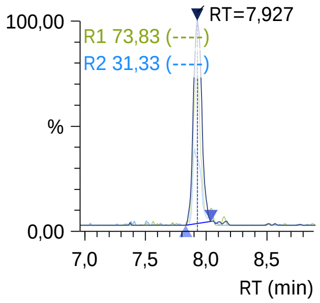

100ppb002
Conc 102,3252
Area 210206
R#1 354,80>88,95 71,98 (73,04)
R#2 354,80>135,00 36,32 (29,33)
Q 354,80>162,95 (+) 4,45e4

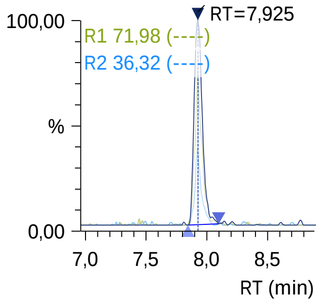

130 ppb001
Conc 127,1630
Area 266484
R#1 354,80>88,95 63,16 (73,04)
R#2 354,80>135,00 23,87 (29,33)
Q 354,80>162,95 (+) 6,15e4

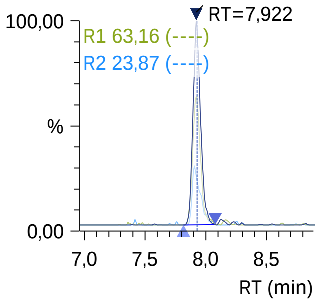

130ppb002
Conc 114,6236
Area 238072
R#1 354,80>88,95 70,80 (73,04)
R#2 354,80>135,00 28,16 (29,33)
Q 354,80>162,95 (+) 5,88e4

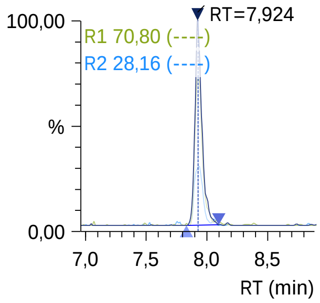

160 ppb001
Conc 157,9351
Area 336209
R#1 354,80>88,95 72,08 (73,04)
R#2 354,80>135,00 31,64 (29,33)
Q 354,80>162,95 (+) 7,42e4

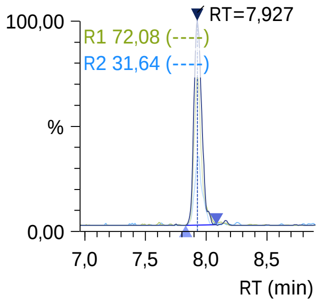

Compound: Ac. Clorogenico (continued)

160ppb002
Conc 160,4388
Area 341881
R#1 354,80>88,95 73,04 (73,04)
R#2 354,80>135,00 29,33 (29,33)
Q 354,80>162,95 (+) 8,03e4

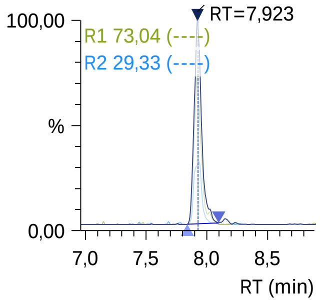

190 ppb001
Conc 184,3744
Area 396115
R#1 354,80>88,95 64,57 (73,04)
R#2 354,80>135,00 38,54 (29,33)
Q 354,80>162,95 (+) 8,91e4

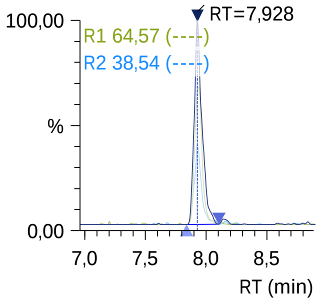

190ppb002
Conc 194,6333
Area 419360
R#1 354,80>88,95 63,02 (73,04)
R#2 354,80>135,00 33,57 (29,33)
Q 354,80>162,95 (+) 9,21e4

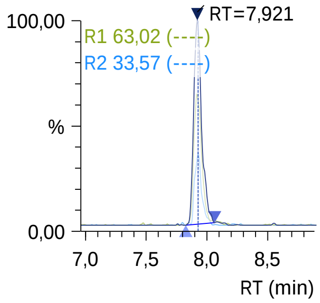

220 ppb001
Conc 211,3782
Area 457301
R#1 354,80>88,95 71,27 (73,04)
R#2 354,80>135,00 35,14 (29,33)
Q 354,80>162,95 (+) 1,10e5

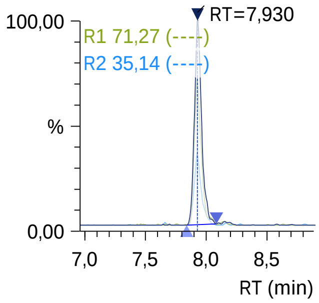

220ppb002
Conc 230,0643
Area 499640
R#1 354,80>88,95 59,96 (73,04)
R#2 354,80>135,00 28,55 (29,33)
Q 354,80>162,95 (+) 1,12e5

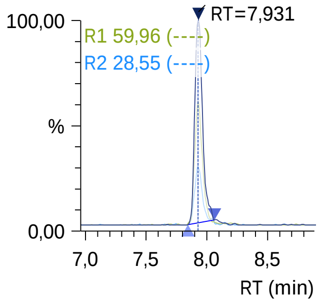

250 ppb001
Conc 259,0812
Area 565388
R#1 354,80>88,95 63,99 (73,04)
R#2 354,80>135,00 32,88 (29,33)
Q 354,80>162,95 (+) 1,32e5

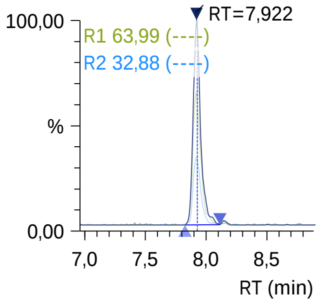

250 ppb002
Conc 253,7992
Area 553420
R#1 354,80>88,95 64,50 (73,04)
R#2 354,80>135,00 29,47 (29,33)
Q 354,80>162,95 (+) 1,26e5

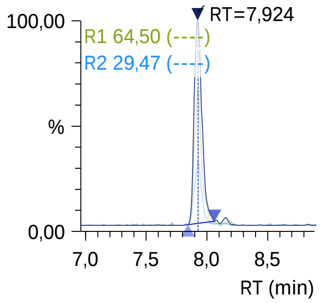

10H
Conc 14,9715
Area 12278
R#1 354,80>88,95 64,85 (73,04)
R#2 354,80>135,00 0,00 (29,33)
Q 354,80>162,95 (+) 2,03e3

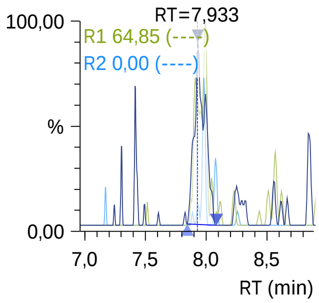

10M
Conc 23,7175
Area 32095
R#1 354,80>88,95 27,36 (73,04)
R#2 354,80>135,00 27,16 (29,33)
Q 354,80>162,95 (+) 9,11e3

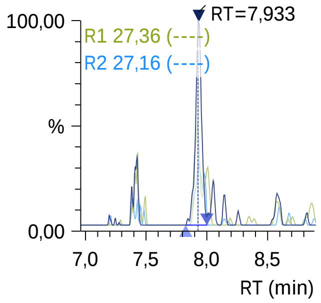

9H
Conc 14,4637
Area 11128
R#1 354,80>88,95 57,01 (73,04)
R#2 354,80>135,00 0,00 (29,33)
Q 354,80>162,95 (+) 2,03e3

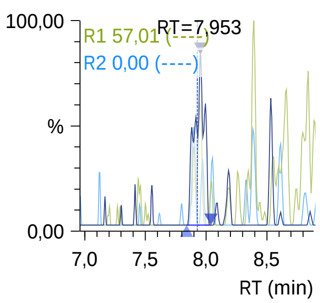

9M
Conc 19,5547
Area 22663
R#1 354,80>88,95 115,69 (73,04)
R#2 354,80>135,00 37,52 (29,33)
Q 354,80>162,95 (+) 3,63e3

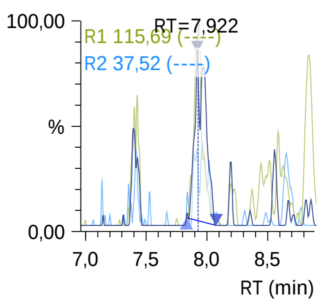

Compound: Coniferil Aldeido

Curve Fit: Linear | Weighting: None | Zero: Not Forced
Quantitative Method: External Standard
Q 178,90>90,95
R^2 = 0.9956635 R = 0.9978294
y = 1419,701x - 414,4849

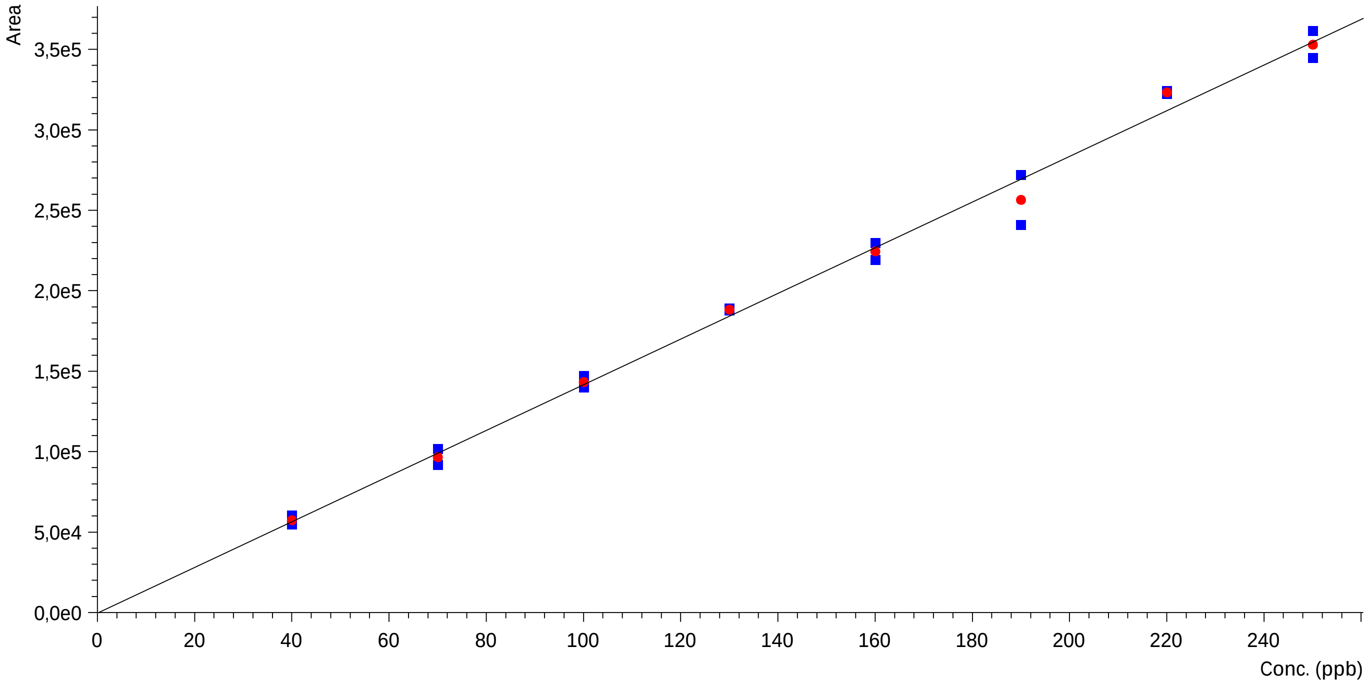

Sample ID	Area	Conc.	Sample Type	Level	Cal Point	Found RT	Ref 1 Actual Ratio
0	----	----	Ctrl.	0	----	----	----
10ppb001	----	----	Std.	1	No	----	----
10 ppb002	----	----	Std.	1	No	----	----
40 ppb001	60361	42,8086	Std.	2	Yes	9,071	38,88
40ppb002	54787	38,8825	Std.	2	Yes	9,076	45,55
70 ppb001	91644	64,8438	Std.	3	Yes	9,090	60,33
70ppb002	101697	71,9248	Std.	3	Yes	9,087	41,46
100 ppb001	146926	103,7829	Std.	4	Yes	9,087	48,56
100ppb002	139979	98,8896	Std.	4	Yes	9,090	44,46
130 ppb001	188938	133,3749	Std.	5	Yes	9,084	55,31
130ppb002	187702	132,5046	Std.	5	Yes	9,091	52,93
160 ppb001	219259	154,7319	Std.	6	Yes	9,087	51,20
160ppb002	229752	162,1235	Std.	6	Yes	9,084	49,61
190 ppb001	272031	191,9032	Std.	7	Yes	9,092	61,27
190ppb002	240986	170,0359	Std.	7	Yes	9,087	52,39
220 ppb001	322431	227,4038	Std.	8	Yes	9,083	64,18
220ppb002	324446	228,8230	Std.	8	Yes	9,092	47,06
250 ppb001	344592	243,0139	Std.	9	Yes	9,079	67,14
250 ppb002	361543	254,9533	Std.	9	Yes	9,086	50,11
10H	----	----	Unk.	----	----	----	----
10M	----	----	Unk.	----	----	----	----
9H	----	----	Unk.	----	----	----	----
9M	----	----	Unk.	----	----	----	----
Average	205442	145,0000				9,085	51,90
%RSD	49	49,1910				0,065	15,52
Maximum	361543	254,9533				9,092	67,14
Minimum	54787	38,8825				9,071	38,88
Std. Dev.	101263	71,3270				0,006	8,05

Compound: Coniferil Aldeido (continued)

0
Conc ----
Area ----
R#1 178,90>146,90 ---- (49,61)
R#2 178,90>119,00 ---- (58,25)
Q 178,90>90,95 (+) 5,41e3

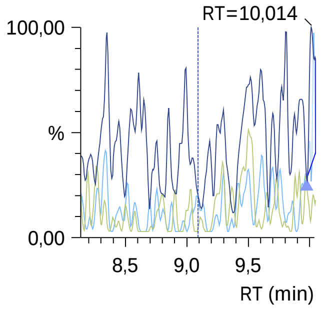

10ppb001
Conc ----
Area ----
R#1 178,90>146,90 ---- (49,61)
R#2 178,90>119,00 ---- (58,25)
Q 178,90>90,95 (+) 1,13e4

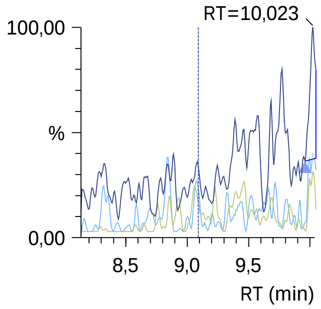

10 ppb002
Conc ----
Area ----
R#1 178,90>146,90 ---- (49,61)
R#2 178,90>119,00 ---- (58,25)
Q 178,90>90,95 (+) 1,52e4

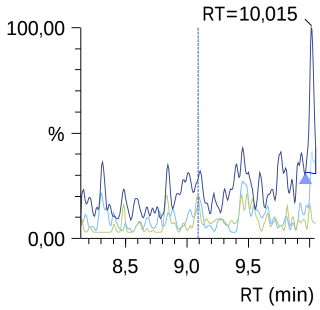

40 ppb001
Conc 42,8086
Area 60361
R#1 178,90>146,90 38,88 (49,61)
R#2 178,90>119,00 30,00 (58,25)
Q 178,90>90,95 (+) 2,21e4

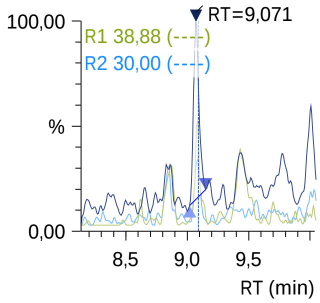

40ppb002
Conc 38,8825
Area 54787
R#1 178,90>146,90 45,55 (49,61)
R#2 178,90>119,00 57,09 (58,25)
Q 178,90>90,95 (+) 1,26e4

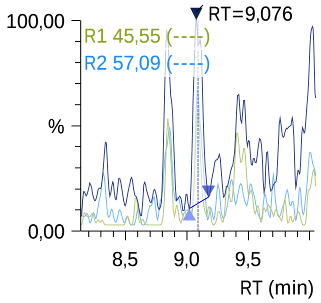

70 ppb001
Conc 64,8438
Area 91644
R#1 178,90>146,90 60,33 (49,61)
R#2 178,90>119,00 62,23 (58,25)
Q 178,90>90,95 (+) 2,63e4

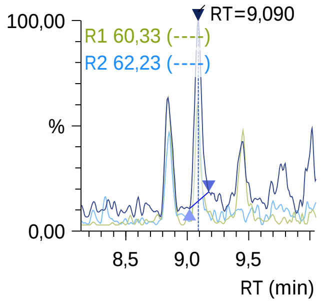

70ppb002
Conc 71,9248
Area 101697
R#1 178,90>146,90 41,46 (49,61)
R#2 178,90>119,00 41,36 (58,25)
Q 178,90>90,95 (+) 3,04e4

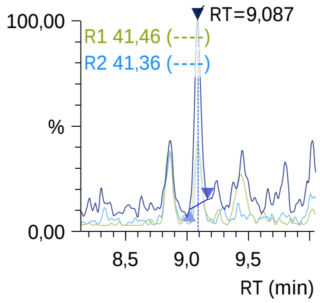

100 ppb001
Conc 103,7829
Area 146926
R#1 178,90>146,90 48,56 (49,61)
R#2 178,90>119,00 42,81 (58,25)
Q 178,90>90,95 (+) 4,82e4

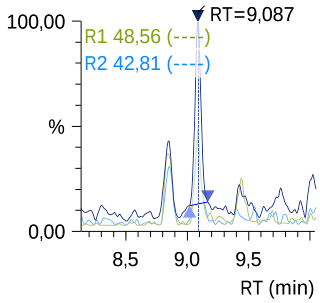

100ppb002
Conc 98,8896
Area 139979
R#1 178,90>146,90 44,46 (49,61)
R#2 178,90>119,00 50,90 (58,25)
Q 178,90>90,95 (+) 4,58e4

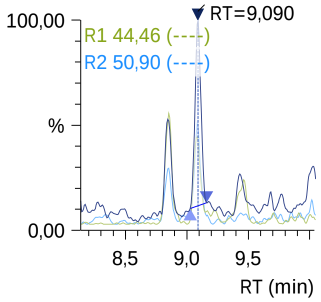

130 ppb001
Conc 133,3749
Area 188938
R#1 178,90>146,90 55,31 (49,61)
R#2 178,90>119,00 35,62 (58,25)
Q 178,90>90,95 (+) 5,66e4

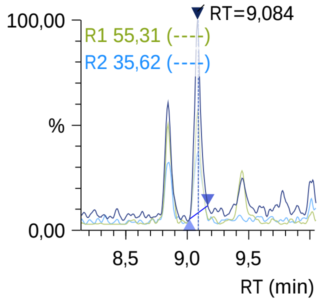

130ppb002
Conc 132,5046
Area 187702
R#1 178,90>146,90 52,93 (49,61)
R#2 178,90>119,00 47,43 (58,25)
Q 178,90>90,95 (+) 5,41e4

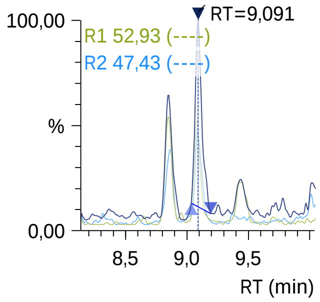

160 ppb001
Conc 154,7319
Area 219259
R#1 178,90>146,90 51,20 (49,61)
R#2 178,90>119,00 45,90 (58,25)
Q 178,90>90,95 (+) 7,02e4

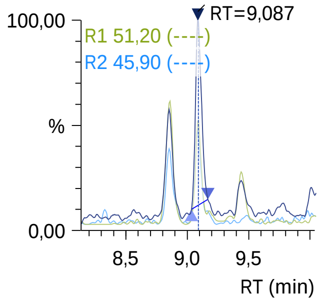

Compound: Coniferil Aldeido (continued)

160ppb002
Conc 162,1235
Area 229752
R#1 178,90>146,90 49,61 (49,61)
R#2 178,90>119,00 58,25 (58,25)
Q 178,90>90,95 (+) 6,56e4

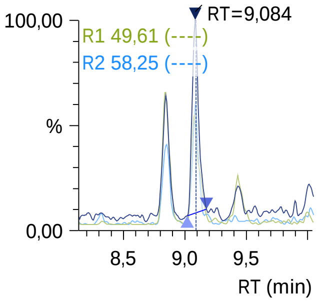

190 ppb001
Conc 191,9032
Area 272031
R#1 178,90>146,90 61,27 (49,61)
R#2 178,90>119,00 43,66 (58,25)
Q 178,90>90,95 (+) 7,50e4

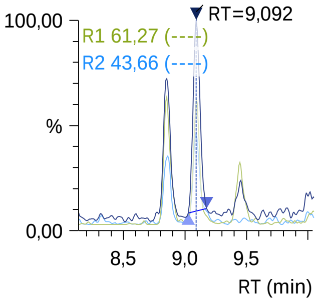

190ppb002
Conc 170,0359
Area 240986
R#1 178,90>146,90 52,39 (49,61)
R#2 178,90>119,00 58,87 (58,25)
Q 178,90>90,95 (+) 7,48e4

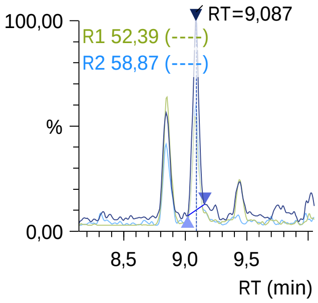

220 ppb001
Conc 227,4038
Area 322431
R#1 178,90>146,90 64,18 (49,61)
R#2 178,90>119,00 53,66 (58,25)
Q 178,90>90,95 (+) 7,94e4

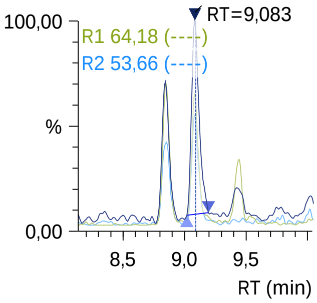

220ppb002
Conc 228,8230
Area 324446
R#1 178,90>146,90 47,06 (49,61)
R#2 178,90>119,00 45,59 (58,25)
Q 178,90>90,95 (+) 9,33e4

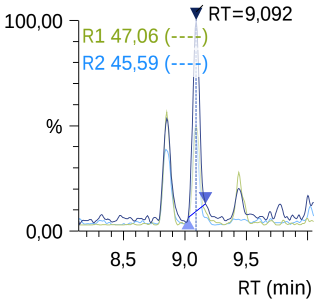

250 ppb001
Conc 243,0139
Area 344592
R#1 178,90>146,90 67,14 (49,61)
R#2 178,90>119,00 50,51 (58,25)
Q 178,90>90,95 (+) 9,32e4

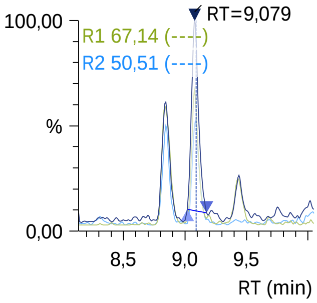

250 ppb002
Conc 254,9533
Area 361543
R#1 178,90>146,90 50,11 (49,61)
R#2 178,90>119,00 43,02 (58,25)
Q 178,90>90,95 (+) 1,05e5

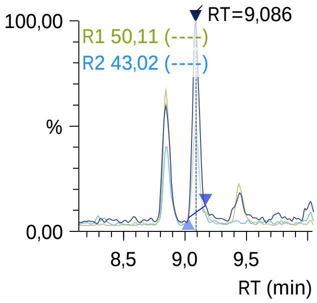

10H
Conc ----
Area ----
R#1 178,90>146,90 ---- (49,61)
R#2 178,90>119,00 ---- (58,25)
Q 178,90>90,95 (+) 9,97e3

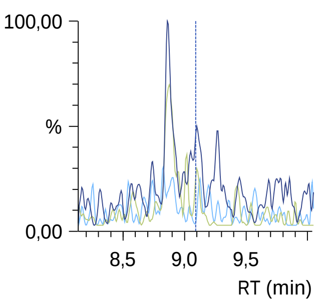

10M
Conc ----
Area ----
R#1 178,90>146,90 ---- (49,61)
R#2 178,90>119,00 ---- (58,25)
Q 178,90>90,95 (+) 1,56e4

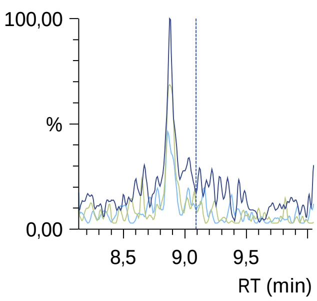

9H
Conc ----
Area ----
R#1 178,90>146,90 ---- (49,61)
R#2 178,90>119,00 ---- (58,25)
Q 178,90>90,95 (+) 1,67e4

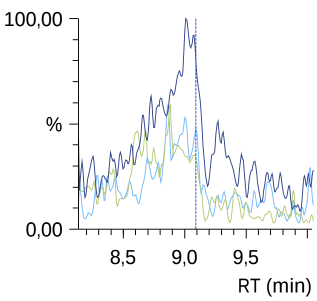

9M
Conc ----
Area ----
R#1 178,90>146,90 ---- (49,61)
R#2 178,90>119,00 ---- (58,25)
Q 178,90>90,95 (+) 4,56e4

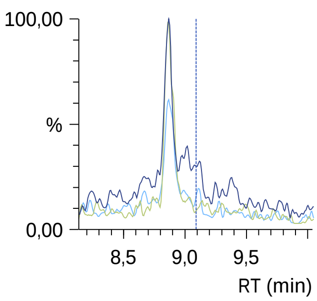

Compound: Ac. Sinapico

Curve Fit: Linear | Weighting: None | Zero: Not Forced
Quantitative Method: External Standard
Q 225,00>206,95
R^2 = 0.9297312 R = 0.9642257
y = 969,0258x + 9042,887

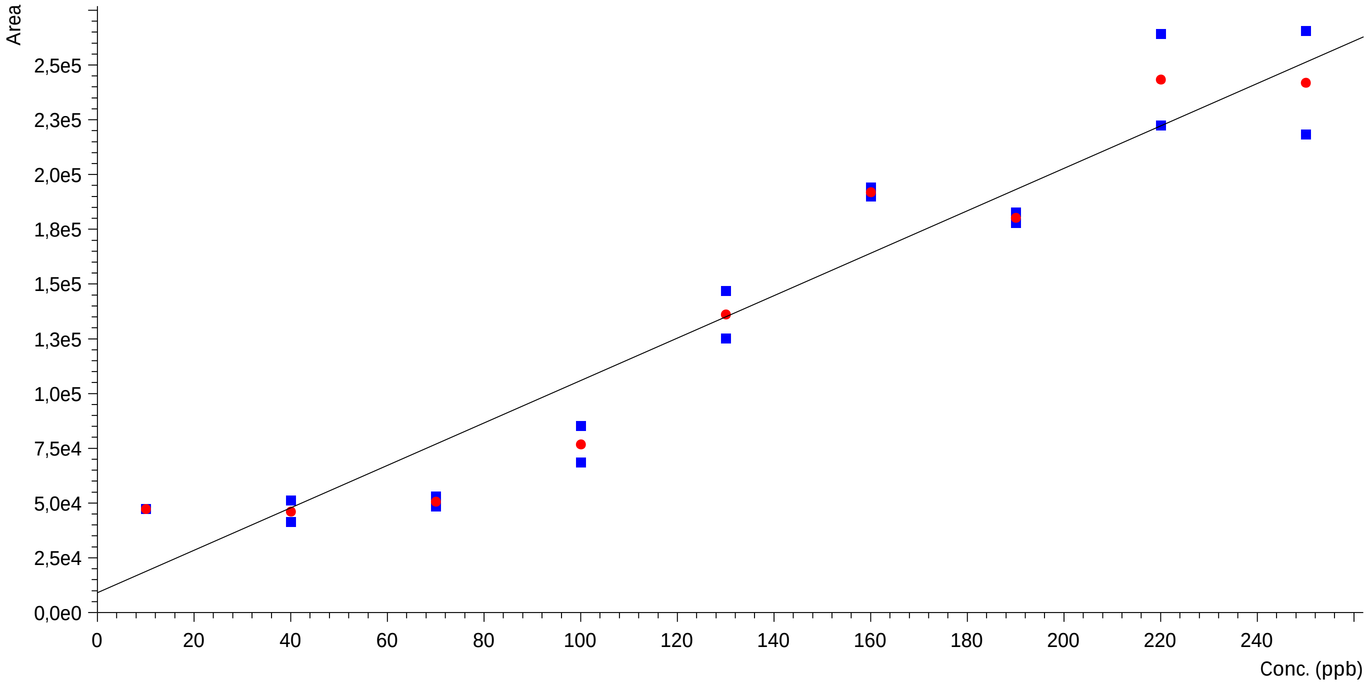

Sample ID	Area	Conc.	Sample Type	Level	Cal Point	Found RT	Ref 1 Actual Ratio
0	----	----	Ctrl.	0	----	----	----
10ppb001	47385	39,5674	Std.	1	Yes	8,919	0,00
10 ppb002	----	----	Std.	1	No	----	----
40 ppb001	51082	43,3831	Std.	2	Yes	8,885	54,16
40ppb002	41341	33,3305	Std.	2	Yes	8,878	39,25
70 ppb001	52970	45,3309	Std.	3	Yes	8,889	60,54
70ppb002	48525	40,7437	Std.	3	Yes	8,900	70,87
100 ppb001	68655	61,5172	Std.	4	Yes	8,884	63,25
100ppb002	85170	78,5605	Std.	4	Yes	8,889	51,80
130 ppb001	125277	119,9495	Std.	5	Yes	8,875	49,88
130ppb002	146988	142,3540	Std.	5	Yes	8,881	34,31
160 ppb001	189886	186,6234	Std.	6	Yes	8,888	50,08
160ppb002	194237	191,1139	Std.	6	Yes	8,881	53,30
190 ppb001	177936	174,2913	Std.	7	Yes	8,889	49,28
190ppb002	182692	179,2000	Std.	7	Yes	8,889	56,43
220 ppb001	222564	220,3466	Std.	8	Yes	8,882	57,58
220ppb002	264221	263,3348	Std.	8	Yes	8,893	52,12
250 ppb001	265673	264,8334	Std.	9	Yes	8,875	47,58
250 ppb002	218306	215,9522	Std.	9	Yes	8,879	65,86
10H	----	----	Unk.	----	----	----	----
10M	----	----	Unk.	----	----	----	----
9H	----	----	Unk.	----	----	----	----
9M	----	----	Unk.	----	----	----	----
Average	140171	135,3196				8,887	50,37
%RSD	57	61,3022				0,117	31,29
Maximum	265673	264,8334				8,919	70,87
Minimum	41341	33,3305				8,875	0,00
Std. Dev.	80384	82,9539				0,010	15,76

Compound: Ac. Sinapico (continued)

0
Conc ----
Area ----
R#1 225,00>174,95 ---- (65,86)
R#2 225,00>90,95 ---- (87,91)
Q 225,00>206,95 (+) 1,13e4

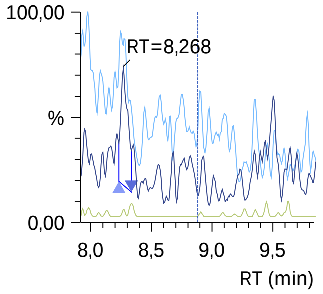

10ppb001
Conc 39,5674
Area 47385
R#1 225,00>174,95 0,00 (65,86)
R#2 225,00>90,95 0,00 (87,91)
Q 225,00>206,95 (+) 1,43e4

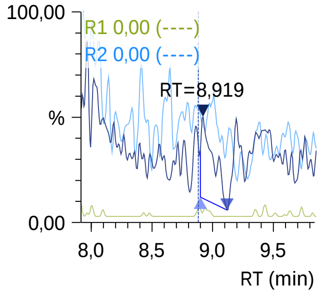

10 ppb002
Conc ----
Area ----
R#1 225,00>174,95 ---- (65,86)
R#2 225,00>90,95 ---- (87,91)
Q 225,00>206,95 (+) 1,36e4

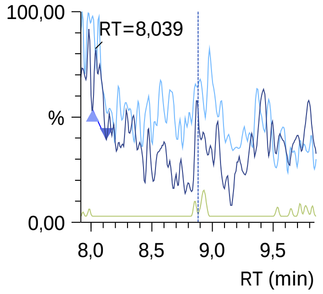

40 ppb001
Conc 43,3831
Area 51082
R#1 225,00>174,95 54,16 (65,86)
R#2 225,00>90,95 78,14 (87,91)
Q 225,00>206,95 (+) 1,52e4

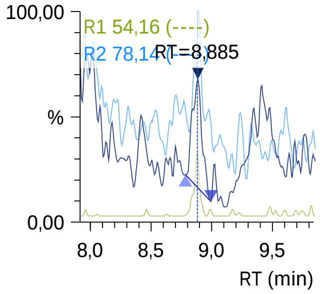

40ppb002
Conc 33,3305
Area 41341
R#1 225,00>174,95 39,25 (65,86)
R#2 225,00>90,95 50,32 (87,91)
Q 225,00>206,95 (+) 1,41e4

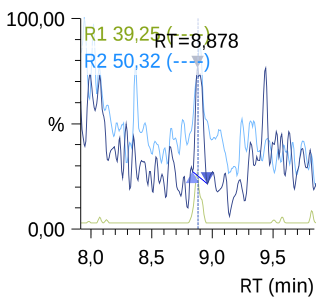

70 ppb001
Conc 45,3309
Area 52970
R#1 225,00>174,95 60,54 (65,86)
R#2 225,00>90,95 47,13 (87,91)
Q 225,00>206,95 (+) 2,09e4

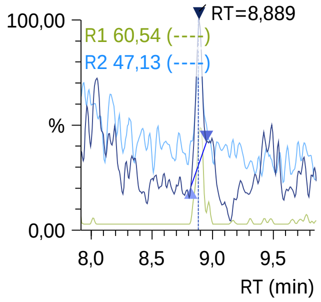

70ppb002
Conc 40,7437
Area 48525
R#1 225,00>174,95 70,87 (65,86)
R#2 225,00>90,95 125,88 (87,91)
Q 225,00>206,95 (+) 1,36e4

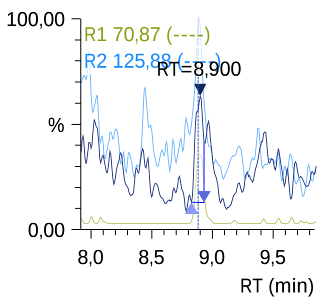

100 ppb001
Conc 61,5172
Area 68655
R#1 225,00>174,95 63,25 (65,86)
R#2 225,00>90,95 102,50 (87,91)
Q 225,00>206,95 (+) 2,75e4

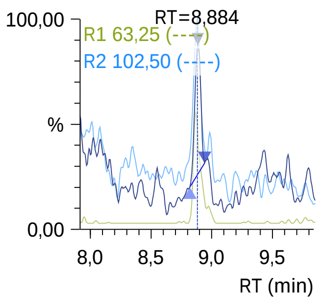

100ppb002
Conc 78,5605
Area 85170
R#1 225,00>174,95 51,80 (65,86)
R#2 225,00>90,95 70,97 (87,91)
Q 225,00>206,95 (+) 3,13e4

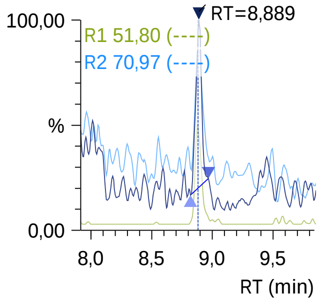

130 ppb001
Conc 119,9495
Area 125277
R#1 225,00>174,95 49,88 (65,86)
R#2 225,00>90,95 69,74 (87,91)
Q 225,00>206,95 (+) 2,95e4

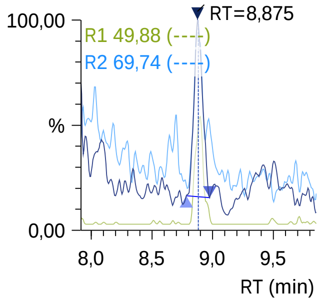

130ppb002
Conc 142,3540
Area 146988
R#1 225,00>174,95 34,31 (65,86)
R#2 225,00>90,95 75,15 (87,91)
Q 225,00>206,95 (+) 3,90e4

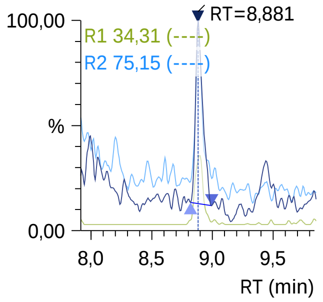

160 ppb001
Conc 186,6234
Area 189886
R#1 225,00>174,95 50,08 (65,86)
R#2 225,00>90,95 82,86 (87,91)
Q 225,00>206,95 (+) 4,92e4

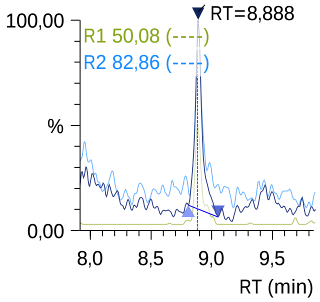

Compound: Ac. Sinapico (continued)

160ppb002
Conc 191,1139
Area 194237
R#1 225,00>174,95 53,30 (65,86)
R#2 225,00>90,95 80,20 (87,91)
Q 225,00>206,95 (+) 4,12e4

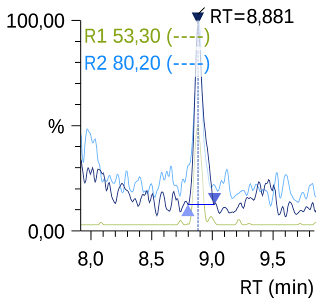

190 ppb001
Conc 174,2913
Area 177936
R#1 225,00>174,95 49,28 (65,86)
R#2 225,00>90,95 67,22 (87,91)
Q 225,00>206,95 (+) 5,23e4

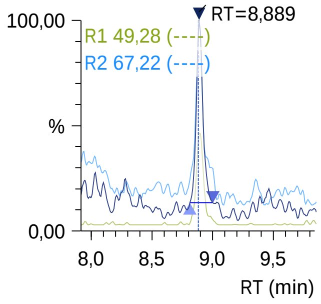

190ppb002
Conc 179,2000
Area 182692
R#1 225,00>174,95 56,43 (65,86)
R#2 225,00>90,95 84,29 (87,91)
Q 225,00>206,95 (+) 4,28e4

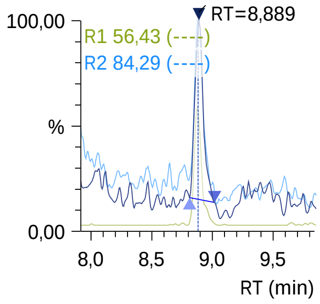

220 ppb001
Conc 220,3466
Area 222564
R#1 225,00>174,95 57,58 (65,86)
R#2 225,00>90,95 84,87 (87,91)
Q 225,00>206,95 (+) 5,88e4

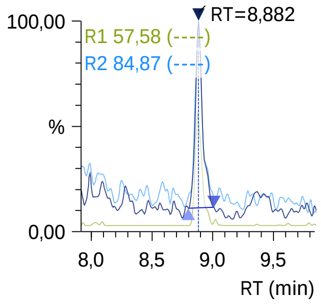

220ppb002
Conc 263,3348
Area 264221
R#1 225,00>174,95 52,12 (65,86)
R#2 225,00>90,95 65,79 (87,91)
Q 225,00>206,95 (+) 6,01e4

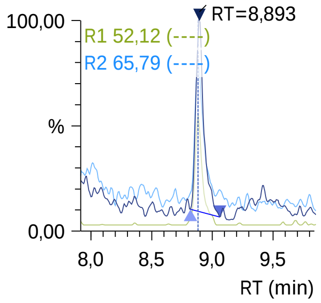

250 ppb001
Conc 264,8334
Area 265673
R#1 225,00>174,95 47,58 (65,86)
R#2 225,00>90,95 80,57 (87,91)
Q 225,00>206,95 (+) 6,69e4

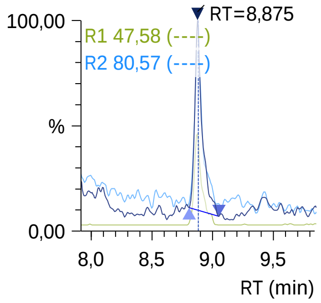

250 ppb002
Conc 215,9522
Area 218306
R#1 225,00>174,95 65,86 (65,86)
R#2 225,00>90,95 87,91 (87,91)
Q 225,00>206,95 (+) 6,63e4

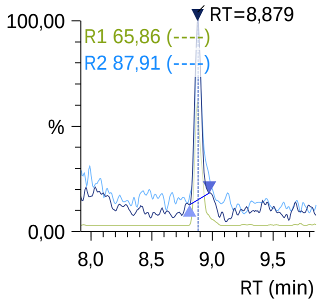

10H
Conc ----
Area ----
R#1 225,00>174,95 ---- (65,86)
R#2 225,00>90,95 ---- (87,91)
Q 225,00>206,95 (+) 4,26e3

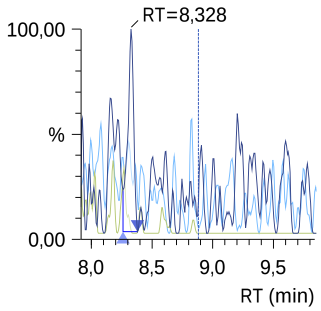

10M
Conc ----
Area ----
R#1 225,00>174,95 ---- (65,86)
R#2 225,00>90,95 ---- (87,91)
Q 225,00>206,95 (+) 3,92e3

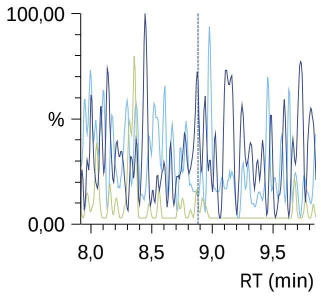

9H
Conc ----
Area ----
R#1 225,00>174,95 ---- (65,86)
R#2 225,00>90,95 ---- (87,91)
Q 225,00>206,95 (+) 6,52e3

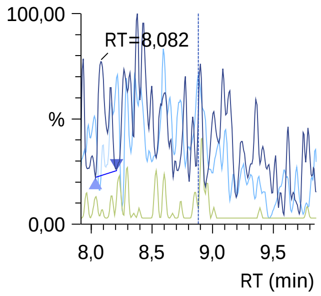

9M
Conc ----
Area ----
R#1 225,00>174,95 ---- (65,86)
R#2 225,00>90,95 ---- (87,91)
Q 225,00>206,95 (+) 7,24e3

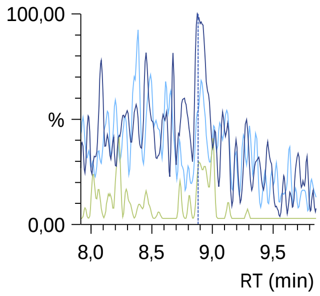

Compound: Seringaldazina

Curve Fit: Linear | Weighting: None | Zero: Not Forced
Quantitative Method: External Standard
Q 361,00>182,00
R^2 = 0.9870559 R = 0.9935069
y = 2081,640x + 15378,56

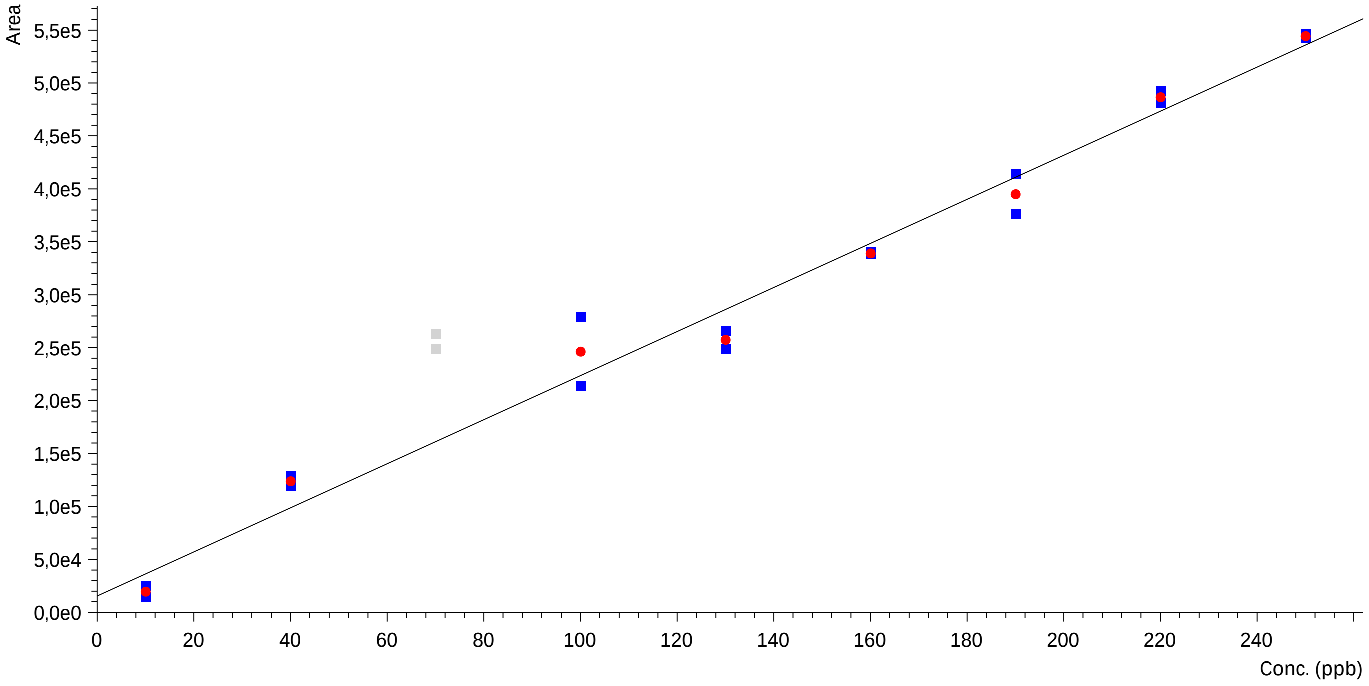

Sample ID	Area	Conc.	Sample Type	Level	Cal Point	Found RT	Ref 1 Actual Ratio
0	----	----	Ctrl.	0	----	----	----
10ppb001	14556	-0,3950	Std.	1	Yes	9,844	113,97
10 ppb002	24717	4,4862	Std.	1	Yes	9,868	61,06
40 ppb001	119500	50,0187	Std.	2	Yes	9,848	70,99
40ppb002	128609	54,3946	Std.	2	Yes	9,851	62,28
70 ppb001	263345	119,1207	Std.	3	No	9,848	81,34
70ppb002	249213	112,3319	Std.	3	No	9,861	67,61
100 ppb001	278571	126,4352	Std.	4	Yes	9,848	60,16
100ppb002	214304	95,5620	Std.	4	Yes	9,844	62,24
130 ppb001	249224	112,3371	Std.	5	Yes	9,849	65,14
130ppb002	265820	120,3099	Std.	5	Yes	9,848	68,25
160 ppb001	338188	155,0744	Std.	6	Yes	9,847	62,70
160ppb002	340385	156,1302	Std.	6	Yes	9,842	70,71
190 ppb001	413845	191,4194	Std.	7	Yes	9,851	53,97
190ppb002	376168	173,3198	Std.	7	Yes	9,849	69,64
220 ppb001	480821	223,5944	Std.	8	Yes	9,838	74,25
220ppb002	492314	229,1152	Std.	8	Yes	9,851	69,14
250 ppb001	546266	255,0332	Std.	9	Yes	9,840	62,13
250 ppb002	542376	253,1644	Std.	9	Yes	9,848	63,82
10H	----	----	Unk.	----	----	----	----
10M	----	----	Unk.	----	----	----	----
9H	----	----	Unk.	----	----	----	----
9M	----	----	Unk.	----	----	----	----
Average	296568	135,0807				9,849	68,85
%RSD	55	57,5641				0,070	18,63
Maximum	546266	255,0332				9,868	113,97
Minimum	14556	-0,3950				9,838	53,97
Std. Dev.	161864	77,7580				0,007	12,83

Compound: Seringaldazina (continued)

0

Conc ----
Area ----
R#1 361,00>179,90 ---- (62,24)
R#2 361,00>139,95 ---- (46,02)
Q 361,00>182,00 (+) 2,77e2

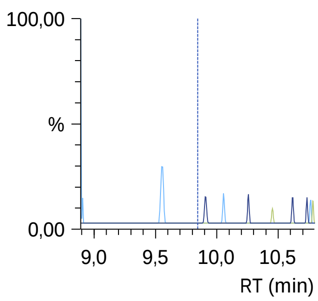

10ppb001

Conc -0,3950
Area 14556
R#1 361,00>179,90 113,97 (62,24)
R#2 361,00>139,95 79,50 (46,02)
Q 361,00>182,00 (+) 2,68e3

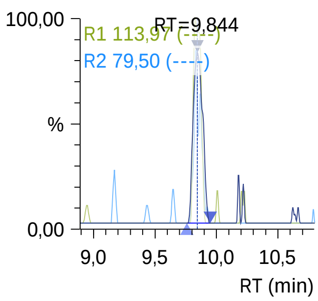

10 ppb002

Conc 4,4862
Area 24717
R#1 361,00>179,90 61,06 (62,24)
R#2 361,00>139,95 73,24 (46,02)
Q 361,00>182,00 (+) 5,07e3

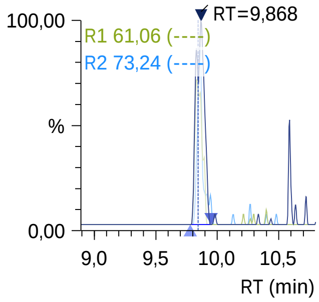

40 ppb001

Conc 50,0187
Area 119500
R#1 361,00>179,90 70,99 (62,24)
R#2 361,00>139,95 47,48 (46,02)
Q 361,00>182,00 (+) 2,44e4

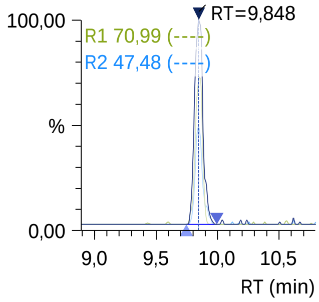

40ppb002

Conc 54,3946
Area 128609
R#1 361,00>179,90 62,28 (62,24)
R#2 361,00>139,95 56,51 (46,02)
Q 361,00>182,00 (+) 3,09e4

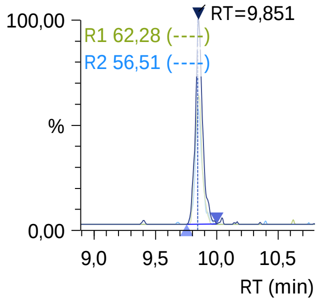

70 ppb001

Conc 119,1207
Area 263345
R#1 361,00>179,90 81,34 (62,24)
R#2 361,00>139,95 44,70 (46,02)
Q 361,00>182,00 (+) 5,53e4

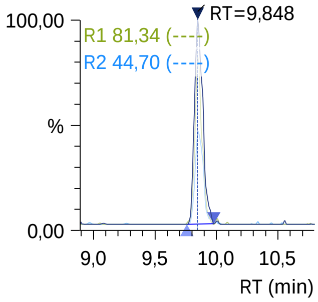

70ppb002

Conc 112,3319
Area 249213
R#1 361,00>179,90 67,61 (62,24)
R#2 361,00>139,95 43,45 (46,02)
Q 361,00>182,00 (+) 5,65e4

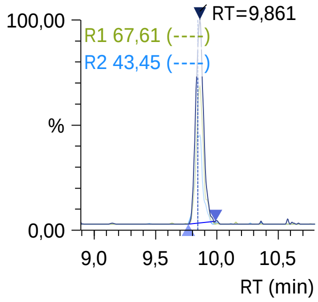

100 ppb001

Conc 126,4352
Area 278571
R#1 361,00>179,90 60,16 (62,24)
R#2 361,00>139,95 37,04 (46,02)
Q 361,00>182,00 (+) 7,04e4

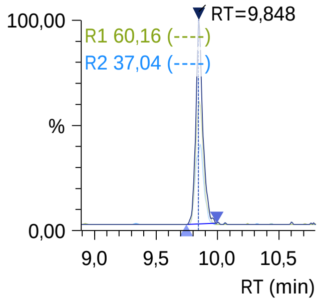

100ppb002

Conc 95,5620
Area 214304
R#1 361,00>179,90 62,24 (62,24)
R#2 361,00>139,95 46,02 (46,02)
Q 361,00>182,00 (+) 4,81e4

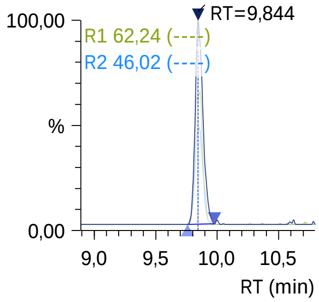

130 ppb001

Conc 112,3371
Area 249224
R#1 361,00>179,90 65,14 (62,24)
R#2 361,00>139,95 52,11 (46,02)
Q 361,00>182,00 (+) 5,58e4

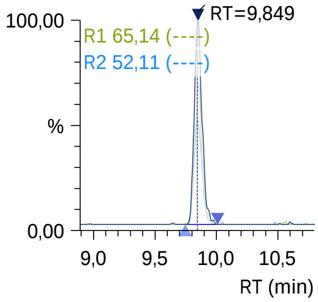

130ppb002

Conc 120,3099
Area 265820
R#1 361,00>179,90 68,25 (62,24)
R#2 361,00>139,95 49,04 (46,02)
Q 361,00>182,00 (+) 5,90e4

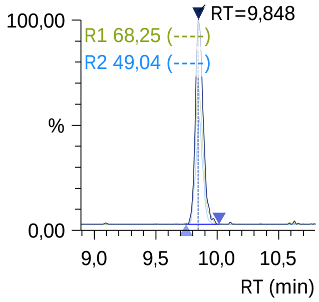

160 ppb001

Conc 155,0744
Area 338188
R#1 361,00>179,90 62,70 (62,24)
R#2 361,00>139,95 44,67 (46,02)
Q 361,00>182,00 (+) 8,21e4

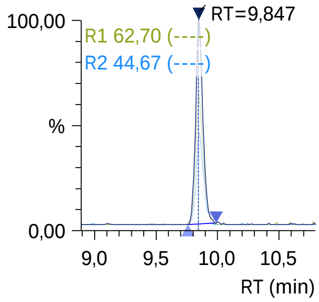

Compound: Seringaldazina (continued)

160ppb002
Conc 156,1302
Area 340385
R#1 361,00>179,90 70,71 (62,24)
R#2 361,00>139,95 44,44 (46,02)
Q 361,00>182,00 (+) 8,08e4

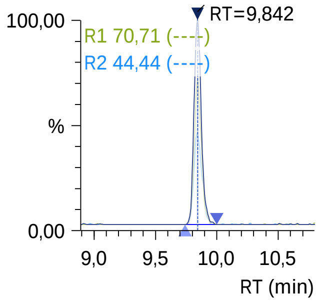

190 ppb001
Conc 191,4194
Area 413845
R#1 361,00>179,90 53,97 (62,24)
R#2 361,00>139,95 45,87 (46,02)
Q 361,00>182,00 (+) 9,66e4

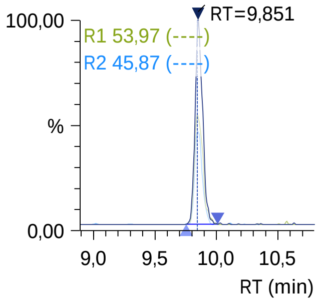

190ppb002
Conc 173,3198
Area 376168
R#1 361,00>179,90 69,64 (62,24)
R#2 361,00>139,95 42,74 (46,02)
Q 361,00>182,00 (+) 9,35e4

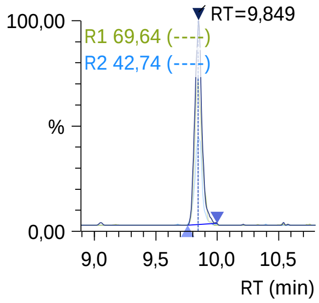

220 ppb001
Conc 223,5944
Area 480821
R#1 361,00>179,90 74,25 (62,24)
R#2 361,00>139,95 53,06 (46,02)
Q 361,00>182,00 (+) 1,03e5

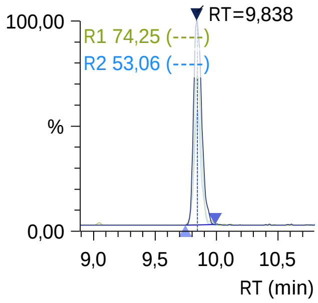

220ppb002
Conc 229,1152
Area 492314
R#1 361,00>179,90 69,14 (62,24)
R#2 361,00>139,95 45,74 (46,02)
Q 361,00>182,00 (+) 1,15e5

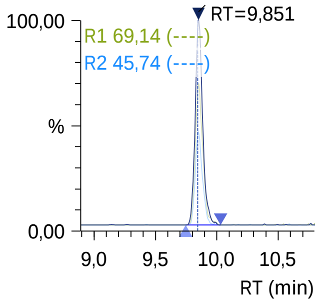

250 ppb001
Conc 255,0332
Area 546266
R#1 361,00>179,90 62,13 (62,24)
R#2 361,00>139,95 40,31 (46,02)
Q 361,00>182,00 (+) 1,25e5

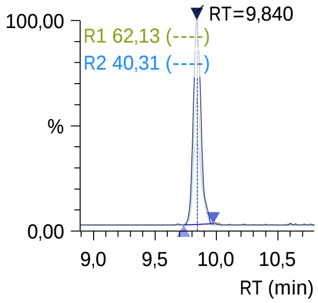

250 ppb002
Conc 253,1644
Area 542376
R#1 361,00>179,90 63,82 (62,24)
R#2 361,00>139,95 45,52 (46,02)
Q 361,00>182,00 (+) 1,20e5

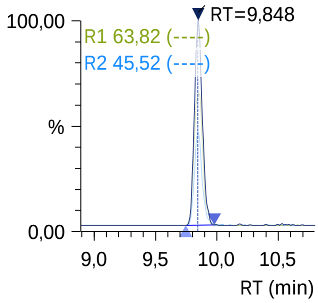

10H
Conc ----
Area ----
R#1 361,00>179,90 ---- (62,24)
R#2 361,00>139,95 ---- (46,02)
Q 361,00>182,00 (+) 2,41e2

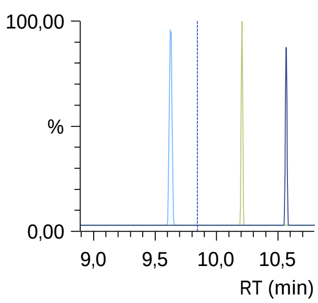

10M
Conc ----
Area ----
R#1 361,00>179,90 ---- (62,24)
R#2 361,00>139,95 ---- (46,02)
Q 361,00>182,00 (+) 2,77e2

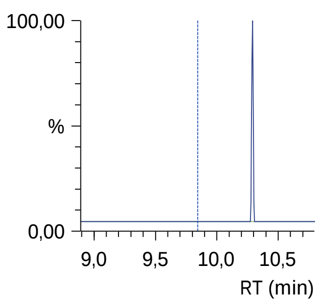

9H
Conc ----
Area ----
R#1 361,00>179,90 ---- (62,24)
R#2 361,00>139,95 ---- (46,02)
Q 361,00>182,00 (+) 1,52e2

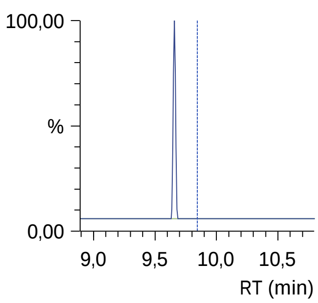

9M
Conc ----
Area ----
R#1 361,00>179,90 ---- (62,24)
R#2 361,00>139,95 ---- (46,02)
Q 361,00>182,00 (+) 0,00e0

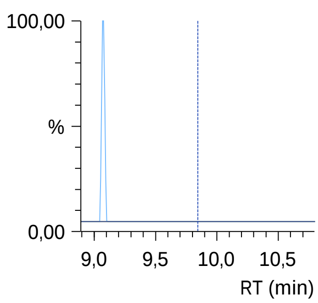

Compound: Catequina

Curve Fit: Linear | Weighting: None | Zero: Not Forced
Quantitative Method: External Standard
Q 290,80>138,90
R^2 = 0.9951241 R = 0.9975591
y = 2644,606x - 25730,99

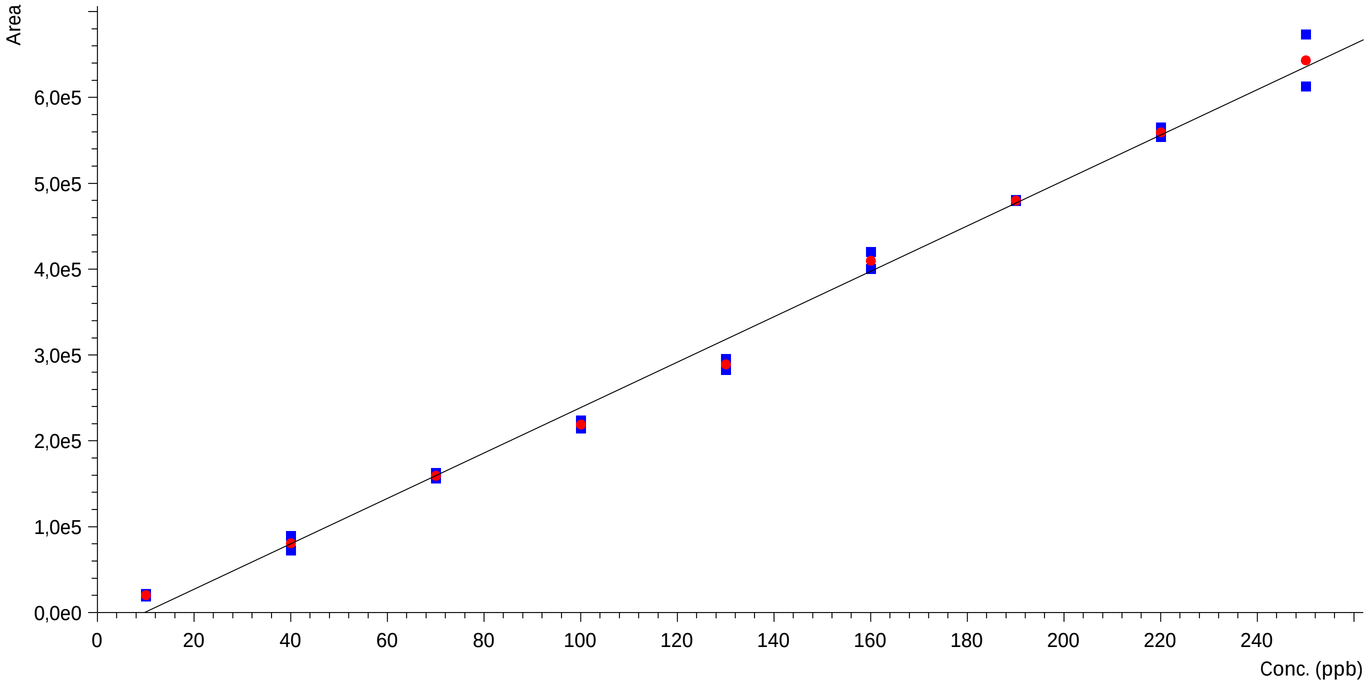

Sample ID	Area	Conc.	Sample Type	Level	Cal Point	Found RT	Ref 1 Actual Ratio
0	----	----	Ctrl.	0	----	----	----
10ppb001	21921	18,0185	Std.	1	Yes	7,657	41,05
10 ppb002	18758	16,8227	Std.	1	Yes	8,025	60,43
40 ppb001	72293	37,0655	Std.	2	Yes	7,645	50,62
40ppb002	89391	43,5307	Std.	2	Yes	8,034	45,81
70 ppb001	162702	71,2518	Std.	3	Yes	7,654	44,89
70ppb002	156750	69,0011	Std.	3	Yes	7,654	55,94
100 ppb001	223965	94,4170	Std.	4	Yes	7,640	48,90
100ppb002	214270	90,7513	Std.	4	Yes	8,040	67,67
130 ppb001	295622	121,5125	Std.	5	Yes	7,638	62,06
130ppb002	282903	116,7032	Std.	5	Yes	7,647	48,24
160 ppb001	400214	161,0617	Std.	6	Yes	7,646	58,88
160ppb002	420155	168,6020	Std.	6	Yes	7,642	58,21
190 ppb001	480656	191,4791	Std.	7	Yes	7,647	58,78
190ppb002	479735	191,1309	Std.	7	Yes	7,637	51,56
220 ppb001	553969	219,2007	Std.	8	Yes	7,653	66,00
220ppb002	565244	223,4644	Std.	8	Yes	7,651	55,43
250 ppb001	613020	241,5298	Std.	9	Yes	7,642	60,72
250 ppb002	673655	264,4573	Std.	9	Yes	7,648	53,94
10H	----	----	Unk.	----	----	----	----
10M	----	----	Unk.	----	----	----	----
9H	----	----	Unk.	----	----	----	----
9M	----	----	Unk.	----	----	----	----
Average	318068	130,0000				7,711	54,95
%RSD	67	61,5588				1,922	13,46
Maximum	673655	264,4573				8,040	67,67
Minimum	18758	16,8227				7,637	41,05
Std. Dev.	211639	80,0265				0,148	7,40

Compound: Catequina (continued)

0
Conc ----
Area ----
R#1 290,80>122,90 ---- (58,16)
R#2 290,80>146,90 ---- (28,77)
Q 290,80>138,90 (+) 2,67e2

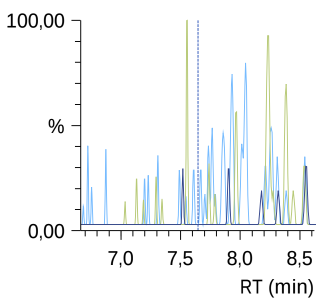

10ppb001
Conc 18,0185
Area 21921
R#1 290,80>122,90 41,05 (58,16)
R#2 290,80>146,90 23,63 (28,77)
Q 290,80>138,90 (+) 6,38e3

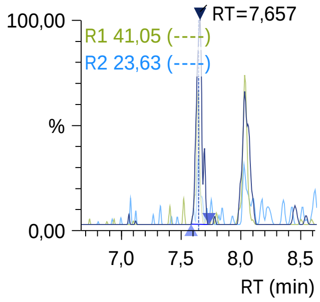

10 ppb002
Conc 16,8227
Area 18758
R#1 290,80>122,90 60,43 (58,16)
R#2 290,80>146,90 0,00 (28,77)
Q 290,80>138,90 (+) 5,90e3

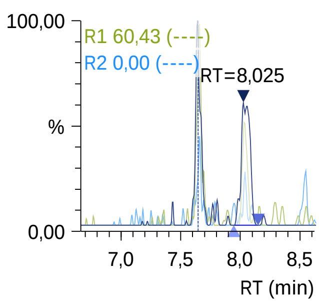

40 ppb001
Conc 37,0655
Area 72293
R#1 290,80>122,90 50,62 (58,16)
R#2 290,80>146,90 18,71 (28,77)
Q 290,80>138,90 (+) 2,16e4

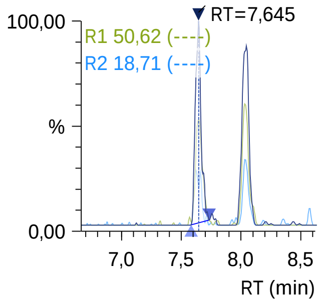

40ppb002
Conc 43,5307
Area 89391
R#1 290,80>122,90 45,81 (58,16)
R#2 290,80>146,90 15,78 (28,77)
Q 290,80>138,90 (+) 2,47e4

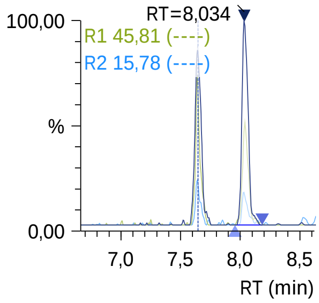

70 ppb001
Conc 71,2518
Area 162702
R#1 290,80>122,90 44,89 (58,16)
R#2 290,80>146,90 18,52 (28,77)
Q 290,80>138,90 (+) 4,43e4

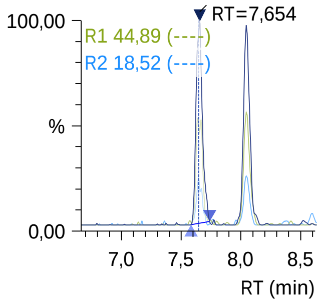

70ppb002
Conc 69,0011
Area 156750
R#1 290,80>122,90 55,94 (58,16)
R#2 290,80>146,90 26,30 (28,77)
Q 290,80>138,90 (+) 4,54e4

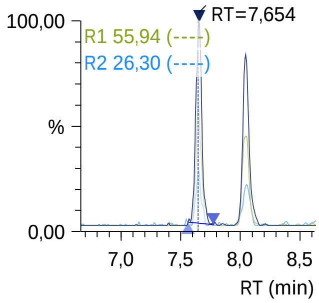

100 ppb001
Conc 94,4170
Area 223965
R#1 290,80>122,90 48,90 (58,16)
R#2 290,80>146,90 21,97 (28,77)
Q 290,80>138,90 (+) 6,41e4

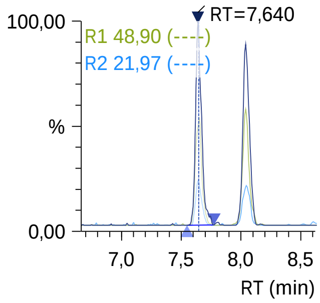

100ppb002
Conc 90,7513
Area 214270
R#1 290,80>122,90 67,67 (58,16)
R#2 290,80>146,90 20,93 (28,77)
Q 290,80>138,90 (+) 5,77e4

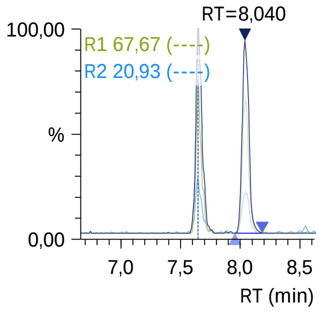

130 ppb001
Conc 121,5125
Area 295622
R#1 290,80>122,90 62,06 (58,16)
R#2 290,80>146,90 27,79 (28,77)
Q 290,80>138,90 (+) 8,14e4

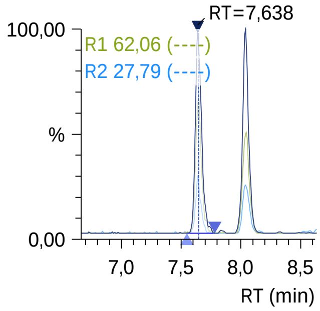

130ppb002
Conc 116,7032
Area 282903
R#1 290,80>122,90 48,24 (58,16)
R#2 290,80>146,90 21,17 (28,77)
Q 290,80>138,90 (+) 9,03e4

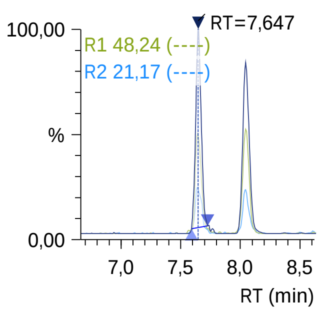

160 ppb001
Conc 161,0617
Area 400214
R#1 290,80>122,90 58,88 (58,16)
R#2 290,80>146,90 25,37 (28,77)
Q 290,80>138,90 (+) 1,09e5

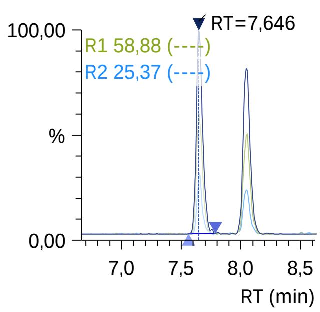

Compound: Catequina (continued)

160ppb002
Conc 168,6020
Area 420155
R#1 290,80>122,90 58,21 (58,16)
R#2 290,80>146,90 28,76 (28,77)
Q 290,80>138,90 (+) 1,19e5

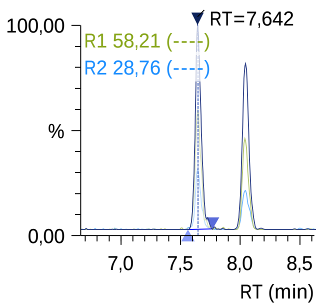

190 ppb001
Conc 191,4791
Area 480656
R#1 290,80>122,90 58,78 (58,16)
R#2 290,80>146,90 27,14 (28,77)
Q 290,80>138,90 (+) 1,25e5

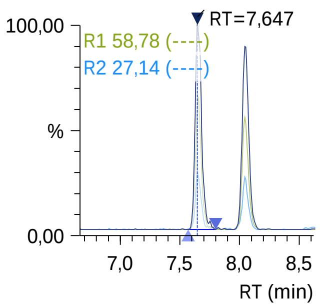

190ppb002
Conc 191,1309
Area 479735
R#1 290,80>122,90 51,56 (58,16)
R#2 290,80>146,90 23,55 (28,77)
Q 290,80>138,90 (+) 1,31e5

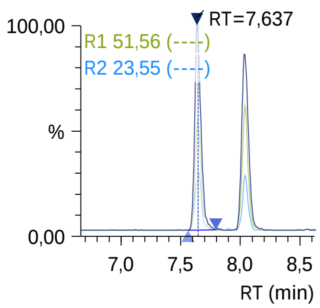

220 ppb001
Conc 219,2007
Area 553969
R#1 290,80>122,90 66,00 (58,16)
R#2 290,80>146,90 23,54 (28,77)
Q 290,80>138,90 (+) 1,46e5

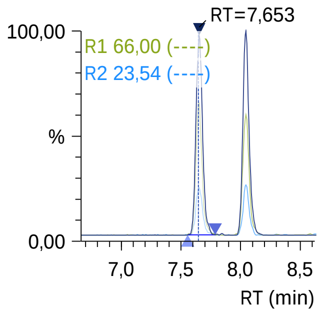

220ppb002
Conc 223,4644
Area 565244
R#1 290,80>122,90 55,43 (58,16)
R#2 290,80>146,90 23,48 (28,77)
Q 290,80>138,90 (+) 1,61e5

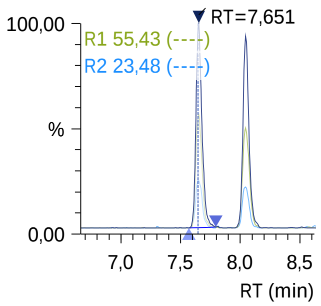

250 ppb001
Conc 241,5298
Area 613020
R#1 290,80>122,90 60,72 (58,16)
R#2 290,80>146,90 26,67 (28,77)
Q 290,80>138,90 (+) 1,76e5

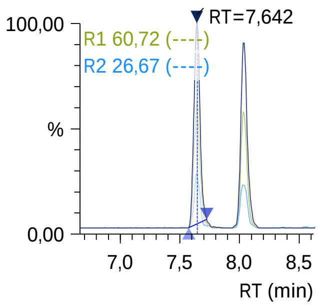

250 ppb002
Conc 264,4573
Area 673655
R#1 290,80>122,90 53,94 (58,16)
R#2 290,80>146,90 27,34 (28,77)
Q 290,80>138,90 (+) 1,88e5

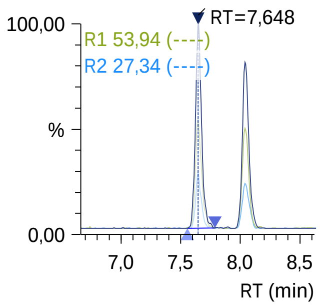

10H
Conc ----
Area ----
R#1 290,80>122,90 ---- (58,16)
R#2 290,80>146,90 ---- (28,77)
Q 290,80>138,90 (+) 1,91e3

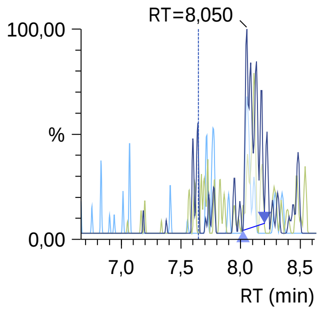

10M
Conc ----
Area ----
R#1 290,80>122,90 ---- (58,16)
R#2 290,80>146,90 ---- (28,77)
Q 290,80>138,90 (+) 3,93e3

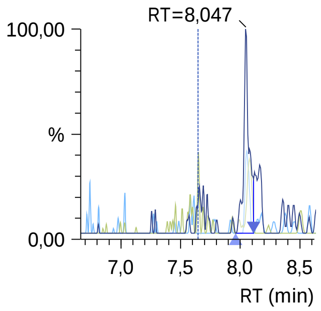

9H
Conc ----
Area ----
R#1 290,80>122,90 ---- (58,16)
R#2 290,80>146,90 ---- (28,77)
Q 290,80>138,90 (+) 2,94e3

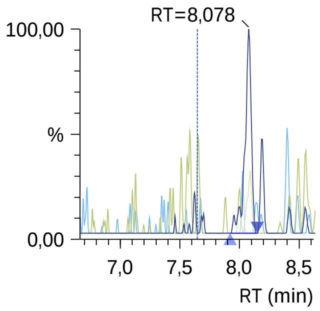

9M
Conc ----
Area ----
R#1 290,80>122,90 ---- (58,16)
R#2 290,80>146,90 ---- (28,77)
Q 290,80>138,90 (+) 1,71e3

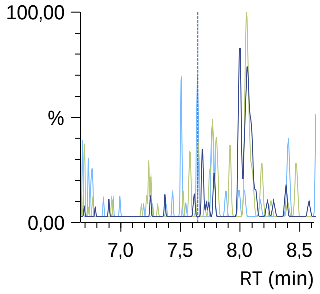

Compound: Sinapaldeido

Curve Fit: Linear | Weighting: None | Zero: Not Forced
Quantitative Method: External Standard
Q 209,00>54,95
R^2 = 0.9959511 R = 0.9979735
y = 1583,239x + 5070,180

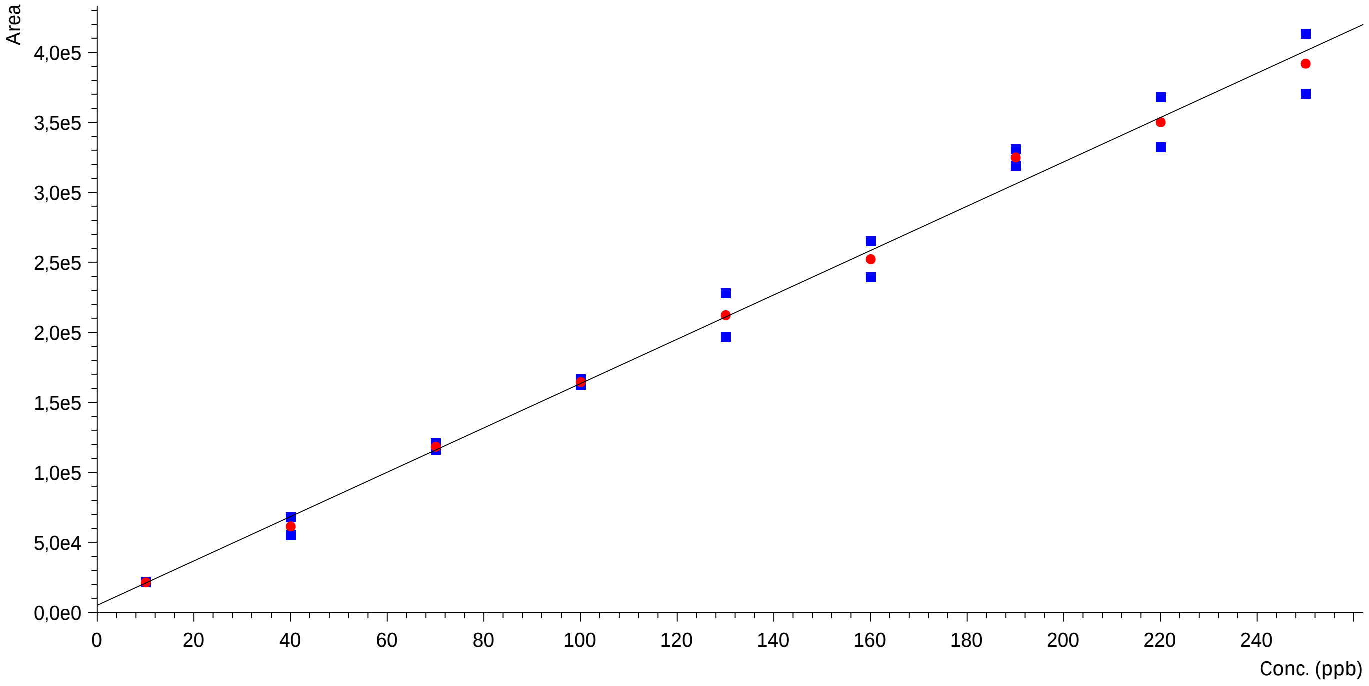

Sample ID	Area	Conc.	Sample Type	Level	Cal Point	Found RT	Ref 1 Actual Ratio
0	----	----	Ctrl.	0	----	----	----
10ppb001	21383	10,3037	Std.	1	Yes	9,045	27,79
10 ppb002	----	----	Std.	1	No	----	----
40 ppb001	55032	31,5564	Std.	2	Yes	9,025	40,31
40ppb002	68102	39,8117	Std.	2	Yes	9,038	10,74
70 ppb001	116112	70,1358	Std.	3	Yes	9,046	33,26
70ppb002	120695	73,0303	Std.	3	Yes	9,052	45,28
100 ppb001	166549	101,9928	Std.	4	Yes	9,040	39,59
100ppb002	162724	99,5765	Std.	4	Yes	9,048	35,32
130 ppb001	227940	140,7683	Std.	5	Yes	9,043	48,72
130ppb002	196732	121,0568	Std.	5	Yes	9,041	42,69
160 ppb001	239390	148,0002	Std.	6	Yes	9,048	37,27
160ppb002	265169	164,2824	Std.	6	Yes	9,042	49,53
190 ppb001	319088	198,3389	Std.	7	Yes	9,056	47,65
190ppb002	331086	205,9170	Std.	7	Yes	9,038	40,86
220 ppb001	368069	229,2759	Std.	8	Yes	9,043	41,07
220ppb002	332460	206,7846	Std.	8	Yes	9,048	42,96
250 ppb001	413568	258,0142	Std.	9	Yes	9,037	44,64
250 ppb002	370562	230,8508	Std.	9	Yes	9,042	51,25
10H	----	----	Unk.	----	----	----	----
10M	----	----	Unk.	----	----	----	----
9H	----	----	Unk.	----	----	----	----
9M	----	----	Unk.	----	----	----	----
Average	222039	137,0410				9,043	39,94
%RSD	55	56,0166				0,075	24,24
Maximum	413568	258,0142				9,056	51,25
Minimum	21383	10,3037				9,025	10,74
Std. Dev.	121539	76,7657				0,007	9,68

Compound: Sinapaldeido (continued)

0
Conc ----
Area ----
R#1 209,00>176,95 ---- (49,53)
R#2 209,00>91,00 ---- (84,86)
Q 209,00>54,95 (+) 1,09e5

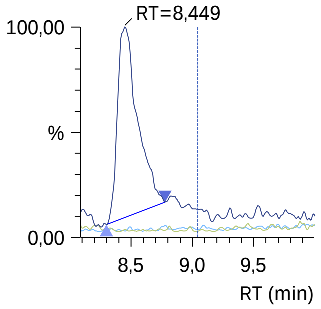

10ppb001
Conc 10,3037
Area 21383
R#1 209,00>176,95 27,79 (49,53)
R#2 209,00>91,00 31,40 (84,86)
Q 209,00>54,95 (+) 1,30e5

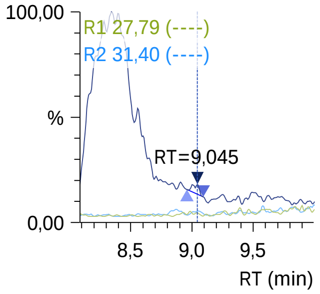

10 ppb002
Conc ----
Area ----
R#1 209,00>176,95 ---- (49,53)
R#2 209,00>91,00 ---- (84,86)
Q 209,00>54,95 (+) 1,42e5

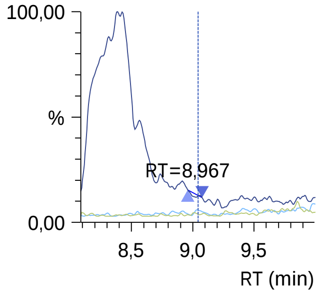

40 ppb001
Conc 31,5564
Area 55032
R#1 209,00>176,95 40,31 (49,53)
R#2 209,00>91,00 69,26 (84,86)
Q 209,00>54,95 (+) 1,32e5

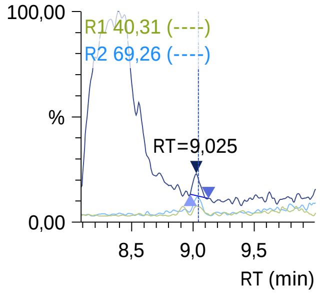

40ppb002
Conc 39,8117
Area 68102
R#1 209,00>176,95 10,74 (49,53)
R#2 209,00>91,00 46,75 (84,86)
Q 209,00>54,95 (+) 1,39e5

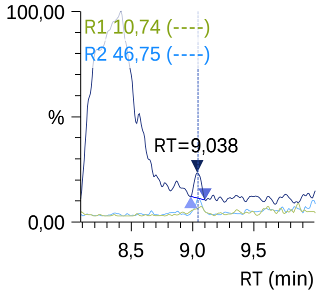

70 ppb001
Conc 70,1358
Area 116112
R#1 209,00>176,95 33,26 (49,53)
R#2 209,00>91,00 65,36 (84,86)
Q 209,00>54,95 (+) 1,47e5

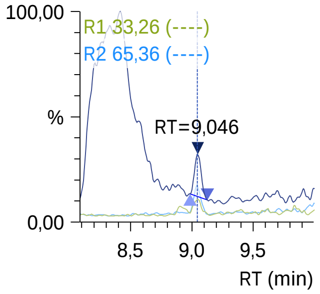

70ppb002
Conc 73,0303
Area 120695
R#1 209,00>176,95 45,28 (49,53)
R#2 209,00>91,00 84,65 (84,86)
Q 209,00>54,95 (+) 1,39e5

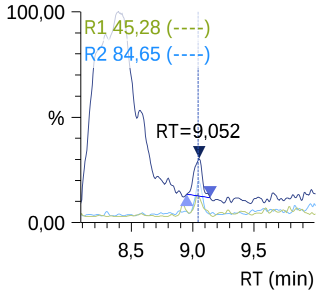

100 ppb001
Conc 101,9928
Area 166549
R#1 209,00>176,95 39,59 (49,53)
R#2 209,00>91,00 86,21 (84,86)
Q 209,00>54,95 (+) 1,50e5

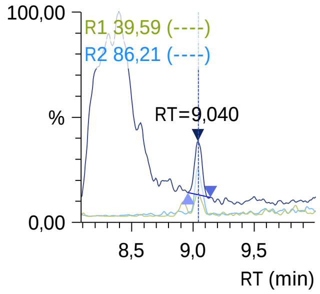

100ppb002
Conc 99,5765
Area 162724
R#1 209,00>176,95 35,32 (49,53)
R#2 209,00>91,00 75,34 (84,86)
Q 209,00>54,95 (+) 1,44e5

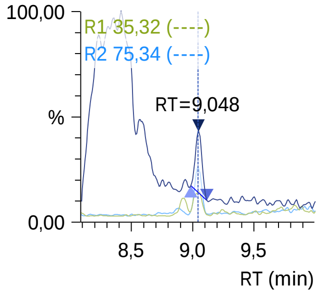

130 ppb001
Conc 140,7683
Area 227940
R#1 209,00>176,95 48,72 (49,53)
R#2 209,00>91,00 87,52 (84,86)
Q 209,00>54,95 (+) 1,54e5

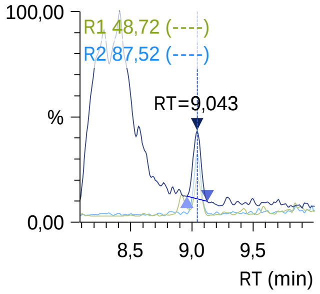

130ppb002
Conc 121,0568
Area 196732
R#1 209,00>176,95 42,69 (49,53)
R#2 209,00>91,00 87,94 (84,86)
Q 209,00>54,95 (+) 1,43e5

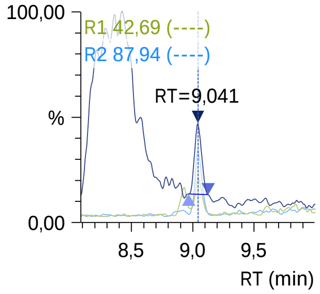

160 ppb001
Conc 148,0002
Area 239390
R#1 209,00>176,95 37,27 (49,53)
R#2 209,00>91,00 72,01 (84,86)
Q 209,00>54,95 (+) 1,43e5

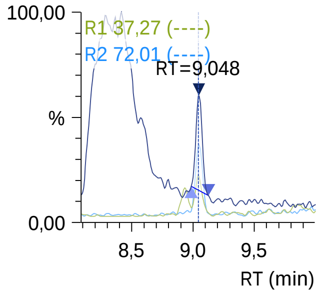

Compound: Sinapaldeido (continued)

160ppb002
Conc 164,2824
Area 265169
R#1 209,00>176,95 49,53 (49,53)
R#2 209,00>91,00 84,86 (84,86)
Q 209,00>54,95 (+) 1,40e5

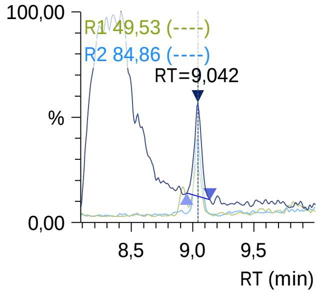

190 ppb001
Conc 198,3389
Area 319088
R#1 209,00>176,95 47,65 (49,53)
R#2 209,00>91,00 74,06 (84,86)
Q 209,00>54,95 (+) 1,43e5

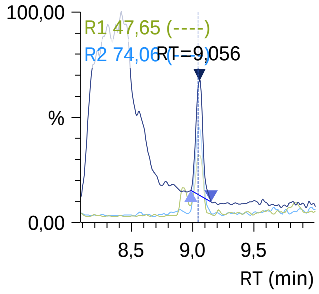

190ppb002
Conc 205,9170
Area 331086
R#1 209,00>176,95 40,86 (49,53)
R#2 209,00>91,00 79,85 (84,86)
Q 209,00>54,95 (+) 1,44e5

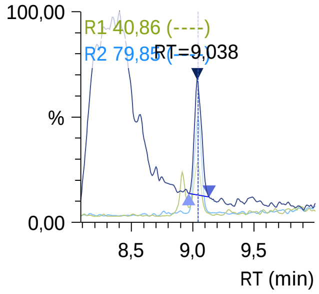

220 ppb001
Conc 229,2759
Area 368069
R#1 209,00>176,95 41,07 (49,53)
R#2 209,00>91,00 76,01 (84,86)
Q 209,00>54,95 (+) 1,42e5

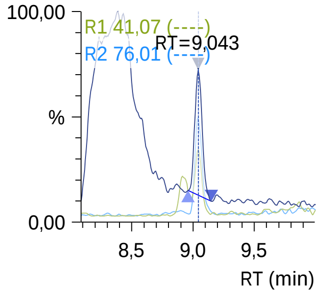

220ppb002
Conc 206,7846
Area 332460
R#1 209,00>176,95 42,96 (49,53)
R#2 209,00>91,00 71,56 (84,86)
Q 209,00>54,95 (+) 1,44e5

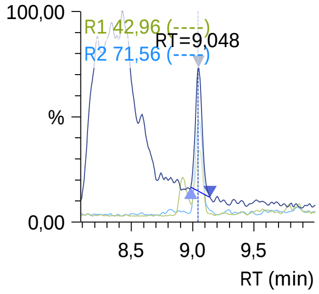

250 ppb001
Conc 258,0142
Area 413568
R#1 209,00>176,95 44,64 (49,53)
R#2 209,00>91,00 83,49 (84,86)
Q 209,00>54,95 (+) 1,44e5

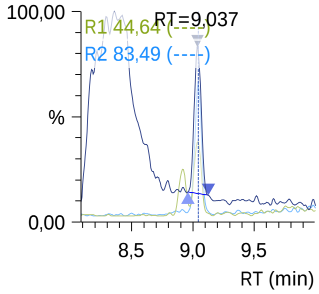

250 ppb002
Conc 230,8508
Area 370562
R#1 209,00>176,95 51,25 (49,53)
R#2 209,00>91,00 81,57 (84,86)
Q 209,00>54,95 (+) 1,42e5

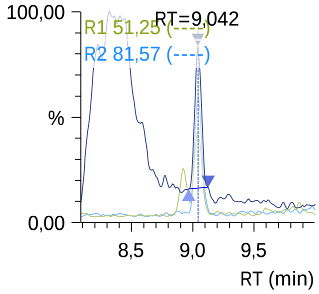

10H
Conc ----
Area ----
R#1 209,00>176,95 ---- (49,53)
R#2 209,00>91,00 ---- (84,86)
Q 209,00>54,95 (+) 1,22e4

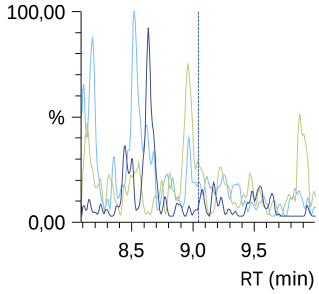

10M
Conc ----
Area ----
R#1 209,00>176,95 ---- (49,53)
R#2 209,00>91,00 ---- (84,86)
Q 209,00>54,95 (+) 1,43e4

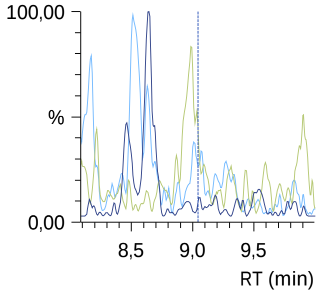

9H
Conc ----
Area ----
R#1 209,00>176,95 ---- (49,53)
R#2 209,00>91,00 ---- (84,86)
Q 209,00>54,95 (+) 5,67e4

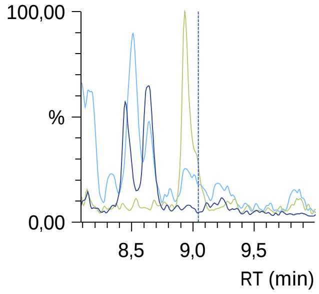

9M
Conc ----
Area ----
R#1 209,00>176,95 ---- (49,53)
R#2 209,00>91,00 ---- (84,86)
Q 209,00>54,95 (+) 7,24e4

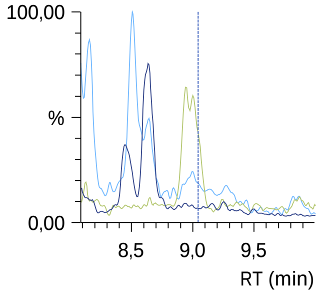

Compound: Luteolin

Curve Fit: Linear | Weighting: None | Zero: Not Forced
Quantitative Method: External Standard
Q 286,90>152,85
R^2 = 0.9701065 R = 0.9849398
y = 1880,476x + 69297,92

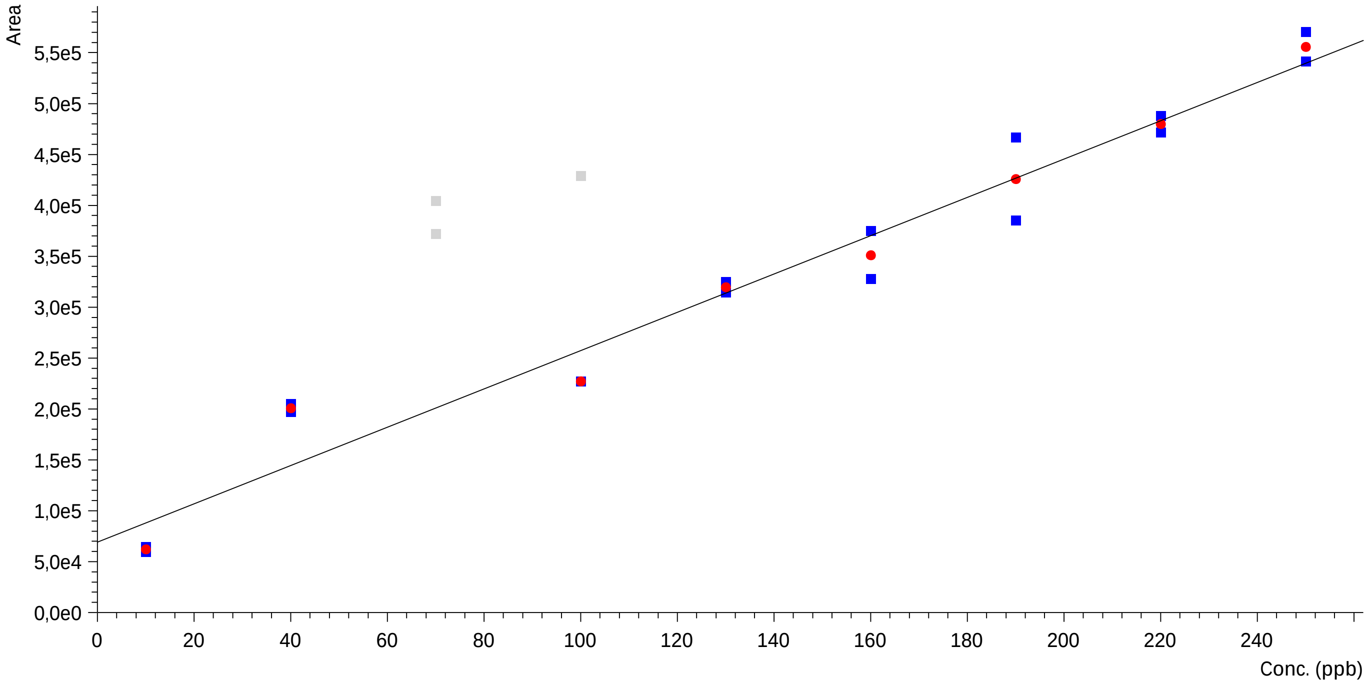

Sample ID	Area	Conc.	Sample Type	Level	Cal Point	Found RT	Ref 1 Actual Ratio
0	10817	-31,0992	Ctrl.	0	----	9,949	17,64
10ppb001	64746	-2,4204	Std.	1	Yes	9,902	20,54
10 ppb002	59781	-5,0608	Std.	1	Yes	9,903	32,44
40 ppb001	197037	67,9292	Std.	2	Yes	9,879	34,67
40ppb002	204980	72,1528	Std.	2	Yes	9,888	37,16
70 ppb001	404250	178,1208	Std.	3	No	9,895	31,03
70ppb002	372357	161,1611	Std.	3	No	9,895	26,17
100 ppb001	429000	191,2826	Std.	4	No	9,891	25,60
100ppb002	227243	83,9920	Std.	4	Yes	9,890	32,01
130 ppb001	314488	130,3873	Std.	5	Yes	9,904	27,36
130ppb002	324714	135,8255	Std.	5	Yes	9,892	30,05
160 ppb001	327438	137,2738	Std.	6	Yes	9,897	26,49
160ppb002	374711	162,4125	Std.	6	Yes	9,890	35,22
190 ppb001	466836	211,4030	Std.	7	Yes	9,906	22,53
190ppb002	385155	167,9666	Std.	7	Yes	9,893	32,39
220 ppb001	487801	222,5519	Std.	8	Yes	9,882	41,41
220ppb002	472009	214,1539	Std.	8	Yes	9,896	30,95
250 ppb001	570218	266,3796	Std.	9	Yes	9,883	30,84
250 ppb002	541412	251,0610	Std.	9	Yes	9,891	31,45
10H	----	----	Unk.	----	----	----	----
10M	----	----	Unk.	----	----	----	----
9H	----	----	Unk.	----	----	----	----
9M	----	----	Unk.	----	----	----	----
Average	328158	137,6565				9,896	29,79
%RSD	50	62,9312				0,149	19,40
Maximum	570218	266,3796				9,949	41,41
Minimum	10817	-31,0992				9,879	17,64
Std. Dev.	162903	86,6289				0,015	5,78

Compound: Luteolin (continued)

0
Conc -31,0992
Area 10817
R#1 286,90>134,90 17,64 (41,41)
R#2 286,90>88,90 0,00 (26,41)
Q 286,90>152,85 (+) 1,96e3

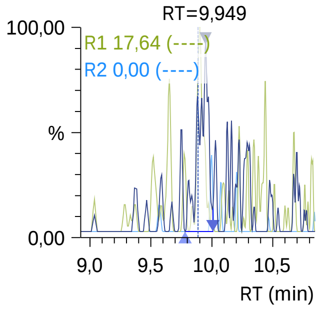

10ppb001
Conc -2,4204
Area 64746
R#1 286,90>134,90 20,54 (41,41)
R#2 286,90>88,90 23,21 (26,41)
Q 286,90>152,85 (+) 1,17e4

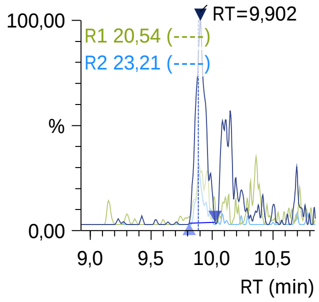

10 ppb002
Conc -5,0608
Area 59781
R#1 286,90>134,90 32,44 (41,41)
R#2 286,90>88,90 29,29 (26,41)
Q 286,90>152,85 (+) 1,17e4

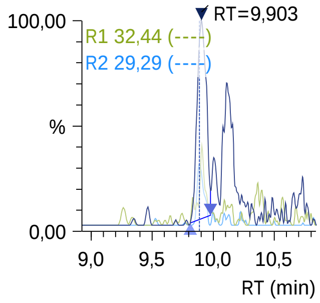

40 ppb001
Conc 67,9292
Area 197037
R#1 286,90>134,90 34,67 (41,41)
R#2 286,90>88,90 28,66 (26,41)
Q 286,90>152,85 (+) 3,86e4

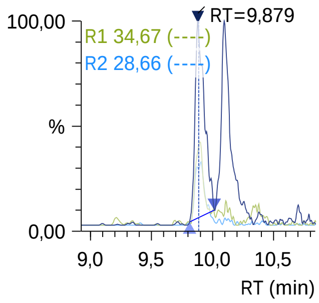

40ppb002
Conc 72,1528
Area 204980
R#1 286,90>134,90 37,16 (41,41)
R#2 286,90>88,90 37,08 (26,41)
Q 286,90>152,85 (+) 4,43e4

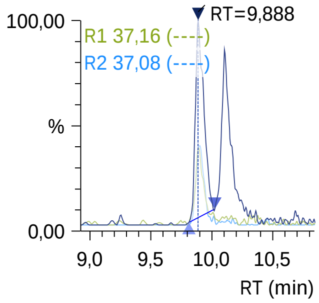

70 ppb001
Conc 178,1208
Area 404250
R#1 286,90>134,90 31,03 (41,41)
R#2 286,90>88,90 28,83 (26,41)
Q 286,90>152,85 (+) 9,04e4

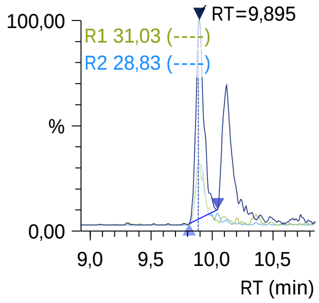

70ppb002
Conc 161,1611
Area 372357
R#1 286,90>134,90 26,17 (41,41)
R#2 286,90>88,90 37,57 (26,41)
Q 286,90>152,85 (+) 8,17e4

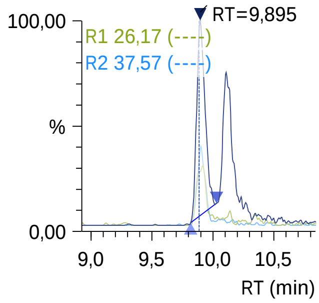

100 ppb001
Conc 191,2826
Area 429000
R#1 286,90>134,90 25,60 (41,41)
R#2 286,90>88,90 31,48 (26,41)
Q 286,90>152,85 (+) 1,02e5

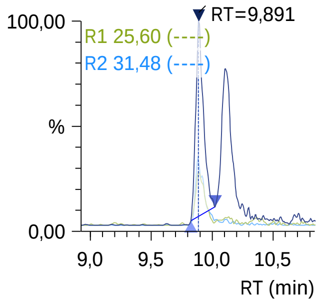

100ppb002
Conc 83,9920
Area 227243
R#1 286,90>134,90 32,01 (41,41)
R#2 286,90>88,90 41,72 (26,41)
Q 286,90>152,85 (+) 4,97e4

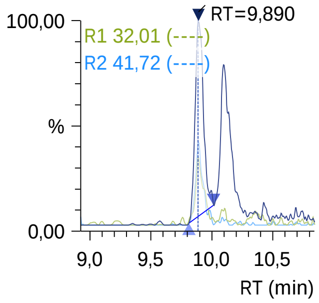

130 ppb001
Conc 130,3873
Area 314488
R#1 286,90>134,90 27,36 (41,41)
R#2 286,90>88,90 32,33 (26,41)
Q 286,90>152,85 (+) 6,07e4

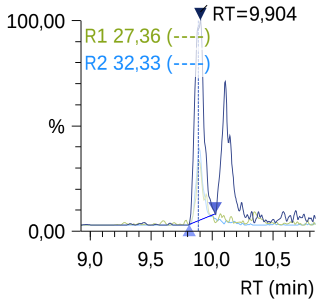

130ppb002
Conc 135,8255
Area 324714
R#1 286,90>134,90 30,05 (41,41)
R#2 286,90>88,90 31,56 (26,41)
Q 286,90>152,85 (+) 6,52e4

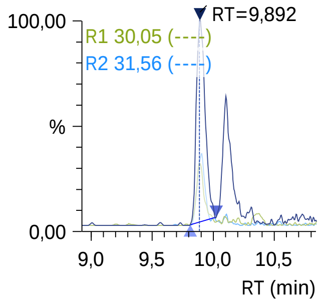

160 ppb001
Conc 137,2738
Area 327438
R#1 286,90>134,90 26,49 (41,41)
R#2 286,90>88,90 38,82 (26,41)
Q 286,90>152,85 (+) 8,00e4

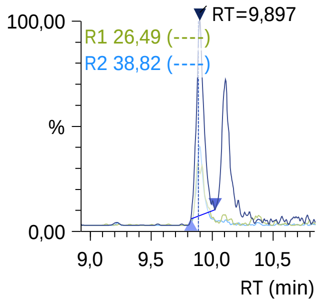

Compound: Luteolin (continued)

160ppb002
Conc 162,4125
Area 374711
R#1 286,90>134,90 35,22 (41,41)
R#2 286,90>88,90 30,25 (26,41)
Q 286,90>152,85 (+)   7,99e4

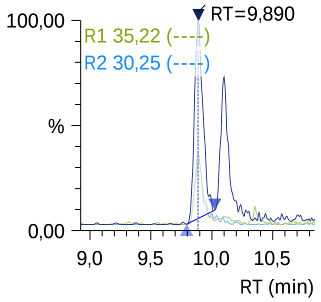

190 ppb001
Conc 211,4030
Area 466836
R#1 286,90>134,90 22,53 (41,41)
R#2 286,90>88,90 31,38 (26,41)
Q 286,90>152,85 (+)   8,78e4

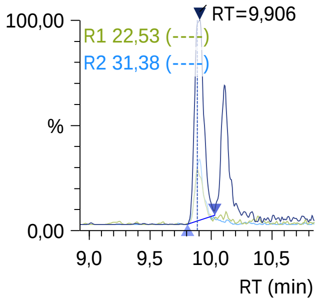

190ppb002
Conc 167,9666
Area 385155
R#1 286,90>134,90 32,39 (41,41)
R#2 286,90>88,90 35,35 (26,41)
Q 286,90>152,85 (+)   9,53e4

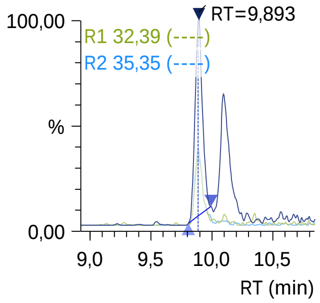

220 ppb001
Conc 222,5519
Area 487801
R#1 286,90>134,90 41,41 (41,41)
R#2 286,90>88,90 26,41 (26,41)
Q 286,90>152,85 (+)   1,08e5

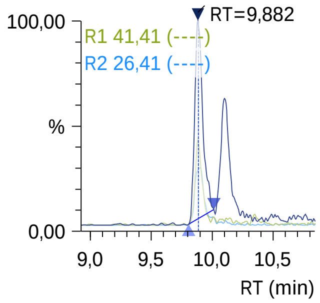

220ppb002
Conc 214,1539
Area 472009
R#1 286,90>134,90 30,95 (41,41)
R#2 286,90>88,90 43,05 (26,41)
Q 286,90>152,85 (+)   1,09e5

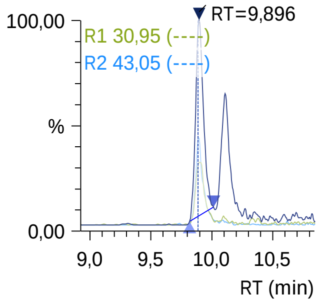

250 ppb001
Conc 266,3796
Area 570218
R#1 286,90>134,90 30,84 (41,41)
R#2 286,90>88,90 32,79 (26,41)
Q 286,90>152,85 (+)   1,26e5

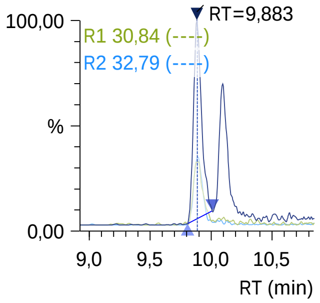

250 ppb002
Conc 251,0610
Area 541412
R#1 286,90>134,90 31,45 (41,41)
R#2 286,90>88,90 36,44 (26,41)
Q 286,90>152,85 (+)   1,22e5

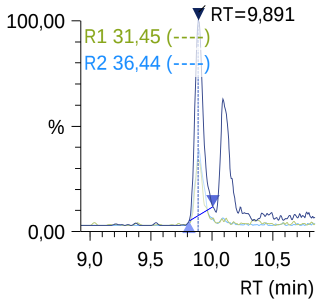

10H
Conc ----
Area ----
R#1 286,90>134,90 ---- (41,41)
R#2 286,90>88,90 ---- (26,41)
Q 286,90>152,85 (+)   1,22e3

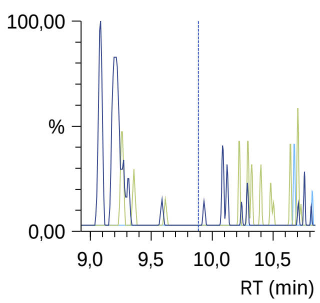

10M
Conc ----
Area ----
R#1 286,90>134,90 ---- (41,41)
R#2 286,90>88,90 ---- (26,41)
Q 286,90>152,85 (+)   3,00e3

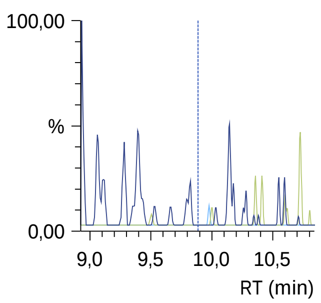

9H
Conc ----
Area ----
R#1 286,90>134,90 ---- (41,41)
R#2 286,90>88,90 ---- (26,41)
Q 286,90>152,85 (+)   2,57e3

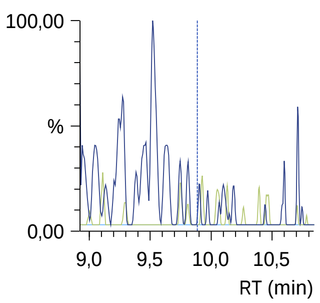

9M
Conc ----
Area ----
R#1 286,90>134,90 ---- (41,41)
R#2 286,90>88,90 ---- (26,41)
Q 286,90>152,85 (+)   4,11e3

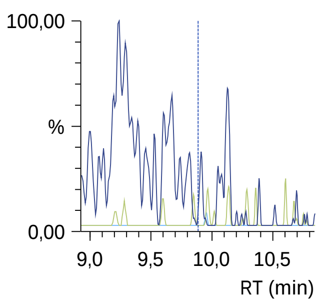

Compound: Baicalin

Curve Fit: Linear | Weighting: None | Zero: Not Forced
Quantitative Method: External Standard
Q 446,90>270,95
R^2 = 0.9655942 R = 0.9826465
y = 7215,872x + 102358,2

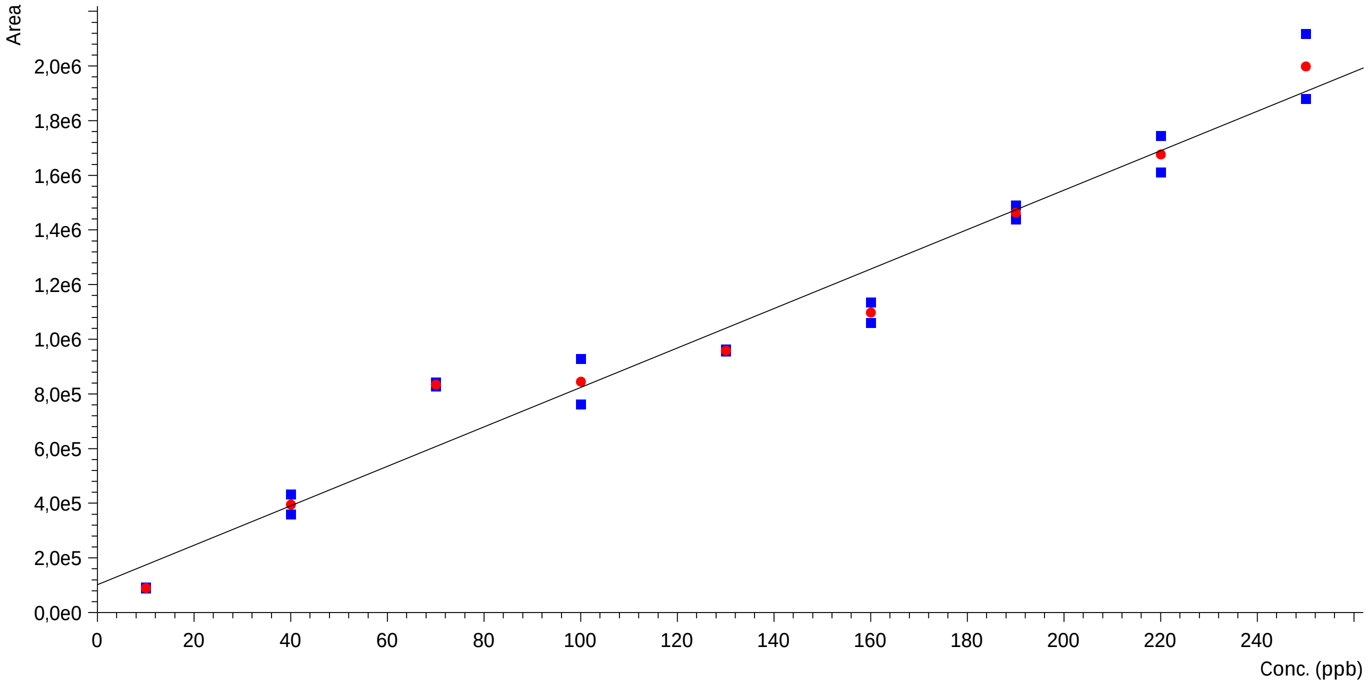

Sample ID	Area	Conc.	Sample Type	Level	Cal Point	Found RT	Ref 1 Actual Ratio
0	----	----	Ctrl.	0	----	----	----
10ppb001	89172	-1,8274	Std.	1	Yes	9,674	16,99
10 ppb002	91596	-1,4915	Std.	1	Yes	9,683	40,77
40 ppb001	358956	35,5602	Std.	2	Yes	9,681	26,94
40ppb002	432290	45,7231	Std.	2	Yes	9,689	21,18
70 ppb001	843411	102,6976	Std.	3	Yes	9,689	25,19
70ppb002	827536	100,4976	Std.	3	Yes	9,684	24,24
100 ppb001	928959	114,5531	Std.	4	Yes	9,676	23,42
100ppb002	761169	91,3002	Std.	4	Yes	9,679	25,97
130 ppb001	955183	118,1874	Std.	5	Yes	9,677	20,47
130ppb002	963294	119,3115	Std.	5	Yes	9,677	26,77
160 ppb001	1136201	143,2734	Std.	6	Yes	9,679	25,66
160ppb002	1060297	132,7545	Std.	6	Yes	9,667	27,40
190 ppb001	1437757	185,0641	Std.	7	Yes	9,685	24,23
190ppb002	1489806	192,2772	Std.	7	Yes	9,671	25,45
220 ppb001	1609770	208,9023	Std.	8	Yes	9,672	23,64
220ppb002	1743731	227,4670	Std.	8	Yes	9,680	23,27
250 ppb001	2117721	279,2958	Std.	9	Yes	9,667	22,87
250 ppb002	1880739	246,4540	Std.	9	Yes	9,673	25,69
10H	----	----	Unk.	----	----	----	----
10M	----	----	Unk.	----	----	----	----
9H	----	----	Unk.	----	----	----	----
9M	----	----	Unk.	----	----	----	----
Average	1040421	130,0000				9,678	25,01
%RSD	57	62,7056				0,068	18,80
Maximum	2117721	279,2958				9,689	40,77
Minimum	89172	-1,8274				9,667	16,99
Std. Dev.	588219	81,5173				0,007	4,70

Compound: Baicalin (continued)

0
Conc ----
Area ----
R#1 446,90>122,85 ---- (27,40)
R#2 446,90>252,85 ---- (4,50)
Q 446,90>270,95 (+) 2,71e3

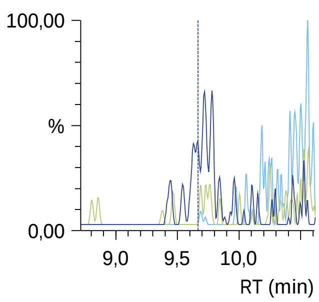

10ppb001
Conc -1,8274
Area 89172
R#1 446,90>122,85 16,99 (27,40)
R#2 446,90>252,85 2,31 (4,50)
Q 446,90>270,95 (+) 1,50e4

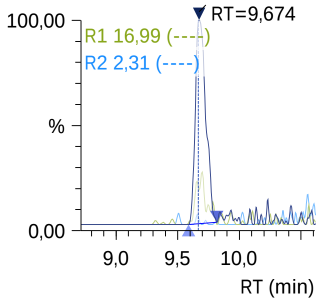

10 ppb002
Conc -1,4915
Area 91596
R#1 446,90>122,85 40,77 (27,40)
R#2 446,90>252,85 1,61 (4,50)
Q 446,90>270,95 (+) 1,54e4

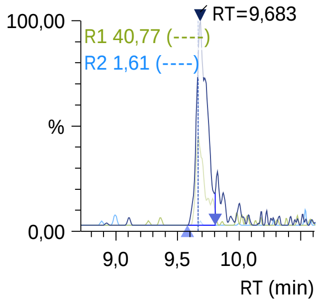

40 ppb001
Conc 35,5602
Area 358956
R#1 446,90>122,85 26,94 (27,40)
R#2 446,90>252,85 5,12 (4,50)
Q 446,90>270,95 (+) 7,16e4

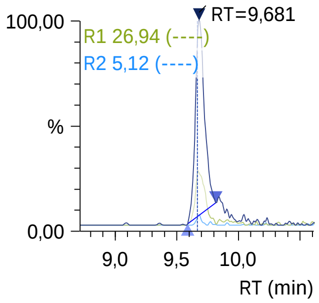

40ppb002
Conc 45,7231
Area 432290
R#1 446,90>122,85 21,18 (27,40)
R#2 446,90>252,85 6,53 (4,50)
Q 446,90>270,95 (+) 7,54e4

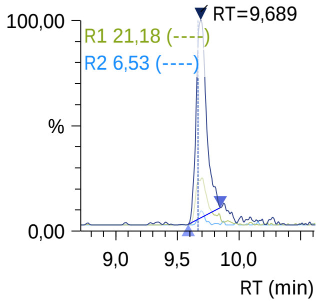

70 ppb001
Conc 102,6976
Area 843411
R#1 446,90>122,85 25,19 (27,40)
R#2 446,90>252,85 2,38 (4,50)
Q 446,90>270,95 (+) 1,50e5

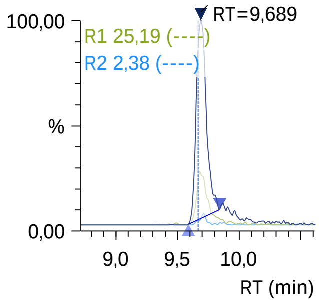

70ppb002
Conc 100,4976
Area 827536
R#1 446,90>122,85 24,24 (27,40)
R#2 446,90>252,85 6,35 (4,50)
Q 446,90>270,95 (+) 1,56e5

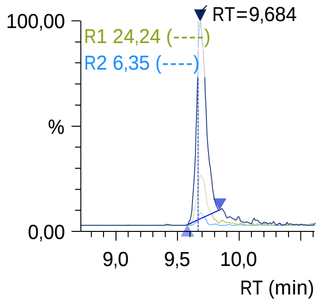

100 ppb001
Conc 114,5531
Area 928959
R#1 446,90>122,85 23,42 (27,40)
R#2 446,90>252,85 3,18 (4,50)
Q 446,90>270,95 (+) 1,69e5

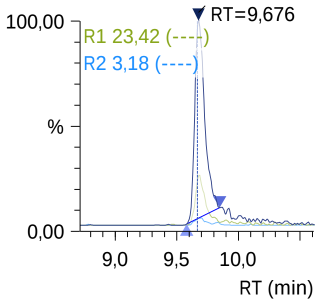

100ppb002
Conc 91,3002
Area 761169
R#1 446,90>122,85 25,97 (27,40)
R#2 446,90>252,85 2,58 (4,50)
Q 446,90>270,95 (+) 1,40e5

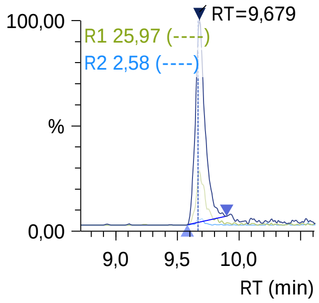

130 ppb001
Conc 118,1874
Area 955183
R#1 446,90>122,85 20,47 (27,40)
R#2 446,90>252,85 2,64 (4,50)
Q 446,90>270,95 (+) 1,85e5

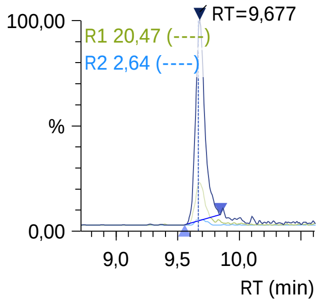

130ppb002
Conc 119,3115
Area 963294
R#1 446,90>122,85 26,77 (27,40)
R#2 446,90>252,85 4,87 (4,50)
Q 446,90>270,95 (+) 1,82e5

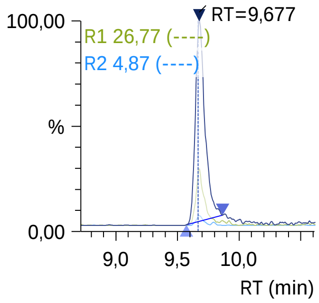

160 ppb001
Conc 143,2734
Area 1136201
R#1 446,90>122,85 25,66 (27,40)
R#2 446,90>252,85 5,08 (4,50)
Q 446,90>270,95 (+) 2,02e5

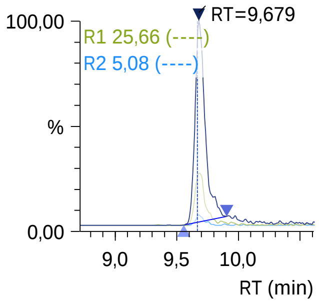

Compound: Baicalin (continued)

160ppb002
Conc 132,7545
Area 1060297
R#1 446,90>122,85 27,40 (27,40)
R#2 446,90>252,85 4,50 (4,50)
Q 446,90>270,95 (+) 2,05e5

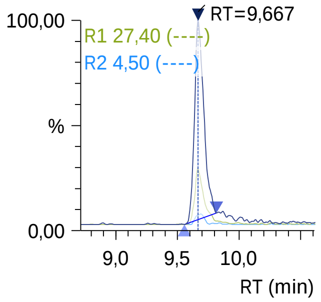

190 ppb001
Conc 185,0641
Area 1437757
R#1 446,90>122,85 24,23 (27,40)
R#2 446,90>252,85 3,44 (4,50)
Q 446,90>270,95 (+) 2,59e5

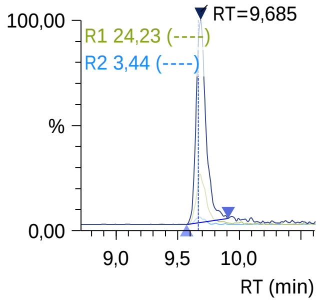

190ppb002
Conc 192,2772
Area 1489806
R#1 446,90>122,85 25,45 (27,40)
R#2 446,90>252,85 4,61 (4,50)
Q 446,90>270,95 (+) 2,73e5

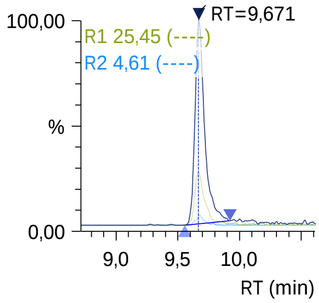

220 ppb001
Conc 208,9023
Area 1609770
R#1 446,90>122,85 23,64 (27,40)
R#2 446,90>252,85 4,82 (4,50)
Q 446,90>270,95 (+) 3,05e5

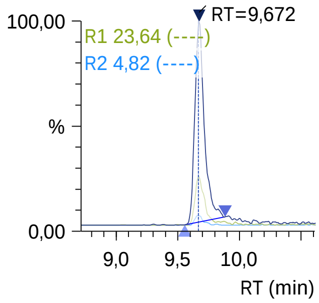

220ppb002
Conc 227,4670
Area 1743731
R#1 446,90>122,85 23,27 (27,40)
R#2 446,90>252,85 3,74 (4,50)
Q 446,90>270,95 (+) 3,14e5

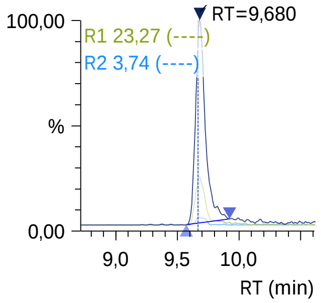

250 ppb001
Conc 279,2958
Area 2117721
R#1 446,90>122,85 22,87 (27,40)
R#2 446,90>252,85 3,91 (4,50)
Q 446,90>270,95 (+) 3,79e5

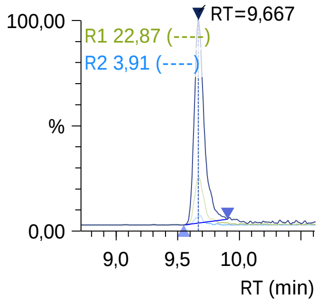

250 ppb002
Conc 246,4540
Area 1880739
R#1 446,90>122,85 25,69 (27,40)
R#2 446,90>252,85 3,94 (4,50)
Q 446,90>270,95 (+) 3,61e5

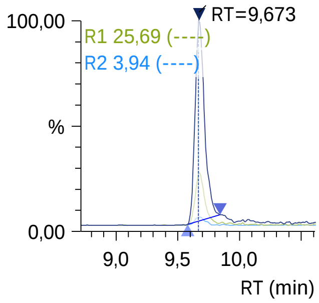

10H
Conc ----
Area ----
R#1 446,90>122,85 ---- (27,40)
R#2 446,90>252,85 ---- (4,50)
Q 446,90>270,95 (+) 1,11e3

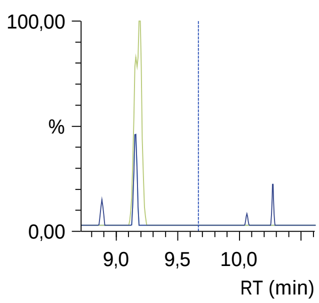

10M
Conc ----
Area ----
R#1 446,90>122,85 ---- (27,40)
R#2 446,90>252,85 ---- (4,50)
Q 446,90>270,95 (+) 8,81e2

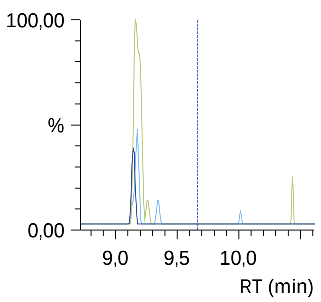

9H
Conc ----
Area ----
R#1 446,90>122,85 ---- (27,40)
R#2 446,90>252,85 ---- (4,50)
Q 446,90>270,95 (+) 3,06e3

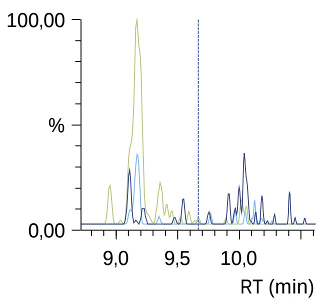

9M
Conc ----
Area ----
R#1 446,90>122,85 ---- (27,40)
R#2 446,90>252,85 ---- (4,50)
Q 446,90>270,95 (+) 2,60e3

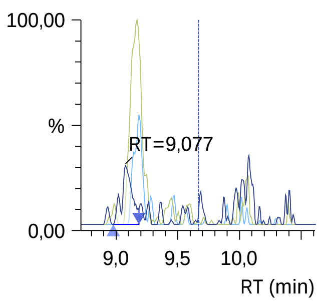

Compound: Chrysin

Curve Fit: Linear | Weighting: None | Zero: Not Forced
Quantitative Method: External Standard
Q 254,90>152,80
R^2 = 0.9557416 R = 0.9776204
y = 17602,16x - 10584,63

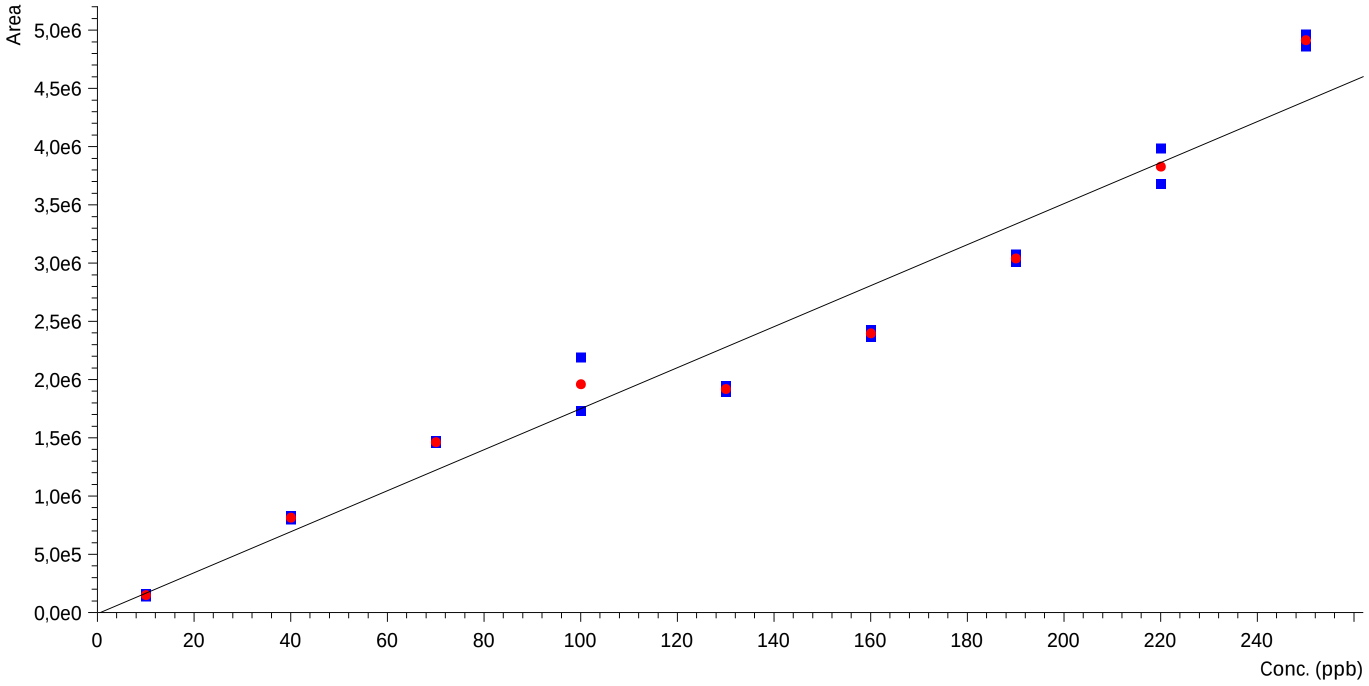

Sample ID	Area	Conc.	Sample Type	Level	Cal Point	Found RT	Ref 1 Actual Ratio
0	----	----	Ctrl.	0	----	----	----
10ppb001	141280	8,6276	Std.	1	Yes	10,893	128,95
10 ppb002	163344	9,8811	Std.	1	Yes	10,897	141,35
40 ppb001	829516	47,7271	Std.	2	Yes	10,888	83,81
40ppb002	801884	46,1573	Std.	2	Yes	10,890	80,84
70 ppb001	1473030	84,2859	Std.	3	Yes	10,898	76,07
70ppb002	1456533	83,3488	Std.	3	Yes	10,894	78,38
100 ppb001	2188502	124,9328	Std.	4	Yes	10,894	76,36
100ppb002	1734420	99,1359	Std.	4	Yes	10,887	81,63
130 ppb001	1894601	108,2359	Std.	5	Yes	10,884	85,04
130ppb002	1946363	111,1766	Std.	5	Yes	10,891	80,64
160 ppb001	2366607	135,0512	Std.	6	Yes	10,888	77,50
160ppb002	2428308	138,5565	Std.	6	Yes	10,879	80,83
190 ppb001	3010417	171,6267	Std.	7	Yes	10,891	78,56
190ppb002	3073414	175,2057	Std.	7	Yes	10,879	73,54
220 ppb001	3678543	209,5838	Std.	8	Yes	10,881	72,90
220ppb002	3982757	226,8666	Std.	8	Yes	10,889	71,04
250 ppb001	4863586	276,9075	Std.	9	Yes	10,875	68,85
250 ppb002	4965425	282,6931	Std.	9	Yes	10,885	72,38
10H	----	----	Unk.	----	----	----	----
10M	----	----	Unk.	----	----	----	----
9H	----	----	Unk.	----	----	----	----
9M	----	----	Unk.	----	----	----	----
Average	2277696	130,0000				10,888	83,81
%RSD	63	62,8590				0,060	23,04
Maximum	4965425	282,6931				10,898	141,35
Minimum	141280	8,6276				10,875	68,85
Std. Dev.	1438390	81,7167				0,006	19,31

Compound: Chrysin (continued)

0
Conc ----
Area ----
R#1 254,90>76,85 ---- (72,38)
R#2 254,90>102,95 ---- (55,32)
Q 254,90>152,80 (+) 1,94e3

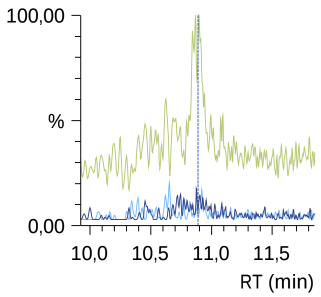

10ppb001
Conc 8,6276
Area 141280
R#1 254,90>76,85 128,95 (72,38)
R#2 254,90>102,95 66,20 (55,32)
Q 254,90>152,80 (+) 3,30e4

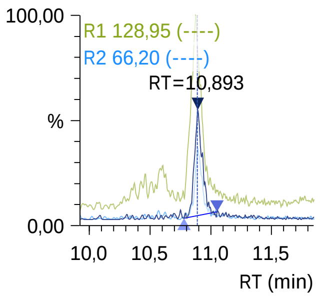

10 ppb002
Conc 9,8811
Area 163344
R#1 254,90>76,85 141,35 (72,38)
R#2 254,90>102,95 54,56 (55,32)
Q 254,90>152,80 (+) 3,49e4

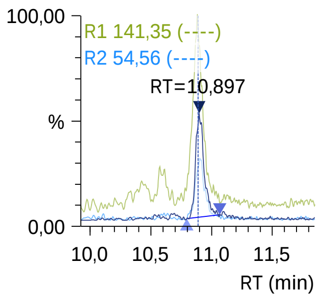

40 ppb001
Conc 47,7271
Area 829516
R#1 254,90>76,85 83,81 (72,38)
R#2 254,90>102,95 56,24 (55,32)
Q 254,90>152,80 (+) 1,64e5

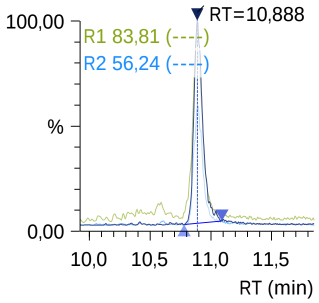

40ppb002
Conc 46,1573
Area 801884
R#1 254,90>76,85 80,84 (72,38)
R#2 254,90>102,95 62,62 (55,32)
Q 254,90>152,80 (+) 1,53e5

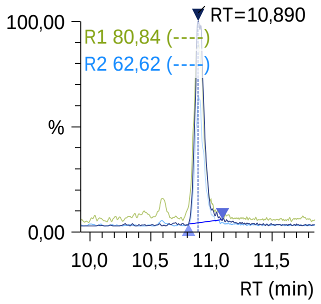

70 ppb001
Conc 84,2859
Area 1473030
R#1 254,90>76,85 76,07 (72,38)
R#2 254,90>102,95 58,85 (55,32)
Q 254,90>152,80 (+) 2,86e5

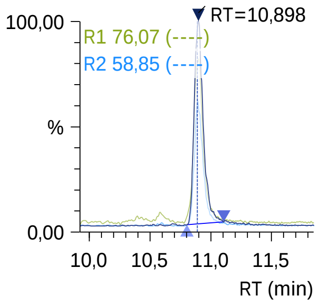

70ppb002
Conc 83,3488
Area 1456533
R#1 254,90>76,85 78,38 (72,38)
R#2 254,90>102,95 58,09 (55,32)
Q 254,90>152,80 (+) 2,84e5

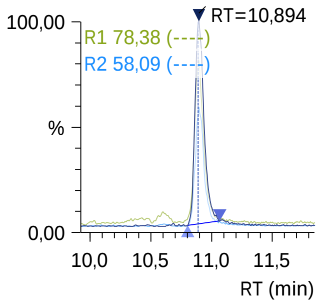

100 ppb001
Conc 124,9328
Area 2188502
R#1 254,90>76,85 76,36 (72,38)
R#2 254,90>102,95 59,37 (55,32)
Q 254,90>152,80 (+) 4,28e5

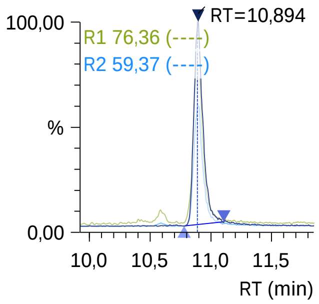

100ppb002
Conc 99,1359
Area 1734420
R#1 254,90>76,85 81,63 (72,38)
R#2 254,90>102,95 57,50 (55,32)
Q 254,90>152,80 (+) 3,51e5

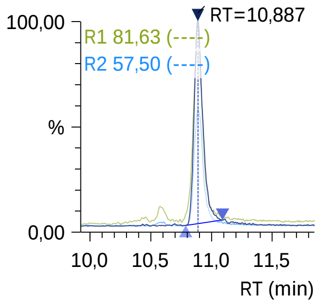

130 ppb001
Conc 108,2359
Area 1894601
R#1 254,90>76,85 85,04 (72,38)
R#2 254,90>102,95 56,22 (55,32)
Q 254,90>152,80 (+) 3,81e5

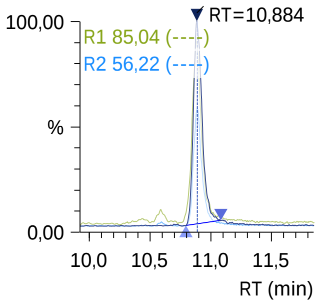

130ppb002
Conc 111,1766
Area 1946363
R#1 254,90>76,85 80,64 (72,38)
R#2 254,90>102,95 56,87 (55,32)
Q 254,90>152,80 (+) 3,88e5

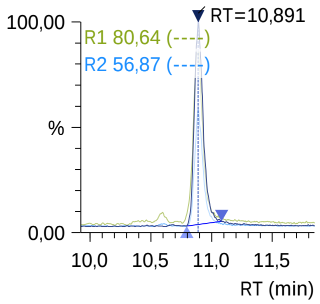

160 ppb001
Conc 135,0512
Area 2366607
R#1 254,90>76,85 77,50 (72,38)
R#2 254,90>102,95 52,82 (55,32)
Q 254,90>152,80 (+) 4,80e5

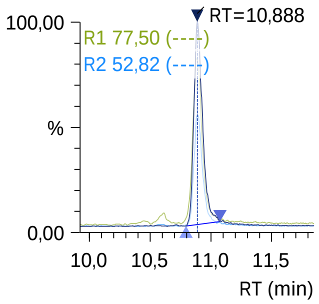

Compound: Chrysin (continued)

160ppb002
Conc 138,5565
Area 2428308
R#1 254,90>76,85 80,83 (72,38)
R#2 254,90>102,95 52,15 (55,32)
Q 254,90>152,80 (+) 5,05e5

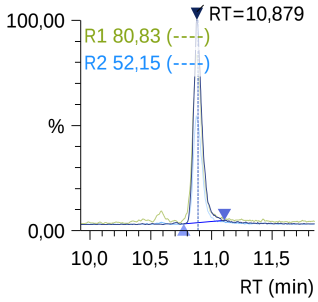

190 ppb001
Conc 171,6267
Area 3010417
R#1 254,90>76,85 78,56 (72,38)
R#2 254,90>102,95 52,17 (55,32)
Q 254,90>152,80 (+) 6,16e5

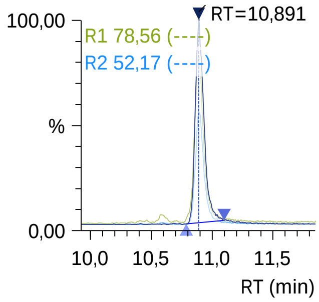

190ppb002
Conc 175,2057
Area 3073414
R#1 254,90>76,85 73,54 (72,38)
R#2 254,90>102,95 54,98 (55,32)
Q 254,90>152,80 (+) 6,29e5

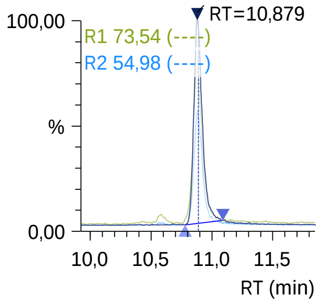

220 ppb001
Conc 209,5838
Area 3678543
R#1 254,90>76,85 72,90 (72,38)
R#2 254,90>102,95 54,76 (55,32)
Q 254,90>152,80 (+) 7,55e5

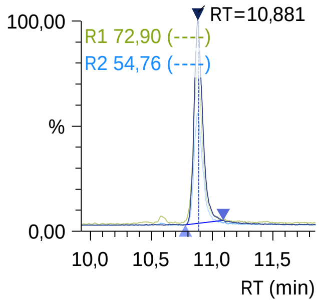

220ppb002
Conc 226,8666
Area 3982757
R#1 254,90>76,85 71,04 (72,38)
R#2 254,90>102,95 52,83 (55,32)
Q 254,90>152,80 (+) 8,24e5

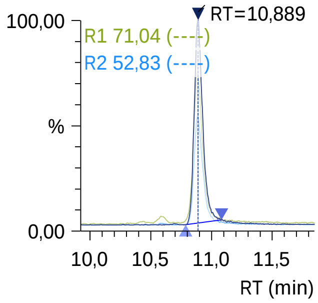

250 ppb001
Conc 276,9075
Area 4863586
R#1 254,90>76,85 68,85 (72,38)
R#2 254,90>102,95 56,46 (55,32)
Q 254,90>152,80 (+) 9,88e5

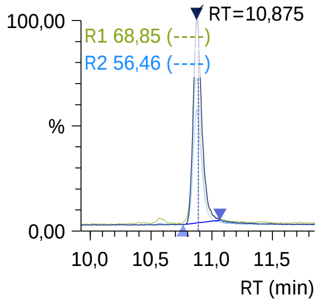

250 ppb002
Conc 282,6931
Area 4965425
R#1 254,90>76,85 72,38 (72,38)
R#2 254,90>102,95 55,32 (55,32)
Q 254,90>152,80 (+) 1,03e6

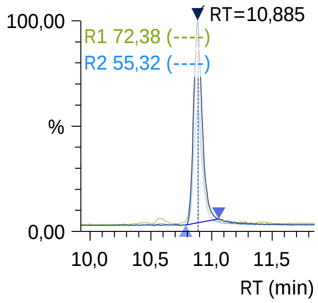

10H
Conc ----
Area ----
R#1 254,90>76,85 ---- (72,38)
R#2 254,90>102,95 ---- (55,32)
Q 254,90>152,80 (+) 9,30e2

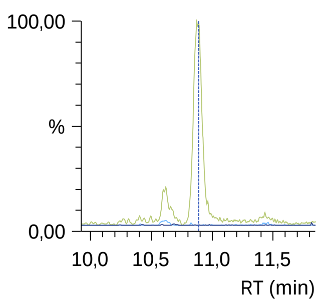

10M
Conc ----
Area ----
R#1 254,90>76,85 ---- (72,38)
R#2 254,90>102,95 ---- (55,32)
Q 254,90>152,80 (+) 1,61e3

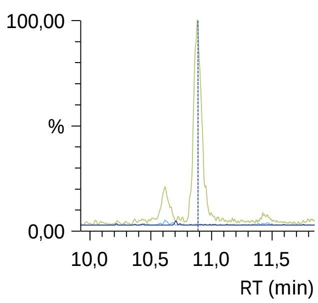

9H
Conc ----
Area ----
R#1 254,90>76,85 ---- (72,38)
R#2 254,90>102,95 ---- (55,32)
Q 254,90>152,80 (+) 9,93e2

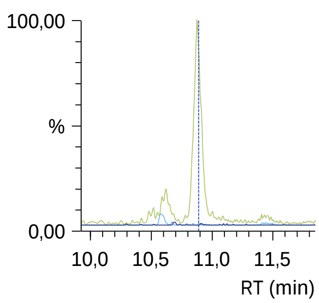

9M
Conc ----
Area ----
R#1 254,90>76,85 ---- (72,38)
R#2 254,90>102,95 ---- (55,32)
Q 254,90>152,80 (+) 9,66e2

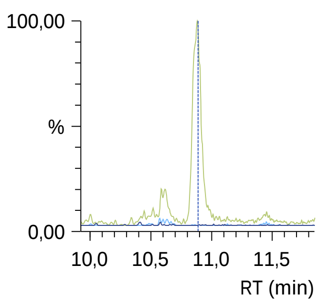

Compound: Kaempherol

Curve Fit: Linear | Weighting: None | Zero: Not Forced
Quantitative Method: External Standard
Q 286,80>152,90
R^2 = 0.9499101 R = 0.9746333
y = 2346,105x + 93405,49

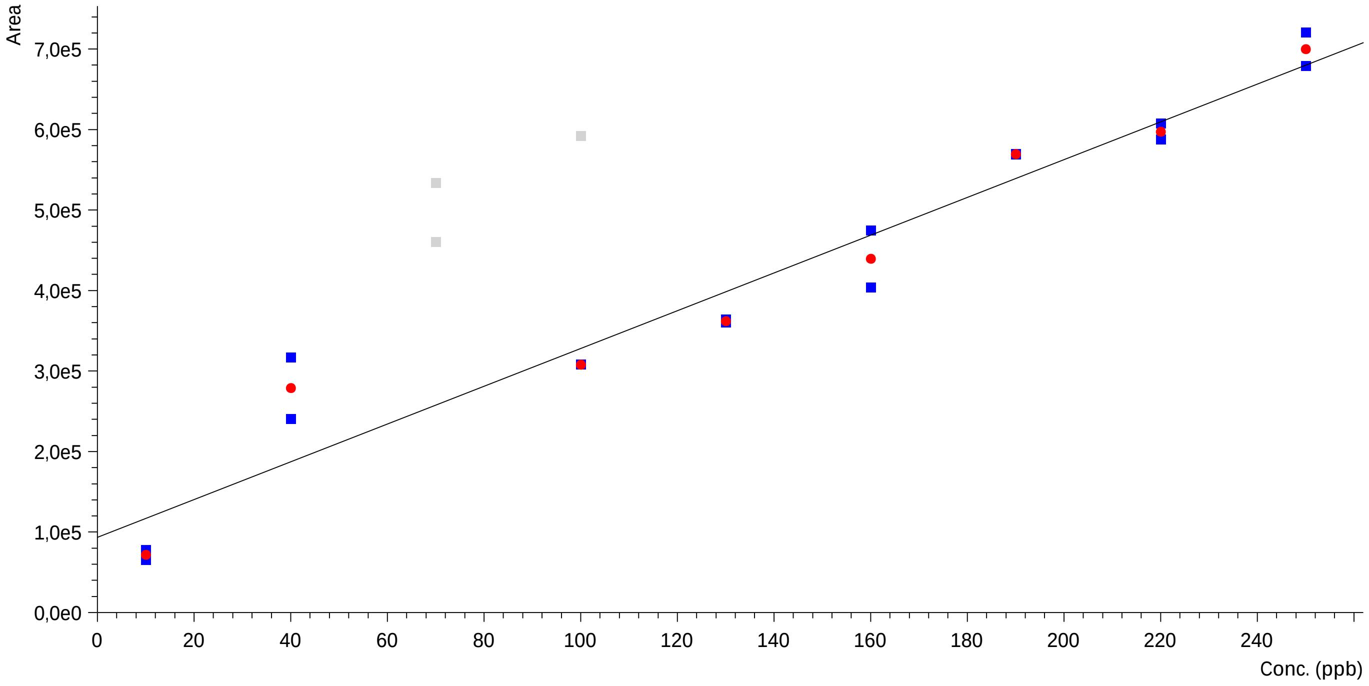

Sample ID	Area	Conc.	Sample Type	Level	Cal Point	Found RT	Ref 1 Actual Ratio
0	14900	-33,4620	Ctrl.	0	----	9,935	0,00
10ppb001	65736	-11,7938	Std.	1	Yes	9,885	6,53
10 ppb002	77855	-6,6283	Std.	1	Yes	9,905	0,00
40 ppb001	240890	62,8637	Std.	2	Yes	9,877	0,00
40ppb002	317354	95,4556	Std.	2	Yes	9,890	0,00
70 ppb001	460660	156,5382	Std.	3	No	9,901	0,00
70ppb002	533797	187,7120	Std.	3	No	9,895	0,93
100 ppb001	592069	212,5494	Std.	4	No	9,895	0,00
100ppb002	308153	91,5337	Std.	4	Yes	9,890	0,49
130 ppb001	360735	113,9462	Std.	5	Yes	9,899	0,48
130ppb002	363966	115,3232	Std.	5	Yes	9,900	0,00
160 ppb001	404023	132,3969	Std.	6	Yes	9,896	0,00
160ppb002	475116	162,6996	Std.	6	Yes	9,889	0,00
190 ppb001	569821	203,0667	Std.	7	Yes	9,899	0,00
190ppb002	569320	202,8532	Std.	7	Yes	9,883	0,00
220 ppb001	587435	210,5745	Std.	8	Yes	9,885	0,00
220ppb002	607256	219,0230	Std.	8	Yes	9,892	0,32
250 ppb001	720581	267,3264	Std.	9	Yes	9,884	0,37
250 ppb002	679523	249,8257	Std.	9	Yes	9,892	0,25
10H	----	----	Unk.	----	----	----	----
10M	----	----	Unk.	----	----	----	----
9H	----	----	Unk.	----	----	----	----
9M	----	----	Unk.	----	----	----	----
Average	418379	138,5160				9,894	0,49
%RSD	50	64,1160				0,122	301,15
Maximum	720581	267,3264				9,935	6,53
Minimum	14900	-33,4620				9,877	0,00
Std. Dev.	208360	88,8109				0,012	1,48

Compound: Kaempherol (continued)

0
Conc -33,4620
Area 14900
R#1 286,80>120,90 0,00 (51,96)
R#2 286,80>68,85 29,58 (57,24)
Q 286,80>152,90 (+) 1,96e3

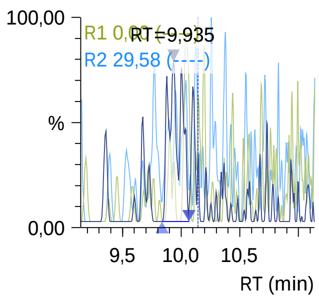

10ppb001
Conc -11,7938
Area 65736
R#1 286,80>120,90 6,53 (51,96)
R#2 286,80>68,85 33,20 (57,24)
Q 286,80>152,90 (+) 1,50e4

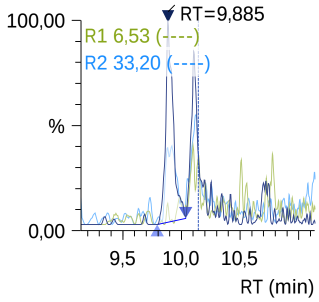

10 ppb002
Conc -6,6283
Area 77855
R#1 286,80>120,90 0,00 (51,96)
R#2 286,80>68,85 46,60 (57,24)
Q 286,80>152,90 (+) 1,35e4

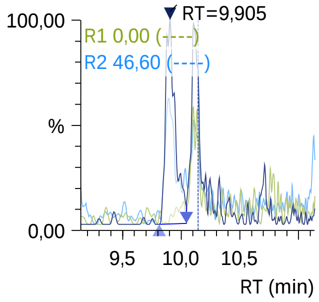

40 ppb001
Conc 62,8637
Area 240890
R#1 286,80>120,90 0,00 (51,96)
R#2 286,80>68,85 42,41 (57,24)
Q 286,80>152,90 (+) 4,76e4

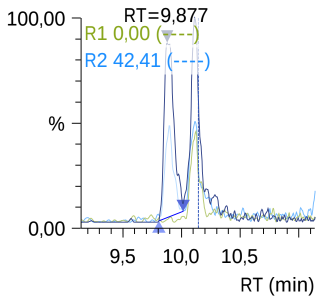

40ppb002
Conc 95,4556
Area 317354
R#1 286,80>120,90 0,00 (51,96)
R#2 286,80>68,85 41,50 (57,24)
Q 286,80>152,90 (+) 5,97e4

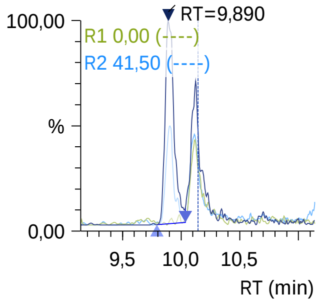

70 ppb001
Conc 156,5382
Area 460660
R#1 286,80>120,90 0,00 (51,96)
R#2 286,80>68,85 39,17 (57,24)
Q 286,80>152,90 (+) 1,05e5

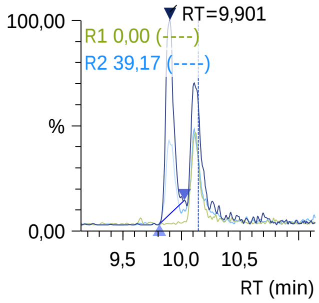

70ppb002
Conc 187,7120
Area 533797
R#1 286,80>120,90 0,93 (51,96)
R#2 286,80>68,85 45,63 (57,24)
Q 286,80>152,90 (+) 1,02e5

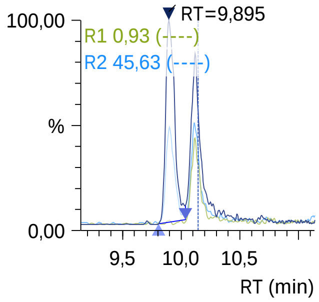

100 ppb001
Conc 212,5494
Area 592069
R#1 286,80>120,90 0,00 (51,96)
R#2 286,80>68,85 43,89 (57,24)
Q 286,80>152,90 (+) 1,24e5

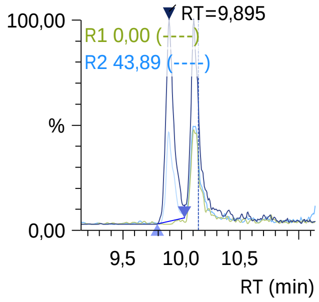

100ppb002
Conc 91,5337
Area 308153
R#1 286,80>120,90 0,49 (51,96)
R#2 286,80>68,85 42,48 (57,24)
Q 286,80>152,90 (+) 7,24e4

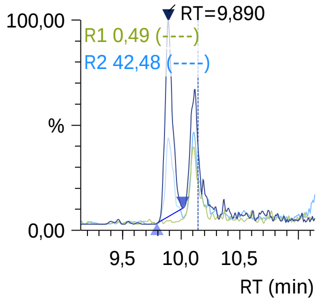

130 ppb001
Conc 113,9462
Area 360735
R#1 286,80>120,90 0,48 (51,96)
R#2 286,80>68,85 37,76 (57,24)
Q 286,80>152,90 (+) 7,59e4

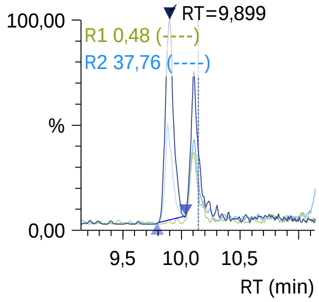

130ppb002
Conc 115,3232
Area 363966
R#1 286,80>120,90 0,00 (51,96)
R#2 286,80>68,85 37,90 (57,24)
Q 286,80>152,90 (+) 8,29e4

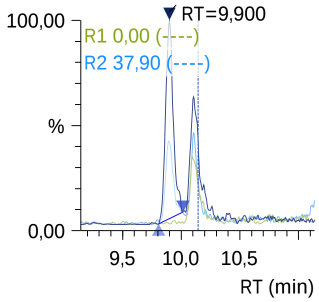

160 ppb001
Conc 132,3969
Area 404023
R#1 286,80>120,90 0,00 (51,96)
R#2 286,80>68,85 42,32 (57,24)
Q 286,80>152,90 (+) 9,11e4

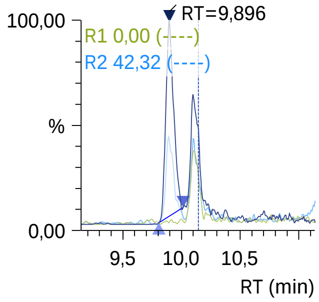

Compound: Kaempherol (continued)

160ppb002
Conc 162,6996
Area 475116
R#1 286,80>120,90 0,00 (51,96)
R#2 286,80>68,85 42,50 (57,24)
Q 286,80>152,90 (+) 1,00e5

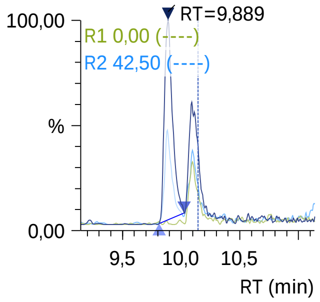

190 ppb001
Conc 203,0667
Area 569821
R#1 286,80>120,90 0,00 (51,96)
R#2 286,80>68,85 39,24 (57,24)
Q 286,80>152,90 (+) 1,13e5

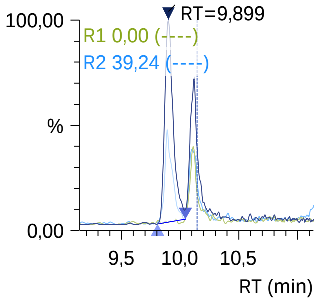

190ppb002
Conc 202,8532
Area 569320
R#1 286,80>120,90 0,00 (51,96)
R#2 286,80>68,85 39,75 (57,24)
Q 286,80>152,90 (+) 1,07e5

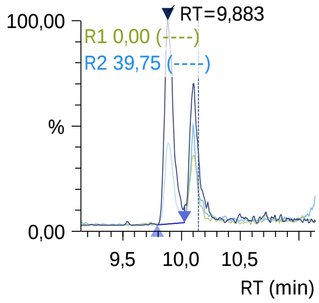

220 ppb001
Conc 210,5745
Area 587435
R#1 286,80>120,90 0,00 (51,96)
R#2 286,80>68,85 50,06 (57,24)
Q 286,80>152,90 (+) 1,22e5

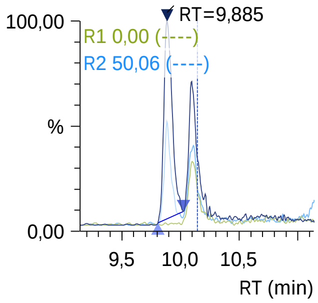

220ppb002
Conc 219,0230
Area 607256
R#1 286,80>120,90 0,32 (51,96)
R#2 286,80>68,85 41,50 (57,24)
Q 286,80>152,90 (+) 1,40e5

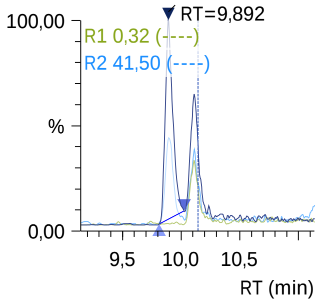

250 ppb001
Conc 267,3264
Area 720581
R#1 286,80>120,90 0,37 (51,96)
R#2 286,80>68,85 43,35 (57,24)
Q 286,80>152,90 (+) 1,50e5

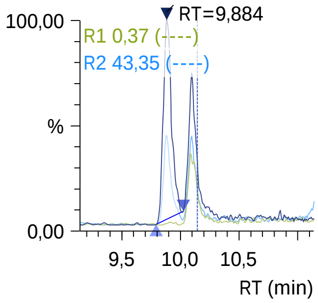

250 ppb002
Conc 249,8257
Area 679523
R#1 286,80>120,90 0,25 (51,96)
R#2 286,80>68,85 43,50 (57,24)
Q 286,80>152,90 (+) 1,48e5

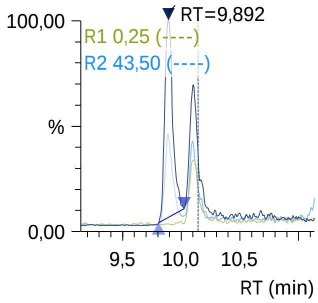

10H
Conc ----
Area ----
R#1 286,80>120,90 ---- (51,96)
R#2 286,80>68,85 ---- (57,24)
Q 286,80>152,90 (+) 1,09e3

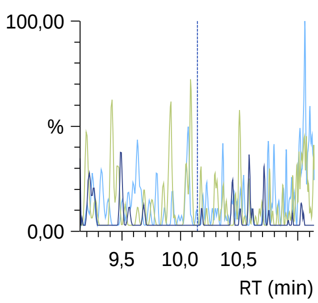

10M
Conc ----
Area ----
R#1 286,80>120,90 ---- (51,96)
R#2 286,80>68,85 ---- (57,24)
Q 286,80>152,90 (+) 2,11e3

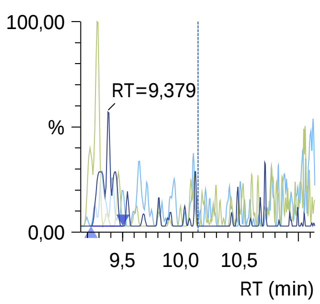

9H
Conc ----
Area ----
R#1 286,80>120,90 ---- (51,96)
R#2 286,80>68,85 ---- (57,24)
Q 286,80>152,90 (+) 2,33e3

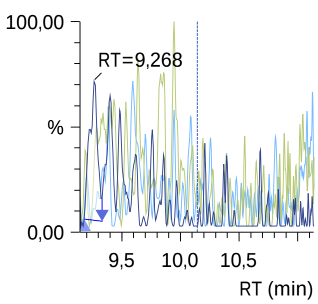

9M
Conc ----
Area ----
R#1 286,80>120,90 ---- (51,96)
R#2 286,80>68,85 ---- (57,24)
Q 286,80>152,90 (+) 1,00e4

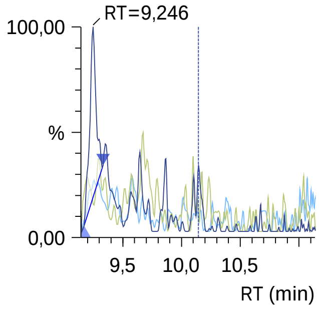

Compound: Cumarina

Curve Fit: Linear | Weighting: None | Zero: Not Forced
Quantitative Method: External Standard
Q 146,80>90,95
R^2 = 0.9922713 R = 0.9961282
y = 6953,266x + 307826,0

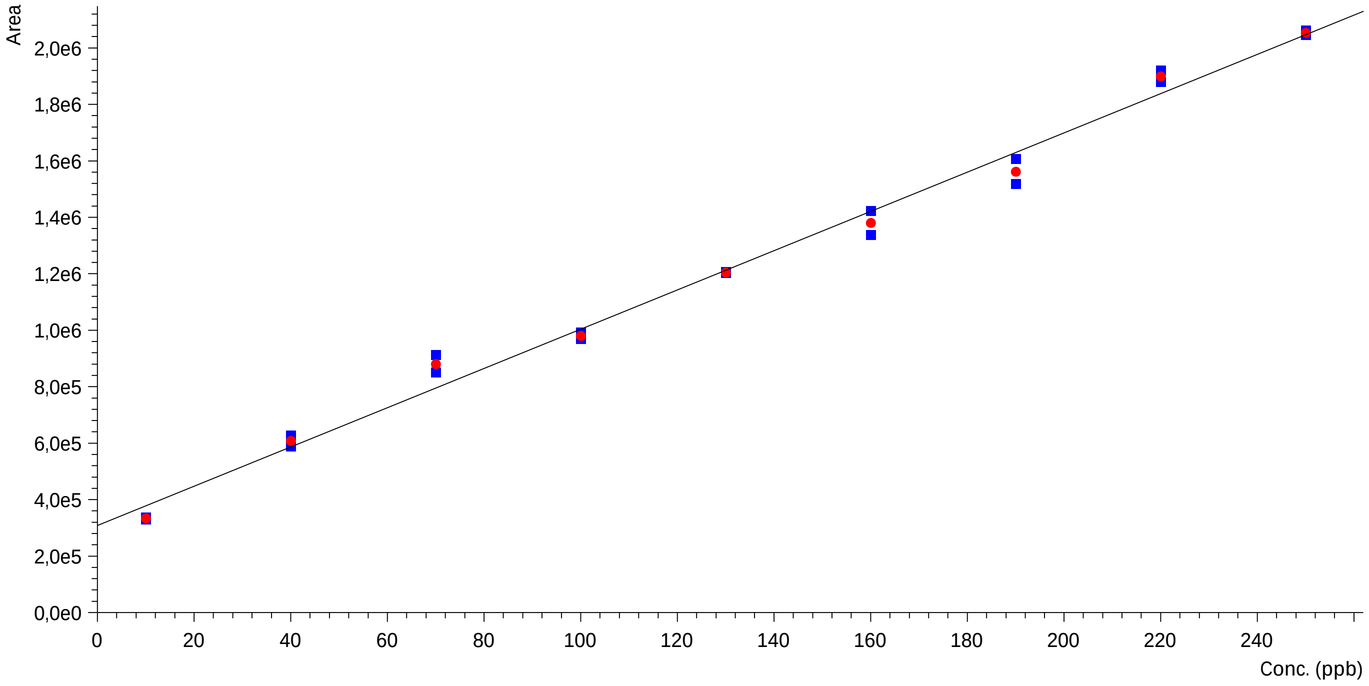

Sample ID	Area	Conc.	Sample Type	Level	Cal Point	Found RT	Ref 1 Actual Ratio
0	136373	-24,6580	Ctrl.	0	----	9,483	28,46
10ppb001	331287	3,3741	Std.	1	Yes	9,435	44,83
10 ppb002	337095	4,2094	Std.	1	Yes	9,450	45,12
40 ppb001	589302	40,4811	Std.	2	Yes	9,437	51,74
40ppb002	627237	45,9368	Std.	2	Yes	9,441	49,51
70 ppb001	912026	86,8944	Std.	3	Yes	9,446	49,78
70ppb002	849979	77,9710	Std.	3	Yes	9,443	48,35
100 ppb001	991434	98,3147	Std.	4	Yes	9,436	51,96
100ppb002	970380	95,2867	Std.	4	Yes	9,440	52,80
130 ppb001	1202774	128,7090	Std.	5	Yes	9,439	49,63
130ppb002	1206450	129,2378	Std.	5	Yes	9,441	50,72
160 ppb001	1422664	160,3330	Std.	6	Yes	9,443	53,91
160ppb002	1339023	148,3039	Std.	6	Yes	9,437	54,73
190 ppb001	1517328	173,9473	Std.	7	Yes	9,446	51,52
190ppb002	1607059	186,8522	Std.	7	Yes	9,440	49,61
220 ppb001	1879829	226,0812	Std.	8	Yes	9,436	50,52
220ppb002	1919801	231,8299	Std.	8	Yes	9,442	54,72
250 ppb001	2061605	252,2237	Std.	9	Yes	9,433	53,95
250 ppb002	2046238	250,0138	Std.	9	Yes	9,436	49,92
10H	68610	-34,4034	Unk.	----	----	9,458	47,42
10M	58545	-35,8510	Unk.	----	----	9,472	59,24
9H	46247	-37,6196	Unk.	----	----	9,501	59,17
9M	61273	-35,4586	Unk.	----	----	9,432	34,90
Average	964459	94,4352				9,446	49,67
%RSD	71	104,4170				0,180	13,66
Maximum	2061605	252,2237				9,501	59,24
Minimum	46247	-37,6196				9,432	28,46
Std. Dev.	685636	98,6064				0,017	6,79

Compound: Cumarina (continued)

0

Conc -24,6580
Area 136373
R#1 146,80>102,95 28,46 (54,72)
R#2 146,80>64,90 49,66 (61,96)
Q 146,80>90,95 (+) 3,26e4

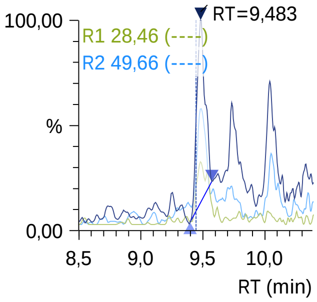

10ppb001

Conc 3,3741
Area 331287
R#1 146,80>102,95 44,83 (54,72)
R#2 146,80>64,90 60,13 (61,96)
Q 146,80>90,95 (+) 7,04e4

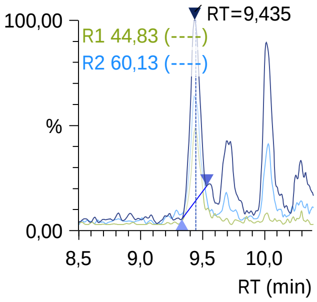

10 ppb002

Conc 4,2094
Area 337095
R#1 146,80>102,95 45,12 (54,72)
R#2 146,80>64,90 52,75 (61,96)
Q 146,80>90,95 (+) 7,33e4

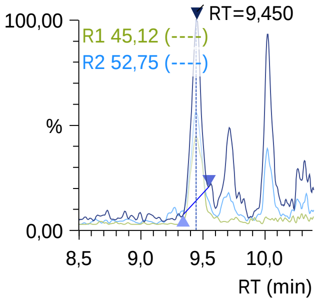

40 ppb001

Conc 40,4811
Area 589302
R#1 146,80>102,95 51,74 (54,72)
R#2 146,80>64,90 62,14 (61,96)
Q 146,80>90,95 (+) 1,19e5

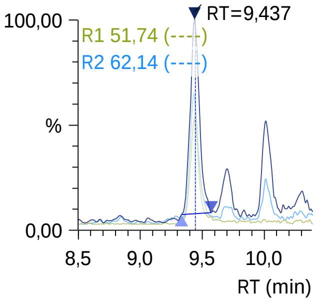

40ppb002

Conc 45,9368
Area 627237
R#1 146,80>102,95 49,51 (54,72)
R#2 146,80>64,90 62,92 (61,96)
Q 146,80>90,95 (+) 1,34e5

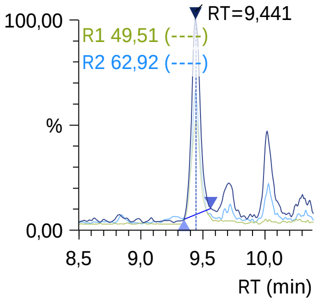

70 ppb001

Conc 86,8944
Area 912026
R#1 146,80>102,95 49,78 (54,72)
R#2 146,80>64,90 63,76 (61,96)
Q 146,80>90,95 (+) 1,88e5

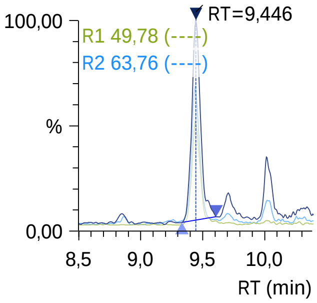

70ppb002

Conc 77,9710
Area 849979
R#1 146,80>102,95 48,35 (54,72)
R#2 146,80>64,90 68,37 (61,96)
Q 146,80>90,95 (+) 1,89e5

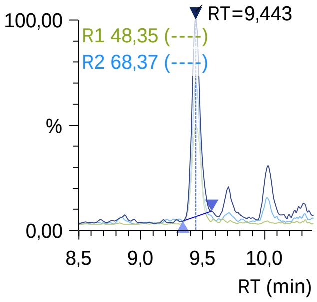

100 ppb001

Conc 98,3147
Area 991434
R#1 146,80>102,95 51,96 (54,72)
R#2 146,80>64,90 65,18 (61,96)
Q 146,80>90,95 (+) 2,18e5

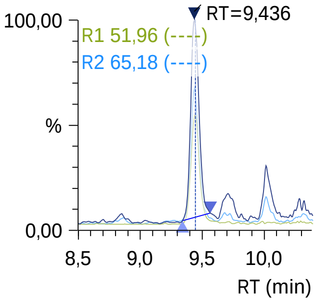

100ppb002

Conc 95,2867
Area 970380
R#1 146,80>102,95 52,80 (54,72)
R#2 146,80>64,90 62,85 (61,96)
Q 146,80>90,95 (+) 2,19e5

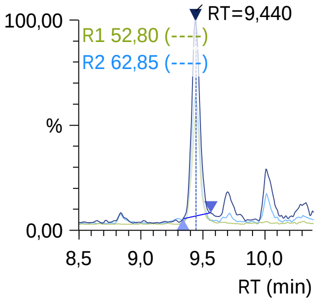

130 ppb001

Conc 128,7090
Area 1202774
R#1 146,80>102,95 49,63 (54,72)
R#2 146,80>64,90 65,73 (61,96)
Q 146,80>90,95 (+) 2,76e5

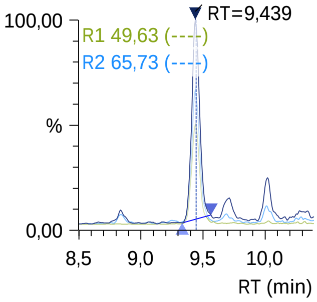

130ppb002

Conc 129,2378
Area 1206450
R#1 146,80>102,95 50,72 (54,72)
R#2 146,80>64,90 59,34 (61,96)
Q 146,80>90,95 (+) 2,85e5

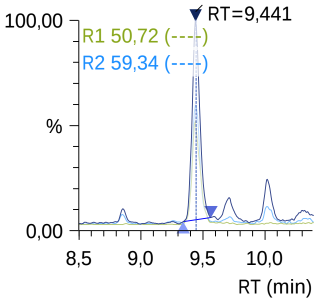

160 ppb001

Conc 160,3330
Area 1422664
R#1 146,80>102,95 53,91 (54,72)
R#2 146,80>64,90 61,39 (61,96)
Q 146,80>90,95 (+) 3,17e5

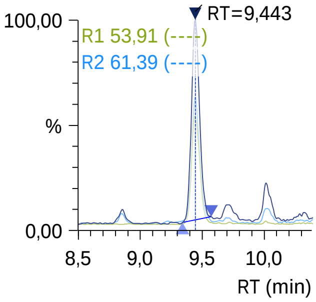

Compound: Cumarina (continued)

160ppb002
Conc 148,3039
Area 1339023
R#1 146,80>102,95 54,73 (54,72)
R#2 146,80>64,90 62,12 (61,96)
Q 146,80>90,95 (+) 3,00e5

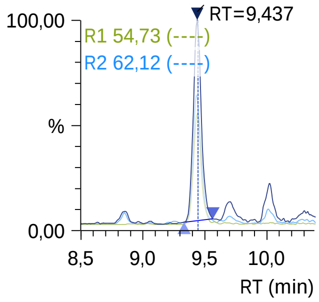

190 ppb001
Conc 173,9473
Area 1517328
R#1 146,80>102,95 51,52 (54,72)
R#2 146,80>64,90 67,87 (61,96)
Q 146,80>90,95 (+) 3,34e5

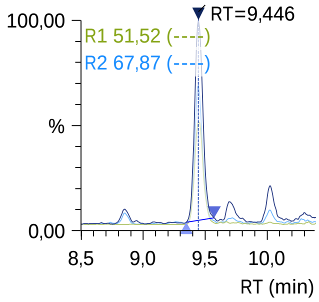

190ppb002
Conc 186,8522
Area 1607059
R#1 146,80>102,95 49,61 (54,72)
R#2 146,80>64,90 67,08 (61,96)
Q 146,80>90,95 (+) 3,74e5

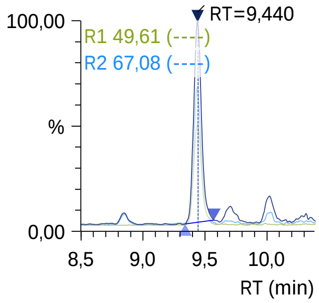

220 ppb001
Conc 226,0812
Area 1879829
R#1 146,80>102,95 50,52 (54,72)
R#2 146,80>64,90 62,01 (61,96)
Q 146,80>90,95 (+) 4,45e5

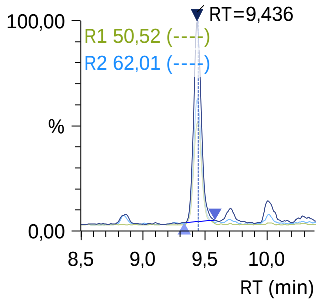

220ppb002
Conc 231,8299
Area 1919801
R#1 146,80>102,95 54,72 (54,72)
R#2 146,80>64,90 61,96 (61,96)
Q 146,80>90,95 (+) 4,26e5

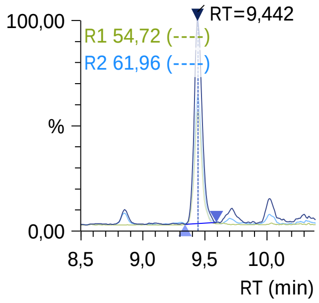

250 ppb001
Conc 252,2237
Area 2061605
R#1 146,80>102,95 53,95 (54,72)
R#2 146,80>64,90 66,21 (61,96)
Q 146,80>90,95 (+) 4,87e5

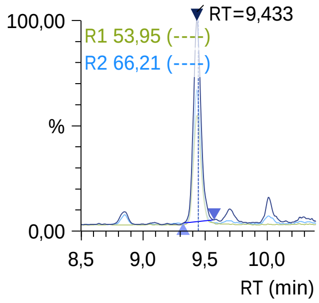

250 ppb002
Conc 250,0138
Area 2046238
R#1 146,80>102,95 49,92 (54,72)
R#2 146,80>64,90 60,97 (61,96)
Q 146,80>90,95 (+) 4,86e5

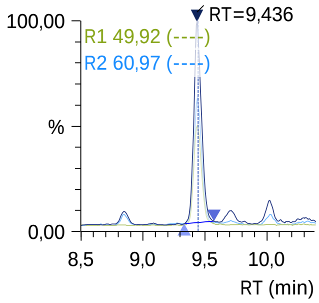

10H
Conc -34,4034
Area 68610
R#1 146,80>102,95 47,42 (54,72)
R#2 146,80>64,90 91,04 (61,96)
Q 146,80>90,95 (+) 2,29e4

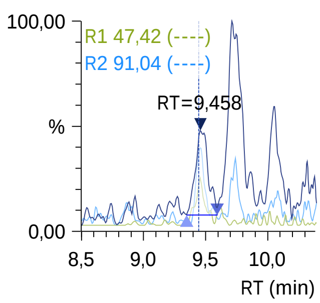

10M
Conc -35,8510
Area 58545
R#1 146,80>102,95 59,24 (54,72)
R#2 146,80>64,90 53,44 (61,96)
Q 146,80>90,95 (+) 2,39e4

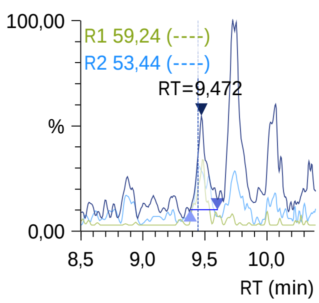

9H
Conc -37,6196
Area 46247
R#1 146,80>102,95 59,17 (54,72)
R#2 146,80>64,90 31,04 (61,96)
Q 146,80>90,95 (+) 2,25e4

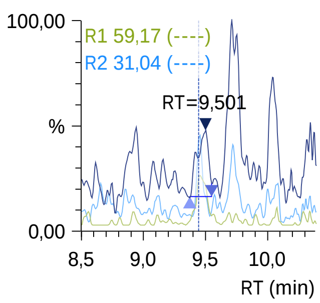

9M
Conc -35,4586
Area 61273
R#1 146,80>102,95 34,90 (54,72)
R#2 146,80>64,90 77,80 (61,96)
Q 146,80>90,95 (+) 2,80e4

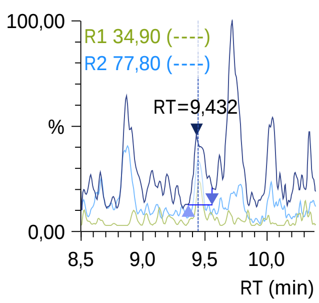

Compound: Cafeina

Curve Fit: Linear | Weighting: None | Zero: Not Forced
Quantitative Method: External Standard
Q 194,90>137,95
R^2 = 0.9974951 R = 0.9987468
y = 2951,902x - 12594,49

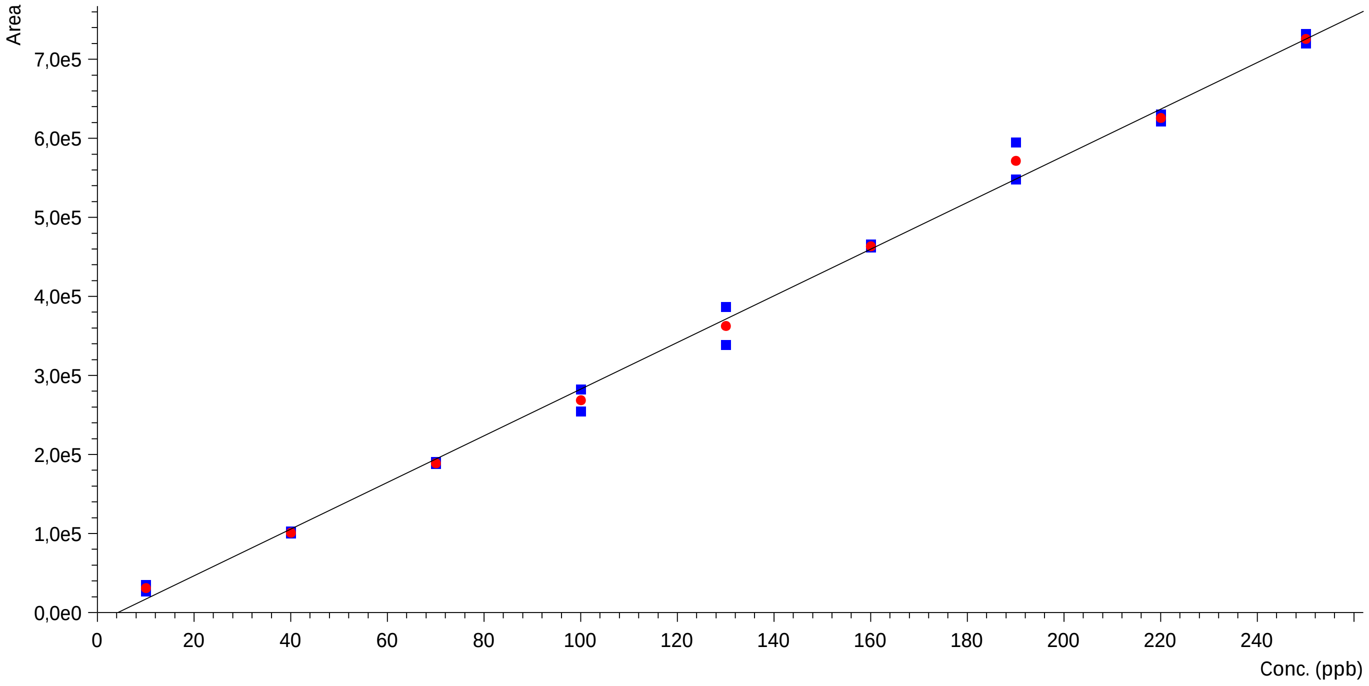

Sample ID	Area	Conc.	Sample Type	Level	Cal Point	Found RT	Ref 1 Actual Ratio
0	----	----	Ctrl.	0	----	----	----
10ppb001	35126	16,1660	Std.	1	Yes	8,572	23,99
10 ppb002	27088	13,4432	Std.	1	Yes	8,560	33,53
40 ppb001	99842	38,0897	Std.	2	Yes	8,562	44,90
40ppb002	102633	39,0350	Std.	2	Yes	8,567	29,36
70 ppb001	190622	68,8425	Std.	3	Yes	8,565	31,50
70ppb002	187750	67,8697	Std.	3	Yes	8,575	33,49
100 ppb001	282626	100,0104	Std.	4	Yes	8,562	30,24
100ppb002	254699	90,5497	Std.	4	Yes	8,564	41,85
130 ppb001	387080	135,3955	Std.	5	Yes	8,562	32,38
130ppb002	338781	119,0336	Std.	5	Yes	8,564	41,07
160 ppb001	461913	160,7463	Std.	6	Yes	8,571	33,20
160ppb002	465673	162,0202	Std.	6	Yes	8,568	31,83
190 ppb001	594931	205,8081	Std.	7	Yes	8,571	29,85
190ppb002	548152	189,9610	Std.	7	Yes	8,558	39,41
220 ppb001	630377	217,8161	Std.	8	Yes	8,563	35,52
220ppb002	621502	214,8093	Std.	8	Yes	8,569	34,47
250 ppb001	719923	248,1511	Std.	9	Yes	8,557	39,46
250 ppb002	732030	252,2526	Std.	9	Yes	8,563	36,81
10H	50320	21,3133	Unk.	----	----	8,588	37,90
10M	181031	65,5934	Unk.	----	----	8,588	38,46
9H	42391	18,6271	Unk.	----	----	8,577	20,88
9M	117754	44,1575	Unk.	----	----	8,577	36,02
Average	321466	113,1678				8,568	34,37
%RSD	74	71,6565				0,099	16,51
Maximum	732030	252,2526				8,588	44,90
Minimum	27088	13,4432				8,557	20,88
Std. Dev.	239376	81,0921				0,009	5,67

Compound: Cafeina (continued)

0
Conc ----
Area ----
R#1 194,90>109,90 ---- (31,33)
R#2 194,90>42,00 ---- (53,07)
Q 194,90>137,95 (+) 2,55e3

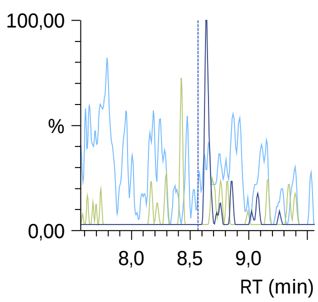

10ppb001
Conc 16,1660
Area 35126
R#1 194,90>109,90 23,99 (31,33)
R#2 194,90>42,00 82,09 (53,07)
Q 194,90>137,95 (+) 7,14e3

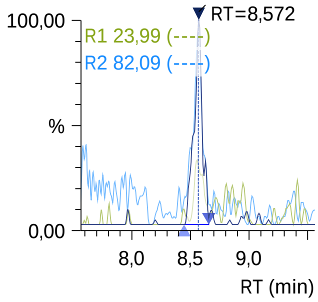

10 ppb002
Conc 13,4432
Area 27088
R#1 194,90>109,90 33,53 (31,33)
R#2 194,90>42,00 42,28 (53,07)
Q 194,90>137,95 (+) 7,25e3

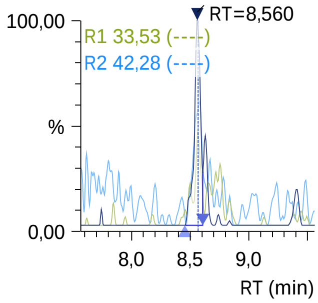

40 ppb001
Conc 38,0897
Area 99842
R#1 194,90>109,90 44,90 (31,33)
R#2 194,90>42,00 67,51 (53,07)
Q 194,90>137,95 (+) 2,17e4

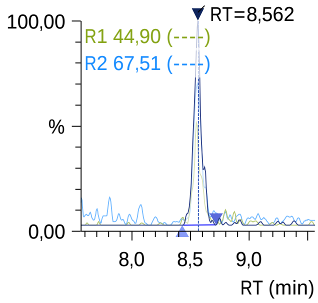

40ppb002
Conc 39,0350
Area 102633
R#1 194,90>109,90 29,36 (31,33)
R#2 194,90>42,00 89,40 (53,07)
Q 194,90>137,95 (+) 2,29e4

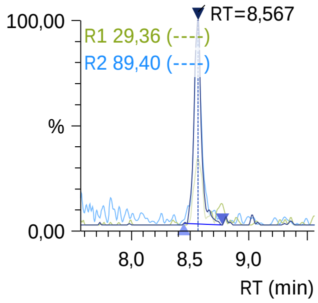

70 ppb001
Conc 68,8425
Area 190622
R#1 194,90>109,90 31,50 (31,33)
R#2 194,90>42,00 60,99 (53,07)
Q 194,90>137,95 (+) 4,55e4

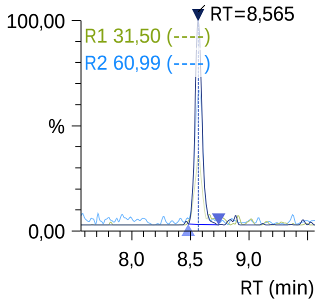

70ppb002
Conc 67,8697
Area 187750
R#1 194,90>109,90 33,49 (31,33)
R#2 194,90>42,00 79,07 (53,07)
Q 194,90>137,95 (+) 3,89e4

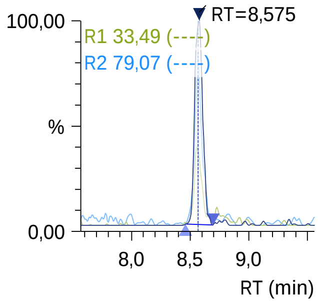

100 ppb001
Conc 100,0104
Area 282626
R#1 194,90>109,90 30,24 (31,33)
R#2 194,90>42,00 63,69 (53,07)
Q 194,90>137,95 (+) 6,56e4

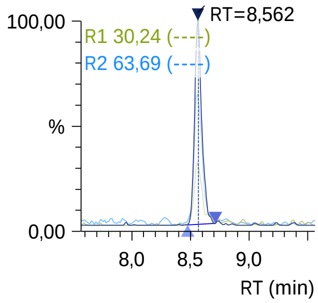

100ppb002
Conc 90,5497
Area 254699
R#1 194,90>109,90 41,85 (31,33)
R#2 194,90>42,00 63,70 (53,07)
Q 194,90>137,95 (+) 5,93e4

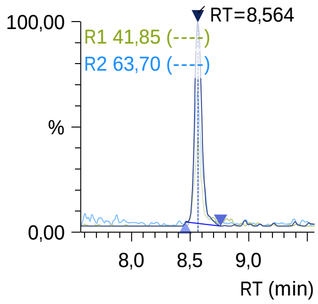

130 ppb001
Conc 135,3955
Area 387080
R#1 194,90>109,90 32,38 (31,33)
R#2 194,90>42,00 69,71 (53,07)
Q 194,90>137,95 (+) 9,00e4

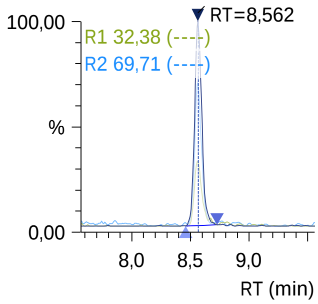

130ppb002
Conc 119,0336
Area 338781
R#1 194,90>109,90 41,07 (31,33)
R#2 194,90>42,00 75,24 (53,07)
Q 194,90>137,95 (+) 7,83e4

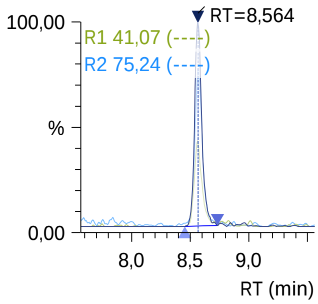

160 ppb001
Conc 160,7463
Area 461913
R#1 194,90>109,90 33,20 (31,33)
R#2 194,90>42,00 63,30 (53,07)
Q 194,90>137,95 (+) 1,08e5

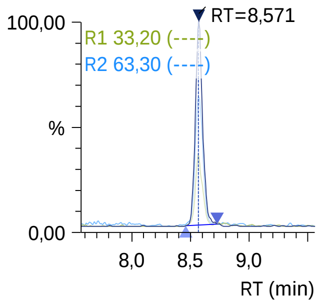

Compound: Cafeina (continued)

160ppb002
Conc 162,0202
Area 465673
R#1 194,90>109,90 31,83 (31,33)
R#2 194,90>42,00 74,82 (53,07)
Q 194,90>137,95 (+) 1,07e5

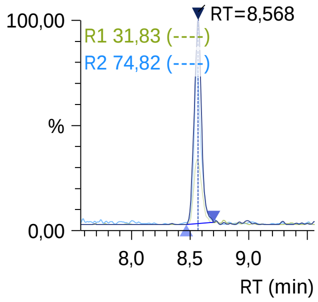

190 ppb001
Conc 205,8081
Area 594931
R#1 194,90>109,90 29,85 (31,33)
R#2 194,90>42,00 55,45 (53,07)
Q 194,90>137,95 (+) 1,48e5

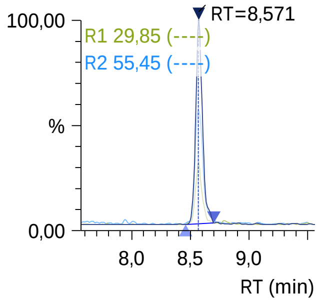

190ppb002
Conc 189,9610
Area 548152
R#1 194,90>109,90 39,41 (31,33)
R#2 194,90>42,00 79,42 (53,07)
Q 194,90>137,95 (+) 1,12e5

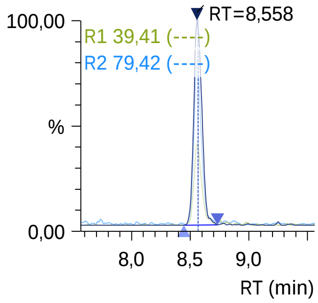

220 ppb001
Conc 217,8161
Area 630377
R#1 194,90>109,90 35,52 (31,33)
R#2 194,90>42,00 64,47 (53,07)
Q 194,90>137,95 (+) 1,48e5

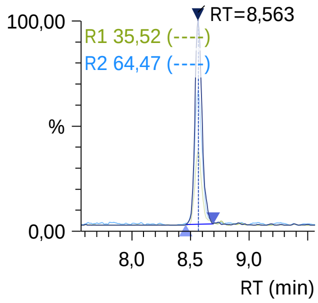

220ppb002
Conc 214,8093
Area 621502
R#1 194,90>109,90 34,47 (31,33)
R#2 194,90>42,00 71,03 (53,07)
Q 194,90>137,95 (+) 1,45e5

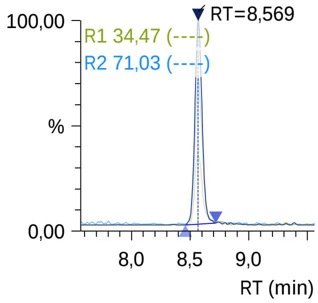

250 ppb001
Conc 248,1511
Area 719923
R#1 194,90>109,90 39,46 (31,33)
R#2 194,90>42,00 70,70 (53,07)
Q 194,90>137,95 (+) 1,63e5

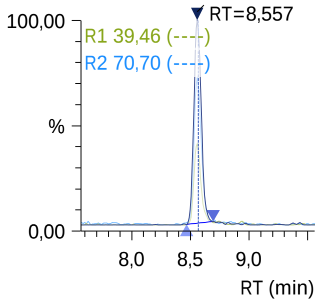

250 ppb002
Conc 252,2526
Area 732030
R#1 194,90>109,90 36,81 (31,33)
R#2 194,90>42,00 77,13 (53,07)
Q 194,90>137,95 (+) 1,62e5

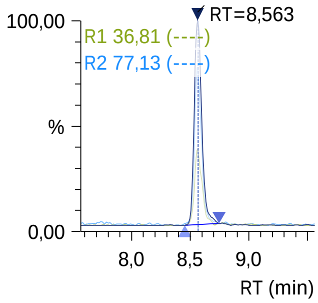

10H
Conc 21,3133
Area 50320
R#1 194,90>109,90 37,90 (31,33)
R#2 194,90>42,00 36,15 (53,07)
Q 194,90>137,95 (+) 5,85e3

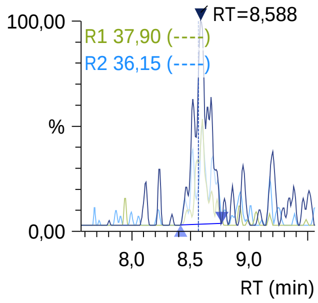

10M
Conc 65,5934
Area 181031
R#1 194,90>109,90 38,46 (31,33)
R#2 194,90>42,00 70,16 (53,07)
Q 194,90>137,95 (+) 3,47e4

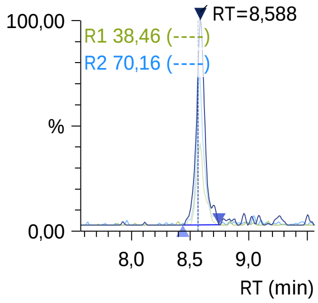

9H
Conc 18,6271
Area 42391
R#1 194,90>109,90 20,88 (31,33)
R#2 194,90>42,00 64,71 (53,07)
Q 194,90>137,95 (+) 5,72e3

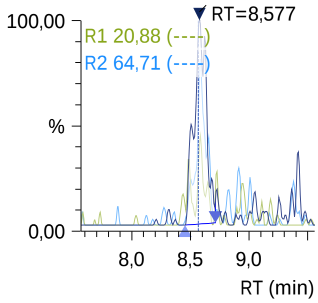

9M
Conc 44,1575
Area 117754
R#1 194,90>109,90 36,02 (31,33)
R#2 194,90>42,00 63,55 (53,07)
Q 194,90>137,95 (+) 2,54e4

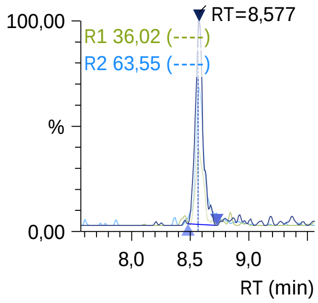

Compound: Ac. Nicotinico

Curve Fit: Linear | Weighting: None | Zero: Not Forced
Quantitative Method: External Standard
Q 123,90>79,85
R^2 = 0.9933527 R = 0.9966708
y = 3423,454x - 36799,78

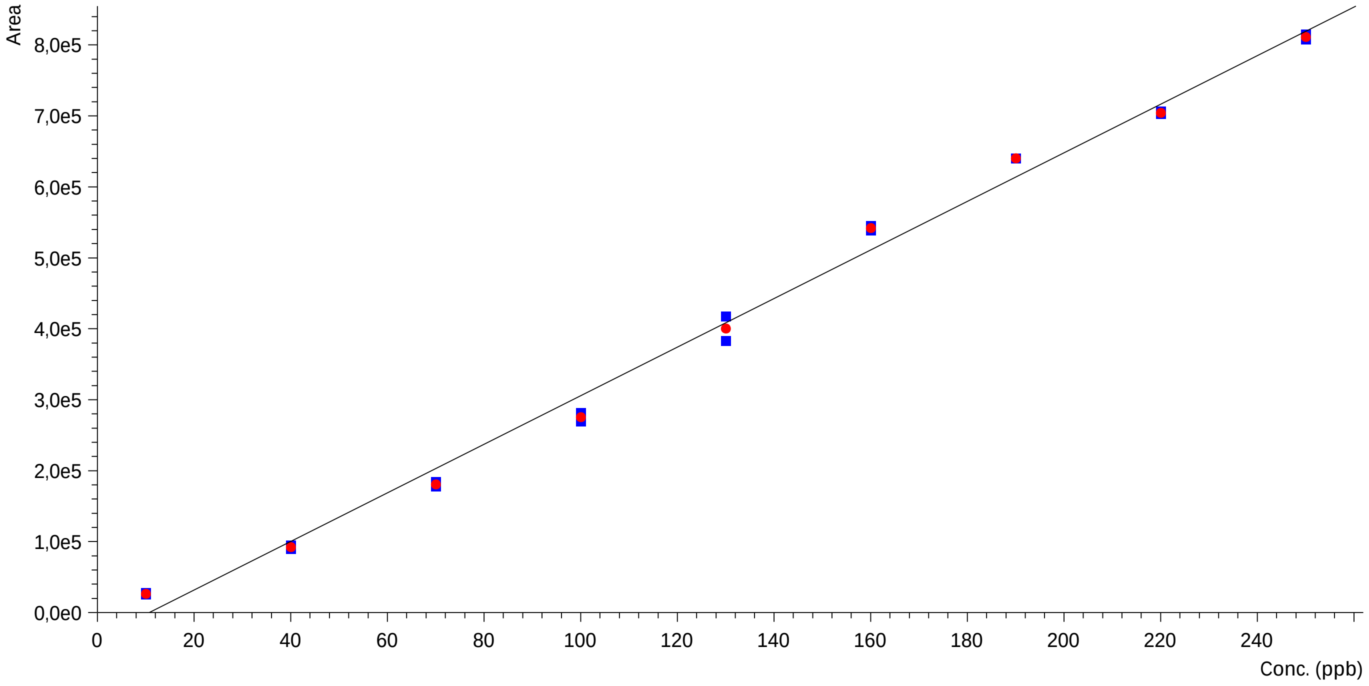

Sample ID	Area	Conc.	Sample Type	Level	Cal Point	Found RT	Ref 1 Actual Ratio
0	----	----	Ctrl.	0	----	----	----
10ppb001	27548	18,7962	Std.	1	Yes	4,572	51,72
10 ppb002	25821	18,2917	Std.	1	Yes	4,588	53,75
40 ppb001	89830	36,9888	Std.	2	Yes	4,579	62,82
40ppb002	94661	38,4001	Std.	2	Yes	4,587	74,13
70 ppb001	177663	62,6451	Std.	3	Yes	4,581	63,58
70ppb002	184256	64,5709	Std.	3	Yes	4,598	70,05
100 ppb001	269299	89,4124	Std.	4	Yes	4,607	67,68
100ppb002	281577	92,9988	Std.	4	Yes	4,607	66,29
130 ppb001	382941	122,6073	Std.	5	Yes	4,600	99,18
130ppb002	417702	132,7610	Std.	5	Yes	4,574	85,20
160 ppb001	538685	168,1005	Std.	6	Yes	4,596	79,48
160ppb002	545349	170,0471	Std.	6	Yes	4,607	75,75
190 ppb001	640400	197,8120	Std.	7	Yes	4,589	76,64
190ppb002	640248	197,7674	Std.	7	Yes	4,602	75,14
220 ppb001	703017	216,1026	Std.	8	Yes	4,592	82,58
220ppb002	706243	217,0449	Std.	8	Yes	4,602	77,21
250 ppb001	807955	246,7551	Std.	9	Yes	4,605	80,26
250 ppb002	815292	248,8982	Std.	9	Yes	4,646	68,69
10H	218646	74,6163	Unk.	----	----	4,657	49,81
10M	239258	80,6373	Unk.	----	----	4,642	56,51
9H	1823961	543,5333	Unk.	----	----	4,648	60,47
9M	1604770	479,5068	Unk.	----	----	4,645	55,36
Average	510687	159,9224				4,606	69,65
%RSD	91	84,9693				0,554	17,76
Maximum	1823961	543,5333				4,657	99,18
Minimum	25821	18,2917				4,572	49,81
Std. Dev.	465196	135,8850				0,026	12,37

Compound: Ac. Nicotinico (continued)

0
Conc ----
Area ----
R#1 123,90>77,90 ---- (5,61)
R#2 123,90>52,85 ---- (0,00)
Q 123,90>79,85 (+) 1,87e3

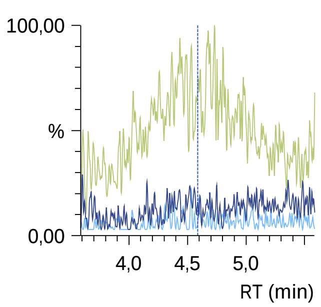

10ppb001
Conc 18,7962
Area 27548
R#1 123,90>77,90 51,72 (5,61)
R#2 123,90>52,85 39,75 (0,00)
Q 123,90>79,85 (+) 4,91e3

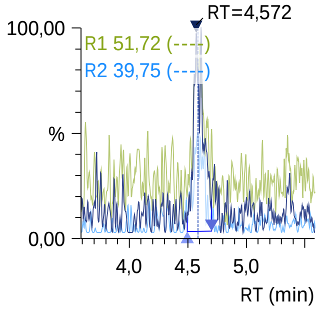

10 ppb002
Conc 18,2917
Area 25821
R#1 123,90>77,90 53,75 (5,61)
R#2 123,90>52,85 20,62 (0,00)
Q 123,90>79,85 (+) 4,98e3

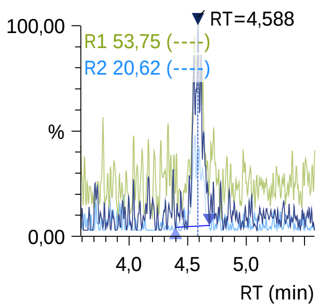

40 ppb001
Conc 36,9888
Area 89830
R#1 123,90>77,90 62,82 (5,61)
R#2 123,90>52,85 73,52 (0,00)
Q 123,90>79,85 (+) 1,53e4

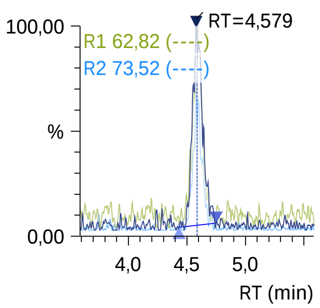

40ppb002
Conc 38,4001
Area 94661
R#1 123,90>77,90 74,13 (5,61)
R#2 123,90>52,85 58,06 (0,00)
Q 123,90>79,85 (+) 1,64e4

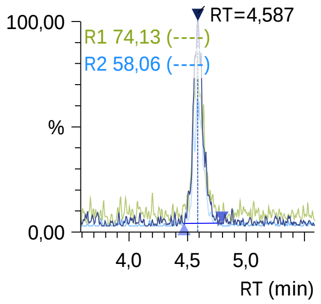

70 ppb001
Conc 62,6451
Area 177663
R#1 123,90>77,90 63,58 (5,61)
R#2 123,90>52,85 45,48 (0,00)
Q 123,90>79,85 (+) 2,95e4

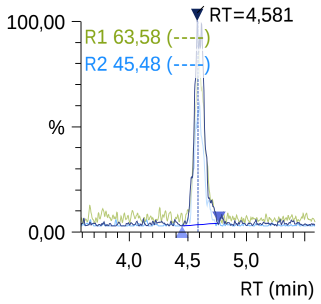

70ppb002
Conc 64,5709
Area 184256
R#1 123,90>77,90 70,05 (5,61)
R#2 123,90>52,85 56,33 (0,00)
Q 123,90>79,85 (+) 3,18e4

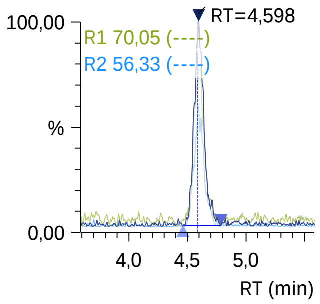

100 ppb001
Conc 89,4124
Area 269299
R#1 123,90>77,90 67,68 (5,61)
R#2 123,90>52,85 48,57 (0,00)
Q 123,90>79,85 (+) 4,66e4

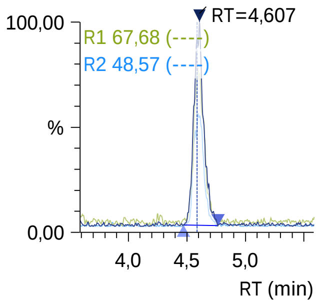

100ppb002
Conc 92,9988
Area 281577
R#1 123,90>77,90 66,29 (5,61)
R#2 123,90>52,85 50,88 (0,00)
Q 123,90>79,85 (+) 5,11e4

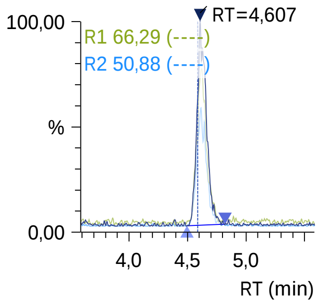

130 ppb001
Conc 122,6073
Area 382941
R#1 123,90>77,90 99,18 (5,61)
R#2 123,90>52,85 60,94 (0,00)
Q 123,90>79,85 (+) 6,07e4

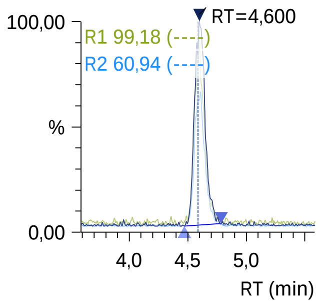

130ppb002
Conc 132,7610
Area 417702
R#1 123,90>77,90 85,20 (5,61)
R#2 123,90>52,85 58,08 (0,00)
Q 123,90>79,85 (+) 6,91e4

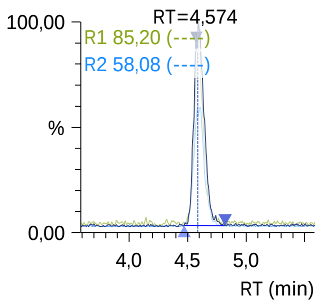

160 ppb001
Conc 168,1005
Area 538685
R#1 123,90>77,90 79,48 (5,61)
R#2 123,90>52,85 57,06 (0,00)
Q 123,90>79,85 (+) 9,49e4

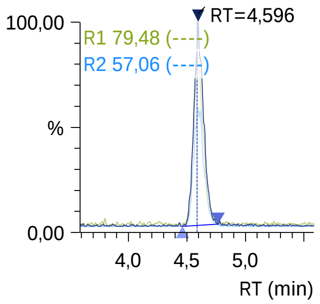

Compound: Ac. Nicotinico (continued)

160ppb002
Conc 170,0471
Area 545349
R#1 123,90>77,90 75,75 (5,61)
R#2 123,90>52,85 65,10 (0,00)
Q 123,90>79,85 (+) 9,23e4

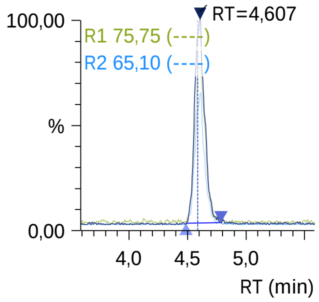

190 ppb001
Conc 197,8120
Area 640400
R#1 123,90>77,90 76,64 (5,61)
R#2 123,90>52,85 47,46 (0,00)
Q 123,90>79,85 (+) 1,12e5

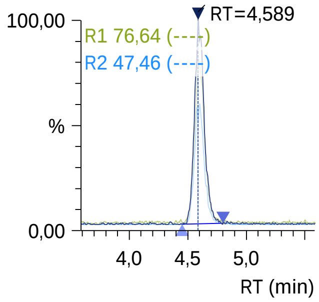

190ppb002
Conc 197,7674
Area 640248
R#1 123,90>77,90 75,14 (5,61)
R#2 123,90>52,85 58,19 (0,00)
Q 123,90>79,85 (+) 1,09e5

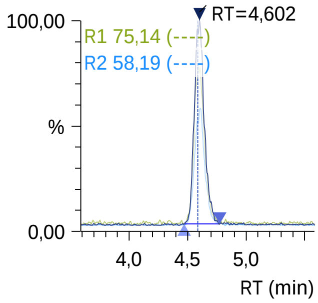

220 ppb001
Conc 216,1026
Area 703017
R#1 123,90>77,90 82,58 (5,61)
R#2 123,90>52,85 58,03 (0,00)
Q 123,90>79,85 (+) 1,14e5

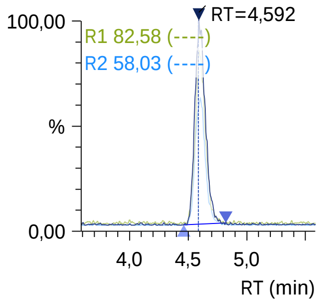

220ppb002
Conc 217,0449
Area 706243
R#1 123,90>77,90 77,21 (5,61)
R#2 123,90>52,85 64,75 (0,00)
Q 123,90>79,85 (+) 1,19e5

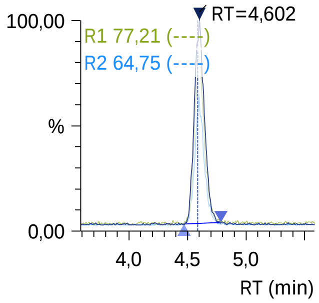

250 ppb001
Conc 246,7551
Area 807955
R#1 123,90>77,90 80,26 (5,61)
R#2 123,90>52,85 58,60 (0,00)
Q 123,90>79,85 (+) 1,33e5

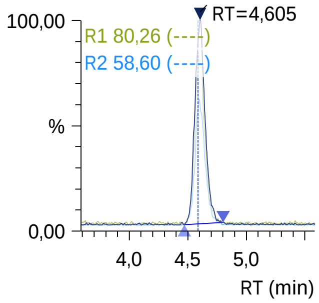

250 ppb002
Conc 248,8982
Area 815292
R#1 123,90>77,90 68,69 (5,61)
R#2 123,90>52,85 55,28 (0,00)
Q 123,90>79,85 (+) 1,51e5

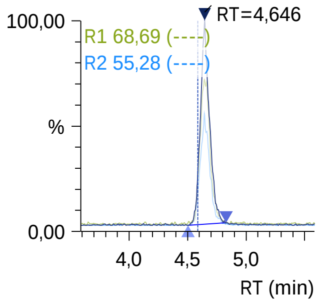

10H
Conc 74,6163
Area 218646
R#1 123,90>77,90 49,81 (5,61)
R#2 123,90>52,85 49,93 (0,00)
Q 123,90>79,85 (+) 3,92e4

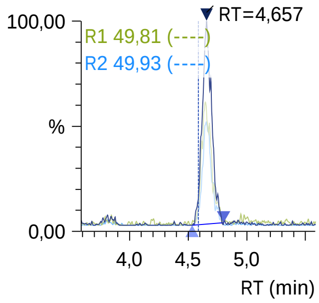

10M
Conc 80,6373
Area 239258
R#1 123,90>77,90 56,51 (5,61)
R#2 123,90>52,85 43,94 (0,00)
Q 123,90>79,85 (+) 4,40e4

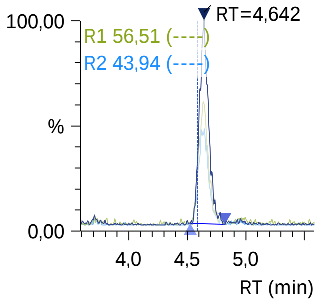

9H
Conc 543,5333
Area 1823961
R#1 123,90>77,90 60,47 (5,61)
R#2 123,90>52,85 50,30 (0,00)
Q 123,90>79,85 (+) 3,12e5

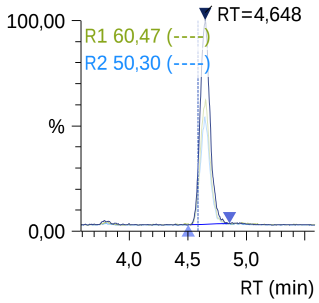

9M
Conc 479,5068
Area 1604770
R#1 123,90>77,90 55,36 (5,61)
R#2 123,90>52,85 45,09 (0,00)
Q 123,90>79,85 (+) 2,69e5

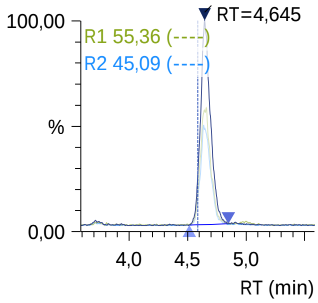

Compound: Catecol

Curve Fit: Linear | Weighting: None | Zero: Not Forced
Quantitative Method: External Standard
Q 109,40>90,90
R^2 = 0.9805841 R = 0.9902445
y = 73,18593x - 913,5105

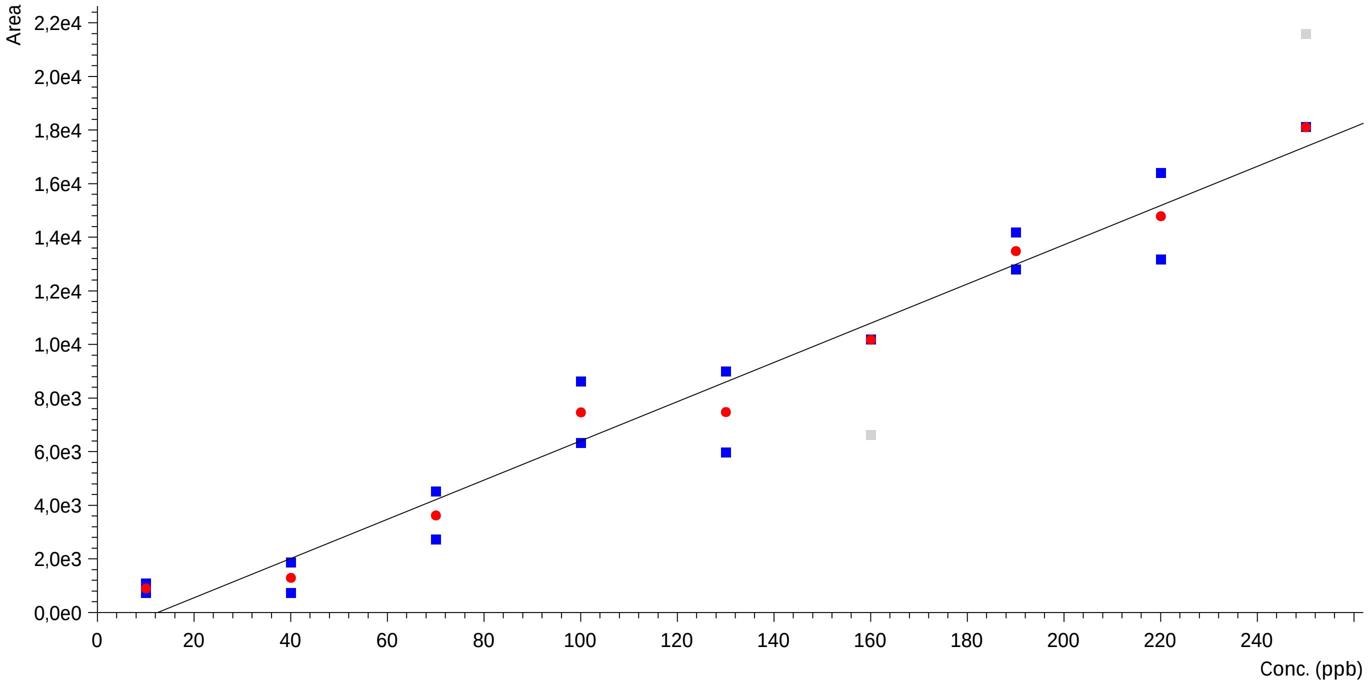

Sample ID	Area	Conc.	Sample Type	Level	Cal Point	Found RT	Ref 1 Actual Ratio
0	----	----	Ctrl.	0	----	----	----
10ppb001	1089	27,3662	Std.	1	Yes	7,959	0,00
10 ppb002	733	22,5029	Std.	1	Yes	7,966	0,00
40 ppb001	742	22,6216	Std.	2	Yes	7,994	76,76
40ppb002	1871	38,0436	Std.	2	Yes	7,978	47,06
70 ppb001	2738	49,8957	Std.	3	Yes	7,977	68,89
70ppb002	4523	74,2805	Std.	3	Yes	7,986	34,84
100 ppb001	8617	130,2207	Std.	4	Yes	7,974	28,76
100ppb002	6331	98,9858	Std.	4	Yes	7,990	74,15
130 ppb001	5972	94,0844	Std.	5	Yes	7,998	33,00
130ppb002	8997	135,4138	Std.	5	Yes	7,988	30,47
160 ppb001	6641	103,2216	Std.	6	No	7,994	66,63
160ppb002	10191	151,7244	Std.	6	Yes	7,987	25,50
190 ppb001	14179	206,2256	Std.	7	Yes	7,990	35,80
190ppb002	12815	187,5795	Std.	7	Yes	7,981	87,90
220 ppb001	16410	236,7109	Std.	8	Yes	7,984	83,84
220ppb002	13185	192,6404	Std.	8	Yes	7,986	59,56
250 ppb001	21584	307,4028	Std.	9	No	7,982	48,61
250 ppb002	18114	259,9897	Std.	9	Yes	7,979	42,33
10H	----	----	Unk.	----	----	----	----
10M	----	----	Unk.	----	----	----	----
9H	----	----	Unk.	----	----	----	----
9M	----	----	Unk.	----	----	----	----
Average	8596	129,9395				7,983	46,89
%RSD	74	66,7457				0,123	55,84
Maximum	21584	307,4028				7,998	87,90
Minimum	733	22,5029				7,959	0,00
Std. Dev.	6347	86,7291				0,010	26,18

Compound: Catecol (continued)

0
Conc ----
Area ----
R#1 109,40>80,95 ---- (42,33)
R#2 109,40>64,85 ---- (60,43)
Q 109,40>90,90 (-) 0,00e0

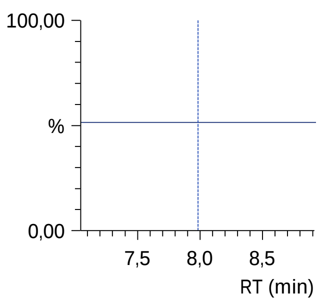

10ppb001
Conc 27,3662
Area 1089
R#1 109,40>80,95 0,00 (42,33)
R#2 109,40>64,85 0,00 (60,43)
Q 109,40>90,90 (-) 7,33e2

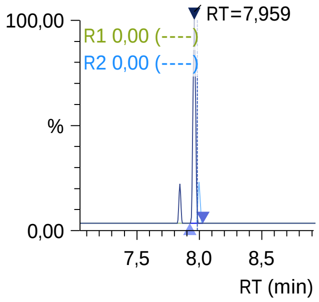

10 ppb002
Conc 22,5029
Area 733
R#1 109,40>80,95 0,00 (42,33)
R#2 109,40>64,85 0,00 (60,43)
Q 109,40>90,90 (-) 4,41e2

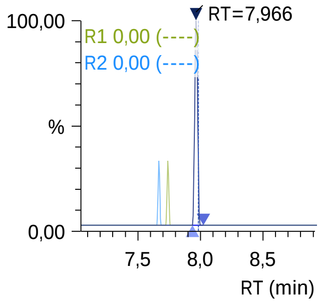

40 ppb001
Conc 22,6216
Area 742
R#1 109,40>80,95 76,76 (42,33)
R#2 109,40>64,85 0,00 (60,43)
Q 109,40>90,90 (-) 2,60e2

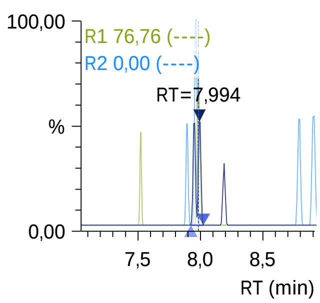

40ppb002
Conc 38,0436
Area 1871
R#1 109,40>80,95 47,06 (42,33)
R#2 109,40>64,85 76,31 (60,43)
Q 109,40>90,90 (-) 6,47e2

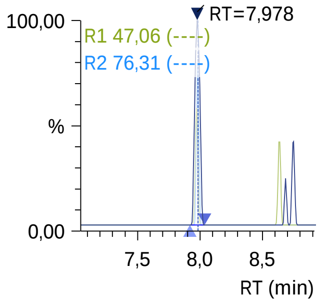

70 ppb001
Conc 49,8957
Area 2738
R#1 109,40>80,95 68,89 (42,33)
R#2 109,40>64,85 50,13 (60,43)
Q 109,40>90,90 (-) 1,23e3

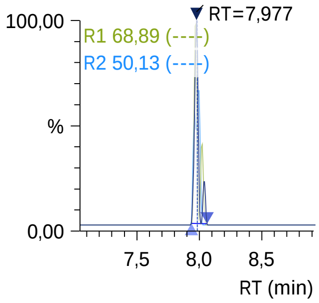

70ppb002
Conc 74,2805
Area 4523
R#1 109,40>80,95 34,84 (42,33)
R#2 109,40>64,85 8,35 (60,43)
Q 109,40>90,90 (-) 1,39e3

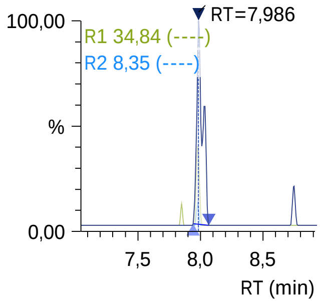

100 ppb001
Conc 130,2207
Area 8617
R#1 109,40>80,95 28,76 (42,33)
R#2 109,40>64,85 35,64 (60,43)
Q 109,40>90,90 (-) 2,76e3

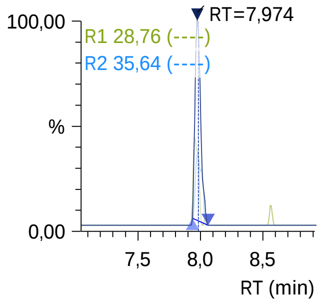

100ppb002
Conc 98,9858
Area 6331
R#1 109,40>80,95 74,15 (42,33)
R#2 109,40>64,85 52,08 (60,43)
Q 109,40>90,90 (-) 2,20e3

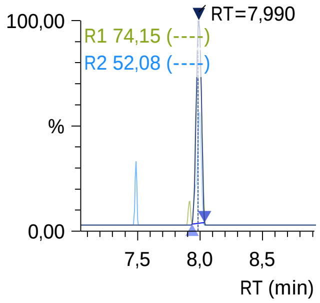

130 ppb001
Conc 94,0844
Area 5972
R#1 109,40>80,95 33,00 (42,33)
R#2 109,40>64,85 14,18 (60,43)
Q 109,40>90,90 (-) 1,73e3

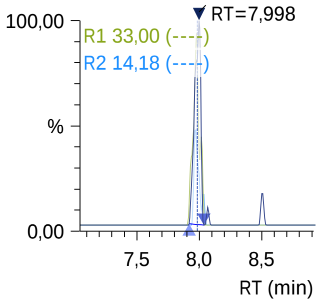

130ppb002
Conc 135,4138
Area 8997
R#1 109,40>80,95 30,47 (42,33)
R#2 109,40>64,85 22,76 (60,43)
Q 109,40>90,90 (-) 3,55e3

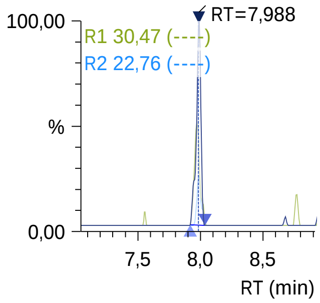

160 ppb001
Conc 103,2216
Area 6641
R#1 109,40>80,95 66,63 (42,33)
R#2 109,40>64,85 18,77 (60,43)
Q 109,40>90,90 (-) 2,05e3

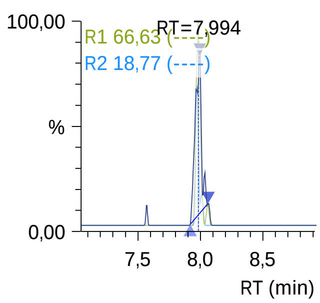

Compound: Catecol (continued)

160ppb002
Conc 151,7244
Area 10191
R#1 109,40>80,95 25,50 (42,33)
R#2 109,40>64,85 50,90 (60,43)
Q 109,40>90,90 (-) 3,17e3

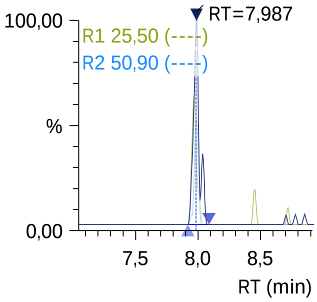

190 ppb001
Conc 206,2256
Area 14179
R#1 109,40>80,95 35,80 (42,33)
R#2 109,40>64,85 41,64 (60,43)
Q 109,40>90,90 (-) 5,27e3

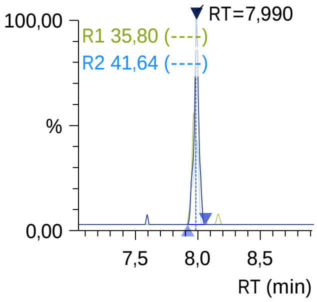

190ppb002
Conc 187,5795
Area 12815
R#1 109,40>80,95 87,90 (42,33)
R#2 109,40>64,85 58,38 (60,43)
Q 109,40>90,90 (-) 4,12e3

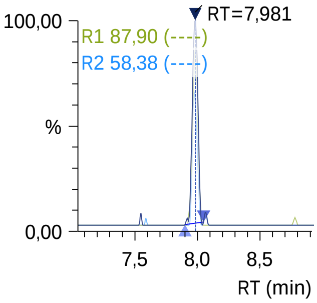

220 ppb001
Conc 236,7109
Area 16410
R#1 109,40>80,95 83,84 (42,33)
R#2 109,40>64,85 52,71 (60,43)
Q 109,40>90,90 (-) 4,42e3

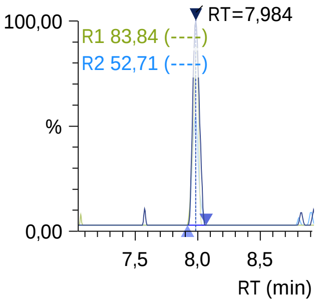

220ppb002
Conc 192,6404
Area 13185
R#1 109,40>80,95 59,56 (42,33)
R#2 109,40>64,85 54,51 (60,43)
Q 109,40>90,90 (-) 4,64e3

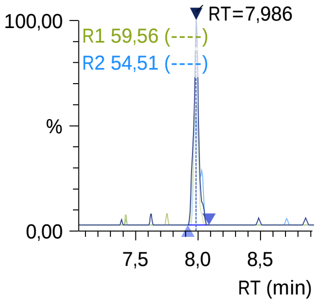

250 ppb001
Conc 307,4028
Area 21584
R#1 109,40>80,95 48,61 (42,33)
R#2 109,40>64,85 23,44 (60,43)
Q 109,40>90,90 (-) 5,71e3

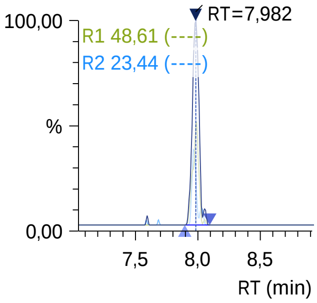

250 ppb002
Conc 259,9897
Area 18114
R#1 109,40>80,95 42,33 (42,33)
R#2 109,40>64,85 60,43 (60,43)
Q 109,40>90,90 (-) 4,68e3

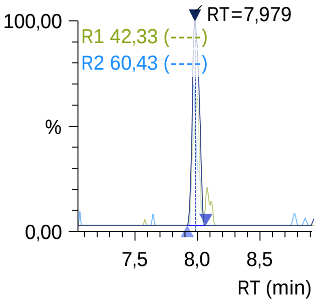

10H
Conc ----
Area ----
R#1 109,40>80,95 ---- (42,33)
R#2 109,40>64,85 ---- (60,43)
Q 109,40>90,90 (-) 0,00e0

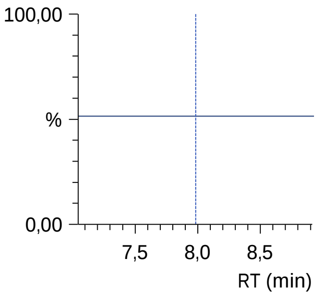

10M
Conc ----
Area ----
R#1 109,40>80,95 ---- (42,33)
R#2 109,40>64,85 ---- (60,43)
Q 109,40>90,90 (-) 0,00e0

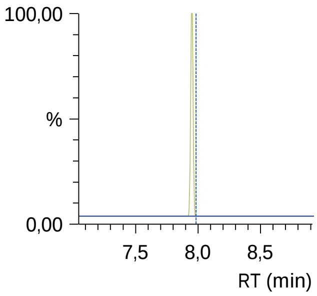

9H
Conc ----
Area ----
R#1 109,40>80,95 ---- (42,33)
R#2 109,40>64,85 ---- (60,43)
Q 109,40>90,90 (-) 0,00e0

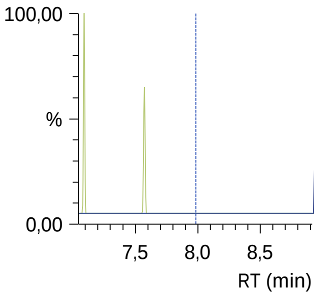

9M
Conc ----
Area ----
R#1 109,40>80,95 ---- (42,33)
R#2 109,40>64,85 ---- (60,43)
Q 109,40>90,90 (-) 2,77e2

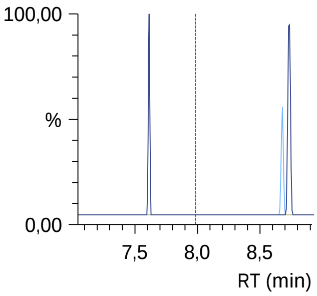

Compound: Ac. Galico

Curve Fit: Linear | Weighting: None | Zero: Not Forced
Quantitative Method: External Standard
Q 169,20>124,90
R^2 = 0.9976334 R = 0.9988160
y = 1243,611x - 6489,515

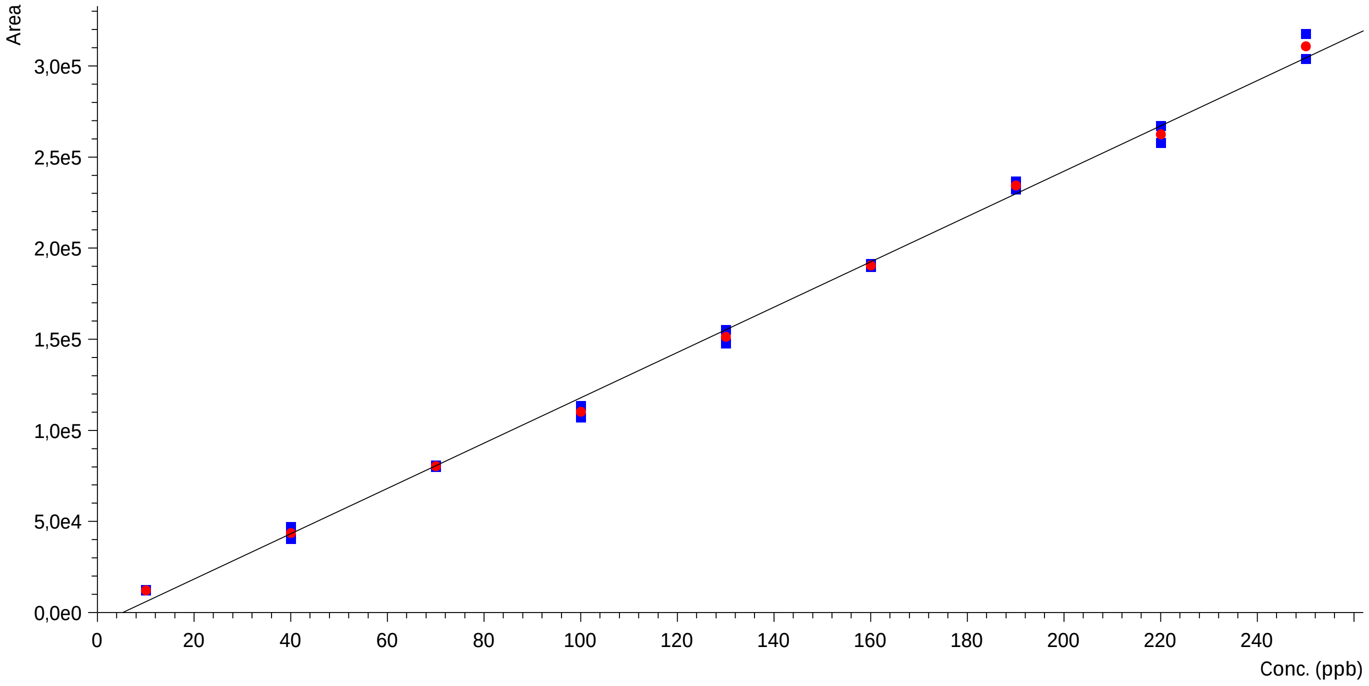

Sample ID	Area	Conc.	Sample Type	Level	Cal Point	Found RT	Ref 1 Actual Ratio
0	----	----	Ctrl.	0	----	----	----
10ppb001	12491	15,2622	Std.	1	Yes	6,584	0,00
10 ppb002	12132	14,9737	Std.	1	Yes	6,574	0,00
40 ppb001	47041	43,0445	Std.	2	Yes	6,577	0,00
40ppb002	40464	37,7555	Std.	2	Yes	6,563	0,00
70 ppb001	80706	70,1146	Std.	3	Yes	6,587	0,00
70ppb002	80136	69,6567	Std.	3	Yes	6,579	0,00
100 ppb001	113527	96,5061	Std.	4	Yes	6,580	0,00
100ppb002	107206	91,4235	Std.	4	Yes	6,600	0,23
130 ppb001	147799	124,0653	Std.	5	Yes	6,584	0,00
130ppb002	155108	129,9419	Std.	5	Yes	6,582	0,00
160 ppb001	189802	157,8400	Std.	6	Yes	6,585	0,67
160ppb002	191329	159,0675	Std.	6	Yes	6,588	0,00
190 ppb001	236525	195,4104	Std.	7	Yes	6,589	0,03
190ppb002	232313	192,0235	Std.	7	Yes	6,578	0,32
220 ppb001	257895	212,5941	Std.	8	Yes	6,577	0,26
220ppb002	267135	220,0242	Std.	8	Yes	6,592	0,03
250 ppb001	317558	260,5700	Std.	9	Yes	6,578	0,35
250 ppb002	304073	249,7264	Std.	9	Yes	6,611	0,00
10H	----	----	Unk.	----	----	----	----
10M	----	----	Unk.	----	----	----	----
9H	----	----	Unk.	----	----	----	----
9M	----	----	Unk.	----	----	----	----
Average	155180	130,0000				6,584	0,11
%RSD	64	61,4234				0,158	178,41
Maximum	317558	260,5700				6,611	0,67
Minimum	12132	14,9737				6,563	0,00
Std. Dev.	99303	79,8504				0,010	0,19

Compound: Ac. Galico (continued)

0
Conc ----
Area ----
R#1 169,20>49,00 ---- (0,35)
R#2 169,20>78,85 ---- (10,40)
Q 169,20>124,90 (-) 1,72e3

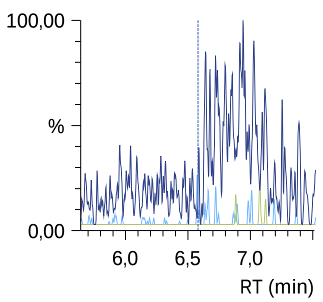

10ppb001
Conc 15,2622
Area 12491
R#1 169,20>49,00 0,00 (0,35)
R#2 169,20>78,85 0,00 (10,40)
Q 169,20>124,90 (-) 3,79e3

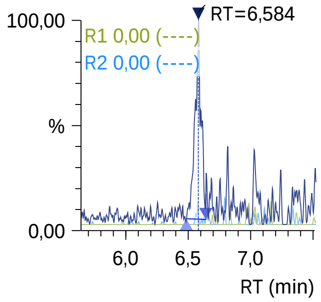

10 ppb002
Conc 14,9737
Area 12132
R#1 169,20>49,00 0,00 (0,35)
R#2 169,20>78,85 13,76 (10,40)
Q 169,20>124,90 (-) 3,00e3

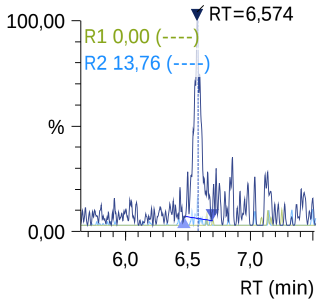

40 ppb001
Conc 43,0445
Area 47041
R#1 169,20>49,00 0,00 (0,35)
R#2 169,20>78,85 5,81 (10,40)
Q 169,20>124,90 (-) 1,04e4

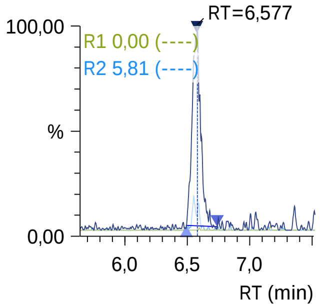

40ppb002
Conc 37,7555
Area 40464
R#1 169,20>49,00 0,00 (0,35)
R#2 169,20>78,85 11,96 (10,40)
Q 169,20>124,90 (-) 8,82e3

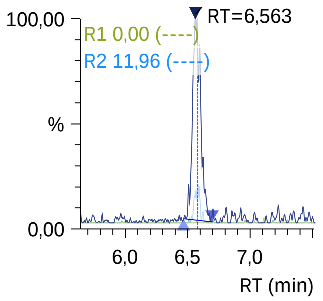

70 ppb001
Conc 70,1146
Area 80706
R#1 169,20>49,00 0,00 (0,35)
R#2 169,20>78,85 8,47 (10,40)
Q 169,20>124,90 (-) 1,89e4

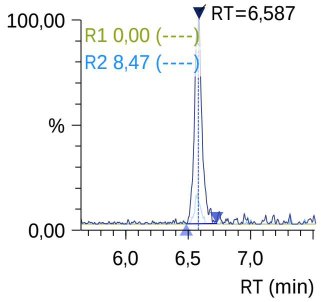

70ppb002
Conc 69,6567
Area 80136
R#1 169,20>49,00 0,00 (0,35)
R#2 169,20>78,85 12,10 (10,40)
Q 169,20>124,90 (-) 1,80e4

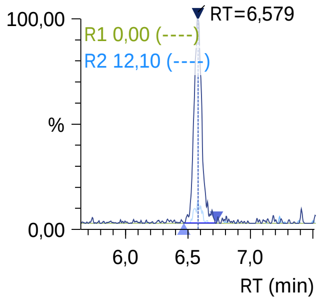

100 ppb001
Conc 96,5061
Area 113527
R#1 169,20>49,00 0,00 (0,35)
R#2 169,20>78,85 14,98 (10,40)
Q 169,20>124,90 (-) 2,57e4

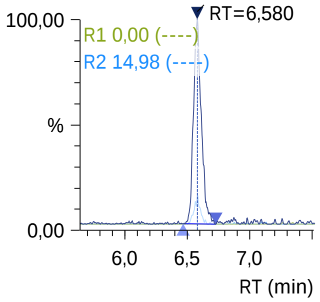

100ppb002
Conc 91,4235
Area 107206
R#1 169,20>49,00 0,23 (0,35)
R#2 169,20>78,85 11,42 (10,40)
Q 169,20>124,90 (-) 2,48e4

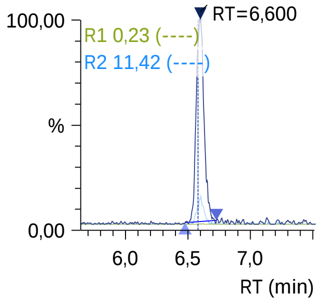

130 ppb001
Conc 124,0653
Area 147799
R#1 169,20>49,00 0,00 (0,35)
R#2 169,20>78,85 10,24 (10,40)
Q 169,20>124,90 (-) 3,39e4

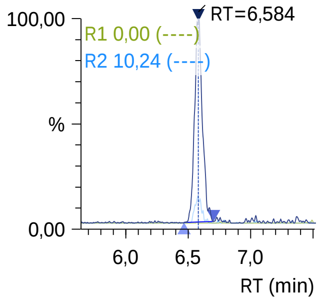

130ppb002
Conc 129,9419
Area 155108
R#1 169,20>49,00 0,00 (0,35)
R#2 169,20>78,85 9,50 (10,40)
Q 169,20>124,90 (-) 3,78e4

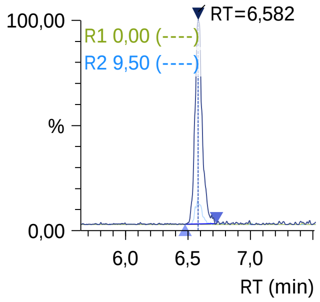

160 ppb001
Conc 157,8400
Area 189802
R#1 169,20>49,00 0,67 (0,35)
R#2 169,20>78,85 16,65 (10,40)
Q 169,20>124,90 (-) 4,17e4

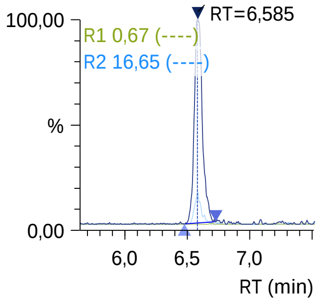

Compound: Ac. Galico (continued)

160ppb002
Conc 159,0675
Area 191329
R#1 169,20>49,00 0,00 (0,35)
R#2 169,20>78,85 18,02 (10,40)
Q 169,20>124,90 (-) 4,51e4

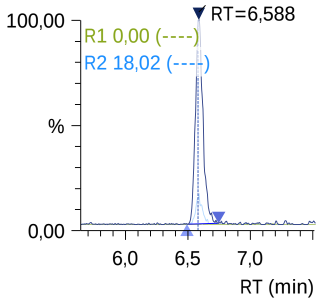

190 ppb001
Conc 195,4104
Area 236525
R#1 169,20>49,00 0,03 (0,35)
R#2 169,20>78,85 10,65 (10,40)
Q 169,20>124,90 (-) 5,64e4

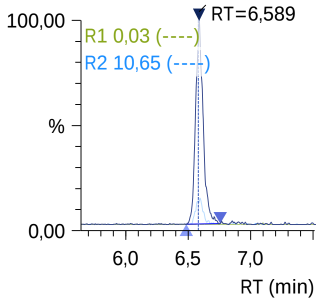

190ppb002
Conc 192,0235
Area 232313
R#1 169,20>49,00 0,32 (0,35)
R#2 169,20>78,85 11,55 (10,40)
Q 169,20>124,90 (-) 5,45e4

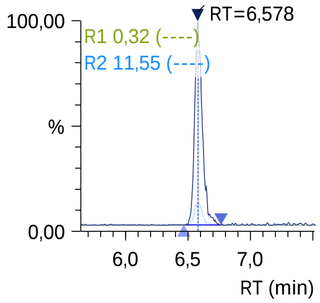

220 ppb001
Conc 212,5941
Area 257895
R#1 169,20>49,00 0,26 (0,35)
R#2 169,20>78,85 14,73 (10,40)
Q 169,20>124,90 (-) 5,75e4

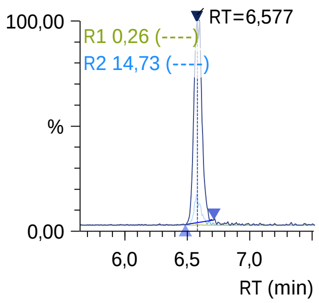

220ppb002
Conc 220,0242
Area 267135
R#1 169,20>49,00 0,03 (0,35)
R#2 169,20>78,85 11,41 (10,40)
Q 169,20>124,90 (-) 6,12e4

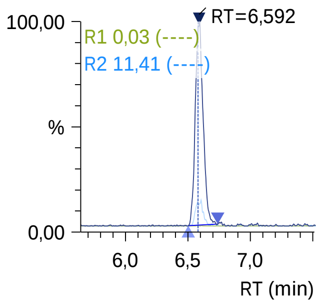

250 ppb001
Conc 260,5700
Area 317558
R#1 169,20>49,00 0,35 (0,35)
R#2 169,20>78,85 10,40 (10,40)
Q 169,20>124,90 (-) 7,47e4

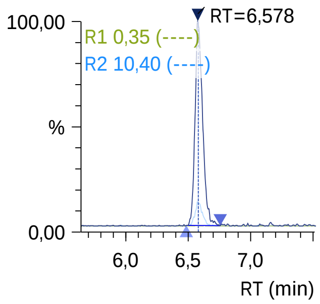

250 ppb002
Conc 249,7264
Area 304073
R#1 169,20>49,00 0,00 (0,35)
R#2 169,20>78,85 11,64 (10,40)
Q 169,20>124,90 (-) 7,28e4

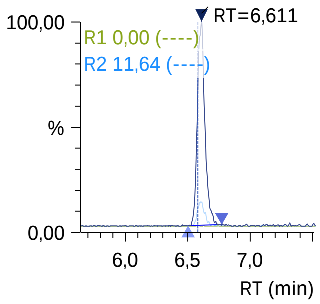

10H
Conc ----
Area ----
R#1 169,20>49,00 ---- (0,35)
R#2 169,20>78,85 ---- (10,40)
Q 169,20>124,90 (-) 1,03e4

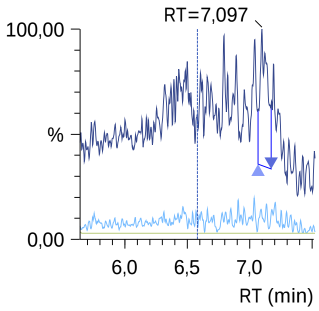

10M
Conc ----
Area ----
R#1 169,20>49,00 ---- (0,35)
R#2 169,20>78,85 ---- (10,40)
Q 169,20>124,90 (-) 1,12e4

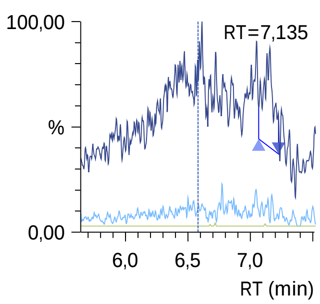

9H
Conc ----
Area ----
R#1 169,20>49,00 ---- (0,35)
R#2 169,20>78,85 ---- (10,40)
Q 169,20>124,90 (-) 1,71e4

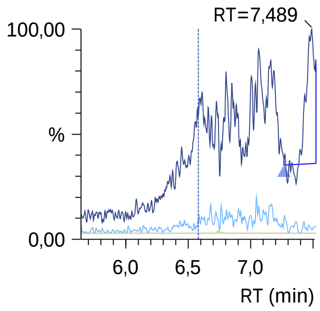

9M
Conc ----
Area ----
R#1 169,20>49,00 ---- (0,35)
R#2 169,20>78,85 ---- (10,40)
Q 169,20>124,90 (-) 1,19e4

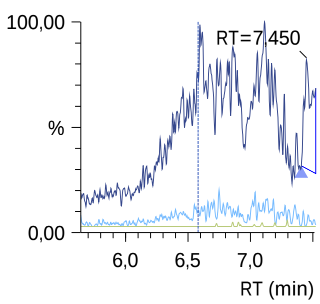

Compound: Quercetina

Curve Fit: Linear | Weighting: None | Zero: Not Forced
Quantitative Method: External Standard
Q 301,10>151,00
R^2 = 0.9507576 R = 0.9750680
y = 5080,717x + 119391,4

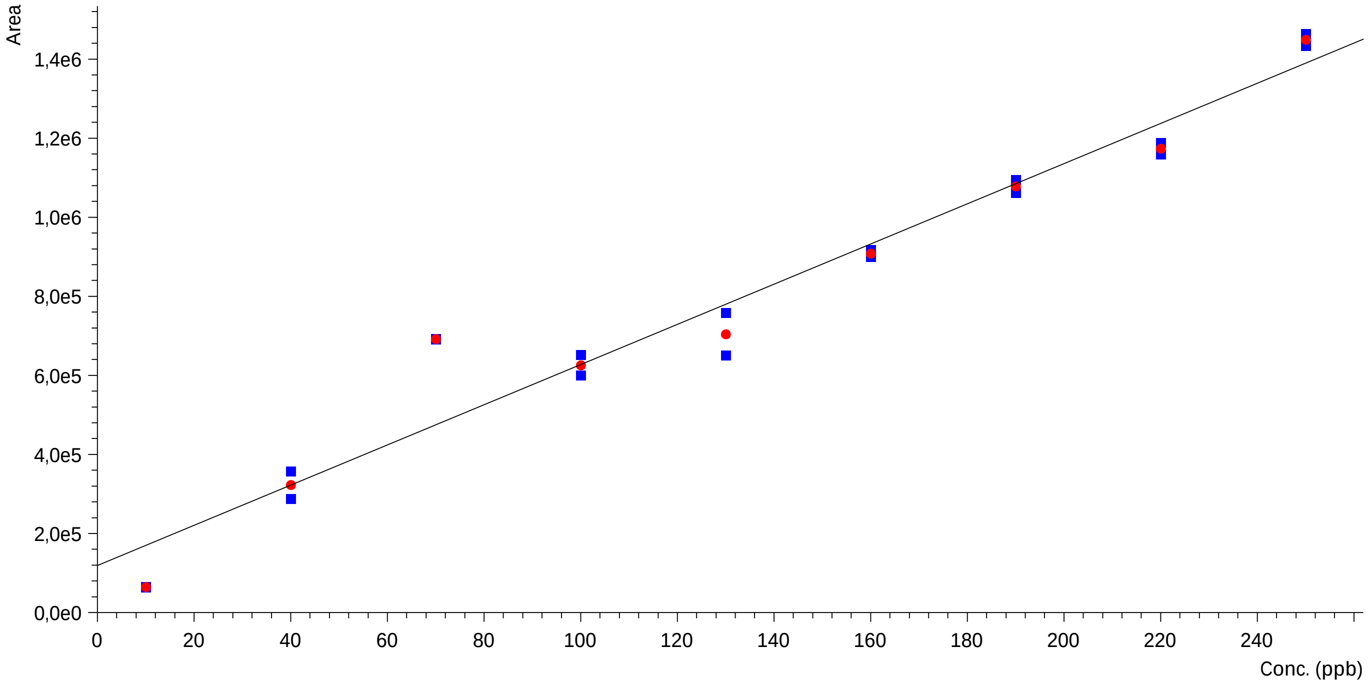

Sample ID	Area	Conc.	Sample Type	Level	Cal Point	Found RT	Ref 1 Actual Ratio
0	15054	-20,5360	Ctrl.	0	----	9,733	25,04
10ppb001	65079	-10,6900	Std.	1	Yes	9,712	39,46
10 ppb002	63724	-10,9567	Std.	1	Yes	9,726	50,31
40 ppb001	288462	33,2770	Std.	2	Yes	9,716	47,60
40ppb002	358032	46,9698	Std.	2	Yes	9,720	47,77
70 ppb001	691072	112,5196	Std.	3	Yes	9,725	50,18
70ppb002	693136	112,9259	Std.	3	Yes	9,720	48,72
100 ppb001	651725	104,7753	Std.	4	Yes	9,724	54,84
100ppb002	599924	94,5796	Std.	4	Yes	9,719	50,20
130 ppb001	650228	104,4807	Std.	5	Yes	9,717	49,94
130ppb002	757921	125,6770	Std.	5	Yes	9,719	48,33
160 ppb001	899079	153,4601	Std.	6	Yes	9,716	49,43
160ppb002	916878	156,9634	Std.	6	Yes	9,712	46,87
190 ppb001	1061767	185,4808	Std.	7	Yes	9,719	46,46
190ppb002	1094775	191,9776	Std.	7	Yes	9,709	54,81
220 ppb001	1159663	204,7489	Std.	8	Yes	9,710	49,42
220ppb002	1188531	210,4309	Std.	8	Yes	9,717	56,33
250 ppb001	1433870	258,7192	Std.	9	Yes	9,709	55,88
250 ppb002	1464058	264,6608	Std.	9	Yes	9,715	49,50
10H	----	----	Unk.	----	----	----	----
10M	----	----	Unk.	----	----	----	----
9H	----	----	Unk.	----	----	----	----
9M	----	----	Unk.	----	----	----	----
Average	739630	122,0771				9,718	48,48
%RSD	60	71,0704				0,064	14,17
Maximum	1464058	264,6608				9,733	56,33
Minimum	15054	-20,5360				9,709	25,04
Std. Dev.	440807	86,7607				0,006	6,87

Compound: Quercetina (continued)

0
Conc -20,5360
Area 15054
R#1 301,10>179,05 25,04 (46,87)
R#2 301,10>106,85 29,21 (22,82)
Q 301,10>151,00 (-) 2,72e3

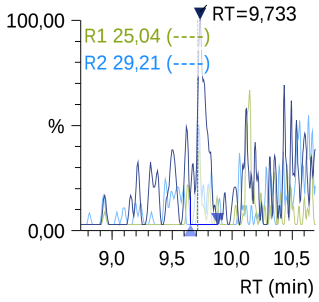

10ppb001
Conc -10,6900
Area 65079
R#1 301,10>179,05 39,46 (46,87)
R#2 301,10>106,85 20,35 (22,82)
Q 301,10>151,00 (-) 1,59e4

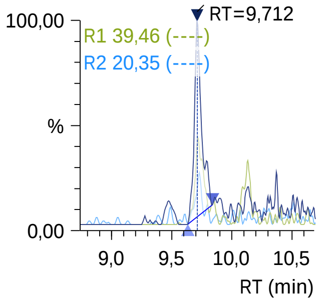

10 ppb002
Conc -10,9567
Area 63724
R#1 301,10>179,05 50,31 (46,87)
R#2 301,10>106,85 26,31 (22,82)
Q 301,10>151,00 (-) 1,30e4

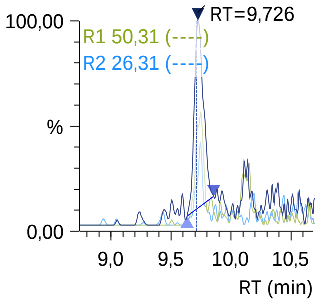

40 ppb001
Conc 33,2770
Area 288462
R#1 301,10>179,05 47,60 (46,87)
R#2 301,10>106,85 27,20 (22,82)
Q 301,10>151,00 (-) 6,14e4

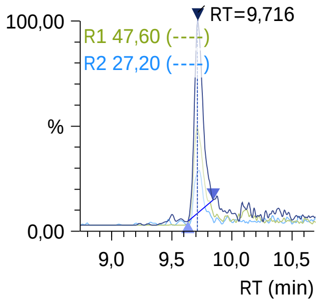

40ppb002
Conc 46,9698
Area 358032
R#1 301,10>179,05 47,77 (46,87)
R#2 301,10>106,85 23,73 (22,82)
Q 301,10>151,00 (-) 6,86e4

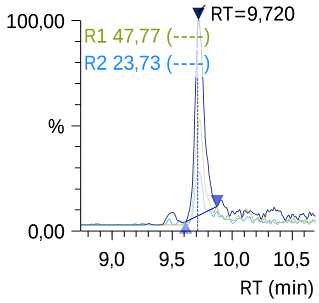

70 ppb001
Conc 112,5196
Area 691072
R#1 301,10>179,05 50,18 (46,87)
R#2 301,10>106,85 23,19 (22,82)
Q 301,10>151,00 (-) 1,35e5

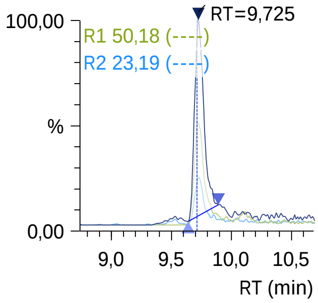

70ppb002
Conc 112,9259
Area 693136
R#1 301,10>179,05 48,72 (46,87)
R#2 301,10>106,85 24,29 (22,82)
Q 301,10>151,00 (-) 1,25e5

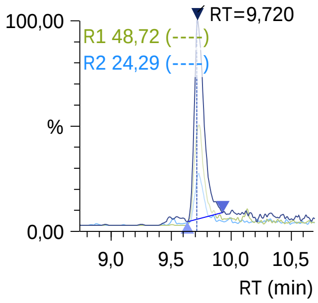

100 ppb001
Conc 104,7753
Area 651725
R#1 301,10>179,05 54,84 (46,87)
R#2 301,10>106,85 22,42 (22,82)
Q 301,10>151,00 (-) 1,30e5

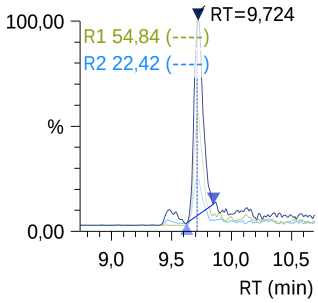

100ppb002
Conc 94,5796
Area 599924
R#1 301,10>179,05 50,20 (46,87)
R#2 301,10>106,85 22,45 (22,82)
Q 301,10>151,00 (-) 1,17e5

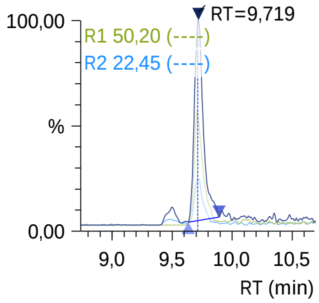

130 ppb001
Conc 104,4807
Area 650228
R#1 301,10>179,05 49,94 (46,87)
R#2 301,10>106,85 25,70 (22,82)
Q 301,10>151,00 (-) 1,48e5

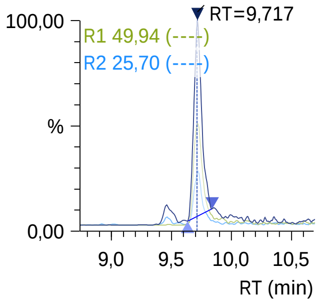

130ppb002
Conc 125,6770
Area 757921
R#1 301,10>179,05 48,33 (46,87)
R#2 301,10>106,85 23,46 (22,82)
Q 301,10>151,00 (-) 1,53e5

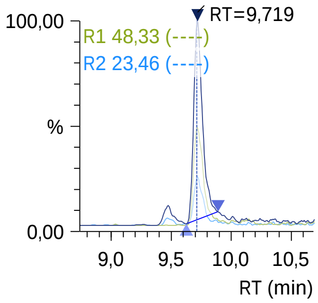

160 ppb001
Conc 153,4601
Area 899079
R#1 301,10>179,05 49,43 (46,87)
R#2 301,10>106,85 21,66 (22,82)
Q 301,10>151,00 (-) 1,89e5

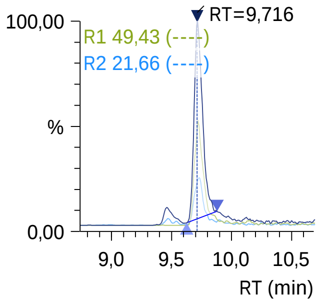

Compound: Quercetina (continued)

160ppb002

Conc 156,9634
Area 916878
R#1 301,10>179,05 46,87 (46,87)
R#2 301,10>106,85 22,82 (22,82)
Q 301,10>151,00 (-) 1,87e5

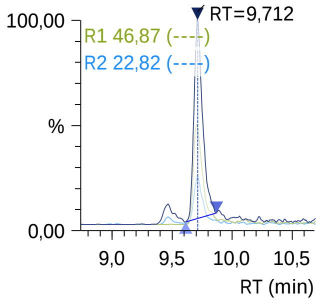

190 ppb001

Conc 185,4808
Area 1061767
R#1 301,10>179,05 46,46 (46,87)
R#2 301,10>106,85 20,73 (22,82)
Q 301,10>151,00 (-) 2,31e5

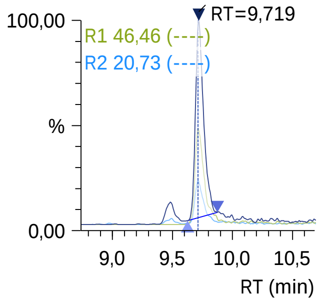

190ppb002

Conc 191,9776
Area 1094775
R#1 301,10>179,05 54,81 (46,87)
R#2 301,10>106,85 26,74 (22,82)
Q 301,10>151,00 (-) 2,20e5

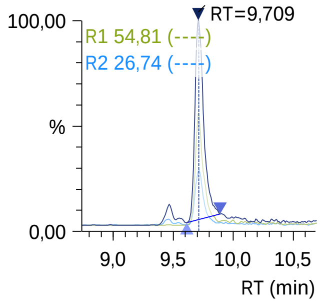

220 ppb001

Conc 204,7489
Area 1159663
R#1 301,10>179,05 49,42 (46,87)
R#2 301,10>106,85 24,85 (22,82)
Q 301,10>151,00 (-) 2,52e5

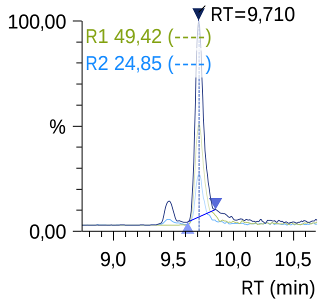

220ppb002

Conc 210,4309
Area 1188531
R#1 301,10>179,05 56,33 (46,87)
R#2 301,10>106,85 23,28 (22,82)
Q 301,10>151,00 (-) 2,63e5

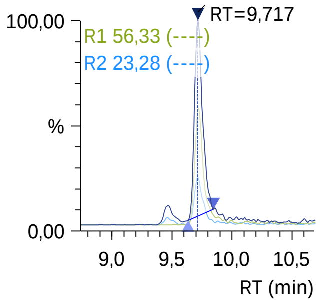

250 ppb001

Conc 258,7192
Area 1433870
R#1 301,10>179,05 55,88 (46,87)
R#2 301,10>106,85 25,15 (22,82)
Q 301,10>151,00 (-) 2,90e5

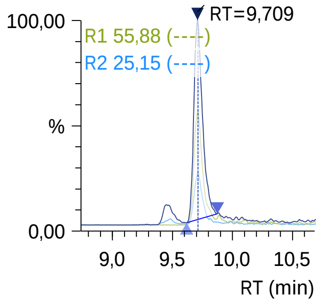

250 ppb002

Conc 264,6608
Area 1464058
R#1 301,10>179,05 49,50 (46,87)
R#2 301,10>106,85 20,75 (22,82)
Q 301,10>151,00 (-) 3,15e5

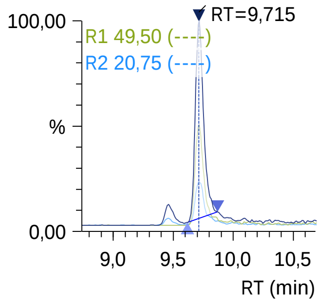

10H

Conc ----
Area ----
R#1 301,10>179,05 ---- (46,87)
R#2 301,10>106,85 ---- (22,82)
Q 301,10>151,00 (-) 2,78e3

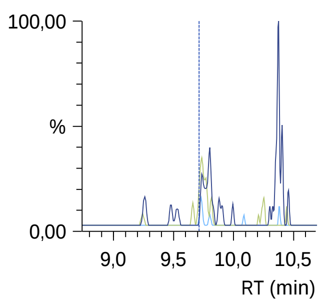

10M

Conc ----
Area ----
R#1 301,10>179,05 ---- (46,87)
R#2 301,10>106,85 ---- (22,82)
Q 301,10>151,00 (-) 1,69e3

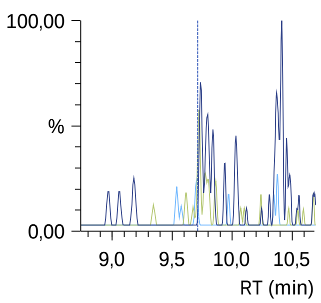

9H

Conc ----
Area ----
R#1 301,10>179,05 ---- (46,87)
R#2 301,10>106,85 ---- (22,82)
Q 301,10>151,00 (-) 2,26e3

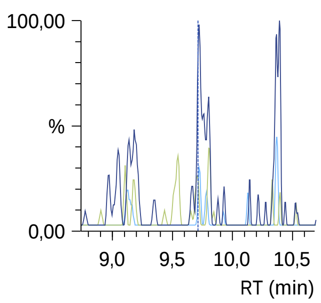

9M

Conc ----
Area ----
R#1 301,10>179,05 ---- (46,87)
R#2 301,10>106,85 ---- (22,82)
Q 301,10>151,00 (-) 2,31e3

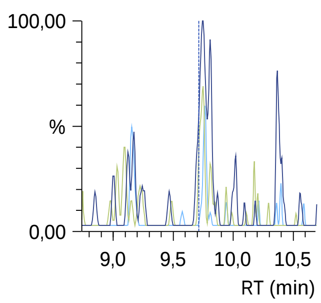

Compound: Hidroxibenzaldeido

Curve Fit: Linear | Weighting: None | Zero: Not Forced
Quantitative Method: External Standard
Q 121,40>91,90
R^2 = 0.9987906 R = 0.9993951
y = 2301,319x + 10896,57

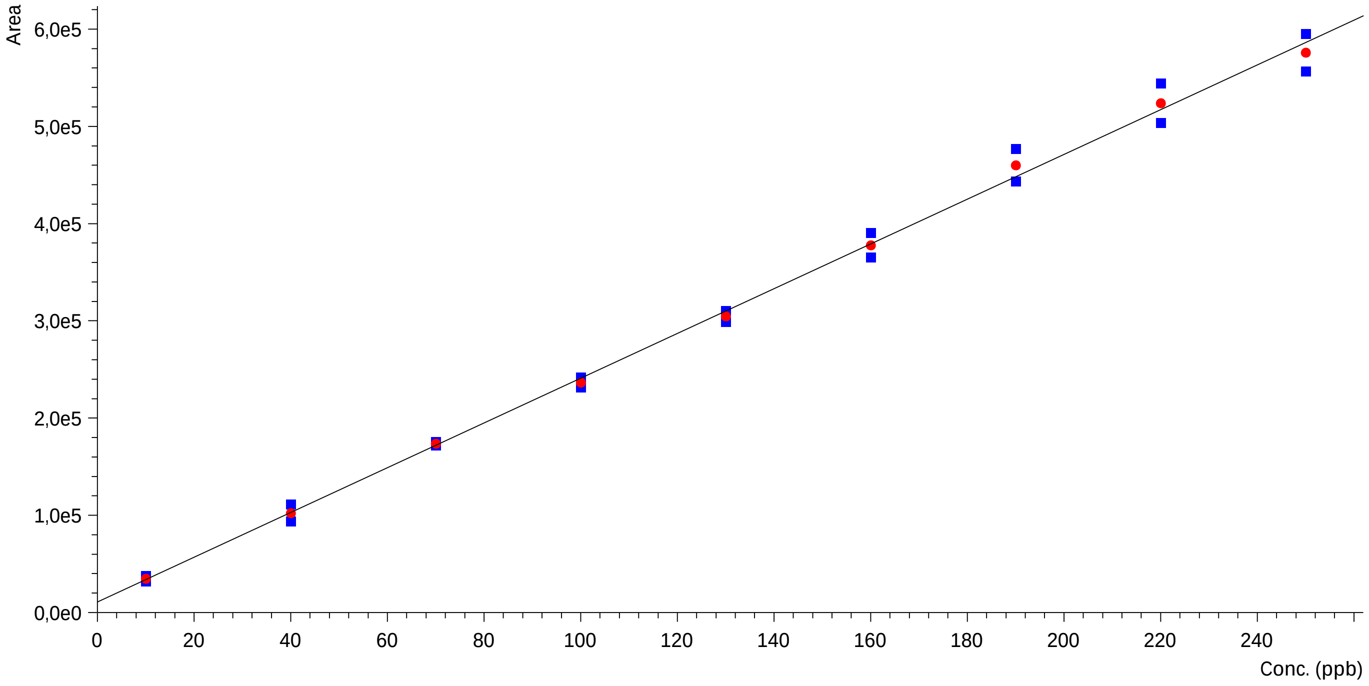

Sample ID	Area	Conc.	Sample Type	Level	Cal Point	Found RT	Ref 1 Actual Ratio
0	16292	2,3444	Ctrl.	0	----	8,683	0,00
10ppb001	32336	9,3162	Std.	1	Yes	8,551	4,71
10 ppb002	37449	11,5379	Std.	1	Yes	8,553	18,85
40 ppb001	111082	43,5340	Std.	2	Yes	8,552	7,04
40ppb002	93775	36,0133	Std.	2	Yes	8,556	12,51
70 ppb001	172109	70,0521	Std.	3	Yes	8,555	11,69
70ppb002	175732	71,6264	Std.	3	Yes	8,562	11,78
100 ppb001	231794	95,9875	Std.	4	Yes	8,555	13,20
100ppb002	241752	100,3142	Std.	4	Yes	8,563	11,11
130 ppb001	310491	130,1839	Std.	5	Yes	8,554	13,31
130ppb002	298824	125,1143	Std.	5	Yes	8,555	12,22
160 ppb001	365300	154,0001	Std.	6	Yes	8,562	12,29
160ppb002	390548	164,9713	Std.	6	Yes	8,555	10,10
190 ppb001	443342	187,9119	Std.	7	Yes	8,563	9,74
190ppb002	476958	202,5191	Std.	7	Yes	8,555	11,24
220 ppb001	503497	214,0514	Std.	8	Yes	8,556	13,53
220ppb002	544194	231,7357	Std.	8	Yes	8,563	11,41
250 ppb001	556542	237,1013	Std.	9	Yes	8,554	13,03
250 ppb002	595499	254,0293	Std.	9	Yes	8,555	12,77
10H	----	----	Unk.	----	----	----	----
10M	----	----	Unk.	----	----	----	----
9H	----	----	Unk.	----	----	----	----
9M	----	----	Unk.	----	----	----	----
Average	294606	123,2813				8,563	11,08
%RSD	65	67,3495				0,343	35,00
Maximum	595499	254,0293				8,683	18,85
Minimum	16292	2,3444				8,551	0,00
Std. Dev.	191077	83,0294				0,029	3,88

Compound: Hidroxibenzaldeido (continued)

0
Conc 2,3444
Area 16292
R#1 121,40>92,90 0,00 (11,24)
R#2 121,40>40,95 0,00 (2,26)
Q 121,40>91,90 (-) 2,80e3

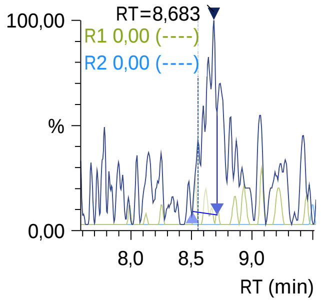

10ppb001
Conc 9,3162
Area 32336
R#1 121,40>92,90 4,71 (11,24)
R#2 121,40>40,95 0,00 (2,26)
Q 121,40>91,90 (-) 6,58e3

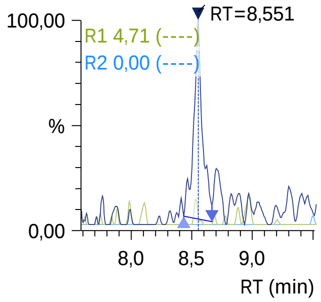

10 ppb002
Conc 11,5379
Area 37449
R#1 121,40>92,90 18,85 (11,24)
R#2 121,40>40,95 0,00 (2,26)
Q 121,40>91,90 (-) 5,96e3

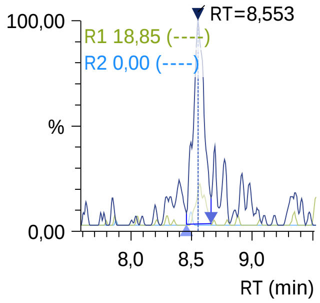

40 ppb001
Conc 43,5340
Area 111082
R#1 121,40>92,90 7,04 (11,24)
R#2 121,40>40,95 1,87 (2,26)
Q 121,40>91,90 (-) 2,58e4

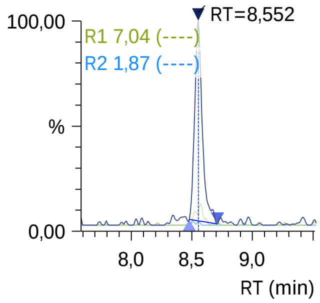

40ppb002
Conc 36,0133
Area 93775
R#1 121,40>92,90 12,51 (11,24)
R#2 121,40>40,95 0,90 (2,26)
Q 121,40>91,90 (-) 2,19e4

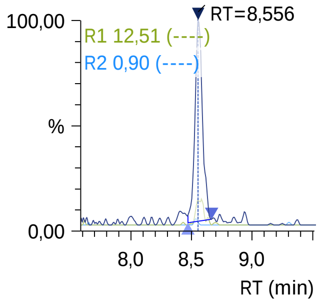

70 ppb001
Conc 70,0521
Area 172109
R#1 121,40>92,90 11,69 (11,24)
R#2 121,40>40,95 1,59 (2,26)
Q 121,40>91,90 (-) 3,81e4

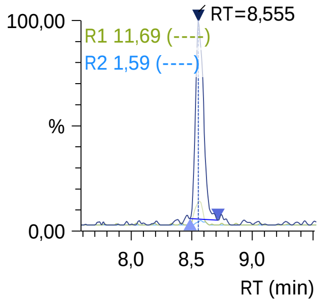

70ppb002
Conc 71,6264
Area 175732
R#1 121,40>92,90 11,78 (11,24)
R#2 121,40>40,95 0,89 (2,26)
Q 121,40>91,90 (-) 4,30e4

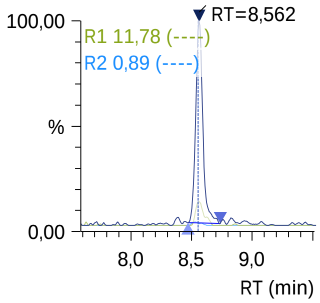

100 ppb001
Conc 95,9875
Area 231794
R#1 121,40>92,90 13,20 (11,24)
R#2 121,40>40,95 2,14 (2,26)
Q 121,40>91,90 (-) 5,32e4

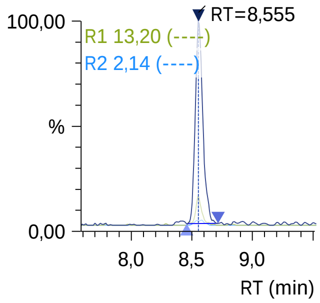

100ppb002
Conc 100,3142
Area 241752
R#1 121,40>92,90 11,11 (11,24)
R#2 121,40>40,95 2,06 (2,26)
Q 121,40>91,90 (-) 5,94e4

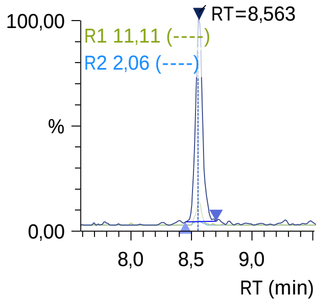

130 ppb001
Conc 130,1839
Area 310491
R#1 121,40>92,90 13,31 (11,24)
R#2 121,40>40,95 2,24 (2,26)
Q 121,40>91,90 (-) 7,32e4

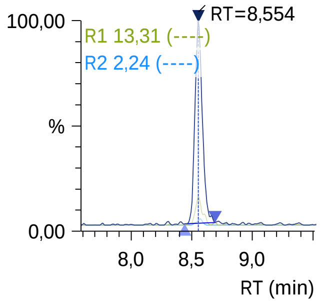

130ppb002
Conc 125,1143
Area 298824
R#1 121,40>92,90 12,22 (11,24)
R#2 121,40>40,95 2,78 (2,26)
Q 121,40>91,90 (-) 7,48e4

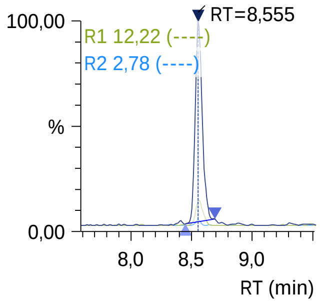

160 ppb001
Conc 154,0001
Area 365300
R#1 121,40>92,90 12,29 (11,24)
R#2 121,40>40,95 2,52 (2,26)
Q 121,40>91,90 (-) 8,24e4

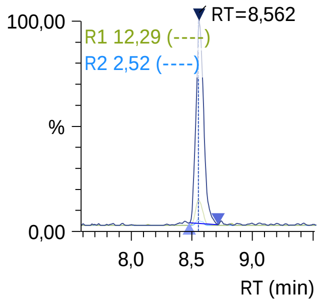

Compound: Hidroxibenzaldeido (continued)

160ppb002
Conc 164,9713
Area 390548
R#1 121,40>92,90 10,10 (11,24)
R#2 121,40>40,95 1,79 (2,26)
Q 121,40>91,90 (-) 9,63e4

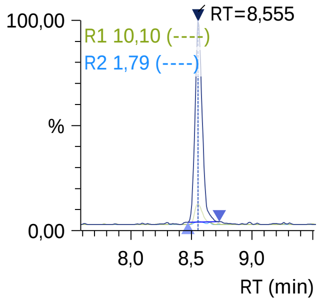

190 ppb001
Conc 187,9119
Area 443342
R#1 121,40>92,90 9,74 (11,24)
R#2 121,40>40,95 1,52 (2,26)
Q 121,40>91,90 (-) 1,03e5

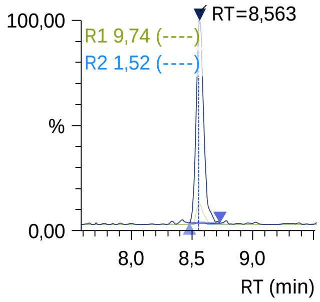

190ppb002
Conc 202,5191
Area 476958
R#1 121,40>92,90 11,24 (11,24)
R#2 121,40>40,95 2,26 (2,26)
Q 121,40>91,90 (-) 1,10e5

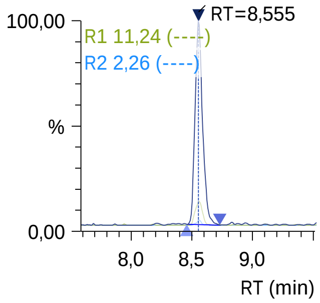

220 ppb001
Conc 214,0514
Area 503497
R#1 121,40>92,90 13,53 (11,24)
R#2 121,40>40,95 2,07 (2,26)
Q 121,40>91,90 (-) 1,19e5

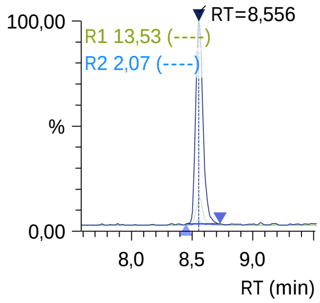

220ppb002
Conc 231,7357
Area 544194
R#1 121,40>92,90 11,41 (11,24)
R#2 121,40>40,95 2,14 (2,26)
Q 121,40>91,90 (-) 1,22e5

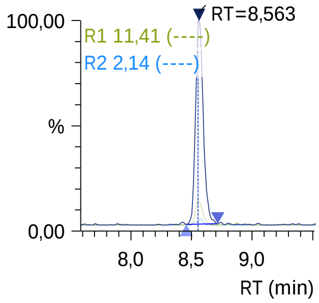

250 ppb001
Conc 237,1013
Area 556542
R#1 121,40>92,90 13,03 (11,24)
R#2 121,40>40,95 2,07 (2,26)
Q 121,40>91,90 (-) 1,33e5

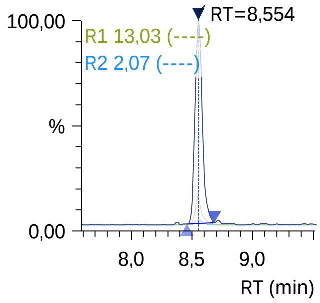

250 ppb002
Conc 254,0293
Area 595499
R#1 121,40>92,90 12,77 (11,24)
R#2 121,40>40,95 2,36 (2,26)
Q 121,40>91,90 (-) 1,43e5

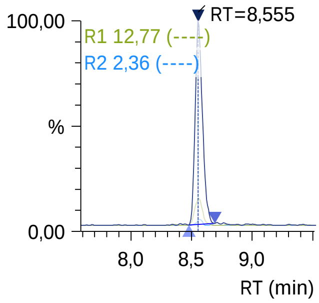

10H
Conc ----
Area ----
R#1 121,40>92,90 ---- (11,24)
R#2 121,40>40,95 ---- (2,26)
Q 121,40>91,90 (-) 3,72e3

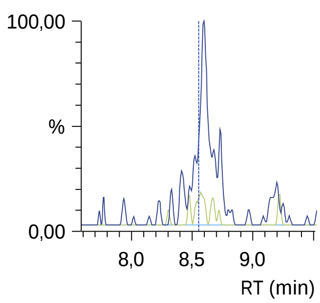

10M
Conc ----
Area ----
R#1 121,40>92,90 ---- (11,24)
R#2 121,40>40,95 ---- (2,26)
Q 121,40>91,90 (-) 4,20e3

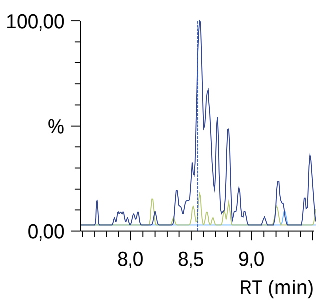

9H
Conc ----
Area ----
R#1 121,40>92,90 ---- (11,24)
R#2 121,40>40,95 ---- (2,26)
Q 121,40>91,90 (-) 2,18e3

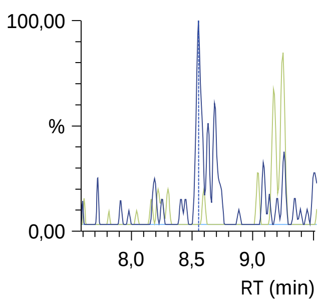

9M
Conc ----
Area ----
R#1 121,40>92,90 ---- (11,24)
R#2 121,40>40,95 ---- (2,26)
Q 121,40>91,90 (-) 1,95e3

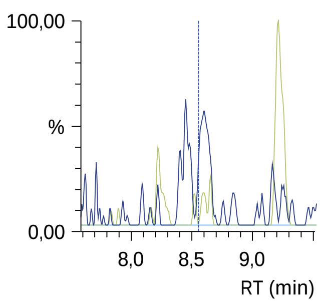

Compound: Naringenina

Curve Fit: Linear | Weighting: None | Zero: Not Forced
Quantitative Method: External Standard
Q 271,10>151,00
R^2 = 0.9933495 R = 0.9966692
y = 6281,776x + 59537,76

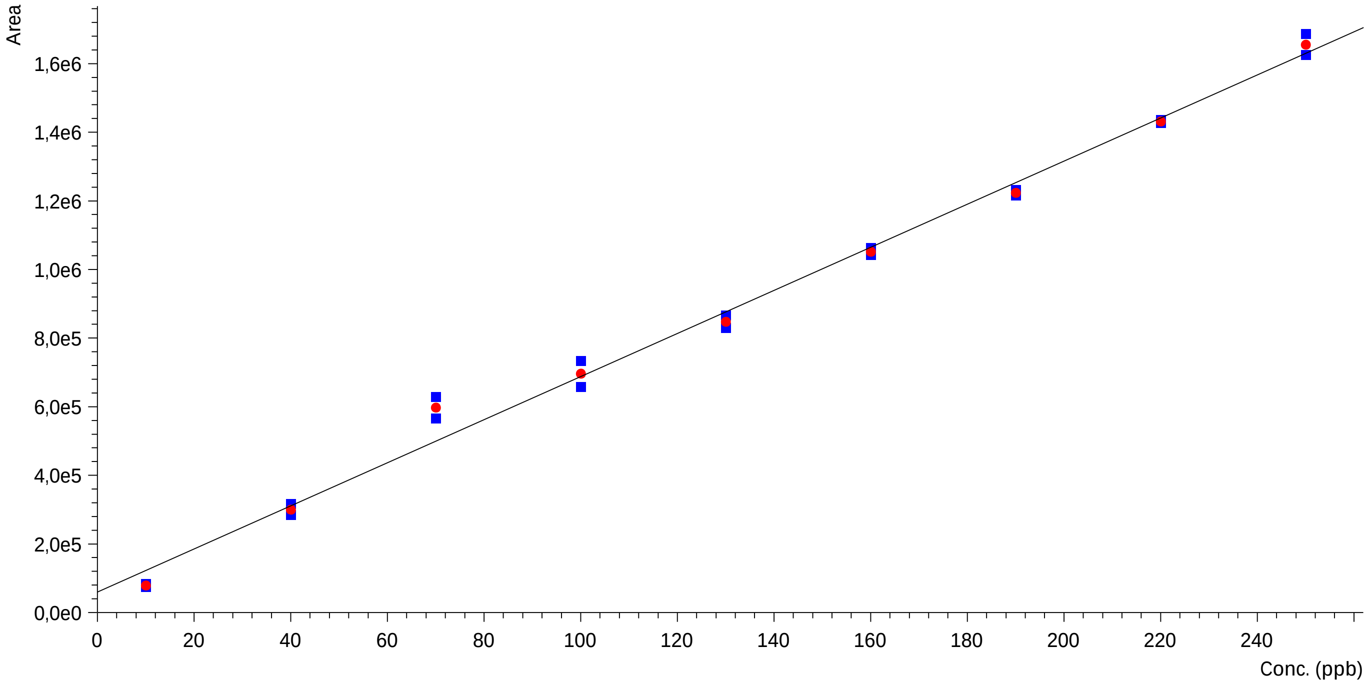

Sample ID	Area	Conc.	Sample Type	Level	Cal Point	Found RT	Ref 1 Actual Ratio
0	----	----	Ctrl.	0	----	----	----
10ppb001	75154	2,4860	Std.	1	Yes	9,756	48,56
10 ppb002	83344	3,7897	Std.	1	Yes	9,762	75,38
40 ppb001	284968	35,8865	Std.	2	Yes	9,751	81,94
40ppb002	316195	40,8574	Std.	2	Yes	9,757	79,21
70 ppb001	628798	90,6210	Std.	3	Yes	9,762	79,38
70ppb002	566302	80,6721	Std.	3	Yes	9,761	75,23
100 ppb001	734413	107,4339	Std.	4	Yes	9,754	71,68
100ppb002	658511	95,3510	Std.	4	Yes	9,755	73,44
130 ppb001	829812	122,6205	Std.	5	Yes	9,752	81,24
130ppb002	866623	128,4805	Std.	5	Yes	9,755	74,80
160 ppb001	1041973	156,3945	Std.	6	Yes	9,760	70,89
160ppb002	1063148	159,7653	Std.	6	Yes	9,752	75,96
190 ppb001	1232201	186,6770	Std.	7	Yes	9,759	80,18
190ppb002	1214996	183,9381	Std.	7	Yes	9,752	80,76
220 ppb001	1427269	217,7301	Std.	8	Yes	9,750	74,99
220ppb002	1435627	219,0605	Std.	8	Yes	9,757	76,90
250 ppb001	1687068	259,0876	Std.	9	Yes	9,746	71,43
250 ppb002	1624632	249,1483	Std.	9	Yes	9,752	78,38
10H	8224	-8,1687	Unk.	----	----	9,787	8,54
10M	10465	-7,8119	Unk.	----	----	9,802	16,03
9H	6246	-8,4836	Unk.	----	----	9,769	54,05
9M	11759	-7,6059	Unk.	----	----	9,769	63,74
Average	718533	104,9059				9,760	67,85
%RSD	79	86,0877				0,130	29,26
Maximum	1687068	259,0876				9,802	81,94
Minimum	6246	-8,4836				9,746	8,54
Std. Dev.	567314	90,3110				0,013	19,85

Compound: Naringenina (continued)

0
Conc ----
Area ----
R#1 271,10>119,00 ---- (80,76)
R#2 271,10>106,90 ---- (27,97)
Q 271,10>151,00 (-) 1,72e3

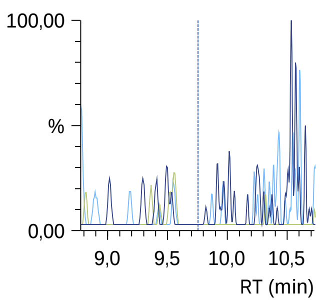

10ppb001
Conc 2,4860
Area 75154
R#1 271,10>119,00 48,56 (80,76)
R#2 271,10>106,90 25,56 (27,97)
Q 271,10>151,00 (-) 1,78e4

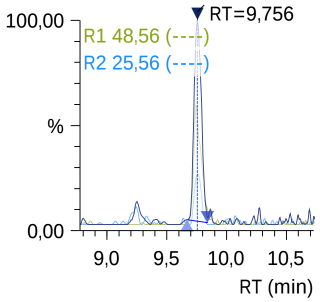

10 ppb002
Conc 3,7897
Area 83344
R#1 271,10>119,00 75,38 (80,76)
R#2 271,10>106,90 24,91 (27,97)
Q 271,10>151,00 (-) 1,72e4

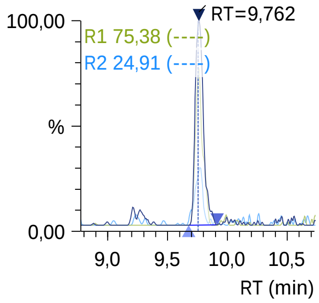

40 ppb001
Conc 35,8865
Area 284968
R#1 271,10>119,00 81,94 (80,76)
R#2 271,10>106,90 23,95 (27,97)
Q 271,10>151,00 (-) 6,39e4

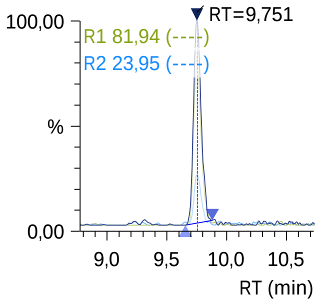

40ppb002
Conc 40,8574
Area 316195
R#1 271,10>119,00 79,21 (80,76)
R#2 271,10>106,90 27,21 (27,97)
Q 271,10>151,00 (-) 7,48e4

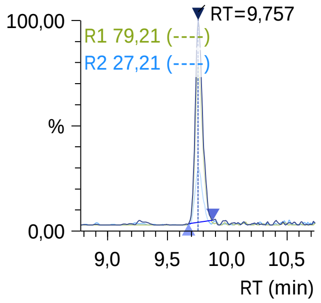

70 ppb001
Conc 90,6210
Area 628798
R#1 271,10>119,00 79,38 (80,76)
R#2 271,10>106,90 31,71 (27,97)
Q 271,10>151,00 (-) 1,41e5

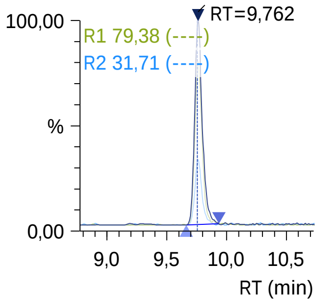

70ppb002
Conc 80,6721
Area 566302
R#1 271,10>119,00 75,23 (80,76)
R#2 271,10>106,90 29,10 (27,97)
Q 271,10>151,00 (-) 1,29e5

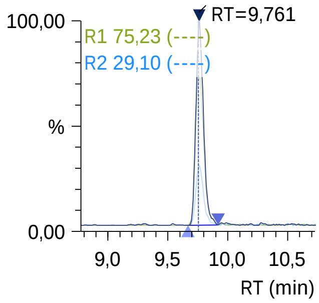

100 ppb001
Conc 107,4339
Area 734413
R#1 271,10>119,00 71,68 (80,76)
R#2 271,10>106,90 27,57 (27,97)
Q 271,10>151,00 (-) 1,72e5

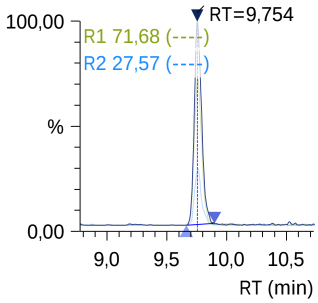

100ppb002
Conc 95,3510
Area 658511
R#1 271,10>119,00 73,44 (80,76)
R#2 271,10>106,90 26,11 (27,97)
Q 271,10>151,00 (-) 1,60e5

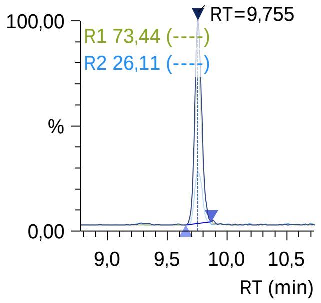

130 ppb001
Conc 122,6205
Area 829812
R#1 271,10>119,00 81,24 (80,76)
R#2 271,10>106,90 29,06 (27,97)
Q 271,10>151,00 (-) 1,95e5

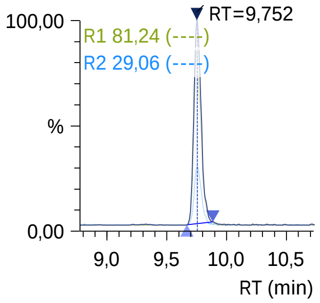

130ppb002
Conc 128,4805
Area 866623
R#1 271,10>119,00 74,80 (80,76)
R#2 271,10>106,90 26,77 (27,97)
Q 271,10>151,00 (-) 1,95e5

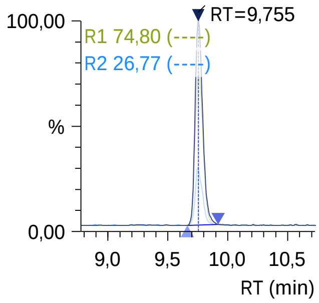

160 ppb001
Conc 156,3945
Area 1041973
R#1 271,10>119,00 70,89 (80,76)
R#2 271,10>106,90 26,44 (27,97)
Q 271,10>151,00 (-) 2,47e5

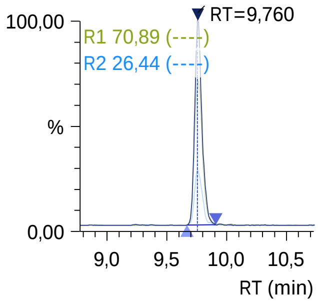

Compound: Naringenina (continued)

160ppb002
Conc 159,7653
Area 1063148
R#1 271,10>119,00 75,96 (80,76)
R#2 271,10>106,90 23,78 (27,97)
Q 271,10>151,00 (-) 2,57e5

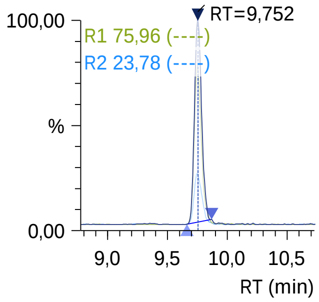

190 ppb001
Conc 186,6770
Area 1232201
R#1 271,10>119,00 80,18 (80,76)
R#2 271,10>106,90 26,92 (27,97)
Q 271,10>151,00 (-) 2,80e5

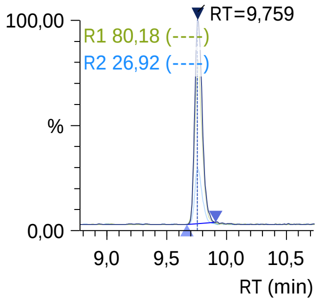

190ppb002
Conc 183,9381
Area 1214996
R#1 271,10>119,00 80,76 (80,76)
R#2 271,10>106,90 27,97 (27,97)
Q 271,10>151,00 (-) 2,72e5

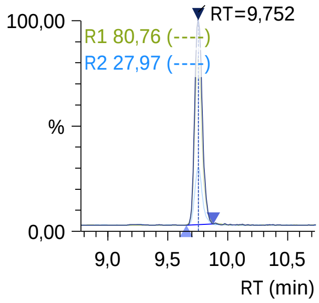

220 ppb001
Conc 217,7301
Area 1427269
R#1 271,10>119,00 74,99 (80,76)
R#2 271,10>106,90 25,68 (27,97)
Q 271,10>151,00 (-) 3,42e5

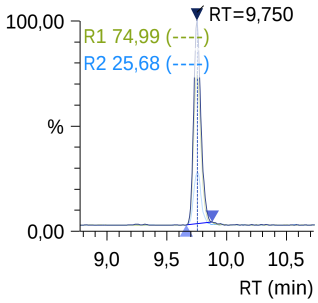

220ppb002
Conc 219,0605
Area 1435627
R#1 271,10>119,00 76,90 (80,76)
R#2 271,10>106,90 27,41 (27,97)
Q 271,10>151,00 (-) 3,28e5

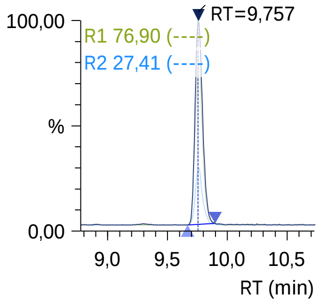

250 ppb001
Conc 259,0876
Area 1687068
R#1 271,10>119,00 71,43 (80,76)
R#2 271,10>106,90 26,76 (27,97)
Q 271,10>151,00 (-) 3,82e5

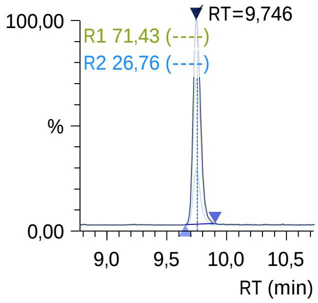

250 ppb002
Conc 249,1483
Area 1624632
R#1 271,10>119,00 78,38 (80,76)
R#2 271,10>106,90 28,31 (27,97)
Q 271,10>151,00 (-) 3,66e5

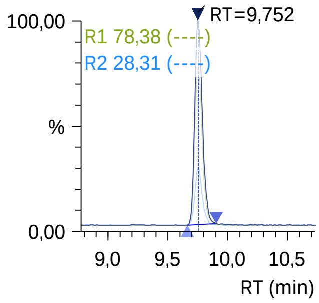

10H
Conc -8,1687
Area 8224
R#1 271,10>119,00 8,54 (80,76)
R#2 271,10>106,90 18,61 (27,97)
Q 271,10>151,00 (-) 1,97e3

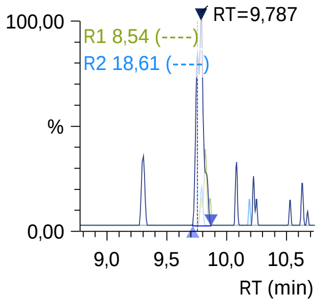

10M
Conc -7,8119
Area 10465
R#1 271,10>119,00 16,03 (80,76)
R#2 271,10>106,90 27,18 (27,97)
Q 271,10>151,00 (-) 2,35e3

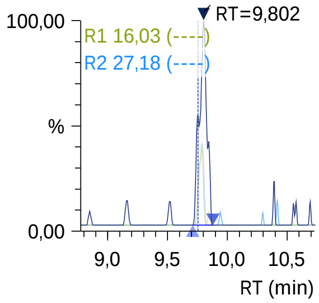

9H
Conc -8,4836
Area 6246
R#1 271,10>119,00 54,05 (80,76)
R#2 271,10>106,90 0,00 (27,97)
Q 271,10>151,00 (-) 1,56e3

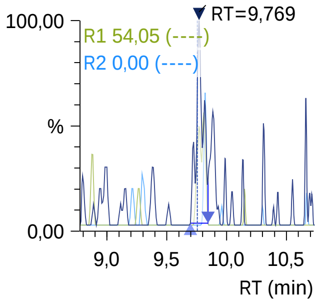

9M
Conc -7,6059
Area 11759
R#1 271,10>119,00 63,74 (80,76)
R#2 271,10>106,90 30,97 (27,97)
Q 271,10>151,00 (-) 2,24e3

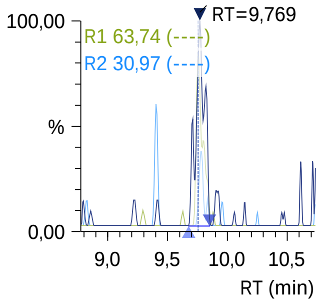

Compound: Ac. Clorogenico

Curve Fit: Linear | Weighting: None | Zero: Not Forced
Quantitative Method: External Standard
Q 353,10>191,10
R^2 = 0.9982929 R = 0.9991461
y = 6247,516x - 41458,85

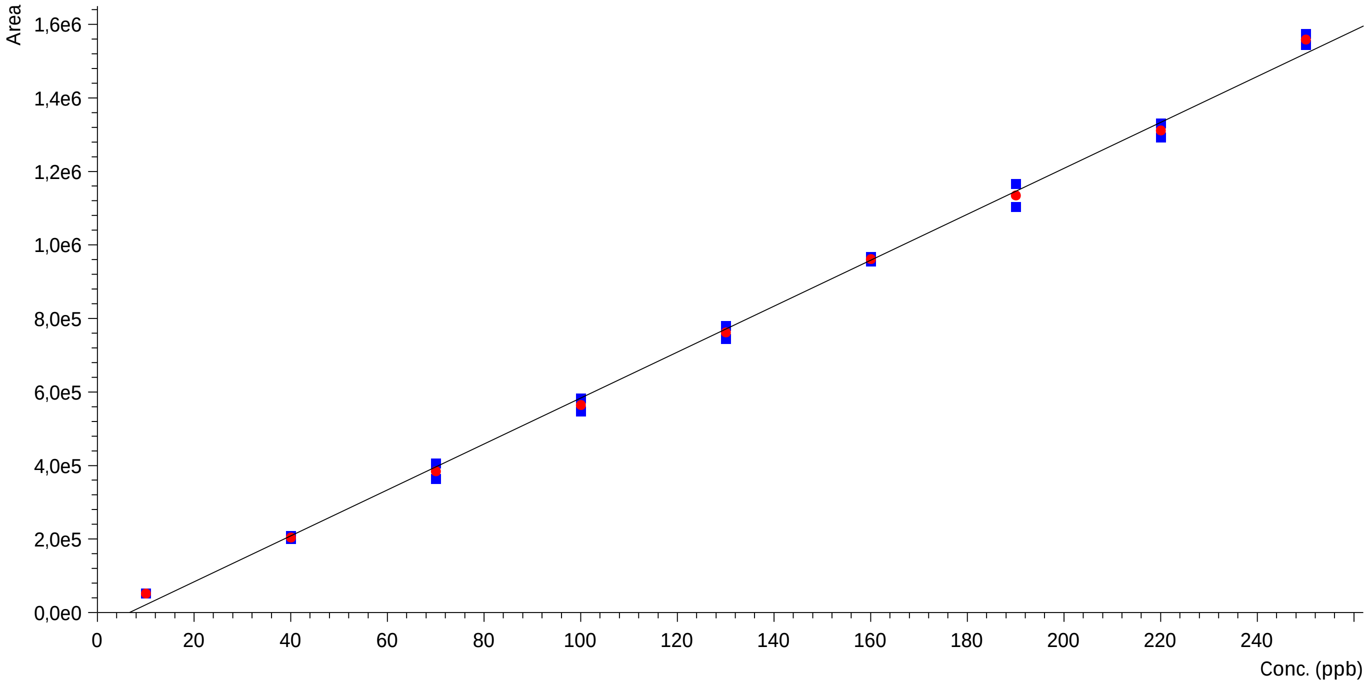

Sample ID	Area	Conc.	Sample Type	Level	Cal Point	Found RT	Ref 1 Actual Ratio
0	----	----	Ctrl.	0	----	----	----
10ppb001	51925	14,9474	Std.	1	Yes	7,923	11,61
10 ppb002	52380	15,0203	Std.	1	Yes	7,912	7,96
40 ppb001	201217	38,8435	Std.	2	Yes	7,921	8,93
40ppb002	208685	40,0389	Std.	2	Yes	7,927	8,07
70 ppb001	405844	71,5968	Std.	3	Yes	7,931	5,61
70ppb002	363962	64,8932	Std.	3	Yes	7,923	6,61
100 ppb001	546396	94,0942	Std.	4	Yes	7,927	8,41
100ppb002	582773	99,9168	Std.	4	Yes	7,928	7,66
130 ppb001	744221	125,7587	Std.	5	Yes	7,919	7,74
130ppb002	780102	131,5020	Std.	5	Yes	7,928	6,35
160 ppb001	955651	159,6010	Std.	6	Yes	7,930	8,57
160ppb002	968287	161,6236	Std.	6	Yes	7,920	7,28
190 ppb001	1102929	183,1749	Std.	7	Yes	7,932	7,55
190ppb002	1166937	193,4201	Std.	7	Yes	7,921	6,01
220 ppb001	1330388	219,5827	Std.	8	Yes	7,927	6,86
220ppb002	1292996	213,5976	Std.	8	Yes	7,928	7,52
250 ppb001	1544651	253,8785	Std.	9	Yes	7,917	8,22
250 ppb002	1573585	258,5098	Std.	9	Yes	7,925	5,88
10H	154488	31,3639	Unk.	----	----	7,967	7,02
10M	290387	53,1165	Unk.	----	----	7,936	6,24
9H	72276	18,2048	Unk.	----	----	7,921	8,65
9M	188215	36,7625	Unk.	----	----	7,939	11,83
Average	662650	112,7022				7,927	7,75
%RSD	77	72,0772				0,137	20,60
Maximum	1573585	258,5098				7,967	11,83
Minimum	51925	14,9474				7,912	5,61
Std. Dev.	507502	81,2326				0,011	1,60

Compound: Ac. Clorogenico (continued)

0
Conc ----
Area ----
R#1 353,10>84,85 ---- (7,79)
R#2 353,10>183,10 ---- (2,72)
Q 353,10>191,10 (-) 1,98e3

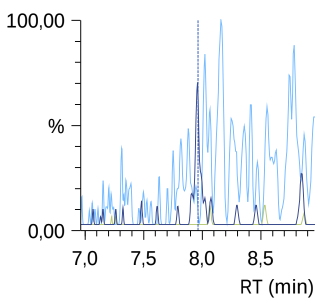

10ppb001
Conc 14,9474
Area 51925
R#1 353,10>84,85 11,61 (7,79)
R#2 353,10>183,10 0,00 (2,72)
Q 353,10>191,10 (-) 1,08e4

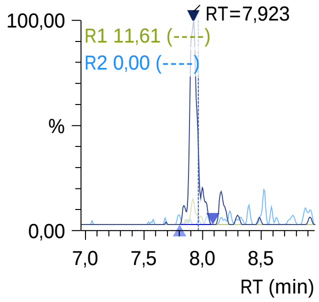

10 ppb002
Conc 15,0203
Area 52380
R#1 353,10>84,85 7,96 (7,79)
R#2 353,10>183,10 1,60 (2,72)
Q 353,10>191,10 (-) 1,07e4

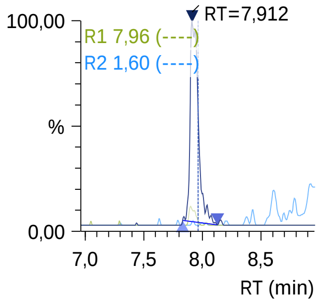

40 ppb001
Conc 38,8435
Area 201217
R#1 353,10>84,85 8,93 (7,79)
R#2 353,10>183,10 0,00 (2,72)
Q 353,10>191,10 (-) 4,99e4

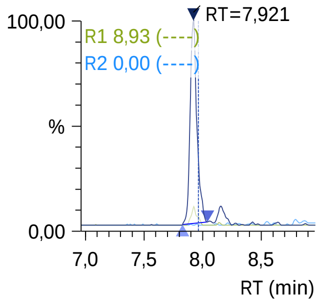

40ppb002
Conc 40,0389
Area 208685
R#1 353,10>84,85 8,07 (7,79)
R#2 353,10>183,10 0,00 (2,72)
Q 353,10>191,10 (-) 4,77e4

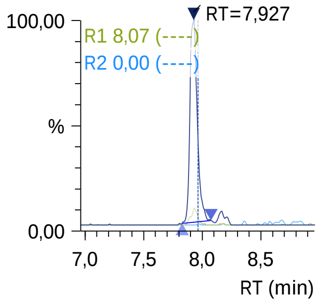

70 ppb001
Conc 71,5968
Area 405844
R#1 353,10>84,85 5,61 (7,79)
R#2 353,10>183,10 0,08 (2,72)
Q 353,10>191,10 (-) 9,29e4

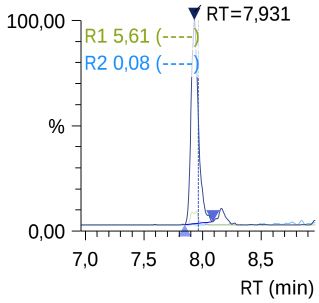

70ppb002
Conc 64,8932
Area 363962
R#1 353,10>84,85 6,61 (7,79)
R#2 353,10>183,10 0,00 (2,72)
Q 353,10>191,10 (-) 7,89e4

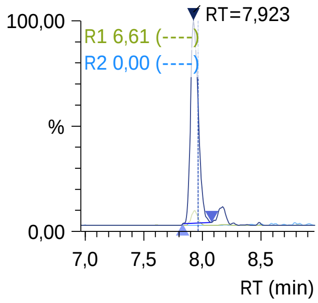

100 ppb001
Conc 94,0942
Area 546396
R#1 353,10>84,85 8,41 (7,79)
R#2 353,10>183,10 0,00 (2,72)
Q 353,10>191,10 (-) 1,27e5

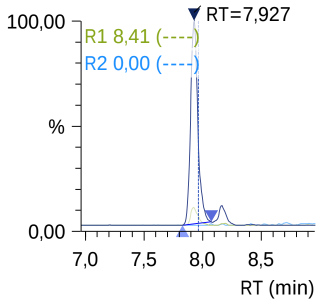

100ppb002
Conc 99,9168
Area 582773
R#1 353,10>84,85 7,66 (7,79)
R#2 353,10>183,10 0,00 (2,72)
Q 353,10>191,10 (-) 1,31e5

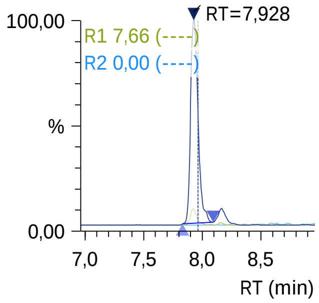

130 ppb001
Conc 125,7587
Area 744221
R#1 353,10>84,85 7,74 (7,79)
R#2 353,10>183,10 0,00 (2,72)
Q 353,10>191,10 (-) 1,69e5

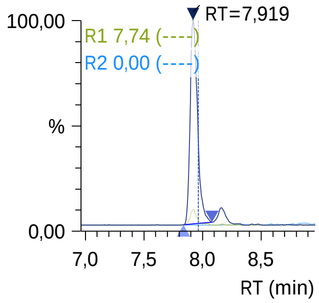

130ppb002
Conc 131,5020
Area 780102
R#1 353,10>84,85 6,35 (7,79)
R#2 353,10>183,10 0,00 (2,72)
Q 353,10>191,10 (-) 1,82e5

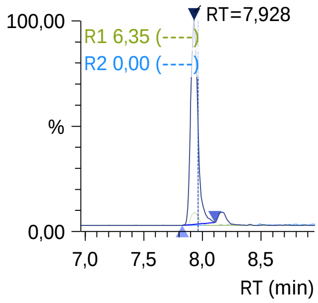

160 ppb001
Conc 159,6010
Area 955651
R#1 353,10>84,85 8,57 (7,79)
R#2 353,10>183,10 0,03 (2,72)
Q 353,10>191,10 (-) 2,23e5

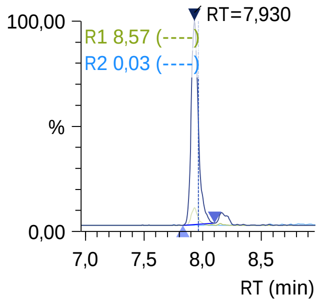

Compound: Ac. Clorogenico (continued)

160ppb002
Conc 161,6236
Area 968287
R#1 353,10>84,85 7,28 (7,79)
R#2 353,10>183,10 0,00 (2,72)
Q 353,10>191,10 (-) 2,24e5

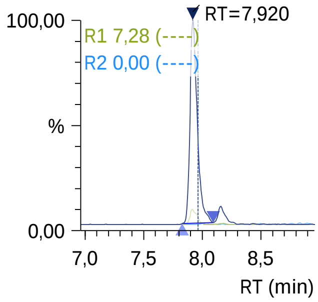

190 ppb001
Conc 183,1749
Area 1102929
R#1 353,10>84,85 7,55 (7,79)
R#2 353,10>183,10 0,00 (2,72)
Q 353,10>191,10 (-) 2,68e5

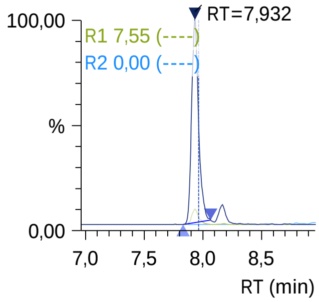

190ppb002
Conc 193,4201
Area 1166937
R#1 353,10>84,85 6,01 (7,79)
R#2 353,10>183,10 0,00 (2,72)
Q 353,10>191,10 (-) 2,72e5

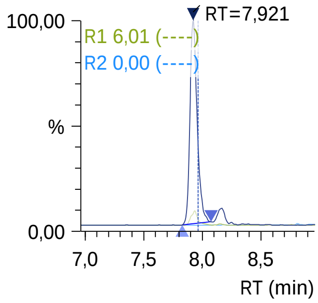

220 ppb001
Conc 219,5827
Area 1330388
R#1 353,10>84,85 6,86 (7,79)
R#2 353,10>183,10 0,00 (2,72)
Q 353,10>191,10 (-) 3,05e5

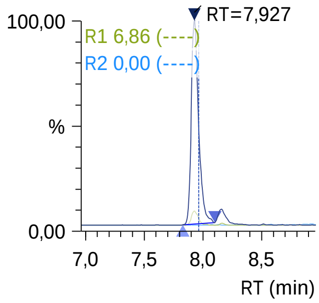

220ppb002
Conc 213,5976
Area 1292996
R#1 353,10>84,85 7,52 (7,79)
R#2 353,10>183,10 0,00 (2,72)
Q 353,10>191,10 (-) 3,00e5

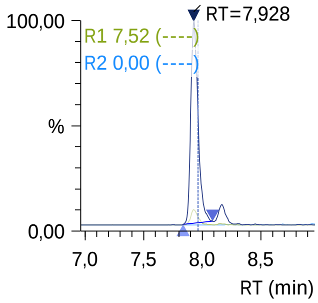

250 ppb001
Conc 253,8785
Area 1544651
R#1 353,10>84,85 8,22 (7,79)
R#2 353,10>183,10 0,00 (2,72)
Q 353,10>191,10 (-) 3,56e5

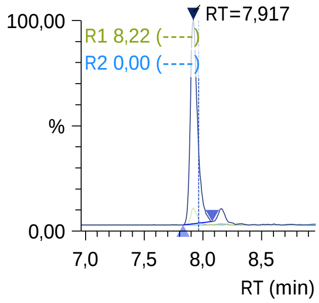

250 ppb002
Conc 258,5098
Area 1573585
R#1 353,10>84,85 5,88 (7,79)
R#2 353,10>183,10 0,00 (2,72)
Q 353,10>191,10 (-) 3,66e5

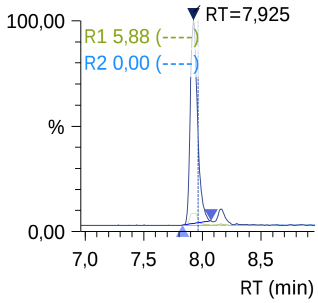

10H
Conc 31,3639
Area 154488
R#1 353,10>84,85 7,02 (7,79)
R#2 353,10>183,10 0,00 (2,72)
Q 353,10>191,10 (-) 1,94e4

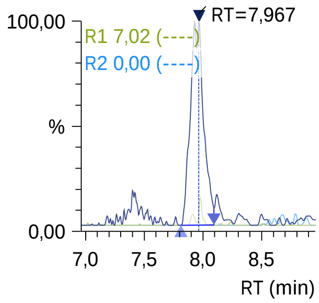

10M
Conc 53,1165
Area 290387
R#1 353,10>84,85 6,24 (7,79)
R#2 353,10>183,10 0,00 (2,72)
Q 353,10>191,10 (-) 6,34e4

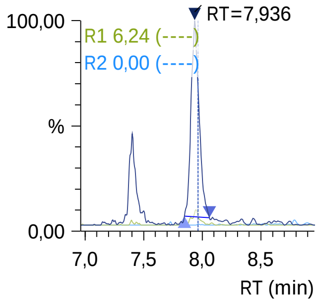

9H
Conc 18,2048
Area 72276
R#1 353,10>84,85 8,65 (7,79)
R#2 353,10>183,10 0,00 (2,72)
Q 353,10>191,10 (-) 1,01e4

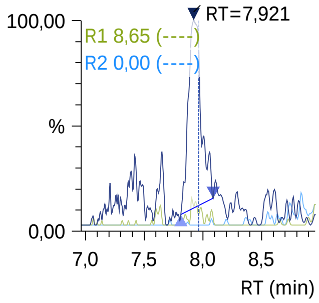

9M
Conc 36,7625
Area 188215
R#1 353,10>84,85 11,83 (7,79)
R#2 353,10>183,10 0,00 (2,72)
Q 353,10>191,10 (-) 3,25e4

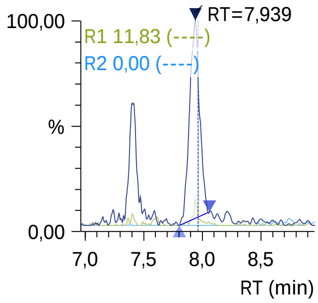

Compound: Ac. Siringico

Curve Fit: Linear | Weighting: None | Zero: Not Forced
Quantitative Method: External Standard
Q 197,20>182,05
$R^2 = 0.9929331$ R = 0.9964603
y = 439,2870x - 3476,172

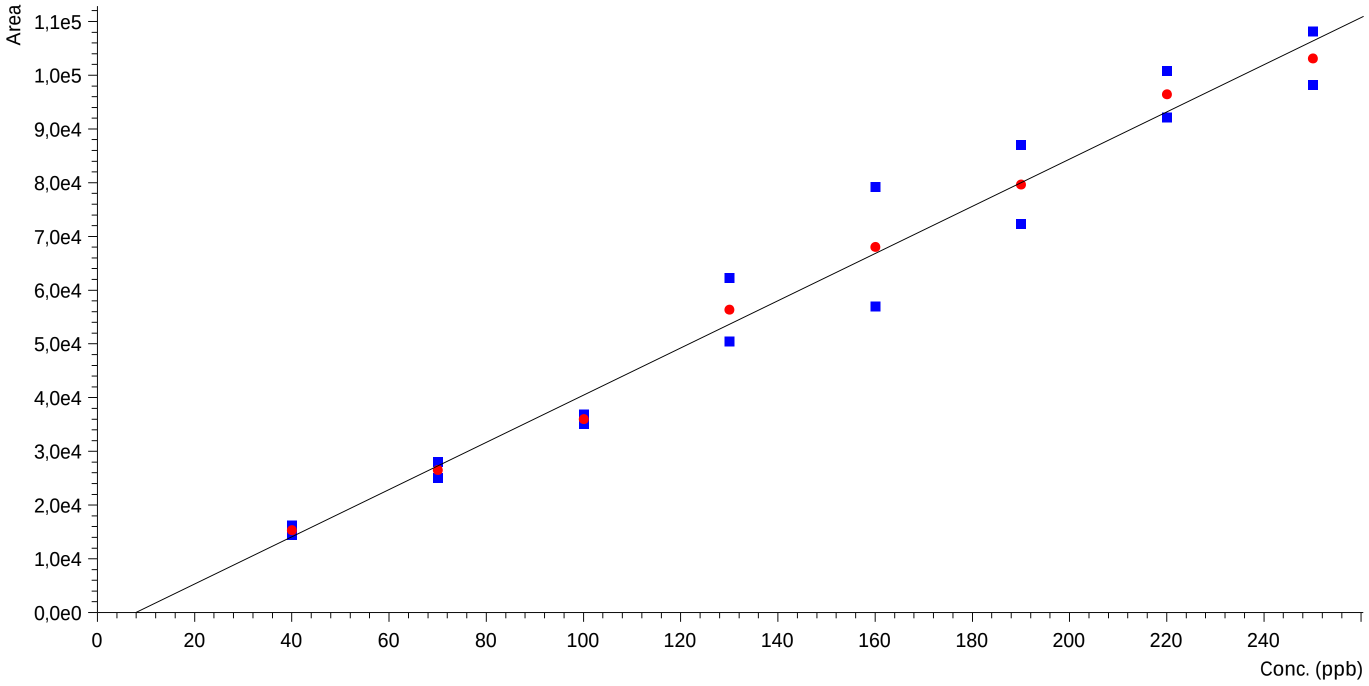

Sample ID	Area	Conc.	Sample Type	Level	Cal Point	Found RT	Ref 1 Actual Ratio
0	----	----	Ctrl.	0	----	----	----
10ppb001	----	----	Std.	1	No	----	----
10 ppb002	----	----	Std.	1	No	----	----
40 ppb001	16241	44,8838	Std.	2	Yes	8,462	87,50
40ppb002	14461	40,8328	Std.	2	Yes	8,467	100,43
70 ppb001	28035	71,7335	Std.	3	Yes	8,467	81,06
70ppb002	25073	64,9900	Std.	3	Yes	8,467	102,97
100 ppb001	35158	87,9467	Std.	4	Yes	8,461	99,51
100ppb002	36938	91,9987	Std.	4	Yes	8,465	77,82
130 ppb001	62307	149,7507	Std.	5	Yes	8,475	64,23
130ppb002	50477	122,8209	Std.	5	Yes	8,466	66,05
160 ppb001	79207	188,2210	Std.	6	Yes	8,475	69,23
160ppb002	56970	137,6012	Std.	6	Yes	8,458	110,24
190 ppb001	72306	172,5107	Std.	7	Yes	8,477	67,50
190ppb002	87022	206,0124	Std.	7	Yes	8,466	84,64
220 ppb001	92153	217,6912	Std.	8	Yes	8,467	87,02
220ppb002	100822	237,4250	Std.	8	Yes	8,470	74,81
250 ppb001	98181	231,4151	Std.	9	Yes	8,459	101,28
250 ppb002	108176	254,1663	Std.	9	Yes	8,460	93,54
10H	11344	33,7366	Unk.	----	----	8,504	44,86
10M	14458	40,8257	Unk.	----	----	8,467	72,80
9H	158485	368,6900	Unk.	----	----	8,483	57,83
9M	151524	352,8456	Unk.	----	----	8,487	80,25
Average	64967	155,8049				8,470	81,18
%RSD	68	64,6825				0,130	20,96
Maximum	158485	368,6900				8,504	110,24
Minimum	11344	33,7366				8,458	44,86
Std. Dev.	44271	100,7785				0,011	17,02

Compound: Ac. Siringico (continued)

0
Conc ----
Area ----
R#1 197,20>122,95 ---- (87,02)
R#2 197,20>94,85 ---- (35,36)
Q 197,20>182,05 (-) 8,10e2

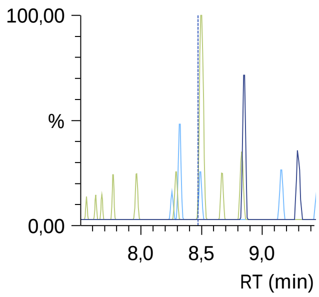

10ppb001
Conc ----
Area ----
R#1 197,20>122,95 ---- (87,02)
R#2 197,20>94,85 ---- (35,36)
Q 197,20>182,05 (-) 2,13e3

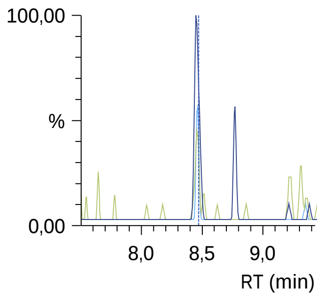

10 ppb002
Conc ----
Area ----
R#1 197,20>122,95 ---- (87,02)
R#2 197,20>94,85 ---- (35,36)
Q 197,20>182,05 (-) 8,18e2

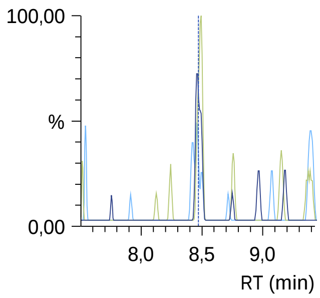

40 ppb001
Conc 44,8838
Area 16241
R#1 197,20>122,95 87,50 (87,02)
R#2 197,20>94,85 27,97 (35,36)
Q 197,20>182,05 (-) 4,45e3

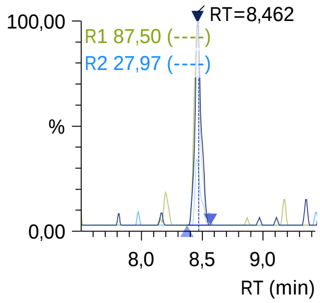

40ppb002
Conc 40,8328
Area 14461
R#1 197,20>122,95 100,43 (87,02)
R#2 197,20>94,85 65,42 (35,36)
Q 197,20>182,05 (-) 3,67e3

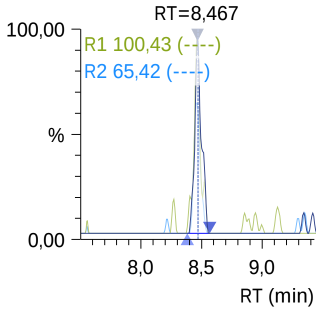

70 ppb001
Conc 71,7335
Area 28035
R#1 197,20>122,95 81,06 (87,02)
R#2 197,20>94,85 39,28 (35,36)
Q 197,20>182,05 (-) 7,72e3

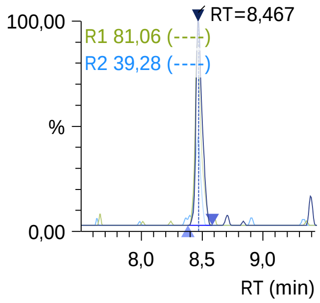

70ppb002
Conc 64,9900
Area 25073
R#1 197,20>122,95 102,97 (87,02)
R#2 197,20>94,85 54,78 (35,36)
Q 197,20>182,05 (-) 6,90e3

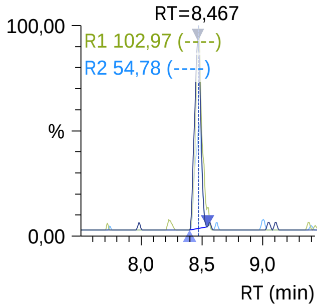

100 ppb001
Conc 87,9467
Area 35158
R#1 197,20>122,95 99,51 (87,02)
R#2 197,20>94,85 44,54 (35,36)
Q 197,20>182,05 (-) 9,32e3

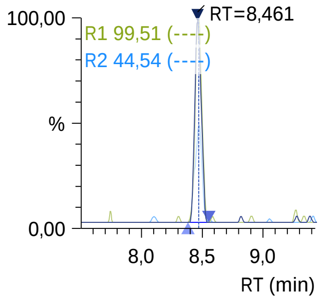

100ppb002
Conc 91,9987
Area 36938
R#1 197,20>122,95 77,82 (87,02)
R#2 197,20>94,85 60,00 (35,36)
Q 197,20>182,05 (-) 9,01e3

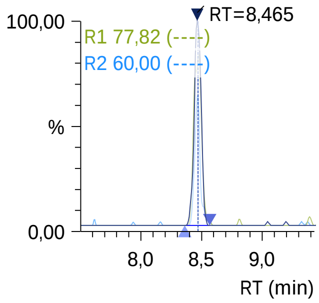

130 ppb001
Conc 149,7507
Area 62307
R#1 197,20>122,95 64,23 (87,02)
R#2 197,20>94,85 20,60 (35,36)
Q 197,20>182,05 (-) 1,67e4

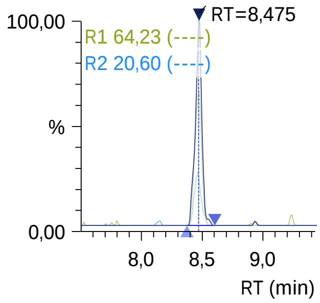

130ppb002
Conc 122,8209
Area 50477
R#1 197,20>122,95 66,05 (87,02)
R#2 197,20>94,85 37,30 (35,36)
Q 197,20>182,05 (-) 1,48e4

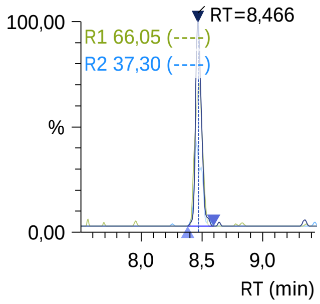

160 ppb001
Conc 188,2210
Area 79207
R#1 197,20>122,95 69,23 (87,02)
R#2 197,20>94,85 47,14 (35,36)
Q 197,20>182,05 (-) 1,99e4

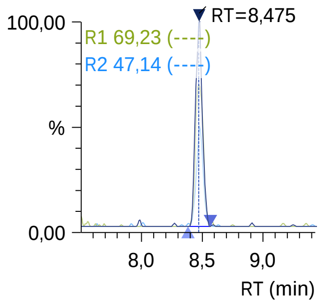

Compound: Ac. Siringico (continued)

160ppb002
Conc 137,6012
Area 56970
R#1 197,20>122,95 110,24 (87,02)
R#2 197,20>94,85 58,29 (35,36)
Q 197,20>182,05 (-) 1,34e4

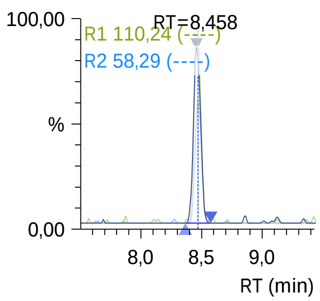

190 ppb001
Conc 172,5107
Area 72306
R#1 197,20>122,95 67,50 (87,02)
R#2 197,20>94,85 63,07 (35,36)
Q 197,20>182,05 (-) 1,88e4

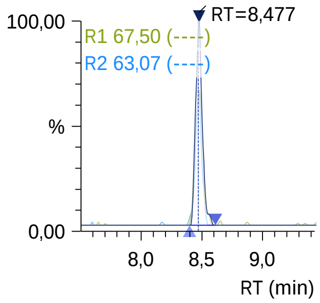

190ppb002
Conc 206,0124
Area 87022
R#1 197,20>122,95 84,64 (87,02)
R#2 197,20>94,85 55,22 (35,36)
Q 197,20>182,05 (-) 2,11e4

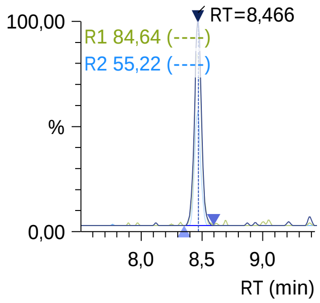

220 ppb001
Conc 217,6912
Area 92153
R#1 197,20>122,95 87,02 (87,02)
R#2 197,20>94,85 35,36 (35,36)
Q 197,20>182,05 (-) 2,50e4

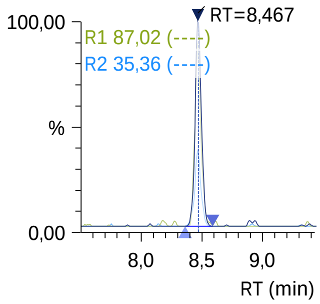

220ppb002
Conc 237,4250
Area 100822
R#1 197,20>122,95 74,81 (87,02)
R#2 197,20>94,85 33,09 (35,36)
Q 197,20>182,05 (-) 2,68e4

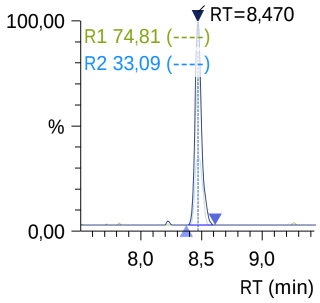

250 ppb001
Conc 231,4151
Area 98181
R#1 197,20>122,95 101,28 (87,02)
R#2 197,20>94,85 51,71 (35,36)
Q 197,20>182,05 (-) 2,48e4

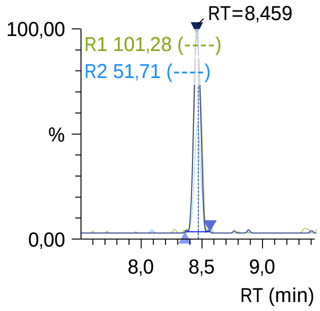

250 ppb002
Conc 254,1663
Area 108176
R#1 197,20>122,95 93,54 (87,02)
R#2 197,20>94,85 56,87 (35,36)
Q 197,20>182,05 (-) 2,46e4

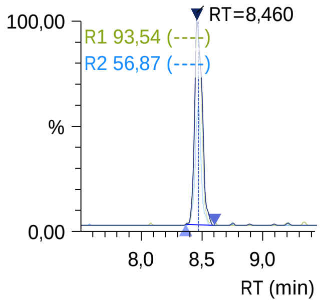

10H
Conc 33,7366
Area 11344
R#1 197,20>122,95 44,86 (87,02)
R#2 197,20>94,85 24,01 (35,36)
Q 197,20>182,05 (-) 3,51e3

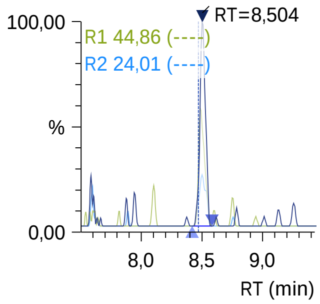

10M
Conc 40,8257
Area 14458
R#1 197,20>122,95 72,80 (87,02)
R#2 197,20>94,85 14,51 (35,36)
Q 197,20>182,05 (-) 2,48e3

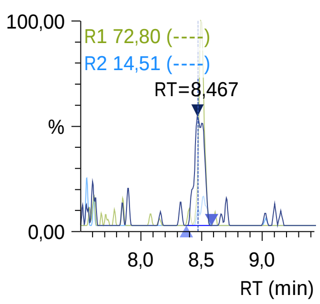

9H
Conc 368,6900
Area 158485
R#1 197,20>122,95 57,83 (87,02)
R#2 197,20>94,85 29,35 (35,36)
Q 197,20>182,05 (-) 4,04e4

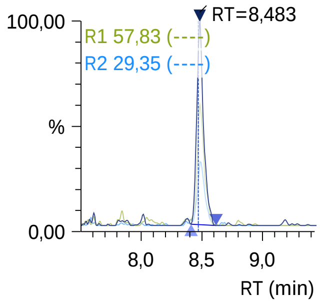

9M
Conc 352,8456
Area 151524
R#1 197,20>122,95 80,25 (87,02)
R#2 197,20>94,85 30,59 (35,36)
Q 197,20>182,05 (-) 3,48e4

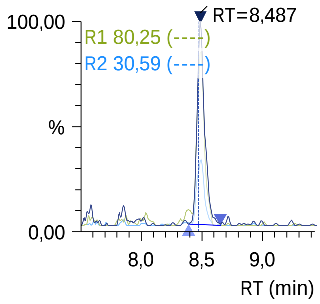

Compound: Ac. Protocatecuico

Curve Fit: Linear | Weighting: None | Zero: Not Forced
Quantitative Method: External Standard
Q 153,20>108,95
R^2 = 0.9941546 R = 0.9970730
y = 1428,893x - 5939,097

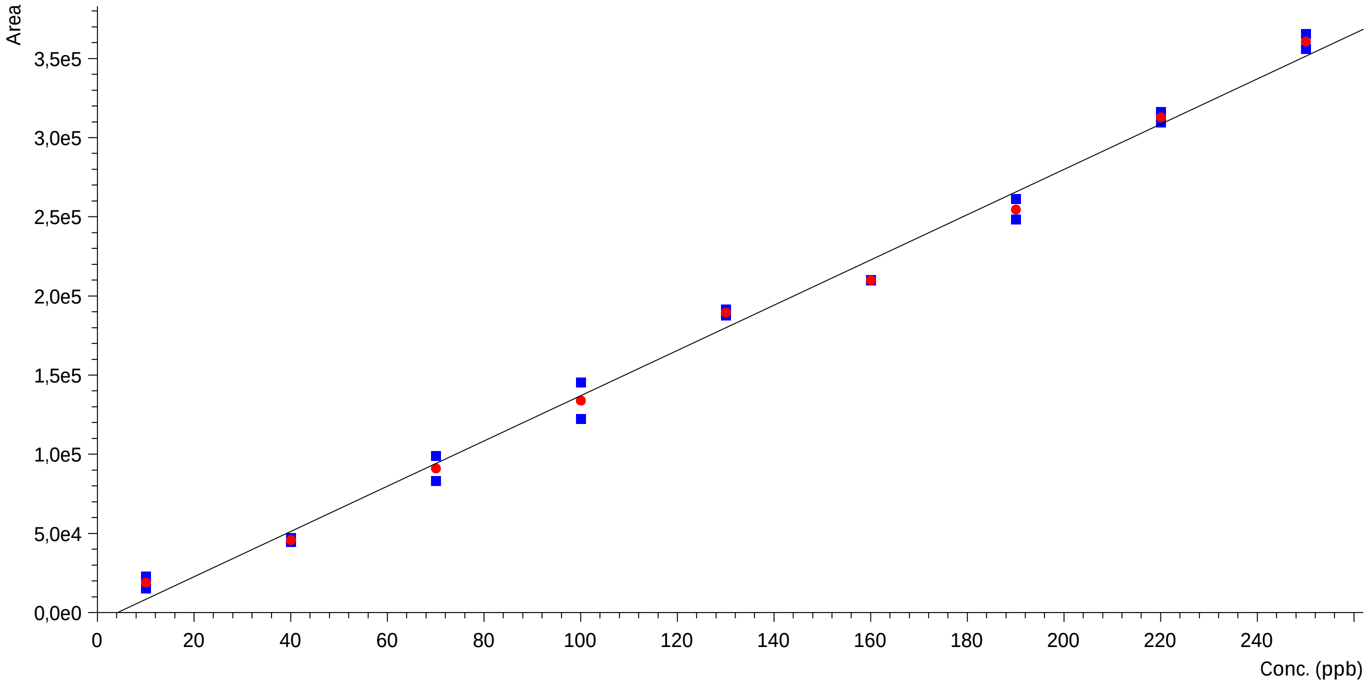

Sample ID	Area	Conc.	Sample Type	Level	Cal Point	Found RT	Ref 1 Actual Ratio
0	----	----	Ctrl.	0	----	----	----
10ppb001	22910	20,1901	Std.	1	Yes	7,562	19,81
10 ppb002	15072	14,7044	Std.	1	Yes	7,560	22,42
40 ppb001	44609	35,3754	Std.	2	Yes	7,561	28,22
40ppb002	47001	37,0499	Std.	2	Yes	7,565	28,69
70 ppb001	99130	73,5320	Std.	3	Yes	7,573	26,05
70ppb002	83299	62,4527	Std.	3	Yes	7,568	27,41
100 ppb001	145580	106,0393	Std.	4	Yes	7,565	25,31
100ppb002	122557	89,9270	Std.	4	Yes	7,580	23,16
130 ppb001	187772	135,5676	Std.	5	Yes	7,570	34,07
130ppb002	191645	138,2778	Std.	5	Yes	7,576	23,13
160 ppb001	209855	151,0218	Std.	6	Yes	7,574	34,65
160ppb002	210168	151,2411	Std.	6	Yes	7,567	37,69
190 ppb001	261113	186,8947	Std.	7	Yes	7,577	28,16
190ppb002	248299	177,9269	Std.	7	Yes	7,571	31,32
220 ppb001	309784	220,9563	Std.	8	Yes	7,568	24,83
220ppb002	316073	225,3579	Std.	8	Yes	7,578	29,65
250 ppb001	365560	259,9907	Std.	9	Yes	7,567	28,92
250 ppb002	356277	253,4945	Std.	9	Yes	7,577	28,16
10H	----	----	Unk.	----	----	----	----
10M	----	----	Unk.	----	----	----	----
9H	213949	153,8868	Unk.	----	----	7,575	24,99
9M	179085	129,4880	Unk.	----	----	7,586	21,93
Average	181487	131,1687				7,571	27,43
%RSD	60	57,8658				0,090	16,65
Maximum	365560	259,9907				7,586	37,69
Minimum	15072	14,7044				7,560	19,81
Std. Dev.	108456	75,9019				0,007	4,57

Compound: Ac. Protocatecuico (continued)

0
Conc ----
Area ----
R#1 153,20>108,05 ---- (28,16)
R#2 153,20>80,80 ---- (6,10)
Q 153,20>108,95 (-) 1,80e3

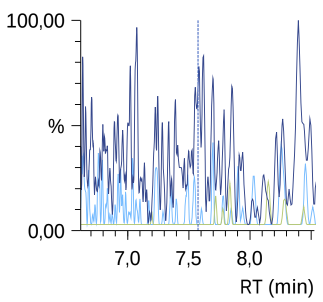

10ppb001
Conc 20,1901
Area 22910
R#1 153,20>108,05 19,81 (28,16)
R#2 153,20>80,80 4,81 (6,10)
Q 153,20>108,95 (-) 5,86e3

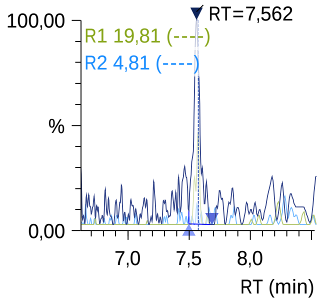

10 ppb002
Conc 14,7044
Area 15072
R#1 153,20>108,05 22,42 (28,16)
R#2 153,20>80,80 0,00 (6,10)
Q 153,20>108,95 (-) 3,59e3

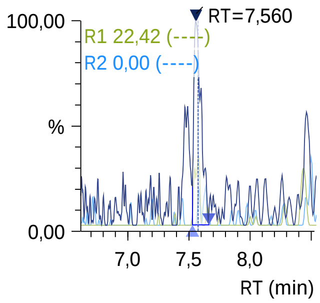

40 ppb001
Conc 35,3754
Area 44609
R#1 153,20>108,05 28,22 (28,16)
R#2 153,20>80,80 1,23 (6,10)
Q 153,20>108,95 (-) 1,99e4

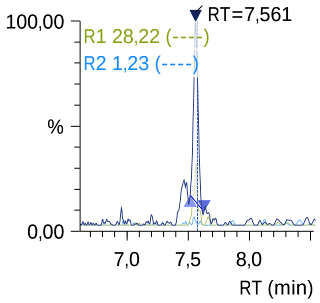

40ppb002
Conc 37,0499
Area 47001
R#1 153,20>108,05 28,69 (28,16)
R#2 153,20>80,80 3,43 (6,10)
Q 153,20>108,95 (-) 1,50e4

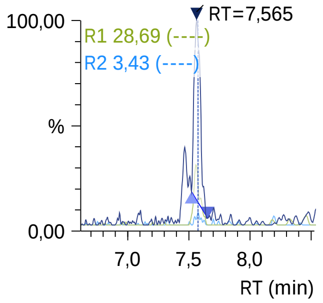

70 ppb001
Conc 73,5320
Area 99130
R#1 153,20>108,05 26,05 (28,16)
R#2 153,20>80,80 3,07 (6,10)
Q 153,20>108,95 (-) 3,50e4

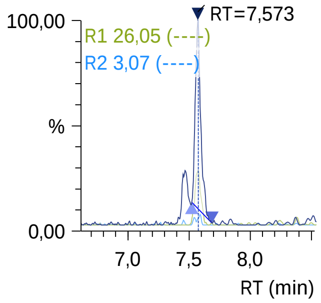

70ppb002
Conc 62,4527
Area 83299
R#1 153,20>108,05 27,41 (28,16)
R#2 153,20>80,80 1,88 (6,10)
Q 153,20>108,95 (-) 3,02e4

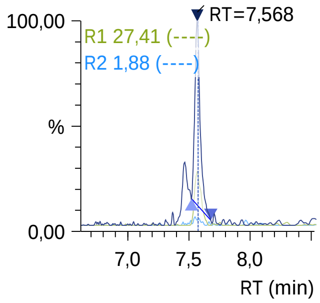

100 ppb001
Conc 106,0393
Area 145580
R#1 153,20>108,05 25,31 (28,16)
R#2 153,20>80,80 4,74 (6,10)
Q 153,20>108,95 (-) 4,45e4

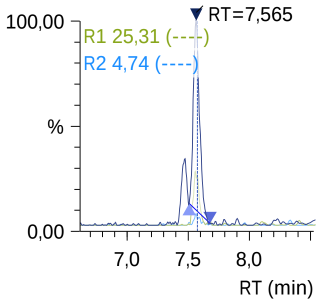

100ppb002
Conc 89,9270
Area 122557
R#1 153,20>108,05 23,16 (28,16)
R#2 153,20>80,80 6,29 (6,10)
Q 153,20>108,95 (-) 4,36e4

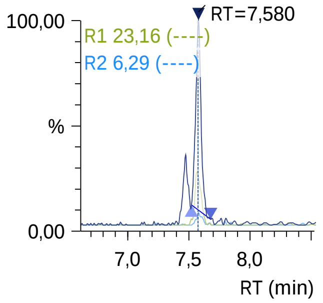

130 ppb001
Conc 135,5676
Area 187772
R#1 153,20>108,05 34,07 (28,16)
R#2 153,20>80,80 3,51 (6,10)
Q 153,20>108,95 (-) 5,55e4

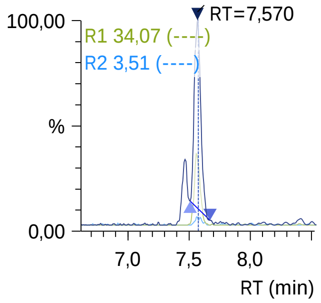

130ppb002
Conc 138,2778
Area 191645
R#1 153,20>108,05 23,13 (28,16)
R#2 153,20>80,80 4,78 (6,10)
Q 153,20>108,95 (-) 6,29e4

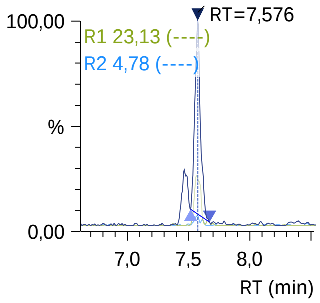

160 ppb001
Conc 151,0218
Area 209855
R#1 153,20>108,05 34,65 (28,16)
R#2 153,20>80,80 4,81 (6,10)
Q 153,20>108,95 (-) 6,85e4

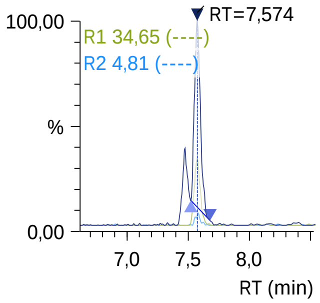

Compound: Ac. Protocatecuico (continued)

160ppb002
Conc 151,2411
Area 210168
R#1 153,20>108,05 37,69 (28,16)
R#2 153,20>80,80 5,40 (6,10)
Q 153,20>108,95 (-) 6,83e4

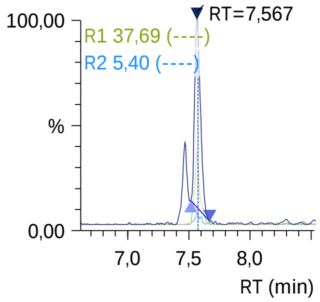

190 ppb001
Conc 186,8947
Area 261113
R#1 153,20>108,05 28,16 (28,16)
R#2 153,20>80,80 2,26 (6,10)
Q 153,20>108,95 (-) 8,80e4

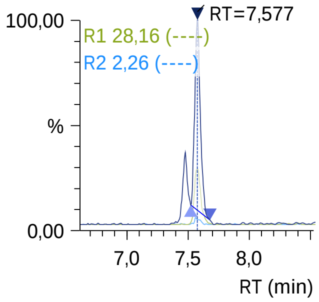

190ppb002
Conc 177,9269
Area 248299
R#1 153,20>108,05 31,32 (28,16)
R#2 153,20>80,80 5,57 (6,10)
Q 153,20>108,95 (-) 8,11e4

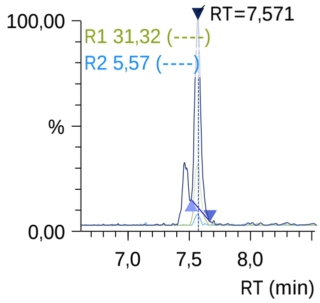

220 ppb001
Conc 220,9563
Area 309784
R#1 153,20>108,05 24,83 (28,16)
R#2 153,20>80,80 4,14 (6,10)
Q 153,20>108,95 (-) 1,04e5

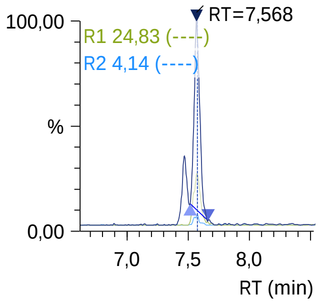

220ppb002
Conc 225,3579
Area 316073
R#1 153,20>108,05 29,65 (28,16)
R#2 153,20>80,80 4,73 (6,10)
Q 153,20>108,95 (-) 1,03e5

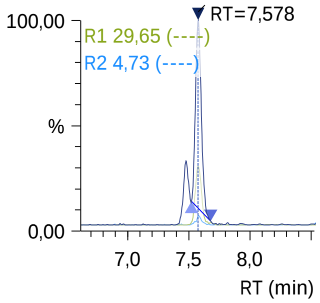

250 ppb001
Conc 259,9907
Area 365560
R#1 153,20>108,05 28,92 (28,16)
R#2 153,20>80,80 3,78 (6,10)
Q 153,20>108,95 (-) 1,22e5

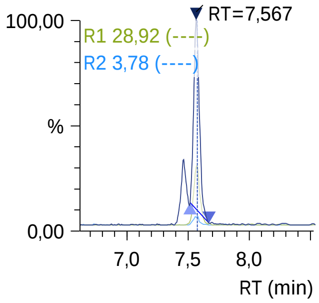

250 ppb002
Conc 253,4945
Area 356277
R#1 153,20>108,05 28,16 (28,16)
R#2 153,20>80,80 6,10 (6,10)
Q 153,20>108,95 (-) 1,06e5

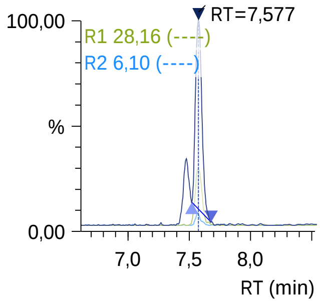

10H
Conc ----
Area ----
R#1 153,20>108,05 ---- (28,16)
R#2 153,20>80,80 ---- (6,10)
Q 153,20>108,95 (-) 1,33e4

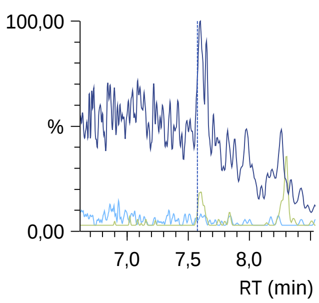

10M
Conc ----
Area ----
R#1 153,20>108,05 ---- (28,16)
R#2 153,20>80,80 ---- (6,10)
Q 153,20>108,95 (-) 1,30e4

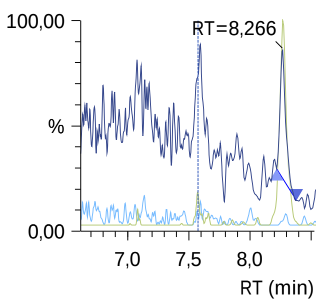

9H
Conc 153,8868
Area 213949
R#1 153,20>108,05 24,99 (28,16)
R#2 153,20>80,80 3,32 (6,10)
Q 153,20>108,95 (-) 5,94e4

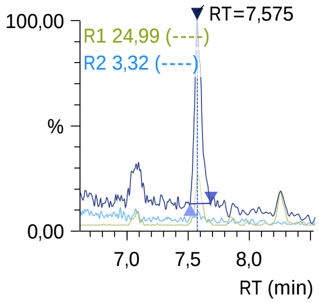

9M
Conc 129,4880
Area 179085
R#1 153,20>108,05 21,93 (28,16)
R#2 153,20>80,80 5,03 (6,10)
Q 153,20>108,95 (-) 5,40e4

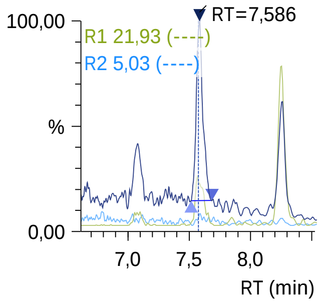

Compound: Ac. Vanilico

Curve Fit: Linear | Weighting: None | Zero: Not Forced
Quantitative Method: External Standard
Q 167,40>152,00
R^2 = 0.9870441 R = 0.9935009
y = 363,8030x - 6538,059

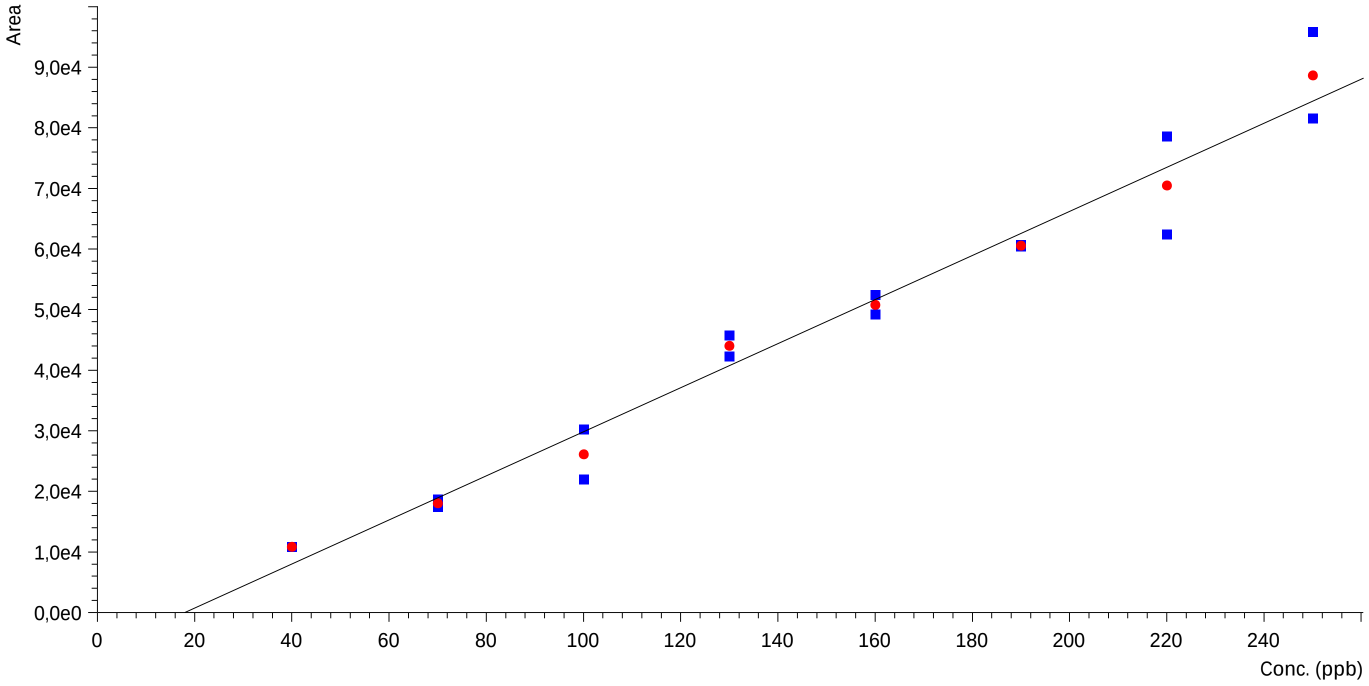

Sample ID	Area	Conc.	Sample Type	Level	Cal Point	Found RT	Ref 1 Actual Ratio
0	----	----	Ctrl.	0	----	----	----
10ppb001	----	----	Std.	1	No	----	----
10 ppb002	----	----	Std.	1	No	----	----
40 ppb001	----	----	Std.	2	No	----	----
40ppb002	10898	47,9286	Std.	2	Yes	8,426	62,00
70 ppb001	18687	69,3380	Std.	3	Yes	8,411	128,01
70ppb002	17449	65,9339	Std.	3	Yes	8,412	151,38
100 ppb001	30258	101,1422	Std.	4	Yes	8,427	117,57
100ppb002	22029	78,5224	Std.	4	Yes	8,418	204,05
130 ppb001	42278	134,1830	Std.	5	Yes	8,419	142,62
130ppb002	45795	143,8486	Std.	5	Yes	8,416	120,21
160 ppb001	49213	153,2464	Std.	6	Yes	8,427	152,35
160ppb002	52387	161,9696	Std.	6	Yes	8,413	103,03
190 ppb001	60667	184,7289	Std.	7	Yes	8,425	128,08
190ppb002	60450	184,1325	Std.	7	Yes	8,407	135,58
220 ppb001	62438	189,5959	Std.	8	Yes	8,419	149,08
220ppb002	78575	233,9524	Std.	8	Yes	8,416	128,02
250 ppb001	81549	242,1282	Std.	9	Yes	8,409	111,69
250 ppb002	95844	281,4208	Std.	9	Yes	8,419	98,55
10H	----	----	Unk.	----	----	----	----
10M	----	----	Unk.	----	----	----	----
9H	53928	166,2055	Unk.	----	----	8,427	74,14
9M	47531	148,6227	Unk.	----	----	8,436	71,19
Average	48822	152,1706				8,419	122,21
%RSD	49	43,1116				0,094	28,55
Maximum	95844	281,4208				8,436	204,05
Minimum	10898	47,9286				8,407	62,00
Std. Dev.	23867	65,6032				0,008	34,89

Compound: Ac. Vanilico (continued)

0
Conc ----
Area ----
R#1 167,40>107,95 ---- (98,55)
R#2 167,40>123,00 ---- (38,88)
Q 167,40>152,00 (-) 7,22e2

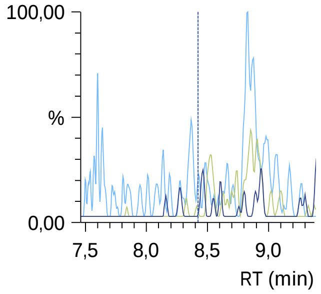

10ppb001
Conc ----
Area ----
R#1 167,40>107,95 ---- (98,55)
R#2 167,40>123,00 ---- (38,88)
Q 167,40>152,00 (-) 1,31e3

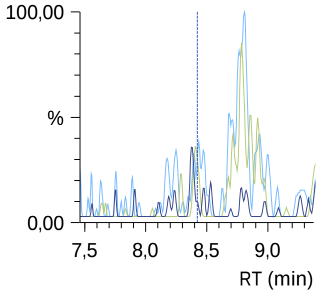

10 ppb002
Conc ----
Area ----
R#1 167,40>107,95 ---- (98,55)
R#2 167,40>123,00 ---- (38,88)
Q 167,40>152,00 (-) 1,41e3

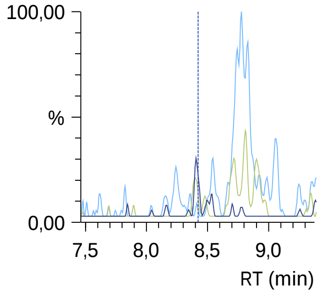

40 ppb001
Conc ----
Area ----
R#1 167,40>107,95 ---- (98,55)
R#2 167,40>123,00 ---- (38,88)
Q 167,40>152,00 (-) 2,16e3

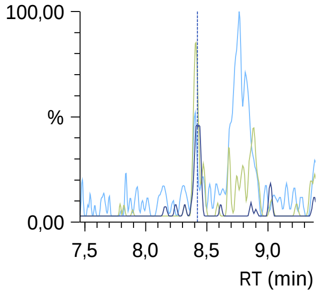

40ppb002
Conc 47,9286
Area 10898
R#1 167,40>107,95 62,00 (98,55)
R#2 167,40>123,00 43,15 (38,88)
Q 167,40>152,00 (-) 2,47e3

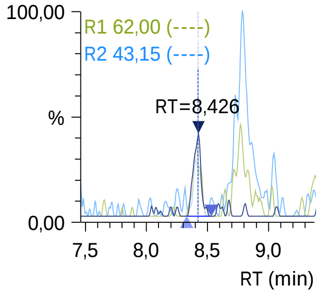

70 ppb001
Conc 69,3380
Area 18687
R#1 167,40>107,95 128,01 (98,55)
R#2 167,40>123,00 20,13 (38,88)
Q 167,40>152,00 (-) 4,78e3

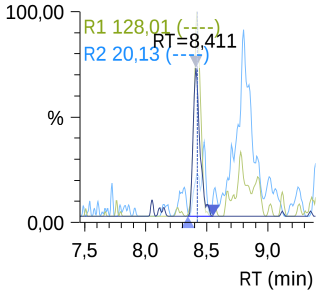

70ppb002
Conc 65,9339
Area 17449
R#1 167,40>107,95 151,38 (98,55)
R#2 167,40>123,00 15,99 (38,88)
Q 167,40>152,00 (-) 4,14e3

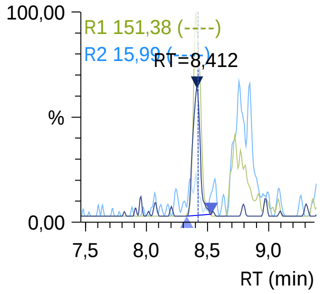

100 ppb001
Conc 101,1422
Area 30258
R#1 167,40>107,95 117,57 (98,55)
R#2 167,40>123,00 22,00 (38,88)
Q 167,40>152,00 (-) 8,42e3

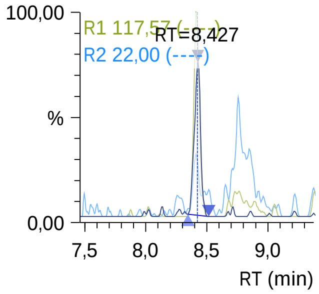

100ppb002
Conc 78,5224
Area 22029
R#1 167,40>107,95 204,05 (98,55)
R#2 167,40>123,00 43,83 (38,88)
Q 167,40>152,00 (-) 5,32e3

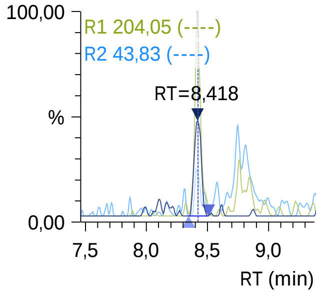

130 ppb001
Conc 134,1830
Area 42278
R#1 167,40>107,95 142,62 (98,55)
R#2 167,40>123,00 22,33 (38,88)
Q 167,40>152,00 (-) 9,89e3

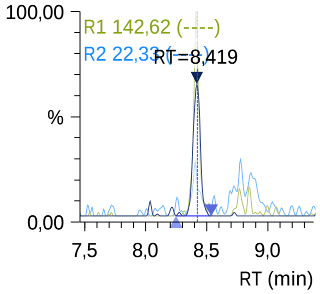

130ppb002
Conc 143,8486
Area 45795
R#1 167,40>107,95 120,21 (98,55)
R#2 167,40>123,00 30,04 (38,88)
Q 167,40>152,00 (-) 1,27e4

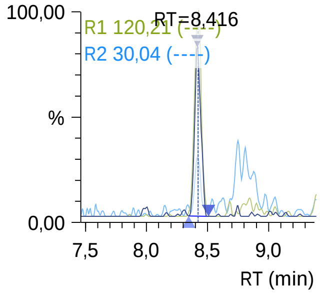

160 ppb001
Conc 153,2464
Area 49213
R#1 167,40>107,95 152,35 (98,55)
R#2 167,40>123,00 15,62 (38,88)
Q 167,40>152,00 (-) 1,30e4

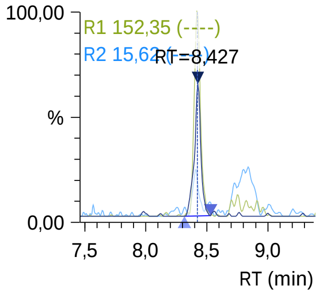

Compound: Ac. Vanilico (continued)

160ppb002
Conc 161,9696
Area 52387
R#1 167,40>107,95 103,03 (98,55)
R#2 167,40>123,00 38,78 (38,88)
Q 167,40>152,00 (-) 1,41e4

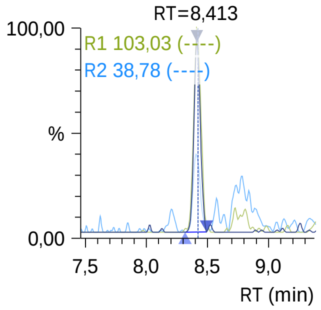

190 ppb001
Conc 184,7289
Area 60667
R#1 167,40>107,95 128,08 (98,55)
R#2 167,40>123,00 31,29 (38,88)
Q 167,40>152,00 (-) 1,61e4

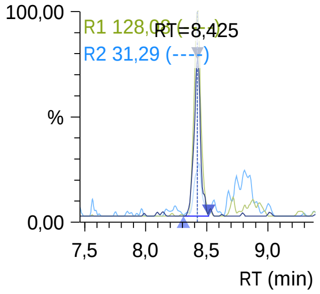

190ppb002
Conc 184,1325
Area 60450
R#1 167,40>107,95 135,58 (98,55)
R#2 167,40>123,00 28,83 (38,88)
Q 167,40>152,00 (-) 1,59e4

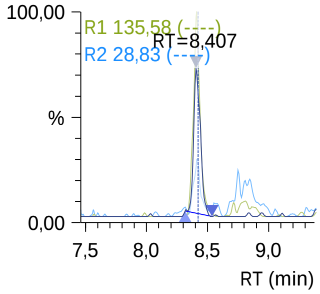

220 ppb001
Conc 189,5959
Area 62438
R#1 167,40>107,95 149,08 (98,55)
R#2 167,40>123,00 49,48 (38,88)
Q 167,40>152,00 (-) 1,45e4

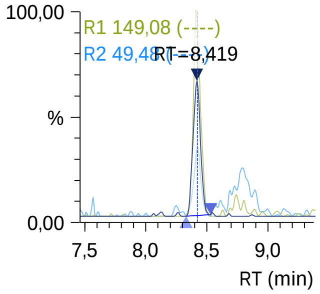

220ppb002
Conc 233,9524
Area 78575
R#1 167,40>107,95 128,02 (98,55)
R#2 167,40>123,00 29,25 (38,88)
Q 167,40>152,00 (-) 2,17e4

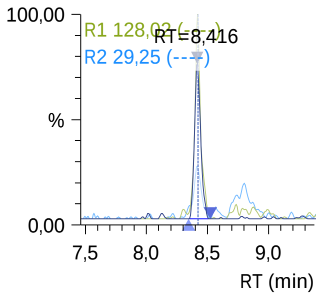

250 ppb001
Conc 242,1282
Area 81549
R#1 167,40>107,95 111,69 (98,55)
R#2 167,40>123,00 32,26 (38,88)
Q 167,40>152,00 (-) 2,19e4

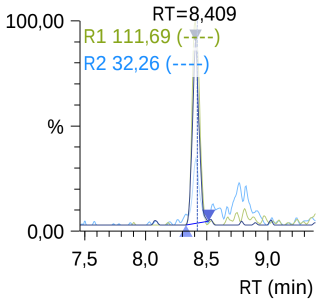

250 ppb002
Conc 281,4208
Area 95844
R#1 167,40>107,95 98,55 (98,55)
R#2 167,40>123,00 38,88 (38,88)
Q 167,40>152,00 (-) 2,40e4

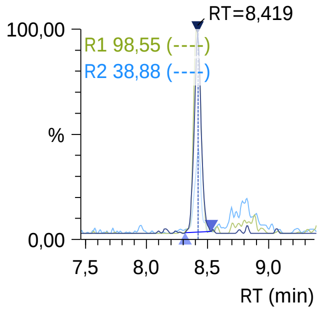

10H
Conc ----
Area ----
R#1 167,40>107,95 ---- (98,55)
R#2 167,40>123,00 ---- (38,88)
Q 167,40>152,00 (-) 2,28e3

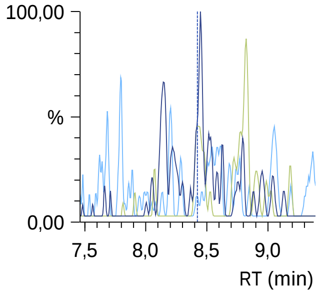

10M
Conc ----
Area ----
R#1 167,40>107,95 ---- (98,55)
R#2 167,40>123,00 ---- (38,88)
Q 167,40>152,00 (-) 2,37e3

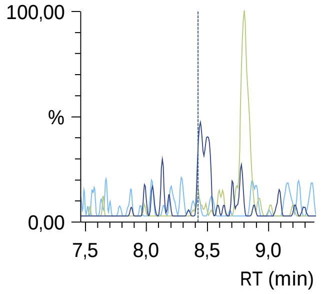

9H
Conc 166,2055
Area 53928
R#1 167,40>107,95 74,14 (98,55)
R#2 167,40>123,00 18,61 (38,88)
Q 167,40>152,00 (-) 1,27e4

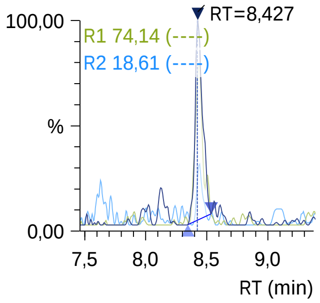

9M
Conc 148,6227
Area 47531
R#1 167,40>107,95 71,19 (98,55)
R#2 167,40>123,00 12,16 (38,88)
Q 167,40>152,00 (-) 1,15e4

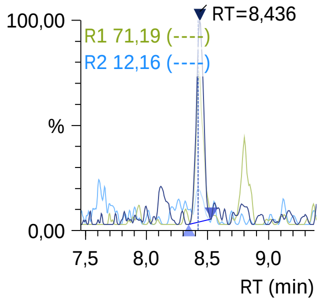

Compound: Ac. Salicilico

Curve Fit: Linear | Weighting: None | Zero: Not Forced
Quantitative Method: External Standard
Q 137,40>92,90
R^2 = 0.3403895 R = 0.5834291
y = 8018,649x + 4478111

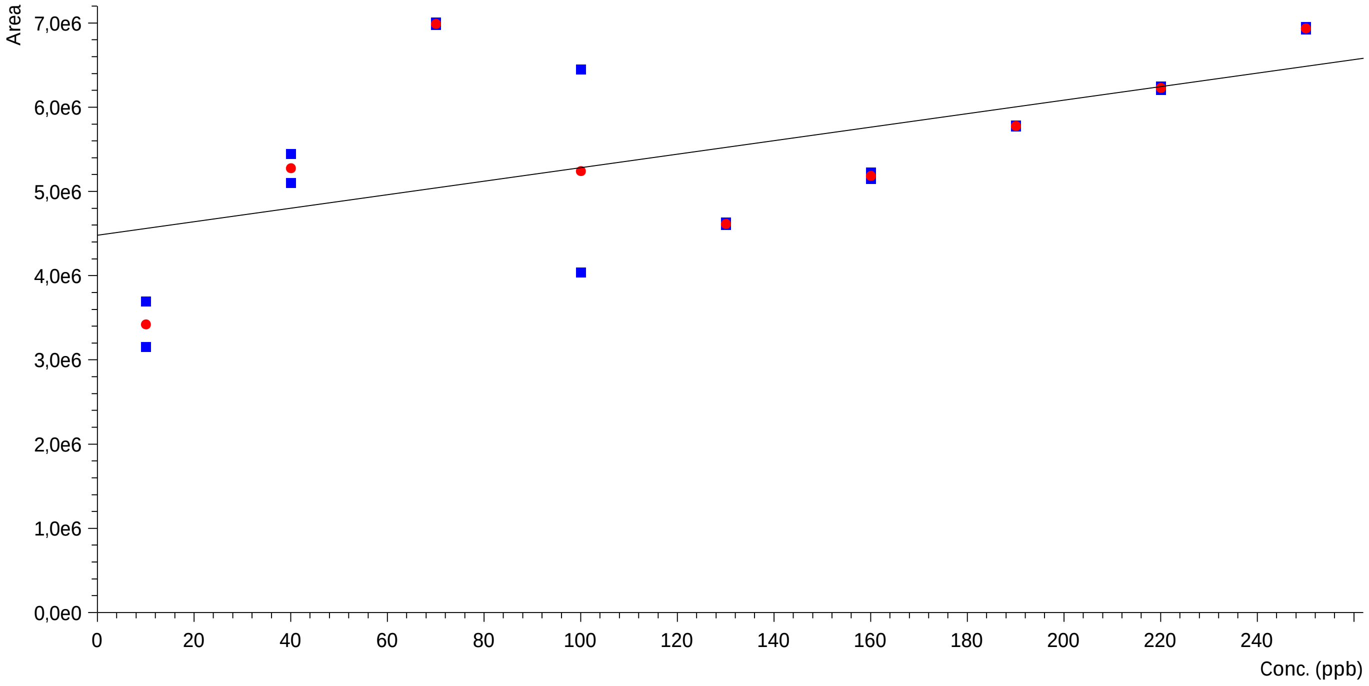

Sample ID	Area	Conc.	Sample Type	Level	Cal Point	Found RT	Ref 1 Actual Ratio
0	1308529	-395,2763	Ctrl.	0	----	9,881	9,82
10ppb001	3153092	-165,2422	Std.	1	Yes	9,846	9,00
10 ppb002	3694819	-97,6838	Std.	1	Yes	9,840	9,84
40 ppb001	5103387	77,9777	Std.	2	Yes	9,835	9,52
40ppb002	5449721	121,1688	Std.	2	Yes	9,839	10,25
70 ppb001	6974358	311,3052	Std.	3	Yes	9,835	9,26
70ppb002	7007436	315,4303	Std.	3	Yes	9,839	9,72
100 ppb001	6449219	245,8154	Std.	4	Yes	9,842	9,93
100ppb002	4038326	-54,8453	Std.	4	Yes	9,829	10,14
130 ppb001	4630308	18,9804	Std.	5	Yes	9,830	10,04
130ppb002	4604138	15,7167	Std.	5	Yes	9,834	9,31
160 ppb001	5149932	83,7824	Std.	6	Yes	9,835	8,95
160ppb002	5223227	92,9229	Std.	6	Yes	9,830	9,27
190 ppb001	5784040	162,8614	Std.	7	Yes	9,839	9,98
190ppb002	5771499	161,2975	Std.	7	Yes	9,829	9,43
220 ppb001	6208209	215,7593	Std.	8	Yes	9,830	8,71
220ppb002	6251332	221,1371	Std.	8	Yes	9,833	10,09
250 ppb001	6953221	308,6691	Std.	9	Yes	9,827	8,62
250 ppb002	6923374	304,9470	Std.	9	Yes	9,831	9,19
10H	1459215	-376,4844	Unk.	----	----	9,812	9,58
10M	1551267	-365,0047	Unk.	----	----	9,804	10,31
9H	2868700	-200,7085	Unk.	----	----	9,798	11,13
9M	3108537	-170,7986	Unk.	----	----	9,792	10,29
Average	4768082	36,1621				9,831	9,67
%RSD	38	629,6449				0,180	6,15
Maximum	7007436	315,4303				9,881	11,13
Minimum	1308529	-395,2763				9,792	8,62
Std. Dev.	1825787	227,6926				0,018	0,59

Compound: Ac. Salicilico (continued)

0
Conc -395,2763
Area 1308529
R#1 137,40>65,00 9,82 (10,07)
R#2 137,40>74,85 0,79 (1,28)
Q 137,40>92,90 (-) 1,58e5

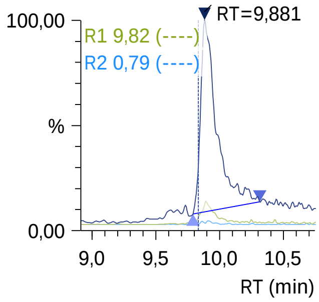

10ppb001
Conc -165,2422
Area 3153092
R#1 137,40>65,00 9,00 (10,07)
R#2 137,40>74,85 1,05 (1,28)
Q 137,40>92,90 (-) 3,11e5

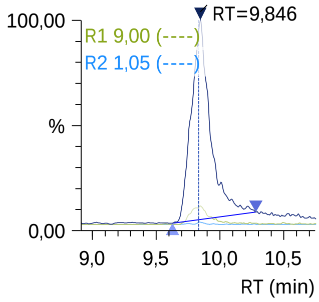

10 ppb002
Conc -97,6838
Area 3694819
R#1 137,40>65,00 9,84 (10,07)
R#2 137,40>74,85 0,73 (1,28)
Q 137,40>92,90 (-) 3,68e5

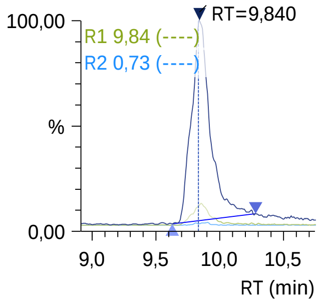

40 ppb001
Conc 77,9777
Area 5103387
R#1 137,40>65,00 9,52 (10,07)
R#2 137,40>74,85 1,08 (1,28)
Q 137,40>92,90 (-) 6,48e5

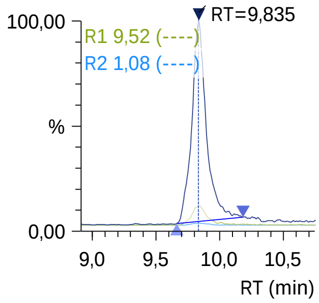

40ppb002
Conc 121,1688
Area 5449721
R#1 137,40>65,00 10,25 (10,07)
R#2 137,40>74,85 1,14 (1,28)
Q 137,40>92,90 (-) 6,98e5

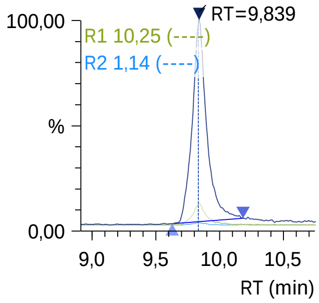

70 ppb001
Conc 311,3052
Area 6974358
R#1 137,40>65,00 9,26 (10,07)
R#2 137,40>74,85 1,03 (1,28)
Q 137,40>92,90 (-) 9,82e5

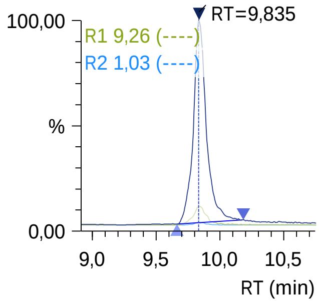

70ppb002
Conc 315,4303
Area 7007436
R#1 137,40>65,00 9,72 (10,07)
R#2 137,40>74,85 1,08 (1,28)
Q 137,40>92,90 (-) 1,04e6

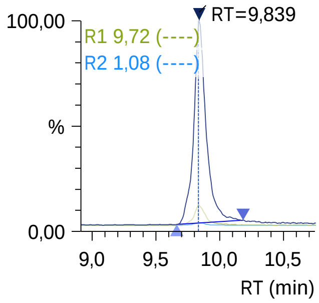

100 ppb001
Conc 245,8154
Area 6449219
R#1 137,40>65,00 9,93 (10,07)
R#2 137,40>74,85 1,00 (1,28)
Q 137,40>92,90 (-) 9,54e5

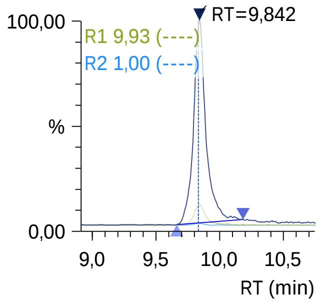

100ppb002
Conc -54,8453
Area 4038326
R#1 137,40>65,00 10,14 (10,07)
R#2 137,40>74,85 0,82 (1,28)
Q 137,40>92,90 (-) 6,02e5

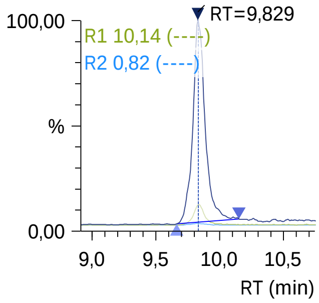

130 ppb001
Conc 18,9804
Area 4630308
R#1 137,40>65,00 10,04 (10,07)
R#2 137,40>74,85 1,28 (1,28)
Q 137,40>92,90 (-) 7,31e5

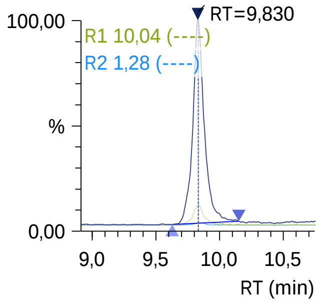

130ppb002
Conc 15,7167
Area 4604138
R#1 137,40>65,00 9,31 (10,07)
R#2 137,40>74,85 1,27 (1,28)
Q 137,40>92,90 (-) 7,24e5

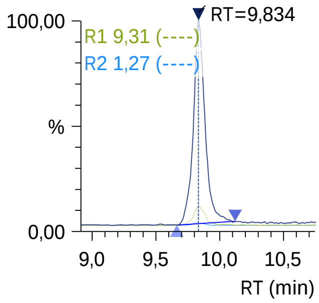

160 ppb001
Conc 83,7824
Area 5149932
R#1 137,40>65,00 8,95 (10,07)
R#2 137,40>74,85 1,20 (1,28)
Q 137,40>92,90 (-) 8,25e5

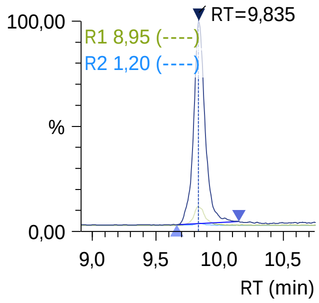

Compound: Ac. Salicilico (continued)

160ppb002
Conc 92,9229
Area 5223227
R#1 137,40>65,00 9,27 (10,07)
R#2 137,40>74,85 1,13 (1,28)
Q 137,40>92,90 (-) 8,29e5

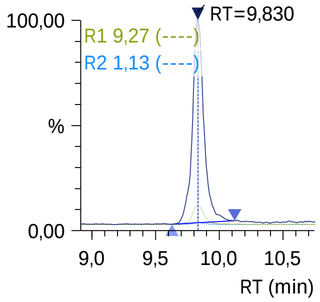

190 ppb001
Conc 162,8614
Area 5784040
R#1 137,40>65,00 9,98 (10,07)
R#2 137,40>74,85 1,61 (1,28)
Q 137,40>92,90 (-) 9,72e5

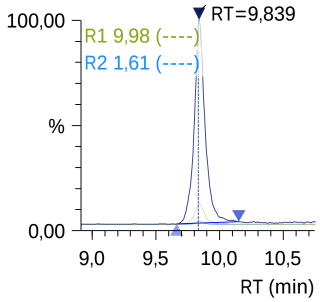

190ppb002
Conc 161,2975
Area 5771499
R#1 137,40>65,00 9,43 (10,07)
R#2 137,40>74,85 1,22 (1,28)
Q 137,40>92,90 (-) 9,83e5

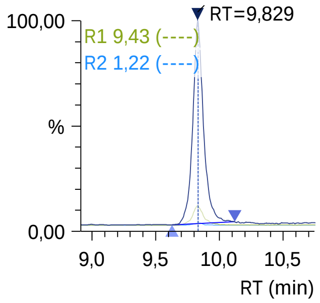

220 ppb001
Conc 215,7593
Area 6208209
R#1 137,40>65,00 8,71 (10,07)
R#2 137,40>74,85 1,07 (1,28)
Q 137,40>92,90 (-) 1,06e6

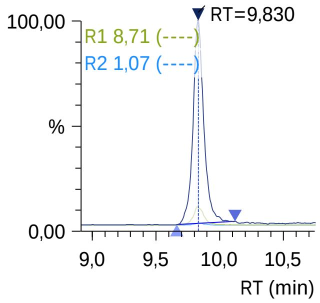

220ppb002
Conc 221,1371
Area 6251332
R#1 137,40>65,00 10,09 (10,07)
R#2 137,40>74,85 1,26 (1,28)
Q 137,40>92,90 (-) 1,03e6

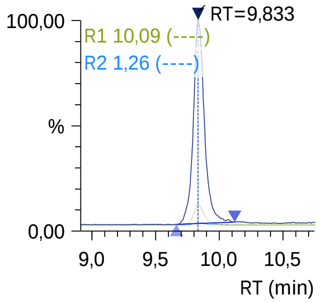

250 ppb001
Conc 308,6691
Area 6953221
R#1 137,40>65,00 8,62 (10,07)
R#2 137,40>74,85 0,95 (1,28)
Q 137,40>92,90 (-) 1,17e6

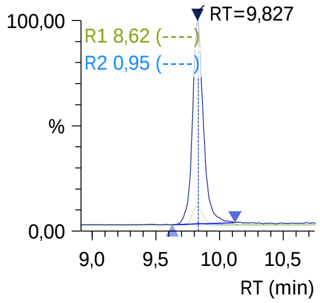

250 ppb002
Conc 304,9470
Area 6923374
R#1 137,40>65,00 9,19 (10,07)
R#2 137,40>74,85 1,26 (1,28)
Q 137,40>92,90 (-) 1,19e6

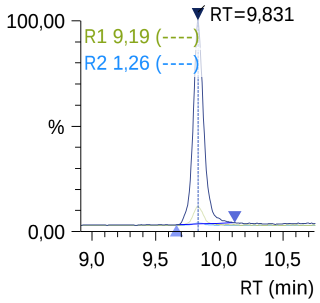

10H
Conc -376,4844
Area 1459215
R#1 137,40>65,00 9,58 (10,07)
R#2 137,40>74,85 1,22 (1,28)
Q 137,40>92,90 (-) 1,61e5

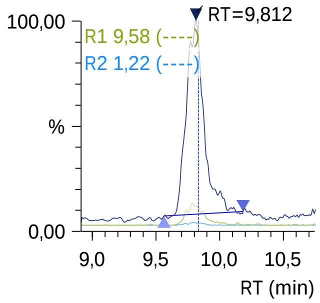

10M
Conc -365,0047
Area 1551267
R#1 137,40>65,00 10,31 (10,07)
R#2 137,40>74,85 0,64 (1,28)
Q 137,40>92,90 (-) 1,58e5

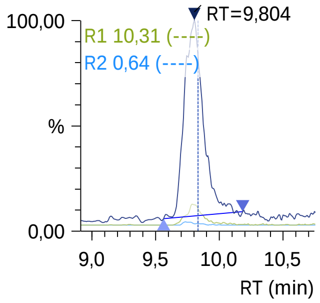

9H
Conc -200,7085
Area 2868700
R#1 137,40>65,00 11,13 (10,07)
R#2 137,40>74,85 1,50 (1,28)
Q 137,40>92,90 (-) 4,12e5

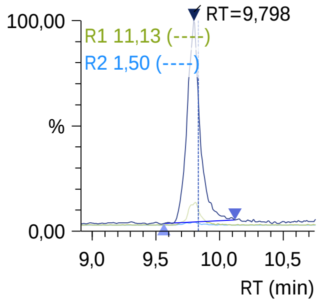

9M
Conc -170,7986
Area 3108537
R#1 137,40>65,00 10,29 (10,07)
R#2 137,40>74,85 1,06 (1,28)
Q 137,40>92,90 (-) 4,55e5

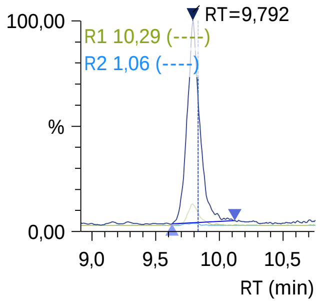

Compound: Vanilina

Curve Fit: Linear | Weighting: None | Zero: Not Forced
Quantitative Method: External Standard
Q 151,40>136,05
R^2 = 0.9804180 R = 0.9901606
y = 344,7863x + 74949,51

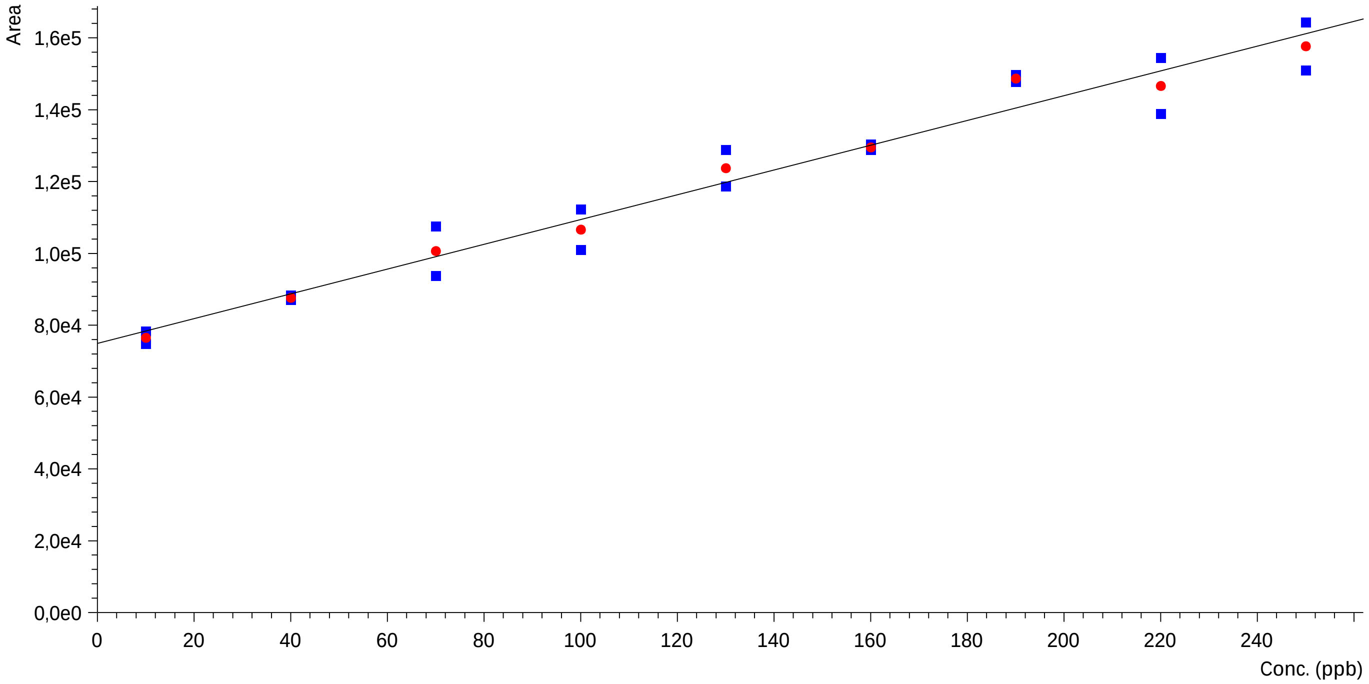

Sample ID	Area	Conc.	Sample Type	Level	Cal Point	Found RT	Ref 1 Actual Ratio
0	48509	-76,6874	Ctrl.	0	----	8,787	35,08
10ppb001	74836	-0,3306	Std.	1	Yes	8,730	20,00
10 ppb002	78280	9,6599	Std.	1	Yes	8,779	16,49
40 ppb001	87066	35,1427	Std.	2	Yes	8,726	0,00
40ppb002	88299	38,7192	Std.	2	Yes	8,713	0,00
70 ppb001	107537	94,5159	Std.	3	Yes	8,730	0,00
70ppb002	93740	54,4997	Std.	3	Yes	8,748	48,33
100 ppb001	101027	75,6352	Std.	4	Yes	8,737	21,27
100ppb002	112263	108,2214	Std.	4	Yes	8,730	18,78
130 ppb001	118689	126,8599	Std.	5	Yes	8,724	45,12
130ppb002	128832	156,2781	Std.	5	Yes	8,730	0,00
160 ppb001	128808	156,2095	Std.	6	Yes	8,727	5,22
160ppb002	130349	160,6777	Std.	6	Yes	8,724	17,18
190 ppb001	147719	211,0560	Std.	7	Yes	8,725	27,58
190ppb002	149726	216,8788	Std.	7	Yes	8,715	34,09
220 ppb001	138784	185,1421	Std.	8	Yes	8,728	36,12
220ppb002	154502	230,7311	Std.	8	Yes	8,727	8,13
250 ppb001	164366	259,3387	Std.	9	Yes	8,720	27,90
250 ppb002	151066	220,7648	Std.	9	Yes	8,726	38,58
10H	91624	48,3630	Unk.	----	----	8,772	48,63
10M	84055	26,4083	Unk.	----	----	8,758	30,49
9H	85669	31,0903	Unk.	----	----	8,775	31,05
9M	84921	28,9197	Unk.	----	----	8,747	54,12
Average	110899	104,2650				8,738	24,53
%RSD	28	86,4325				0,246	69,05
Maximum	164366	259,3387				8,787	54,12
Minimum	48509	-76,6874				8,713	0,00
Std. Dev.	31072	90,1188				0,021	16,94

Compound: Vanilina (continued)

0
Conc -76,6874
Area 48509
R#1 151,40>92,05 35,08 (17,18)
R#2 151,40>108,10 15,32 (10,23)
Q 151,40>136,05 (-) 6,54e3

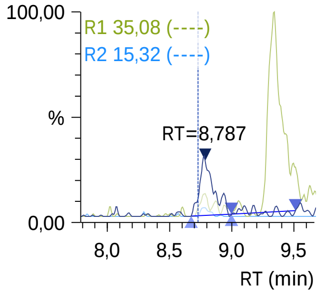

10ppb001
Conc -0,3306
Area 74836
R#1 151,40>92,05 20,00 (17,18)
R#2 151,40>108,10 16,81 (10,23)
Q 151,40>136,05 (-) 6,58e3

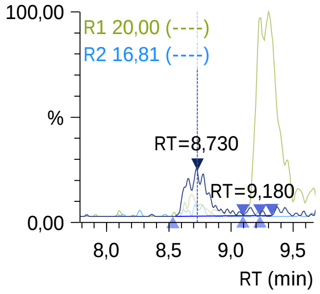

10 ppb002
Conc 9,6599
Area 78280
R#1 151,40>92,05 16,49 (17,18)
R#2 151,40>108,10 15,42 (10,23)
Q 151,40>136,05 (-) 7,26e3

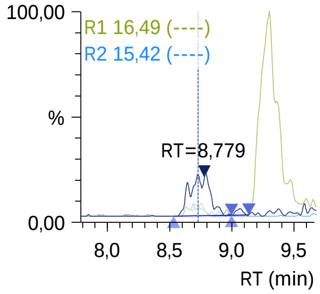

40 ppb001
Conc 35,1427
Area 87066
R#1 151,40>92,05 0,00 (17,18)
R#2 151,40>108,10 12,42 (10,23)
Q 151,40>136,05 (-) 9,19e3

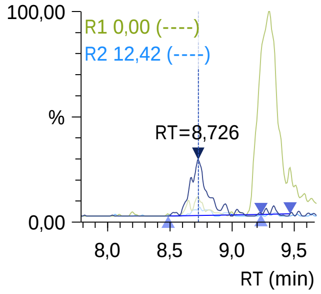

40ppb002
Conc 38,7192
Area 88299
R#1 151,40>92,05 0,00 (17,18)
R#2 151,40>108,10 18,77 (10,23)
Q 151,40>136,05 (-) 8,19e3

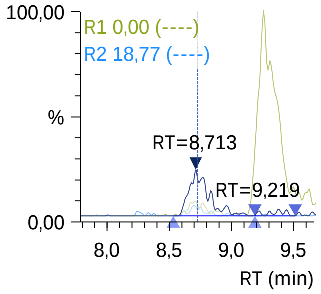

70 ppb001
Conc 94,5159
Area 107537
R#1 151,40>92,05 0,00 (17,18)
R#2 151,40>108,10 16,48 (10,23)
Q 151,40>136,05 (-) 1,05e4

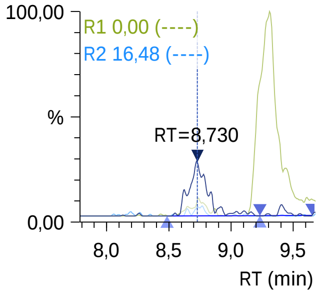

70ppb002
Conc 54,4997
Area 93740
R#1 151,40>92,05 48,33 (17,18)
R#2 151,40>108,10 14,92 (10,23)
Q 151,40>136,05 (-) 1,01e4

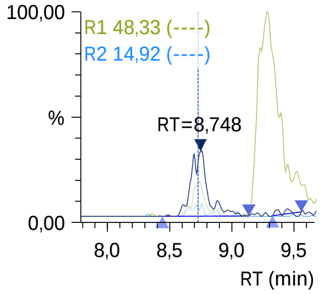

100 ppb001
Conc 75,6352
Area 101027
R#1 151,40>92,05 21,27 (17,18)
R#2 151,40>108,10 14,91 (10,23)
Q 151,40>136,05 (-) 1,38e4

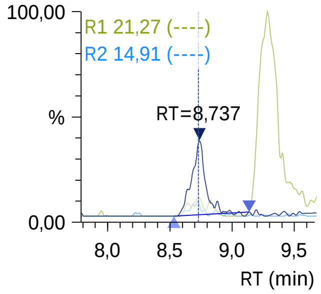

100ppb002
Conc 108,2214
Area 112263
R#1 151,40>92,05 18,78 (17,18)
R#2 151,40>108,10 18,63 (10,23)
Q 151,40>136,05 (-) 1,41e4

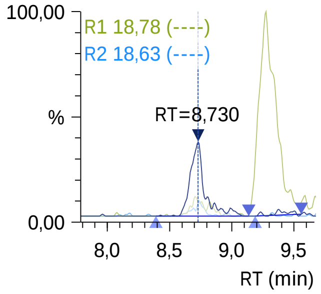

130 ppb001
Conc 126,8599
Area 118689
R#1 151,40>92,05 45,12 (17,18)
R#2 151,40>108,10 24,23 (10,23)
Q 151,40>136,05 (-) 1,36e4

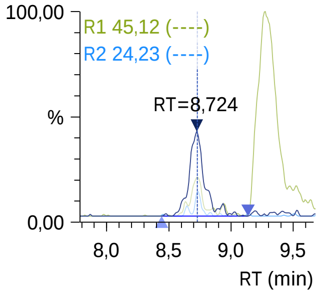

130ppb002
Conc 156,2781
Area 128832
R#1 151,40>92,05 0,00 (17,18)
R#2 151,40>108,10 20,73 (10,23)
Q 151,40>136,05 (-) 1,60e4

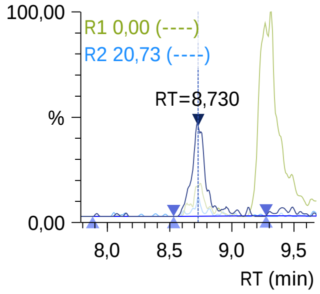

160 ppb001
Conc 156,2095
Area 128808
R#1 151,40>92,05 5,22 (17,18)
R#2 151,40>108,10 11,21 (10,23)
Q 151,40>136,05 (-) 1,76e4

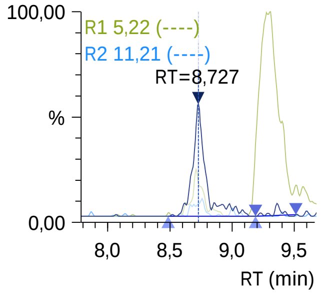

Compound: Vanilina (continued)

160ppb002
Conc 160,6777
Area 130349
R#1 151,40>92,05 17,18 (17,18)
R#2 151,40>108,10 10,23 (10,23)
Q 151,40>136,05 (-)   1,89e4

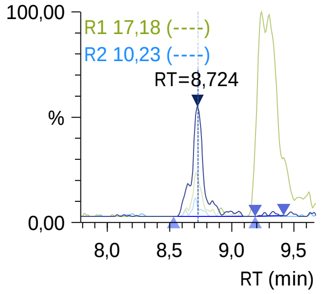

190 ppb001
Conc 211,0560
Area 147719
R#1 151,40>92,05 27,58 (17,18)
R#2 151,40>108,10 11,32 (10,23)
Q 151,40>136,05 (-)   2,12e4

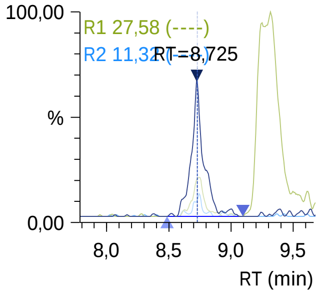

190ppb002
Conc 216,8788
Area 149726
R#1 151,40>92,05 34,09 (17,18)
R#2 151,40>108,10 12,42 (10,23)
Q 151,40>136,05 (-)   2,08e4

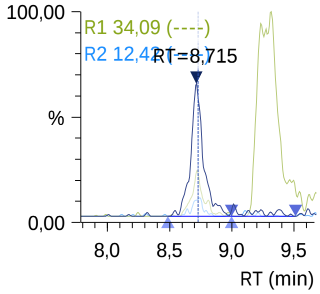

220 ppb001
Conc 185,1421
Area 138784
R#1 151,40>92,05 36,12 (17,18)
R#2 151,40>108,10 20,77 (10,23)
Q 151,40>136,05 (-)   2,04e4

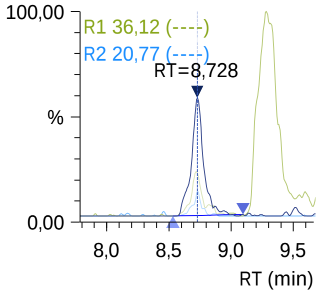

220ppb002
Conc 230,7311
Area 154502
R#1 151,40>92,05 8,13 (17,18)
R#2 151,40>108,10 25,53 (10,23)
Q 151,40>136,05 (-)   1,99e4

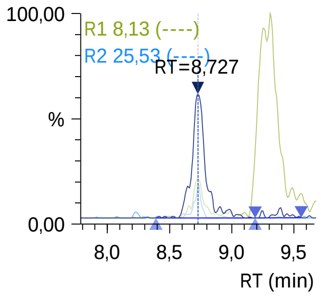

250 ppb001
Conc 259,3387
Area 164366
R#1 151,40>92,05 27,90 (17,18)
R#2 151,40>108,10 13,65 (10,23)
Q 151,40>136,05 (-)   2,61e4

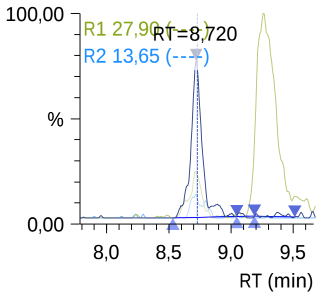

250 ppb002
Conc 220,7648
Area 151066
R#1 151,40>92,05 38,58 (17,18)
R#2 151,40>108,10 16,55 (10,23)
Q 151,40>136,05 (-)   2,65e4

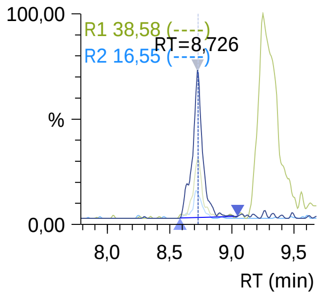

10H
Conc 48,3630
Area 91624
R#1 151,40>92,05 48,63 (17,18)
R#2 151,40>108,10 13,23 (10,23)
Q 151,40>136,05 (-)   8,68e3

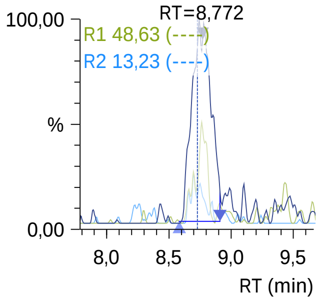

10M
Conc 26,4083
Area 84055
R#1 151,40>92,05 30,49 (17,18)
R#2 151,40>108,10 10,63 (10,23)
Q 151,40>136,05 (-)   9,69e3

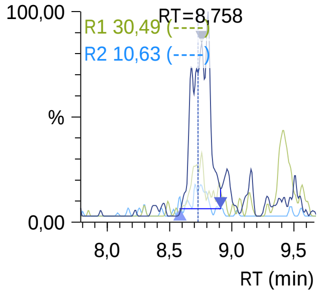

9H
Conc 31,0903
Area 85669
R#1 151,40>92,05 31,05 (17,18)
R#2 151,40>108,10 26,49 (10,23)
Q 151,40>136,05 (-)   7,47e3

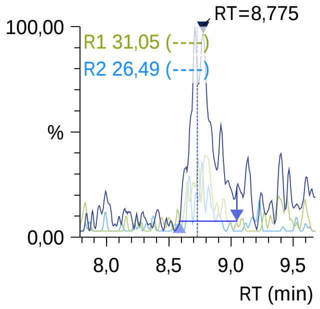

9M
Conc 28,9197
Area 84921
R#1 151,40>92,05 54,12 (17,18)
R#2 151,40>108,10 21,93 (10,23)
Q 151,40>136,05 (-)   7,90e3

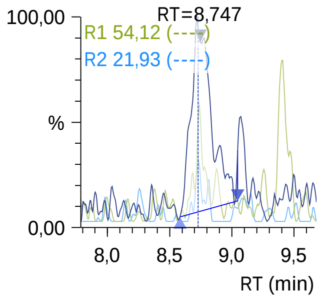

Compound: Ac. Ferulico

Curve Fit: Linear | Weighting: None | Zero: Not Forced
Quantitative Method: External Standard
Q 193,20>133,95
R^2 = 0.9920584 R = 0.9960213
y = 737,7287x - 7004,621

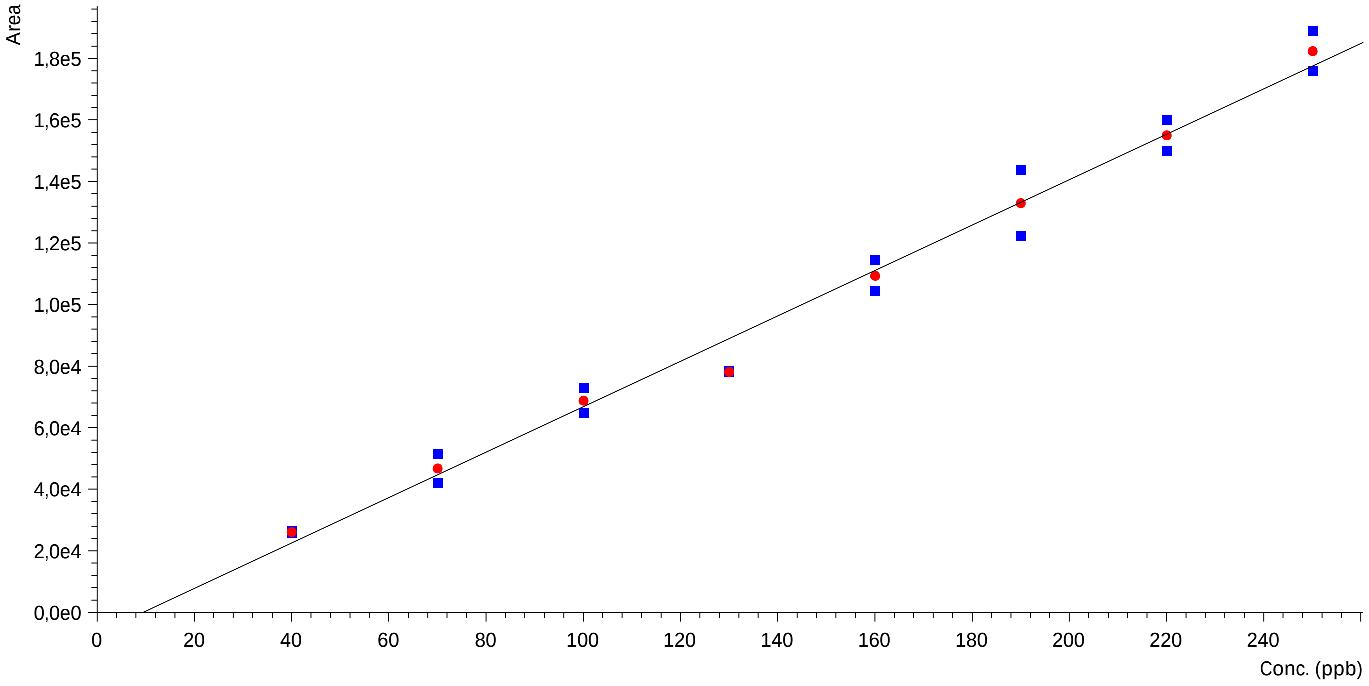

Sample ID	Area	Conc.	Sample Type	Level	Cal Point	Found RT	Ref 1 Actual Ratio
0	----	----	Ctrl.	0	----	----	----
10ppb001	----	----	Std.	1	No	----	----
10 ppb002	----	----	Std.	1	No	----	----
40 ppb001	25677	44,3005	Std.	2	Yes	8,904	45,02
40ppb002	26483	45,3932	Std.	2	Yes	8,925	47,54
70 ppb001	42052	66,4973	Std.	3	Yes	8,929	84,73
70ppb002	51523	79,3354	Std.	3	Yes	8,923	35,00
100 ppb001	64691	97,1837	Std.	4	Yes	8,912	27,86
100ppb002	73037	108,4978	Std.	4	Yes	8,911	40,66
130 ppb001	77993	115,2150	Std.	5	Yes	8,921	49,24
130ppb002	78315	115,6518	Std.	5	Yes	8,923	43,72
160 ppb001	114538	164,7522	Std.	6	Yes	8,921	40,61
160ppb002	104334	150,9209	Std.	6	Yes	8,914	37,47
190 ppb001	122162	175,0875	Std.	7	Yes	8,930	36,50
190ppb002	143787	204,4001	Std.	7	Yes	8,917	40,98
220 ppb001	160038	226,4283	Std.	8	Yes	8,912	55,95
220ppb002	150021	212,8505	Std.	8	Yes	8,921	44,38
250 ppb001	188906	265,5587	Std.	9	Yes	8,918	38,49
250 ppb002	175898	247,9272	Std.	9	Yes	8,914	45,63
10H	100594	145,8512	Unk.	----	----	8,955	70,33
10M	103456	149,7310	Unk.	----	----	9,025	25,05
9H	888955	1214,4843	Unk.	----	----	8,937	46,09
9M	853855	1166,9055	Unk.	----	----	8,939	56,83
Average	177316	249,8486				8,928	45,60
%RSD	137	131,3204				0,287	29,84
Maximum	888955	1214,4843				9,025	84,73
Minimum	25677	44,3005				8,904	25,05
Std. Dev.	242050	328,1023				0,026	13,61

Compound: Ac. Ferulico (continued)

0
Conc ----
Area ----
R#1 193,20>178,05 ---- (36,50)
R#2 193,20>149,05 ---- (12,96)
Q 193,20>133,95 (-) 3,33e2

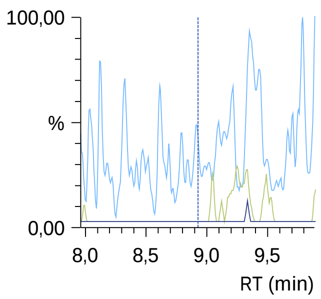

10ppb001
Conc ----
Area ----
R#1 193,20>178,05 ---- (36,50)
R#2 193,20>149,05 ---- (12,96)
Q 193,20>133,95 (-) 1,27e3

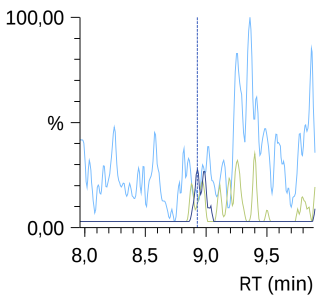

10 ppb002
Conc ----
Area ----
R#1 193,20>178,05 ---- (36,50)
R#2 193,20>149,05 ---- (12,96)
Q 193,20>133,95 (-) 1,33e3

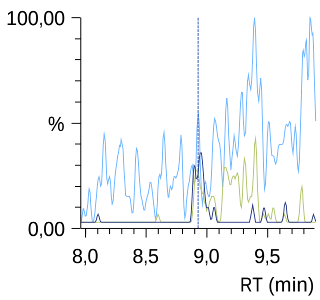

40 ppb001
Conc 44,3005
Area 25677
R#1 193,20>178,05 45,02 (36,50)
R#2 193,20>149,05 0,00 (12,96)
Q 193,20>133,95 (-) 6,25e3

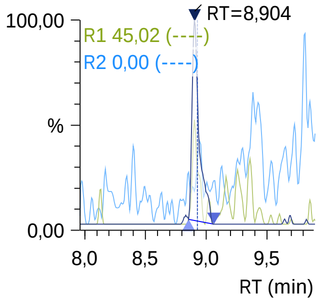

40ppb002
Conc 45,3932
Area 26483
R#1 193,20>178,05 47,54 (36,50)
R#2 193,20>149,05 12,80 (12,96)
Q 193,20>133,95 (-) 5,27e3

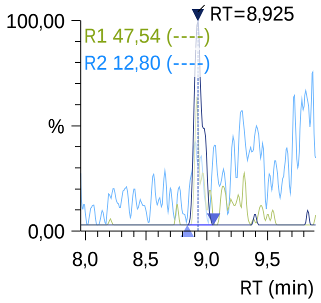

70 ppb001
Conc 66,4973
Area 42052
R#1 193,20>178,05 84,73 (36,50)
R#2 193,20>149,05 28,14 (12,96)
Q 193,20>133,95 (-) 8,00e3

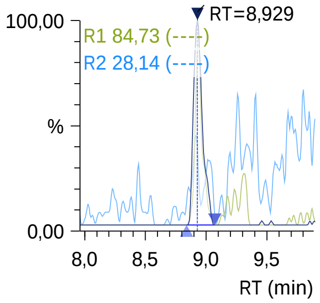

70ppb002
Conc 79,3354
Area 51523
R#1 193,20>178,05 35,00 (36,50)
R#2 193,20>149,05 9,64 (12,96)
Q 193,20>133,95 (-) 1,20e4

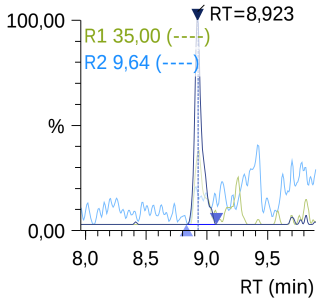

100 ppb001
Conc 97,1837
Area 64691
R#1 193,20>178,05 27,86 (36,50)
R#2 193,20>149,05 12,08 (12,96)
Q 193,20>133,95 (-) 1,56e4

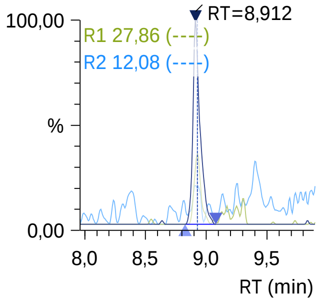

100ppb002
Conc 108,4978
Area 73037
R#1 193,20>178,05 40,66 (36,50)
R#2 193,20>149,05 19,49 (12,96)
Q 193,20>133,95 (-) 1,33e4

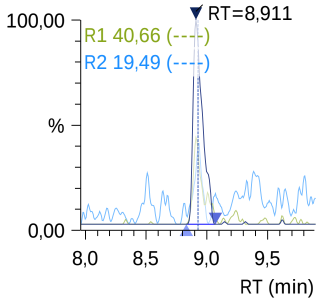

130 ppb001
Conc 115,2150
Area 77993
R#1 193,20>178,05 49,24 (36,50)
R#2 193,20>149,05 15,49 (12,96)
Q 193,20>133,95 (-) 1,82e4

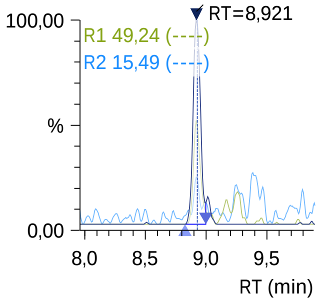

130ppb002
Conc 115,6518
Area 78315
R#1 193,20>178,05 43,72 (36,50)
R#2 193,20>149,05 13,15 (12,96)
Q 193,20>133,95 (-) 1,61e4

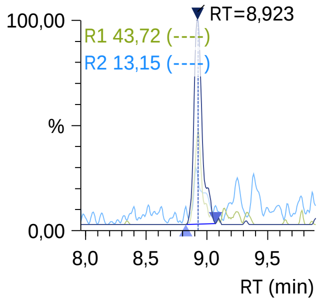

160 ppb001
Conc 164,7522
Area 114538
R#1 193,20>178,05 40,61 (36,50)
R#2 193,20>149,05 17,81 (12,96)
Q 193,20>133,95 (-) 2,63e4

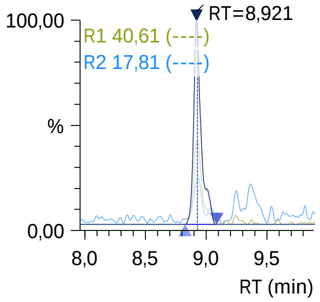

Compound: Ac. Ferulico (continued)

160ppb002
Conc 150,9209
Area 104334
R#1 193,20>178,05 37,47 (36,50)
R#2 193,20>149,05 12,16 (12,96)
Q 193,20>133,95 (-) 2,30e4

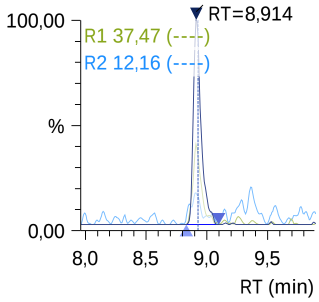

190 ppb001
Conc 175,0875
Area 122162
R#1 193,20>178,05 36,50 (36,50)
R#2 193,20>149,05 12,96 (12,96)
Q 193,20>133,95 (-) 2,74e4

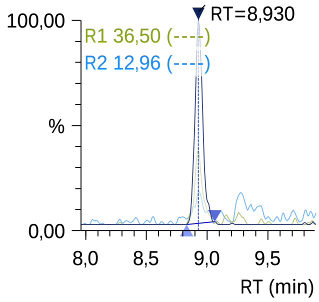

190ppb002
Conc 204,4001
Area 143787
R#1 193,20>178,05 40,98 (36,50)
R#2 193,20>149,05 15,70 (12,96)
Q 193,20>133,95 (-) 3,30e4

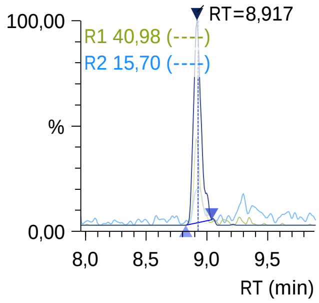

220 ppb001
Conc 226,4283
Area 160038
R#1 193,20>178,05 55,95 (36,50)
R#2 193,20>149,05 17,07 (12,96)
Q 193,20>133,95 (-) 3,04e4

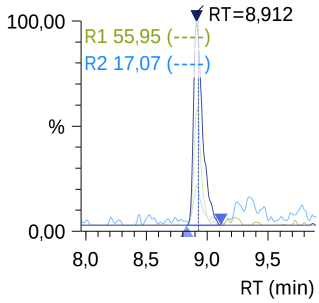

220ppb002
Conc 212,8505
Area 150021
R#1 193,20>178,05 44,38 (36,50)
R#2 193,20>149,05 13,56 (12,96)
Q 193,20>133,95 (-) 3,21e4

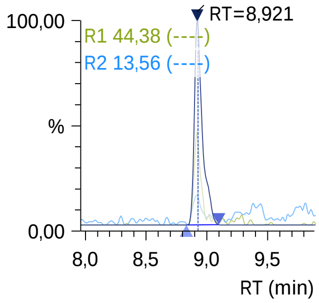

250 ppb001
Conc 265,5587
Area 188906
R#1 193,20>178,05 38,49 (36,50)
R#2 193,20>149,05 13,89 (12,96)
Q 193,20>133,95 (-) 4,07e4

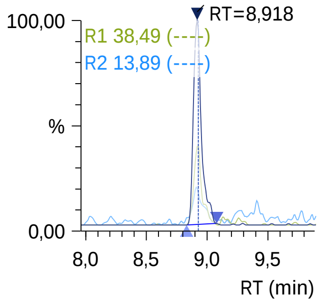

250 ppb002
Conc 247,9272
Area 175898
R#1 193,20>178,05 45,63 (36,50)
R#2 193,20>149,05 18,74 (12,96)
Q 193,20>133,95 (-) 3,71e4

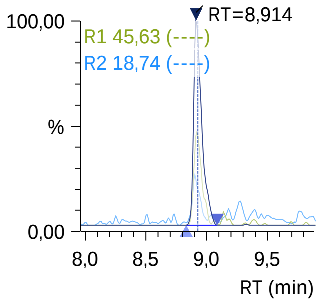

10H
Conc 145,8512
Area 100594
R#1 193,20>178,05 70,33 (36,50)
R#2 193,20>149,05 9,59 (12,96)
Q 193,20>133,95 (-) 1,44e4

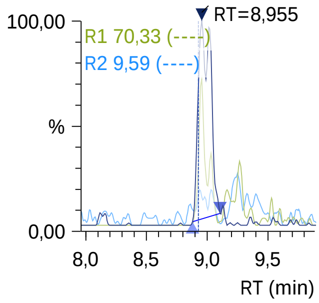

10M
Conc 149,7310
Area 103456
R#1 193,20>178,05 25,05 (36,50)
R#2 193,20>149,05 2,63 (12,96)
Q 193,20>133,95 (-) 1,18e4

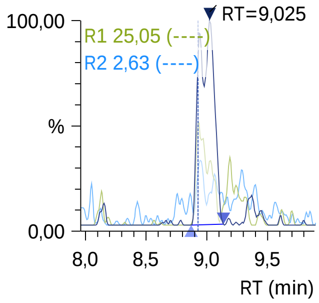

9H
Conc 1214,4843
Area 888955
R#1 193,20>178,05 46,09 (36,50)
R#2 193,20>149,05 17,90 (12,96)
Q 193,20>133,95 (-) 1,50e5

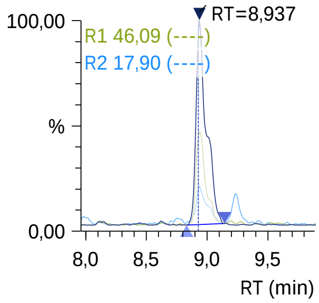

9M
Conc 1166,9055
Area 853855
R#1 193,20>178,05 56,83 (36,50)
R#2 193,20>149,05 18,18 (12,96)
Q 193,20>133,95 (-) 1,06e5

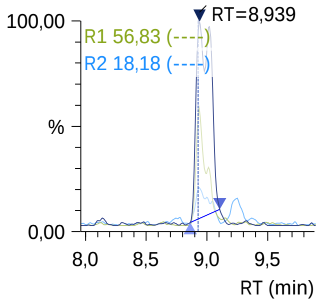

Compound: Ac. p-hiroxibenzoico

Curve Fit: Linear | Weighting: None | Zero: Not Forced
Quantitative Method: External Standard
Q 137,20>92,90
R^2 = 0.9989886 R = 0.9994942
y = 1903,034x + 1133,031

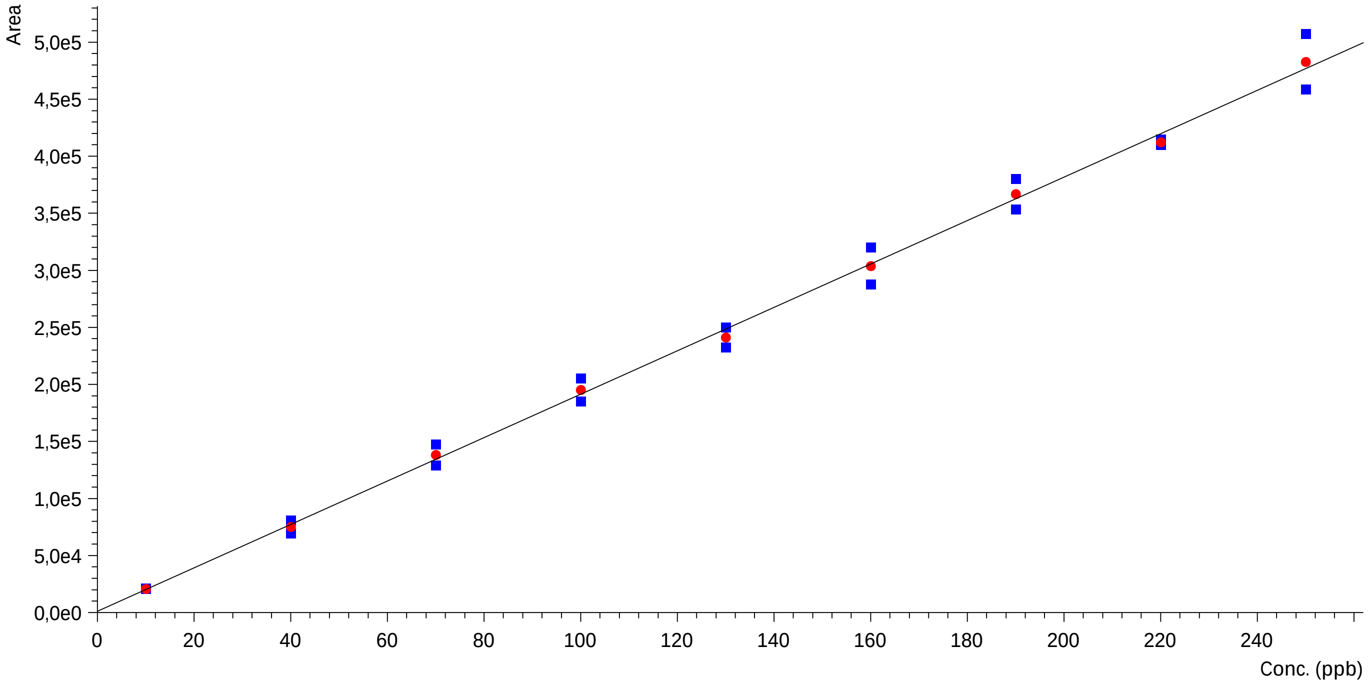

Sample ID	Area	Conc.	Sample Type	Level	Cal Point	Found RT	Ref 1 Actual Ratio
0	15757	7,6846	Ctrl.	0	----	8,313	0,00
10ppb001	20711	10,2876	Std.	1	Yes	8,234	1,57
10 ppb002	20959	10,4183	Std.	1	Yes	8,249	7,27
40 ppb001	69663	36,0107	Std.	2	Yes	8,238	5,77
40ppb002	80728	41,8252	Std.	2	Yes	8,246	8,85
70 ppb001	147606	76,9679	Std.	3	Yes	8,248	4,65
70ppb002	128924	67,1510	Std.	3	Yes	8,251	6,07
100 ppb001	185169	96,7066	Std.	4	Yes	8,246	4,30
100ppb002	205246	107,2569	Std.	4	Yes	8,251	3,94
130 ppb001	250164	130,8602	Std.	5	Yes	8,247	7,77
130ppb002	232549	121,6040	Std.	5	Yes	8,254	6,33
160 ppb001	287593	150,5280	Std.	6	Yes	8,253	7,27
160ppb002	319991	167,5525	Std.	6	Yes	8,248	5,15
190 ppb001	380175	199,1776	Std.	7	Yes	8,257	8,05
190ppb002	353536	185,1798	Std.	7	Yes	8,245	7,87
220 ppb001	409744	214,7153	Std.	8	Yes	8,249	5,87
220ppb002	414936	217,4436	Std.	8	Yes	8,254	5,84
250 ppb001	507267	265,9615	Std.	9	Yes	8,246	6,39
250 ppb002	458534	240,3535	Std.	9	Yes	8,250	7,63
10H	100713	52,3269	Unk.	----	----	8,280	2,22
10M	100425	52,1755	Unk.	----	----	8,269	1,71
9H	510757	267,7958	Unk.	----	----	8,264	5,49
9M	345556	180,9861	Unk.	----	----	8,259	5,04
Average	241161	126,1291				8,254	5,44
%RSD	67	67,0067				0,195	42,45
Maximum	510757	267,7958				8,313	8,85
Minimum	15757	7,6846				8,234	0,00
Std. Dev.	160835	84,5149				0,016	2,31

Compound: Ac. p-hiroxibenzoico (continued)

0
Conc 7,6846
Area 15757
R#1 137,20>64,95 0,00 (8,05)
R#2 137,20>74,90 0,00 (1,51)
Q 137,20>92,90 (-) 5,13e3

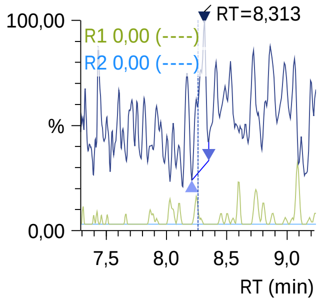

10ppb001
Conc 10,2876
Area 20711
R#1 137,20>64,95 1,57 (8,05)
R#2 137,20>74,90 0,00 (1,51)
Q 137,20>92,90 (-) 8,09e3

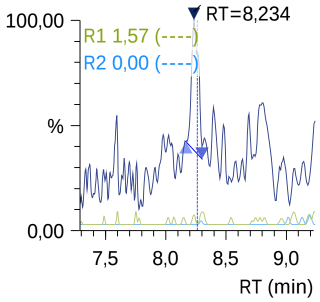

10 ppb002
Conc 10,4183
Area 20959
R#1 137,20>64,95 7,27 (8,05)
R#2 137,20>74,90 0,00 (1,51)
Q 137,20>92,90 (-) 1,02e4

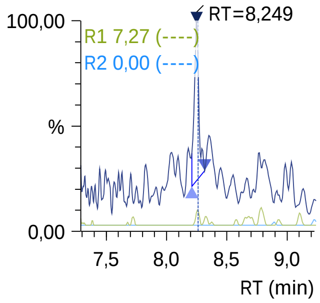

40 ppb001
Conc 36,0107
Area 69663
R#1 137,20>64,95 5,77 (8,05)
R#2 137,20>74,90 0,95 (1,51)
Q 137,20>92,90 (-) 2,08e4

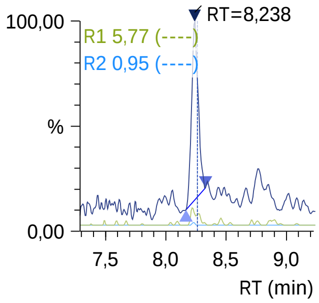

40ppb002
Conc 41,8252
Area 80728
R#1 137,20>64,95 8,85 (8,05)
R#2 137,20>74,90 0,00 (1,51)
Q 137,20>92,90 (-) 2,07e4

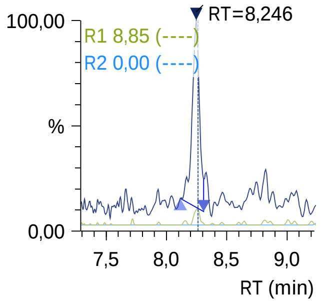

70 ppb001
Conc 76,9679
Area 147606
R#1 137,20>64,95 4,65 (8,05)
R#2 137,20>74,90 0,00 (1,51)
Q 137,20>92,90 (-) 3,61e4

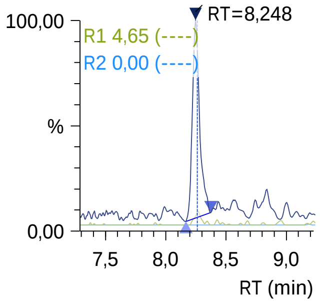

70ppb002
Conc 67,1510
Area 128924
R#1 137,20>64,95 6,07 (8,05)
R#2 137,20>74,90 0,47 (1,51)
Q 137,20>92,90 (-) 3,15e4

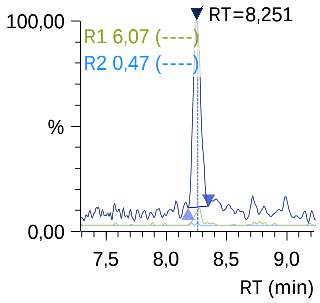

100 ppb001
Conc 96,7066
Area 185169
R#1 137,20>64,95 4,30 (8,05)
R#2 137,20>74,90 0,04 (1,51)
Q 137,20>92,90 (-) 5,03e4

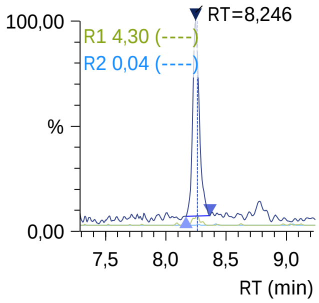

100ppb002
Conc 107,2569
Area 205246
R#1 137,20>64,95 3,94 (8,05)
R#2 137,20>74,90 0,14 (1,51)
Q 137,20>92,90 (-) 5,65e4

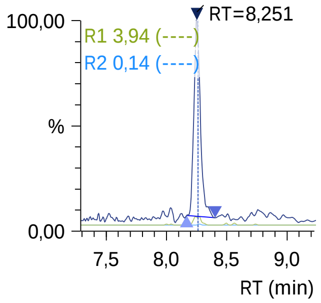

130 ppb001
Conc 130,8602
Area 250164
R#1 137,20>64,95 7,77 (8,05)
R#2 137,20>74,90 0,48 (1,51)
Q 137,20>92,90 (-) 6,52e4

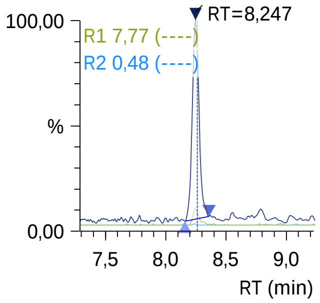

130ppb002
Conc 121,6040
Area 232549
R#1 137,20>64,95 6,33 (8,05)
R#2 137,20>74,90 1,45 (1,51)
Q 137,20>92,90 (-) 5,92e4

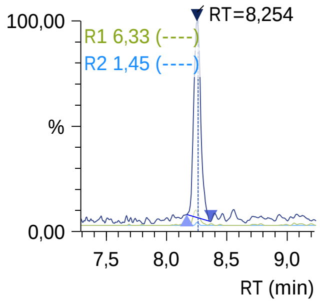

160 ppb001
Conc 150,5280
Area 287593
R#1 137,20>64,95 7,27 (8,05)
R#2 137,20>74,90 0,95 (1,51)
Q 137,20>92,90 (-) 7,63e4

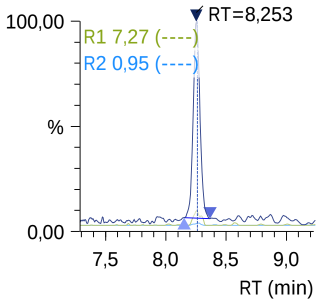

Compound: Ac. p-hiroxibenzoico (continued)

160ppb002
Conc 167,5525
Area 319991
R#1 137,20>64,95 5,15 (8,05)
R#2 137,20>74,90 0,14 (1,51)
Q 137,20>92,90 (-) 8,57e4

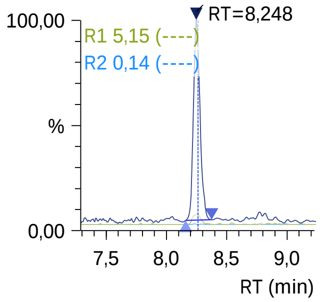

190 ppb001
Conc 199,1776
Area 380175
R#1 137,20>64,95 8,05 (8,05)
R#2 137,20>74,90 1,51 (1,51)
Q 137,20>92,90 (-) 9,99e4

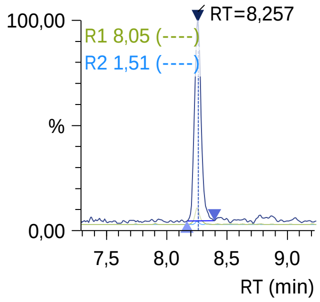

190ppb002
Conc 185,1798
Area 353536
R#1 137,20>64,95 7,87 (8,05)
R#2 137,20>74,90 0,40 (1,51)
Q 137,20>92,90 (-) 9,27e4

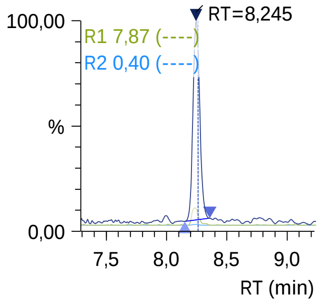

220 ppb001
Conc 214,7153
Area 409744
R#1 137,20>64,95 5,87 (8,05)
R#2 137,20>74,90 0,63 (1,51)
Q 137,20>92,90 (-) 1,11e5

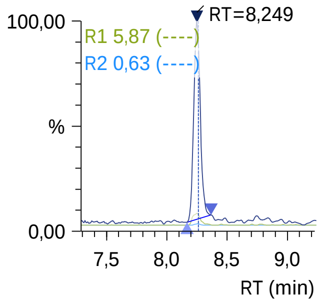

220ppb002
Conc 217,4436
Area 414936
R#1 137,20>64,95 5,84 (8,05)
R#2 137,20>74,90 0,46 (1,51)
Q 137,20>92,90 (-) 1,09e5

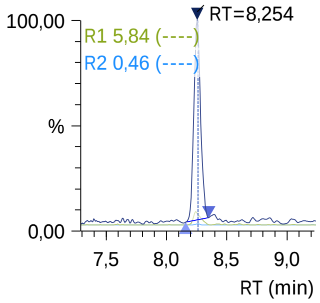

250 ppb001
Conc 265,9615
Area 507267
R#1 137,20>64,95 6,39 (8,05)
R#2 137,20>74,90 0,41 (1,51)
Q 137,20>92,90 (-) 1,28e5

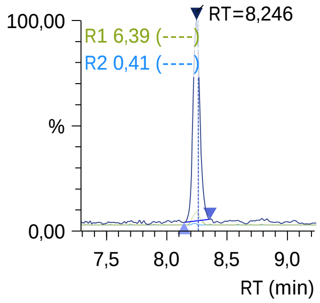

250 ppb002
Conc 240,3535
Area 458534
R#1 137,20>64,95 7,63 (8,05)
R#2 137,20>74,90 0,17 (1,51)
Q 137,20>92,90 (-) 1,16e5

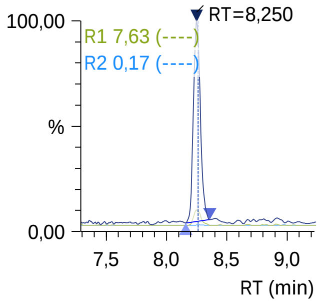

10H
Conc 52,3269
Area 100713
R#1 137,20>64,95 2,22 (8,05)
R#2 137,20>74,90 0,00 (1,51)
Q 137,20>92,90 (-) 2,64e4

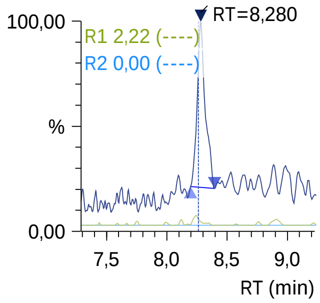

10M
Conc 52,1755
Area 100425
R#1 137,20>64,95 1,71 (8,05)
R#2 137,20>74,90 0,00 (1,51)
Q 137,20>92,90 (-) 2,57e4

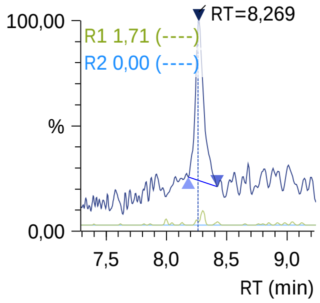

9H
Conc 267,7958
Area 510757
R#1 137,20>64,95 5,49 (8,05)
R#2 137,20>74,90 0,88 (1,51)
Q 137,20>92,90 (-) 1,24e5

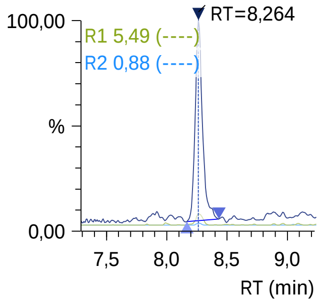

9M
Conc 180,9861
Area 345556
R#1 137,20>64,95 5,04 (8,05)
R#2 137,20>74,90 0,73 (1,51)
Q 137,20>92,90 (-) 8,70e4

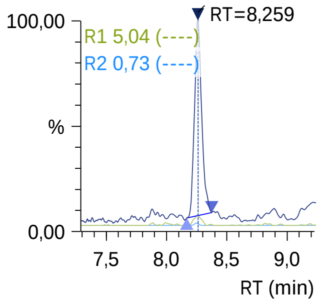

Compound: Naringina

Curve Fit: Linear | Weighting: None | Zero: Not Forced
Quantitative Method: External Standard
Q 579,00>271,15
R^2 = 0.9967188 R = 0.9983580
y = 1955,294x - 316,6205

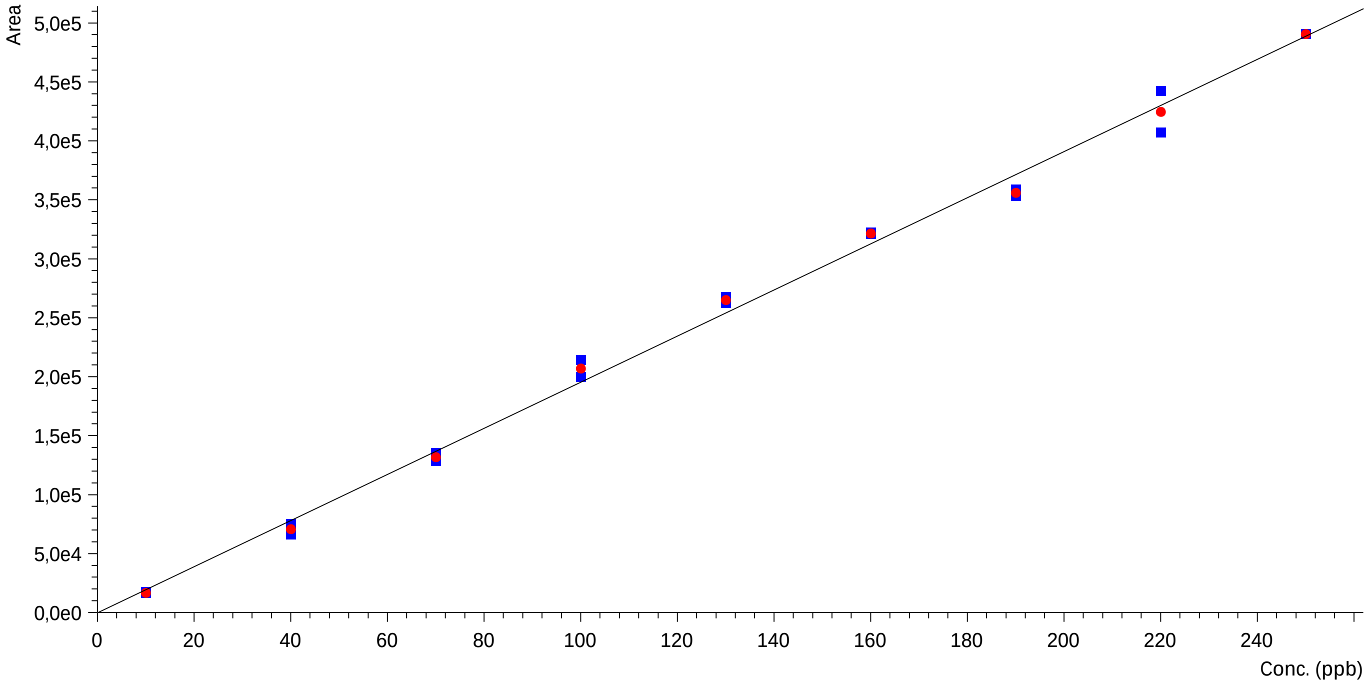

Sample ID	Area	Conc.	Sample Type	Level	Cal Point	Found RT	Ref 1 Actual Ratio
0	----	----	Ctrl.	0	----	----	----
10ppb001	17321	9,0203	Std.	1	Yes	8,795	144,59
10 ppb002	16525	8,6132	Std.	1	Yes	8,812	61,50
40 ppb001	75094	38,5674	Std.	2	Yes	8,805	76,54
40ppb002	66476	34,1600	Std.	2	Yes	8,810	93,74
70 ppb001	135336	69,3771	Std.	3	Yes	8,811	77,94
70ppb002	128677	65,9714	Std.	3	Yes	8,809	95,49
100 ppb001	214127	109,6732	Std.	4	Yes	8,799	76,19
100ppb002	199760	102,3258	Std.	4	Yes	8,810	81,37
130 ppb001	262495	134,4102	Std.	5	Yes	8,806	74,55
130ppb002	267544	136,9926	Std.	5	Yes	8,807	78,39
160 ppb001	321146	164,4063	Std.	6	Yes	8,811	98,81
160ppb002	322263	164,9776	Std.	6	Yes	8,801	82,49
190 ppb001	358867	183,6981	Std.	7	Yes	8,816	95,41
190ppb002	353355	180,8788	Std.	7	Yes	8,799	85,80
220 ppb001	442664	226,5545	Std.	8	Yes	8,803	86,20
220ppb002	406937	208,2827	Std.	8	Yes	8,806	100,49
250 ppb001	490546	251,0431	Std.	9	Yes	8,795	82,13
250 ppb002	490555	251,0477	Std.	9	Yes	8,804	78,63
10H	----	----	Unk.	----	----	----	----
10M	----	----	Unk.	----	----	----	----
9H	----	----	Unk.	----	----	----	----
9M	----	----	Unk.	----	----	----	----
Average	253872	130,0000				8,805	87,24
%RSD	62	61,4741				0,067	19,97
Maximum	490555	251,0477				8,816	144,59
Minimum	16525	8,6132				8,795	61,50
Std. Dev.	156260	79,9163				0,006	17,42

Compound: Naringina (continued)

0
Conc ----
Area ----
R#1 579,00>151,00 ---- (95,41)
R#2 579,00>459,10 ---- (44,29)
Q 579,00>271,15 (-) 2,87e2

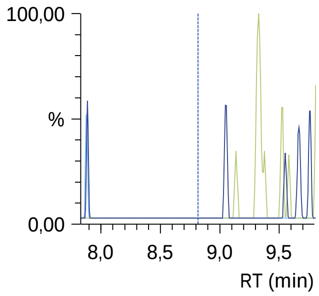

10ppb001
Conc 9,0203
Area 17321
R#1 579,00>151,00 144,59 (95,41)
R#2 579,00>459,10 37,13 (44,29)
Q 579,00>271,15 (-) 3,89e3

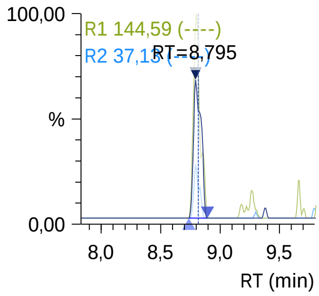

10 ppb002
Conc 8,6132
Area 16525
R#1 579,00>151,00 61,50 (95,41)
R#2 579,00>459,10 15,99 (44,29)
Q 579,00>271,15 (-) 4,79e3

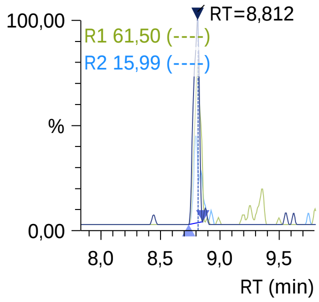

40 ppb001
Conc 38,5674
Area 75094
R#1 579,00>151,00 76,54 (95,41)
R#2 579,00>459,10 35,85 (44,29)
Q 579,00>271,15 (-) 1,88e4

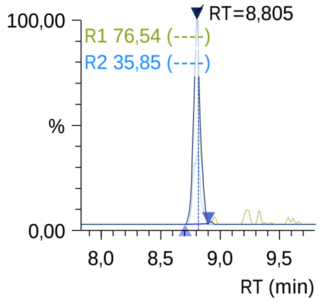

40ppb002
Conc 34,1600
Area 66476
R#1 579,00>151,00 93,74 (95,41)
R#2 579,00>459,10 38,83 (44,29)
Q 579,00>271,15 (-) 1,40e4

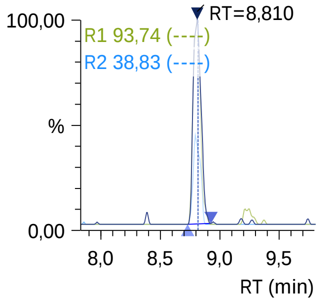

70 ppb001
Conc 69,3771
Area 135336
R#1 579,00>151,00 77,94 (95,41)
R#2 579,00>459,10 50,14 (44,29)
Q 579,00>271,15 (-) 3,22e4

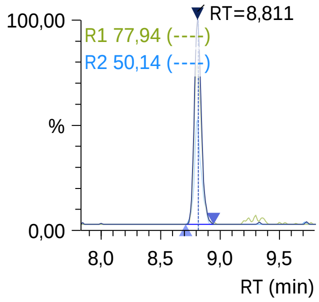

70ppb002
Conc 65,9714
Area 128677
R#1 579,00>151,00 95,49 (95,41)
R#2 579,00>459,10 48,02 (44,29)
Q 579,00>271,15 (-) 2,82e4

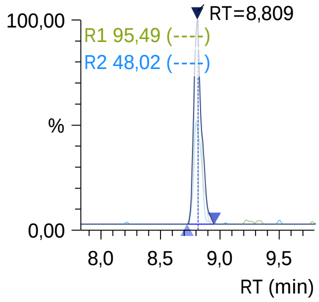

100 ppb001
Conc 109,6732
Area 214127
R#1 579,00>151,00 76,19 (95,41)
R#2 579,00>459,10 44,67 (44,29)
Q 579,00>271,15 (-) 5,32e4

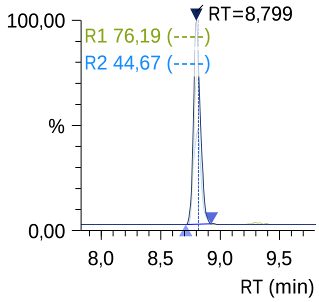

100ppb002
Conc 102,3258
Area 199760
R#1 579,00>151,00 81,37 (95,41)
R#2 579,00>459,10 43,09 (44,29)
Q 579,00>271,15 (-) 4,72e4

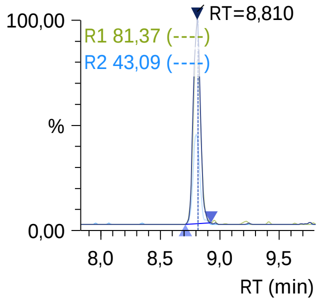

130 ppb001
Conc 134,4102
Area 262495
R#1 579,00>151,00 74,55 (95,41)
R#2 579,00>459,10 49,07 (44,29)
Q 579,00>271,15 (-) 6,42e4

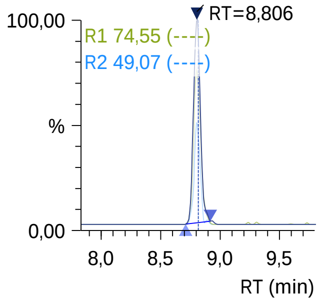

130ppb002
Conc 136,9926
Area 267544
R#1 579,00>151,00 78,39 (95,41)
R#2 579,00>459,10 46,06 (44,29)
Q 579,00>271,15 (-) 6,12e4

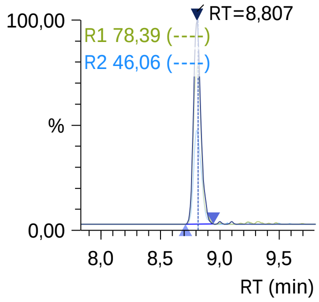

160 ppb001
Conc 164,4063
Area 321146
R#1 579,00>151,00 98,81 (95,41)
R#2 579,00>459,10 50,66 (44,29)
Q 579,00>271,15 (-) 7,07e4

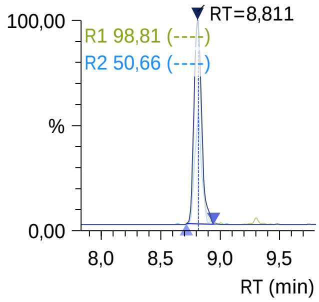

Compound: Naringina (continued)

160ppb002
Conc 164,9776
Area 322263
R#1 579,00>151,00 82,49 (95,41)
R#2 579,00>459,10 51,15 (44,29)
Q 579,00>271,15 (-) 8,48e4

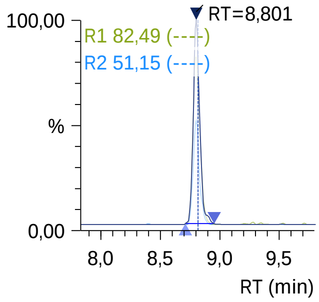

190 ppb001
Conc 183,6981
Area 358867
R#1 579,00>151,00 95,41 (95,41)
R#2 579,00>459,10 44,29 (44,29)
Q 579,00>271,15 (-) 7,89e4

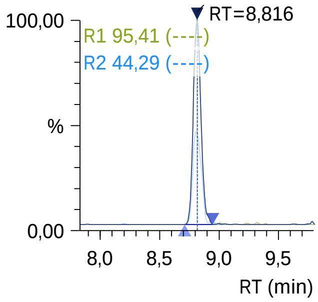

190ppb002
Conc 180,8788
Area 353355
R#1 579,00>151,00 85,80 (95,41)
R#2 579,00>459,10 43,94 (44,29)
Q 579,00>271,15 (-) 8,75e4

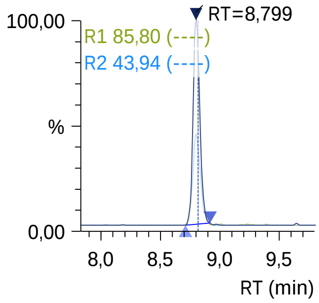

220 ppb001
Conc 226,5545
Area 442664
R#1 579,00>151,00 86,20 (95,41)
R#2 579,00>459,10 48,26 (44,29)
Q 579,00>271,15 (-) 9,77e4

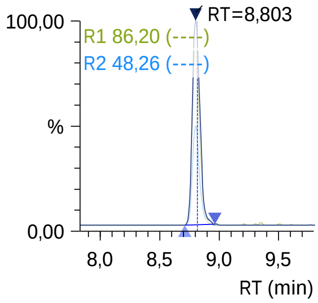

220ppb002
Conc 208,2827
Area 406937
R#1 579,00>151,00 100,49 (95,41)
R#2 579,00>459,10 51,74 (44,29)
Q 579,00>271,15 (-) 8,56e4

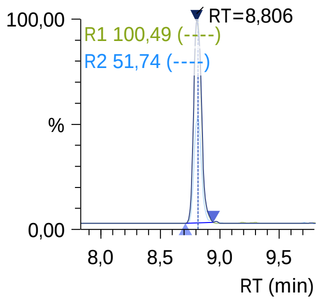

250 ppb001
Conc 251,0431
Area 490546
R#1 579,00>151,00 82,13 (95,41)
R#2 579,00>459,10 44,52 (44,29)
Q 579,00>271,15 (-) 1,13e5

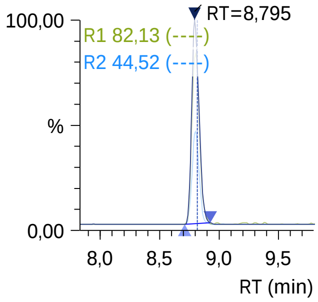

250 ppb002
Conc 251,0477
Area 490555
R#1 579,00>151,00 78,63 (95,41)
R#2 579,00>459,10 39,61 (44,29)
Q 579,00>271,15 (-) 1,19e5

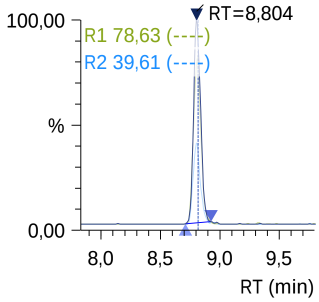

10H
Conc ----
Area ----
R#1 579,00>151,00 ---- (95,41)
R#2 579,00>459,10 ---- (44,29)
Q 579,00>271,15 (-) 1,56e2

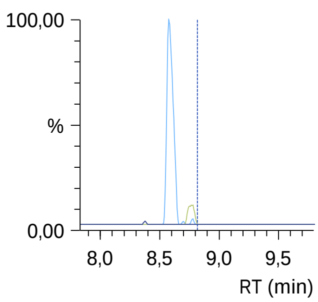

10M
Conc ----
Area ----
R#1 579,00>151,00 ---- (95,41)
R#2 579,00>459,10 ---- (44,29)
Q 579,00>271,15 (-) 3,86e3

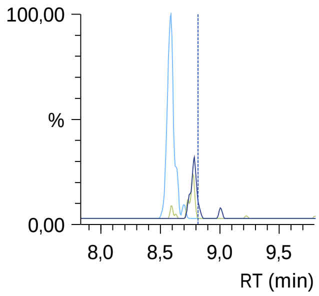

9H
Conc ----
Area ----
R#1 579,00>151,00 ---- (95,41)
R#2 579,00>459,10 ---- (44,29)
Q 579,00>271,15 (-) 6,63e2

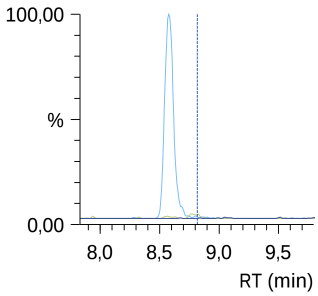

9M
Conc ----
Area ----
R#1 579,00>151,00 ---- (95,41)
R#2 579,00>459,10 ---- (44,29)
Q 579,00>271,15 (-) 8,33e2

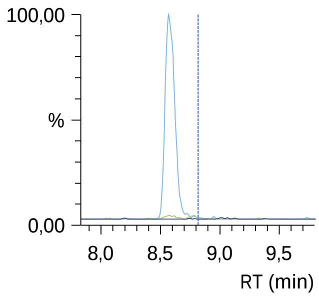

Compound: Ac. p-cumarico

Curve Fit: Linear | Weighting: None | Zero: Not Forced
Quantitative Method: External Standard
Q 163,20>119,00
R^2 = 0.9978567 R = 0.9989278
y = 3426,103x - 14433,06

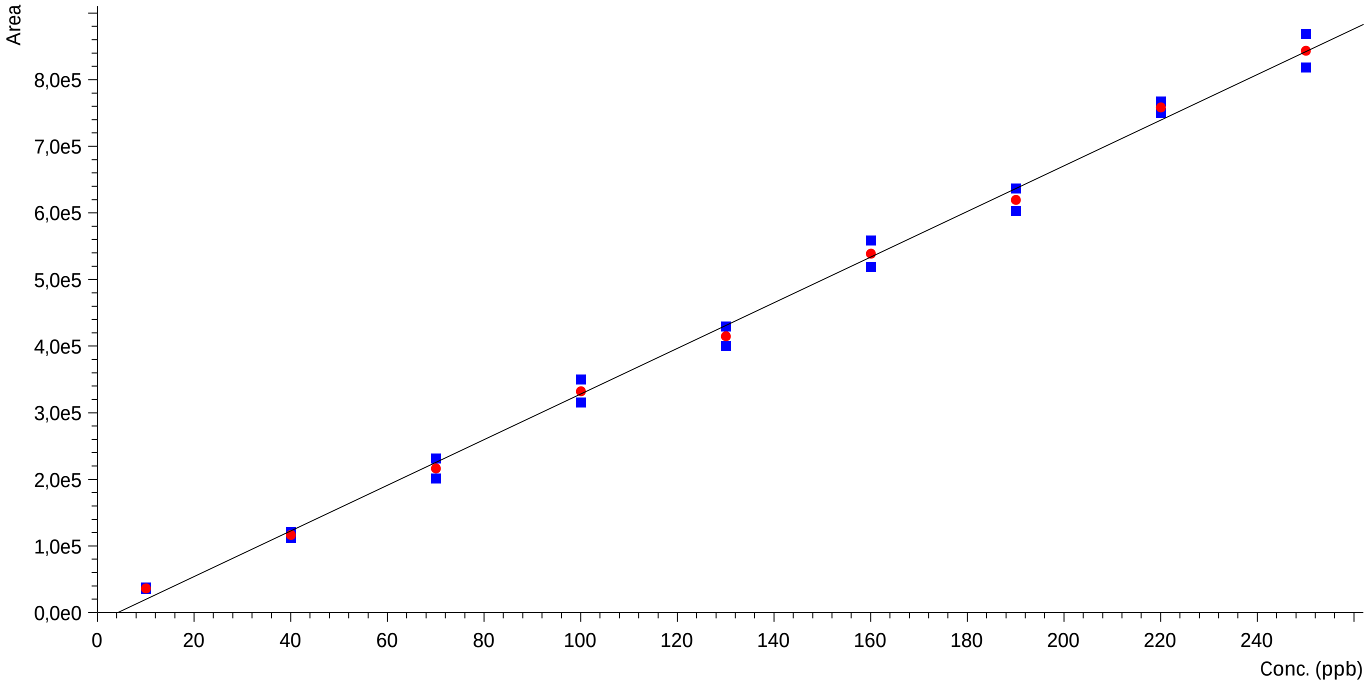

Sample ID	Area	Conc.	Sample Type	Level	Cal Point	Found RT	Ref 1 Actual Ratio
0	----	----	Ctrl.	0	----	----	----
10ppb001	35565	14,5931	Std.	1	Yes	8,855	2,78
10 ppb002	37424	15,1358	Std.	1	Yes	8,857	5,18
40 ppb001	121022	39,5361	Std.	2	Yes	8,844	17,98
40ppb002	112420	37,0253	Std.	2	Yes	8,852	14,53
70 ppb001	201724	63,0914	Std.	3	Yes	8,849	11,56
70ppb002	231667	71,8309	Std.	3	Yes	8,851	12,09
100 ppb001	349687	106,2783	Std.	4	Yes	8,846	11,96
100ppb002	315720	96,3640	Std.	4	Yes	8,847	9,81
130 ppb001	400257	121,0384	Std.	5	Yes	8,845	11,54
130ppb002	429720	129,6381	Std.	5	Yes	8,848	11,79
160 ppb001	518989	155,6935	Std.	6	Yes	8,850	11,52
160ppb002	558908	167,3449	Std.	6	Yes	8,844	10,59
190 ppb001	602812	180,1595	Std.	7	Yes	8,850	13,76
190ppb002	636420	189,9688	Std.	7	Yes	8,842	15,51
220 ppb001	750103	223,1504	Std.	8	Yes	8,848	11,19
220ppb002	767626	228,2647	Std.	8	Yes	8,851	11,64
250 ppb001	818436	243,0950	Std.	9	Yes	8,844	10,72
250 ppb002	868787	257,7916	Std.	9	Yes	8,846	11,06
10H	102628	34,1675	Unk.	----	----	8,877	12,49
10M	151658	48,4782	Unk.	----	----	8,887	15,40
9H	57323	20,9438	Unk.	----	----	8,864	16,29
9M	640787	191,2437	Unk.	----	----	8,865	9,71
Average	395895	119,7651				8,853	11,78
%RSD	70	67,6286				0,126	28,35
Maximum	868787	257,7916				8,887	17,98
Minimum	35565	14,5931				8,842	2,78
Std. Dev.	277499	80,9955				0,011	3,34

Compound: Ac. p-cumarico (continued)

0
Conc ----
Area ----
R#1 163,20>92,95 ---- (13,76)
R#2 163,20>116,85 ---- (2,64)
Q 163,20>119,00 (-) 7,00e3

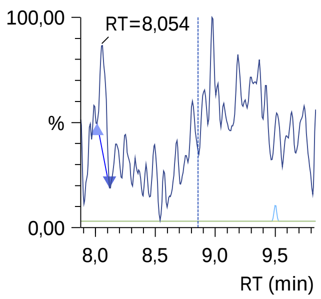

10ppb001
Conc 14,5931
Area 35565
R#1 163,20>92,95 2,78 (13,76)
R#2 163,20>116,85 6,50 (2,64)
Q 163,20>119,00 (-) 1,08e4

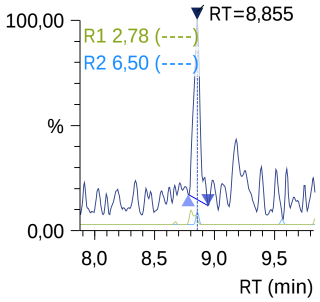

10 ppb002
Conc 15,1358
Area 37424
R#1 163,20>92,95 5,18 (13,76)
R#2 163,20>116,85 0,21 (2,64)
Q 163,20>119,00 (-) 1,09e4

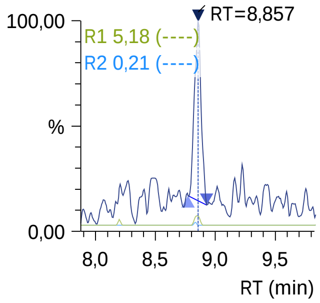

40 ppb001
Conc 39,5361
Area 121022
R#1 163,20>92,95 17,98 (13,76)
R#2 163,20>116,85 1,54 (2,64)
Q 163,20>119,00 (-) 2,88e4

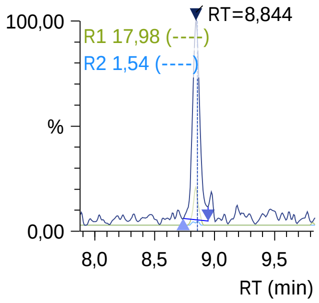

40ppb002
Conc 37,0253
Area 112420
R#1 163,20>92,95 14,53 (13,76)
R#2 163,20>116,85 3,09 (2,64)
Q 163,20>119,00 (-) 2,79e4

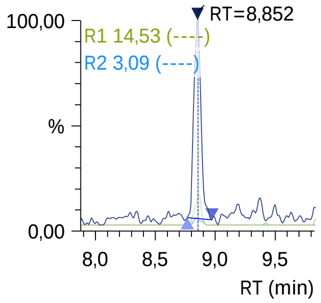

70 ppb001
Conc 63,0914
Area 201724
R#1 163,20>92,95 11,56 (13,76)
R#2 163,20>116,85 2,83 (2,64)
Q 163,20>119,00 (-) 5,62e4

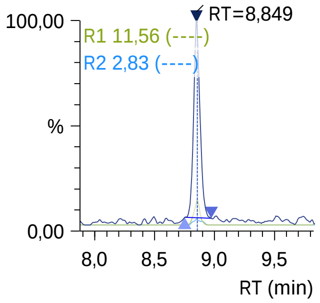

70ppb002
Conc 71,8309
Area 231667
R#1 163,20>92,95 12,09 (13,76)
R#2 163,20>116,85 3,82 (2,64)
Q 163,20>119,00 (-) 5,58e4

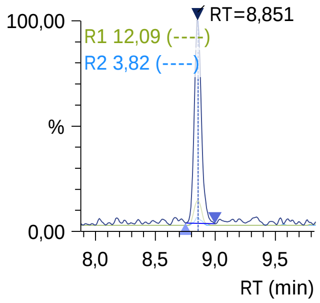

100 ppb001
Conc 106,2783
Area 349687
R#1 163,20>92,95 11,96 (13,76)
R#2 163,20>116,85 5,04 (2,64)
Q 163,20>119,00 (-) 8,96e4

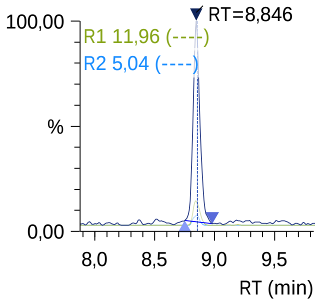

100ppb002
Conc 96,3640
Area 315720
R#1 163,20>92,95 9,81 (13,76)
R#2 163,20>116,85 3,91 (2,64)
Q 163,20>119,00 (-) 8,00e4

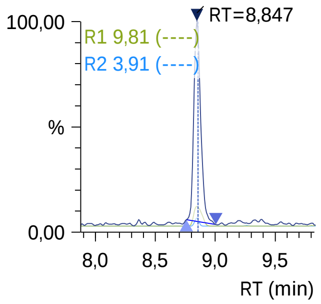

130 ppb001
Conc 121,0384
Area 400257
R#1 163,20>92,95 11,54 (13,76)
R#2 163,20>116,85 4,95 (2,64)
Q 163,20>119,00 (-) 1,03e5

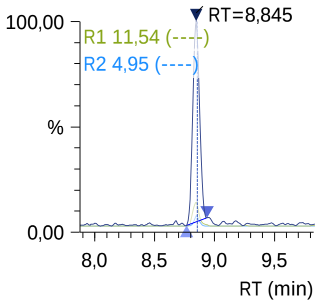

130ppb002
Conc 129,6381
Area 429720
R#1 163,20>92,95 11,79 (13,76)
R#2 163,20>116,85 4,30 (2,64)
Q 163,20>119,00 (-) 1,04e5

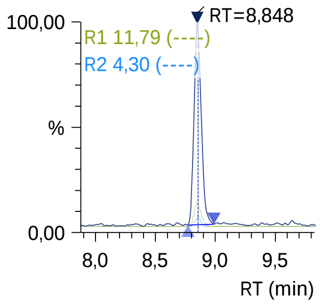

160 ppb001
Conc 155,6935
Area 518989
R#1 163,20>92,95 11,52 (13,76)
R#2 163,20>116,85 4,18 (2,64)
Q 163,20>119,00 (-) 1,24e5

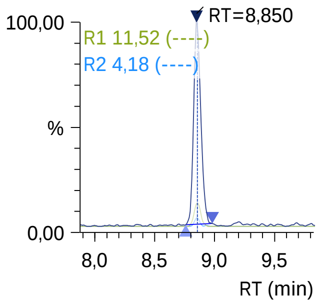

Compound: Ac. p-cumarico (continued)

160ppb002
Conc 167,3449
Area 558908
R#1 163,20>92,95 10,59 (13,76)
R#2 163,20>116,85 2,37 (2,64)
Q 163,20>119,00 (-) 1,33e5

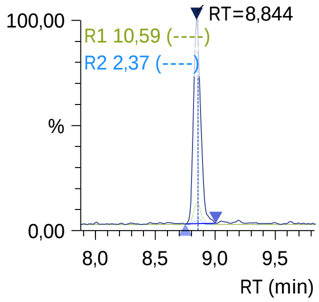

190 ppb001
Conc 180,1595
Area 602812
R#1 163,20>92,95 13,76 (13,76)
R#2 163,20>116,85 2,64 (2,64)
Q 163,20>119,00 (-) 1,53e5

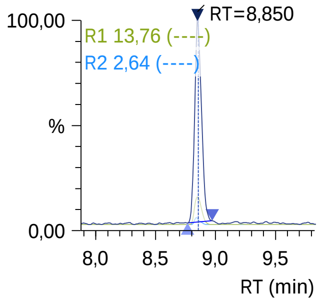

190ppb002
Conc 189,9688
Area 636420
R#1 163,20>92,95 15,51 (13,76)
R#2 163,20>116,85 2,39 (2,64)
Q 163,20>119,00 (-) 1,60e5

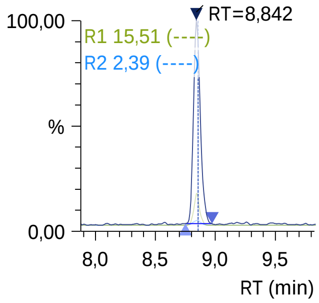

220 ppb001
Conc 223,1504
Area 750103
R#1 163,20>92,95 11,19 (13,76)
R#2 163,20>116,85 3,08 (2,64)
Q 163,20>119,00 (-) 1,85e5

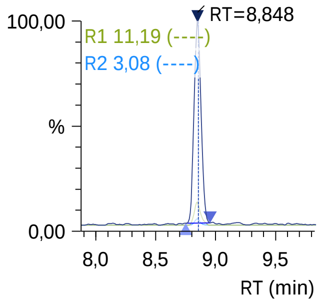

220ppb002
Conc 228,2647
Area 767626
R#1 163,20>92,95 11,64 (13,76)
R#2 163,20>116,85 2,83 (2,64)
Q 163,20>119,00 (-) 1,88e5

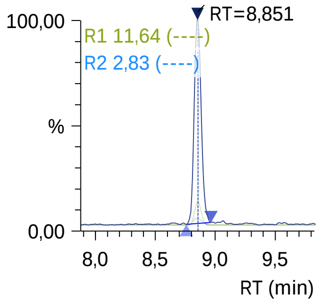

250 ppb001
Conc 243,0950
Area 818436
R#1 163,20>92,95 10,72 (13,76)
R#2 163,20>116,85 3,38 (2,64)
Q 163,20>119,00 (-) 2,11e5

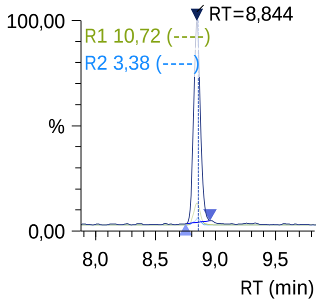

250 ppb002
Conc 257,7916
Area 868787
R#1 163,20>92,95 11,06 (13,76)
R#2 163,20>116,85 3,42 (2,64)
Q 163,20>119,00 (-) 2,25e5

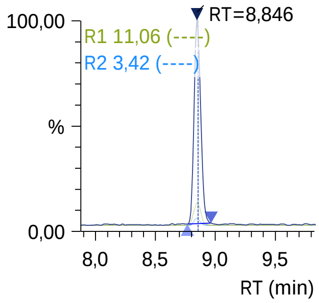

10H
Conc 34,1675
Area 102628
R#1 163,20>92,95 12,49 (13,76)
R#2 163,20>116,85 3,31 (2,64)
Q 163,20>119,00 (-) 2,48e4

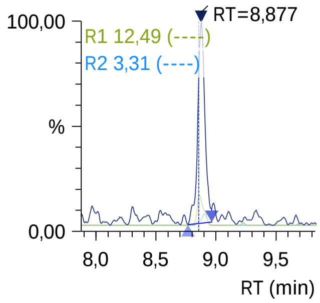

10M
Conc 48,4782
Area 151658
R#1 163,20>92,95 15,40 (13,76)
R#2 163,20>116,85 5,71 (2,64)
Q 163,20>119,00 (-) 3,20e4

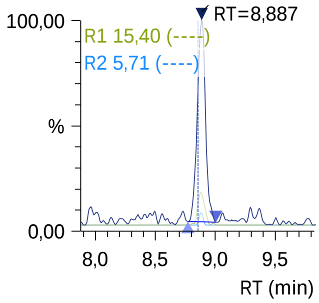

9H
Conc 20,9438
Area 57323
R#1 163,20>92,95 16,29 (13,76)
R#2 163,20>116,85 2,80 (2,64)
Q 163,20>119,00 (-) 1,52e4

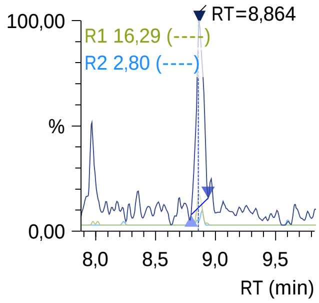

9M
Conc 191,2437
Area 640787
R#1 163,20>92,95 9,71 (13,76)
R#2 163,20>116,85 3,95 (2,64)
Q 163,20>119,00 (-) 1,43e5

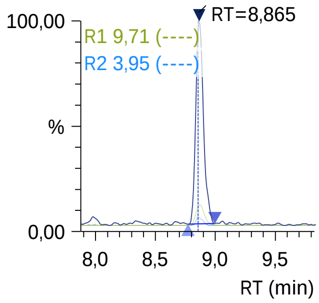

Compound: Ac. Cafeico

Curve Fit: Linear | Weighting: None | Zero: Not Forced
Quantitative Method: External Standard
Q 179,20>135,00
R^2 = 0.9978860 R = 0.9989425
y = 3786,001x - 24911,06

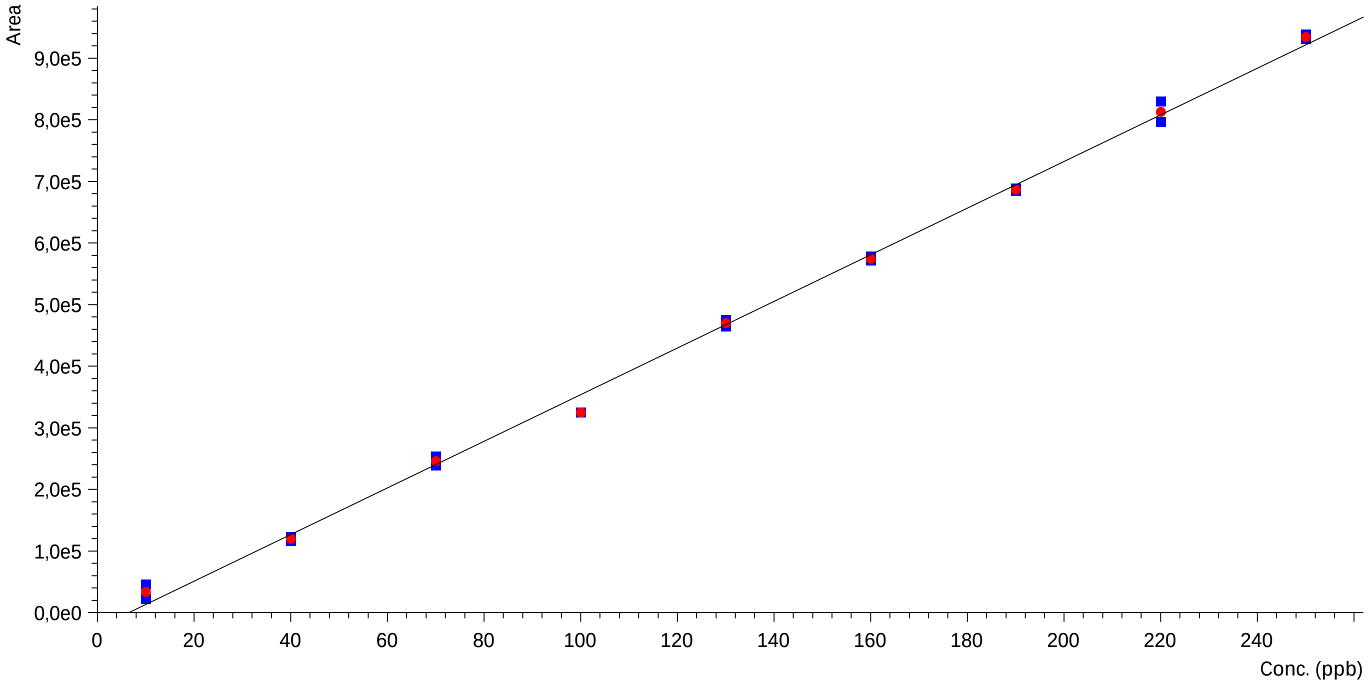

Sample ID	Area	Conc.	Sample Type	Level	Cal Point	Found RT	Ref 1 Actual Ratio
0	12569	9,8998	Ctrl.	0	----	8,480	0,00
10ppb001	45496	18,5967	Std.	1	Yes	8,348	7,65
10 ppb002	22440	12,5069	Std.	1	Yes	8,349	13,78
40 ppb001	123296	39,1461	Std.	2	Yes	8,333	16,41
40ppb002	116298	37,2976	Std.	2	Yes	8,336	17,89
70 ppb001	253517	73,5415	Std.	3	Yes	8,346	12,35
70ppb002	239240	69,7705	Std.	3	Yes	8,345	10,19
100 ppb001	325325	92,5082	Std.	4	Yes	8,340	15,62
100ppb002	325332	92,5101	Std.	4	Yes	8,341	14,90
130 ppb001	475635	132,2096	Std.	5	Yes	8,339	15,52
130ppb002	464438	129,2523	Std.	5	Yes	8,345	15,97
160 ppb001	578269	159,3184	Std.	6	Yes	8,345	19,08
160ppb002	571536	157,5402	Std.	6	Yes	8,343	19,58
190 ppb001	685010	187,5122	Std.	7	Yes	8,344	17,91
190ppb002	688469	188,4258	Std.	7	Yes	8,336	15,72
220 ppb001	796514	216,9639	Std.	8	Yes	8,341	17,32
220ppb002	830114	225,8385	Std.	8	Yes	8,347	16,97
250 ppb001	938827	254,5530	Std.	9	Yes	8,337	16,43
250 ppb002	931087	252,5086	Std.	9	Yes	8,343	16,26
10H	79686	27,6274	Unk.	----	----	8,364	13,85
10M	92729	31,0724	Unk.	----	----	8,372	8,70
9H	351776	99,4947	Unk.	----	----	8,355	14,54
9M	813490	221,4477	Unk.	----	----	8,359	12,88
Average	424395	118,6757				8,352	14,33
%RSD	73	69,4245				0,353	30,40
Maximum	938827	254,5530				8,480	19,58
Minimum	12569	9,8998				8,333	0,00
Std. Dev.	311929	82,3901				0,029	4,36

Compound: Ac. Cafeico (continued)

0
Conc 9,8998
Area 12569
R#1 179,20>134,00 0,00 (17,32)
R#2 179,20>106,90 0,00 (3,96)
Q 179,20>135,00 (-) 7,62e3

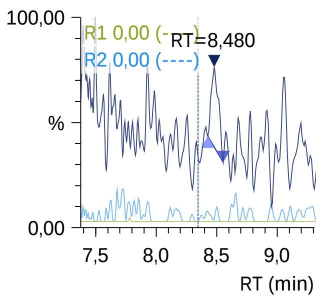

10ppb001
Conc 18,5967
Area 45496
R#1 179,20>134,00 7,65 (17,32)
R#2 179,20>106,90 1,47 (3,96)
Q 179,20>135,00 (-) 1,18e4

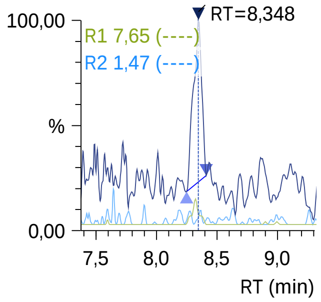

10 ppb002
Conc 12,5069
Area 22440
R#1 179,20>134,00 13,78 (17,32)
R#2 179,20>106,90 0,00 (3,96)
Q 179,20>135,00 (-) 9,13e3

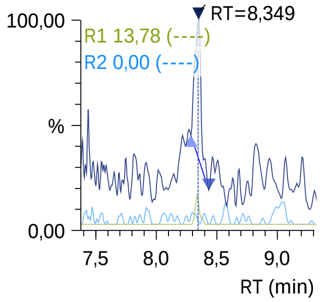

40 ppb001
Conc 39,1461
Area 123296
R#1 179,20>134,00 16,41 (17,32)
R#2 179,20>106,90 4,36 (3,96)
Q 179,20>135,00 (-) 3,39e4

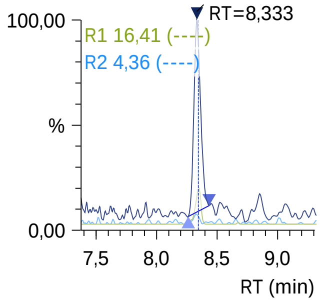

40ppb002
Conc 37,2976
Area 116298
R#1 179,20>134,00 17,89 (17,32)
R#2 179,20>106,90 4,64 (3,96)
Q 179,20>135,00 (-) 2,84e4

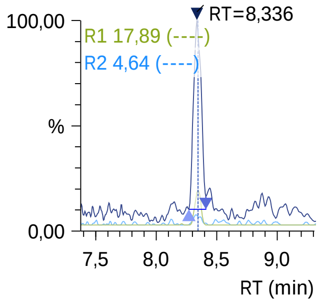

70 ppb001
Conc 73,5415
Area 253517
R#1 179,20>134,00 12,35 (17,32)
R#2 179,20>106,90 5,50 (3,96)
Q 179,20>135,00 (-) 6,25e4

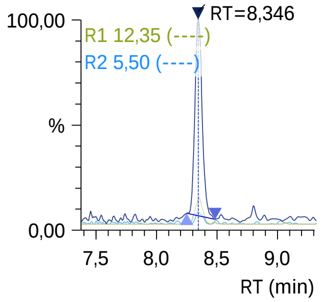

70ppb002
Conc 69,7705
Area 239240
R#1 179,20>134,00 10,19 (17,32)
R#2 179,20>106,90 5,23 (3,96)
Q 179,20>135,00 (-) 6,45e4

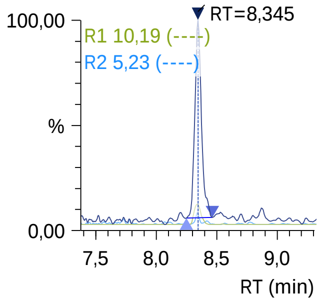

100 ppb001
Conc 92,5082
Area 325325
R#1 179,20>134,00 15,62 (17,32)
R#2 179,20>106,90 5,58 (3,96)
Q 179,20>135,00 (-) 7,87e4

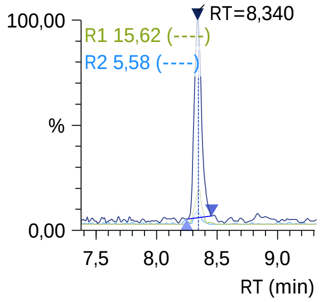

100ppb002
Conc 92,5101
Area 325332
R#1 179,20>134,00 14,90 (17,32)
R#2 179,20>106,90 3,30 (3,96)
Q 179,20>135,00 (-) 8,31e4

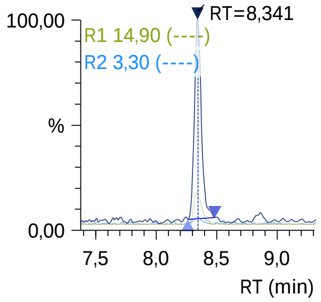

130 ppb001
Conc 132,2096
Area 475635
R#1 179,20>134,00 15,52 (17,32)
R#2 179,20>106,90 2,76 (3,96)
Q 179,20>135,00 (-) 1,24e5

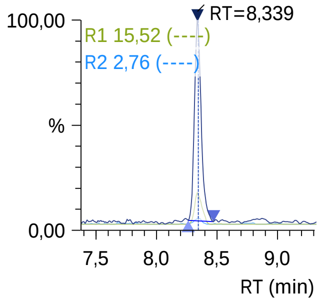

130ppb002
Conc 129,2523
Area 464438
R#1 179,20>134,00 15,97 (17,32)
R#2 179,20>106,90 4,55 (3,96)
Q 179,20>135,00 (-) 1,15e5

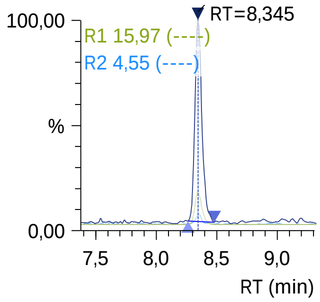

160 ppb001
Conc 159,3184
Area 578269
R#1 179,20>134,00 19,08 (17,32)
R#2 179,20>106,90 3,87 (3,96)
Q 179,20>135,00 (-) 1,39e5

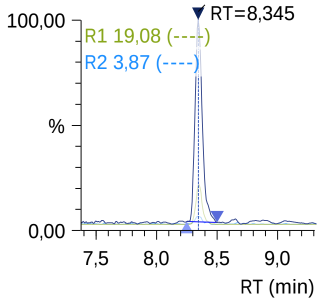

Compound: Ac. Cafeico (continued)

160ppb002
Conc 157,5402
Area 571536
R#1 179,20>134,00 19,58 (17,32)
R#2 179,20>106,90 4,15 (3,96)
Q 179,20>135,00 (-) 1,41e5

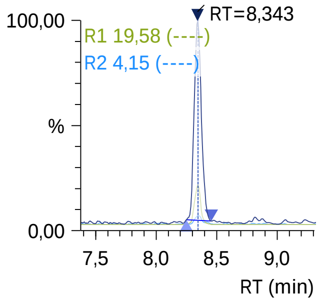

190 ppb001
Conc 187,5122
Area 685010
R#1 179,20>134,00 17,91 (17,32)
R#2 179,20>106,90 5,02 (3,96)
Q 179,20>135,00 (-) 1,71e5

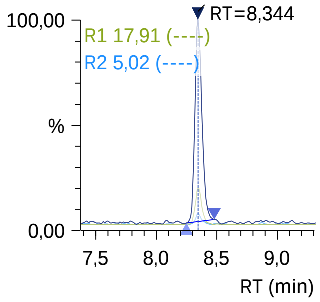

190ppb002
Conc 188,4258
Area 688469
R#1 179,20>134,00 15,72 (17,32)
R#2 179,20>106,90 4,08 (3,96)
Q 179,20>135,00 (-) 1,77e5

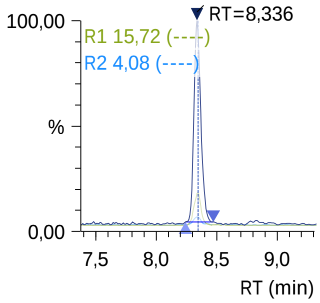

220 ppb001
Conc 216,9639
Area 796514
R#1 179,20>134,00 17,32 (17,32)
R#2 179,20>106,90 3,96 (3,96)
Q 179,20>135,00 (-) 1,93e5

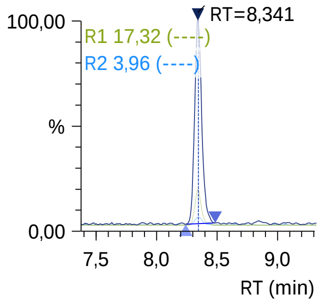

220ppb002
Conc 225,8385
Area 830114
R#1 179,20>134,00 16,97 (17,32)
R#2 179,20>106,90 3,83 (3,96)
Q 179,20>135,00 (-) 2,07e5

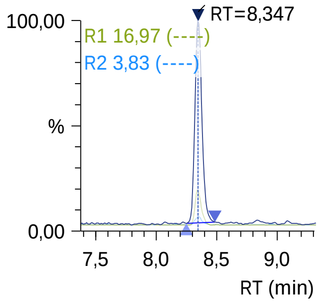

250 ppb001
Conc 254,5530
Area 938827
R#1 179,20>134,00 16,43 (17,32)
R#2 179,20>106,90 3,97 (3,96)
Q 179,20>135,00 (-) 2,36e5

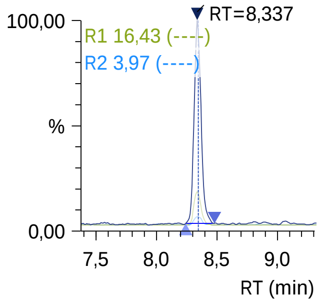

250 ppb002
Conc 252,5086
Area 931087
R#1 179,20>134,00 16,26 (17,32)
R#2 179,20>106,90 3,41 (3,96)
Q 179,20>135,00 (-) 2,35e5

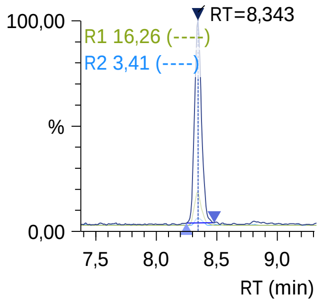

10H
Conc 27,6274
Area 79686
R#1 179,20>134,00 13,85 (17,32)
R#2 179,20>106,90 0,44 (3,96)
Q 179,20>135,00 (-) 1,88e4

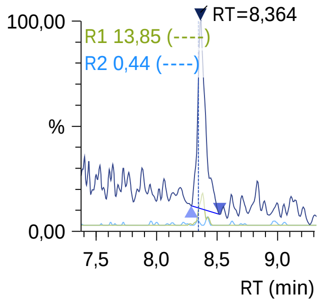

10M
Conc 31,0724
Area 92729
R#1 179,20>134,00 8,70 (17,32)
R#2 179,20>106,90 4,27 (3,96)
Q 179,20>135,00 (-) 2,32e4

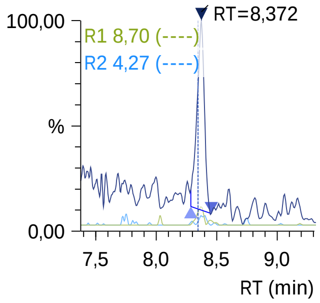

9H
Conc 99,4947
Area 351776
R#1 179,20>134,00 14,54 (17,32)
R#2 179,20>106,90 3,62 (3,96)
Q 179,20>135,00 (-) 8,28e4

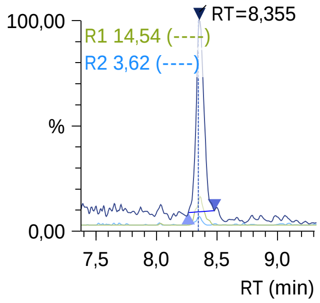

9M
Conc 221,4477
Area 813490
R#1 179,20>134,00 12,88 (17,32)
R#2 179,20>106,90 3,08 (3,96)
Q 179,20>135,00 (-) 2,00e5

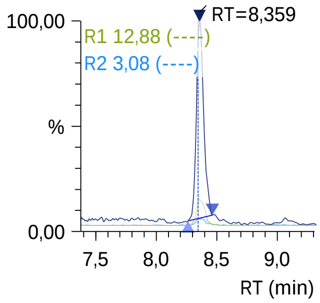

Compound: Coniferil Aldeido

Curve Fit: Linear | Weighting: None | Zero: Not Forced
Quantitative Method: External Standard
Q 177,20>162,05
R^2 = 0.9858206 R = 0.9928850
y = 1010,621x + 6663,198

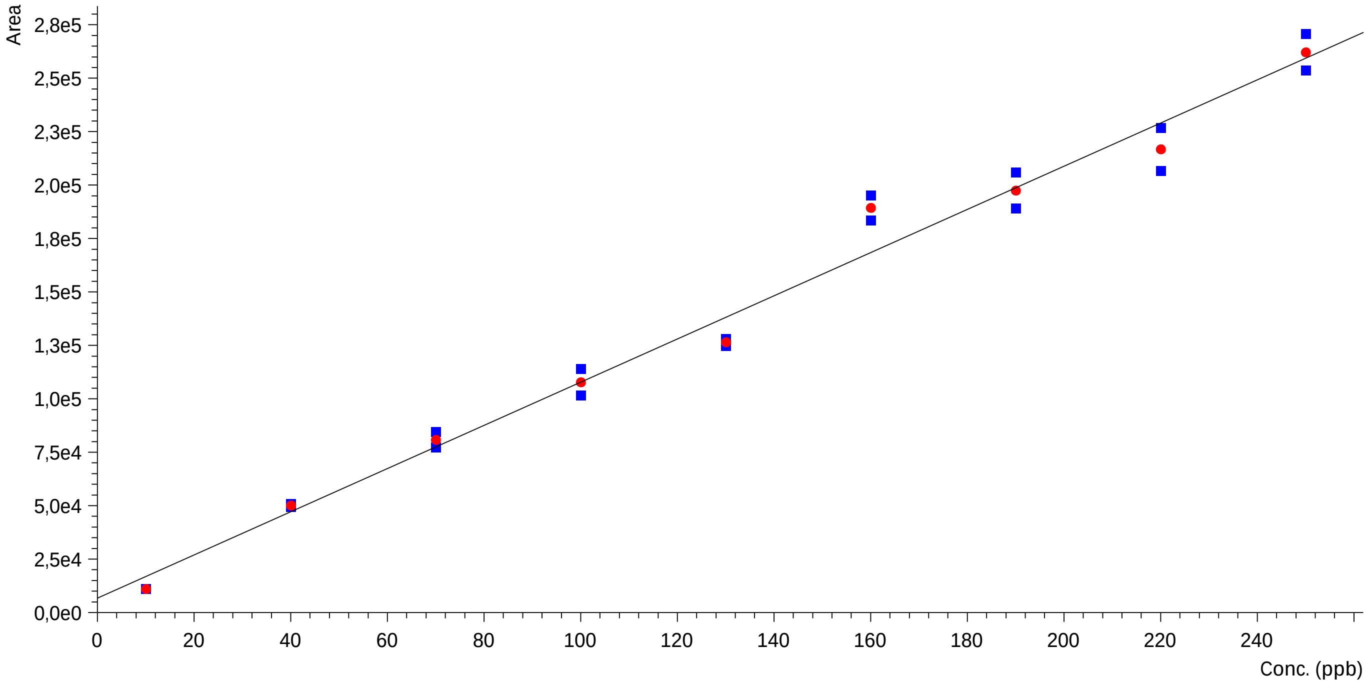

Sample ID	Area	Conc.	Sample Type	Level	Cal Point	Found RT	Ref 1 Actual Ratio
0	----	----	Ctrl.	0	----	----	----
10ppb001	----	----	Std.	1	No	----	----
10 ppb002	11052	4,3426	Std.	1	Yes	9,079	0,00
40 ppb001	50959	43,8303	Std.	2	Yes	9,073	0,00
40ppb002	49450	42,3372	Std.	2	Yes	9,078	2,46
70 ppb001	84373	76,8927	Std.	3	Yes	9,078	3,62
70ppb002	77476	70,0682	Std.	3	Yes	9,089	2,24
100 ppb001	101558	93,8972	Std.	4	Yes	9,084	6,27
100ppb002	114124	106,3318	Std.	4	Yes	9,085	1,91
130 ppb001	124762	116,8572	Std.	5	Yes	9,078	2,74
130ppb002	128169	120,2293	Std.	5	Yes	9,081	6,63
160 ppb001	183619	175,0962	Std.	6	Yes	9,086	6,17
160ppb002	195289	186,6436	Std.	6	Yes	9,081	5,50
190 ppb001	189046	180,4661	Std.	7	Yes	9,086	3,83
190ppb002	205823	197,0665	Std.	7	Yes	9,082	5,73
220 ppb001	226829	217,8523	Std.	8	Yes	9,081	7,41
220ppb002	206719	197,9538	Std.	8	Yes	9,087	7,46
250 ppb001	270749	261,3105	Std.	9	Yes	9,076	5,68
250 ppb002	253742	244,4822	Std.	9	Yes	9,080	4,80
10H	----	----	Unk.	----	----	----	----
10M	----	----	Unk.	----	----	----	----
9H	21826	15,0032	Unk.	----	----	9,109	4,87
9M	12148	5,4270	Unk.	----	----	9,095	0,00
Average	131985	124,0046				9,084	4,07
%RSD	63	66,5195				0,089	60,57
Maximum	270749	261,3105				9,109	7,46
Minimum	11052	4,3426				9,073	0,00
Std. Dev.	83363	82,4873				0,008	2,47

Compound: Coniferil Aldeido (continued)

0
Conc ----
Area ----
R#1 177,20>133,95 ---- (0,00)
R#2 177,20>104,90 ---- (0,00)
Q 177,20>162,05 (-) 2,54e2

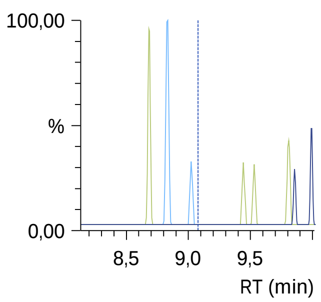

10ppb001
Conc ----
Area ----
R#1 177,20>133,95 ---- (0,00)
R#2 177,20>104,90 ---- (0,00)
Q 177,20>162,05 (-) 1,83e3

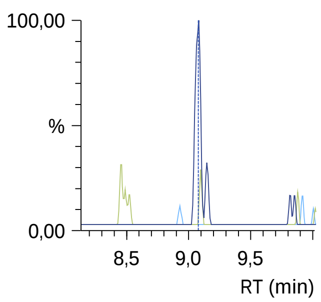

10 ppb002
Conc 4,3426
Area 11052
R#1 177,20>133,95 0,00 (0,00)
R#2 177,20>104,90 3,63 (0,00)
Q 177,20>162,05 (-) 3,82e3

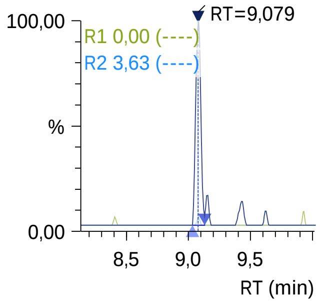

40 ppb001
Conc 43,8303
Area 50959
R#1 177,20>133,95 0,00 (0,00)
R#2 177,20>104,90 0,00 (0,00)
Q 177,20>162,05 (-) 1,31e4

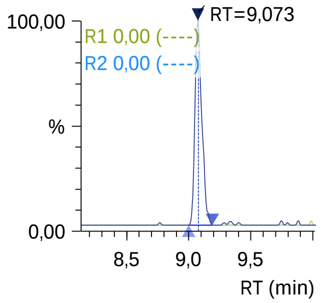

40ppb002
Conc 42,3372
Area 49450
R#1 177,20>133,95 2,46 (0,00)
R#2 177,20>104,90 0,00 (0,00)
Q 177,20>162,05 (-) 1,33e4

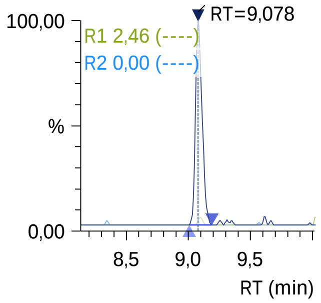

70 ppb001
Conc 76,8927
Area 84373
R#1 177,20>133,95 3,62 (0,00)
R#2 177,20>104,90 0,00 (0,00)
Q 177,20>162,05 (-) 2,22e4

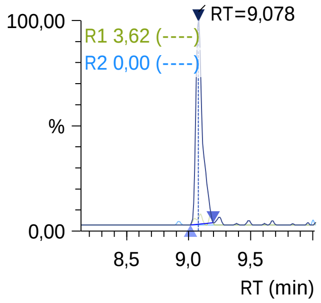

70ppb002
Conc 70,0682
Area 77476
R#1 177,20>133,95 2,24 (0,00)
R#2 177,20>104,90 0,00 (0,00)
Q 177,20>162,05 (-) 1,88e4

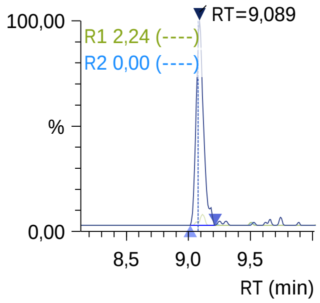

100 ppb001
Conc 93,8972
Area 101558
R#1 177,20>133,95 6,27 (0,00)
R#2 177,20>104,90 0,23 (0,00)
Q 177,20>162,05 (-) 2,90e4

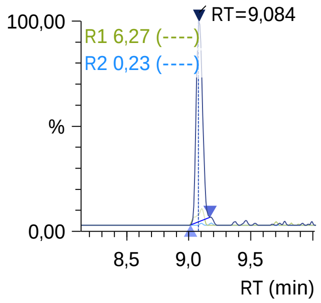

100ppb002
Conc 106,3318
Area 114124
R#1 177,20>133,95 1,91 (0,00)
R#2 177,20>104,90 1,84 (0,00)
Q 177,20>162,05 (-) 2,82e4

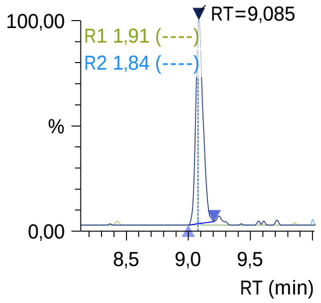

130 ppb001
Conc 116,8572
Area 124762
R#1 177,20>133,95 2,74 (0,00)
R#2 177,20>104,90 0,18 (0,00)
Q 177,20>162,05 (-) 3,30e4

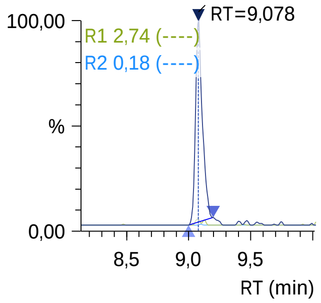

130ppb002
Conc 120,2293
Area 128169
R#1 177,20>133,95 6,63 (0,00)
R#2 177,20>104,90 0,00 (0,00)
Q 177,20>162,05 (-) 3,32e4

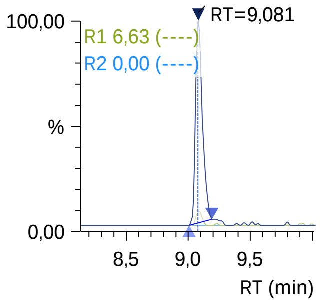

160 ppb001
Conc 175,0962
Area 183619
R#1 177,20>133,95 6,17 (0,00)
R#2 177,20>104,90 0,37 (0,00)
Q 177,20>162,05 (-) 4,76e4

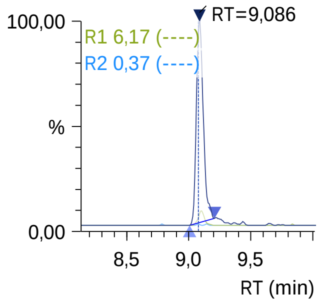

Compound: Coniferil Aldeido (continued)

160ppb002
Conc 186,6436
Area 195289
R#1 177,20>133,95 5,50 (0,00)
R#2 177,20>104,90 0,88 (0,00)
Q 177,20>162,05 (-) 5,05e4

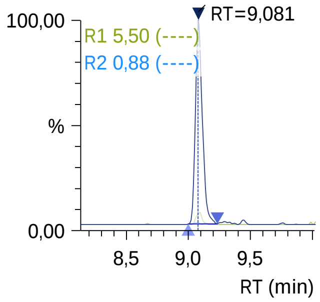

190 ppb001
Conc 180,4661
Area 189046
R#1 177,20>133,95 3,83 (0,00)
R#2 177,20>104,90 2,04 (0,00)
Q 177,20>162,05 (-) 4,85e4

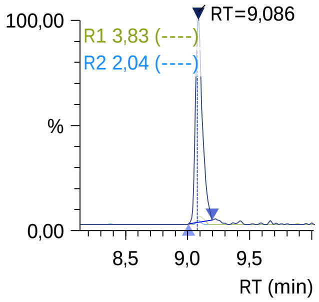

190ppb002
Conc 197,0665
Area 205823
R#1 177,20>133,95 5,73 (0,00)
R#2 177,20>104,90 0,37 (0,00)
Q 177,20>162,05 (-) 5,24e4

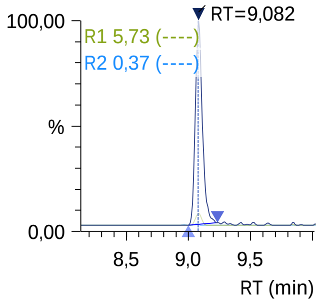

220 ppb001
Conc 217,8523
Area 226829
R#1 177,20>133,95 7,41 (0,00)
R#2 177,20>104,90 0,00 (0,00)
Q 177,20>162,05 (-) 5,73e4

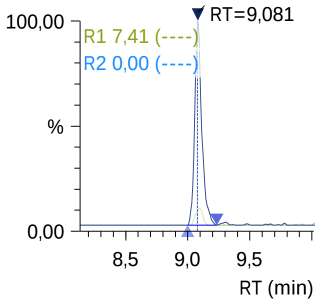

220ppb002
Conc 197,9538
Area 206719
R#1 177,20>133,95 7,46 (0,00)
R#2 177,20>104,90 1,29 (0,00)
Q 177,20>162,05 (-) 5,44e4

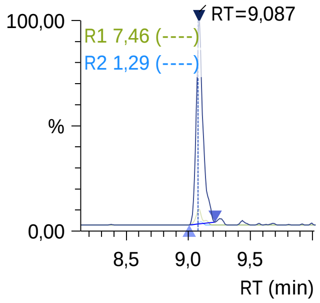

250 ppb001
Conc 261,3105
Area 270749
R#1 177,20>133,95 5,68 (0,00)
R#2 177,20>104,90 1,36 (0,00)
Q 177,20>162,05 (-) 6,98e4

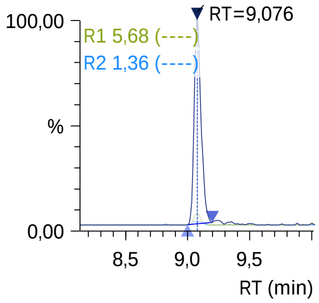

250 ppb002
Conc 244,4822
Area 253742
R#1 177,20>133,95 4,80 (0,00)
R#2 177,20>104,90 1,01 (0,00)
Q 177,20>162,05 (-) 6,93e4

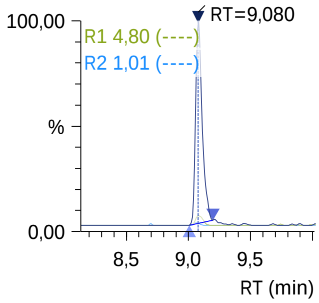

10H
Conc ----
Area ----
R#1 177,20>133,95 ---- (0,00)
R#2 177,20>104,90 ---- (0,00)
Q 177,20>162,05 (-) 9,93e2

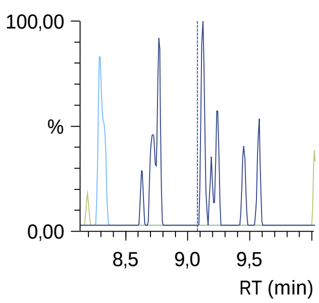

10M
Conc ----
Area ----
R#1 177,20>133,95 ---- (0,00)
R#2 177,20>104,90 ---- (0,00)
Q 177,20>162,05 (-) 1,49e3

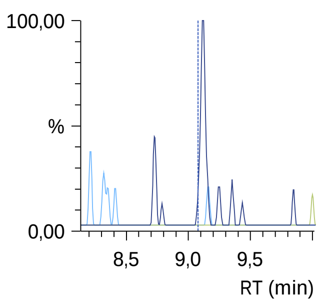

9H
Conc 15,0032
Area 21826
R#1 177,20>133,95 4,87 (0,00)
R#2 177,20>104,90 0,00 (0,00)
Q 177,20>162,05 (-) 7,24e3

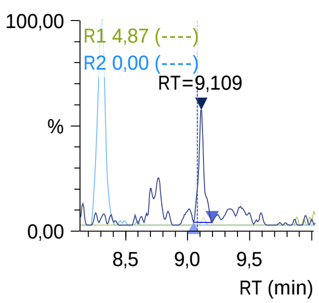

9M
Conc 5,4270
Area 12148
R#1 177,20>133,95 0,00 (0,00)
R#2 177,20>104,90 0,00 (0,00)
Q 177,20>162,05 (-) 5,04e3

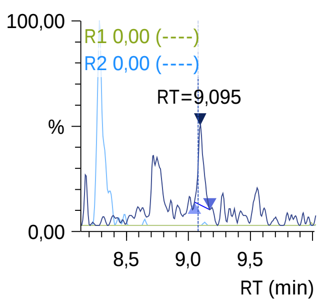

Compound: Ac. Sinapico

Curve Fit: Linear | Weighting: None | Zero: Not Forced
Quantitative Method: External Standard
Q 223,20>208,05
R^2 = 0.9902149 R = 0.9950954
y = 1222,678x - 4619,034

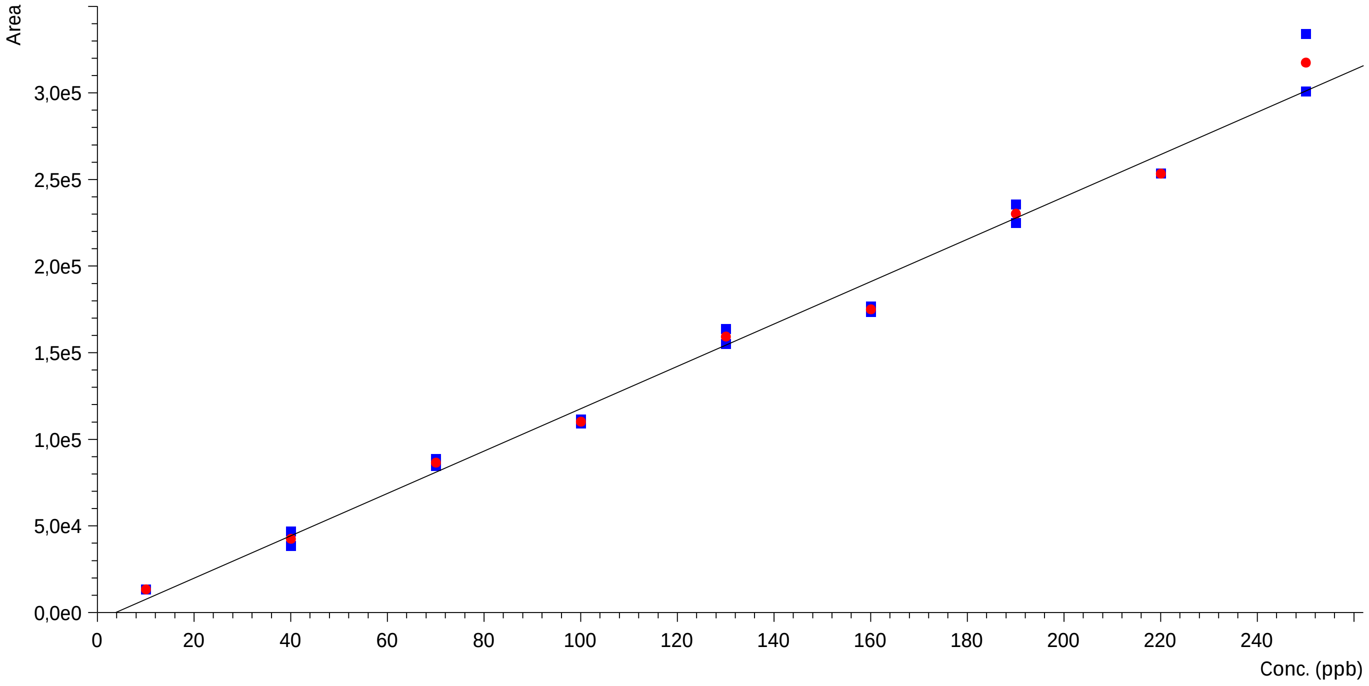

Sample ID	Area	Conc.	Sample Type	Level	Cal Point	Found RT	Ref 1 Actual Ratio
0	----	----	Ctrl.	0	----	----	----
10ppb001	13445	14,7741	Std.	1	Yes	8,898	41,99
10 ppb002	13367	14,7107	Std.	1	Yes	8,891	29,90
40 ppb001	38615	35,3603	Std.	2	Yes	8,876	69,09
40ppb002	46696	41,9693	Std.	2	Yes	8,881	50,93
70 ppb001	88806	76,4099	Std.	3	Yes	8,890	48,36
70ppb002	84667	73,0247	Std.	3	Yes	8,876	80,05
100 ppb001	109105	93,0125	Std.	4	Yes	8,876	111,83
100ppb002	111559	95,0197	Std.	4	Yes	8,873	92,40
130 ppb001	155186	130,7010	Std.	5	Yes	8,880	80,78
130ppb002	163655	137,6272	Std.	5	Yes	8,883	67,22
160 ppb001	176753	148,3400	Std.	6	Yes	8,887	69,74
160ppb002	173454	145,6419	Std.	6	Yes	8,883	64,53
190 ppb001	235594	196,4647	Std.	7	Yes	8,883	67,25
190ppb002	225027	187,8225	Std.	7	Yes	8,877	76,15
220 ppb001	253535	211,1378	Std.	8	Yes	8,883	77,81
220ppb002	253458	211,0751	Std.	8	Yes	8,886	76,68
250 ppb001	334070	277,0061	Std.	9	Yes	8,879	70,13
250 ppb002	300931	249,9025	Std.	9	Yes	8,885	81,82
10H	----	----	Unk.	----	----	----	----
10M	----	----	Unk.	----	----	----	----
9H	----	----	Unk.	----	----	----	----
9M	----	----	Unk.	----	----	----	----
Average	154329	130,0000				8,882	69,81
%RSD	64	61,7443				0,072	26,96
Maximum	334070	277,0061				8,898	111,83
Minimum	13367	14,7107				8,873	29,90
Std. Dev.	98141	80,2676				0,006	18,82

Compound: Ac. Sinapico (continued)

0
Conc ----
Area ----
R#1 223,20>193,00 ---- (92,40)
R#2 223,20>164,10 ---- (77,70)
Q 223,20>208,05 (-) 1,09e3

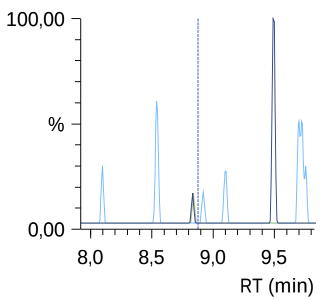

10ppb001
Conc 14,7741
Area 13445
R#1 223,20>193,00 41,99 (92,40)
R#2 223,20>164,10 106,09 (77,70)
Q 223,20>208,05 (-) 2,39e3

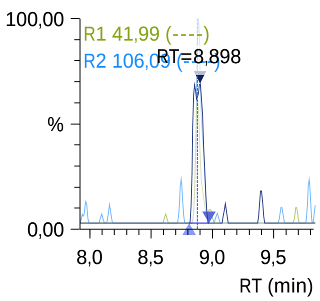

10 ppb002
Conc 14,7107
Area 13367
R#1 223,20>193,00 29,90 (92,40)
R#2 223,20>164,10 54,80 (77,70)
Q 223,20>208,05 (-) 3,59e3

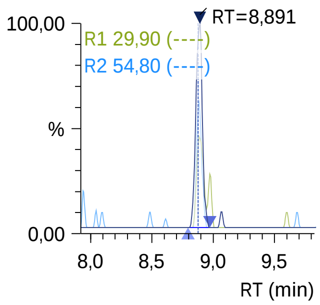

40 ppb001
Conc 35,3603
Area 38615
R#1 223,20>193,00 69,09 (92,40)
R#2 223,20>164,10 73,99 (77,70)
Q 223,20>208,05 (-) 9,93e3

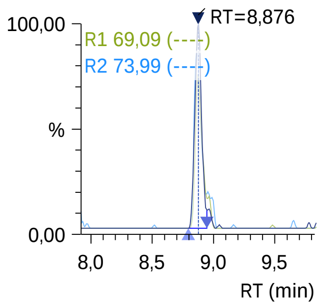

40ppb002
Conc 41,9693
Area 46696
R#1 223,20>193,00 50,93 (92,40)
R#2 223,20>164,10 72,42 (77,70)
Q 223,20>208,05 (-) 1,06e4

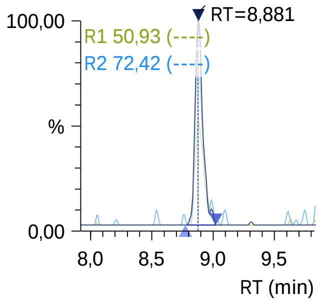

70 ppb001
Conc 76,4099
Area 88806
R#1 223,20>193,00 48,36 (92,40)
R#2 223,20>164,10 75,49 (77,70)
Q 223,20>208,05 (-) 2,21e4

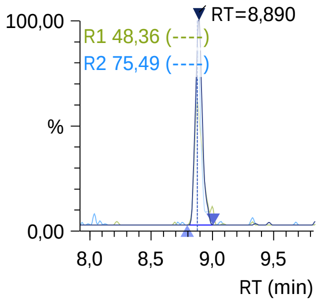

70ppb002
Conc 73,0247
Area 84667
R#1 223,20>193,00 80,05 (92,40)
R#2 223,20>164,10 75,64 (77,70)
Q 223,20>208,05 (-) 1,90e4

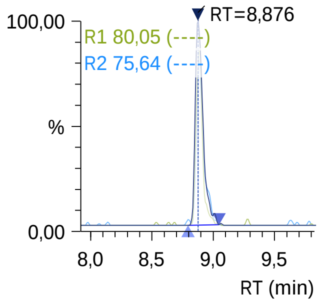

100 ppb001
Conc 93,0125
Area 109105
R#1 223,20>193,00 111,83 (92,40)
R#2 223,20>164,10 77,30 (77,70)
Q 223,20>208,05 (-) 2,17e4

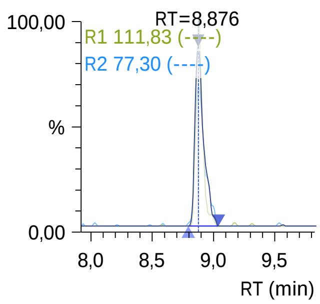

100ppb002
Conc 95,0197
Area 111559
R#1 223,20>193,00 92,40 (92,40)
R#2 223,20>164,10 77,70 (77,70)
Q 223,20>208,05 (-) 2,16e4

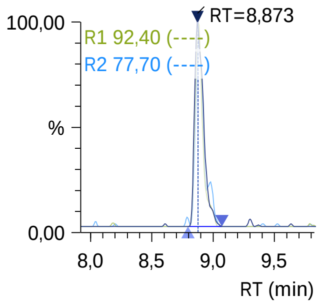

130 ppb001
Conc 130,7010
Area 155186
R#1 223,20>193,00 80,78 (92,40)
R#2 223,20>164,10 65,29 (77,70)
Q 223,20>208,05 (-) 3,58e4

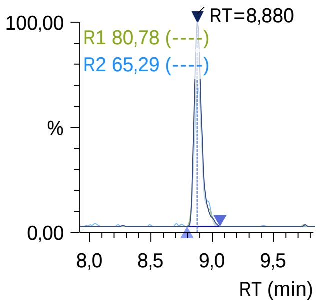

130ppb002
Conc 137,6272
Area 163655
R#1 223,20>193,00 67,22 (92,40)
R#2 223,20>164,10 67,76 (77,70)
Q 223,20>208,05 (-) 3,81e4

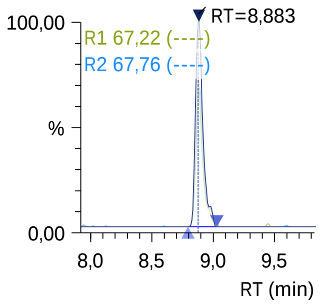

160 ppb001
Conc 148,3400
Area 176753
R#1 223,20>193,00 69,74 (92,40)
R#2 223,20>164,10 67,44 (77,70)
Q 223,20>208,05 (-) 4,14e4

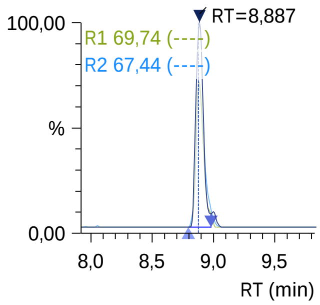

Compound: Ac. Sinapico (continued)

160ppb002
Conc 145,6419
Area 173454
R#1 223,20>193,00 64,53 (92,40)
R#2 223,20>164,10 76,30 (77,70)
Q 223,20>208,05 (-) 4,29e4

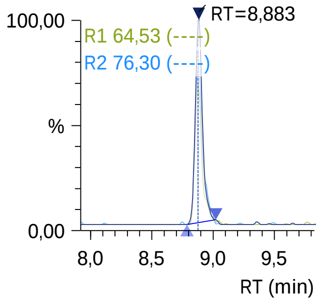

190 ppb001
Conc 196,4647
Area 235594
R#1 223,20>193,00 67,25 (92,40)
R#2 223,20>164,10 61,15 (77,70)
Q 223,20>208,05 (-) 5,27e4

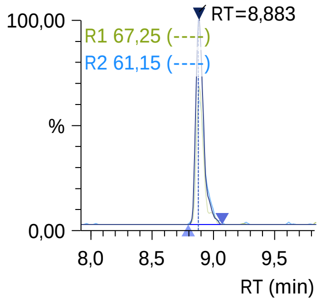

190ppb002
Conc 187,8225
Area 225027
R#1 223,20>193,00 76,15 (92,40)
R#2 223,20>164,10 78,52 (77,70)
Q 223,20>208,05 (-) 5,07e4

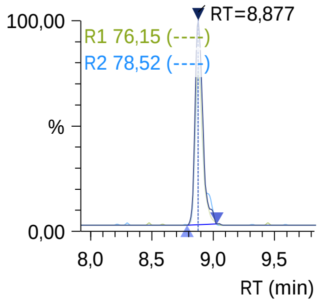

220 ppb001
Conc 211,1378
Area 253535
R#1 223,20>193,00 77,81 (92,40)
R#2 223,20>164,10 87,53 (77,70)
Q 223,20>208,05 (-) 5,62e4

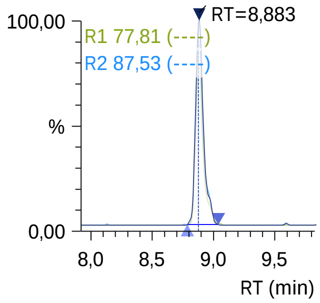

220ppb002
Conc 211,0751
Area 253458
R#1 223,20>193,00 76,68 (92,40)
R#2 223,20>164,10 68,68 (77,70)
Q 223,20>208,05 (-) 6,00e4

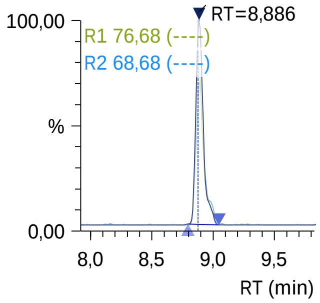

250 ppb001
Conc 277,0061
Area 334070
R#1 223,20>193,00 70,13 (92,40)
R#2 223,20>164,10 74,86 (77,70)
Q 223,20>208,05 (-) 7,62e4

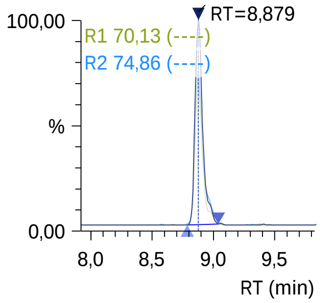

250 ppb002
Conc 249,9025
Area 300931
R#1 223,20>193,00 81,82 (92,40)
R#2 223,20>164,10 69,15 (77,70)
Q 223,20>208,05 (-) 6,72e4

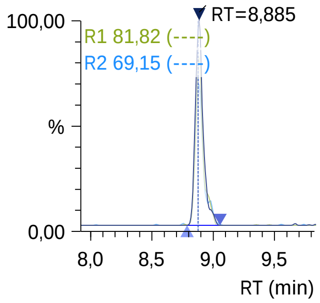

10H
Conc ----
Area ----
R#1 223,20>193,00 ---- (92,40)
R#2 223,20>164,10 ---- (77,70)
Q 223,20>208,05 (-) 1,42e3

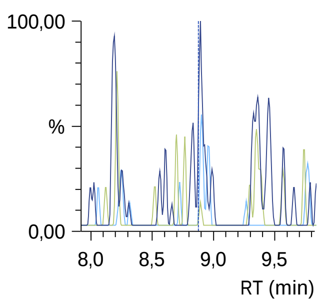

10M
Conc ----
Area ----
R#1 223,20>193,00 ---- (92,40)
R#2 223,20>164,10 ---- (77,70)
Q 223,20>208,05 (-) 1,46e3

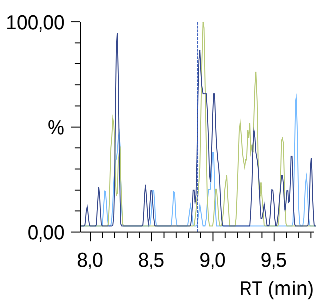

9H
Conc ----
Area ----
R#1 223,20>193,00 ---- (92,40)
R#2 223,20>164,10 ---- (77,70)
Q 223,20>208,05 (-) 5,38e3

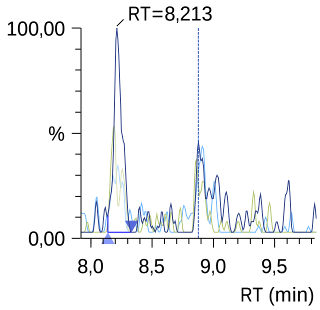

9M
Conc ----
Area ----
R#1 223,20>193,00 ---- (92,40)
R#2 223,20>164,10 ---- (77,70)
Q 223,20>208,05 (-) 6,55e3

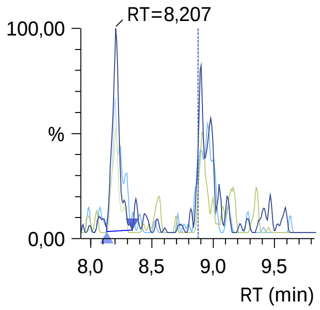

Compound: Seringaldazina

Curve Fit: Linear | Weighting: None | Zero: Not Forced
Quantitative Method: External Standard
Q 359,10>344,10
R^2 = 0.2000250 R = 0.4472415
y = 84,63672x + 24133,46

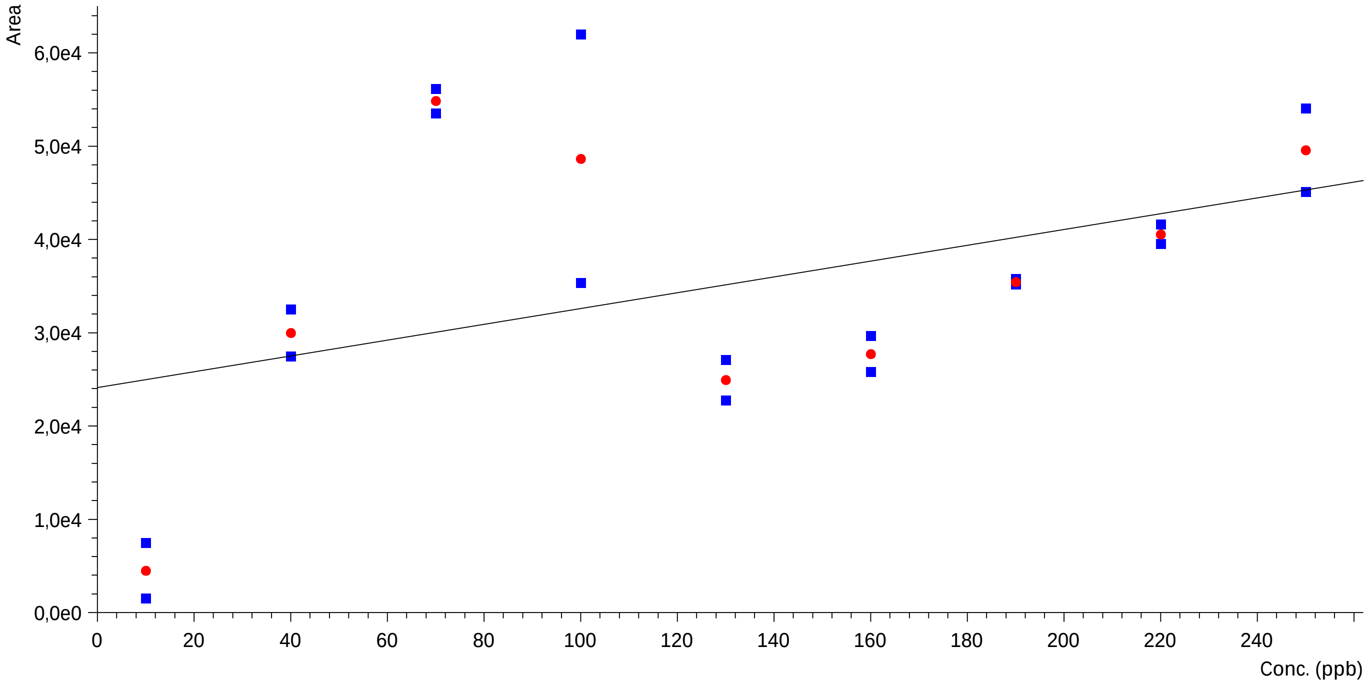

Sample ID	Area	Conc.	Sample Type	Level	Cal Point	Found RT	Ref 1 Actual Ratio
0	----	----	Ctrl.	0	----	----	----
10ppb001	1504	-267,3679	Std.	1	Yes	9,816	82,53
10 ppb002	7473	-196,8458	Std.	1	Yes	9,844	8,50
40 ppb001	32503	98,8915	Std.	2	Yes	9,851	59,13
40ppb002	27477	39,5074	Std.	2	Yes	9,867	77,80
70 ppb001	53510	347,0891	Std.	3	Yes	9,857	51,73
70ppb002	56176	378,5838	Std.	3	Yes	9,856	41,41
100 ppb001	61990	447,2822	Std.	4	Yes	9,856	53,58
100ppb002	35323	132,2017	Std.	4	Yes	9,865	44,24
130 ppb001	27113	35,2038	Std.	5	Yes	9,866	34,32
130ppb002	22748	-16,3647	Std.	5	Yes	9,859	43,60
160 ppb001	25813	19,8464	Std.	6	Yes	9,859	61,04
160ppb002	29653	65,2175	Std.	6	Yes	9,834	33,51
190 ppb001	35738	137,1053	Std.	7	Yes	9,863	38,34
190ppb002	35159	130,2743	Std.	7	Yes	9,854	46,41
220 ppb001	41602	206,3979	Std.	8	Yes	9,839	79,04
220ppb002	39517	181,7546	Std.	8	Yes	9,849	48,20
250 ppb001	54041	353,3662	Std.	9	Yes	9,845	50,76
250 ppb002	45111	247,8566	Std.	9	Yes	9,838	62,33
10H	----	----	Unk.	----	----	----	----
10M	----	----	Unk.	----	----	----	----
9H	----	----	Unk.	----	----	----	----
9M	----	----	Unk.	----	----	----	----
Average	35136	130,0000				9,851	50,91
%RSD	45	144,8223				0,133	35,56
Maximum	61990	447,2822				9,867	82,53
Minimum	1504	-267,3679				9,816	8,50
Std. Dev.	15934	188,2689				0,013	18,11

Compound: Seringaldazina (continued)

0
Conc ----
Area ----
R#1 359,10>329,00 ---- (48,20)
R#2 359,10>147,90 ---- (18,11)
Q 359,10>344,10 (-) 5,66e2

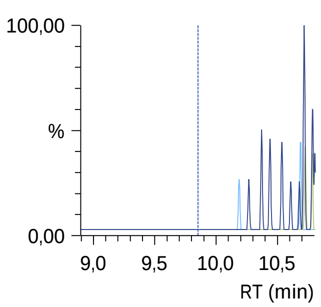

10ppb001
Conc -267,3679
Area 1504
R#1 359,10>329,00 82,53 (48,20)
R#2 359,10>147,90 0,00 (18,11)
Q 359,10>344,10 (-) 6,93e2

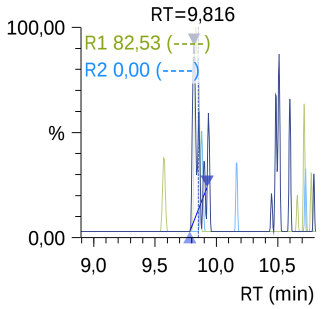

10 ppb002
Conc -196,8458
Area 7473
R#1 359,10>329,00 8,50 (48,20)
R#2 359,10>147,90 1,09 (18,11)
Q 359,10>344,10 (-) 1,76e3

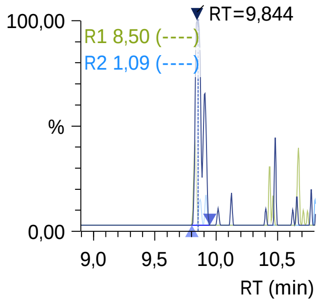

40 ppb001
Conc 98,8915
Area 32503
R#1 359,10>329,00 59,13 (48,20)
R#2 359,10>147,90 27,26 (18,11)
Q 359,10>344,10 (-) 7,64e3

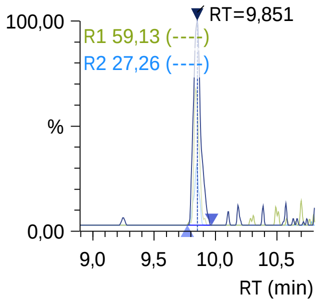

40ppb002
Conc 39,5074
Area 27477
R#1 359,10>329,00 77,80 (48,20)
R#2 359,10>147,90 14,63 (18,11)
Q 359,10>344,10 (-) 6,52e3

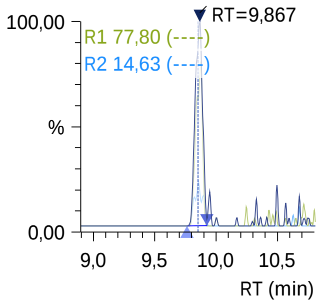

70 ppb001
Conc 347,0891
Area 53510
R#1 359,10>329,00 51,73 (48,20)
R#2 359,10>147,90 20,84 (18,11)
Q 359,10>344,10 (-) 1,37e4

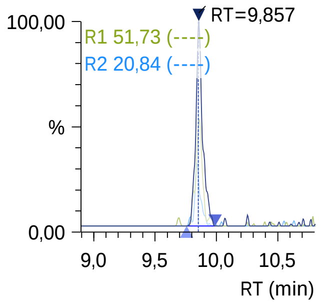

70ppb002
Conc 378,5838
Area 56176
R#1 359,10>329,00 41,41 (48,20)
R#2 359,10>147,90 27,05 (18,11)
Q 359,10>344,10 (-) 1,44e4

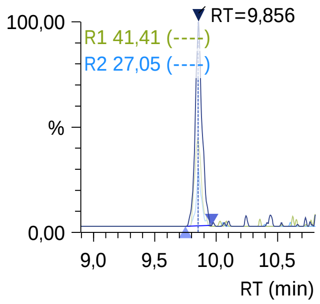

100 ppb001
Conc 447,2822
Area 61990
R#1 359,10>329,00 53,58 (48,20)
R#2 359,10>147,90 35,94 (18,11)
Q 359,10>344,10 (-) 1,33e4

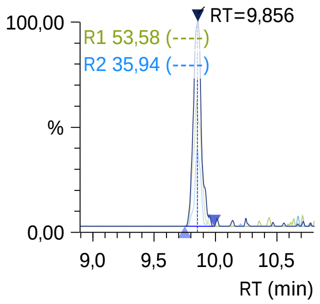

100ppb002
Conc 132,2017
Area 35323
R#1 359,10>329,00 44,24 (48,20)
R#2 359,10>147,90 26,92 (18,11)
Q 359,10>344,10 (-) 1,01e4

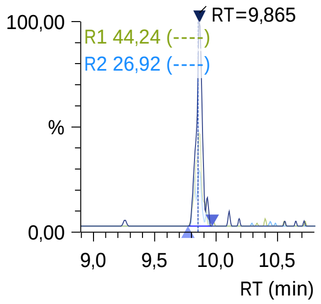

130 ppb001
Conc 35,2038
Area 27113
R#1 359,10>329,00 34,32 (48,20)
R#2 359,10>147,90 20,21 (18,11)
Q 359,10>344,10 (-) 6,81e3

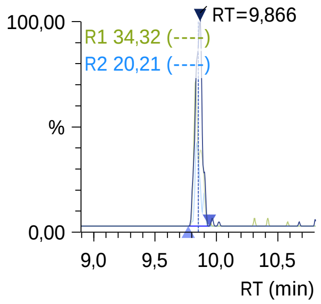

130ppb002
Conc -16,3647
Area 22748
R#1 359,10>329,00 43,60 (48,20)
R#2 359,10>147,90 28,96 (18,11)
Q 359,10>344,10 (-) 5,55e3

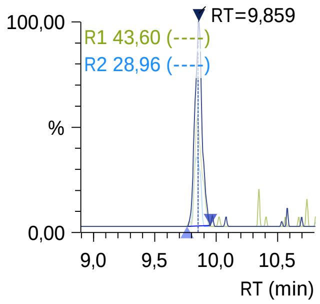

160 ppb001
Conc 19,8464
Area 25813
R#1 359,10>329,00 61,04 (48,20)
R#2 359,10>147,90 31,98 (18,11)
Q 359,10>344,10 (-) 5,94e3

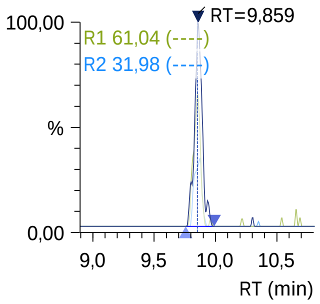

Compound: Seringaldazina (continued)

160ppb002
Conc 65,2175
Area 29653
R#1 359,10>329,00 33,51 (48,20)
R#2 359,10>147,90 32,92 (18,11)
Q 359,10>344,10 (-) 6,34e3

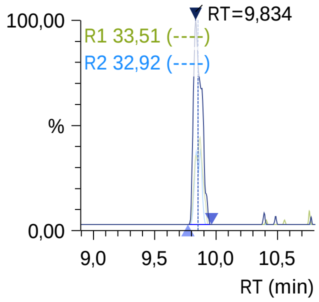

190 ppb001
Conc 137,1053
Area 35738
R#1 359,10>329,00 38,34 (48,20)
R#2 359,10>147,90 15,94 (18,11)
Q 359,10>344,10 (-) 9,34e3

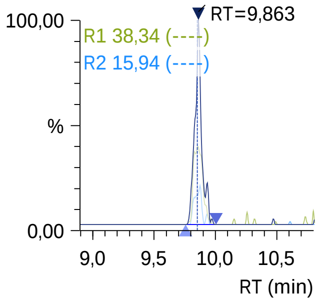

190ppb002
Conc 130,2743
Area 35159
R#1 359,10>329,00 46,41 (48,20)
R#2 359,10>147,90 29,60 (18,11)
Q 359,10>344,10 (-) 7,97e3

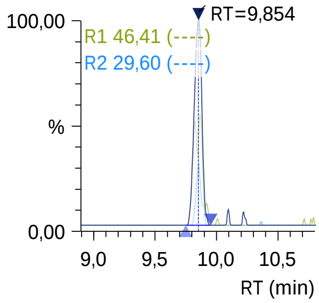

220 ppb001
Conc 206,3979
Area 41602
R#1 359,10>329,00 79,04 (48,20)
R#2 359,10>147,90 28,40 (18,11)
Q 359,10>344,10 (-) 8,54e3

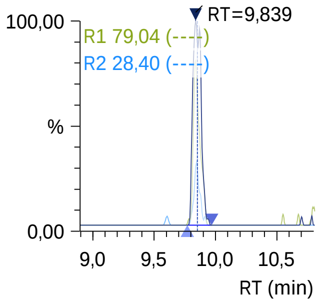

220ppb002
Conc 181,7546
Area 39517
R#1 359,10>329,00 48,20 (48,20)
R#2 359,10>147,90 18,11 (18,11)
Q 359,10>344,10 (-) 1,04e4

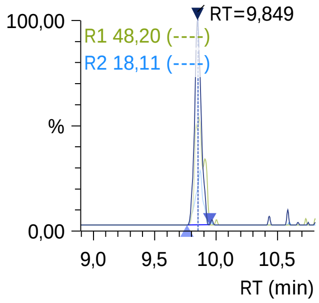

250 ppb001
Conc 353,3662
Area 54041
R#1 359,10>329,00 50,76 (48,20)
R#2 359,10>147,90 33,59 (18,11)
Q 359,10>344,10 (-) 1,20e4

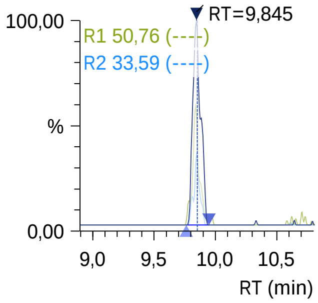

250 ppb002
Conc 247,8566
Area 45111
R#1 359,10>329,00 62,33 (48,20)
R#2 359,10>147,90 23,10 (18,11)
Q 359,10>344,10 (-) 9,96e3

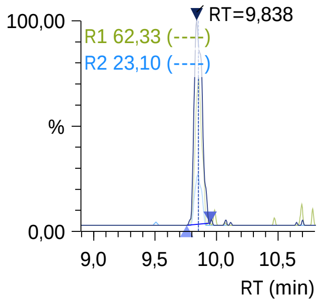

10H
Conc ----
Area ----
R#1 359,10>329,00 ---- (48,20)
R#2 359,10>147,90 ---- (18,11)
Q 359,10>344,10 (-) 6,03e3

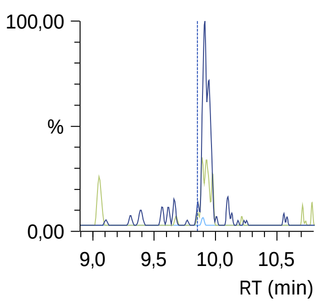

10M
Conc ----
Area ----
R#1 359,10>329,00 ---- (48,20)
R#2 359,10>147,90 ---- (18,11)
Q 359,10>344,10 (-) 5,34e3

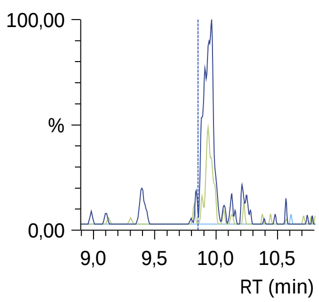

9H
Conc ----
Area ----
R#1 359,10>329,00 ---- (48,20)
R#2 359,10>147,90 ---- (18,11)
Q 359,10>344,10 (-) 1,11e4

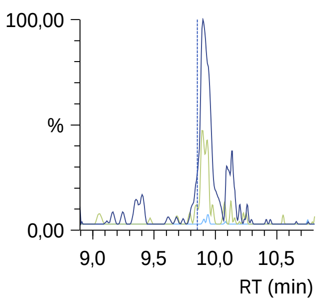

9M
Conc ----
Area ----
R#1 359,10>329,00 ---- (48,20)
R#2 359,10>147,90 ---- (18,11)
Q 359,10>344,10 (-) 1,37e4

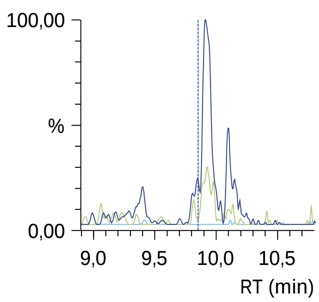

Compound: Catequina

Curve Fit: Linear | Weighting: None | Zero: Not Forced
Quantitative Method: External Standard
Q 289,10>245,10
R^2 = 0.9929667 R = 0.9964771
y = 1162,653x - 8457,580

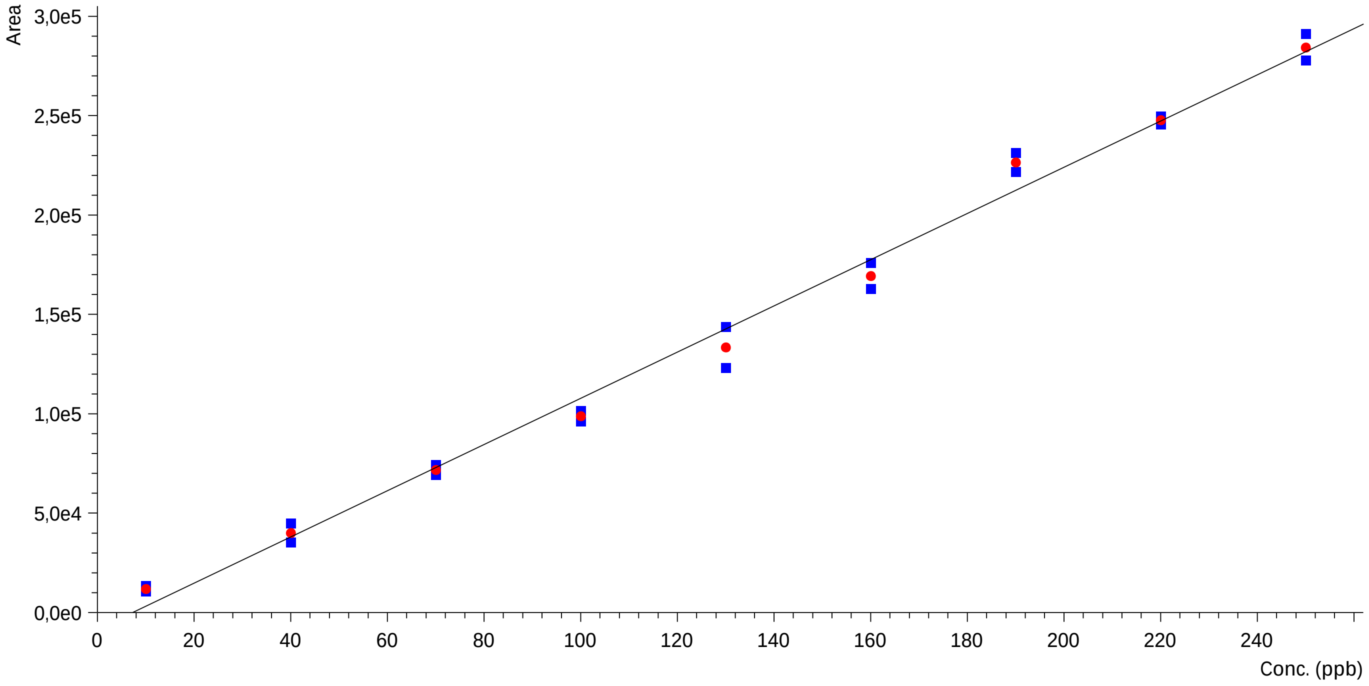

Sample ID	Area	Conc.	Sample Type	Level	Cal Point	Found RT	Ref 1 Actual Ratio
0	----	----	Ctrl.	0	----	----	----
10ppb001	13322	18,7330	Std.	1	Yes	7,651	49,71
10 ppb002	10585	16,3787	Std.	1	Yes	7,637	51,66
40 ppb001	44849	45,8492	Std.	2	Yes	7,650	29,84
40ppb002	35312	37,6464	Std.	2	Yes	7,643	46,57
70 ppb001	69170	66,7680	Std.	3	Yes	7,649	53,67
70ppb002	74419	71,2821	Std.	3	Yes	7,651	44,49
100 ppb001	101479	94,5563	Std.	4	Yes	7,647	53,18
100ppb002	96180	89,9990	Std.	4	Yes	7,648	43,54
130 ppb001	123114	113,1645	Std.	5	Yes	7,642	52,65
130ppb002	143841	130,9919	Std.	5	Yes	8,041	49,23
160 ppb001	175945	158,6046	Std.	6	Yes	7,643	45,30
160ppb002	162859	147,3497	Std.	6	Yes	7,639	54,90
190 ppb001	231264	206,1845	Std.	7	Yes	7,645	52,92
190ppb002	221726	197,9809	Std.	7	Yes	7,647	52,67
220 ppb001	249811	222,1373	Std.	8	Yes	7,655	43,86
220ppb002	245701	218,6023	Std.	8	Yes	7,651	41,07
250 ppb001	291102	257,6514	Std.	9	Yes	7,639	53,96
250 ppb002	277695	246,1200	Std.	9	Yes	7,646	48,15
10H	----	----	Unk.	----	----	----	----
10M	----	----	Unk.	----	----	----	----
9H	----	----	Unk.	----	----	----	----
9M	----	----	Unk.	----	----	----	----
Average	142687	130,0000				7,668	48,19
%RSD	65	61,6375				1,216	12,96
Maximum	291102	257,6514				8,041	54,90
Minimum	10585	16,3787				7,637	29,84
Std. Dev.	93162	80,1288				0,093	6,25

Compound: Catequina (continued)

0
Conc ----
Area ----
R#1 289,10>108,95 ---- (44,49)
R#2 289,10>203,05 ---- (41,50)

Q 289,10>245,10 (-)   1,04e3

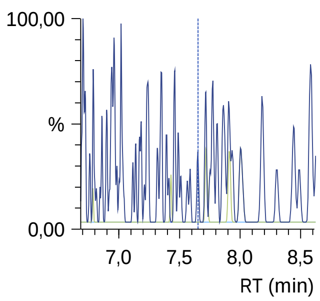

10ppb001
Conc 18,7330
Area 13322
R#1 289,10>108,95 49,71 (44,49)
R#2 289,10>203,05 41,94 (41,50)

Q 289,10>245,10 (-)   3,78e3

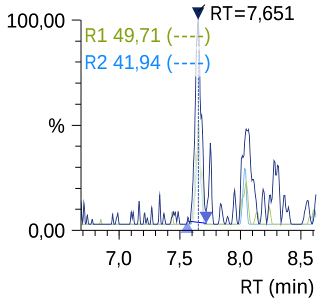

10 ppb002
Conc 16,3787
Area 10585
R#1 289,10>108,95 51,66 (44,49)
R#2 289,10>203,05 115,32 (41,50)

Q 289,10>245,10 (-)   2,58e3

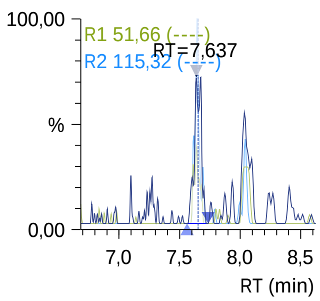

40 ppb001
Conc 45,8492
Area 44849
R#1 289,10>108,95 29,84 (44,49)
R#2 289,10>203,05 34,06 (41,50)

Q 289,10>245,10 (-)   1,39e4

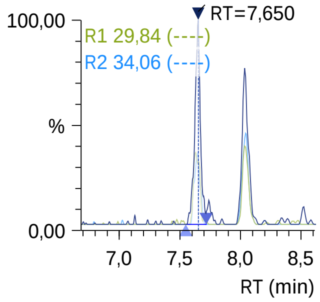

40ppb002
Conc 37,6464
Area 35312
R#1 289,10>108,95 46,57 (44,49)
R#2 289,10>203,05 35,83 (41,50)

Q 289,10>245,10 (-)   1,28e4

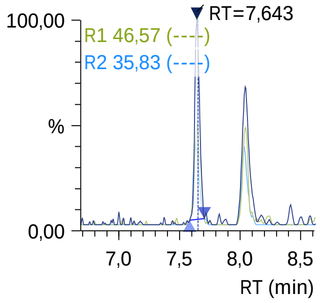

70 ppb001
Conc 66,7680
Area 69170
R#1 289,10>108,95 53,67 (44,49)
R#2 289,10>203,05 50,37 (41,50)

Q 289,10>245,10 (-)   2,30e4

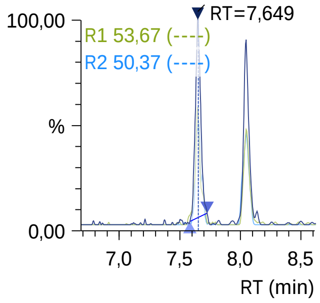

70ppb002
Conc 71,2821
Area 74419
R#1 289,10>108,95 44,49 (44,49)
R#2 289,10>203,05 41,50 (41,50)

Q 289,10>245,10 (-)   2,21e4

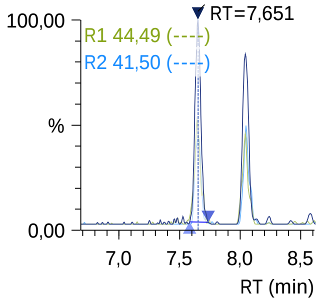

100 ppb001
Conc 94,5563
Area 101479
R#1 289,10>108,95 53,18 (44,49)
R#2 289,10>203,05 43,43 (41,50)

Q 289,10>245,10 (-)   2,94e4

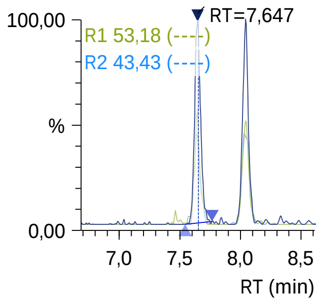

100ppb002
Conc 89,9990
Area 96180
R#1 289,10>108,95 43,54 (44,49)
R#2 289,10>203,05 59,48 (41,50)

Q 289,10>245,10 (-)   2,71e4

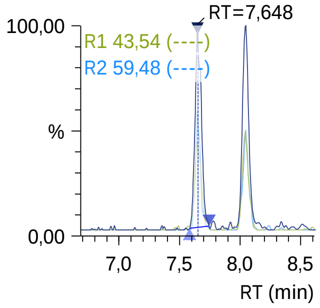

130 ppb001
Conc 113,1645
Area 123114
R#1 289,10>108,95 52,65 (44,49)
R#2 289,10>203,05 45,14 (41,50)

Q 289,10>245,10 (-)   4,00e4

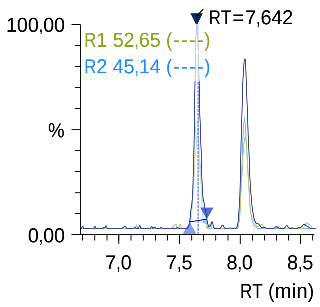

130ppb002
Conc 130,9919
Area 143841
R#1 289,10>108,95 49,23 (44,49)
R#2 289,10>203,05 48,95 (41,50)

Q 289,10>245,10 (-)   3,61e4

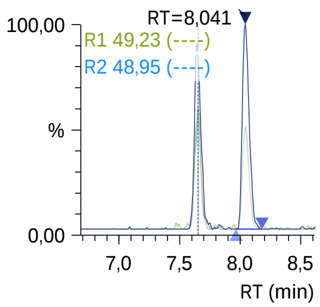

160 ppb001
Conc 158,6046
Area 175945
R#1 289,10>108,95 45,30 (44,49)
R#2 289,10>203,05 49,85 (41,50)

Q 289,10>245,10 (-)   4,59e4

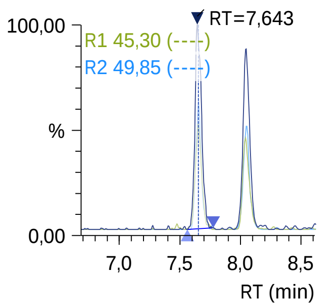

Compound: Catequina (continued)

160ppb002
Conc 147,3497
Area 162859
R#1 289,10>108,95 54,90 (44,49)
R#2 289,10>203,05 64,99 (41,50)
Q 289,10>245,10 (-) 4,44e4

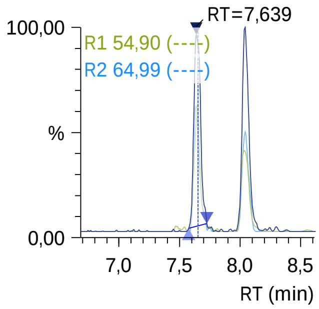

190 ppb001
Conc 206,1845
Area 231264
R#1 289,10>108,95 52,92 (44,49)
R#2 289,10>203,05 50,28 (41,50)
Q 289,10>245,10 (-) 6,06e4

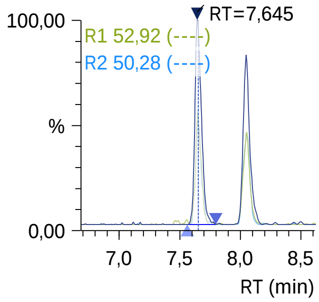

190ppb002
Conc 197,9809
Area 221726
R#1 289,10>108,95 52,67 (44,49)
R#2 289,10>203,05 42,35 (41,50)
Q 289,10>245,10 (-) 6,26e4

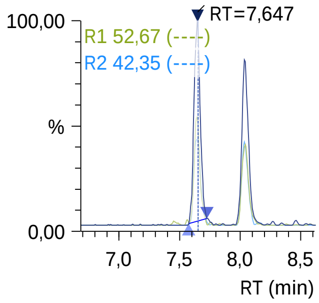

220 ppb001
Conc 222,1373
Area 249811
R#1 289,10>108,95 43,86 (44,49)
R#2 289,10>203,05 38,44 (41,50)
Q 289,10>245,10 (-) 8,24e4

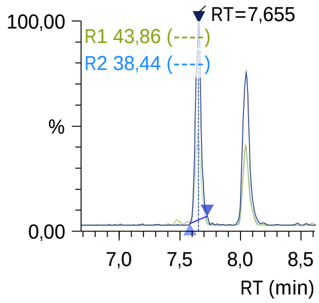

220ppb002
Conc 218,6023
Area 245701
R#1 289,10>108,95 41,07 (44,49)
R#2 289,10>203,05 50,31 (41,50)
Q 289,10>245,10 (-) 7,02e4

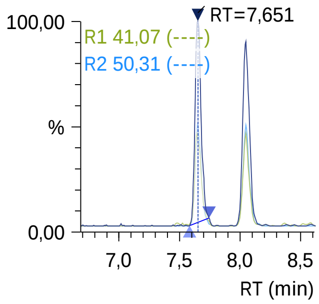

250 ppb001
Conc 257,6514
Area 291102
R#1 289,10>108,95 53,96 (44,49)
R#2 289,10>203,05 41,87 (41,50)
Q 289,10>245,10 (-) 8,56e4

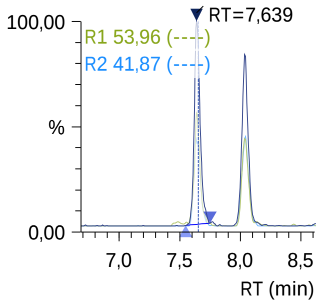

250 ppb002
Conc 246,1200
Area 277695
R#1 289,10>108,95 48,15 (44,49)
R#2 289,10>203,05 42,17 (41,50)
Q 289,10>245,10 (-) 8,33e4

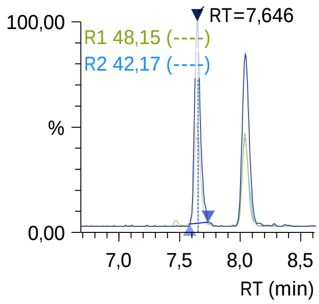

10H
Conc ----
Area ----
R#1 289,10>108,95 ---- (44,49)
R#2 289,10>203,05 ---- (41,50)
Q 289,10>245,10 (-) 5,26e3

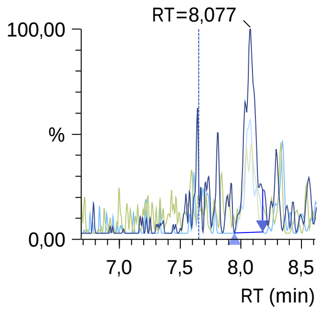

10M
Conc ----
Area ----
R#1 289,10>108,95 ---- (44,49)
R#2 289,10>203,05 ---- (41,50)
Q 289,10>245,10 (-) 3,44e3

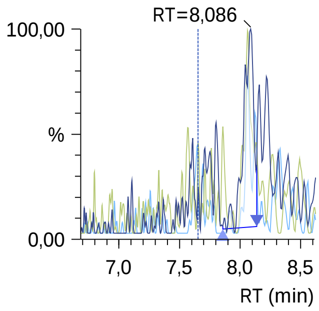

9H
Conc ----
Area ----
R#1 289,10>108,95 ---- (44,49)
R#2 289,10>203,05 ---- (41,50)
Q 289,10>245,10 (-) 3,44e3

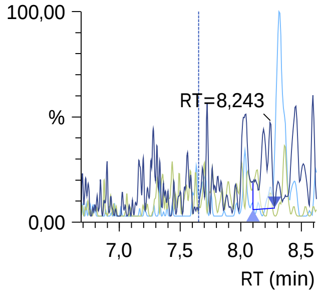

9M
Conc ----
Area ----
R#1 289,10>108,95 ---- (44,49)
R#2 289,10>203,05 ---- (41,50)
Q 289,10>245,10 (-) 5,79e3

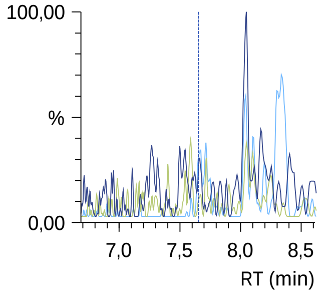

Compound: Sinapaldeido

Curve Fit: Linear | Weighting: None | Zero: Not Forced
Quantitative Method: External Standard
Q 207,20>177,05
R^2 = 0.9985656 R = 0.9992826
y = 1360,995x - 1052,942

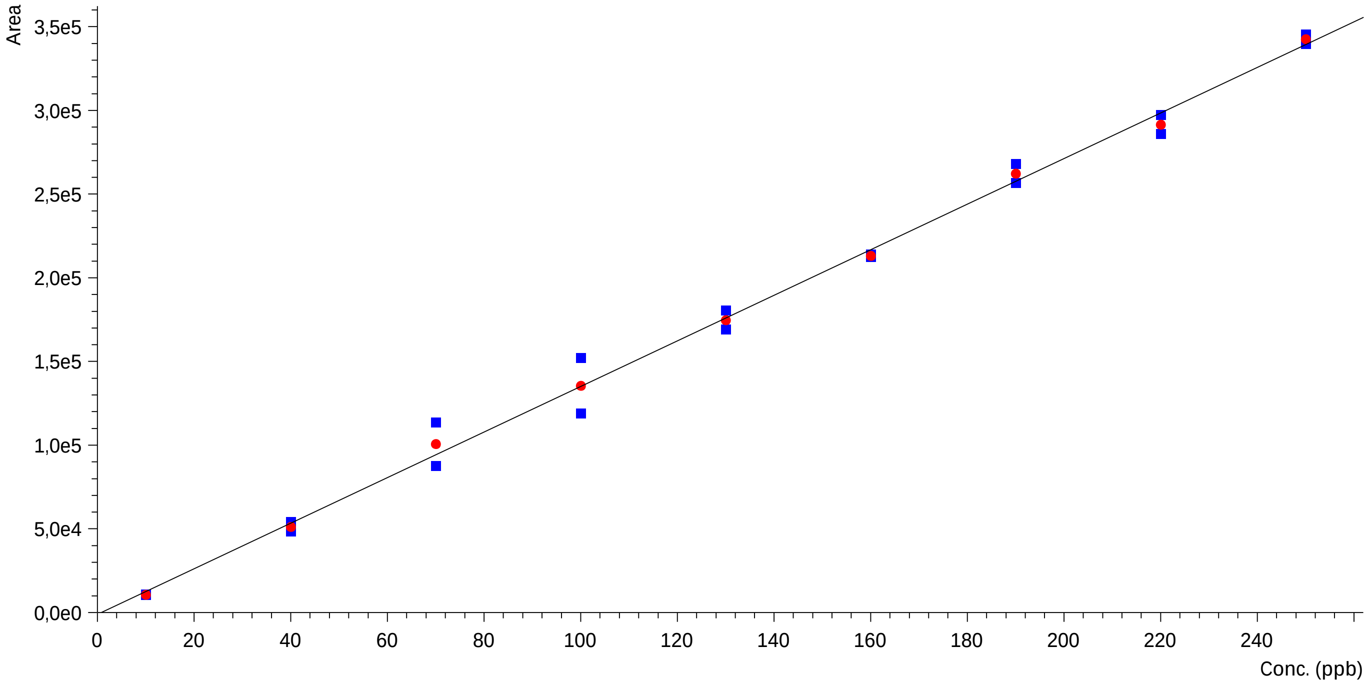

Sample ID	Area	Conc.	Sample Type	Level	Cal Point	Found RT	Ref 1 Actual Ratio
0	----	----	Ctrl.	0	----	----	----
10ppb001	10954	8,8220	Std.	1	Yes	9,020	40,42
10 ppb002	10430	8,4375	Std.	1	Yes	9,042	85,49
40 ppb001	48536	36,4361	Std.	2	Yes	9,035	73,05
40ppb002	54072	40,5035	Std.	2	Yes	9,052	71,08
70 ppb001	113810	84,3966	Std.	3	Yes	9,044	66,49
70ppb002	87577	65,1214	Std.	3	Yes	9,043	95,21
100 ppb001	152111	112,5379	Std.	4	Yes	9,042	79,89
100ppb002	118925	88,1543	Std.	4	Yes	9,046	66,74
130 ppb001	180410	133,3309	Std.	5	Yes	9,043	82,94
130ppb002	169222	125,1108	Std.	5	Yes	9,051	73,01
160 ppb001	212565	156,9573	Std.	6	Yes	9,049	65,53
160ppb002	213954	157,9780	Std.	6	Yes	9,044	84,79
190 ppb001	268017	197,7010	Std.	7	Yes	9,050	71,52
190ppb002	256672	189,3652	Std.	7	Yes	9,040	68,72
220 ppb001	285986	210,9040	Std.	8	Yes	9,041	75,29
220ppb002	297234	219,1685	Std.	8	Yes	9,053	93,36
250 ppb001	345552	254,6703	Std.	9	Yes	9,041	77,95
250 ppb002	339747	250,4047	Std.	9	Yes	9,039	94,86
10H	6962	5,8887	Unk.	----	----	9,060	51,36
10M	4967	4,4231	Unk.	----	----	9,056	107,15
9H	13608	10,7719	Unk.	----	----	9,039	54,40
9M	17409	13,5650	Unk.	----	----	9,087	28,76
Average	145851	107,9386				9,046	73,09
%RSD	81	80,1049				0,136	25,01
Maximum	345552	254,6703				9,087	107,15
Minimum	4967	4,4231				9,020	28,76
Std. Dev.	117677	86,4641				0,012	18,28

Compound: Sinapaldeido (continued)

0
Conc ----
Area ----
R#1 207,20>192,05 ---- (95,21)
R#2 207,20>149,00 ---- (35,11)
Q 207,20>177,05 (-) 4,32e2

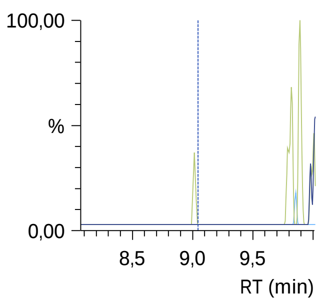

10ppb001
Conc 8,8220
Area 10954
R#1 207,20>192,05 40,42 (95,21)
R#2 207,20>149,00 2,88 (35,11)
Q 207,20>177,05 (-) 2,78e3

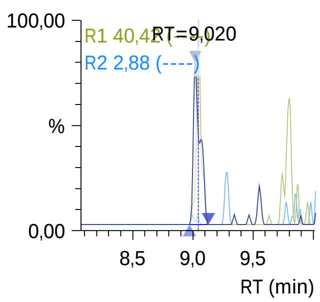

10 ppb002
Conc 8,4375
Area 10430
R#1 207,20>192,05 85,49 (95,21)
R#2 207,20>149,00 31,95 (35,11)
Q 207,20>177,05 (-) 2,83e3

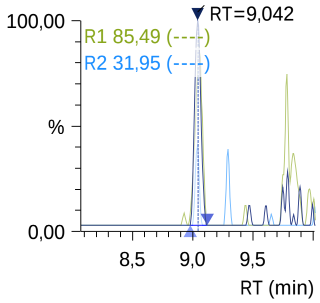

40 ppb001
Conc 36,4361
Area 48536
R#1 207,20>192,05 73,05 (95,21)
R#2 207,20>149,00 26,87 (35,11)
Q 207,20>177,05 (-) 1,38e4

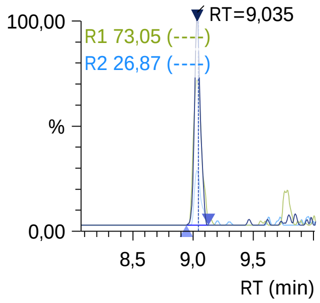

40ppb002
Conc 40,5035
Area 54072
R#1 207,20>192,05 71,08 (95,21)
R#2 207,20>149,00 30,09 (35,11)
Q 207,20>177,05 (-) 1,18e4

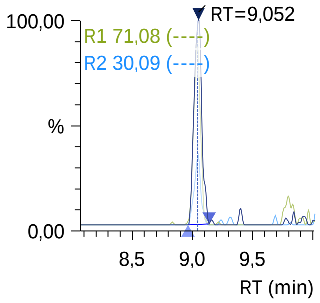

70 ppb001
Conc 84,3966
Area 113810
R#1 207,20>192,05 66,49 (95,21)
R#2 207,20>149,00 20,58 (35,11)
Q 207,20>177,05 (-) 2,96e4

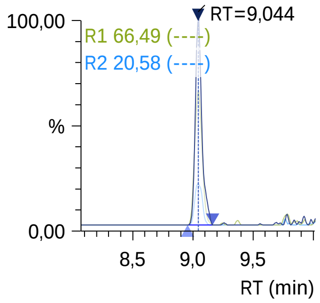

70ppb002
Conc 65,1214
Area 87577
R#1 207,20>192,05 95,21 (95,21)
R#2 207,20>149,00 35,11 (35,11)
Q 207,20>177,05 (-) 2,17e4

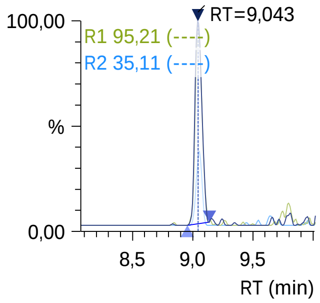

100 ppb001
Conc 112,5379
Area 152111
R#1 207,20>192,05 79,89 (95,21)
R#2 207,20>149,00 20,14 (35,11)
Q 207,20>177,05 (-) 3,94e4

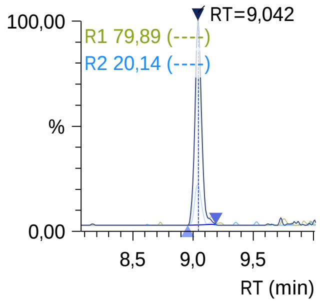

100ppb002
Conc 88,1543
Area 118925
R#1 207,20>192,05 66,74 (95,21)
R#2 207,20>149,00 26,42 (35,11)
Q 207,20>177,05 (-) 2,99e4

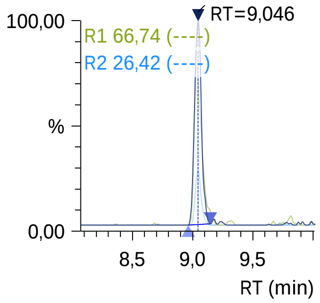

130 ppb001
Conc 133,3309
Area 180410
R#1 207,20>192,05 82,94 (95,21)
R#2 207,20>149,00 22,04 (35,11)
Q 207,20>177,05 (-) 4,36e4

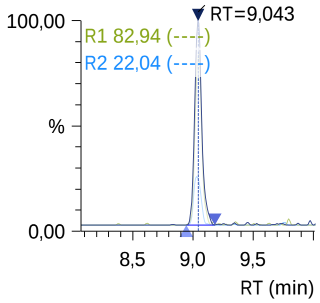

130ppb002
Conc 125,1108
Area 169222
R#1 207,20>192,05 73,01 (95,21)
R#2 207,20>149,00 21,24 (35,11)
Q 207,20>177,05 (-) 4,40e4

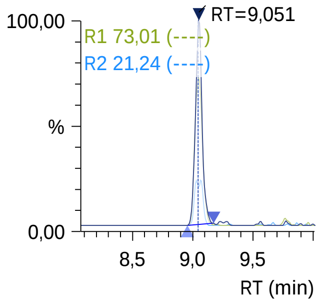

160 ppb001
Conc 156,9573
Area 212565
R#1 207,20>192,05 65,53 (95,21)
R#2 207,20>149,00 22,40 (35,11)
Q 207,20>177,05 (-) 5,47e4

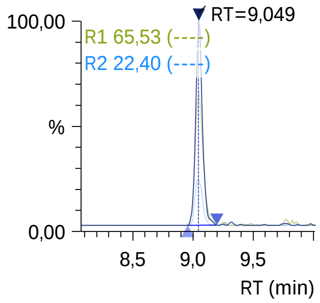

Compound: Sinapaldeido (continued)

160ppb002
Conc 157,9780
Area 213954
R#1 207,20>192,05 84,79 (95,21)
R#2 207,20>149,00 24,22 (35,11)
Q 207,20>177,05 (-) 4,85e4

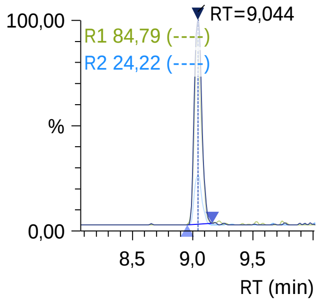

190 ppb001
Conc 197,7010
Area 268017
R#1 207,20>192,05 71,52 (95,21)
R#2 207,20>149,00 22,90 (35,11)
Q 207,20>177,05 (-) 6,57e4

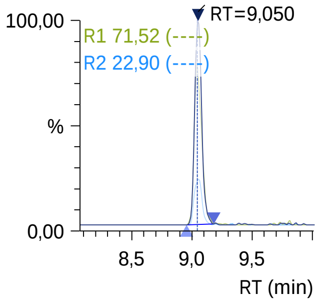

190ppb002
Conc 189,3652
Area 256672
R#1 207,20>192,05 68,72 (95,21)
R#2 207,20>149,00 17,31 (35,11)
Q 207,20>177,05 (-) 6,71e4

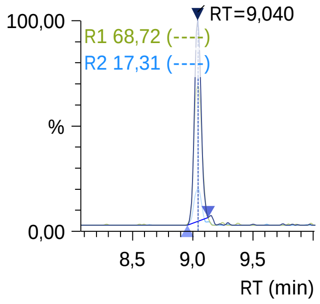

220 ppb001
Conc 210,9040
Area 285986
R#1 207,20>192,05 75,29 (95,21)
R#2 207,20>149,00 22,76 (35,11)
Q 207,20>177,05 (-) 7,15e4

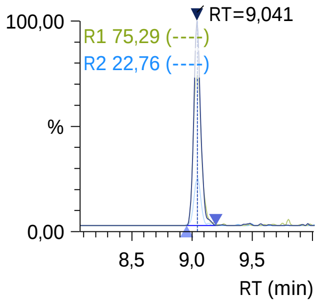

220ppb002
Conc 219,1685
Area 297234
R#1 207,20>192,05 93,36 (95,21)
R#2 207,20>149,00 27,45 (35,11)
Q 207,20>177,05 (-) 6,92e4

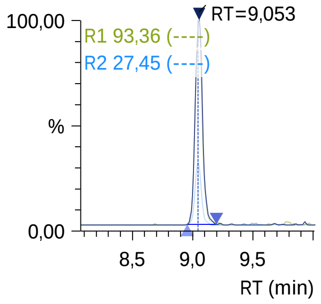

250 ppb001
Conc 254,6703
Area 345552
R#1 207,20>192,05 77,95 (95,21)
R#2 207,20>149,00 29,40 (35,11)
Q 207,20>177,05 (-) 8,72e4

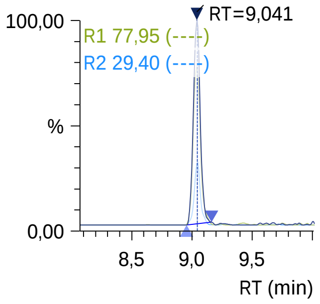

250 ppb002
Conc 250,4047
Area 339747
R#1 207,20>192,05 94,86 (95,21)
R#2 207,20>149,00 31,60 (35,11)
Q 207,20>177,05 (-) 7,62e4

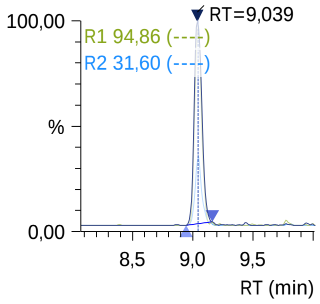

10H
Conc 5,8887
Area 6962
R#1 207,20>192,05 51,36 (95,21)
R#2 207,20>149,00 4,30 (35,11)
Q 207,20>177,05 (-) 1,83e3

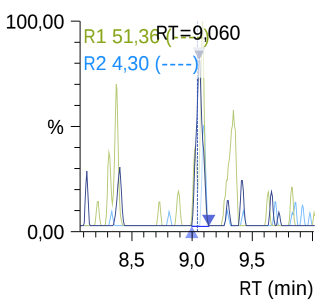

10M
Conc 4,4231
Area 4967
R#1 207,20>192,05 107,15 (95,21)
R#2 207,20>149,00 0,00 (35,11)
Q 207,20>177,05 (-) 1,94e3

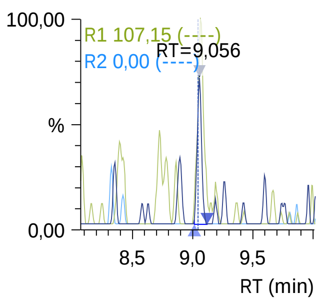

9H
Conc 10,7719
Area 13608
R#1 207,20>192,05 54,40 (95,21)
R#2 207,20>149,00 0,00 (35,11)
Q 207,20>177,05 (-) 4,49e3

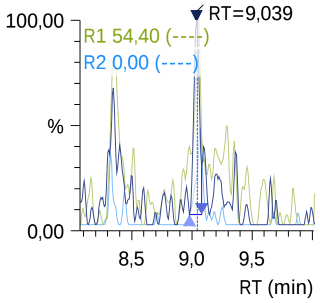

9M
Conc 13,5650
Area 17409
R#1 207,20>192,05 28,76 (95,21)
R#2 207,20>149,00 40,16 (35,11)
Q 207,20>177,05 (-) 3,93e3

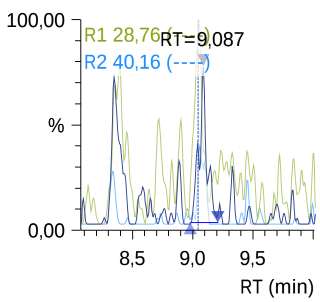

Compound: Luteolin

Curve Fit: Linear | Weighting: None | Zero: Not Forced
Quantitative Method: External Standard
Q 285,00>133,00
R^2 = 0.9736468 R = 0.9867354
y = 6508,426x + 188458,4

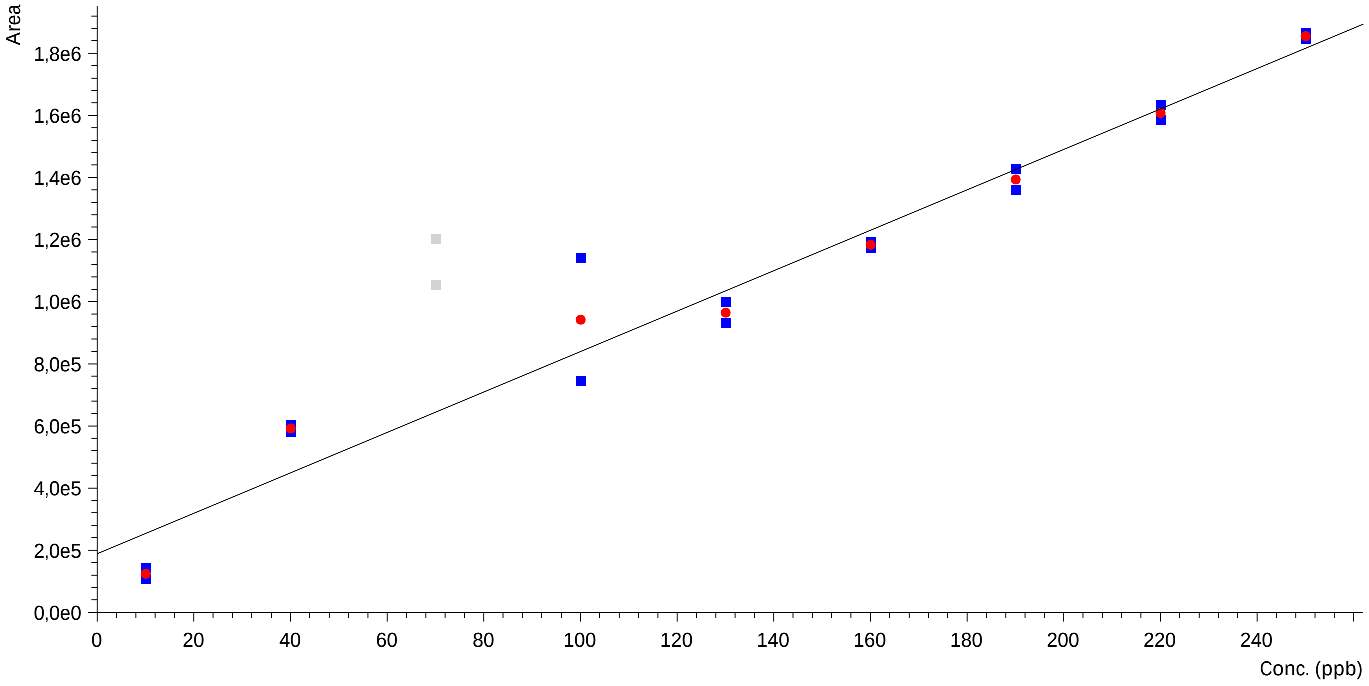

Sample ID	Area	Conc.	Sample Type	Level	Cal Point	Found RT	Ref 1 Actual Ratio
0	10030	-27,4149	Ctrl.	0	----	9,947	5,20
10ppb001	107610	-12,4221	Std.	1	Yes	9,895	25,57
10 ppb002	142371	-7,0811	Std.	1	Yes	9,896	30,71
40 ppb001	580923	60,3010	Std.	2	Yes	9,887	33,68
40ppb002	603059	63,7021	Std.	2	Yes	9,897	25,15
70 ppb001	1201056	155,5825	Std.	3	No	9,891	30,47
70ppb002	1054422	133,0527	Std.	3	No	9,901	32,20
100 ppb001	1141057	146,3640	Std.	4	Yes	9,897	29,66
100ppb002	743426	85,2691	Std.	4	Yes	9,891	27,15
130 ppb001	930847	114,0657	Std.	5	Yes	9,890	30,18
130ppb002	1000075	124,7024	Std.	5	Yes	9,895	23,12
160 ppb001	1174151	151,4487	Std.	6	Yes	9,895	29,92
160ppb002	1192960	154,3386	Std.	6	Yes	9,893	27,51
190 ppb001	1360625	180,0998	Std.	7	Yes	9,897	27,79
190ppb002	1427833	190,4261	Std.	7	Yes	9,888	27,38
220 ppb001	1584333	214,4719	Std.	8	Yes	9,891	26,12
220ppb002	1632551	221,8805	Std.	8	Yes	9,900	28,07
250 ppb001	1864616	257,5365	Std.	9	Yes	9,885	28,51
250 ppb002	1847435	254,8968	Std.	9	Yes	9,887	29,77
10H	----	----	Unk.	----	----	----	----
10M	----	----	Unk.	----	----	----	----
9H	----	----	Unk.	----	----	----	----
9M	----	----	Unk.	----	----	----	----
Average	1031546	129,5379				9,896	27,27
%RSD	54	65,8507				0,133	21,70
Maximum	1864616	257,5365				9,947	33,68
Minimum	10030	-27,4149				9,885	5,20
Std. Dev.	555179	85,3016				0,013	5,92

Compound: Luteolin (continued)

0
Conc -27,4149
Area 10030
R#1 285,00>150,95 5,20 (27,51)
R#2 285,00>175,10 0,00 (17,88)
Q 285,00>133,00 (-) 1,87e3

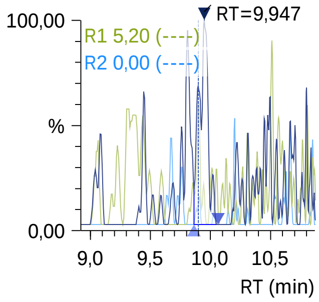

10ppb001
Conc -12,4221
Area 107610
R#1 285,00>150,95 25,57 (27,51)
R#2 285,00>175,10 19,67 (17,88)
Q 285,00>133,00 (-) 1,98e4

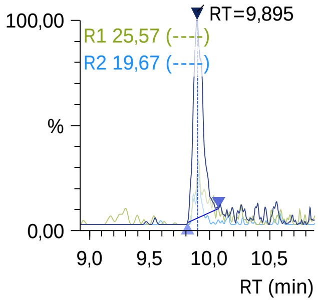

10 ppb002
Conc -7,0811
Area 142371
R#1 285,00>150,95 30,71 (27,51)
R#2 285,00>175,10 20,73 (17,88)
Q 285,00>133,00 (-) 2,71e4

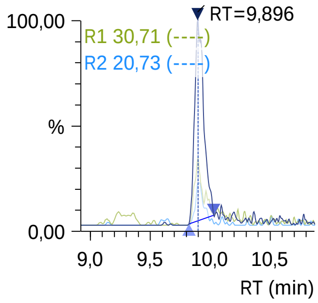

40 ppb001
Conc 60,3010
Area 580923
R#1 285,00>150,95 33,68 (27,51)
R#2 285,00>175,10 17,93 (17,88)
Q 285,00>133,00 (-) 1,16e5

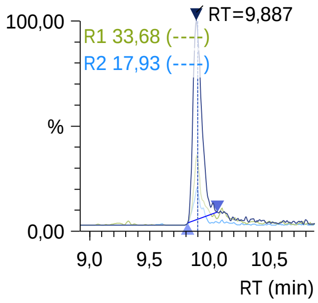

40ppb002
Conc 63,7021
Area 603059
R#1 285,00>150,95 25,15 (27,51)
R#2 285,00>175,10 16,88 (17,88)
Q 285,00>133,00 (-) 1,36e5

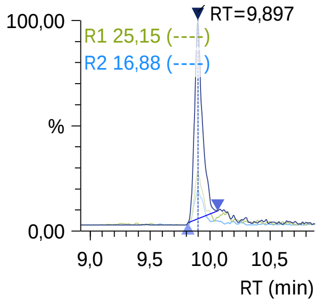

70 ppb001
Conc 155,5825
Area 1201056
R#1 285,00>150,95 30,47 (27,51)
R#2 285,00>175,10 20,89 (17,88)
Q 285,00>133,00 (-) 2,46e5

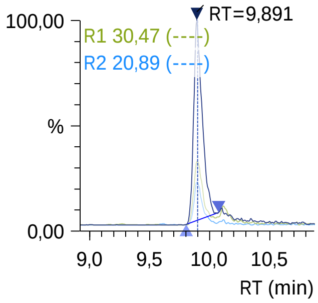

70ppb002
Conc 133,0527
Area 1054422
R#1 285,00>150,95 32,20 (27,51)
R#2 285,00>175,10 18,10 (17,88)
Q 285,00>133,00 (-) 2,16e5

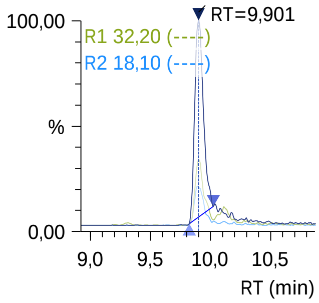

100 ppb001
Conc 146,3640
Area 1141057
R#1 285,00>150,95 29,66 (27,51)
R#2 285,00>175,10 19,63 (17,88)
Q 285,00>133,00 (-) 2,29e5

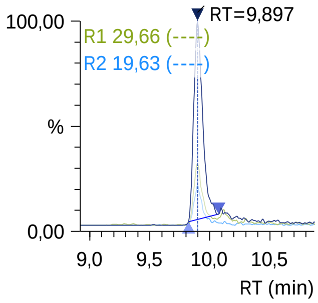

100ppb002
Conc 85,2691
Area 743426
R#1 285,00>150,95 27,15 (27,51)
R#2 285,00>175,10 18,19 (17,88)
Q 285,00>133,00 (-) 1,57e5

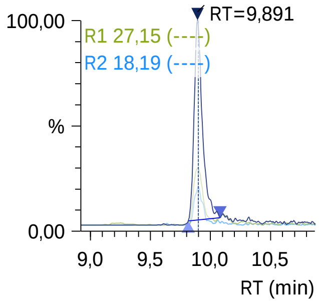

130 ppb001
Conc 114,0657
Area 930847
R#1 285,00>150,95 30,18 (27,51)
R#2 285,00>175,10 19,50 (17,88)
Q 285,00>133,00 (-) 1,97e5

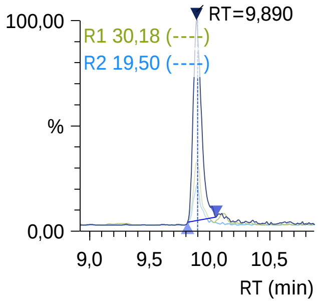

130ppb002
Conc 124,7024
Area 1000075
R#1 285,00>150,95 23,12 (27,51)
R#2 285,00>175,10 18,22 (17,88)
Q 285,00>133,00 (-) 2,05e5

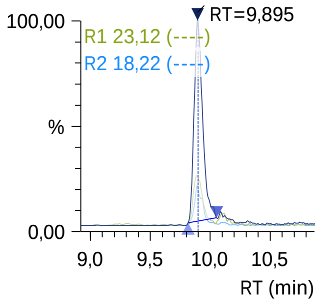

160 ppb001
Conc 151,4487
Area 1174151
R#1 285,00>150,95 29,92 (27,51)
R#2 285,00>175,10 18,05 (17,88)
Q 285,00>133,00 (-) 2,45e5

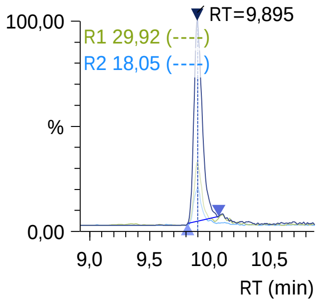

Compound: Luteolin (continued)

160ppb002
Conc 154,3386
Area 1192960
R#1 285,00>150,95 27,51 (27,51)
R#2 285,00>175,10 17,88 (17,88)
Q 285,00>133,00 (-) 2,47e5

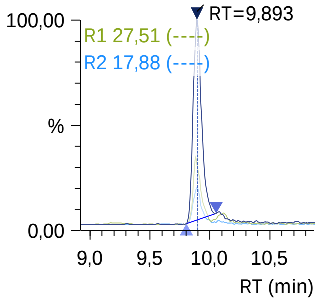

190 ppb001
Conc 180,0998
Area 1360625
R#1 285,00>150,95 27,79 (27,51)
R#2 285,00>175,10 19,59 (17,88)
Q 285,00>133,00 (-) 2,84e5

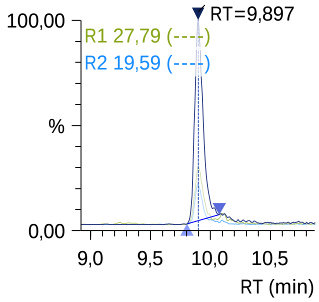

190ppb002
Conc 190,4261
Area 1427833
R#1 285,00>150,95 27,38 (27,51)
R#2 285,00>175,10 17,15 (17,88)
Q 285,00>133,00 (-) 2,97e5

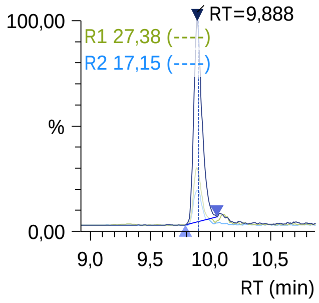

220 ppb001
Conc 214,4719
Area 1584333
R#1 285,00>150,95 26,12 (27,51)
R#2 285,00>175,10 17,92 (17,88)
Q 285,00>133,00 (-) 3,39e5

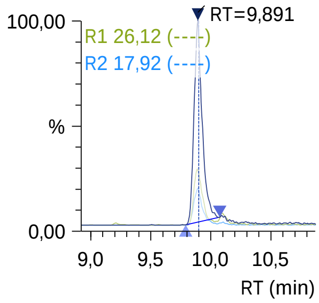

220ppb002
Conc 221,8805
Area 1632551
R#1 285,00>150,95 28,07 (27,51)
R#2 285,00>175,10 19,51 (17,88)
Q 285,00>133,00 (-) 3,34e5

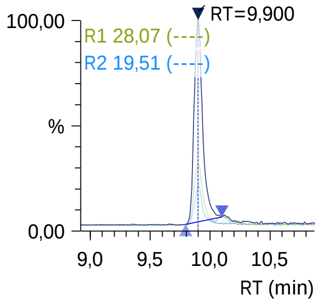

250 ppb001
Conc 257,5365
Area 1864616
R#1 285,00>150,95 28,51 (27,51)
R#2 285,00>175,10 21,19 (17,88)
Q 285,00>133,00 (-) 3,84e5

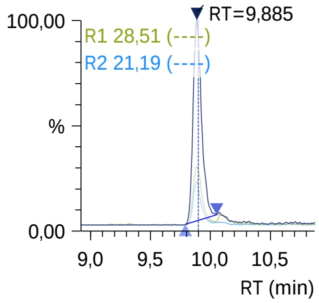

250 ppb002
Conc 254,8968
Area 1847435
R#1 285,00>150,95 29,77 (27,51)
R#2 285,00>175,10 18,56 (17,88)
Q 285,00>133,00 (-) 3,90e5

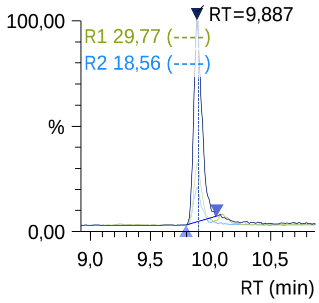

10H
Conc ----
Area ----
R#1 285,00>150,95 ---- (27,51)
R#2 285,00>175,10 ---- (17,88)
Q 285,00>133,00 (-) 1,05e3

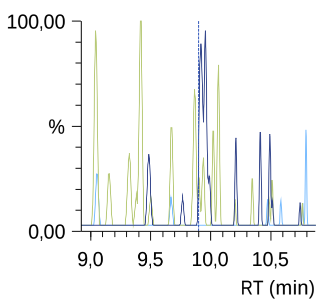

10M
Conc ----
Area ----
R#1 285,00>150,95 ---- (27,51)
R#2 285,00>175,10 ---- (17,88)
Q 285,00>133,00 (-) 2,07e3

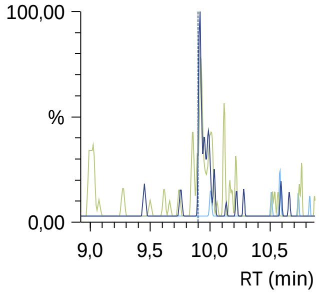

9H
Conc ----
Area ----
R#1 285,00>150,95 ---- (27,51)
R#2 285,00>175,10 ---- (17,88)
Q 285,00>133,00 (-) 1,07e3

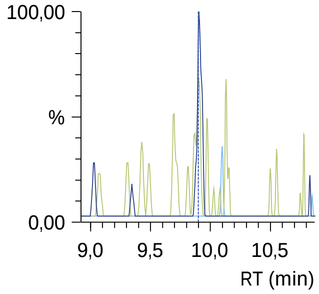

9M
Conc ----
Area ----
R#1 285,00>150,95 ---- (27,51)
R#2 285,00>175,10 ---- (17,88)
Q 285,00>133,00 (-) 1,50e3

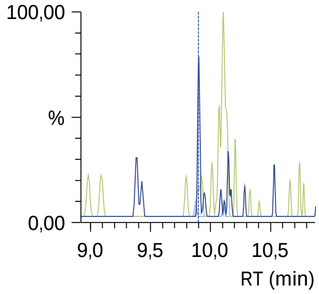

Compound: Rutina

Curve Fit: Linear | Weighting: None | Zero: Not Forced
Quantitative Method: External Standard
Q 609,00>300,00
R^2 = 0.9985252 R = 0.9992623
y = 4219,125x - 8297,430

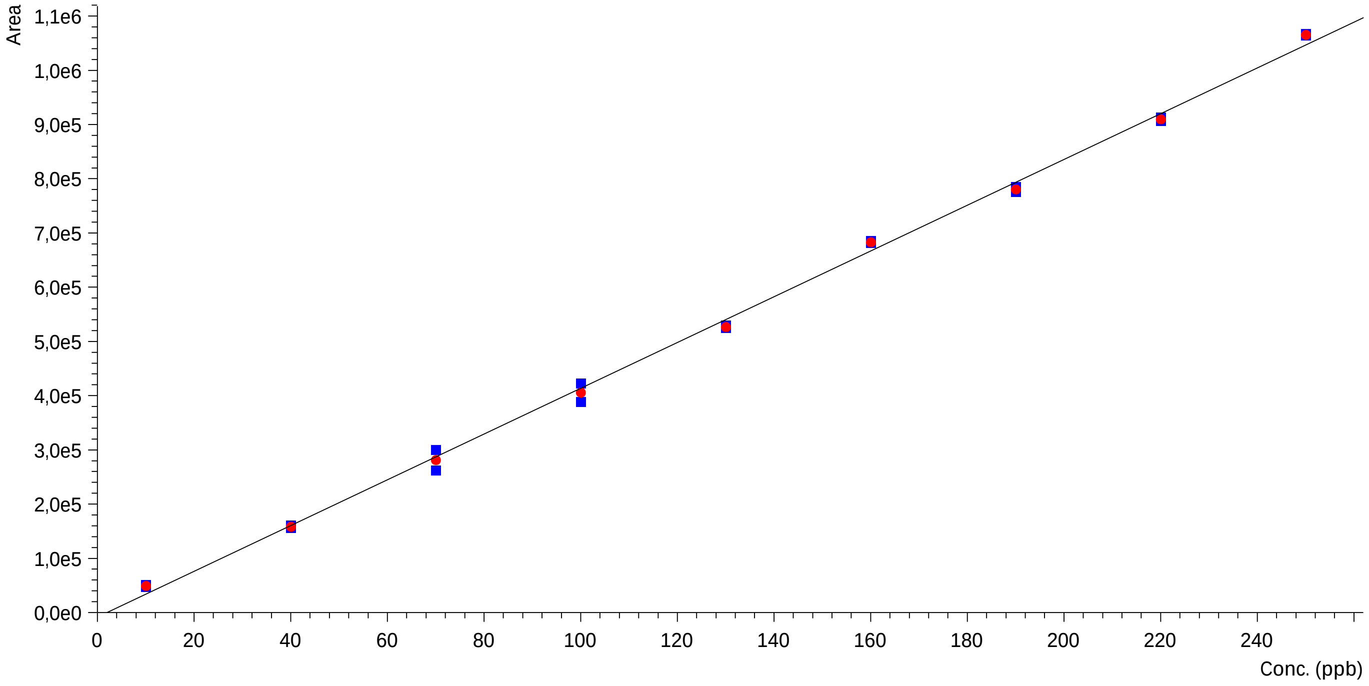

Sample ID	Area	Conc.	Sample Type	Level	Cal Point	Found RT	Ref 1 Actual Ratio
0	----	----	Ctrl.	0	----	----	----
10ppb001	47418	13,2054	Std.	1	Yes	8,929	40,08
10 ppb002	51560	14,1873	Std.	1	Yes	8,933	36,20
40 ppb001	156186	38,9852	Std.	2	Yes	8,928	45,24
40ppb002	160737	40,0639	Std.	2	Yes	8,931	57,35
70 ppb001	300603	73,2143	Std.	3	Yes	8,936	48,88
70ppb002	262712	64,2336	Std.	3	Yes	8,934	58,83
100 ppb001	422597	102,1290	Std.	4	Yes	8,929	57,62
100ppb002	389112	94,1925	Std.	4	Yes	8,935	47,93
130 ppb001	529310	127,4216	Std.	5	Yes	8,930	45,45
130ppb002	524484	126,2776	Std.	5	Yes	8,931	53,78
160 ppb001	681398	163,4688	Std.	6	Yes	8,937	48,88
160ppb002	684957	164,3124	Std.	6	Yes	8,928	49,88
190 ppb001	775885	185,8637	Std.	7	Yes	8,941	48,27
190ppb002	785113	188,0510	Std.	7	Yes	8,926	57,40
220 ppb001	906790	216,8904	Std.	8	Yes	8,932	49,28
220ppb002	912993	218,3606	Std.	8	Yes	8,936	52,99
250 ppb001	1067339	254,9430	Std.	9	Yes	8,927	48,28
250 ppb002	1064204	254,1999	Std.	9	Yes	8,933	53,63
10H	12735	4,9849	Unk.	----	----	8,963	28,01
10M	35787	10,4487	Unk.	----	----	8,948	31,14
9H	15773	5,7050	Unk.	----	----	8,961	40,96
9M	38127	11,0033	Unk.	----	----	8,947	53,32
Average	446628	107,8246				8,936	47,88
%RSD	82	80,1775				0,114	17,41
Maximum	1067339	254,9430				8,963	58,83
Minimum	12735	4,9849				8,926	28,01
Std. Dev.	364748	86,4510				0,010	8,34

Compound: Rutina (continued)

0
Conc ----
Area ----
R#1 609,00>271,05 ---- (45,45)
R#2 609,00>301,00 ---- (40,29)
Q 609,00>300,00 (-) 7,78e2

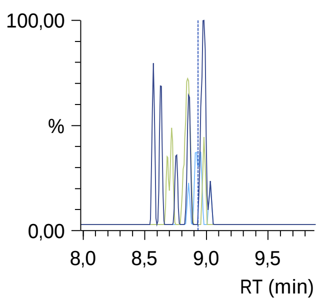

10ppb001
Conc 13,2054
Area 47418
R#1 609,00>271,05 40,08 (45,45)
R#2 609,00>301,00 26,52 (40,29)
Q 609,00>300,00 (-) 1,24e4

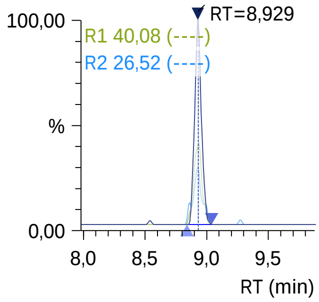

10 ppb002
Conc 14,1873
Area 51560
R#1 609,00>271,05 36,20 (45,45)
R#2 609,00>301,00 27,16 (40,29)
Q 609,00>300,00 (-) 1,09e4

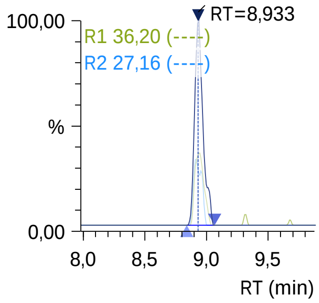

40 ppb001
Conc 38,9852
Area 156186
R#1 609,00>271,05 45,24 (45,45)
R#2 609,00>301,00 35,33 (40,29)
Q 609,00>300,00 (-) 3,70e4

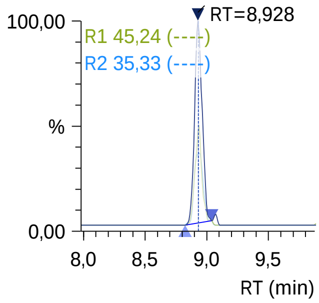

40ppb002
Conc 40,0639
Area 160737
R#1 609,00>271,05 57,35 (45,45)
R#2 609,00>301,00 36,13 (40,29)
Q 609,00>300,00 (-) 3,94e4

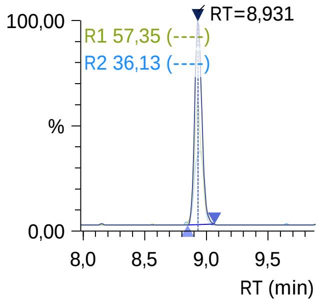

70 ppb001
Conc 73,2143
Area 300603
R#1 609,00>271,05 48,88 (45,45)
R#2 609,00>301,00 45,31 (40,29)
Q 609,00>300,00 (-) 6,76e4

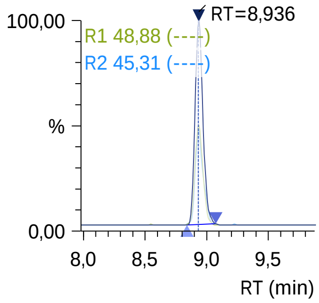

70ppb002
Conc 64,2336
Area 262712
R#1 609,00>271,05 58,83 (45,45)
R#2 609,00>301,00 48,54 (40,29)
Q 609,00>300,00 (-) 5,77e4

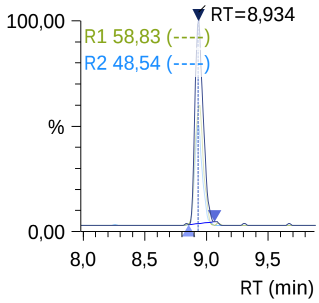

100 ppb001
Conc 102,1290
Area 422597
R#1 609,00>271,05 57,62 (45,45)
R#2 609,00>301,00 40,82 (40,29)
Q 609,00>300,00 (-) 9,58e4

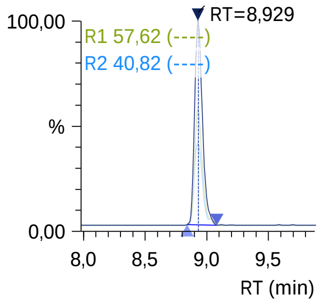

100ppb002
Conc 94,1925
Area 389112
R#1 609,00>271,05 47,93 (45,45)
R#2 609,00>301,00 43,52 (40,29)
Q 609,00>300,00 (-) 8,80e4

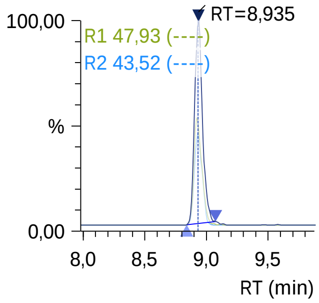

130 ppb001
Conc 127,4216
Area 529310
R#1 609,00>271,05 45,45 (45,45)
R#2 609,00>301,00 40,29 (40,29)
Q 609,00>300,00 (-) 1,17e5

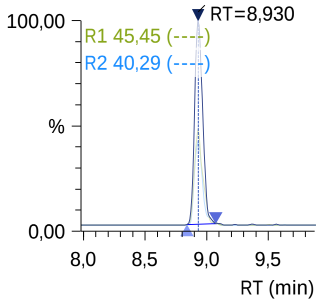

130ppb002
Conc 126,2776
Area 524484
R#1 609,00>271,05 53,78 (45,45)
R#2 609,00>301,00 42,95 (40,29)
Q 609,00>300,00 (-) 1,08e5

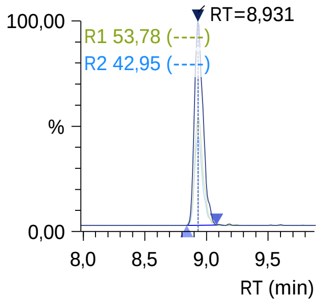

160 ppb001
Conc 163,4688
Area 681398
R#1 609,00>271,05 48,88 (45,45)
R#2 609,00>301,00 38,01 (40,29)
Q 609,00>300,00 (-) 1,57e5

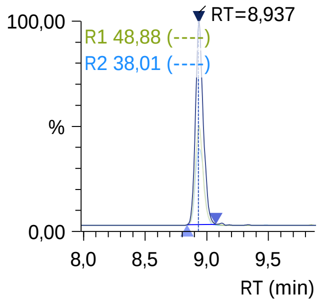

Compound: Rutina (continued)

160ppb002
Conc 164,3124
Area 684957
R#1 609,00>271,05 49,88 (45,45)
R#2 609,00>301,00 42,91 (40,29)
Q 609,00>300,00 (-) 1,49e5

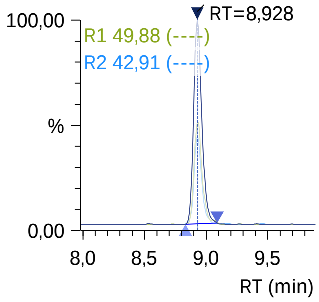

190 ppb001
Conc 185,8637
Area 775885
R#1 609,00>271,05 48,27 (45,45)
R#2 609,00>301,00 43,87 (40,29)
Q 609,00>300,00 (-) 1,70e5

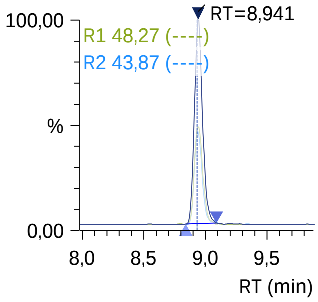

190ppb002
Conc 188,0510
Area 785113
R#1 609,00>271,05 57,40 (45,45)
R#2 609,00>301,00 44,22 (40,29)
Q 609,00>300,00 (-) 1,67e5

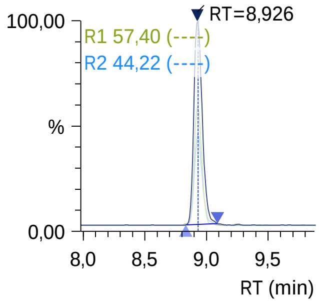

220 ppb001
Conc 216,8904
Area 906790
R#1 609,00>271,05 49,28 (45,45)
R#2 609,00>301,00 42,58 (40,29)
Q 609,00>300,00 (-) 2,04e5

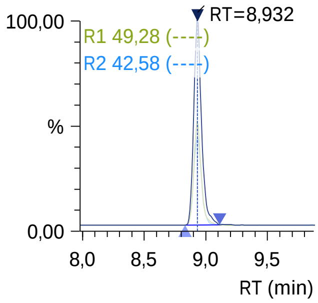

220ppb002
Conc 218,3606
Area 912993
R#1 609,00>271,05 52,99 (45,45)
R#2 609,00>301,00 41,26 (40,29)
Q 609,00>300,00 (-) 2,04e5

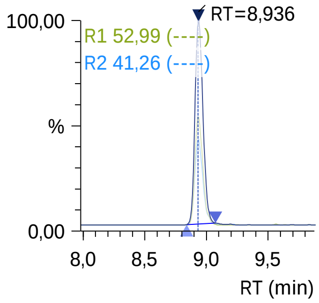

250 ppb001
Conc 254,9430
Area 1067339
R#1 609,00>271,05 48,28 (45,45)
R#2 609,00>301,00 40,82 (40,29)
Q 609,00>300,00 (-) 2,38e5

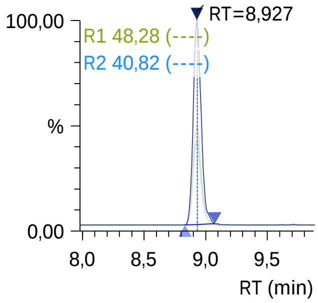

250 ppb002
Conc 254,1999
Area 1064204
R#1 609,00>271,05 53,63 (45,45)
R#2 609,00>301,00 43,78 (40,29)
Q 609,00>300,00 (-) 2,39e5

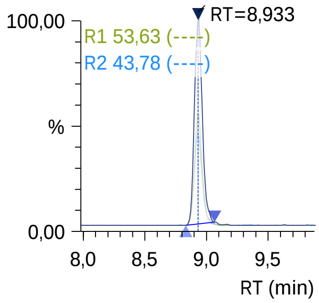

10H
Conc 4,9849
Area 12735
R#1 609,00>271,05 28,01 (45,45)
R#2 609,00>301,00 27,97 (40,29)
Q 609,00>300,00 (-) 2,38e3

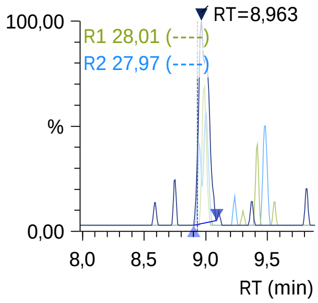

10M
Conc 10,4487
Area 35787
R#1 609,00>271,05 31,14 (45,45)
R#2 609,00>301,00 35,94 (40,29)
Q 609,00>300,00 (-) 6,95e3

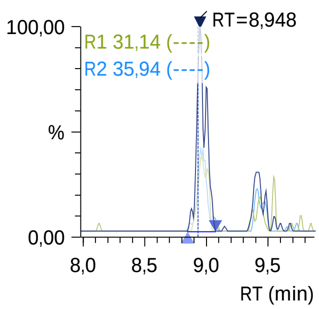

9H
Conc 5,7050
Area 15773
R#1 609,00>271,05 40,96 (45,45)
R#2 609,00>301,00 18,11 (40,29)
Q 609,00>300,00 (-) 3,15e3

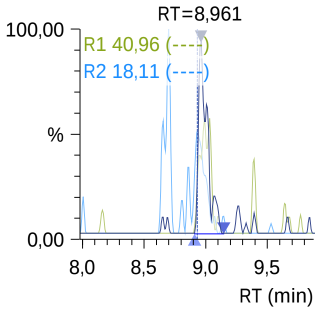

9M
Conc 11,0033
Area 38127
R#1 609,00>271,05 53,32 (45,45)
R#2 609,00>301,00 33,29 (40,29)
Q 609,00>300,00 (-) 8,67e3

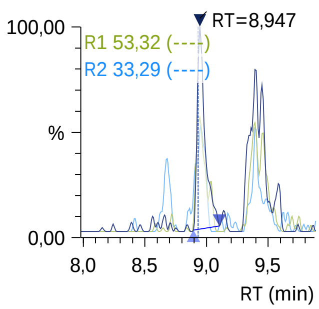

Compound: Epicatequina

Curve Fit: Linear | Weighting: None | Zero: Not Forced
Quantitative Method: External Standard
Q 289,10>245,10
R^2 = 0.9807770 R = 0.9903419
y = 1146,058x - 12562,15

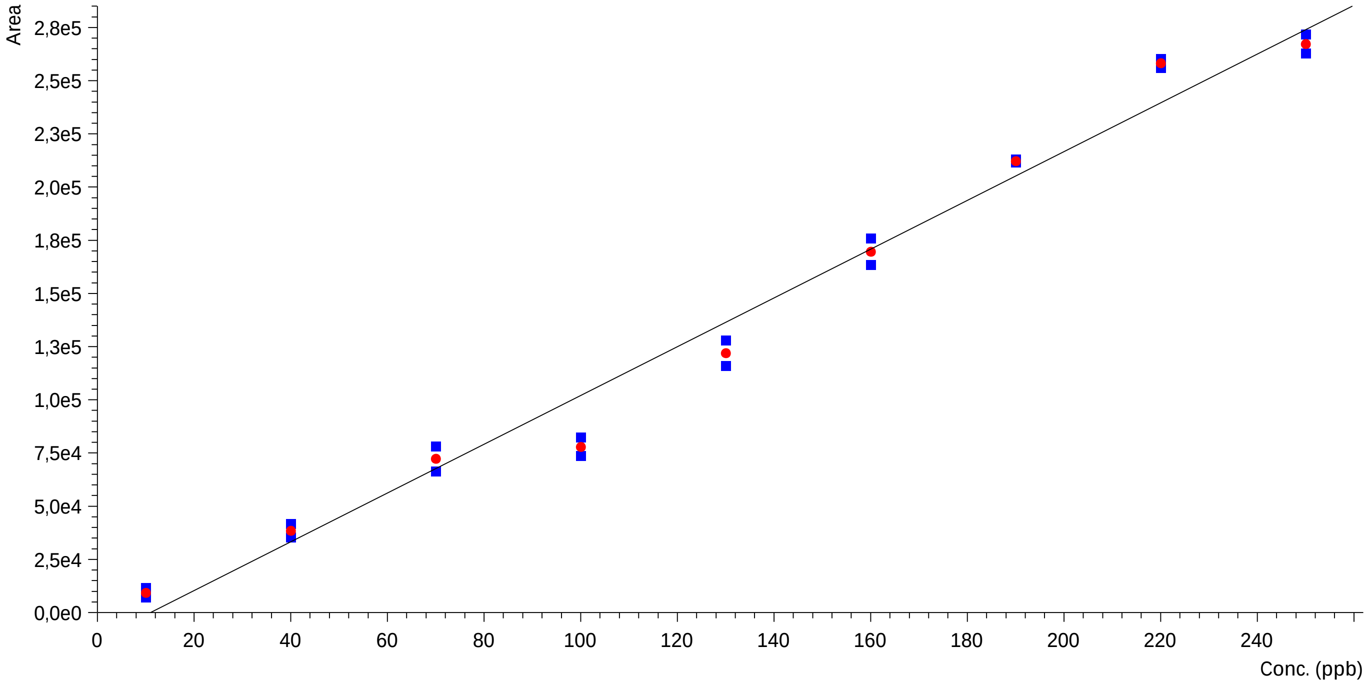

Sample ID	Area	Conc.	Sample Type	Level	Cal Point	Found RT	Ref 1 Actual Ratio
0	----	----	Ctrl.	0	----	----	----
10ppb001	7093	17,1504	Std.	1	Yes	7,637	88,95
10 ppb002	11630	21,1087	Std.	1	Yes	7,664	25,33
40 ppb001	41732	47,3744	Std.	2	Yes	7,631	35,48
40ppb002	35323	41,7826	Std.	2	Yes	7,648	37,35
70 ppb001	78112	79,1186	Std.	3	Yes	7,649	55,12
70ppb002	66498	68,9845	Std.	3	Yes	7,647	62,64
100 ppb001	73628	75,2054	Std.	4	Yes	7,654	69,65
100ppb002	82312	82,7833	Std.	4	Yes	7,662	49,86
130 ppb001	128010	122,6568	Std.	5	Yes	7,654	47,48
130ppb002	115986	112,1652	Std.	5	Yes	7,647	50,94
160 ppb001	163540	153,6590	Std.	6	Yes	7,651	51,52
160ppb002	175969	164,5043	Std.	6	Yes	7,643	47,42
190 ppb001	211634	195,6235	Std.	7	Yes	7,656	54,85
190ppb002	213026	196,8388	Std.	7	Yes	7,645	47,58
220 ppb001	260367	238,1458	Std.	8	Yes	7,647	50,28
220ppb002	256094	234,4178	Std.	8	Yes	7,649	44,68
250 ppb001	271827	248,1459	Std.	9	Yes	7,633	51,30
250 ppb002	262876	240,3350	Std.	9	Yes	7,649	43,16
10H	----	----	Unk.	----	----	----	----
10M	----	----	Unk.	----	----	----	----
9H	----	----	Unk.	----	----	----	----
9M	----	----	Unk.	----	----	----	----
Average	136425	130,0000				7,648	50,75
%RSD	68	61,9815				0,115	26,95
Maximum	271827	248,1459				7,664	88,95
Minimum	7093	17,1504				7,631	25,33
Std. Dev.	92345	80,5760				0,009	13,68

Compound: Epicatequina (continued)

0
Conc ----
Area ----
R#1 289,10>108,95 ---- (55,12)
R#2 289,10>203,05 ---- (38,42)
Q 289,10>245,10 (-) 1,45e3

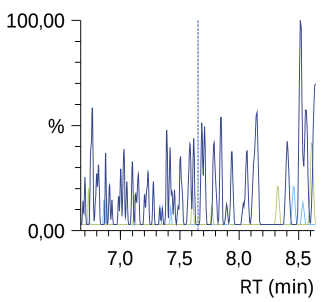

10ppb001
Conc 17,1504
Area 7093
R#1 289,10>108,95 88,95 (55,12)
R#2 289,10>203,05 26,47 (38,42)
Q 289,10>245,10 (-) 3,79e3

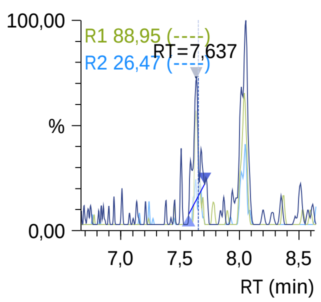

10 ppb002
Conc 21,1087
Area 11630
R#1 289,10>108,95 25,33 (55,12)
R#2 289,10>203,05 54,81 (38,42)
Q 289,10>245,10 (-) 2,78e3

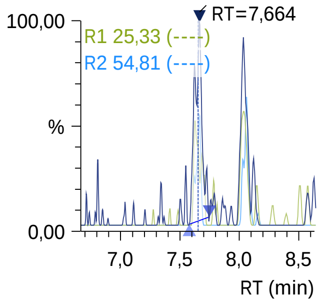

40 ppb001
Conc 47,3744
Area 41732
R#1 289,10>108,95 35,48 (55,12)
R#2 289,10>203,05 27,63 (38,42)
Q 289,10>245,10 (-) 1,11e4

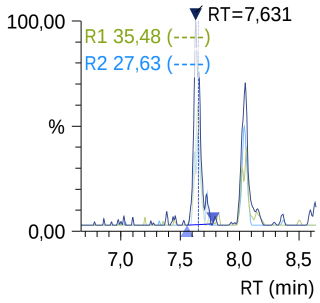

40ppb002
Conc 41,7826
Area 35323
R#1 289,10>108,95 37,35 (55,12)
R#2 289,10>203,05 39,53 (38,42)
Q 289,10>245,10 (-) 1,53e4

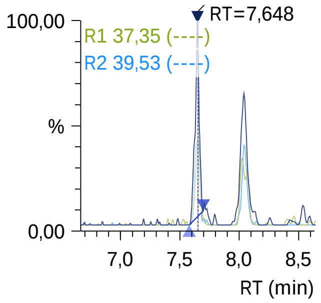

70 ppb001
Conc 79,1186
Area 78112
R#1 289,10>108,95 55,12 (55,12)
R#2 289,10>203,05 38,42 (38,42)
Q 289,10>245,10 (-) 2,14e4

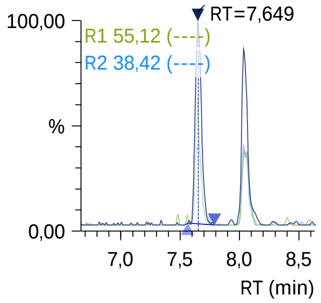

70ppb002
Conc 68,9845
Area 66498
R#1 289,10>108,95 62,64 (55,12)
R#2 289,10>203,05 58,46 (38,42)
Q 289,10>245,10 (-) 1,82e4

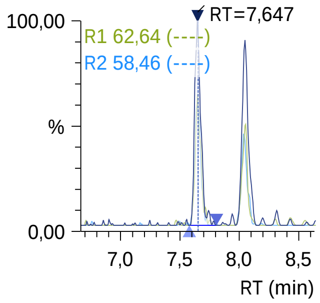

100 ppb001
Conc 75,2054
Area 73628
R#1 289,10>108,95 69,65 (55,12)
R#2 289,10>203,05 42,75 (38,42)
Q 289,10>245,10 (-) 2,76e4

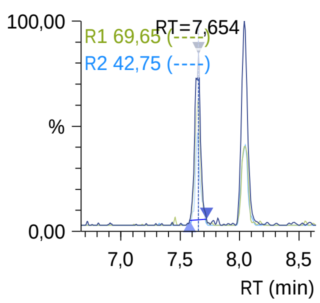

100ppb002
Conc 82,7833
Area 82312
R#1 289,10>108,95 49,86 (55,12)
R#2 289,10>203,05 54,93 (38,42)
Q 289,10>245,10 (-) 2,74e4

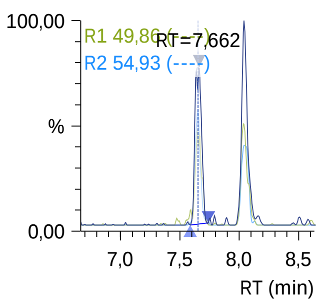

130 ppb001
Conc 122,6568
Area 128010
R#1 289,10>108,95 47,48 (55,12)
R#2 289,10>203,05 41,40 (38,42)
Q 289,10>245,10 (-) 3,65e4

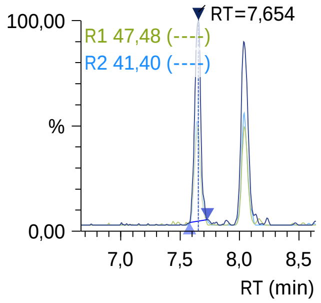

130ppb002
Conc 112,1652
Area 115986
R#1 289,10>108,95 50,94 (55,12)
R#2 289,10>203,05 57,73 (38,42)
Q 289,10>245,10 (-) 3,60e4

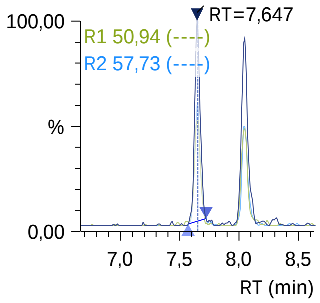

160 ppb001
Conc 153,6590
Area 163540
R#1 289,10>108,95 51,52 (55,12)
R#2 289,10>203,05 52,02 (38,42)
Q 289,10>245,10 (-) 4,88e4

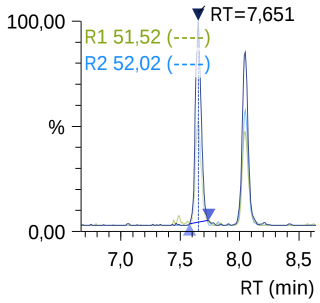

Compound: Epicatequina (continued)

160ppb002
Conc 164,5043
Area 175969
R#1 289,10>108,95 47,42 (55,12)
R#2 289,10>203,05 55,99 (38,42)
Q 289,10>245,10 (-) 4,73e4

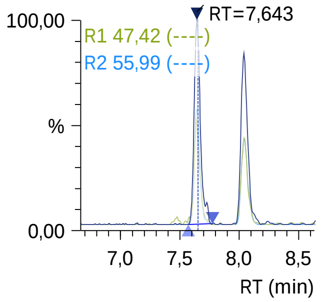

190 ppb001
Conc 195,6235
Area 211634
R#1 289,10>108,95 54,85 (55,12)
R#2 289,10>203,05 44,42 (38,42)
Q 289,10>245,10 (-) 6,42e4

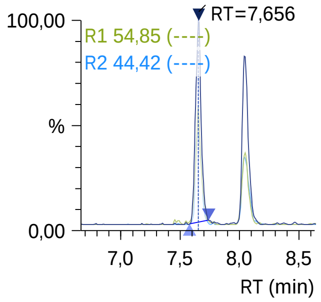

190ppb002
Conc 196,8388
Area 213026
R#1 289,10>108,95 47,58 (55,12)
R#2 289,10>203,05 49,79 (38,42)
Q 289,10>245,10 (-) 6,76e4

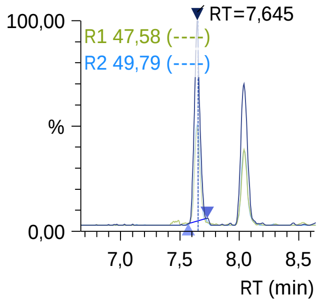

220 ppb001
Conc 238,1458
Area 260367
R#1 289,10>108,95 50,28 (55,12)
R#2 289,10>203,05 44,03 (38,42)
Q 289,10>245,10 (-) 7,43e4

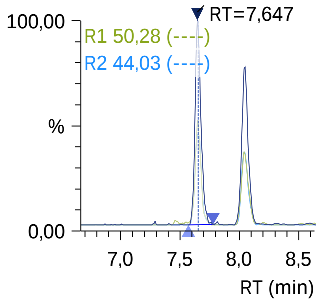

220ppb002
Conc 234,4178
Area 256094
R#1 289,10>108,95 44,68 (55,12)
R#2 289,10>203,05 48,33 (38,42)
Q 289,10>245,10 (-) 7,05e4

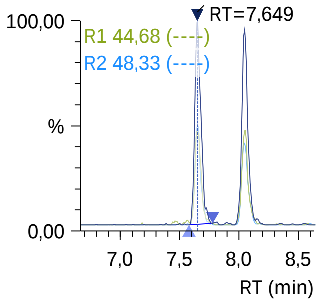

250 ppb001
Conc 248,1459
Area 271827
R#1 289,10>108,95 51,30 (55,12)
R#2 289,10>203,05 51,35 (38,42)
Q 289,10>245,10 (-) 7,65e4

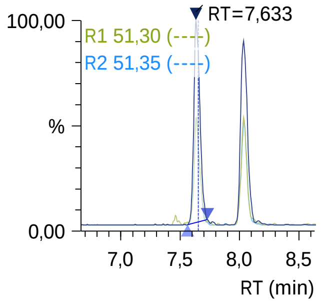

250 ppb002
Conc 240,3350
Area 262876
R#1 289,10>108,95 43,16 (55,12)
R#2 289,10>203,05 54,10 (38,42)
Q 289,10>245,10 (-) 7,75e4

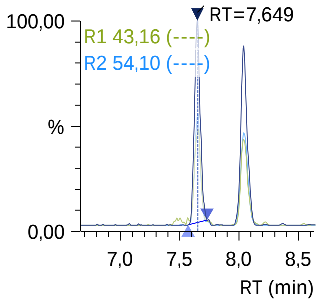

10H
Conc ----
Area ----
R#1 289,10>108,95 ---- (55,12)
R#2 289,10>203,05 ---- (38,42)
Q 289,10>245,10 (-) 3,98e3

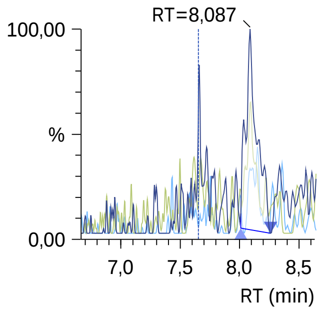

10M
Conc ----
Area ----
R#1 289,10>108,95 ---- (55,12)
R#2 289,10>203,05 ---- (38,42)
Q 289,10>245,10 (-) 4,76e3

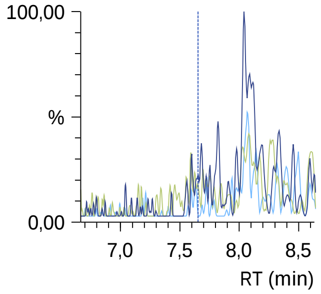

9H
Conc ----
Area ----
R#1 289,10>108,95 ---- (55,12)
R#2 289,10>203,05 ---- (38,42)
Q 289,10>245,10 (-) 4,20e3

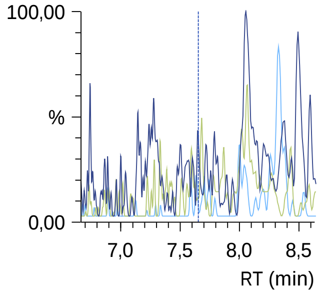

9M
Conc ----
Area ----
R#1 289,10>108,95 ---- (55,12)
R#2 289,10>203,05 ---- (38,42)
Q 289,10>245,10 (-) 3,72e3

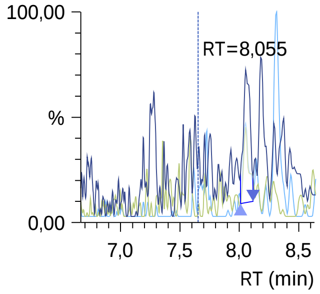

Compound: Baicalin

Curve Fit: Linear | Weighting: None | Zero: Not Forced
Quantitative Method: External Standard
Q 445,20>269,10
R^2 = 0.9976376 R = 0.9988181
y = 4376,237x + 11508,54

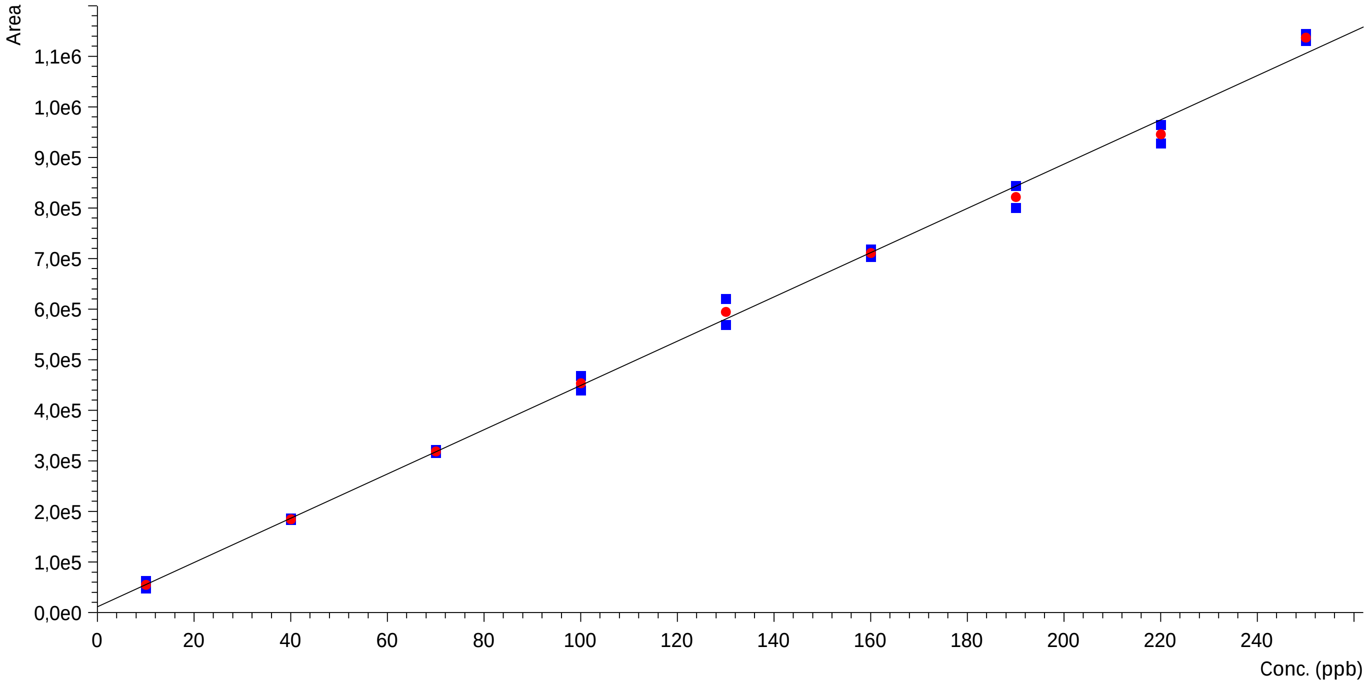

Sample ID	Area	Conc.	Sample Type	Level	Cal Point	Found RT	Ref 1 Actual Ratio
0	----	----	Ctrl.	0	----	----	----
10ppb001	62558	11,6651	Std.	1	Yes	9,655	2,30
10 ppb002	47446	8,2120	Std.	1	Yes	9,711	9,74
40 ppb001	183255	39,2452	Std.	2	Yes	9,682	11,60
40ppb002	186285	39,9377	Std.	2	Yes	9,686	7,85
70 ppb001	322165	70,9871	Std.	3	Yes	9,681	10,17
70ppb002	315838	69,5414	Std.	3	Yes	9,684	9,35
100 ppb001	467643	104,2299	Std.	4	Yes	9,681	11,06
100ppb002	439478	97,7939	Std.	4	Yes	9,684	9,92
130 ppb001	620724	139,2098	Std.	5	Yes	9,672	10,13
130ppb002	568705	127,3233	Std.	5	Yes	9,680	10,16
160 ppb001	718515	161,5559	Std.	6	Yes	9,677	10,22
160ppb002	703629	158,1544	Std.	6	Yes	9,675	10,30
190 ppb001	843425	190,0986	Std.	7	Yes	9,683	7,81
190ppb002	800858	180,3717	Std.	7	Yes	9,668	11,52
220 ppb001	928059	209,4380	Std.	8	Yes	9,673	8,54
220ppb002	964526	217,7710	Std.	8	Yes	9,680	8,87
250 ppb001	1130045	255,5933	Std.	9	Yes	9,669	10,64
250 ppb002	1144392	258,8717	Std.	9	Yes	9,673	9,00
10H	----	----	Unk.	----	----	----	----
10M	----	----	Unk.	----	----	----	----
9H	----	----	Unk.	----	----	----	----
9M	----	----	Unk.	----	----	----	----
Average	580419	130,0000				9,679	9,40
%RSD	60	61,4387				0,114	22,16
Maximum	1144392	258,8717				9,711	11,60
Minimum	47446	8,2120				9,655	2,30
Std. Dev.	349531	79,8703				0,011	2,08

Compound: Baicalin (continued)

0

Conc ----
Area ----
R#1 445,20>112,90 ---- (11,06)
R#2 445,20>175,10 ---- (6,52)
Q 445,20>269,10 (-)   1,69e3

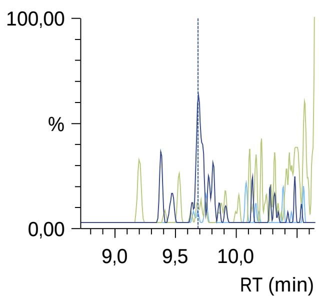

10ppb001

Conc 11,6651
Area 62558
R#1 445,20>112,90 2,30 (11,06)
R#2 445,20>175,10 2,74 (6,52)
Q 445,20>269,10 (-)   9,19e3

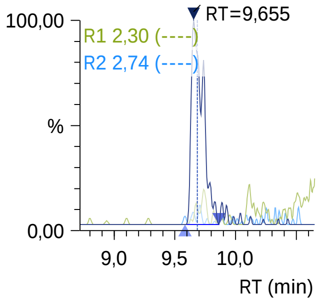

10 ppb002

Conc 8,2120
Area 47446
R#1 445,20>112,90 9,74 (11,06)
R#2 445,20>175,10 7,08 (6,52)
Q 445,20>269,10 (-)   8,06e3

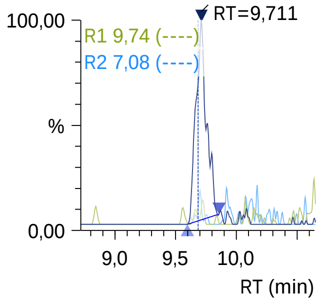

40 ppb001

Conc 39,2452
Area 183255
R#1 445,20>112,90 11,60 (11,06)
R#2 445,20>175,10 10,21 (6,52)
Q 445,20>269,10 (-)   3,46e4

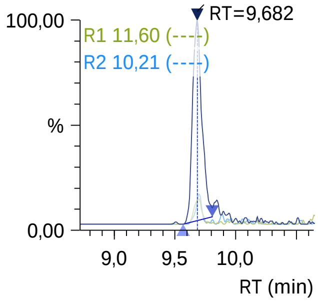

40ppb002

Conc 39,9377
Area 186285
R#1 445,20>112,90 7,85 (11,06)
R#2 445,20>175,10 7,15 (6,52)
Q 445,20>269,10 (-)   3,27e4

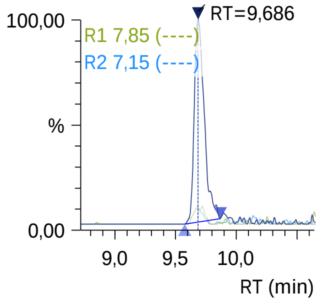

70 ppb001

Conc 70,9871
Area 322165
R#1 445,20>112,90 10,17 (11,06)
R#2 445,20>175,10 6,69 (6,52)
Q 445,20>269,10 (-)   5,36e4

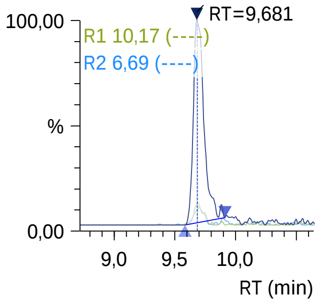

70ppb002

Conc 69,5414
Area 315838
R#1 445,20>112,90 9,35 (11,06)
R#2 445,20>175,10 6,72 (6,52)
Q 445,20>269,10 (-)   6,19e4

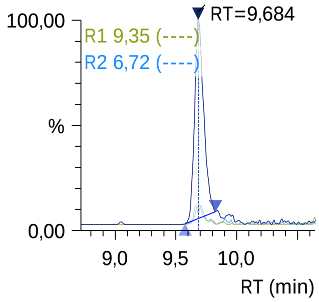

100 ppb001

Conc 104,2299
Area 467643
R#1 445,20>112,90 11,06 (11,06)
R#2 445,20>175,10 6,52 (6,52)
Q 445,20>269,10 (-)   9,35e4

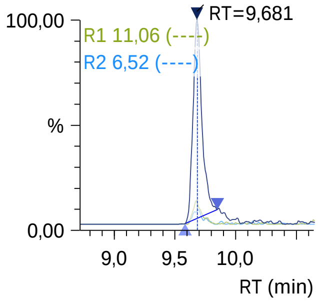

100ppb002

Conc 97,7939
Area 439478
R#1 445,20>112,90 9,92 (11,06)
R#2 445,20>175,10 6,37 (6,52)
Q 445,20>269,10 (-)   7,55e4

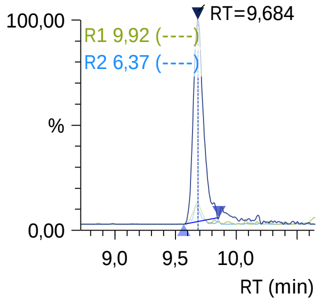

130 ppb001

Conc 139,2098
Area 620724
R#1 445,20>112,90 10,13 (11,06)
R#2 445,20>175,10 4,01 (6,52)
Q 445,20>269,10 (-)   1,06e5

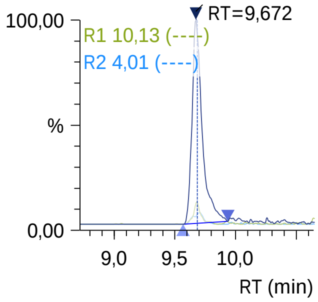

130ppb002

Conc 127,3233
Area 568705
R#1 445,20>112,90 10,16 (11,06)
R#2 445,20>175,10 8,59 (6,52)
Q 445,20>269,10 (-)   9,19e4

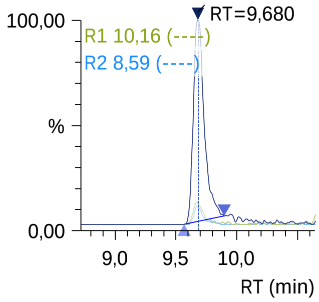

160 ppb001

Conc 161,5559
Area 718515
R#1 445,20>112,90 10,22 (11,06)
R#2 445,20>175,10 9,16 (6,52)
Q 445,20>269,10 (-)   1,21e5

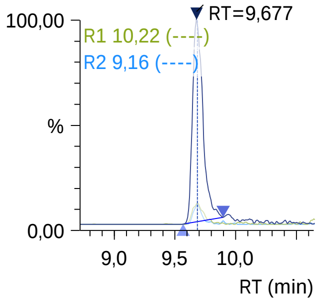

Compound: Baicalin (continued)

160ppb002
Conc 158,1544
Area 703629
R#1 445,20>112,90 10,30 (11,06)
R#2 445,20>175,10 10,80 (6,52)
Q 445,20>269,10 (-) 1,29e5

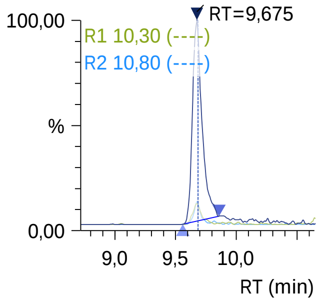

190 ppb001
Conc 190,0986
Area 843425
R#1 445,20>112,90 7,81 (11,06)
R#2 445,20>175,10 7,05 (6,52)
Q 445,20>269,10 (-) 1,56e5

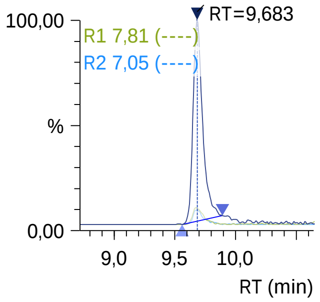

190ppb002
Conc 180,3717
Area 800858
R#1 445,20>112,90 11,52 (11,06)
R#2 445,20>175,10 6,94 (6,52)
Q 445,20>269,10 (-) 1,54e5

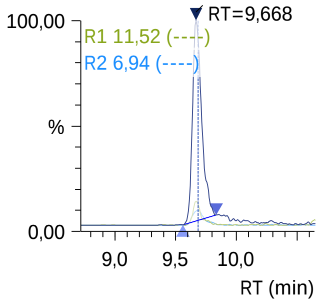

220 ppb001
Conc 209,4380
Area 928059
R#1 445,20>112,90 8,54 (11,06)
R#2 445,20>175,10 9,42 (6,52)
Q 445,20>269,10 (-) 1,74e5

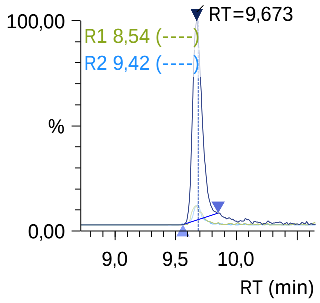

220ppb002
Conc 217,7710
Area 964526
R#1 445,20>112,90 8,87 (11,06)
R#2 445,20>175,10 7,35 (6,52)
Q 445,20>269,10 (-) 1,93e5

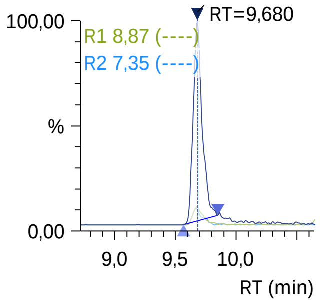

250 ppb001
Conc 255,5933
Area 1130045
R#1 445,20>112,90 10,64 (11,06)
R#2 445,20>175,10 9,81 (6,52)
Q 445,20>269,10 (-) 2,14e5

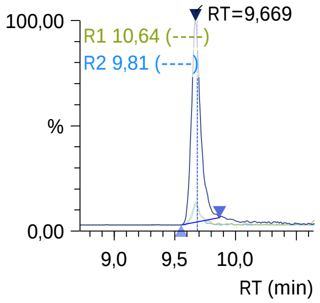

250 ppb002
Conc 258,8717
Area 1144392
R#1 445,20>112,90 9,00 (11,06)
R#2 445,20>175,10 9,10 (6,52)
Q 445,20>269,10 (-) 2,08e5

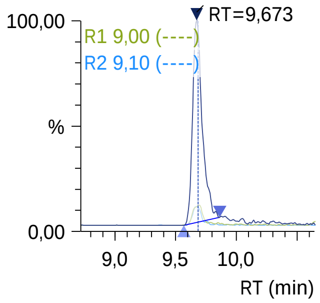

10H
Conc ----
Area ----
R#1 445,20>112,90 ---- (11,06)
R#2 445,20>175,10 ---- (6,52)
Q 445,20>269,10 (-) 2,78e2

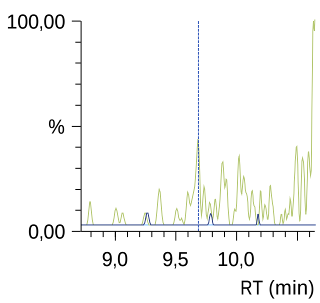

10M
Conc ----
Area ----
R#1 445,20>112,90 ---- (11,06)
R#2 445,20>175,10 ---- (6,52)
Q 445,20>269,10 (-) 2,77e2

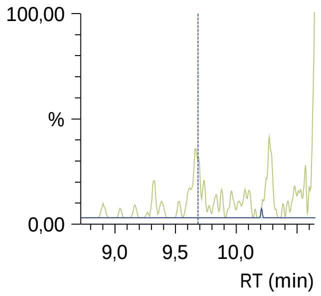

9H
Conc ----
Area ----
R#1 445,20>112,90 ---- (11,06)
R#2 445,20>175,10 ---- (6,52)
Q 445,20>269,10 (-) 4,83e2

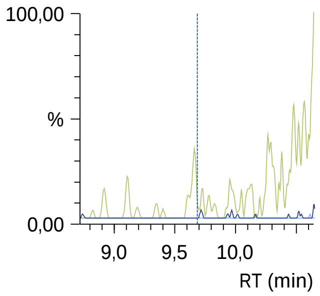

9M
Conc ----
Area ----
R#1 445,20>112,90 ---- (11,06)
R#2 445,20>175,10 ---- (6,52)
Q 445,20>269,10 (-) 5,56e2

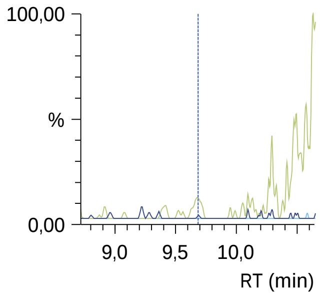

Compound: Chrysin

Curve Fit: Linear | Weighting: None | Zero: Not Forced
Quantitative Method: External Standard
Q 253,00>143,00
R^2 = 0.9373765 R = 0.9681820
y = 11310,64x + 312680,9

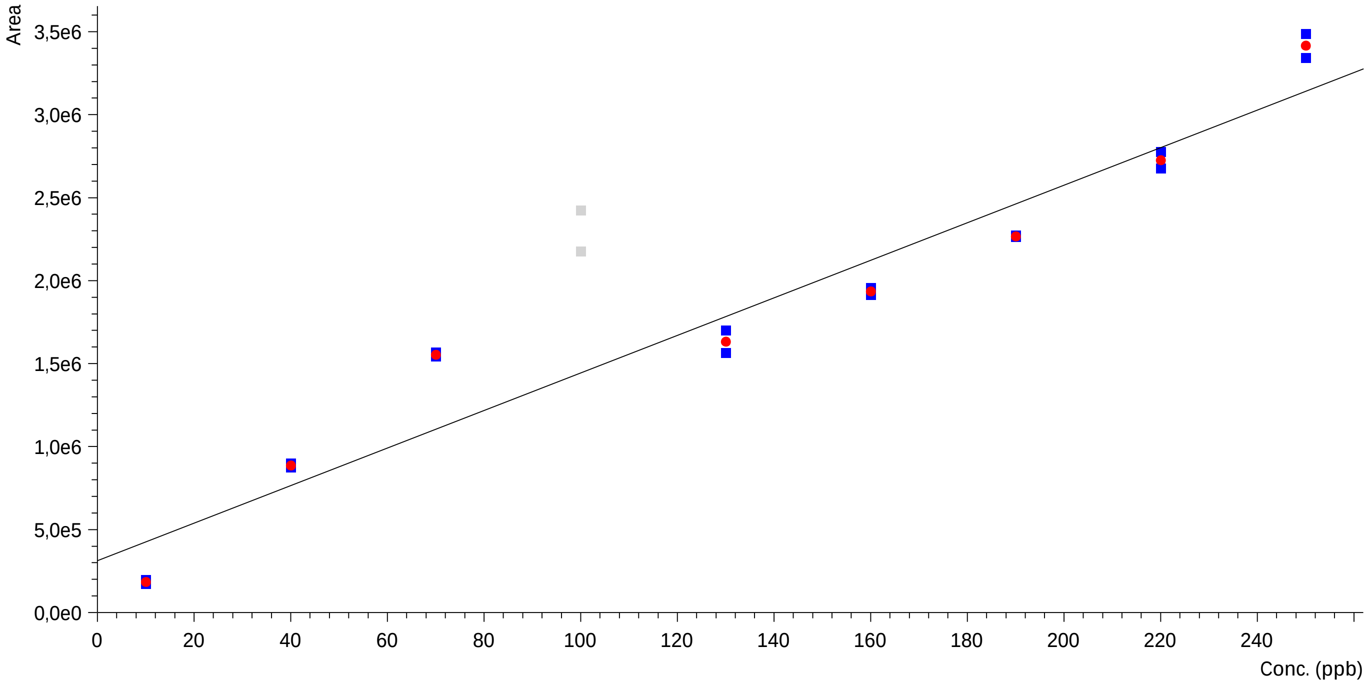

Sample ID	Area	Conc.	Sample Type	Level	Cal Point	Found RT	Ref 1 Actual Ratio
0	10824	-26,6879	Ctrl.	0	----	10,915	87,86
10ppb001	172739	-12,3726	Std.	1	Yes	10,892	84,23
10 ppb002	196430	-10,2780	Std.	1	Yes	10,890	76,15
40 ppb001	897898	51,7404	Std.	2	Yes	10,890	84,27
40ppb002	876322	49,8329	Std.	2	Yes	10,900	81,12
70 ppb001	1541730	108,6631	Std.	3	Yes	10,899	83,92
70ppb002	1567794	110,9675	Std.	3	Yes	10,894	87,38
100 ppb001	2424154	186,6802	Std.	4	No	10,887	86,85
100ppb002	2175242	164,6734	Std.	4	No	10,888	88,40
130 ppb001	1701311	122,7720	Std.	5	Yes	10,882	86,69
130ppb002	1564066	110,6379	Std.	5	Yes	10,892	92,07
160 ppb001	1915512	141,7100	Std.	6	Yes	10,894	86,87
160ppb002	1954986	145,2000	Std.	6	Yes	10,877	84,79
190 ppb001	2263900	172,5118	Std.	7	Yes	10,900	87,60
190ppb002	2271133	173,1513	Std.	7	Yes	10,876	89,01
220 ppb001	2675551	208,9068	Std.	8	Yes	10,886	88,49
220ppb002	2776196	217,8052	Std.	8	Yes	10,889	84,91
250 ppb001	3343707	267,9801	Std.	9	Yes	10,880	90,64
250 ppb002	3488386	280,7715	Std.	9	Yes	10,882	82,90
10H	----	----	Unk.	----	----	----	----
10M	----	----	Unk.	----	----	----	----
9H	----	----	Unk.	----	----	----	----
9M	----	----	Unk.	----	----	----	----
Average	1779888	129,7192				10,890	86,01
%RSD	57	68,5795				0,086	4,18
Maximum	3488386	280,7715				10,915	92,07
Minimum	10824	-26,6879				10,876	76,15
Std. Dev.	1006203	88,9608				0,009	3,60

Compound: Chrysin (continued)

0

Conc -26,6879
Area 10824
R#1 253,00>62,90 87,86 (87,38)
R#2 253,00>209,05 105,02 (66,51)
Q 253,00>143,00 (-) 3,33e3

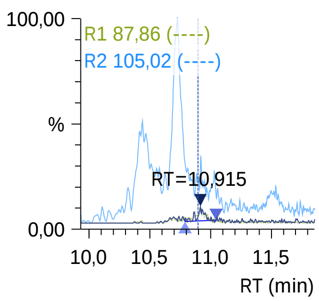

10ppb001

Conc -12,3726
Area 172739
R#1 253,00>62,90 84,23 (87,38)
R#2 253,00>209,05 75,48 (66,51)
Q 253,00>143,00 (-) 3,80e4

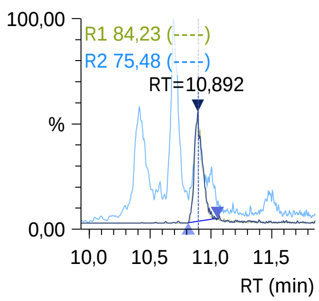

10 ppb002

Conc -10,2780
Area 196430
R#1 253,00>62,90 76,15 (87,38)
R#2 253,00>209,05 52,29 (66,51)
Q 253,00>143,00 (-) 4,08e4

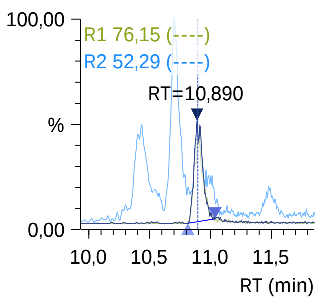

40 ppb001

Conc 51,7404
Area 897898
R#1 253,00>62,90 84,27 (87,38)
R#2 253,00>209,05 65,71 (66,51)
Q 253,00>143,00 (-) 1,88e5

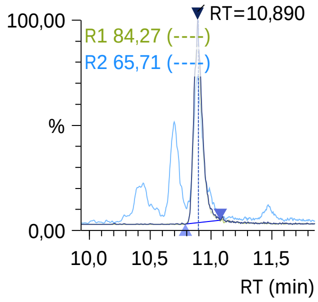

40ppb002

Conc 49,8329
Area 876322
R#1 253,00>62,90 81,12 (87,38)
R#2 253,00>209,05 68,74 (66,51)
Q 253,00>143,00 (-) 1,81e5

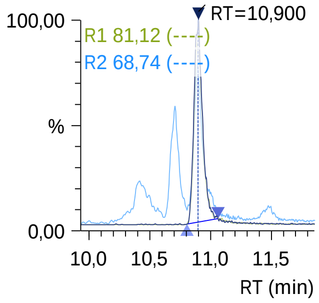

70 ppb001

Conc 108,6631
Area 1541730
R#1 253,00>62,90 83,92 (87,38)
R#2 253,00>209,05 60,87 (66,51)
Q 253,00>143,00 (-) 3,01e5

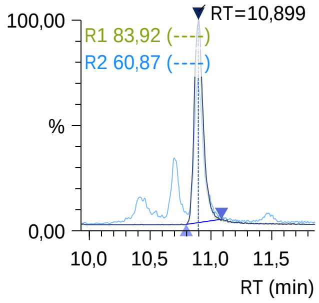

70ppb002

Conc 110,9675
Area 1567794
R#1 253,00>62,90 87,38 (87,38)
R#2 253,00>209,05 66,51 (66,51)
Q 253,00>143,00 (-) 3,11e5

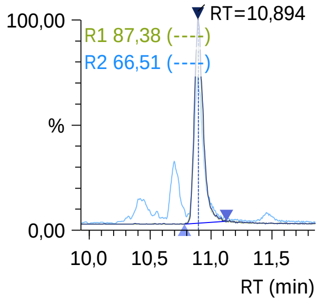

100 ppb001

Conc 186,6802
Area 2424154
R#1 253,00>62,90 86,85 (87,38)
R#2 253,00>209,05 67,90 (66,51)
Q 253,00>143,00 (-) 4,91e5

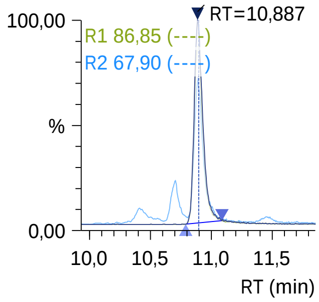

100ppb002

Conc 164,6734
Area 2175242
R#1 253,00>62,90 88,40 (87,38)
R#2 253,00>209,05 66,85 (66,51)
Q 253,00>143,00 (-) 4,36e5

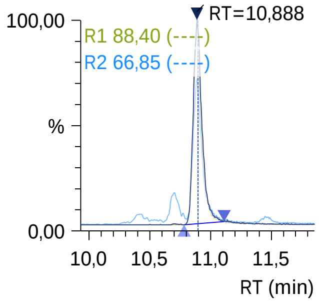

130 ppb001

Conc 122,7720
Area 1701311
R#1 253,00>62,90 86,69 (87,38)
R#2 253,00>209,05 64,16 (66,51)
Q 253,00>143,00 (-) 3,40e5

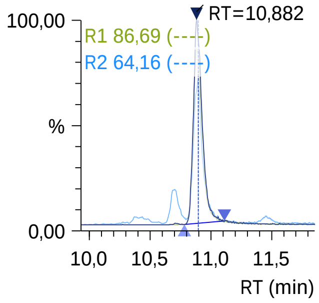

130ppb002

Conc 110,6379
Area 1564066
R#1 253,00>62,90 92,07 (87,38)
R#2 253,00>209,05 67,01 (66,51)
Q 253,00>143,00 (-) 3,13e5

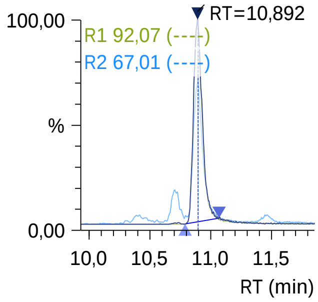

160 ppb001

Conc 141,7100
Area 1915512
R#1 253,00>62,90 86,87 (87,38)
R#2 253,00>209,05 66,86 (66,51)
Q 253,00>143,00 (-) 3,95e5

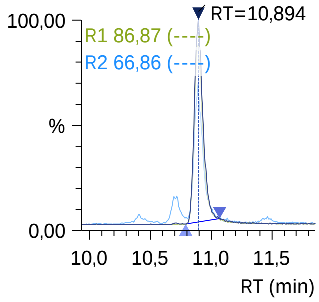

Compound: Chrysin (continued)

160ppb002
Conc 145,2000
Area 1954986
R#1 253,00>62,90 84,79 (87,38)
R#2 253,00>209,05 67,66 (66,51)
Q 253,00>143,00 (-) 3,76e5

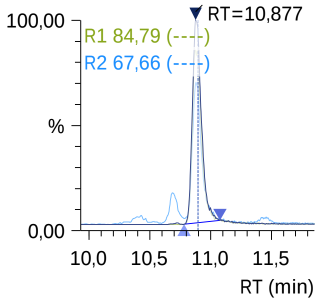

190 ppb001
Conc 172,5118
Area 2263900
R#1 253,00>62,90 87,60 (87,38)
R#2 253,00>209,05 65,54 (66,51)
Q 253,00>143,00 (-) 4,40e5

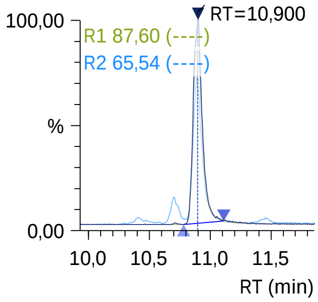

190ppb002
Conc 173,1513
Area 2271133
R#1 253,00>62,90 89,01 (87,38)
R#2 253,00>209,05 67,31 (66,51)
Q 253,00>143,00 (-) 4,37e5

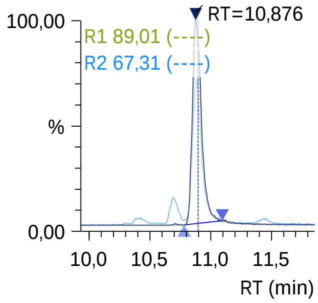

220 ppb001
Conc 208,9068
Area 2675551
R#1 253,00>62,90 88,49 (87,38)
R#2 253,00>209,05 67,18 (66,51)
Q 253,00>143,00 (-) 5,21e5

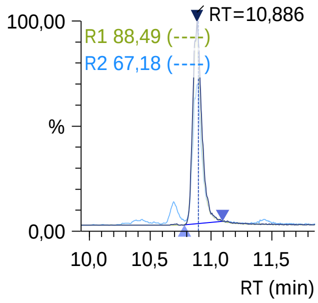

220ppb002
Conc 217,8052
Area 2776196
R#1 253,00>62,90 84,91 (87,38)
R#2 253,00>209,05 68,33 (66,51)
Q 253,00>143,00 (-) 5,59e5

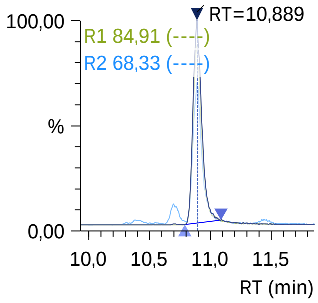

250 ppb001
Conc 267,9801
Area 3343707
R#1 253,00>62,90 90,64 (87,38)
R#2 253,00>209,05 68,45 (66,51)
Q 253,00>143,00 (-) 6,37e5

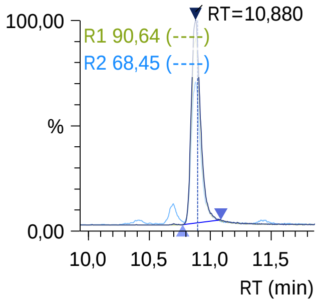

250 ppb002
Conc 280,7715
Area 3488386
R#1 253,00>62,90 82,90 (87,38)
R#2 253,00>209,05 65,31 (66,51)
Q 253,00>143,00 (-) 6,95e5

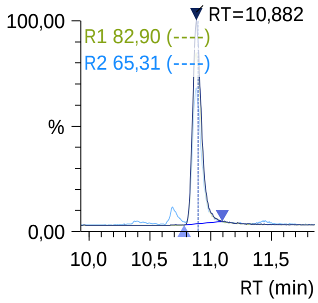

10H
Conc ----
Area ----
R#1 253,00>62,90 ---- (87,38)
R#2 253,00>209,05 ---- (66,51)
Q 253,00>143,00 (-) 6,52e2

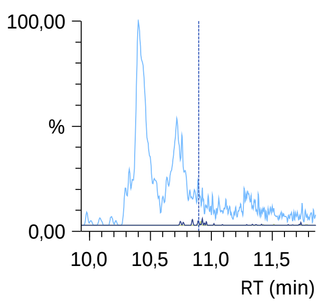

10M
Conc ----
Area ----
R#1 253,00>62,90 ---- (87,38)
R#2 253,00>209,05 ---- (66,51)
Q 253,00>143,00 (-) 5,90e2

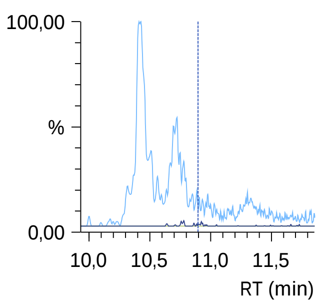

9H
Conc ----
Area ----
R#1 253,00>62,90 ---- (87,38)
R#2 253,00>209,05 ---- (66,51)
Q 253,00>143,00 (-) 1,02e3

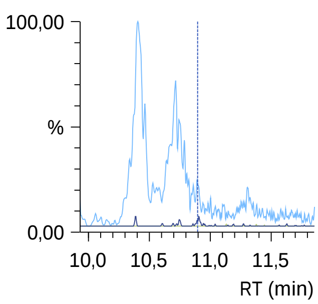

9M
Conc ----
Area ----
R#1 253,00>62,90 ---- (87,38)
R#2 253,00>209,05 ---- (66,51)
Q 253,00>143,00 (-) 4,90e2

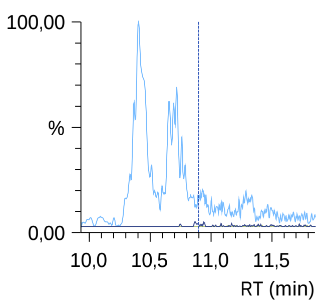

Compound: Ac. Quinico

Curve Fit: Linear | Weighting: None | Zero: Not Forced
Quantitative Method: External Standard
Q 191,20>84,80
R^2 = 0.9905902 R = 0.9952840
y = 1818,244x - 5402,542

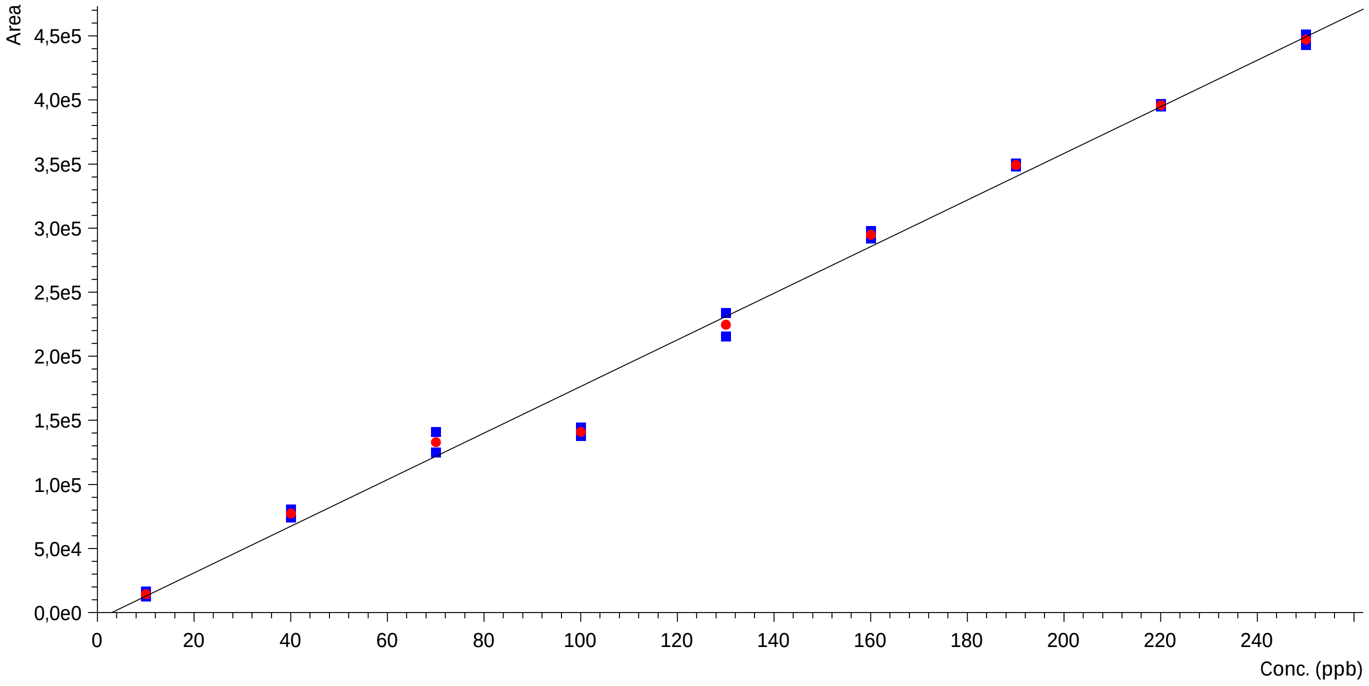

Sample ID	Area	Conc.	Sample Type	Level	Cal Point	Found RT	Ref 1 Actual Ratio
0	10200	8,5813	Ctrl.	0	----	3,522	0,00
10ppb001	12516	9,8548	Std.	1	Yes	3,611	0,00
10 ppb002	16568	12,0836	Std.	1	Yes	3,614	0,00
40 ppb001	74373	43,8750	Std.	2	Yes	3,608	6,58
40ppb002	80604	47,3021	Std.	2	Yes	3,604	9,85
70 ppb001	141075	80,5598	Std.	3	Yes	3,605	0,00
70ppb002	125231	71,8462	Std.	3	Yes	3,608	9,31
100 ppb001	137884	78,8051	Std.	4	Yes	3,607	0,00
100ppb002	144499	82,4428	Std.	4	Yes	3,617	0,00
130 ppb001	215488	121,4857	Std.	5	Yes	3,604	0,51
130ppb002	233923	131,6244	Std.	5	Yes	3,604	1,50
160 ppb001	291986	163,5582	Std.	6	Yes	3,606	0,27
160ppb002	297716	166,7096	Std.	6	Yes	3,608	0,00
190 ppb001	350712	195,8561	Std.	7	Yes	3,604	0,00
190ppb002	348282	194,5200	Std.	7	Yes	3,605	0,00
220 ppb001	397017	221,3232	Std.	8	Yes	3,605	0,00
220ppb002	395081	220,2582	Std.	8	Yes	3,602	0,00
250 ppb001	451333	251,1958	Std.	9	Yes	3,606	0,00
250 ppb002	443157	246,6994	Std.	9	Yes	3,610	1,04
10H	----	----	Unk.	----	----	----	----
10M	----	----	Unk.	----	----	----	----
9H	----	----	Unk.	----	----	----	----
9M	----	----	Unk.	----	----	----	----
Average	219350	123,6095				3,603	1,53
%RSD	69	66,9059				0,553	210,36
Maximum	451333	251,1958				3,617	9,85
Minimum	10200	8,5813				3,522	0,00
Std. Dev.	150373	82,7021				0,020	3,22

Compound: Ac. Quinico (continued)

0
Conc 8,5813
Area 10200
R#1 191,20>147,05 0,00 (0,27)
R#2 191,20>93,00 2,19 (42,47)
Q 191,20>84,80 (-) 1,47e3

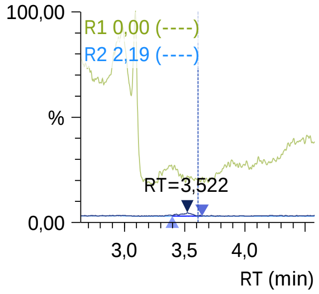

10ppb001
Conc 9,8548
Area 12516
R#1 191,20>147,05 0,00 (0,27)
R#2 191,20>93,00 39,83 (42,47)
Q 191,20>84,80 (-) 3,57e3

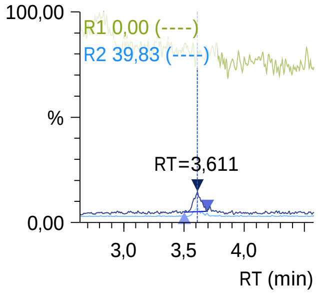

10 ppb002
Conc 12,0836
Area 16568
R#1 191,20>147,05 0,00 (0,27)
R#2 191,20>93,00 28,31 (42,47)
Q 191,20>84,80 (-) 4,10e3

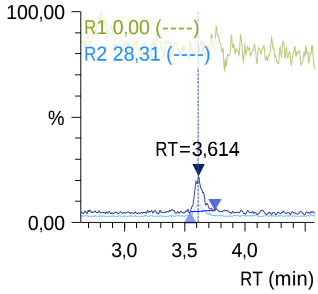

40 ppb001
Conc 43,8750
Area 74373
R#1 191,20>147,05 6,58 (0,27)
R#2 191,20>93,00 34,69 (42,47)
Q 191,20>84,80 (-) 1,49e4

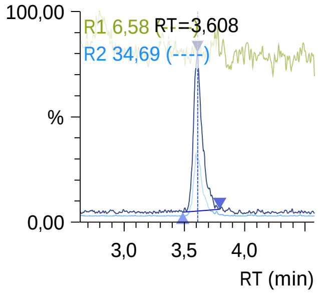

40ppb002
Conc 47,3021
Area 80604
R#1 191,20>147,05 9,85 (0,27)
R#2 191,20>93,00 42,05 (42,47)
Q 191,20>84,80 (-) 1,45e4

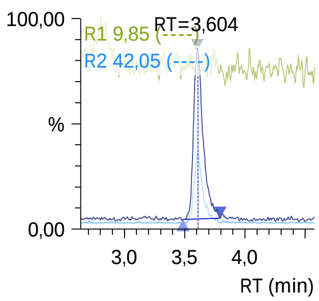

70 ppb001
Conc 80,5598
Area 141075
R#1 191,20>147,05 0,00 (0,27)
R#2 191,20>93,00 40,17 (42,47)
Q 191,20>84,80 (-) 2,64e4

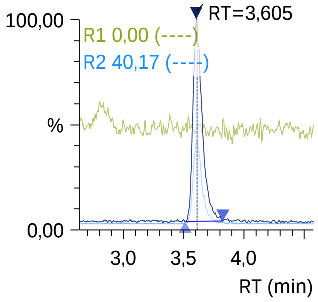

70ppb002
Conc 71,8462
Area 125231
R#1 191,20>147,05 9,31 (0,27)
R#2 191,20>93,00 37,72 (42,47)
Q 191,20>84,80 (-) 2,50e4

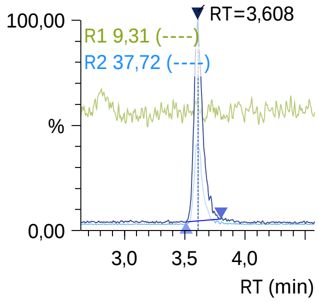

100 ppb001
Conc 78,8051
Area 137884
R#1 191,20>147,05 0,00 (0,27)
R#2 191,20>93,00 40,38 (42,47)
Q 191,20>84,80 (-) 2,79e4

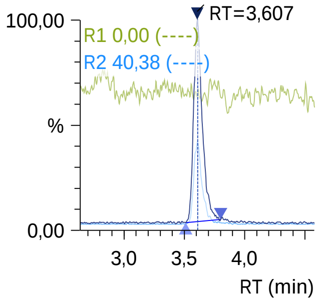

100ppb002
Conc 82,4428
Area 144499
R#1 191,20>147,05 0,00 (0,27)
R#2 191,20>93,00 40,57 (42,47)
Q 191,20>84,80 (-) 2,82e4

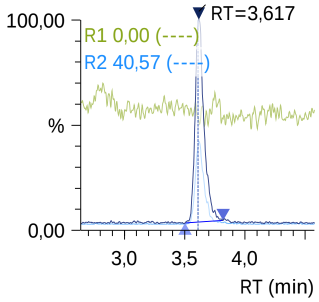

130 ppb001
Conc 121,4857
Area 215488
R#1 191,20>147,05 0,51 (0,27)
R#2 191,20>93,00 41,04 (42,47)
Q 191,20>84,80 (-) 4,23e4

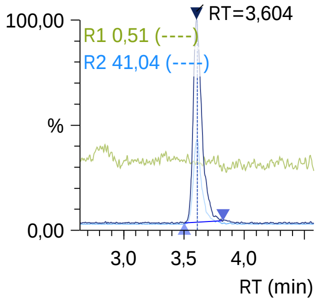

130ppb002
Conc 131,6244
Area 233923
R#1 191,20>147,05 1,50 (0,27)
R#2 191,20>93,00 38,78 (42,47)
Q 191,20>84,80 (-) 4,73e4

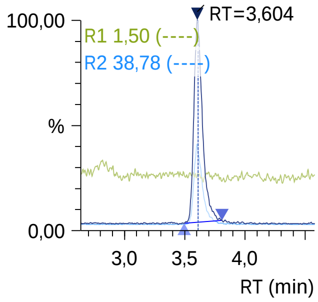

160 ppb001
Conc 163,5582
Area 291986
R#1 191,20>147,05 0,27 (0,27)
R#2 191,20>93,00 42,47 (42,47)
Q 191,20>84,80 (-) 5,70e4

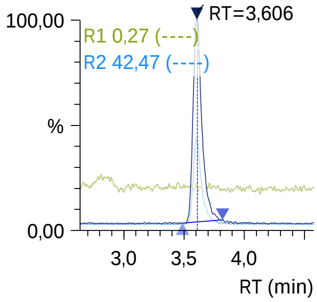

Compound: Ac. Quinico (continued)

160ppb002
Conc 166,7096
Area 297716
R#1 191,20>147,05 0,00 (0,27)
R#2 191,20>93,00 38,35 (42,47)
Q 191,20>84,80 (-) 6,03e4

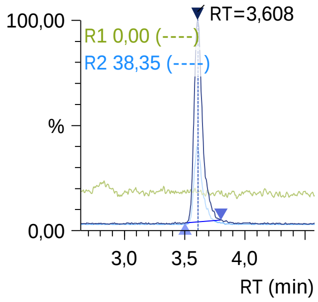

190 ppb001
Conc 195,8561
Area 350712
R#1 191,20>147,05 0,00 (0,27)
R#2 191,20>93,00 40,27 (42,47)
Q 191,20>84,80 (-) 6,98e4

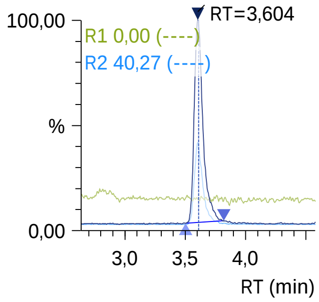

190ppb002
Conc 194,5200
Area 348282
R#1 191,20>147,05 0,00 (0,27)
R#2 191,20>93,00 39,48 (42,47)
Q 191,20>84,80 (-) 7,01e4

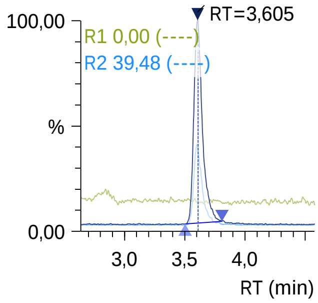

220 ppb001
Conc 221,3232
Area 397017
R#1 191,20>147,05 0,00 (0,27)
R#2 191,20>93,00 39,40 (42,47)
Q 191,20>84,80 (-) 7,78e4

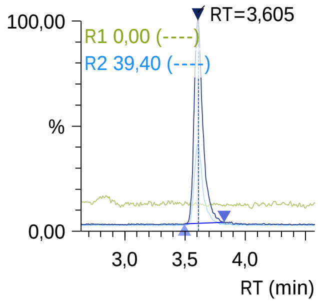

220ppb002
Conc 220,2582
Area 395081
R#1 191,20>147,05 0,00 (0,27)
R#2 191,20>93,00 43,21 (42,47)
Q 191,20>84,80 (-) 7,77e4

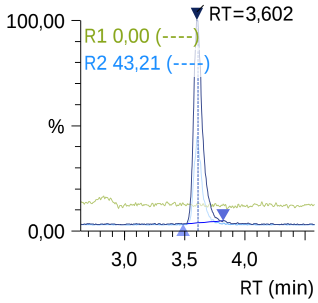

250 ppb001
Conc 251,1958
Area 451333
R#1 191,20>147,05 0,00 (0,27)
R#2 191,20>93,00 38,79 (42,47)
Q 191,20>84,80 (-) 9,16e4

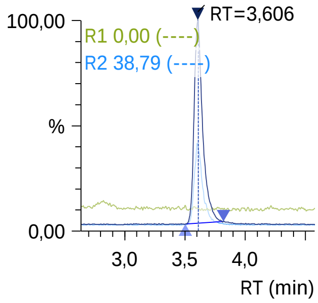

250 ppb002
Conc 246,6994
Area 443157
R#1 191,20>147,05 1,04 (0,27)
R#2 191,20>93,00 40,19 (42,47)
Q 191,20>84,80 (-) 8,87e4

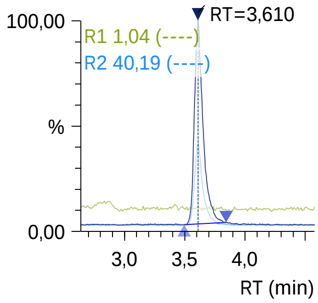

10H
Conc ----
Area ----
R#1 191,20>147,05 ---- (0,27)
R#2 191,20>93,00 ---- (42,47)
Q 191,20>84,80 (-) 1,94e5

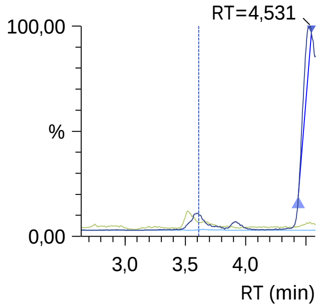

10M
Conc ----
Area ----
R#1 191,20>147,05 ---- (0,27)
R#2 191,20>93,00 ---- (42,47)
Q 191,20>84,80 (-) 2,26e5

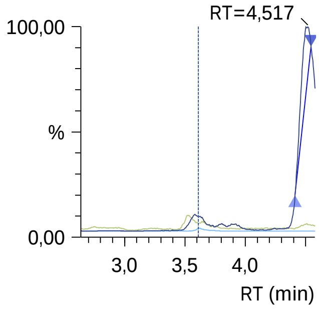

9H
Conc ----
Area ----
R#1 191,20>147,05 ---- (0,27)
R#2 191,20>93,00 ---- (42,47)
Q 191,20>84,80 (-) 8,29e5

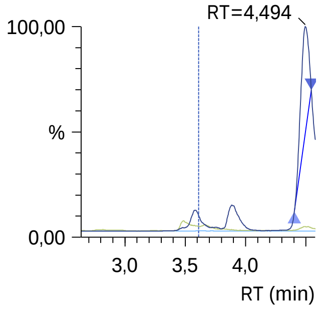

9M
Conc ----
Area ----
R#1 191,20>147,05 ---- (0,27)
R#2 191,20>93,00 ---- (42,47)
Q 191,20>84,80 (-) 8,06e5

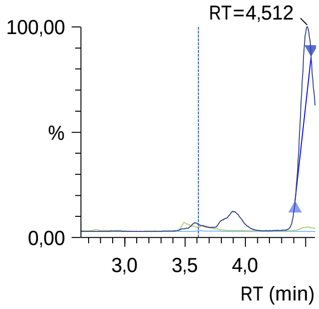

Compound: Ac. Malico

Curve Fit: Linear | Weighting: None | Zero: Not Forced
Quantitative Method: External Standard
Q 133,20>114,90
R^2 = 0.9845702 R = 0.9922551
y = 1793,454x - 16836,25

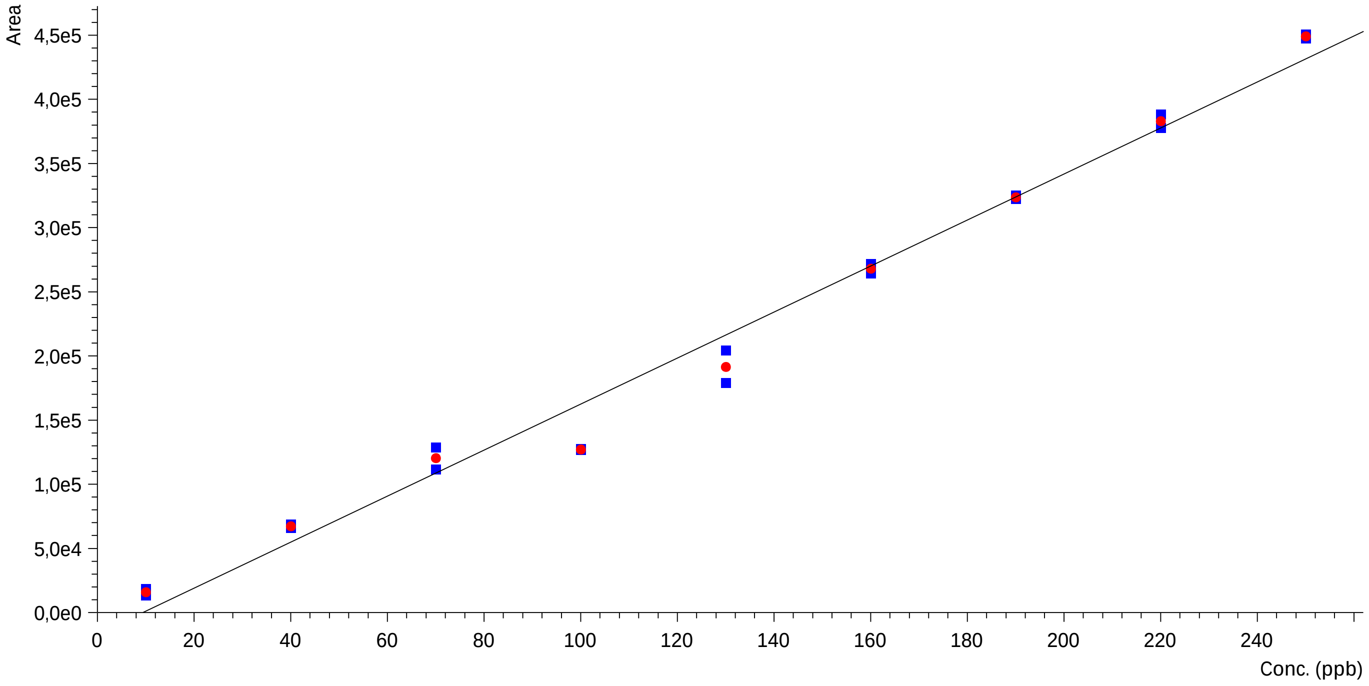

Sample ID	Area	Conc.	Sample Type	Level	Cal Point	Found RT	Ref 1 Actual Ratio
0	----	----	Ctrl.	0	----	----	----
10ppb001	13450	16,8870	Std.	1	Yes	3,912	49,69
10 ppb002	18446	19,6726	Std.	1	Yes	3,917	65,19
40 ppb001	66179	46,2878	Std.	2	Yes	3,923	60,80
40ppb002	68683	47,6844	Std.	2	Yes	3,903	58,43
70 ppb001	129036	81,3361	Std.	3	Yes	3,907	51,08
70ppb002	111687	71,6621	Std.	3	Yes	3,920	59,63
100 ppb001	126687	80,0263	Std.	4	Yes	3,920	56,65
100ppb002	127517	80,4892	Std.	4	Yes	3,918	54,25
130 ppb001	179098	109,2495	Std.	5	Yes	3,918	55,63
130ppb002	204235	123,2655	Std.	5	Yes	3,927	57,74
160 ppb001	264333	156,7755	Std.	6	Yes	3,913	57,80
160ppb002	271889	160,9884	Std.	6	Yes	3,923	58,26
190 ppb001	322281	189,0862	Std.	7	Yes	3,909	61,88
190ppb002	325393	190,8212	Std.	7	Yes	3,906	58,33
220 ppb001	377826	220,0572	Std.	8	Yes	3,916	60,57
220ppb002	388352	225,9264	Std.	8	Yes	3,925	53,45
250 ppb001	450820	260,7571	Std.	9	Yes	3,913	60,87
250 ppb002	447717	259,0273	Std.	9	Yes	3,916	59,47
10H	4424647	2476,4968	Unk.	----	----	3,939	50,41
10M	6045815	3380,4332	Unk.	----	----	3,940	50,89
9H	12421338	6935,3186	Unk.	----	----	3,927	51,87
9M	18244030	10181,9546	Unk.	----	----	3,923	53,22
Average	2046794	1150,6456				3,919	56,64
%RSD	227	224,8892				0,239	7,53
Maximum	18244030	10181,9546				3,940	65,19
Minimum	13450	16,8870				3,903	49,69
Std. Dev.	4640881	2587,6778				0,009	4,27

Compound: Ac. Malico (continued)

0
Conc ----
Area ----
R#1 133,20>70,95 ---- (58,33)
R#2 133,20>72,90 ---- (14,00)
Q 133,20>114,90 (-) 8,15e2

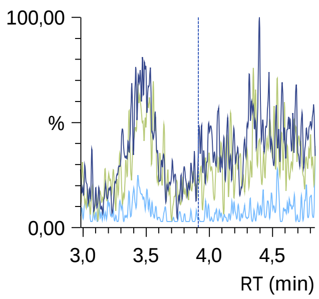

10ppb001
Conc 16,8870
Area 13450
R#1 133,20>70,95 49,69 (58,33)
R#2 133,20>72,90 12,98 (14,00)
Q 133,20>114,90 (-) 3,20e3

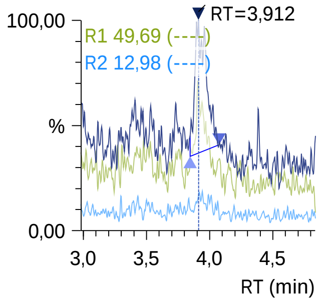

10 ppb002
Conc 19,6726
Area 18446
R#1 133,20>70,95 65,19 (58,33)
R#2 133,20>72,90 13,12 (14,00)
Q 133,20>114,90 (-) 3,04e3

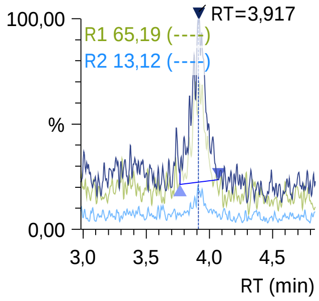

40 ppb001
Conc 46,2878
Area 66179
R#1 133,20>70,95 60,80 (58,33)
R#2 133,20>72,90 13,81 (14,00)
Q 133,20>114,90 (-) 9,64e3

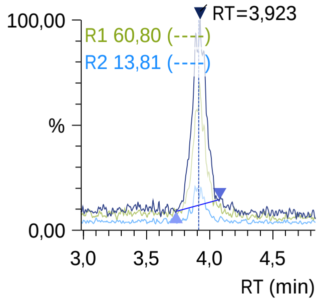

40ppb002
Conc 47,6844
Area 68683
R#1 133,20>70,95 58,43 (58,33)
R#2 133,20>72,90 14,28 (14,00)
Q 133,20>114,90 (-) 9,68e3

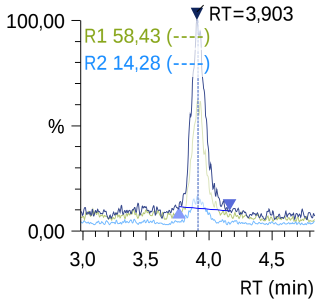

70 ppb001
Conc 81,3361
Area 129036
R#1 133,20>70,95 51,08 (58,33)
R#2 133,20>72,90 16,61 (14,00)
Q 133,20>114,90 (-) 1,72e4

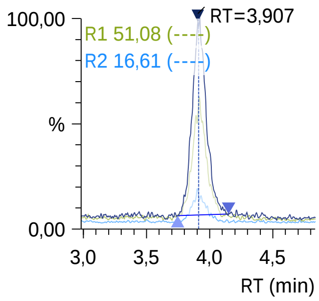

70ppb002
Conc 71,6621
Area 111687
R#1 133,20>70,95 59,63 (58,33)
R#2 133,20>72,90 13,94 (14,00)
Q 133,20>114,90 (-) 1,47e4

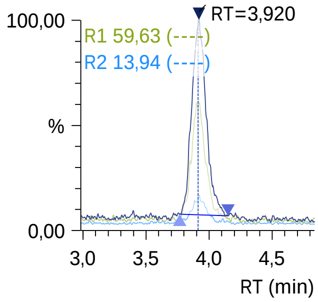

100 ppb001
Conc 80,0263
Area 126687
R#1 133,20>70,95 56,65 (58,33)
R#2 133,20>72,90 15,67 (14,00)
Q 133,20>114,90 (-) 1,67e4

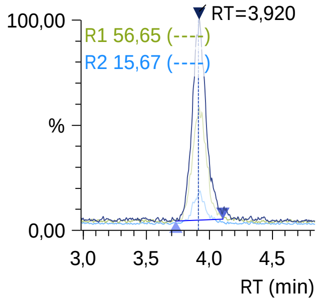

100ppb002
Conc 80,4892
Area 127517
R#1 133,20>70,95 54,25 (58,33)
R#2 133,20>72,90 12,36 (14,00)
Q 133,20>114,90 (-) 1,81e4

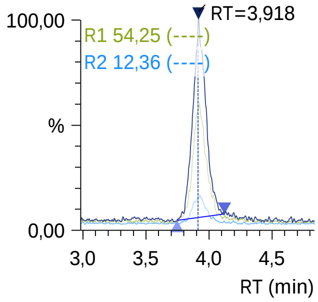

130 ppb001
Conc 109,2495
Area 179098
R#1 133,20>70,95 55,63 (58,33)
R#2 133,20>72,90 13,44 (14,00)
Q 133,20>114,90 (-) 2,63e4

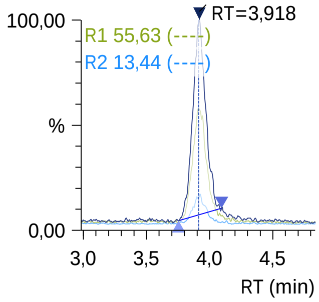

130ppb002
Conc 123,2655
Area 204235
R#1 133,20>70,95 57,74 (58,33)
R#2 133,20>72,90 14,67 (14,00)
Q 133,20>114,90 (-) 2,66e4

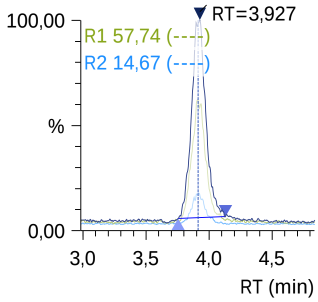

160 ppb001
Conc 156,7755
Area 264333
R#1 133,20>70,95 57,80 (58,33)
R#2 133,20>72,90 14,78 (14,00)
Q 133,20>114,90 (-) 3,57e4

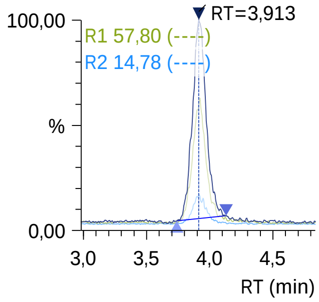

Compound: Ac. Malico (continued)

160ppb002
Conc 160,9884
Area 271889
R#1 133,20>70,95 58,26 (58,33)
R#2 133,20>72,90 14,29 (14,00)
Q 133,20>114,90 (-) 3,68e4

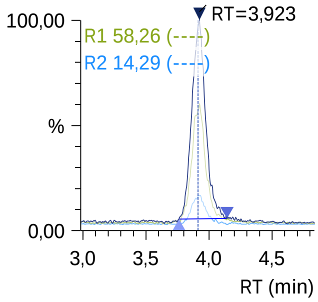

190 ppb001
Conc 189,0862
Area 322281
R#1 133,20>70,95 61,88 (58,33)
R#2 133,20>72,90 15,29 (14,00)
Q 133,20>114,90 (-) 4,24e4

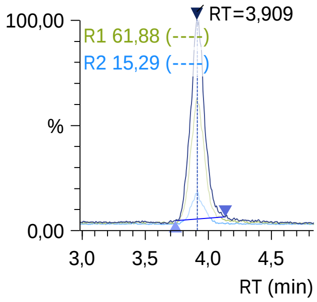

190ppb002
Conc 190,8212
Area 325393
R#1 133,20>70,95 58,33 (58,33)
R#2 133,20>72,90 14,00 (14,00)
Q 133,20>114,90 (-) 4,20e4

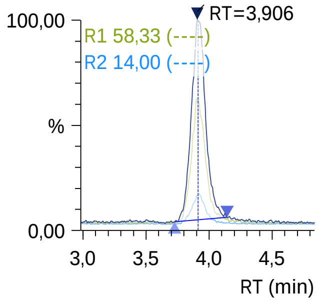

220 ppb001
Conc 220,0572
Area 377826
R#1 133,20>70,95 60,57 (58,33)
R#2 133,20>72,90 14,76 (14,00)
Q 133,20>114,90 (-) 4,92e4

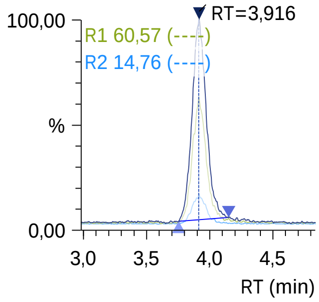

220ppb002
Conc 225,9264
Area 388352
R#1 133,20>70,95 53,45 (58,33)
R#2 133,20>72,90 12,23 (14,00)
Q 133,20>114,90 (-) 5,00e4

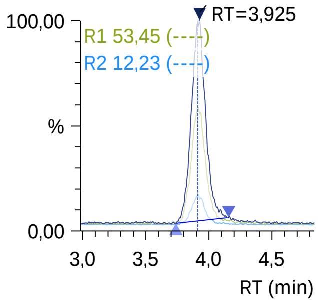

250 ppb001
Conc 260,7571
Area 450820
R#1 133,20>70,95 60,87 (58,33)
R#2 133,20>72,90 15,04 (14,00)
Q 133,20>114,90 (-) 5,76e4

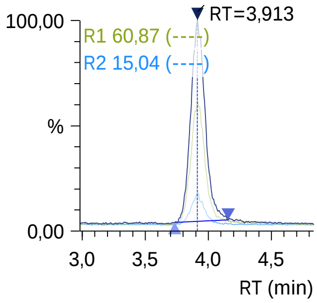

250 ppb002
Conc 259,0273
Area 447717
R#1 133,20>70,95 59,47 (58,33)
R#2 133,20>72,90 13,81 (14,00)
Q 133,20>114,90 (-) 5,76e4

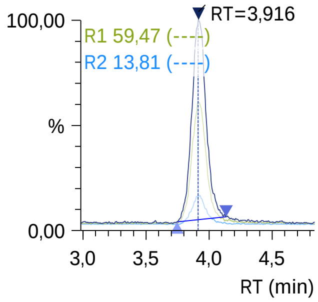

10H
Conc 2476,4968
Area 4424647
R#1 133,20>70,95 50,41 (58,33)
R#2 133,20>72,90 12,16 (14,00)
Q 133,20>114,90 (-) 5,98e5

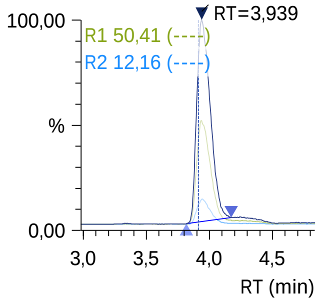

10M
Conc 3380,4332
Area 6045815
R#1 133,20>70,95 50,89 (58,33)
R#2 133,20>72,90 12,21 (14,00)
Q 133,20>114,90 (-) 6,25e5

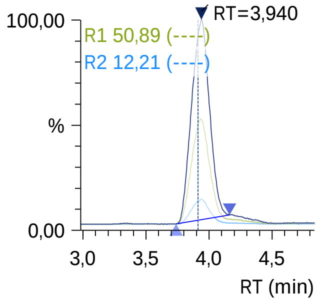

9H
Conc 6935,3186
Area 12421338
R#1 133,20>70,95 51,87 (58,33)
R#2 133,20>72,90 12,59 (14,00)
Q 133,20>114,90 (-) 2,02e6

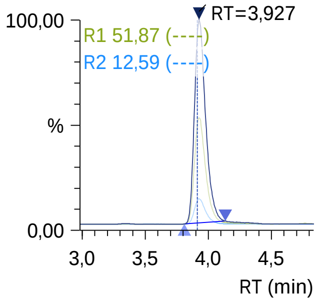

9M
Conc 10181,9546
Area 18244030
R#1 133,20>70,95 53,22 (58,33)
R#2 133,20>72,90 12,76 (14,00)
Q 133,20>114,90 (-) 2,01e6

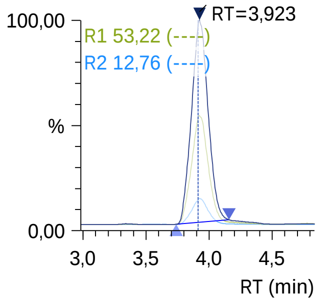

Compound: Kaempherol

Curve Fit: Linear | Weighting: None | Zero: Not Forced
Quantitative Method: External Standard
Q 285,10>229,10
R^2 = 0.8753904 R = 0.9356230
y = 531,9160x + 18769,33

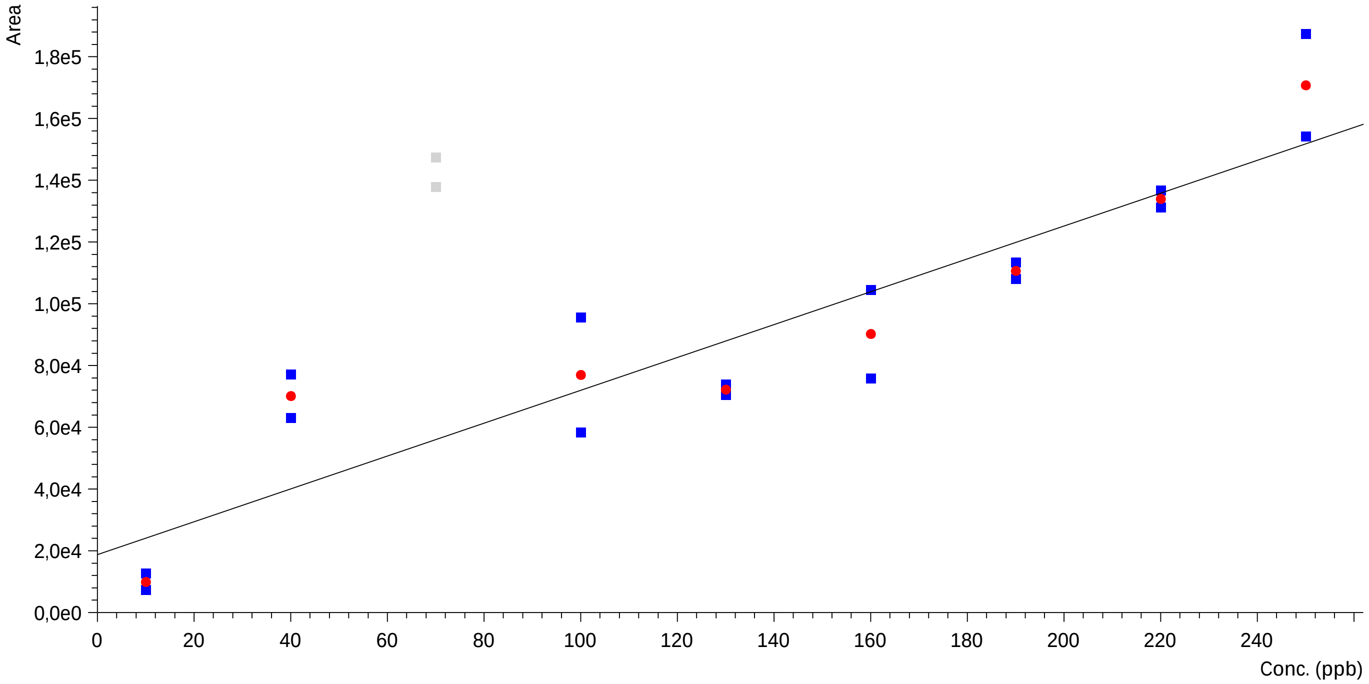

Sample ID	Area	Conc.	Sample Type	Level	Cal Point	Found RT	Ref 1 Actual Ratio
0	----	----	Ctrl.	0	----	----	----
10ppb001	7360	-21,4487	Std.	1	Yes	10,108	65,52
10 ppb002	12626	-11,5485	Std.	1	Yes	10,098	109,62
40 ppb001	63175	83,4831	Std.	2	Yes	10,127	78,00
40ppb002	77130	109,7179	Std.	2	Yes	10,110	128,71
70 ppb001	137891	223,9476	Std.	3	No	10,105	91,54
70ppb002	147376	241,7797	Std.	3	No	10,127	86,31
100 ppb001	95722	144,6703	Std.	4	Yes	10,117	97,12
100ppb002	58407	74,5183	Std.	4	Yes	10,109	103,06
130 ppb001	70635	97,5070	Std.	5	Yes	10,105	89,46
130ppb002	73902	103,6484	Std.	5	Yes	10,109	93,33
160 ppb001	104578	161,3196	Std.	6	Yes	10,117	89,08
160ppb002	75910	107,4251	Std.	6	Yes	10,105	162,93
190 ppb001	107981	167,7181	Std.	7	Yes	10,122	95,06
190ppb002	113482	178,0587	Std.	7	Yes	10,096	83,46
220 ppb001	131216	211,3985	Std.	8	Yes	10,094	95,99
220ppb002	136759	221,8198	Std.	8	Yes	10,102	99,61
250 ppb001	154219	254,6444	Std.	9	Yes	10,108	81,34
250 ppb002	187423	317,0680	Std.	9	Yes	10,096	95,15
10H	----	----	Unk.	----	----	----	----
10M	----	----	Unk.	----	----	----	----
9H	----	----	Unk.	----	----	----	----
9M	4962	-25,9572	Unk.	----	----	10,130	75,17
Average	92671	138,9353				10,110	95,81
%RSD	55	69,0219				0,109	22,10
Maximum	187423	317,0680				10,130	162,93
Minimum	4962	-25,9572				10,094	65,52
Std. Dev.	51009	95,8958				0,011	21,18

Compound: Kaempherol (continued)

0
Conc ----
Area ----
R#1 285,10>116,95 ---- (95,08)
R#2 285,10>187,05 ---- (78,78)

Q 285,10>229,10 (-) 5,62e2

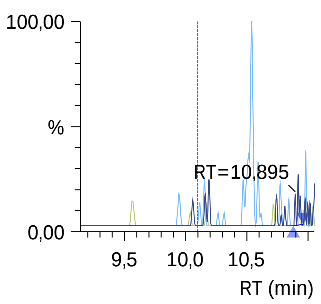

10ppb001
Conc -21,4487
Area 7360
R#1 285,10>116,95 65,52 (95,08)
R#2 285,10>187,05 45,88 (78,78)

Q 285,10>229,10 (-) 2,48e3

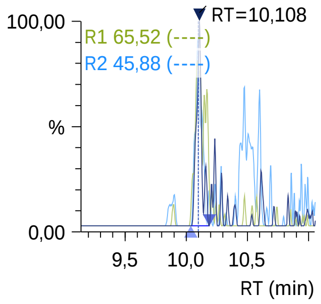

10 ppb002
Conc -11,5485
Area 12626
R#1 285,10>116,95 109,62 (95,08)
R#2 285,10>187,05 122,36 (78,78)

Q 285,10>229,10 (-) 2,67e3

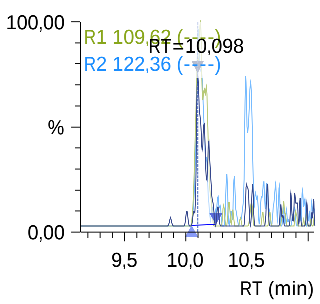

40 ppb001
Conc 83,4831
Area 63175
R#1 285,10>116,95 78,00 (95,08)
R#2 285,10>187,05 97,43 (78,78)

Q 285,10>229,10 (-) 1,29e4

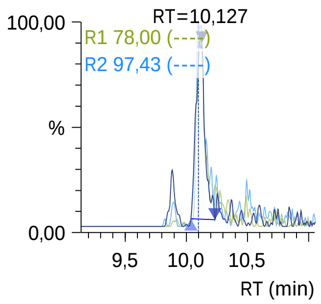

40ppb002
Conc 109,7179
Area 77130
R#1 285,10>116,95 128,71 (95,08)
R#2 285,10>187,05 92,05 (78,78)

Q 285,10>229,10 (-) 1,55e4

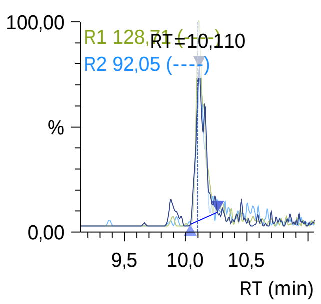

70 ppb001
Conc 223,9476
Area 137891
R#1 285,10>116,95 91,54 (95,08)
R#2 285,10>187,05 90,24 (78,78)

Q 285,10>229,10 (-) 3,26e4

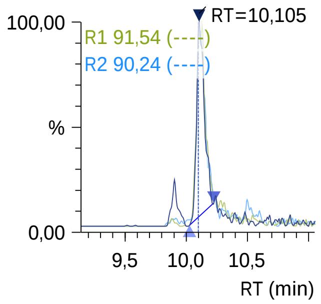

70ppb002
Conc 241,7797
Area 147376
R#1 285,10>116,95 86,31 (95,08)
R#2 285,10>187,05 92,69 (78,78)

Q 285,10>229,10 (-) 2,77e4

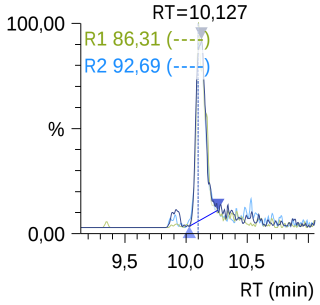

100 ppb001
Conc 144,6703
Area 95722
R#1 285,10>116,95 97,12 (95,08)
R#2 285,10>187,05 90,03 (78,78)

Q 285,10>229,10 (-) 2,35e4

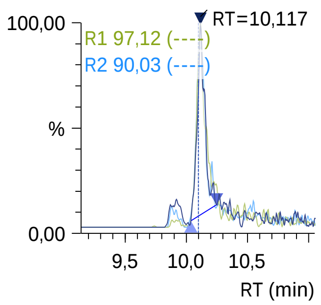

100ppb002
Conc 74,5183
Area 58407
R#1 285,10>116,95 103,06 (95,08)
R#2 285,10>187,05 82,99 (78,78)

Q 285,10>229,10 (-) 1,23e4

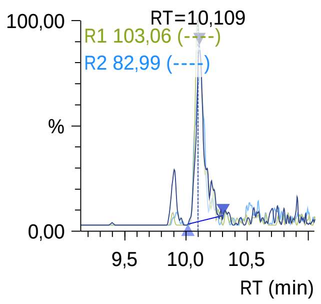

130 ppb001
Conc 97,5070
Area 70635
R#1 285,10>116,95 89,46 (95,08)
R#2 285,10>187,05 72,25 (78,78)

Q 285,10>229,10 (-) 1,81e4

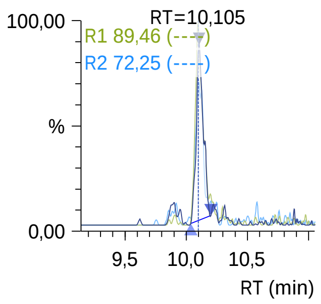

130ppb002
Conc 103,6484
Area 73902
R#1 285,10>116,95 93,33 (95,08)
R#2 285,10>187,05 107,88 (78,78)

Q 285,10>229,10 (-) 1,72e4

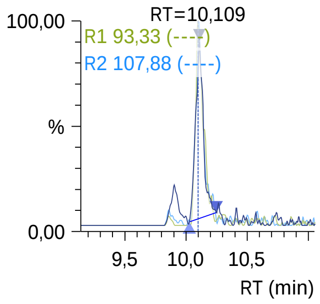

160 ppb001
Conc 161,3196
Area 104578
R#1 285,10>116,95 89,08 (95,08)
R#2 285,10>187,05 77,93 (78,78)

Q 285,10>229,10 (-) 2,27e4

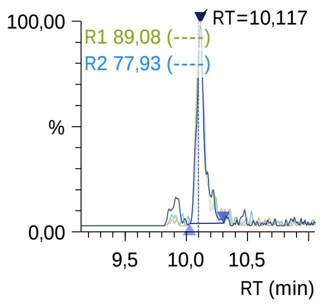

Compound: Kaempherol (continued)

160ppb002

Conc 107,4251

Area 75910

R#1 285,10>116,95 162,93 (95,08)

R#2 285,10>187,05 130,42 (78,78)

Q 285,10>229,10 (-) 1,54e4

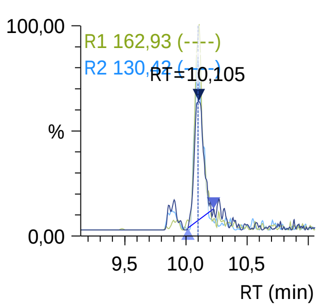

190 ppb001

Conc 167,7181

Area 107981

R#1 285,10>116,95 95,06 (95,08)

R#2 285,10>187,05 93,21 (78,78)

Q 285,10>229,10 (-) 2,39e4

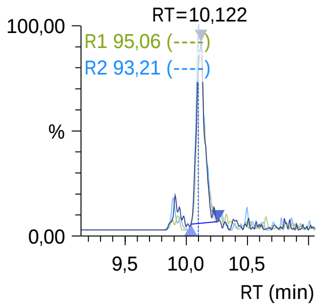

190ppb002

Conc 178,0587

Area 113482

R#1 285,10>116,95 83,46 (95,08)

R#2 285,10>187,05 76,70 (78,78)

Q 285,10>229,10 (-) 3,13e4

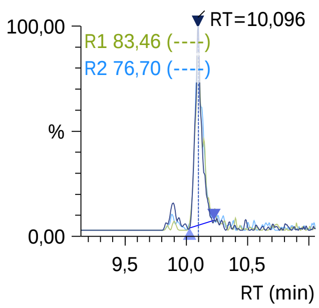

220 ppb001

Conc 211,3985

Area 131216

R#1 285,10>116,95 95,99 (95,08)

R#2 285,10>187,05 104,00 (78,78)

Q 285,10>229,10 (-) 2,84e4

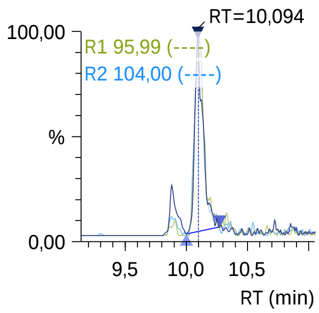

220ppb002

Conc 221,8198

Area 136759

R#1 285,10>116,95 99,61 (95,08)

R#2 285,10>187,05 77,24 (78,78)

Q 285,10>229,10 (-) 3,10e4

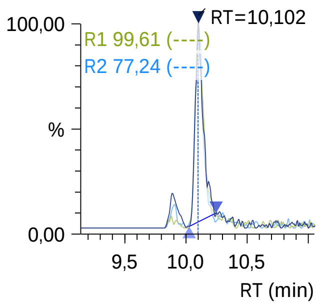

250 ppb001

Conc 254,6444

Area 154219

R#1 285,10>116,95 81,34 (95,08)

R#2 285,10>187,05 80,37 (78,78)

Q 285,10>229,10 (-) 3,63e4

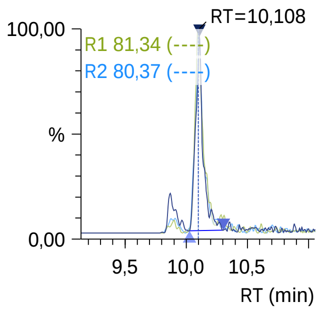

250 ppb002

Conc 317,0680

Area 187423

R#1 285,10>116,95 95,15 (95,08)

R#2 285,10>187,05 78,20 (78,78)

Q 285,10>229,10 (-) 3,60e4

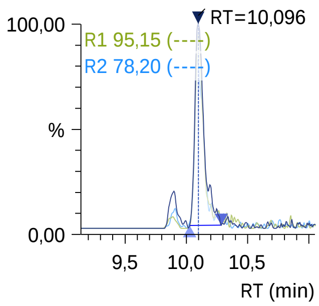

10H

Conc ----

Area ----

R#1 285,10>116,95 ---- (95,08)

R#2 285,10>187,05 ---- (78,78)

Q 285,10>229,10 (-) 3,94e2

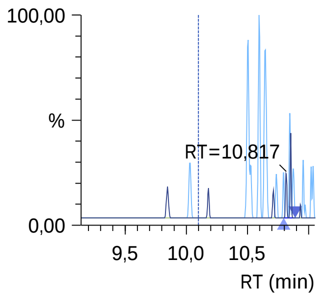

10M

Conc ----

Area ----

R#1 285,10>116,95 ---- (95,08)

R#2 285,10>187,05 ---- (78,78)

Q 285,10>229,10 (-) 5,00e2

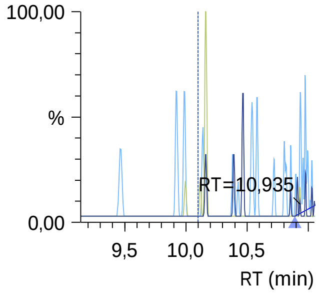

9H

Conc ----

Area ----

R#1 285,10>116,95 ---- (95,08)

R#2 285,10>187,05 ---- (78,78)

Q 285,10>229,10 (-) 9,61e2

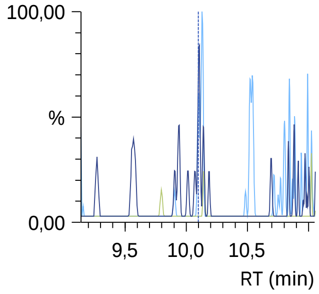

9M

Conc -25,9572

Area 4962

R#1 285,10>116,95 75,17 (95,08)

R#2 285,10>187,05 164,21 (78,78)

Q 285,10>229,10 (-) 1,61e3

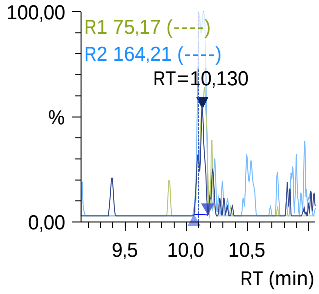

Compound: Ac. Resorcilico

Curve Fit: Linear | Weighting: None | Zero: Not Forced
Quantitative Method: External Standard
Q 153,20>108,90
R^2 = 0.9895954 R = 0.9947841
y = 1547,317x - 9885,575

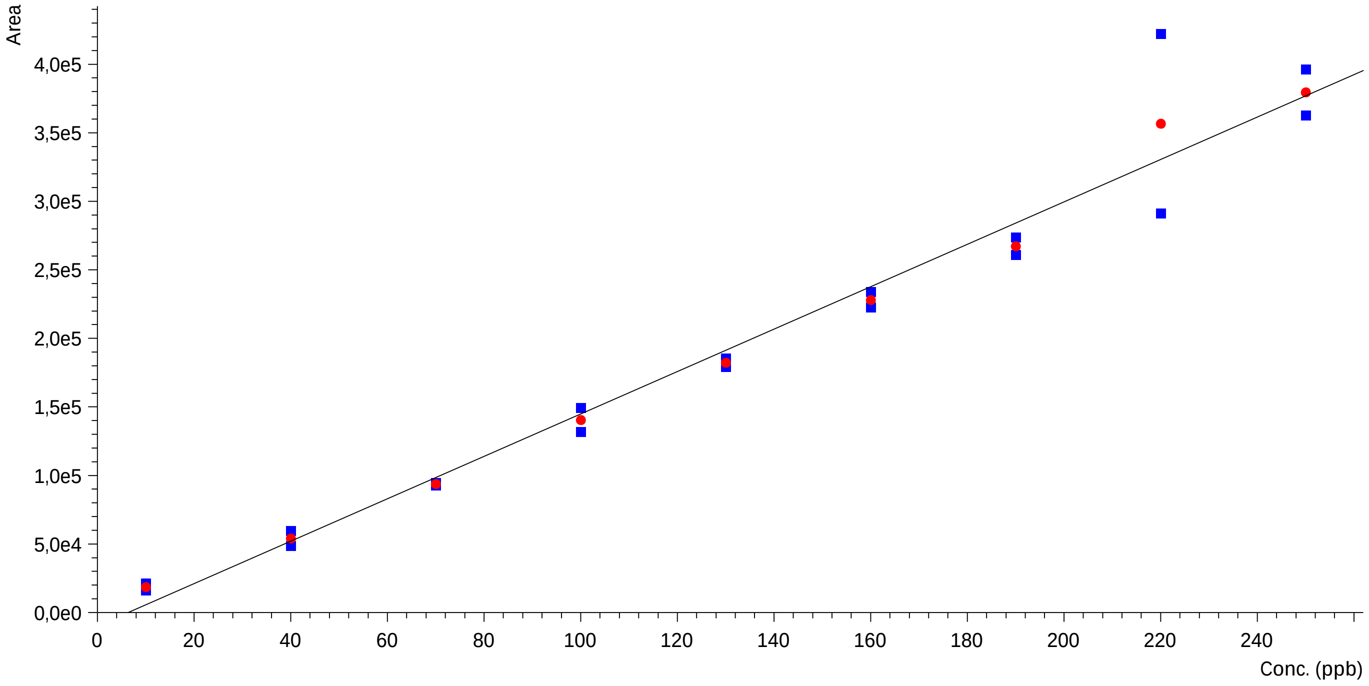

Sample ID	Area	Conc.	Sample Type	Level	Cal Point	Found RT	Ref 1 Actual Ratio
0	----	----	Ctrl.	0	----	----	----
10ppb001	21227	20,1072	Std.	1	Yes	7,570	3,52
10 ppb002	16340	16,9491	Std.	1	Yes	7,550	0,00
40 ppb001	59619	44,9192	Std.	2	Yes	7,555	0,22
40ppb002	48683	37,8520	Std.	2	Yes	7,567	0,00
70 ppb001	94752	67,6253	Std.	3	Yes	7,566	0,00
70ppb002	92833	66,3850	Std.	3	Yes	7,573	3,51
100 ppb001	149514	103,0165	Std.	4	Yes	7,560	0,00
100ppb002	131715	91,5136	Std.	4	Yes	7,574	0,00
130 ppb001	179313	122,2750	Std.	5	Yes	7,570	0,00
130ppb002	185276	126,1289	Std.	5	Yes	7,577	0,00
160 ppb001	222571	150,2319	Std.	6	Yes	7,573	0,00
160ppb002	233821	157,5029	Std.	6	Yes	7,560	0,41
190 ppb001	273792	183,3354	Std.	7	Yes	7,578	0,00
190ppb002	260989	175,0605	Std.	7	Yes	7,567	0,00
220 ppb001	291290	194,6438	Std.	8	Yes	7,573	0,00
220ppb002	422100	279,1836	Std.	8	Yes	7,577	0,01
250 ppb001	362894	240,9203	Std.	9	Yes	7,565	0,00
250 ppb002	396053	262,3497	Std.	9	Yes	7,577	0,20
10H	----	----	Unk.	----	----	----	----
10M	----	----	Unk.	----	----	----	----
9H	224400	151,4141	Unk.	----	----	7,580	1,09
9M	156961	107,8295	Unk.	----	----	7,582	0,11
Average	191207	129,9622				7,570	0,45
%RSD	63	59,6157				0,112	237,76
Maximum	422100	279,1836				7,582	3,52
Minimum	16340	16,9491				7,550	0,00
Std. Dev.	119883	77,4779				0,008	1,08

Compound: Ac. Resorcilico (continued)

0
Conc ----
Area ----
R#1 153,20>65,00 ---- (6,01)
R#2 153,20>66,95 ---- (2,97)
Q 153,20>108,90 (-) 1,55e3

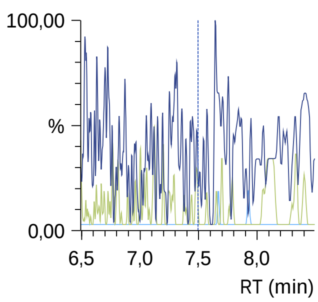

10ppb001
Conc 20,1072
Area 21227
R#1 153,20>65,00 3,52 (6,01)
R#2 153,20>66,95 0,00 (2,97)
Q 153,20>108,90 (-) 7,27e3

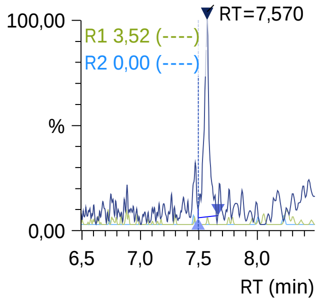

10 ppb002
Conc 16,9491
Area 16340
R#1 153,20>65,00 0,00 (6,01)
R#2 153,20>66,95 0,00 (2,97)
Q 153,20>108,90 (-) 3,90e3

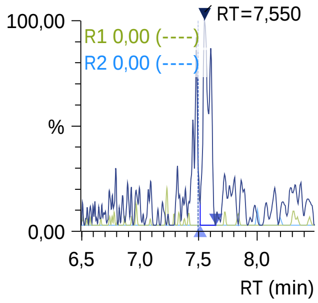

40 ppb001
Conc 44,9192
Area 59619
R#1 153,20>65,00 0,22 (6,01)
R#2 153,20>66,95 0,00 (2,97)
Q 153,20>108,90 (-) 1,81e4

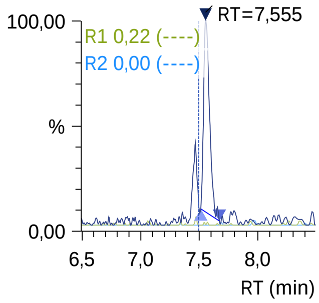

40ppb002
Conc 37,8520
Area 48683
R#1 153,20>65,00 0,00 (6,01)
R#2 153,20>66,95 0,00 (2,97)
Q 153,20>108,90 (-) 1,64e4

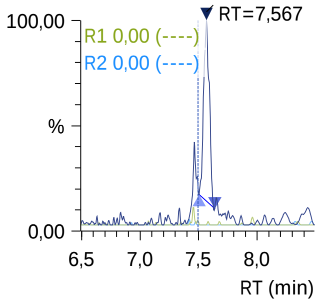

70 ppb001
Conc 67,6253
Area 94752
R#1 153,20>65,00 0,00 (6,01)
R#2 153,20>66,95 0,00 (2,97)
Q 153,20>108,90 (-) 2,89e4

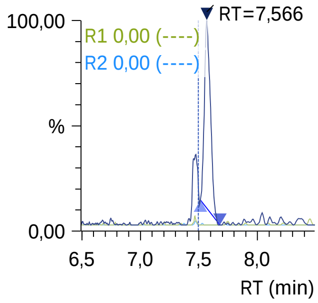

70ppb002
Conc 66,3850
Area 92833
R#1 153,20>65,00 3,51 (6,01)
R#2 153,20>66,95 0,00 (2,97)
Q 153,20>108,90 (-) 2,67e4

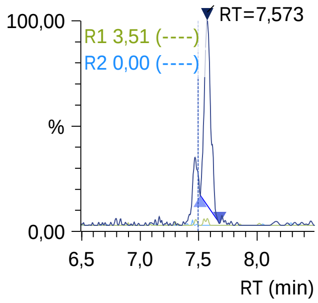

100 ppb001
Conc 103,0165
Area 149514
R#1 153,20>65,00 0,00 (6,01)
R#2 153,20>66,95 0,00 (2,97)
Q 153,20>108,90 (-) 4,34e4

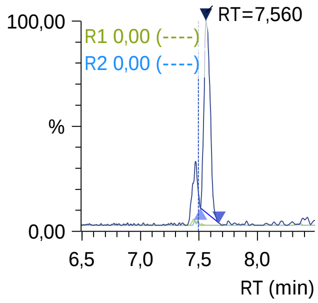

100ppb002
Conc 91,5136
Area 131715
R#1 153,20>65,00 0,00 (6,01)
R#2 153,20>66,95 0,00 (2,97)
Q 153,20>108,90 (-) 4,24e4

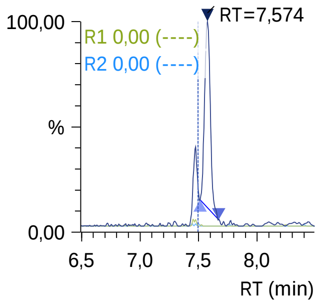

130 ppb001
Conc 122,2750
Area 179313
R#1 153,20>65,00 0,00 (6,01)
R#2 153,20>66,95 0,00 (2,97)
Q 153,20>108,90 (-) 5,07e4

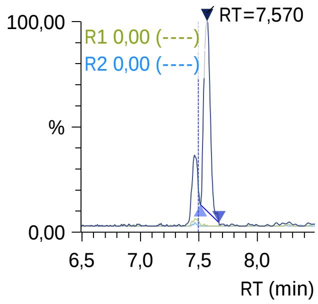

130ppb002
Conc 126,1289
Area 185276
R#1 153,20>65,00 0,00 (6,01)
R#2 153,20>66,95 0,00 (2,97)
Q 153,20>108,90 (-) 5,94e4

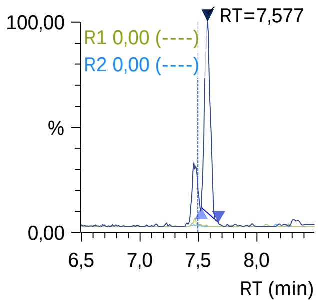

160 ppb001
Conc 150,2319
Area 222571
R#1 153,20>65,00 0,00 (6,01)
R#2 153,20>66,95 0,00 (2,97)
Q 153,20>108,90 (-) 7,46e4

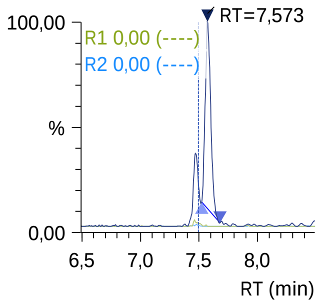

Compound: Ac. Resorcilico (continued)

160ppb002
Conc 157,5029
Area 233821
R#1 153,20>65,00 0,41 (6,01)
R#2 153,20>66,95 0,00 (2,97)
Q 153,20>108,90 (-) 6,79e4

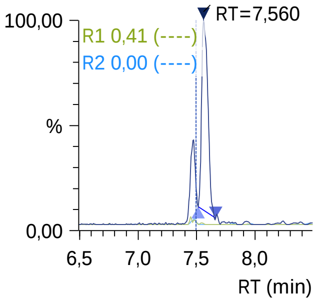

190 ppb001
Conc 183,3354
Area 273792
R#1 153,20>65,00 0,00 (6,01)
R#2 153,20>66,95 0,00 (2,97)
Q 153,20>108,90 (-) 9,01e4

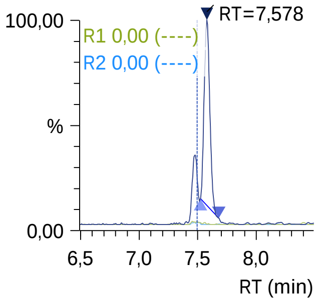

190ppb002
Conc 175,0605
Area 260989
R#1 153,20>65,00 0,00 (6,01)
R#2 153,20>66,95 0,00 (2,97)
Q 153,20>108,90 (-) 8,53e4

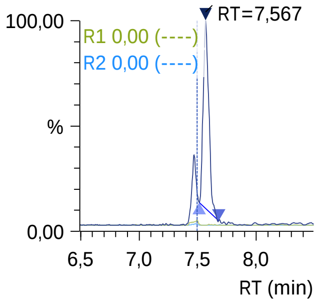

220 ppb001
Conc 194,6438
Area 291290
R#1 153,20>65,00 0,00 (6,01)
R#2 153,20>66,95 0,00 (2,97)
Q 153,20>108,90 (-) 9,88e4

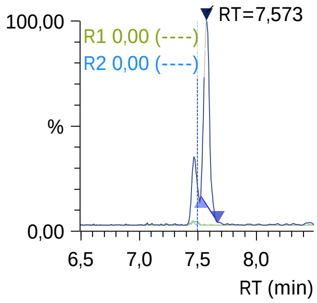

220ppb002
Conc 279,1836
Area 422100
R#1 153,20>65,00 0,01 (6,01)
R#2 153,20>66,95 0,00 (2,97)
Q 153,20>108,90 (-) 1,06e5

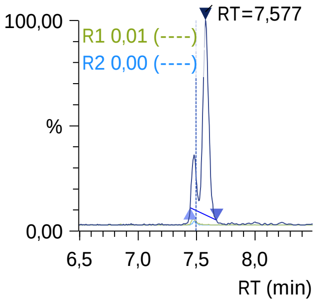

250 ppb001
Conc 240,9203
Area 362894
R#1 153,20>65,00 0,00 (6,01)
R#2 153,20>66,95 0,00 (2,97)
Q 153,20>108,90 (-) 1,19e5

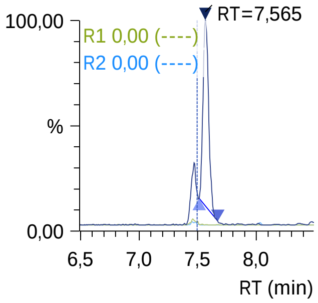

250 ppb002
Conc 262,3497
Area 396053
R#1 153,20>65,00 0,20 (6,01)
R#2 153,20>66,95 0,00 (2,97)
Q 153,20>108,90 (-) 1,28e5

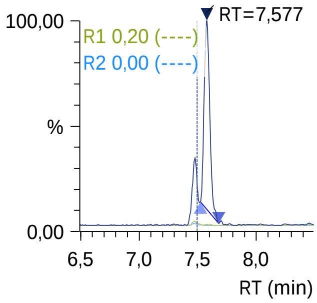

10H
Conc ----
Area ----
R#1 153,20>65,00 ---- (6,01)
R#2 153,20>66,95 ---- (2,97)
Q 153,20>108,90 (-) 1,35e4

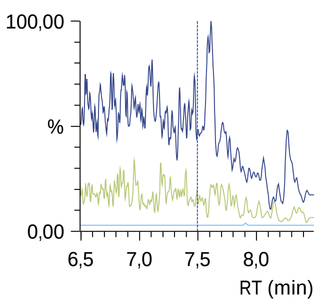

10M
Conc ----
Area ----
R#1 153,20>65,00 ---- (6,01)
R#2 153,20>66,95 ---- (2,97)
Q 153,20>108,90 (-) 1,32e4

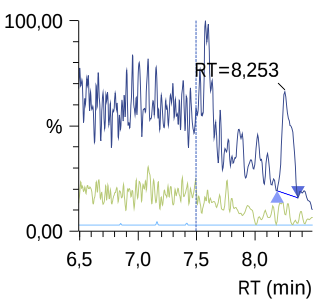

9H
Conc 151,4141
Area 224400
R#1 153,20>65,00 1,09 (6,01)
R#2 153,20>66,95 0,00 (2,97)
Q 153,20>108,90 (-) 5,55e4

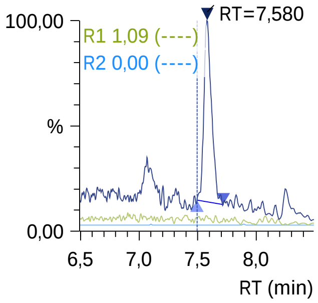

9M
Conc 107,8295
Area 156961
R#1 153,20>65,00 0,11 (6,01)
R#2 153,20>66,95 0,00 (2,97)
Q 153,20>108,90 (-) 5,36e4

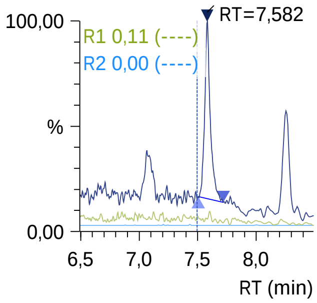

Compound: Ac. Nicotinico

Curve Fit: Linear | Weighting: None | Zero: Not Forced
Quantitative Method: External Standard
Q 122,40>77,90
R^2 = 0.9950711 R = 0.9975325
y = 208,0211x - 1554,629

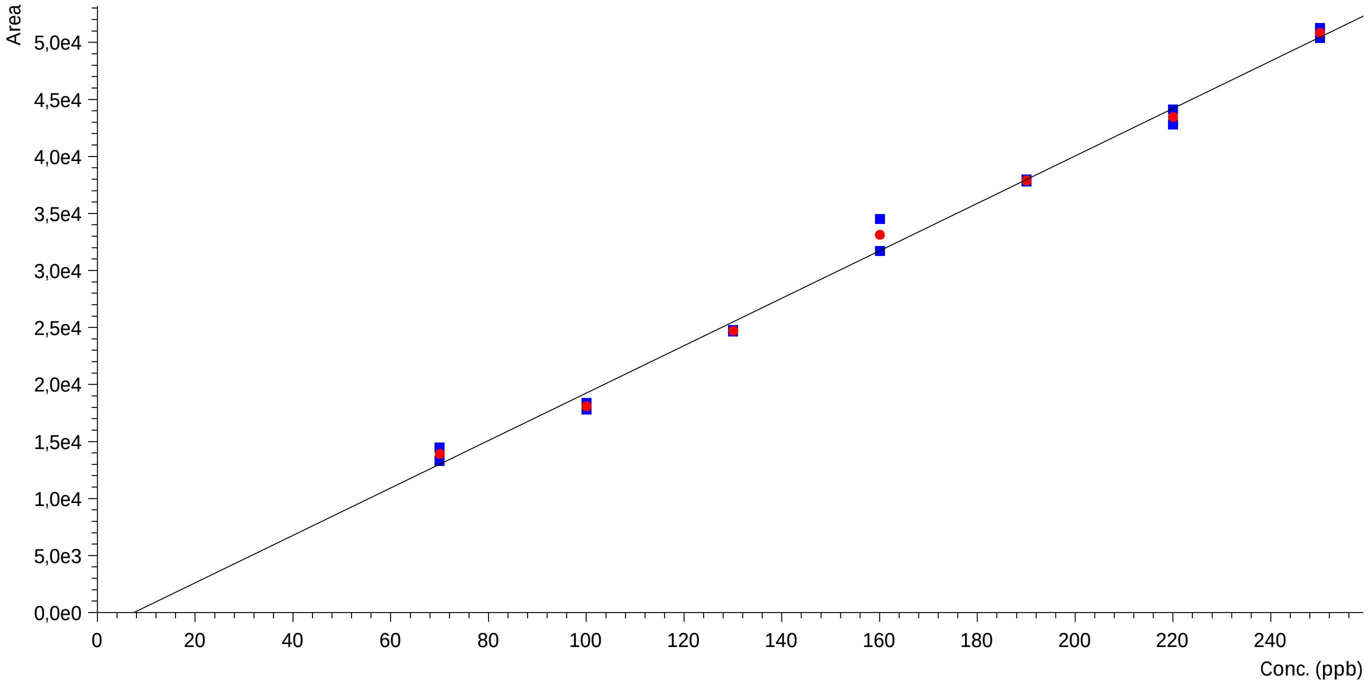

Sample ID	Area	Conc.	Sample Type	Level	Cal Point	Found RT	Ref 1 Actual Ratio
0	----	----	Ctrl.	0	----	----	----
10ppb001	----	----	Std.	1	No	----	----
10 ppb002	----	----	Std.	1	No	----	----
40 ppb001	----	----	Std.	2	No	----	----
40ppb002	----	----	Std.	2	No	----	----
70 ppb001	14512	77,2363	Std.	3	Yes	4,599	----
70ppb002	13302	71,4208	Std.	3	Yes	4,582	----
100 ppb001	17827	93,1703	Std.	4	Yes	4,618	----
100ppb002	18371	95,7872	Std.	4	Yes	4,620	----
130 ppb001	24811	126,7460	Std.	5	Yes	4,596	----
130ppb002	24655	125,9928	Std.	5	Yes	4,584	----
160 ppb001	31743	160,0664	Std.	6	Yes	4,594	----
160ppb002	34539	173,5082	Std.	6	Yes	4,598	----
190 ppb001	37985	190,0761	Std.	7	Yes	4,583	----
190ppb002	37817	189,2677	Std.	7	Yes	4,597	----
220 ppb001	42836	213,3936	Std.	8	Yes	4,595	----
220ppb002	44108	219,5100	Std.	8	Yes	4,590	----
250 ppb001	51282	253,9972	Std.	9	Yes	4,589	----
250 ppb002	50415	249,8275	Std.	9	Yes	4,638	----
10H	5634	34,5595	Unk.	----	----	4,651	----
10M	4526	29,2296	Unk.	----	----	4,636	----
9H	24459	125,0513	Unk.	----	----	4,648	----
9M	25213	128,6766	Unk.	----	----	4,639	----
Average	28002	142,0843				4,609	
%RSD	51	48,2958				0,517	
Maximum	51282	253,9972				4,651	
Minimum	4526	29,2296				4,582	
Std. Dev.	14275	68,6207				0,024	

Compound: Ac. Nicotinico (continued)

0
Conc ----
Area ----
Q 122,40>77,90 (-) 3,17e2

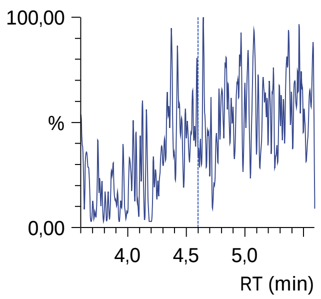

10ppb001
Conc ----
Area ----
Q 122,40>77,90 (-) 4,90e2

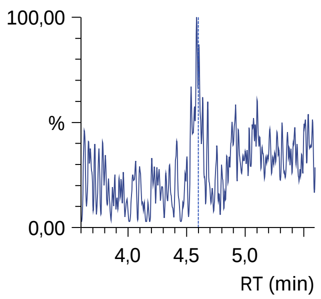

10 ppb002
Conc ----
Area ----
Q 122,40>77,90 (-) 4,49e2

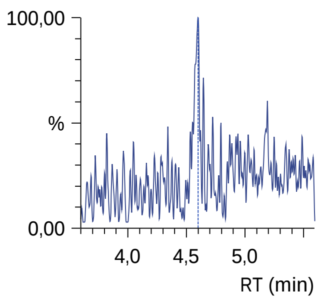

40 ppb001
Conc ----
Area ----
Q 122,40>77,90 (-) 1,50e3

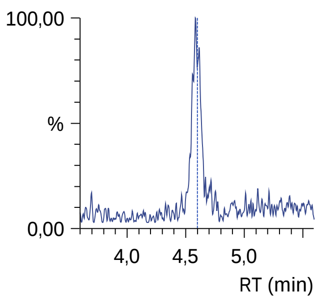

40ppb002
Conc ----
Area ----
Q 122,40>77,90 (-) 1,58e3

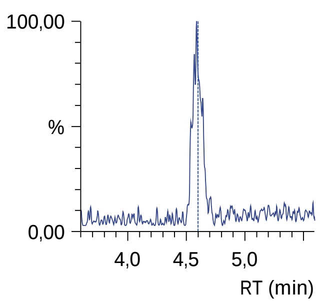

70 ppb001
Conc 77,2363
Area 14512
Q 122,40>77,90 (-) 2,63e3

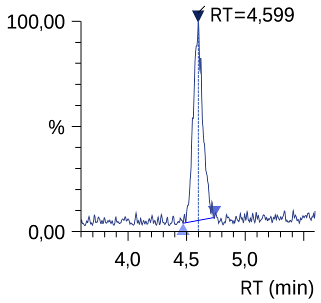

70ppb002
Conc 71,4208
Area 13302
Q 122,40>77,90 (-) 2,29e3

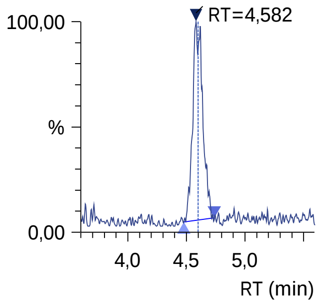

100 ppb001
Conc 93,1703
Area 17827
Q 122,40>77,90 (-) 3,07e3

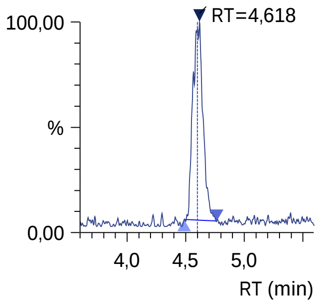

100ppb002
Conc 95,7872
Area 18371
Q 122,40>77,90 (-) 3,25e3

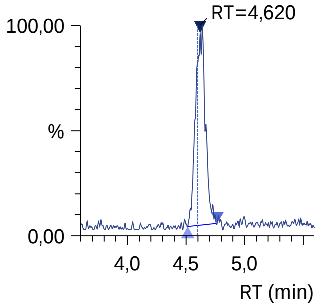

130 ppb001
Conc 126,7460
Area 24811
Q 122,40>77,90 (-) 4,25e3

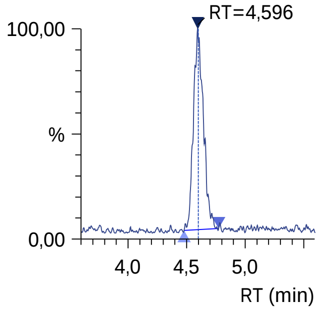

130ppb002
Conc 125,9928
Area 24655
Q 122,40>77,90 (-) 4,45e3

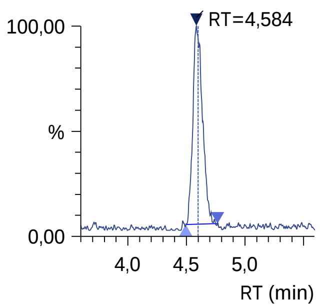

160 ppb001
Conc 160,0664
Area 31743
Q 122,40>77,90 (-) 5,59e3

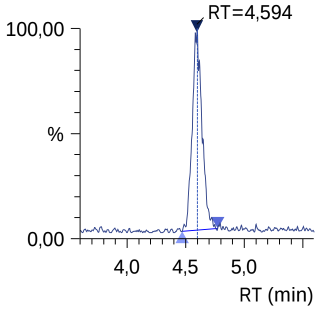

160ppb002
Conc 173,5082
Area 34539
Q 122,40>77,90 (-) 5,79e3

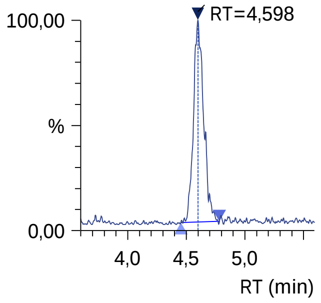

190 ppb001
Conc 190,0761
Area 37985
Q 122,40>77,90 (-) 6,80e3

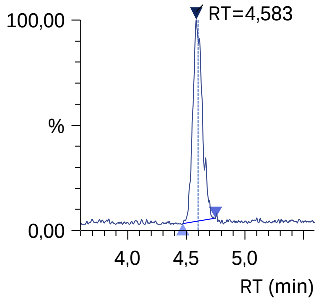

190ppb002
Conc 189,2677
Area 37817
Q 122,40>77,90 (-) 6,57e3

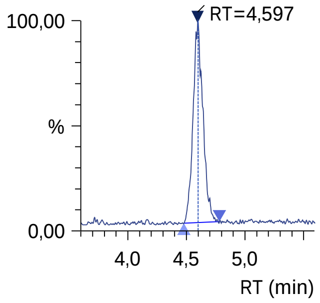

220 ppb001
Conc 213,3936
Area 42836
Q 122,40>77,90 (-) 7,43e3

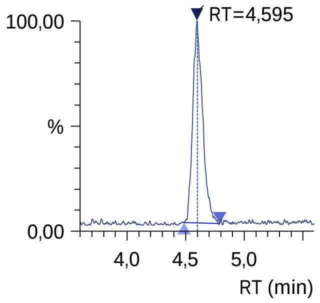

Compound: Ac. Nicotinico (continued)

220ppb002
Conc 219,5100
Area 44108
Q 122,40>77,90 (-)   7,45e3

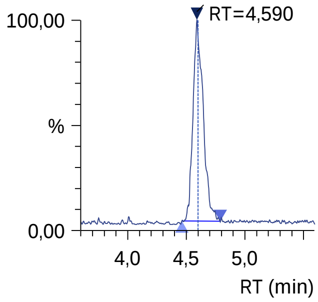

250 ppb001
Conc 253,9972
Area 51282
Q 122,40>77,90 (-)   8,38e3

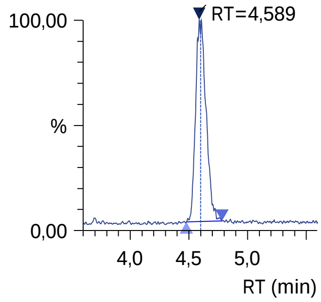

250 ppb002
Conc 249,8275
Area 50415
Q 122,40>77,90 (-)   8,54e3

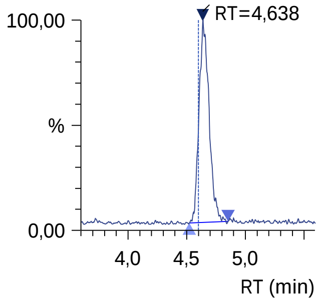

10H
Conc 34,5595
Area 5634
Q 122,40>77,90 (-)   1,25e3

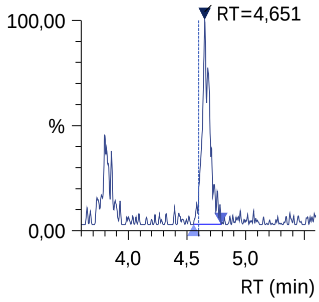

10M
Conc 29,2296
Area 4526
Q 122,40>77,90 (-)   9,26e2

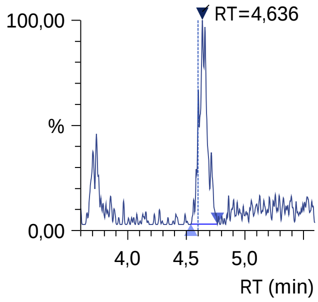

9H
Conc 125,0513
Area 24459
Q 122,40>77,90 (-)   4,31e3

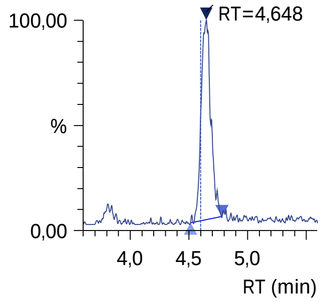

9M
Conc 128,6766
Area 25213
Q 122,40>77,90 (-)   4,26e3

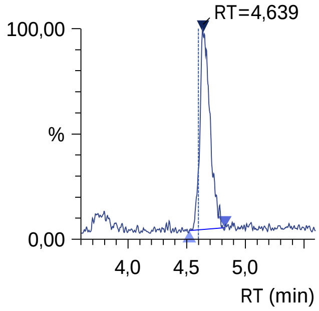

Compound: Ac. Fumarico

Curve Fit: Linear | Weighting: None | Zero: Not Forced
Quantitative Method: External Standard
Q 115,20>71,00
R^2 = 0.9895680 R = 0.9947703
y = 183,4024x - 2448,772

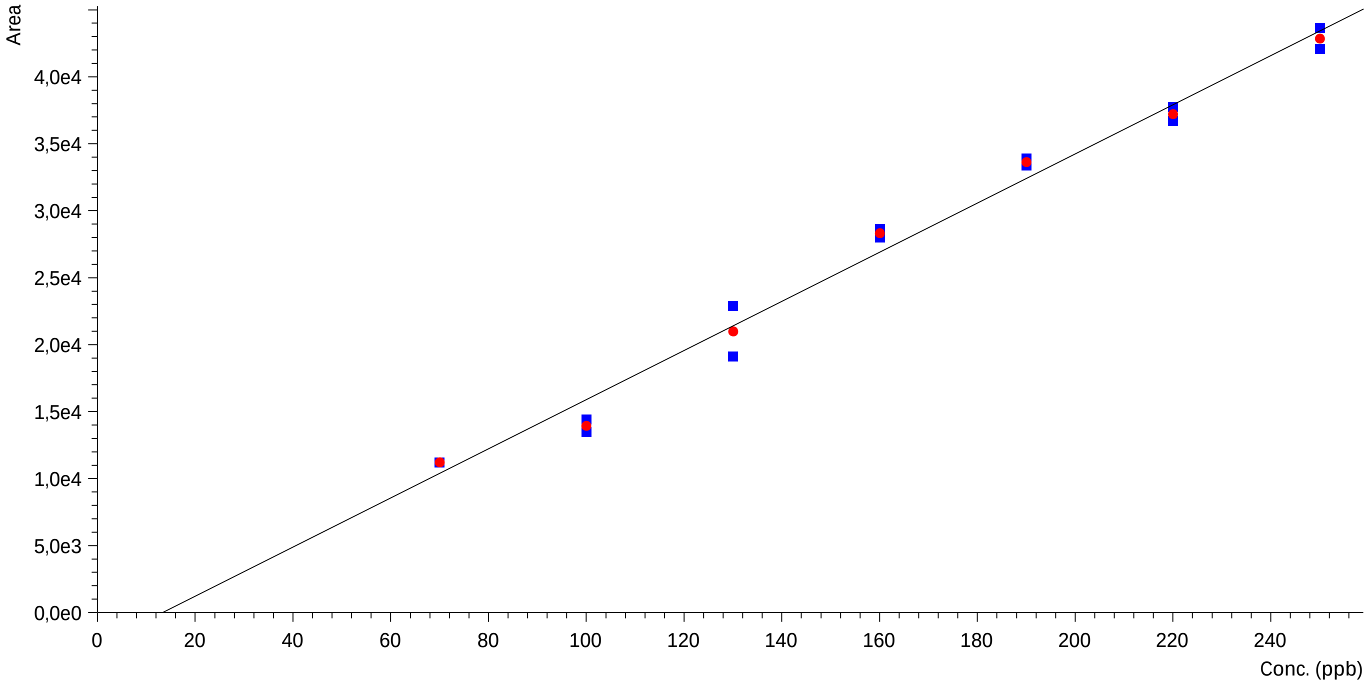

Sample ID	Area	Conc.	Sample Type	Level	Cal Point	Found RT	Ref 1 Actual Ratio
0	----	----	Ctrl.	0	----	----	----
10ppb001	----	----	Std.	1	No	----	----
10 ppb002	----	----	Std.	1	No	----	----
40 ppb001	----	----	Std.	2	No	----	----
40ppb002	----	----	Std.	2	No	----	----
70 ppb001	----	----	Std.	3	No	----	----
70ppb002	11239	74,6347	Std.	3	Yes	5,549	0,00
100 ppb001	13501	86,9655	Std.	4	Yes	5,550	5,32
100ppb002	14419	91,9701	Std.	4	Yes	5,548	0,00
130 ppb001	19119	117,5984	Std.	5	Yes	5,547	1,96
130ppb002	22890	138,1585	Std.	5	Yes	5,556	1,14
160 ppb001	28025	166,1586	Std.	6	Yes	5,545	8,43
160ppb002	28639	169,5039	Std.	6	Yes	5,553	5,14
190 ppb001	33377	195,3425	Std.	7	Yes	5,562	2,97
190ppb002	33895	198,1618	Std.	7	Yes	5,571	4,19
220 ppb001	37746	219,1591	Std.	8	Yes	5,551	1,99
220ppb002	36712	213,5229	Std.	8	Yes	5,551	0,19
250 ppb001	42082	242,8060	Std.	9	Yes	5,545	4,90
250 ppb002	43656	251,3833	Std.	9	Yes	5,602	2,24
10H	40072	231,8432	Unk.	----	----	5,513	6,65
10M	42133	243,0843	Unk.	----	----	5,487	10,22
9H	348081	1911,2594	Unk.	----	----	5,486	5,47
9M	401519	2202,6334	Unk.	----	----	5,510	6,43
Average	70418	397,3050				5,543	3,95
%RSD	164	158,4125				0,525	75,95
Maximum	401519	2202,6334				5,602	10,22
Minimum	11239	74,6347				5,486	0,00
Std. Dev.	115430	629,3808				0,029	3,00

Compound: Ac. Fumarico (continued)

0
Conc ----
Area ----
R#1 115,20>26,90 ---- (0,10)
Q 115,20>71,00 (-) 2,88e3

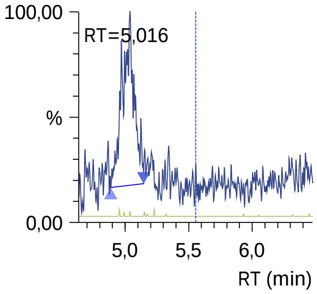

10ppb001
Conc ----
Area ----
R#1 115,20>26,90 ---- (0,10)
Q 115,20>71,00 (-) 1,05e3

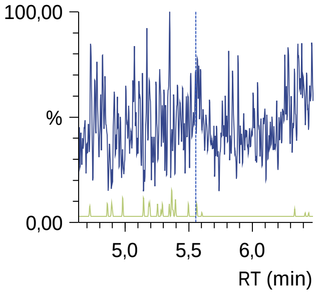

10 ppb002
Conc ----
Area ----
R#1 115,20>26,90 ---- (0,10)
Q 115,20>71,00 (-) 9,49e2

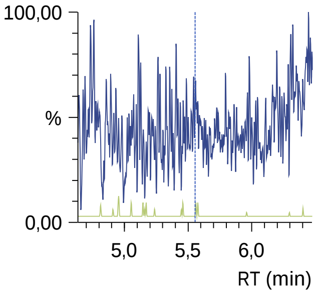

40 ppb001
Conc ----
Area ----
R#1 115,20>26,90 ---- (0,10)
Q 115,20>71,00 (-) 1,60e3

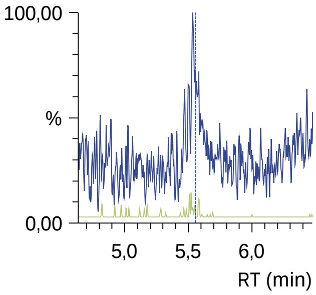

40ppb002
Conc ----
Area ----
R#1 115,20>26,90 ---- (0,10)
Q 115,20>71,00 (-) 1,64e3

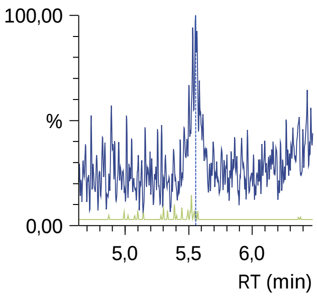

70 ppb001
Conc ----
Area ----
R#1 115,20>26,90 ---- (0,10)
Q 115,20>71,00 (-) 2,57e3

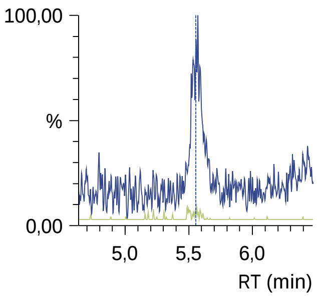

70ppb002
Conc 74,6347
Area 11239
R#1 115,20>26,90 0,00 (0,10)
Q 115,20>71,00 (-) 2,13e3

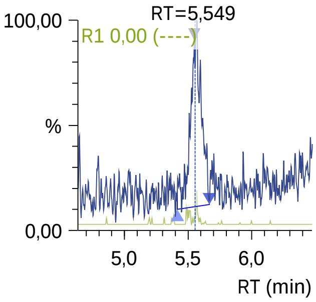

100 ppb001
Conc 86,9655
Area 13501
R#1 115,20>26,90 5,32 (0,10)
Q 115,20>71,00 (-) 2,54e3

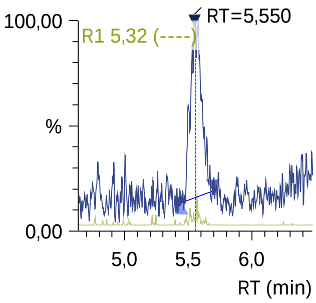

100ppb002
Conc 91,9701
Area 14419
R#1 115,20>26,90 0,00 (0,10)
Q 115,20>71,00 (-) 3,22e3

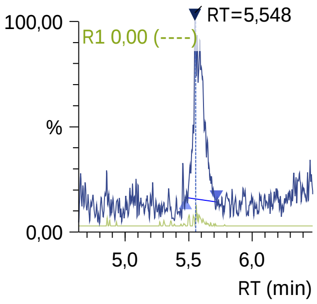

130 ppb001
Conc 117,5984
Area 19119
R#1 115,20>26,90 1,96 (0,10)
Q 115,20>71,00 (-) 3,79e3

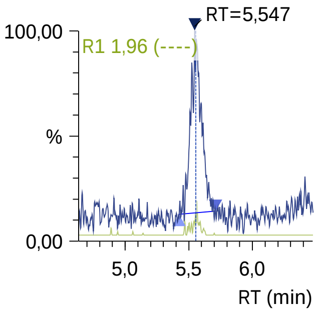

130ppb002
Conc 138,1585
Area 22890
R#1 115,20>26,90 1,14 (0,10)
Q 115,20>71,00 (-) 4,05e3

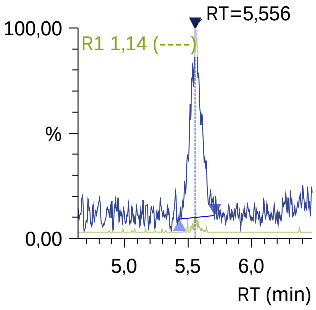

160 ppb001
Conc 166,1586
Area 28025
R#1 115,20>26,90 8,43 (0,10)
Q 115,20>71,00 (-) 4,75e3

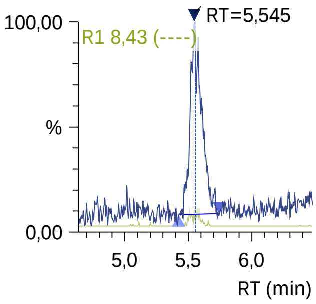

Compound: Ac. Fumarico (continued)

160ppb002
Conc 169,5039
Area 28639
R#1 115,20>26,90 5,14 (0,10)
Q 115,20>71,00 (-) 4,33e3

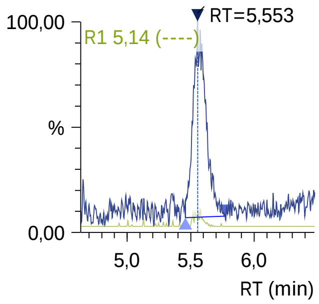

190 ppb001
Conc 195,3425
Area 33377
R#1 115,20>26,90 2,97 (0,10)
Q 115,20>71,00 (-) 5,23e3

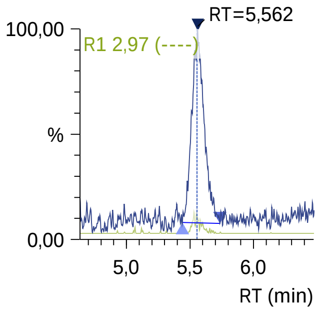

190ppb002
Conc 198,1618
Area 33895
R#1 115,20>26,90 4,19 (0,10)
Q 115,20>71,00 (-) 5,40e3

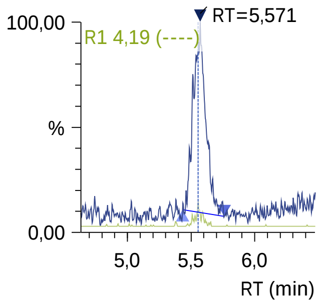

220 ppb001
Conc 219,1591
Area 37746
R#1 115,20>26,90 1,99 (0,10)
Q 115,20>71,00 (-) 6,13e3

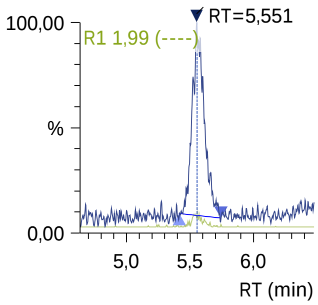

220ppb002
Conc 213,5229
Area 36712
R#1 115,20>26,90 0,19 (0,10)
Q 115,20>71,00 (-) 6,48e3

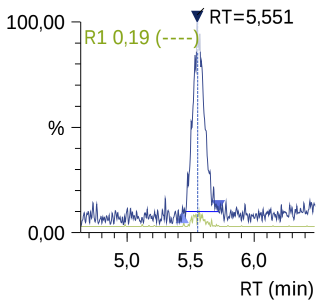

250 ppb001
Conc 242,8060
Area 42082
R#1 115,20>26,90 4,90 (0,10)
Q 115,20>71,00 (-) 7,63e3

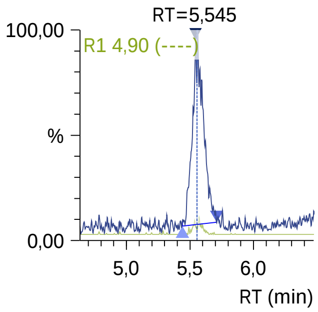

250 ppb002
Conc 251,3833
Area 43656
R#1 115,20>26,90 2,24 (0,10)
Q 115,20>71,00 (-) 7,15e3

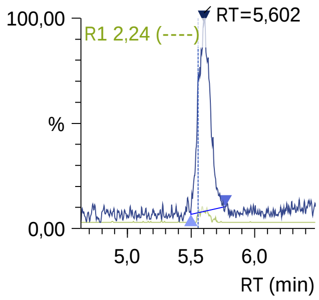

10H
Conc 231,8432
Area 40072
R#1 115,20>26,90 6,65 (0,10)
Q 115,20>71,00 (-) 6,57e3

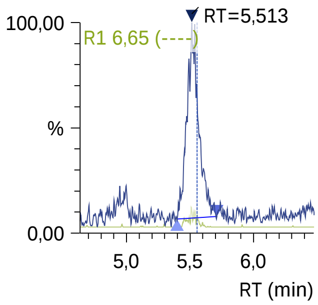

10M
Conc 243,0843
Area 42133
R#1 115,20>26,90 10,22 (0,10)
Q 115,20>71,00 (-) 7,49e3

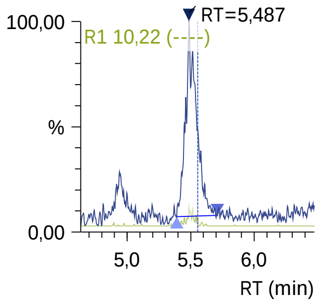

9H
Conc 1911,2594
Area 348081
R#1 115,20>26,90 5,47 (0,10)
Q 115,20>71,00 (-) 5,39e4

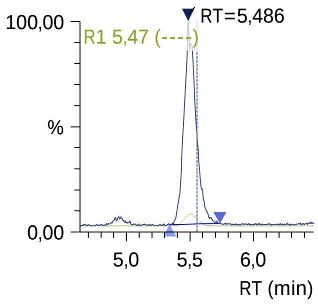

9M
Conc 2202,6334
Area 401519
R#1 115,20>26,90 6,43 (0,10)
Q 115,20>71,00 (-) 5,65e4

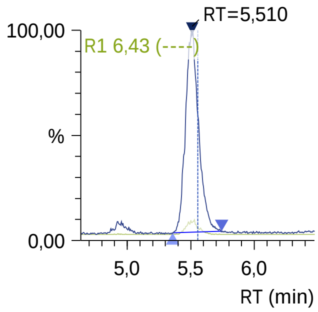

Compound: Teobromina

Curve Fit: Linear | Weighting: None | Zero: Not Forced
Quantitative Method: External Standard
Q 181,00>66,90
R^2 = 0.9969564 R = 0.9984770
y = 1161,701x - 3478,884

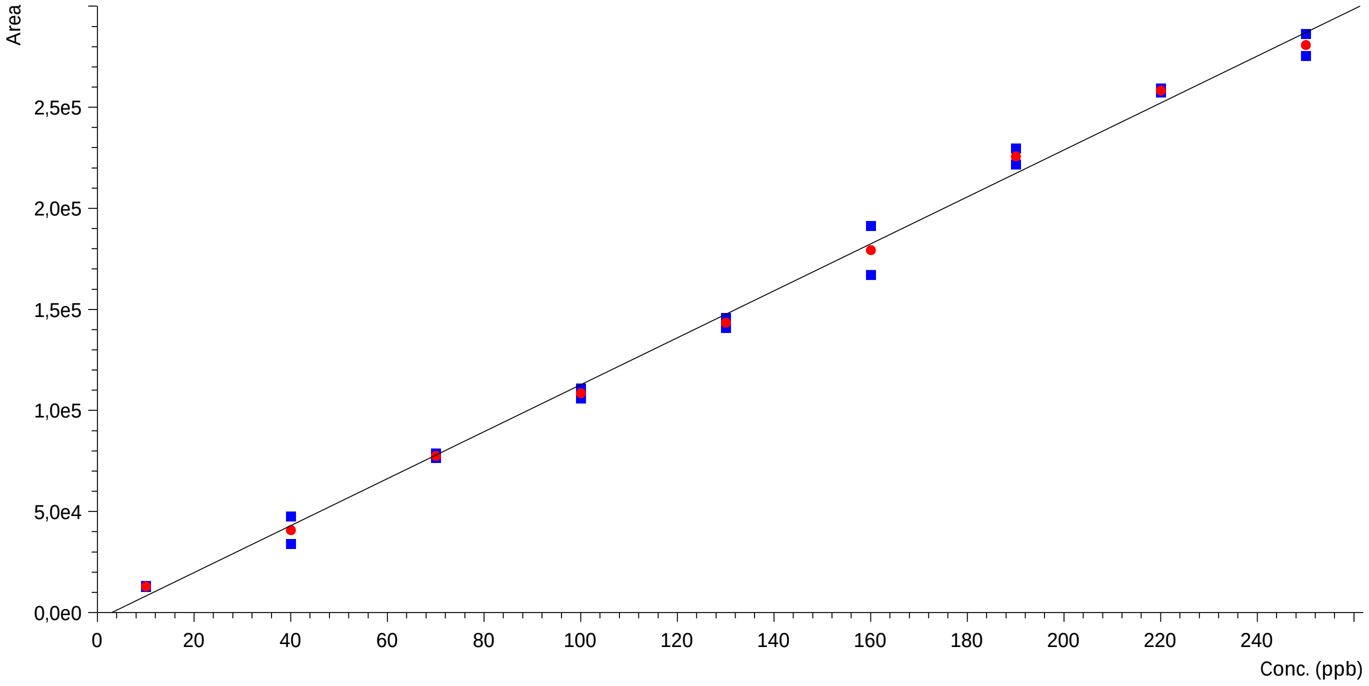

Sample ID	Area	Conc.	Sample Type	Level	Cal Point	Found RT	Ref 1 Actual Ratio
0	----	----	Ctrl.	0	----	----	----
10ppb001	12793	14,0071	Std.	1	Yes	7,548	10,20
10 ppb002	13275	14,4223	Std.	1	Yes	7,562	5,36
40 ppb001	47693	44,0488	Std.	2	Yes	7,544	0,00
40ppb002	34067	32,3199	Std.	2	Yes	7,534	2,53
70 ppb001	78633	70,6821	Std.	3	Yes	7,546	0,00
70ppb002	76475	68,8245	Std.	3	Yes	7,540	4,29
100 ppb001	106170	94,3868	Std.	4	Yes	7,542	5,37
100ppb002	111015	98,5567	Std.	4	Yes	7,550	2,54
130 ppb001	145815	128,5131	Std.	5	Yes	7,541	0,70
130ppb002	140986	124,3561	Std.	5	Yes	7,546	0,00
160 ppb001	167187	146,9100	Std.	6	Yes	7,552	2,91
160ppb002	191517	167,8539	Std.	6	Yes	7,550	3,15
190 ppb001	229825	200,8297	Std.	7	Yes	7,549	0,09
190ppb002	221896	194,0040	Std.	7	Yes	7,542	0,00
220 ppb001	259410	226,2965	Std.	8	Yes	7,549	0,51
220ppb002	257317	224,4949	Std.	8	Yes	7,549	0,57
250 ppb001	275302	239,9765	Std.	9	Yes	7,545	0,00
250 ppb002	286386	249,5172	Std.	9	Yes	7,550	1,19
10H	----	----	Unk.	----	----	----	----
10M	----	----	Unk.	----	----	----	----
9H	----	----	Unk.	----	----	----	----
9M	----	----	Unk.	----	----	----	----
Average	147542	130,0000				7,547	2,19
%RSD	63	61,5123				0,080	125,06
Maximum	286386	249,5172				7,562	10,20
Minimum	12793	14,0071				7,534	0,00
Std. Dev.	92897	79,9659				0,006	2,74

Compound: Teobromina (continued)

0
Conc ----
Area ----
R#1 181,00>106,90 ---- (36,37)
R#2 181,00>138,00 ---- (58,66)
Q 181,00>66,90 (+) 1,70e3

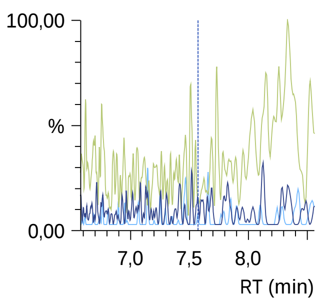

10ppb001
Conc 14,0071
Area 12793
R#1 181,00>106,90 10,20 (36,37)
R#2 181,00>138,00 38,10 (58,66)
Q 181,00>66,90 (+) 3,10e3

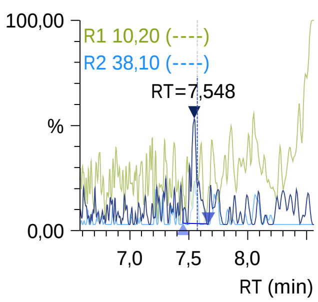

10 ppb002
Conc 14,4223
Area 13275
R#1 181,00>106,90 5,36 (36,37)
R#2 181,00>138,00 45,31 (58,66)
Q 181,00>66,90 (+) 4,30e3

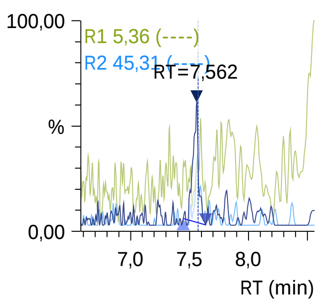

40 ppb001
Conc 44,0488
Area 47693
R#1 181,00>106,90 0,00 (36,37)
R#2 181,00>138,00 58,21 (58,66)
Q 181,00>66,90 (+) 1,35e4

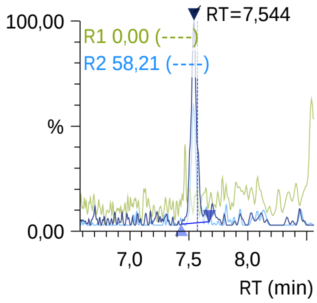

40ppb002
Conc 32,3199
Area 34067
R#1 181,00>106,90 2,53 (36,37)
R#2 181,00>138,00 77,93 (58,66)
Q 181,00>66,90 (+) 9,04e3

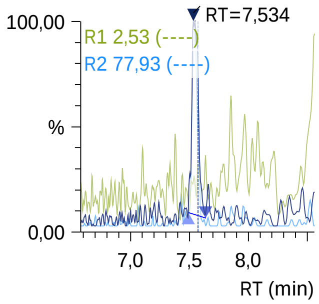

70 ppb001
Conc 70,6821
Area 78633
R#1 181,00>106,90 0,00 (36,37)
R#2 181,00>138,00 60,61 (58,66)
Q 181,00>66,90 (+) 2,05e4

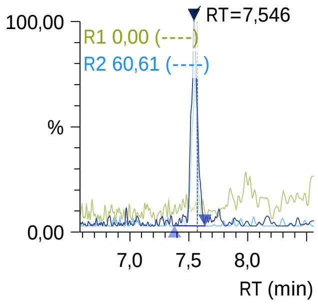

70ppb002
Conc 68,8245
Area 76475
R#1 181,00>106,90 4,29 (36,37)
R#2 181,00>138,00 66,15 (58,66)
Q 181,00>66,90 (+) 2,02e4

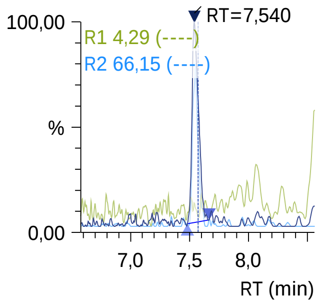

100 ppb001
Conc 94,3868
Area 106170
R#1 181,00>106,90 5,37 (36,37)
R#2 181,00>138,00 53,09 (58,66)
Q 181,00>66,90 (+) 2,93e4

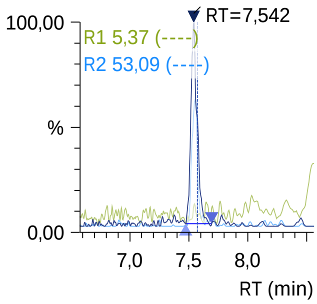

100ppb002
Conc 98,5567
Area 111015
R#1 181,00>106,90 2,54 (36,37)
R#2 181,00>138,00 51,90 (58,66)
Q 181,00>66,90 (+) 3,38e4

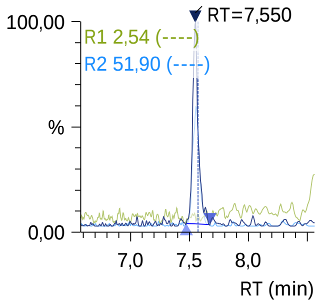

130 ppb001
Conc 128,5131
Area 145815
R#1 181,00>106,90 0,70 (36,37)
R#2 181,00>138,00 59,37 (58,66)
Q 181,00>66,90 (+) 3,86e4

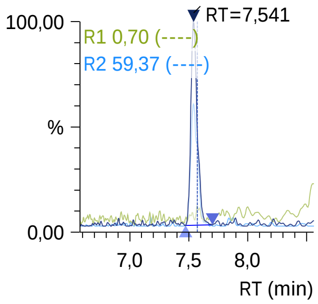

130ppb002
Conc 124,3561
Area 140986
R#1 181,00>106,90 0,00 (36,37)
R#2 181,00>138,00 74,49 (58,66)
Q 181,00>66,90 (+) 3,91e4

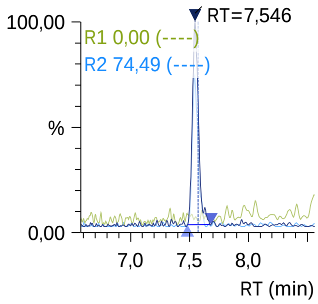

160 ppb001
Conc 146,9100
Area 167187
R#1 181,00>106,90 2,91 (36,37)
R#2 181,00>138,00 68,74 (58,66)
Q 181,00>66,90 (+) 4,92e4

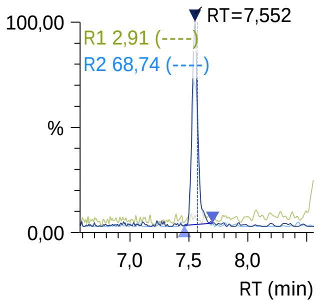

Compound: Teobromina (continued)

160ppb002

Conc 167,8539
Area 191517
R#1 181,00>106,90 3,15 (36,37)
R#2 181,00>138,00 71,50 (58,66)
Q 181,00>66,90 (+) 5,16e4

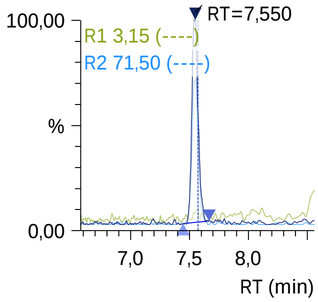

190 ppb001

Conc 200,8297
Area 229825
R#1 181,00>106,90 0,09 (36,37)
R#2 181,00>138,00 68,89 (58,66)
Q 181,00>66,90 (+) 6,20e4

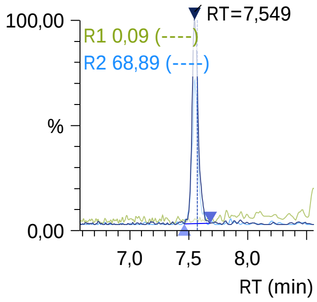

190ppb002

Conc 194,0040
Area 221896
R#1 181,00>106,90 0,00 (36,37)
R#2 181,00>138,00 59,31 (58,66)
Q 181,00>66,90 (+) 6,26e4

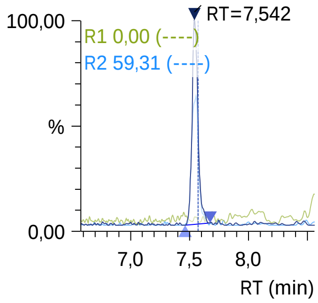

220 ppb001

Conc 226,2965
Area 259410
R#1 181,00>106,90 0,51 (36,37)
R#2 181,00>138,00 70,47 (58,66)
Q 181,00>66,90 (+) 6,87e4

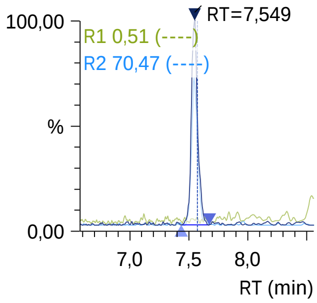

220ppb002

Conc 224,4949
Area 257317
R#1 181,00>106,90 0,57 (36,37)
R#2 181,00>138,00 78,54 (58,66)
Q 181,00>66,90 (+) 7,10e4

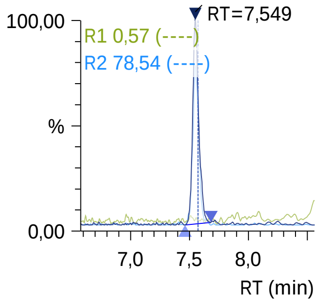

250 ppb001

Conc 239,9765
Area 275302
R#1 181,00>106,90 0,00 (36,37)
R#2 181,00>138,00 84,77 (58,66)
Q 181,00>66,90 (+) 6,78e4

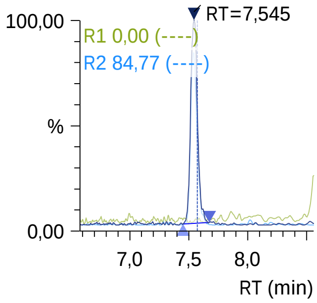

250 ppb002

Conc 249,5172
Area 286386
R#1 181,00>106,90 1,19 (36,37)
R#2 181,00>138,00 71,51 (58,66)
Q 181,00>66,90 (+) 8,05e4

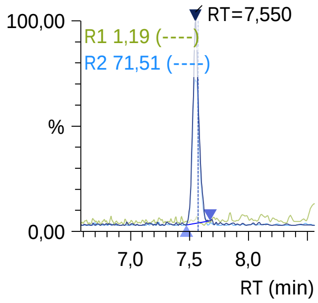

10H

Conc ----
Area ----
R#1 181,00>106,90 ---- (36,37)
R#2 181,00>138,00 ---- (58,66)
Q 181,00>66,90 (+) 6,81e2

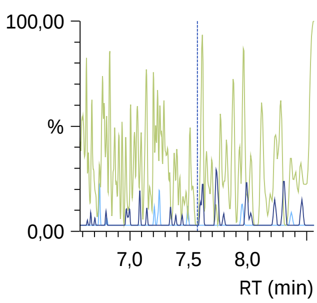

10M

Conc ----
Area ----
R#1 181,00>106,90 ---- (36,37)
R#2 181,00>138,00 ---- (58,66)
Q 181,00>66,90 (+) 1,77e3

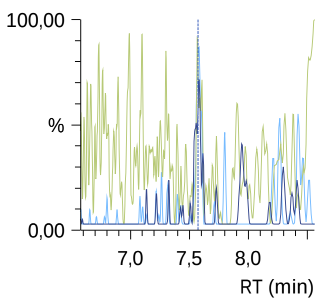

9H

Conc ----
Area ----
R#1 181,00>106,90 ---- (36,37)
R#2 181,00>138,00 ---- (58,66)
Q 181,00>66,90 (+) 9,91e2

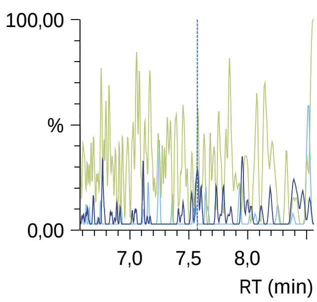

9M

Conc ----
Area ----
R#1 181,00>106,90 ---- (36,37)
R#2 181,00>138,00 ---- (58,66)
Q 181,00>66,90 (+) 2,51e3

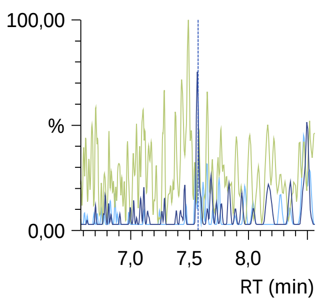

Workbook name	C:\Users\admin\Documents\Litesizer\Resultados Gerais\AuNP.apkw		
Measurement name	AuNP - Raiz Ginseng		
Measurement mode	Particle size		
**Comment**			
**Expert advices**			
**Results**	Hydrodynamic diameter	91,07081625	nm
	Polydispersity index	23,05541315	%
	Intercept g1²	0,657857782	
	Baseline	1,000256103	
	Mean intensity	308,9055556	kcps
	Absolute intensity	995081,1921	kcps
	Fit error	4,2385E-05	
	Diffusion coefficient	5,386755398	
Peak analysis volume	Peak volume 1	10,03774319	nm
	Area volume 1	96,05	%
	Standard deviation volume 1	1,38634	nm
	Peak volume 2	72,61658124	nm
	Area volume 2	3,95	%
	Standard deviation volume 2	18,44109	nm
	Peak volume 3		
	Area volume 3		
	Standard deviation volume 3		
Peak analysis intensity	Peak intensity 1	103,2026683	nm
	Area intensity 1	96,10	%
	Standard deviation intensity 1	37,91713	nm
	Peak intensity 2	10,77559977	nm
	Area intensity 2	3,90	%
	Standard deviation intensity 2	1,58178	nm
	Peak intensity 3		
	Area intensity 3		
	Standard deviation intensity 3		
Peak analysis number	Peak number 1	9,454185205	nm
	Area number 1	100,00	%
	Standard deviation number 1	1,12882	nm
	Peak number 2		

Particle diameter
[nm]
0,20947418
0,227146672
0,246310121
0,267090313
0,289623646
0,314058025
0,340553834
0,369284989
0,400440074
0,434223589
0,470857283
0,510581614
0,553657326
0,600367163
0,65101772
0,705941461
0,765498896
0,830080952
0,900111535
0,976050317
1,058395747
1,14768833
1,244514167
1,349508811
1,463361429
1,586819333
1,720692883
1,865860805

	Area number 2			2,02327596
	Standard deviation number 2			2,193971596
	Peak number 3			2,379068135
	Area number 3			2,579780523
	Standard deviation number 3			2,797426205
D values	D10 volume	7,104420879	nm	3,033433777
	D50 volume	9,460990376	nm	3,289352356
	D90 volume	12,28676194	nm	3,566861754
	D10 intensity	53,2403214	nm	3,867783501
	D50 intensity	93,17979305	nm	4,194092803
	D90 intensity	146,6140899	nm	4,547931505
	D10 number	4,025899712	nm	4,931622152
	D50 number	8,806931035	nm	5,347683232
	D90 number	10,87518362	nm	5,798845708
	Undersize span volume (D90-D10)/D50	0,547758834		6,288070943
	Undersize span intensity (D90-D10)/D50	1,002081733		6,818570139
	Undersize span number (D90-D10)/D50	0,777715175		7,393825414
				8,017612658
**Automatic values**	Filter optical density	3,508032799		8,694026316
	Focus position	-0,00312829	mm	9,427506268
	Auto run criteria	186,59579	%	10,22286697
	Transmittance	73,32146168	%	11,08532904
	Angle used	BackScatter		12,02055356
	Processed runs	6		13,03467921
				14,13436255
**Input parameters**	**General**			15,32682174
	Measurement cell	Disposable		16,6198839
	Measurement angle	BackScatter		18,0220365
	Target temperature	25	°C	19,54248307
	Equilibration time	0h 00m 30s		21,19120359
	Analysis model	General		22,97902
	Cumulant model	Advanced		24,91766729
				27,01987042
	**Quality**			29,29942797
	Mode	Automatic		31,77130259
	Max. number of runs	60		34,45171931

	Measurement time	0h 00m 10s		37,35827198
				40,51003878
	**Filter**			43,92770745
	Mode	Automatic		47,63371104
	Optical density	0	mm	51,65237521
				56,01007788
	**Focus**			60,73542236
	Mode	Automatic		65,8594251
	Position	0	mm	71,4157193
				77,44077564
	**Material**			83,97414169
	Name	Silver		91,0587015
	Refractive index	0.1590		98,74095706
	Absorption coefficient	4.3000	1/m	107,0713335
				116,1045103
	**Solvent**			125,8997798
	Name	Water		136,5214367
	Refractive index	1,330267336		148,0391999
	Viscosity	0,000890308	Pa.s	160,5286704
	Relative permittivity	78,36815402		174,0718272
				188,7575656
**Additional information**	**Measurement**			204,6822804
	User	admin		221,9504993
	Start time	11/04/2023 15:42		240,6755682
	Software version	2.26.3		260,9803956
	Computer name	DESKTOP-9DSA0RN		282,9982595
				306,8736818
	**Instrument**			332,7633772
	Type	Litesizer 100		360,8372819
	Serial number	83641862		391,2796687
				424,290357
	**Module**			460,086024
	Type	BM10		498,901627
	Serial number	83620256		540,9919461
				586,6332557
				636,1251386

689,7924521
747,9874605
811,0921472
879,5207219
953,7223395
1034,184049
1121,433988
1216,044852
1318,637654
1429,885797
1550,519498
1681,330577
1823,17766
1976,991809
2143,782638
2324,644937
2520,765859
2733,432713
2964,041412
3214,105637
3485,266772
3779,304679
4098,14938
4443,893722
4818,80712
5225,350451
5666,192204
6144,225998
6662,589576
7224,685399
7834,202982
8495,143105
9211,844082
9989,010232
10831,74276
11745,57323

12736,49989
13811,02704
14976,20772
16239,68999
17609,76716
19095,43223

Size distribution function

Size distribution Intensity weighted [%]	Size distribution Volume weighted [%]	Size distribution Number weighted [%]	Undersize Intensity weighted [%]	Undersize Volume weighted [%]	Undersize Number weighted [%]
0	0	0	0	0	0
0	0	0	0	0	0
0	0	0	0	0	0
0	0	0	0	0	0
0	0	0	0	0	0
0	0	0	0	0	0
0	0	0	0	0	0
0	0	0	0	0	0
0	0	0	0	0	0
0	0	0	0	0	0
0	0	0	0	0	0
0	0	0	0	0	0
0	0	0	0	0	0
0	0	0	0	0	0
0	0	0	0	0	0
0	0	0	0	0	0
0	0	0	0	0	0
0	0	0	0	0	0
0	0	0	0	0	0
0	0	0	0	0	0
0	0	0	0	0	0
0	0	0	0	0	0
0	0	0	0	0	0
0	0	0	0	0	0
0	0	0	0	0	0
0	0	0	0	0	0
0	0	0	0	0	0
0	0	0	0	0	0

0	0	0	0	0	0
0	0	0	0	0	0
0	0	0	0	0	0
0	0	0	0	0	0
0	0	0	0	0	0
0	0	0	0	0	0
0	0	0	0	0	0
0	0	0	0	0	0
0	0	0	0	0	0
0	0	0	0	0	0
0	0	0	0	0	0
0	0	0	0	0	0
0	0	0	0	0	0
0	0	0	0	0	0
0	0	0	0	0	0
0	0	0	0	0	0
0	0	0	0	0	0
0,21555803	11,32443631	20,45929735	0,21555803	11,32443631	20,45929735
0,450504598	18,49946988	26,21233838	0,666062627	29,82390619	46,67163573
0,606388246	19,45755028	21,62255475	1,272450873	49,28145647	68,29419048
0,680923045	17,06783676	14,87543541	1,953373919	66,34929323	83,16962589
0,674958635	13,21181433	9,030795952	2,628332553	79,56110756	92,20042184
0,592667682	9,05647658	4,855066669	3,221000235	88,61758414	97,05548851
0,441615281	5,266338381	2,214201048	3,662615516	93,88392252	99,26968956
0,2326925	2,164779635	0,713829262	3,895308016	96,04870216	99,98351882
0	0	0	3,895308016	96,04870216	99,98351882
0	0	0	3,895308016	96,04870216	99,98351882
0	0	0	3,895308016	96,04870216	99,98351882
0	0	0	3,895308016	96,04870216	99,98351882
0	0	0	3,895308016	96,04870216	99,98351882
0	0	0	3,895308016	96,04870216	99,98351882
0	0	0	3,895308016	96,04870216	99,98351882
0	0	0	3,895308016	96,04870216	99,98351882
0	0	0	3,895308016	96,04870216	99,98351882
0	0	0	3,895308016	96,04870216	99,98351882
0	0	0	3,895308016	96,04870216	99,98351882

0	0	0	3,895308016	96,04870216	99,98351882
0,158711463	0,056489188	0,000791201	4,054019479	96,10519135	99,98431002
0,818491795	0,225956503	0,002482096	4,872511274	96,33114785	99,98679212
1,566502785	0,335263398	0,002888366	6,439014059	96,66641125	99,98968048
2,381332003	0,394917541	0,002668363	8,820346062	97,06132879	99,99234884
3,237251471	0,415793343	0,002203375	12,05759753	97,47712213	99,99455222
4,105067488	0,408145673	0,001696283	16,16266502	97,88526781	99,9962485
4,953115888	0,381015314	0,001241932	21,11578091	98,26628312	99,99749044
5,748351027	0,341938169	0,000874129	26,86413193	98,60822129	99,99836456
6,457472149	0,296875652	0,000595216	33,32160408	98,90509694	99,99895978
7,048030307	0,250294314	0,000393571	40,36963439	99,15539125	99,99935335
7,489462721	0,205335753	0,000253227	47,85909711	99,36072701	99,99960658
7,754009062	0,164031209	0,000158651	55,61310617	99,52475822	99,99976523
7,817474493	0,127527711	9,67374E-05	63,43058067	99,65228593	99,99986197
7,659816106	0,096246695	5,72595E-05	71,09039677	99,74853262	99,99991923
7,265541313	0,070308798	3,28053E-05	78,35593809	99,81884142	99,99995203
6,623917621	0,053568328	1,96027E-05	84,97985571	99,87240975	99,99997163
5,729002302	0,042969038	1,2332E-05	90,70885801	99,91537879	99,99998397
4,579507584	0,036763914	8,2751E-06	95,28836559	99,9521427	99,99999224
3,178522049	0,029732157	5,24868E-06	98,46688764	99,98187486	99,99999749
1,533112357	0,018125143	2,50944E-06	100	100	100
0	0	0	100	100	100
0	0	0	100	100	100
0	0	0	100	100	100
0	0	0	100	100	100
0	0	0	100	100	100
0	0	0	100	100	100
0	0	0	100	100	100
0	0	0	100	100	100
0	0	0	100	100	100
0	0	0	100	100	100
0	0	0	100	100	100
0	0	0	100	100	100
0	0	0	100	100	100
0	0	0	100	100	100
0	0	0	100	100	100

0	0	0	100	100	100
0	0	0	100	100	100
0	0	0	100	100	100
0	0	0	100	100	100
0	0	0	100	100	100
0	0	0	100	100	100
0	0	0	100	100	100
0	0	0	100	100	100
0	0	0	100	100	100
0	0	0	100	100	100
0	0	0	100	100	100
0	0	0	100	100	100
0	0	0	100	100	100
0	0	0	100	100	100
0	0	0	100	100	100
0	0	0	100	100	100
0	0	0	100	100	100
0	0	0	100	100	100
0	0	0	100	100	100
0	0	0	100	100	100
0	0	0	100	100	100
0	0	0	100	100	100
0	0	0	100	100	100
0	0	0	100	100	100
0	0	0	100	100	100
0	0	0	100	100	100
0	0	0	100	100	100
0	0	0	100	100	100
0	0	0	100	100	100
0	0	0	100	100	100
0	0	0	100	100	100
0	0	0	100	100	100
0	0	0	100	100	100
0	0	0	100	100	100
0	0	0	100	100	100
0	0	0	100	100	100

0	0	0	100	100	100
0	0	0	100	100	100
0	0	0	100	100	100
0	0	0	100	100	100
0	0	0	100	100	100
0	0	0	100	100	100

	**Correlation function**	
Delay time [s]	g2	Cumulant fit Advanced
0,0000002	1,661332424	
0,00000022	1,670121363	
0,00000024	1,666106945	
0,00000026	1,662397309	
0,00000028	1,667380364	
0,0000003	1,67227814	
0,00000032	1,668358325	
0,00000036	1,668674002	
0,0000004	1,669576721	
0,00000044	1,659028814	
0,00000048	1,667472218	1,655939631
0,00000052	1,669627621	1,655758768
0,00000056	1,665246404	1,655577955
0,0000006	1,664243812	1,655397191
0,00000064	1,661245343	1,655216478
0,00000072	1,660054858	1,6548552
0,0000008	1,661887962	1,654494122
0,00000088	1,660246649	1,654133243
0,00000096	1,66144165	1,653772563
0,00000104	1,656928079	1,653412082
0,00000112	1,657069394	1,6530518
0,0000012	1,663076914	1,652691716
0,00000128	1,656685119	1,652331831
0,00000144	1,655614147	1,651612657
0,0000016	1,654817638	1,650894276
0,00000176	1,652742232	1,650176687
0,00000192	1,655574576	1,64945989
0,00000208	1,653841374	1,648743883

**Intensity trace**	
Time [s]	Intensity [kcounts/s]
0,1	323,39
0,2	283,17
0,3	283,35
0,4	313,26
0,5	309,7
0,6	306,4
0,7	285,11
0,8	285,89
0,9	307,27
1	298,85
1,1	301,04
1,2	316,58
1,3	309,61
1,4	329,68
1,5	322,06
1,6	344,78
1,7	329,03
1,8	310,56
1,9	336,57
2	327,21
2,1	322,21
2,2	311,66
2,3	328,32
2,4	332,67
2,5	294,61
2,6	306,94
2,7	296,38
2,8	273,55

0,00000224	1,65328419	1,648028666
0,0000024	1,652734985	1,647314237
0,00000256	1,650882949	1,646600597
0,00000288	1,647495279	1,645175677
0,0000032	1,647635231	1,643753898
0,00000352	1,643829566	1,642335253
0,00000384	1,644521667	1,640919736
0,00000416	1,640989709	1,639507339
0,00000448	1,642003068	1,638098057
0,0000048	1,638085899	1,636691881
0,00000512	1,638060257	1,635288805
0,00000576	1,632804467	1,632491926
0,0000064	1,630312112	1,629707365
0,00000704	1,627716414	1,626935069
0,00000768	1,624453244	1,624174983
0,00000832	1,621748349	1,621427052
0,00000896	1,61740393	1,618691225
0,0000096	1,614498134	1,615967447
0,00001024	1,611829757	1,613255665
0,00001152	1,605029853	1,60786788
0,0000128	1,598450041	1,602527449
0,00001408	1,593910743	1,597233956
0,00001536	1,587393122	1,591986989
0,00001664	1,582916889	1,586786138
0,00001792	1,577534351	1,581630999
0,0000192	1,5717697	1,57652117
0,00002048	1,566013203	1,571456252
0,00002304	1,556359374	1,561459575
0,0000256	1,545181083	1,551637851
0,00002816	1,535654204	1,541988019
0,00003072	1,525466982	1,532507071
0,00003328	1,516780016	1,52319205
0,00003584	1,506524771	1,514040054
0,0000384	1,497436373	1,505048228
0,00004096	1,488663001	1,496213769
0,00004608	1,471175852	1,479005987

2,9	289
3	298,16
3,1	322,49
3,2	309,59
3,3	331,81
3,4	329,52
3,5	316,13
3,6	315,54
3,7	315,71
3,8	302,73
3,9	312,77
4	286,65
4,1	302,83
4,2	273,57
4,3	296,85
4,4	268,35
4,5	287,27
4,6	304,85
4,7	301,77
4,8	295,5
4,9	287,25
5	290,32
5,1	305,94
5,2	286,52
5,3	315,7
5,4	317,92
5,5	297,42
5,6	279,65
5,7	299,17
5,8	311,96
5,9	280,15
6	300,56
6,1	298,06
6,2	290,76
6,3	265,95
6,4	291,54

0,0000512	1,455550715	1,462395247	6,5	315,62
0,00005632	1,440159479	1,446360834	6,6	299,2
0,00006144	1,426098234	1,430882753	6,7	302,49
0,00006656	1,411507216	1,4159417	6,8	308,33
0,00007168	1,398332768	1,401519043	6,9	297,76
0,0000768	1,385184227	1,387596796	7	284,61
0,00008192	1,373417611	1,374157595	7,1	297,61
0,00009216	1,350122259	1,348661879	7,2	325,9
0,0001024	1,328858053	1,324904673	7,3	312,02
0,00011264	1,309152958	1,302767431	7,4	322,97
0,00012288	1,290579683	1,28213969	7,5	318,55
0,00013312	1,274361995	1,262918521	7,6	313,24
0,00014336	1,258104082	1,245008011	7,7	299,15
0,0001536	1,243660319	1,228318788	7,8	286,32
0,00016384	1,229912022	1,212767576	7,9	282,68
0,00018432	1,205028144	1,184774077	8	271,06
0,0002048	1,183718434	1,160468077	8,1	289,74
0,00022528	1,164868663	1,139363834	8,2	283,46
0,00024576	1,14828073	1,12103959	8,3	282,73
0,00026624	1,133292575	1,105129143	8,4	285,5
0,00028672	1,119800981	1,091314532	8,5	311,72
0,0003072	1,108422828	1,079319678	8,6	321,65
0,00032768	1,098512103	1,068904871	8,7	296,7
0,00036864	1,08071274	1,052010272	8,8	313,83
0,0004096	1,065793835	1,039273467	8,9	310,99
0,00045056	1,054387054	1,029671216	9	293,66
0,00049152	1,045047564	1,022432098	9,1	303,54
0,00053248	1,036986413	1,016974541	9,2	296,9
0,00057344	1,029899606	1,0128601	9,3	308,76
0,0006144	1,024566464	1,009758232	9,4	295,29
0,00065536	1,020259257	1,007419739	9,5	312,69
0,00073728	1,013630497	1,004327643	9,6	313,93
0,0008192	1,008516975		9,7	274,11
0,00090112	1,005278761		9,8	296,07
0,00098304	1,003127479		9,9	264,24
0,00106496	1,00251579		10	309,59

0,00114688	1,001878526	10,1	299
0,0012288	1,001418132	10,2	296,45
0,00131072	1,000911117	10,3	325,83
0,00147456	1,000908706	10,4	297,79
0,0016384	1,000599508	10,5	269,53
0,00180224	1,000379225	10,6	281,47
0,00196608	0,999803589	10,7	293,66
0,00212992	1,00056424	10,8	311,73
0,00229376	1,000364605	10,9	320,81
0,0024576	1,001658727	11	299,36
0,00262144	1,002948341	11,1	306,09
0,00294912	1,00260238	11,2	309,06
0,0032768	1,002557308	11,3	312,64
0,00360448	1,003390074	11,4	302,87
0,00393216	1,001125366	11,5	323,62
0,00425984	1,001752154	11,6	307,42
0,00458752	1,002669763	11,7	311,63
0,0049152	1,002264056	11,8	287,62
0,00524288	1,000939136	11,9	292,47
0,00589824	0,999353	12	282,86
0,0065536	0,998774395	12,1	261,53
0,00720896	1,000205749	12,2	295,7
0,00786432	1,000916635	12,3	314,22
0,00851968	0,999615936	12,4	328,59
0,00917504	1,000979463	12,5	299,36
0,0098304	0,999691974	12,6	304,98
0,01048576	0,999655373	12,7	292,85
0,01179648	1,00263728	12,8	274,58
0,0131072	1,003963788	12,9	295,1
0,01441792	1,001224896	13	297,29
0,01572864	0,999040475	13,1	290,84
0,01703936	1,000910927	13,2	308,54
0,01835008	1,000628513	13,3	339,45
0,0196608	0,999703103	13,4	310,67
0,02097152	1,000453671	13,5	295,07
0,02359296	1,000461977	13,6	330,44

0,0262144	0,999992061	13,7	292,57
0,02883584	0,999844515	13,8	291,94
0,03145728	1,000507244	13,9	277,4
0,03407872	1,00073913	14	300,73
0,03670016	1,002154697	14,1	303,75
0,0393216	1,000865134	14,2	306,88
0,04194304	1,001621354	14,3	287,17
0,04718592	0,999960222	14,4	308,93
0,0524288	1,001189853	14,5	303,87
0,05767168	1,001066036	14,6	293,12
0,06291456	1,000990734	14,7	307,92
0,06815744	1,000714538	14,8	326,6
0,07340032	1,000796782	14,9	304,51
0,0786432	0,999055429	15	319,88
0,08388608	1,000877845	15,1	300,24
0,09437184	1,000577629	15,2	305,26
0,1048576	1,000256103	15,3	312,67
0,11534336	1,001081238	15,4	340,11
0,12582912	1,000264128	15,5	327,73
0,13631488	1,000818046	15,6	331,09
0,14680064	1,000491711	15,7	321,03
0,1572864	1,000626688	15,8	313,22
0,16777216	1,000173221	15,9	317,03
0,18874368	1,000477692	16	323,01
0,2097152	1,000294744	16,1	319,25
0,23068672	0,999996351	16,2	321,34
0,25165824	1,000286145	16,3	305,55
0,27262976	0,999995196	16,4	322,64
0,29360128	1,000239804	16,5	326,81
0,3145728	1,000097501	16,6	321,77
0,33554432	0,99995863	16,7	307,1
0,37748736	0,999485047	16,8	290,63
0,4194304	0,998803562	16,9	291,55
0,46137344	0,998857583	17	327,56
0,50331648	0,998558987	17,1	329,47
0,54525952	0,99837162	17,2	345,02

0,58720256	0,998870862
0,6291456	0,999166935
0,67108864	0,99947172
0,75497472	0,999851334
0,8388608	1,000423713
0,92274688	1,000271322
1,00663296	1,000219376
1,09051904	1,001075354
1,17440512	1,000985685
1,2582912	1,001109818
1,34217728	1,000088833
1,50994944	0,999772357
1,6777216	0,999261119
1,84549376	0,998493516
2,01326592	0,999638768
2,18103808	0,9999268
2,34881024	0,999187095
2,5165824	0,997806347
2,68435456	0,995362757
3,01989888	0,998192526
3,3554432	0,996081342
3,69098752	0,995953137
4,02653184	0,996482971
4,36207616	0,997386223
4,69762048	1,000022457
5,0331648	0,999165177
5,36870912	1,003826857
6,03979776	1,002741787
6,7108864	1,007538194
7,38197504	1,004035649
8,05306368	0,993868315

17,3	347,46
17,4	299,73
17,5	309,19
17,6	324,74
17,7	356,88
17,8	331,16
17,9	315,16
18	314,07
18,1	318,94
18,2	309,01
18,3	317,11
18,4	294,56
18,5	306,8
18,6	323,59
18,7	334,81
18,8	283,44
18,9	318,3
19	280,94
19,1	293,24
19,2	313,84
19,3	304
19,4	308,24
19,5	341,03
19,6	327,56
19,7	345,69
19,8	340,82
19,9	318,62
20	342,24
20,1	323,41
20,2	327,82
20,3	328,25
20,4	324,08
20,5	309,57
20,6	301,53
20,7	325,46
20,8	320,68

20,9	291,35
21	335,95
21,1	307,23
21,2	335
21,3	309,18
21,4	292,17
21,5	325,23
21,6	311,07
21,7	291,9
21,8	296,41
21,9	299,1
22	298,89
22,1	317,19
22,2	301,11
22,3	313,64
22,4	321,19
22,5	293,32
22,6	323,9
22,7	276,17
22,8	287,5
22,9	286,9
23	285,47
23,1	317,63
23,2	336,53
23,3	295,89
23,4	323,61
23,5	301,64
23,6	303,98
23,7	298,99
23,8	322,14
23,9	305,56
24	314,32
24,1	301,5
24,2	311,17
24,3	312,09
24,4	288,14

24,5	292,2
24,6	311,68
24,7	338,67
24,8	312,49
24,9	328,01
25	308,58
25,1	336,55
25,2	285,21
25,3	324,35
25,4	322,62
25,5	317,88
25,6	313,99
25,7	336,9
25,8	328,38
25,9	302,71
26	293,95
26,1	320,37
26,2	334,12
26,3	338,5
26,4	302,37
26,5	325,71
26,6	343,18
26,7	298,39
26,8	342,62
26,9	346,28
27	325,01
27,1	347,74
27,2	316,75
27,3	313,96
27,4	333,22
27,5	323,54
27,6	326,43
27,7	311,68
27,8	315,13
27,9	307,83
28	328,32

28,1	334,39
28,2	331,72
28,3	333,35
28,4	297,02
28,5	292,13
28,6	308,28
28,7	300,18
28,8	295,53
28,9	331,07
29	318,96
29,1	305,49
29,2	301,52
29,3	298,59
29,4	315,62
29,5	349,42
29,6	302,22
29,7	290,22
